# Evaluation of autoantibody signatures in meningioma patients using human proteome arrays

**DOI:** 10.18632/oncotarget.16997

**Published:** 2017-04-10

**Authors:** Shabarni Gupta, Shuvolina Mukherjee, Parvez Syed, Narendra Goud Pandala, Saket Choudhary, Vedita Anand Singh, Namrata Singh, Heng Zhu, Sridhar Epari, Santosh B Noronha, Aliasgar Moiyadi, Sanjeeva Srivastava

**Affiliations:** ^1^ Department of Biosciences and Bioengineering, Indian Institute of Technology Bombay, Mumbai, India; ^2^ Department of Biochemistry/Biotechnology, University of Turku, Turun yliopisto, Finland; ^3^ Department of Chemical Engineering, Indian Institute of Technology Bombay, Mumbai, India; ^4^ Molecular and Computational Biology, Department of Biological Sciences, University of Southern California, Los Angeles, CA, USA; ^5^ Department of Pharmacology and Molecular Sciences/High-Throughput Biology Center, Johns Hopkins University School of Medicine, Baltimore, MD, USA; ^6^ Department of Pathology, Tata Memorial Centre, Mumbai, India; ^7^ Department of Neurosurgery, Tata Memorial Centre, Mumbai, India

**Keywords:** meningioma, autoantibody, protein array, brain tumors, HuPort screening

## Abstract

Meningiomas are one of the most common tumors of the Central nervous system (CNS). This study aims to identify the autoantibody biomarkers in meningiomas using high-density human proteome arrays (~17,000 full-length recombinant human proteins). Screening of sera from 15 unaffected healthy individuals, 10 individuals with meningioma grade I and 5 with meningioma grade II was performed. This comprehensive proteomics based investigation revealed the dysregulation of 489 and 104 proteins in grades I and II of meningioma, respectively, along with the enrichment of several signalling pathways, which might play a crucial role in the manifestation of the disease. Autoantibody targets like IGHG4, CRYM, EFCAB2, STAT6, HDAC7A and CCNB1 were significantly dysregulated across both the grades. Further, we compared this to the tissue proteome and gene expression profile from GEO database. Previously reported upregulated proteins from meningioma tissue-based proteomics obtained from high-resolution mass spectrometry demonstrated an aggravated autoimmune response, emphasizing the clinical relevance of these targets. Some of these targets like SELENBP1 were tested for their presence in tumor tissue using immunoblotting. In the light of highly invasive diagnostic modalities employed to diagnose CNS tumors like meningioma, these autoantibody markers offer a minimally invasive diagnostic platform which could be pursued further for clinical translation.

## INTRODUCTION

**Figure 1:**
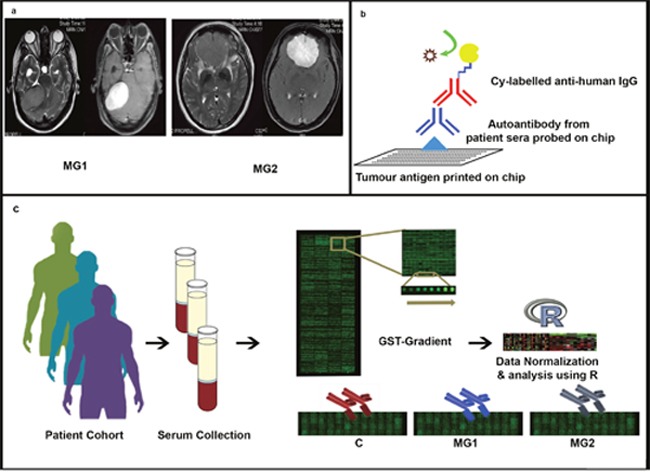
Experimental Design **(Panel a)** shows the MR images of MG1 (Right posterior fossa meningioma. Left – Axial T2 images and Right – Axial T1 post contrast images) and MG2 (Anterior cranial fossa base meningioma. Left – Axial T2 images and Right- Axial T1 post contrast images), **(Panel b)** shows the interactions and molecular design of the microarray experiment while **(Panel c)** is a schematic of the experimental design where serum samples are collected from each of the three representative cohorts (Control, MG1 and MG2) and are subjected to protein microarray assay. This is followed by stringent quality checks, data normalization and analysis using R.

Meningiomas are one of the most commonly reported brain tumors comprising of nearly 30% of all primary central nervous system tumors [1]. The WHO classifies meningiomas as benign Grade I (MG1), atypical Grade II (MG2), (Figure 1a) and anaplastic Grade III [2]. The current diagnostics include imaging techniques like computerized tomography (CT) scan and magnetic resonance imaging (MRI) for the detection of the tumour and surgical resection thereafter [3]. However, it does not take into account the possibilities of recurrence and prevalence of malignancy. Early detection is challenging because meningiomas, which essentially are benign, may remain asymptomatic for years without causing any complications. However, in case of further growth it can cause neurological symptoms related to brain and cranial nerve compression or irritation [4]. Thus, several recent studies have aimed to identify various genetic and molecular alterations of meningioma so as to identify new molecular markers and targeted therapies for early diagnosis and better clinical management of the disease [5–7].

Any neoplasm is a manifestation of de-regulation and alterations at the cellular level, which involves multiple essential components that are responsible for homeostasis and proliferation [8]. This alteration evokes an immune response against the tumor cells which leads to production of several antigens that are either significantly altered, over-expressed or present at the basal levels in normal cells [9]. These are called as tumor specific antigens (TSA) or tumor associated antigens (TAA). Several TAAs which have been associated with various malignancies have been identified and have yielded mechanistic insight into the tumor biology, including PSA (Prostate Specific Antigen) for prostate cancer, CA-125 for ovarian cancer and fibrinogen for bladder cancer [10–12]. Detection of the altered proteins is often challenging due to rapid degradation of the proteins. Anti-TAA antibodies or autoantibodies, on the other hand are specific as well as stable and can easily be detected from the patient sera using several platforms [13]. Thus, detecting ‘autoantibodies’ have emerged as one of the most promising approaches for early diagnosis of malignancies and can be used as sensors to comprehend events associated with tumorigenesis [14, 15].

Studies have pointed out the fact that various grades of meningiomas elicit a complex immune response [16]. In fact, there evidence of an active immune surveillance mechanism targeting the benign tumor much before the onset of malignancy [17]. Thus, identification of specific autoantibody signature in meningiomas could potentially enable early diagnosis of the onset of tumorigenesis [18]. Autoantibody screening in meningiomas have been performed using different platforms like serological analysis of expression cDNA libraries (SEREX) recombinant clones in customized protein arrays [16, 19, 20]. While the above mentioned studies have been successful in identifying several antigens that were specifically binding to meningioma sera, there is a dearth of studies where autoantibodies have been screened for reactivity on full-length proteins (Figure 1b). Furthermore, several other studies which have used mass spectrometry based platforms have pointed out dysregulation in components of Integrin pathway, VEGF pathway and have indicated differential expression of certain proteins in meningioma patients [21–23].

In this study, we have used the Human Proteome Array (HuProt™) [24] to identify autoantibody signatures in meningioma patient cohorts of grade I and II. HuProt™ arrays possess one of the largest collections of full-length human proteins, which are expressed in yeast and thus provide a unique platform for identifying a large number of autoantibodies generated in response to TAAs (Figure 1c). The immunogenicity of ~17000 proteins on the HuProt™ arrays was analysed with 10 MG1 and 5 MG2 meningioma subjects and comparison was performed using 15 healthy subjects. We analysed major perturbed pathways in meningiomas, which may play a vital role in the manifestation and progression of the disease. Further, to correlate the antigenicity of the proteins from this study we compared our existing tissue and serum proteome data obtained from high-resolution mass spectrometry with this autoantibody data [25, 26]. A correlation between the protein levels and their autoantibody levels can highlight some potential candidates for early diagnostic biomarkers at protein and antibody levels. This analysis has been extended to the publically available data repositories like Gene Expression Omnibus (GEO) to co-relate autoantibody responses further from protein to their gene expression [27]. This study is one of the first comprehensive proteome-wide investigations of meningioma patients to detect autoantibodies generated in response to various cellular alterations associated with onset of malignancy in meningioma patients.

## RESULTS

### Differential expression analysis of autoantibodies across different grades of meningioma

Differential expression analysis of autoantibodies was performed to access the extent of dysregulation of autoimmune response towards putative tumor antigens using protein microarray assay. Proteins which showed a log_2_ fold-change greater than 0.5 or less than -0.5 and had an adjusted p-value less than 0.05 were considered as differentially expressed while comparing healthy controls with different grades of meningioma. Upon comparing the data from healthy controls and MG1 samples, we identified 489 significant autoantibodies (Supplementary Table 1). Similarly, comparison of healthy controls with MG2 resulted in the identification of 104 autoantibodies (Supplementary Table 2). When the entire set of meningioma samples (MG which includes all patients from MG1 and MG2) were compared against healthy controls a set of 203 proteins were found differentially expressed (Supplementary Table 3). The detailed list of differentially expressed proteins and their corresponding log fold-change can be found in the Supplementary Tables 1, 2 and 3.

Upon using a higher threshold; log_2_ fold change cut off of greater than or equal to 1 with a corrected p-value less than 0.05; we found a set of 21 significantly dysregulated autoantibodies in MG vs HC, similarly 20 autoantibodies that were dysregulated in MG1 vs HC and 23 autoantibodies in MG2 vs HC (Table 1, Supplementary Table 4.1). Among these, EFCAB2, EPS8L1, LOC285382 proteins were found to be upregulated while a majority of others like IGHG4, CRYM, TIRAP, C17orf57 were found to be downregulated in all the comparisons. We documented the signal intensities of each of these proteins qualitatively by observing each feature for its morphology (Supplementary Figure 1). The intensities of each protein were visualized using box plots and their ability to distinguish between cohorts was assessed using heat maps (Supplementary Figures 2 and 3).

**Table 1:** Significantly dysregulated proteins across all comparisons

ID	Symbol	logFC across various cohorts in comparison to HC		
		MG	MG1	MG2
BC025985.1	IGHG4	-3.1	-3.1	-3.1
NM_001014444.1	CRYM	-1.4	-1.4	-1.4
NM_032328.1	EFCAB2	1.1	1.1	1.1
BC065370.1	C20orf112	-2.0	-1.8	-2.2
BC037876.1	C17orf57	-1.6	-1.6	-1.7
NM_001033515.1	LOC389833	-1.1	-1.1	-1.1
NM_005719.2	ARPC3	-1.2	NS	-1.5
NM_002767.2	PRPSAP2	-1.2	NS	-1.5
NM_001005465.1	OR10G3	-1.3	-1.4	-1.2
NM_139204.1	EPS8L1	1.3	1.2	1.3
NM_001025266.1	LOC285382	1.4	1.3	1.4
NM_014372.3	RNF11	-1.3	NS	-1.5
NM_148910.2	TIRAP	-1.1	-1.1	-1.1
NM_198086.1	JUB	-1.3	-1.1	-1.5
XM_290842.4	LRFN1	-1.1	-1.0	-1.2
NM_021810.3	CDH26	-1.0	-1.0	NS
BC090880.1	EIF3S3	-1.1	-1.0	NS
NM_173809.2	BLOC1S2	-1.7	NS	-2.6
NM_016224.3	SNX9	-1.1	-1.1	NS
Nol3	Nol3	-1.4	NS	-1.9
Lhx1	Lhx1	-1.0	NS	NS
NM_031304.2	DOHH	NS	-1.0	NS
NM_015726.2	WDR42A	NS	-1.3	NS
BC006453.1	HDAC7A	NS	1.0	NS
NM_002893.2	RBBP7	NS	-1.0	NS
NM_018584.4	CAMK2N1	NS	-1.0	NS
NM_004264.2	SURB7	NS	NS	-1.0
NM_182789.2	PAIP1	NS	NS	1.1
NM_001042476.1	CARHSP1	NS	NS	-1.1
NM_003099.3	SNX1	NS	NS	1.1
NM_001033112.1	PAIP2	NS	NS	1.0
BC013992.1	MAPK3	NS	NS	1.1

'NS' denotes a given protein as statistically not significant in that comparison. Expanded form of this table can be found in Supplementary Table 4.

### Correlation of autoantibody response with the tissue proteome

Global proteomic profiling of different grades of meningiomas was done using high throughput mass spectrometry platform [25]. This enabled identification of several differentially expressed proteins across different grades of meningioma. Comparison of the output with the autoantibodies that were screened in meningioma patients yielded 25 common proteins that had been found to be up or downregulated using mass spectrometry based study and generated autoantibody response as well (Table 2). This includes proteins like SELENBP1, TPD52L2, GSTP1, C11orf67, RPS13, FABP5, PDXK, CRYM, APOE, COX4I1, MARCKSL1, EPB41L3, RTN4, QDPR, HSPA2, PPP2R4, NME1, ACO2, YWHAB, C21orf33, VCP, RNPEP, ALDH9A1, CARHSP1 (Table 2). Many of these proteins have similar trends in terms of their antigenicity in the microarray platform which can be correlated to their mass spectrometry based quantitative data.

**Table 2:** Trends of proteins common in mass spectrometric analysis and correlation to the autoantibody response

**Proteins upregulated in MS with elevated autoantibody response**					
**Sr. No**.	**Gene symbol**	**MG1**		**MG2**	
		**Fold change in tissue proteomics**	**Fold change in autoantibody response**	**Fold change in tissue proteomics**	**Fold change in autoantibody response**
1	GSTP1	1.62	0.51	2.32	NS
2	C11orf67	1.77	0.52	2.38	NS
3	RPS13	1.81	0.51	1.46	NS
4	SELENBP1	1.55	0.51	2.14	NS
5	FABP5	1.79	0.55	3.83	NS
6	TPD52L2	1.35	0.60	2.54	NS
7	PDXK	0.83	0.53	0.87	NS
**Proteins downregulated in MS with downregulated autoantibody response**					
**Sr. No**.	**Gene symbol**	**MG1**		**MG2**	
		**Fold change in tissue proteomics**	**Fold change in autoantibody response**	**Fold change in tissue proteomics**	**Fold change in autoantibody response**
9	CRYM*	0.10	-1.44	0.13	-1.43
10	APOE	0.53	-0.54	0.61	NS
11	COX4I1	0.17	-0.51	0.25	-0.62
12	MARCKSL1	0.30	-0.64	0.42	NS
13	EPB41L3	0.48	-0.53	0.47	NS
14	RTN4	0.60	-0.62	0.65	NS
15	QDPR	0.21	-0.52	0.23	NS
16	HSPA2	0.28	-0.70	0.32	NS
**Proteins with opposite trends in MS and autoantibody response**					
**Sr. No**.	**Gene symbol**	**MG1**		**MG2**	
		**Fold change in tissue proteomics**	**Fold change in autoantibody response**	**Fold change in tissue proteomics**	**Fold change in autoantibody response**
17	PPP2R4	0.581	0.69	0.55	0.60
18	NME1	0.624	0.69	1.40	NS
19	ACO2	0.602	0.57	0.53	NS
20	YWHAB	0.393	0.67	0.37	NS
21	C21orf33	0.516	0.50	0.80	NS
22	VCP	1.558	-0.61	1.13	NS
23	RNPEP	2.060	-0.52	1.76	NS
24	ALDH9A1	1.396	-0.63	1.54	NS
25	CARHSP1*	1.191	-0.81	2.21	-1.13
26	UBE2V2	0.37	NS	0.40	0.66

The table denotes partial list of the significant autoantibody signatures across meningioma patients with absolute log FC ≥0.5 which has been considered to be statistically significant along with the tissue proteomic levels. ‘NS’ denotes a given protein as statistically not significant in that comparison. Fold changes in the color green denote upregulation of a given protein in tissue proteome or autoantibody while red indicates a given value of fold change to be downregulated for a given comparison. "*" indicates the availability of trends from GEO dataset which have been elaborated in Supplementary Table 4.

Autoantibody profiles were also compared to serum proteomic profiles of meningioma patients using mass spectrometry by Sharma et al.[26]. The comparison of the various altered proteins from patient sera and the autoantibody signatures yielded common trends in two of the proteins, which include IGHG4 and IGHD. Both of these proteins are members of the immunoglobulin family and have shown a decreased autoantibody response in meningioma patients. The protein levels were also lower in comparison to healthy individuals (Supplementary Table 4.2).

### Comparison with gene expression profiling

**Figure 2:**
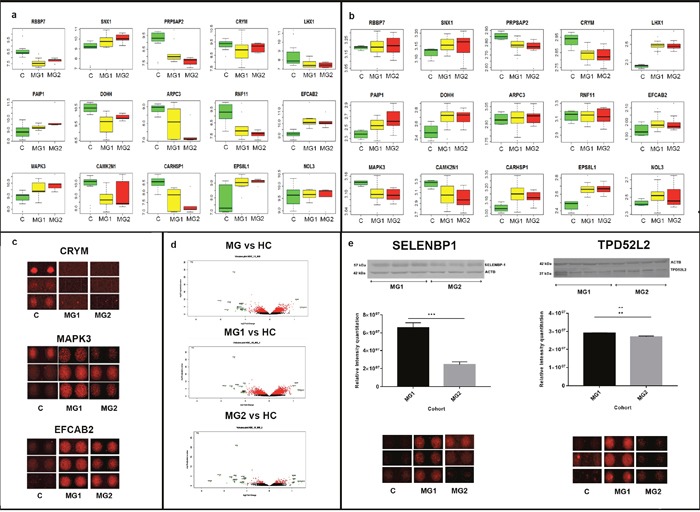
Significantly dysregulated autoantibodies **(Panel a and b)** represent box-plots of 15 targets commonly emerging from the HuProt based autoantibody data in this study and GEO dataset meta-analysis, respectively. **(Panel c)** contains three most significant targets across the above platforms with their feature intensities in protein microarrays. **(Panel d)** represents volcano plots of the proteins emerging from the protein microarray dataset which highlights several of the above targets. **(Panel e)** shows the immunoblot of two proteins SELENBP1 and TPD52 along with their feature intensity in protein microarrays. Quantitation of the blots was done using IQTL and their p-value was less than 0.05 using two-tailed unpaired t-test. ** indicates a p value of 0.0045 and *** indicates a p value of 0.0002. This shows the possible co-relation of the antigenicity due to elevated levels of a protein to their autoantibody response.

We also compared the autoantibody profiles obtained from our experiments with the gene expression profiles obtained from GSE43290 on GEO (Table 2, Figure 2a, Supplementary Table 4.1) [27]. Of 32 unique proteins that were significant across all comparisons in our study (Table 1), gene expression profiles of 15 proteins were available. The corresponding data was analyzed, expression profiles were plotted (Figure 2b) and compared with the expression profiles obtained from proteome array (Figure 2a). The trends of gene expression profiles of candidates such as EFCAB2, EPS8L1, NOL3, PAIP1 and SNX1 showed elevated expression in meningiomas compared to controls, while CAMK2N1, CRYM and PRPSAP2 showed decreased expression levels in meningiomas compared to the controls. The trends of gene expression profiles followed their protein expression profiles. On the other hand, RBBP7, ARPC3 and RNF11 did not show any significant difference in the gene expression profiles of meningiomas and the controls. However, their corresponding proteins were downregulated in meningiomas. The gene expression profiles of CARHSP1, DOHH and LHX1 was up-regulated, while their corresponding protein expression was downregulated. Also, the gene expression profiling of MAPK3 showed downregulation, while the protein was upregulated in meningiomas (Figure 2). EFCAB2 and CRYM showed similar trends across the gene expression profile trends and autoantibody response (Figure 2)

### Validation of SELENBP1 and TPD52 using immunoblotting

SELENBP1 and TPD52 were two promising candidates emerging from the cross comparison of the autoantibody data (Table 2) with tissue proteome profiles with elevated levels across both platforms [25]. In order to assess whether the antigenic levels of SELENBP1 and TPD52L2 are altered, we used immunoblotting using tissue lysates. While SELENBP1 showed a two-fold increase in MG1 as compared to MG2, TPD52L2 showed a response of one fold response in MG1 as compared to MG2. The loading control for normalization of the data was beta-actin in both cases. The densitometric analysis of SELENBP1 and TPD52L2 when analysed via Image Quant TL (IQTL) software (GE Healthcare) and GraphPad Prism version 7 yielded p-values less than 0.05. These indicate elevated levels of these proteins in meningioma tissue samples do alter amongst the grades and could lead to autoantibody production (Figure 2c, Supplementary Tables 4.3 and 4.4)

### Pathway analysis

**Figure 3:**
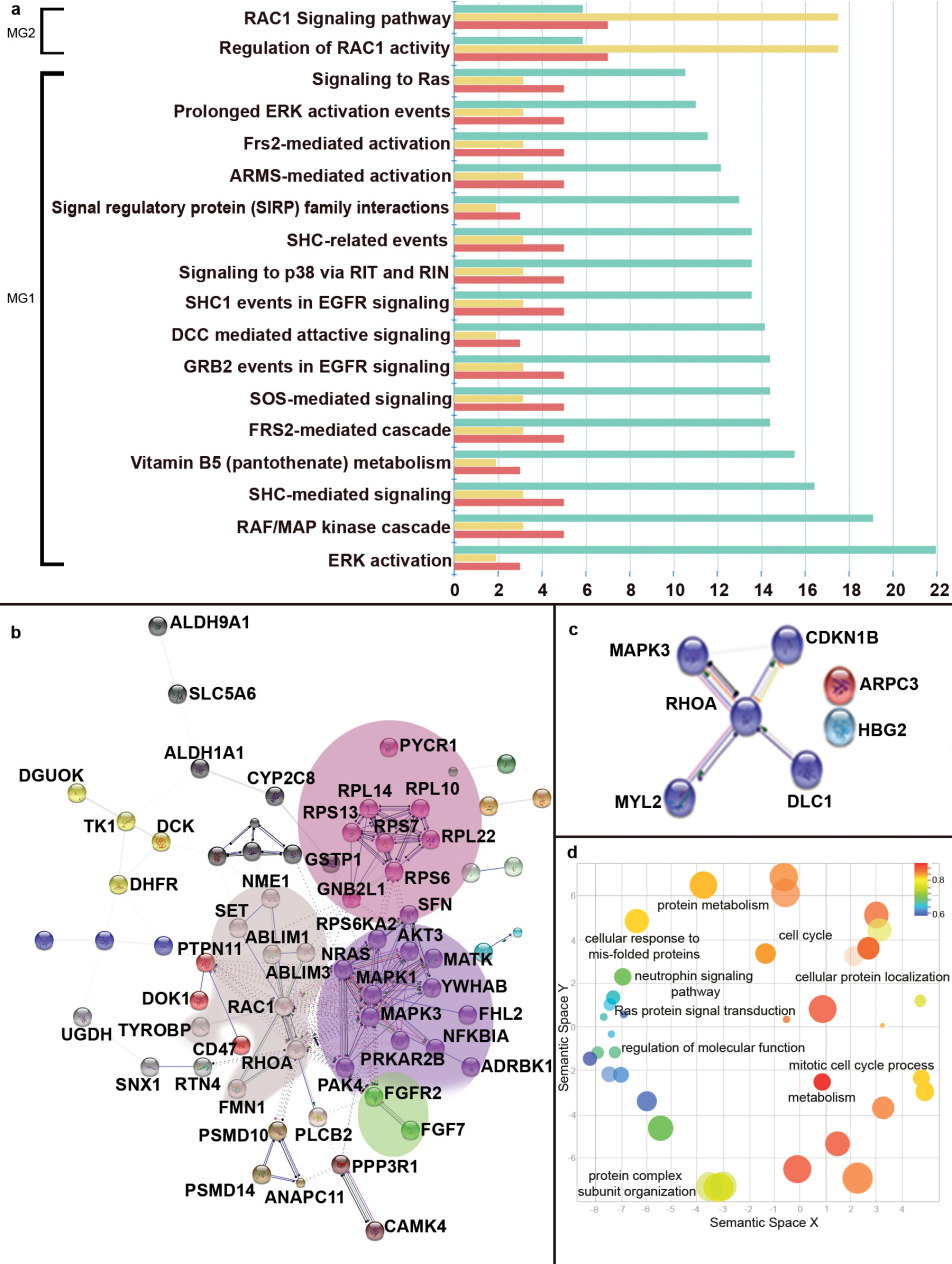
Enrichment analysis of significantly dysregulated autoantibodies **(Panel a)** shows the dysregulated pathways emerging from HC vs MG1 and HC vs MG2 analysis. Green bars represent the fold enrichment of each pathway, yellow represent the percentage of genes and red represent the number of genes in the dataset. **(Panel b)** represents the protein interaction networks from proteins implicated in HC vs MG1 pathways. **(Panel c)** represents the protein interaction networks from proteins implicated in HC vs MG2 pathways. **(Panel d)** is the scatter plot of GO terms emerging from HC vs MG1 redrawn from REVIGO.

Significant proteins with corrected p-value less than 0.05 and absolute log_2_ fold change greater than 0.5 were subjected to enrichment analysis using FunRich: Functional Enrichment Analysis Tool [28]. 48 pathways were found to be significantly dysregulated in MG1 when compared to controls (Supplementary Table 5). These predominantly represented signaling pathways like RAF/MAP kinase cascade, EGFR signaling, osteopontin-mediated events, signaling by NGF, signaling to RAS (Figure 3a). Proteins like NRAS, MAPK3, YWHAB, PTPN11, MAPK1 were implicated in majority of these pathways. RAC1 signaling pathways were found to be significantly dysregulated in MG2 using this enrichment analysis implicating proteins like ARPC3, MAPK3, CDKN1B, DLC1, RHOA, MYL2, HBG2 (Supplementary Table 6) (Figure 3a). There were no significant pathways enriched when MG was taken as a cohort (Supplementary Table 7). On performing a network clustering on proteins enriched in pathway analysis from our data using MCL algorithm on STRING DB v10.0 [29], MG1 gave 4 distinct clusters of protein interactions (Figure 3b, Supplementary Figure 5). Proteins implicated in MG2 gave one distinct cluster (Figure 3b). GeneOntology Terms (GO) enriched from String DB (Supplementary Table 8) was exported to REVIGO [30] for visualization, clustering and removal of redundant GO terms from the data (Figure 3c, Supplementary Figure 6). The above annotations highlighted 4 clusters; signalling pathways, cell cycle, protein organization and metabolism related GO terms. Details of GO terms and clusters can be found in Supplementary Table 8, Supplementary Figures 5 and 6.

## DISCUSSION

Meningiomas are one of the most common CNS tumors with several risk factors like age, gender, ionizing radiation etc. associated with them [17, 18]. Current diagnostic approaches towards detecting most CNS tumors are primarily radiology-based modalities like CT or MRI scans. These diagnoses are further confirmed by histolopathology and then categorized by sequencing and IHC based markers. Early diagnosis of meningioma continues to remain a challenge, as radiology based techniques are not able to detect the tumors, until the tumor is sizeable, which adds to the prognostic risk of the patients. Meningiomas are also highly heterogeneous, where parameters like brain invasion confer grade II like recurrence and mortality rates on to low-grade meningiomas [2]. Autoantibody production provides an ideal minimally invasive platform which enable early diagnosis of patient even before the tumor grows to a sizeable extent. With the onset of malignancy, the immune system triggers production of antibodies against TAAs, which when screened can prove to be an indispensable resource to help early diagnosis in parallel to other existing modalities of treatment.

In this study, we performed autoantibody screening of meningioma patient sera against ~17,000 full-length proteins. Among the most dysregulated autoantibodies, a group of 10 proteins were detected across all grades of meningiomas. These included CRYM, which is a thyroid hormone T3 binding protein and also has been reported to be androgen regulated [31]. Its downregulation is interesting as meningiomas occur predominantly in females. Few other candidates like EFCAB2, which is a calcium binding protein [32]; TIRAP, which is a toll-interleukin receptor protein [33]; and JUB, which is involved in cell-cell adhesion [34], were also found to exhibit a differential autoantibody response with high fold changes (Table 1). Of the many elevated autoantibody targets; significant response against EFCAB2, HDAC7A, and EPS8L1 were seen. EPS8L1, a substrate of EGFR, plays an important role in modulating Rho and Ras mediated signal transduction [35]. It is involved in T-cell receptor binding and has been implicated in carcinogenesis [36]. Furthermore, several components of cell cycle and differentiation were also found to illicit an autoimmune response, namely CCNB1, CKS2 and DRG1. DRG1 is known as NEDD-3 or neural precursor cell expressed developmentally down-regulated protein-3, which was found to elicit autoimmune response in patient sera in our study. DRG1 has also been reported to display antigenic response and was recognized by HLA-DR11-restricted CD4+ Th1 cells [37]. A few other studies have also reported DRG1 to be a tumour suppressor with role in suppression of metastasis in prostate and breast cancer [38, 39]; however, an exact role and relevance of this protein in meningiomas is not known. Apart from the aberrations in cell cycle checkpoints, our study demonstrated that many of the autoantibodies found to be perturbed were components of calcium homeostasis maintenance like HPCAL1, ANXA11, CALCOCO2, CAMK2N1, GADD45A, CDC34 [40]. The calcium concentration often dictates several transcriptional factors, which in turn regulate cell differentiation [41]. The HPCAL1, a neuronal calcium sensor protein which is found in brain [42], was found to generate significant autoantibody levels in meningioma patients. This protein has also been reported to be a good target for monoclonal immunotherapy in cases of pancreatic cancer patients [43]. NME1, NFE2, SFN, PTN, CALN1, GABRA5 and several components involved in neural differentiation and receptors associated with neural transmitter (Supplementary Table 5) were also found to illicit an autoimmune response in the meningioma patients indicating that there could be some alterations in these components. NME1, a nucleoside diphosphate kinase 1, which determines neural patterning and cell fate and is also known to be a metastasis suppressor gene, displayed an elevated autoimmune response in our study [44]. Significant alterations were also seen in interacting partners of NME1 namely ABLIM1, ABLIM3, RAC1 and SET (Figure 3b).

Proteins emerging from the autoantibody screening were enriched in key signaling cascades that are likely to be perturbed in meningioma patients. While RAF/MAP kinase cascade was found to be most significant in Grade I, the involvement of RAC1 signaling cascade was found to be prominent in Grade II. NRAS and MAPK3 were some of the key proteins mapped to these pathways forming the focus of two protein interaction network clusters (Figure 3). EGFR signaling which has earlier been reported in context to meningiomas were also found to be perturbed along with several other growth factor mediated signaling namely FGF signaling pathway, NGF mediated signaling, PDGF signaling and signaling by insulin receptors [22, 45, 46]. Some of the major components that were found to be involved includes FGF7 and FGFR2, and were also part of an interaction network (Figure 3c). Recent studies have suggested the role of ribosomal protein like RPL10, RPL5 in onset and progression of malignancy [47]. In our study, a cluster of ribosomal proteins namely the RPL10, RPL14 along with RPL14 and RPS7 were identified (Figure 3c). Thus, it can be hypothesized that meningioma patients could harbour aberrant expression or mutations in these proteins, which ultimately would lead to a differential autoantibody response (Figure 3b). The predominance of signaling pathways like Neutrophin signaling pathways, Ras signaling pathways and other cell cycle processes which have been implicated in tumorigenesis are well established from the GO terms enriched from our study as well (Figure 2d).

In order to understand the implications of autoantibody production in cancer, one must review the likely causes for autoantibody production. The underlying reasons for autoantibody production have been widely categorized into four broad cellular phenomena; (1) tolerance defects and inflammation, which includes clonal selection defects, downregulation of T regulator cells or inflammation; (2) altered protein structure, which includes mutations, post-translational modifications or exposure of neoepitopes; (3) cellular death mechanisms, which includes presentation of self-antigen peptide onto the cell surface or spillage of TAAs into the circulatory system, and (4) changes in expression level of proteins, which includes TAA over-expression or aberrant expression in an aberrant location [46]. In consideration of these factors it was interesting to compare the tissue proteome profile studied by our group with the autoantibody profile of each of these proteins. Interestingly, we found proteins like SELENBP1, TPD52L2, GSTP1, C11orf67, RPS13, FABP5, UBE2V2, PDXK which were found to be over-expressed in our mass spectrometry based tissue proteome profile, to display enhanced autoantibody response using protein microarray platform (Table 2). The presence of TPD52L2 in patient cohort was also validated using immunoblotting (Figure 2e). TPD52L2 has been extensively associated with several malignancies including gliomas [48–51]. However, the role of TPD52L2 is not well established and its clinical relevance in context to meningioma needs to be substantiated. SELENBP1 is an intracellular transporter protein for selenium [52], it has also been known to be a tumor suppressor in colon cancer [53, 54]. It seems to be highly abundant in brain and has also been implicated in psychiatric disorders [55, 56], the detection of autoantibody for SELENBP1 in context to meningiomas to the best of our knowledge has not been previously reported. Preliminary validation assays from our study shows elevated levels of both autoantibody and antigenic levels of SELENBP1 and further investigation on SELENBP1 could be clinically relevant.

Similarly, proteins like CRYM, APOE, COX4I1, MARCKSL1, EPB41L3, RTN4, QDPR, HSPA2 were found to have lowered expression in meningioma tissue using mass spectrometry and also have lower level of autoantibodies directed towards them (Table 2). However, there were a set of proteins, which seemed to have a differential trend in both the mass spectrometry and protein microarray data (Table 2). While common trends, both at the antigenic level and autoantibody response, demonstrates the significance of these proteins in meningioma, for clinical utility the data must be validated further on independent platforms. We also compared the expression profiles from our study with that of gene expression profiles. We observed a trend in the expression profiles both at protein and gene level for majority of the proteins. This highlights the role of dysregulated expression in evoking immune-response, thereby triggering autoantibody production. On the other hand, protein expression profiles of few proteins did not show any correlation with gene expression profiles (Table 2, Figure 2a). Further, we also compared the autoantibody signatures with the serum proteomic profiles of patients and came across similar trends in IGHG4 levels; autoantibodies to IGHG4 were highly downregulated in meningioma patients and the levels of the IGHG4 protein was also found to be lower in comparison to unaffected healthy individuals (Supplementary Table 4). This shows that apart from dysregulated expression at gene levels and aberrant expression of proteins, there could be several other factors which are responsible for triggering generation of autoantibody production in cancer. Recent research has also pointed out other mechanisms via which the humoral response can be evoked by cancer cells [46]. Thus, in context of biomarker discovery, these proteins seem to be interesting targets showing uniform trends across three unique platforms measuring mRNA, absolute protein level and antibody generated against the antigenic nature of a given protein. Further, we used immunoblotting for validation of protein levels of SELENBP1 and TPD52 in MG1 and MG2, which exhibited an upregulation from Grade I to II of meningioma. The ideal control for such validation study is tissue from the duramater but being an extremely fibrous membrane with less cellular content and a high collagenous extra-cellular matrix, extraction of proteins from the duramater is extremely challenging. By using the same protocol and same lysis conditions, which was used for meningioma tissue samples, the protein yield obtained from duramater sample was poor (Supplementary Figure 4). This resulted in generation of very faint bands in control as compared to the meningioma patient samples. This is a biological limitation of such clinical samples; however, if the grades of meningioma were to be compared, we observed an elevation of these proteins in MG1 as compared to MG2. The finding of enhanced autoantibody production in MG1 could be an indication of the upheaval of the immune response during the onset of the tumor with a gradual decline as the tumor progress. A similar trend was also observed in a study by Comtesse et al., wherein they have observed a decline of seroreactivity with respect to malignancy upon screening several meningioma specific antigens [17].

Thus, this study presents a preliminary insight into the autoantigenecity of several proteins, which could be pursued as minimally invasive markers. Based on our previously reported meningioma proteome profile [25], we have also looked at the correlation of the antigenicity of these TAAs with expression level of the same proteins at the tissue level, which explains the possible reasons for such observed dysregulation. Many of the signaling pathways and interactions networks involved in these pathways revealed aberration of several cell signaling pathways relevant for the meningioma disease pathobiology. This is the first study to investigate meningioma serum autoantibody response using Human Proteome Arrays and its correlation from tissue antigenic changes using high-resolution mass spectrometry, which has resulted in identification of several novel targets displaying similar trends. This study also highlights the underlying pathobiological aberrations resulting from the observed dysregulations in meningiomas and eventually paves the way for identification of clinically relevant biomarkers in meningiomas after validation in large clinical cohorts.

## MATERIALS AND METHODS

### Serum sample collection

The collection of meningioma serum samples was done at Tata Memorial Hospital (TMH), Mumbai and the samples were collected after a written consent from the individuals. This study was approved (ACTREC-TMC IEC No. 15) by the ethics committee of Advanced Centre for Treatment Research and Education in Cancer (ACTREC), Mumbai and TMH.

The collected serum samples were aliquoted into smaller volumes and stored at -80°C until further used. Prior to the assay, the serum samples were thawed on ice. Human proteome arrays (HuProt arrays) (Johns Hopkins University) were used for evaluation of serum samples from 10 meningioma grade I and 5 meningioma grade II patients, which were screened along with 10 healthy controls. A detailed description of the patient cohort and the clinical details has been documented in the Supplementary Table 1.

### Microarray fabrication

Each of the human proteome arrays used in this study consists of ~17000 unique full-length proteins printed in duplicates. These full-length proteins are expressed in yeast as N-terminal GST-His6 fusion proteins^16^. Positive controls (H2A, H2B, H3, H4 and GST in various concentrations) and negative controls (BSA, HeLa cell lysates, p300-BHC) were spotted in duplicates on the microarrays to ensure the integrity of the experiments at various steps.

### Microarray assay

The microarray chip was blocked using 3% BSA in SuperBlock (Thermo Fisher Scientific) for 2 hours on a gentle shaker. After blocking the microarray surface, to prevent the non-specific binding, we incubated the microarray with serum sample (diluted 1:500 in 2% BSA in TBST (tris buffered saline with 0.1% tween20) and anti-GST rabbit antibody (EMD Millipore, catalogue number AB3282) (diluted 1:500 in TBST) for 2 hours on shaker at room temperature. For detecting the antibodies reacting with the proteins, spotted on the microarray, we used 1:1000 dilution of anti-human IgG conjugated with Cy5 (JacksonImmunoResearch, catalogue number 109-175-064) and 1:5000 dilution anti-rabbit antibody conjugated with Cy3 (Invitrogen, catalogue number A21429). Both these antibodies were diluted in 3%BSA in SuperBlock and the microarray was incubated at room temperature for 1 hour. Each of these incubation steps were followed by a washing step which includes three-time brief rising of the microarray with TBST and then thorough washing for 4 times and each wash was for 5 minutes. After the microarray was washed, it was rinsed in distilled water and dried at 900 rpm for 2 minutes. Once the assay was performed, the microarray was scanned using GenePix 4000B Microarray Scanner (Molecular Devices).

### Statistical analysis

The scanning of the processed microarrays was performed using GenePix 4000B MicroarrayScanner (Molecular Devices). The image processing and data acquisition was done using GenePix Pro 7 (Molecular Devices). Prior to the analysis for finding differentially expressed proteins, we performed pre-processing. We make use of ‘limma’ package for pre-processing and determining differentially expressed genes [57–59].

Pre-processing is a two-step process where we perform background correction and then normalize the data. We used “nec” method present in the limma package for background correction. The “nec” method performs “normexp-by-control” background correction by taking only the negative controls into consideration. In order to reduce the variability of the log ratios, we added an offset of 100 to the adjusted values. For the second part of pre-processing, i.e., normalization, we used ‘normalizebetweenarrays’ method with the ‘quantile normalization’ function in limma. After pre-processing of data, we compared healthy controls with meningioma samples with the aim of identifying differentially expressed proteins.

‘limma’ utilizes moderated t-statistic for testing the null hypothesis, which is that the proteins are not differentially expressed. The adjustments for multiple hypotheses testing were done using “Benjamin-Hochberg” (BH) correction method. The proteins which had fold change more than 0.5 or less than -0.5 on log2 scale and adjusted p-values less than 0.05 were considered to be potentially differentially expressed. Such proteins were later analysed to find out the enrichment of pathways which may contribute to tumorigenesis.

### Correlation of autoantibody response with the tissue and serum proteome

Significant proteins dysregulated in our autoantibody study (Supplementary Table 1, 2 and 3) was cross compared to previously published meningioma tissue and serum antigenic protein profiles obtained from high throughput mass-spectromentric platforms. (Supplementary Table 4) [25, 26].

### Comparison with gene expression profiling study

A list of significant proteins obtained from our study (Table 1) were compared with a gene expression profiling study conducted by Tabernero et al. [27]. This dataset was generated by retrieved from GEO (GSE43290). This study was performed using 4 controls, 33 MG1, 12 MG2 and 2 MG3 samples. However, we did not include Grade III samples for comparing with our study. The extracted data was subjected to quantile normalization and was used for comparison.

### Validation using immunoblotting

Immunoblotting was done using whole tissue lysates from the meningioma samples. Tissue lysates were generated as per the standard protocol for immunoblotting. In brief, 50mg of tissue was washed with 1X PBS buffer followed by sonication is 40% amplitude in RIPA buffer for 2-5mins using 5s pulse on and off respectively. The debris was discarded post centrifugation and the lysates quantified using Bradford Assay. The lysates were run on 12% SDS-PAGE and transferred on PVDF membrane in a semi-dry blotting unit (Biorad). The Rb-pAb anti-Selenium Binding Protein antibody (Abcam, catalogue number: ab72249) was used in dilution of 1:3000. The blots were blocked with 5% Skimmed milk overnight prior to probing with antibody. Primary antibody incubation was done for 1 hour and 30 minutes, post subsequent washes incubation with the secondary antibody in dilution of 1:6000 was done (GeNei, Goat anti-rabbit IgG-HRP conjugate, catalogue number 62114038001A) incubation was done for 1 hour and 30 minutes. A similar protocol was followed for TPD52L2, the primary dilutions being 1:1000 (Abcam, catalogue number: ab77337) and secondary antibody used in the dilution of 1:3000 (GeNei, Rabbit anti-goat-IgG-HRP conjugate, catalogue number 62114048001). Post washes with 1X TBST the blots were developed using TMB (Supplementary Table 4, Supplementary Figure 4).

### Pathway analysis

The list of significant proteins obtained after various statistical and manual curation was subjected to gene set enrichment analysis. The dataset obtained by screening patient sera for autoantibody detection was analysed by FunRich: Functional Enrichment Analysis Tool®. The genes involved in the statistically significant pathways emerging from these studies were then traced back to the trends they show in our data. The pathways which had p-value less than 0.05 using “Benjamin-Hochberg” (BH) correction method were considered to be significant. Molecular interaction clusters were generated using this shorter list of proteins in StringDB [29] which were further analysed for its relevance to meningioma manifestation. MCL clustering in String DB was used with an index of 2 for MG1 and 5 for MG2 for deciphering network interactions. GO terms from STRING DB v10.0 were exported to REVIGO [30] to generate a scatter plot to remove redundant GO terms and cluster related GO terms together.

## SUPPLEMENTARY MATERIALS FIGURES AND TABLES






























