# Supplementary material for: Evaluation of autoantibody signatures in meningioma patients using human proteome arrays
Source: Oncotarget. 2017 Apr 10;8(35):58443–56. doi: 10.18632/oncotarget.16997 (PMC5601665; doi:10.18632/oncotarget.16997)
Supplement: Supplementary file 1 [file oncotarget-08-58443-s001.pdf]

Evaluation of autoantibody signatures in meningioma patients using human proteome arrays

SUPPLEMENTARY FIGURES AND TABLES

Supplementary Figure 1: Signal intensities of features for all significant proteins across all comparisons.

See Supplementary Figure 1

Supplementary Figure 2: Box plots generated for all significant entities (Log FC≥0.5, adv. p-value ≤0.05).

See Supplementary Figure 2

Supplementary Figure 3: Heat maps generated for all significant entities (Log FC≥0.5, adv. value ≤0.05).

See Supplementary Figure 3

Supplementary Figure 4: Immunoblotting images for SELENBP1 and TPD52L2.

See Supplementary Figure 4

Supplementary Figure 5: MCL clusters generated via STRING DB V 10.0 HC vs MG1_MCL Index 0.

See Supplementary Figure 5

Supplementary Figure 6: Raw scatter plot outputs of GO outputs from STRING DB V 10.0 using REVIGO.

See Supplementary Figure 6

Supplementary Table 1.1: Experimental details. The details of the healthy control and Grade I Meningioma samples

See Supplementary File 1

Supplementary Table 1.2: Shortlisted proteins. List of proteins with p-value<0.05 and logFC >0.5 or <-0.5 (MG1vsHC)

See Supplementary File 1

Supplementary Table 1.3: Shortlisted proteins. List of proteins with p-value<0.05 and logFC >1 or <-1 (MG1vsHC)

See Supplementary File 1

Supplementary Table 2.1: Experimental details. The details of the healthy control and Grade II Meningioma samples.

See Supplementary File 2

Supplementary Table 2.2: Shortlisted proteins abs FC>0.5 (MGII vs HC)

See Supplementary File 2

Supplementary Table 2.3: Shortlisted proteins abs FC>1 (MGII vs HC)

See Supplementary File 2

Supplementary Table 3.1: Experimental details. The details of the healthy control and Meningioma samples.

See Supplementary File 3

Supplementary Table 3.2: Shortlisted proteins. List of proteins with p-value<0.05 and log FC >0.5or <-0.5 (MG vs HC)

See Supplementary File 3

Supplementary Table 3.3: Shortlisted proteins. List of proteins with p-value<0.05 and log FC >0.5 or <-0.5 (MG vs HC)

See Supplementary File 3

Supplementary Table 3.4: Full extended tables for main Table 1 and Table 2

See Supplementary File 3

Supplementary Table 3.5: Comparison with serum proteomics study

See Supplementary File 4

Supplementary Table 4.1: IQTL analysis for SELENBP1

See Supplementary File 4

Supplementary Table 4.2: IQTL data for TPD52

See Supplementary File 4

Supplementary Table 4.3: IQTL analysis for SELENBP1

See Supplementary File 4

Supplementary Table 4.4: IQTL data for TPD52

See Supplementary File 4

Supplementary Table 5: Gene set enrichment analysis of MGI vs HC

See Supplementary File 5

Supplementary Table 5.1: Gene details

See Supplementary File 5

Supplementary Table 5.2: Cellular component

See Supplementary File 5

Supplementary Table 5.3: Molecular function

See Supplementary File 5

Supplementary Table 5.4: Biological process

See Supplementary File 5

Supplementary Table 5.5: Biological pathway

See Supplementary File 5

Supplementary Table 5.6: Protein domain

See Supplementary File 5

Supplementary Table 5.7: Site of expression

See Supplementary File 5

Supplementary Table 5.8: Transcription factor

See Supplementary File 5

Supplementary Table 5.9: Clinical phenotypes

See Supplementary File 5

Supplementary Table 6: Gene set enrichment analysis of MGII vs HC

See Supplementary File 6

Supplementary Table 6.1: Gene details

See Supplementary File 6

Supplementary Table 6.2: Cellular component

See Supplementary File 6

Supplementary Table 6.3: Molecular function

See Supplementary File 6

Supplementary Table 6.4: Biological process

See Supplementary File 6

Supplementary Table 6.5: Biological pathway

See Supplementary File 6

Supplementary Table 6.6: Protein domain

See Supplementary File 6

Supplementary Table 6.7: Site of expression

See Supplementary File 6

Supplementary Table 6.8: Transcription factor

See Supplementary File 6

Supplementary Table 6.9: Clinical phenotypes

See Supplementary File 6

Supplementary Table 7: Gene set enrichment analysis of MG vs HC

See Supplementary File 7

Supplementary Table 7.1: Gene details

See Supplementary File 7

Supplementary Table 7.2: Cellular component

See Supplementary File 7

Supplementary Table 7.3: Molecular function

See Supplementary File 7

Supplementary Table 7.4: Biological process

See Supplementary File 7

Supplementary Table 7.5: Biological pathway

See Supplementary File 7

Supplementary Table 7.6: Protein domain

See Supplementary File 7

Supplementary Table 7.7: Site of expression

See Supplementary File 7

Supplementary Table 7.8: Transcription factor

See Supplementary File 7

Supplementary Table 7.9: Clinical phenotypes

See Supplementary File 7

Supplementary Table 8: GO clustering using Revigo

See Supplementary File 8

Supplementary Table 8.1: Input list of proteins for generating GO terms (significant proteins in MG1 vs HC with absolute logFC>0.5, p-value<0.05)

See Supplementary File 8

Supplementary Table 8.2: GO output for HC vs MG1 from StringDB

See Supplementary File 8

Supplementary Table 8.3: GO scatter-plot from REVIGO for HCvsMG1

See Supplementary File 8

Supplementary Table 8.4: Input list of proteins for generating GO terms (significant proteins in MG2 vs HC with absolute logFC>0.5, p-value<0.05)

See Supplementary File 8

Supplementary Table 8.5: GO output for HC vs MG2 from StringDB

See Supplementary File 8

Supplementary Table 8.6: GO output for HC vs MG1 from StringDB

See Supplementary File 8
