# Supplementary material for: Evaluation of autoantibody signatures in meningioma patients using human proteome arrays
Source: Oncotarget. 2017 Apr 10;8(35):58443–56. doi: 10.18632/oncotarget.16997 (PMC5601665; doi:10.18632/oncotarget.16997)
Supplement: Supplementary file 2 [file oncotarget-08-58443-s002.docx]

**Supplementary Figure 1:** Signal intensities of features for all significant proteins across all comparisons.

# BC025985.1 IGHG4


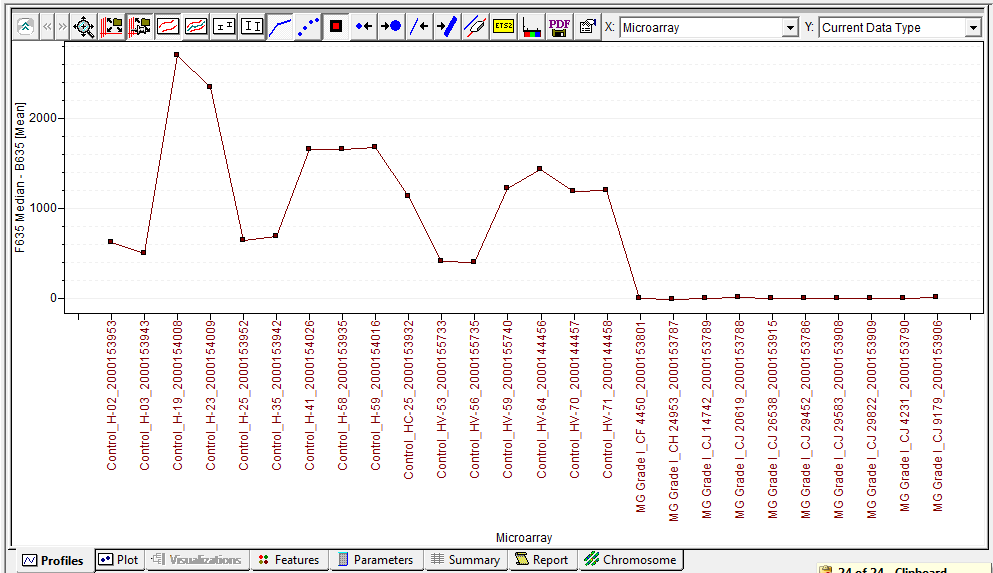


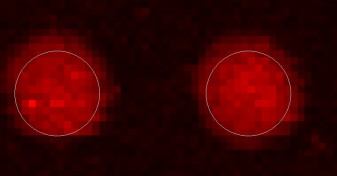

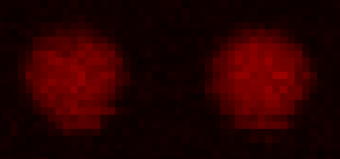

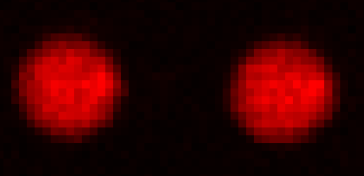

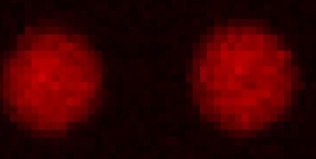


Control_H-02_2000153953 Control_H-03_2000153943 Control_H-19_2000154008 Control_H-25_2000153952


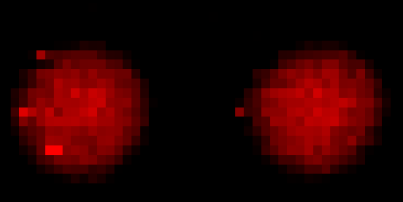

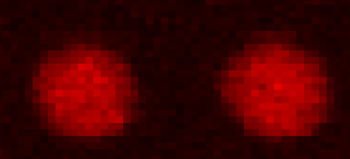

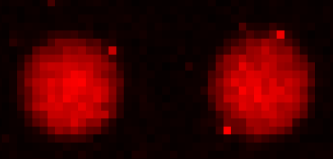

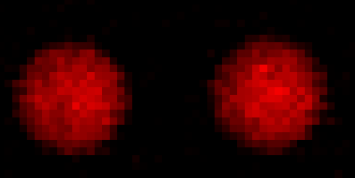


Control_H-23_2000154009

Control_H-35_2000153942

Control_H-58_2000153935

Control_H-41_2000154026


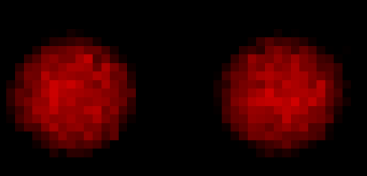

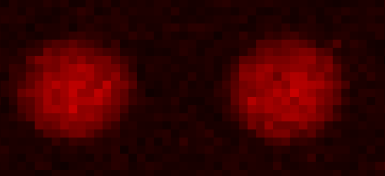

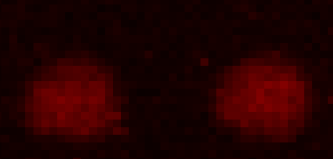

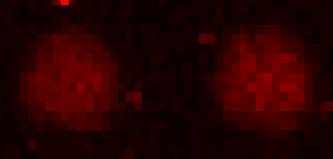


Control_H-59_2000154016 Control_HC 25_2000153932 Control_HV-53_2000155733 Control_HV-56_2000155735


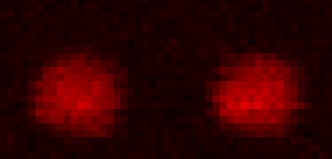

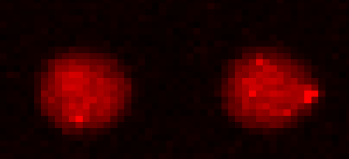

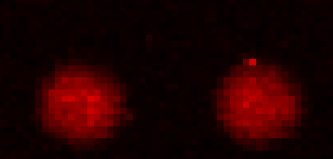

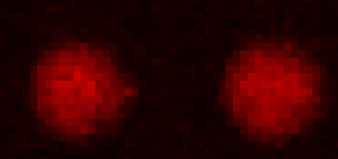


Control_HV-59_2000155740 Control_HV-64_2000144456 Control_HV-70_2000144457 Control_HV-71_2000144458

###### CONTROL


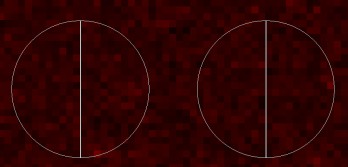

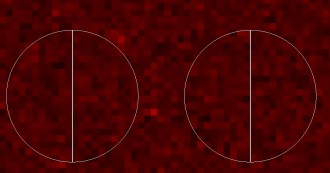

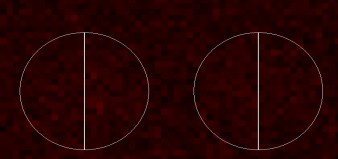


MG_Grade_I_CF 4450_2000153801 MG_Grade_I_CH 24953_2000153787 MG_Grade_I_CJ 4231_2000153790


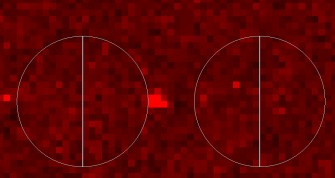

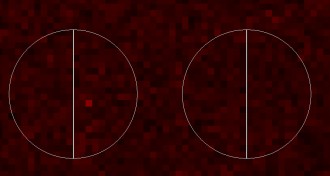

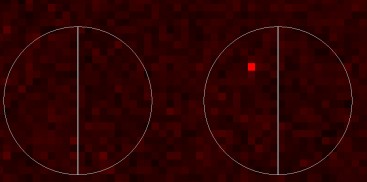


MG_Grade_I_CJ 9179_2000153906 MG_Grade_I_CJ 14742_2000153789 MG_Grade_I_CJ 20619_2000153788


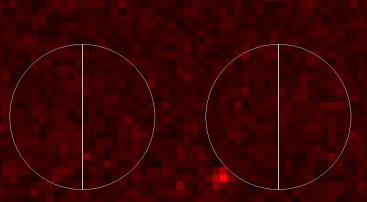

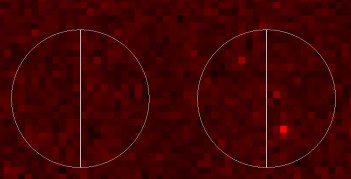

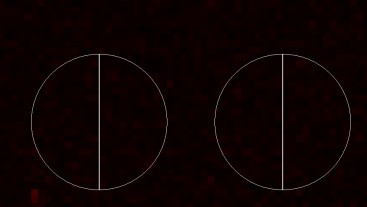


MG_Grade_I_CJ 26538_2000153915 MG_Grade_I_CJ 29452_2000153786 MG_Grade_I_CJ 29583_2000153908


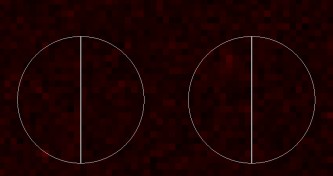


MG_Grade_I_CJ 29822_2000153909

###### GRADE I


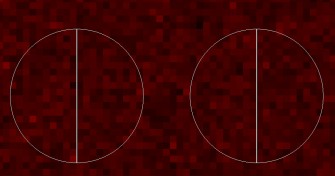

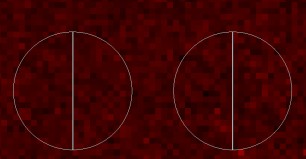

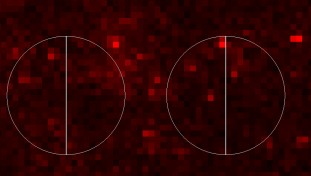


MG_Grade_II_CH 17967_2000153914 MG_Grade_II_CJ 3577_2000153910 MG_Grade_II_CJ 15491_2000153803


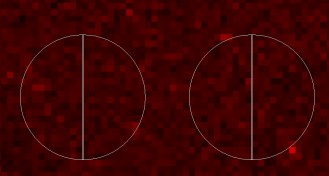

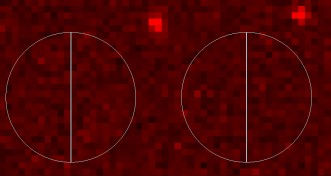


MG_Grade_II_CJ 15753_2000153802 MG_Grade_II_CK 7710_2000153907

###### GRADE II

NM_001014444.1 CRYM


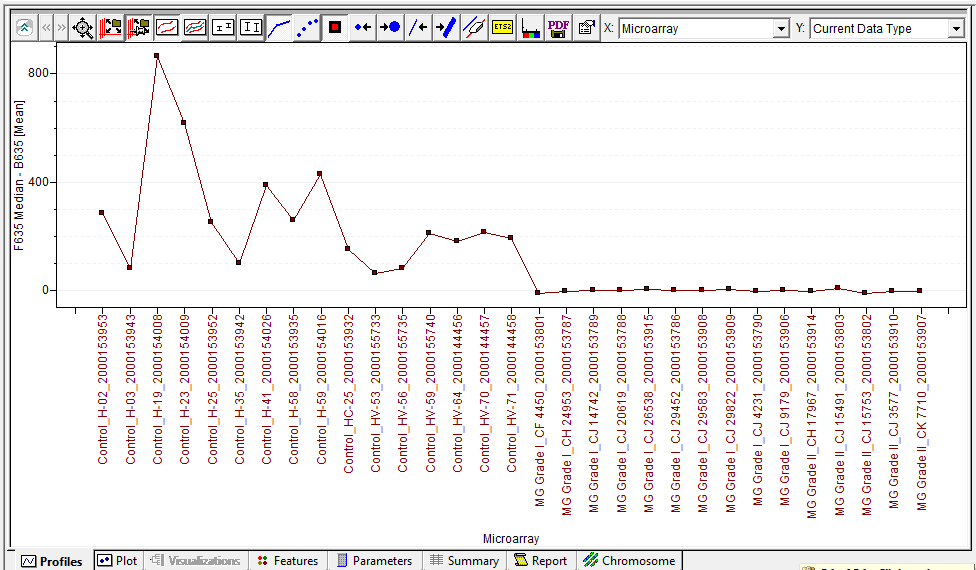


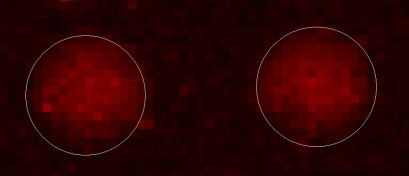

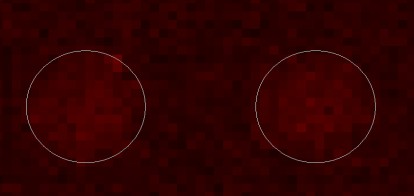

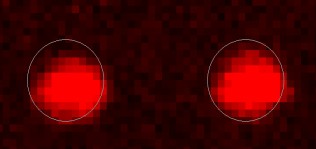

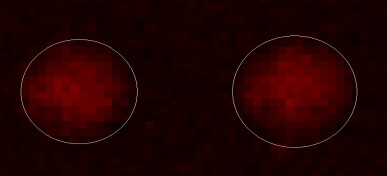


Control_H-02_2000153953 Control_H-03_2000153943 Control_H-19_2000154008 Control_H-25_2000153952


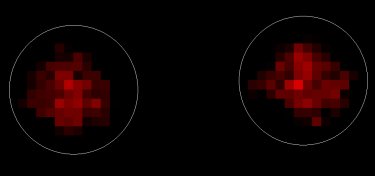

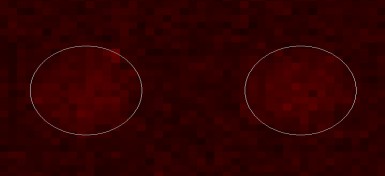

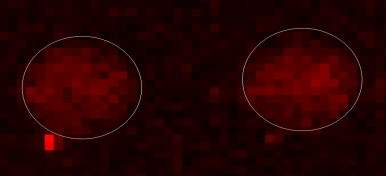

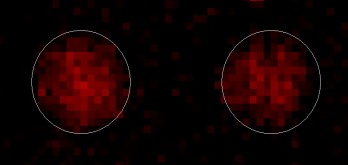


Control_H-23_2000154009

Control_H-35_2000153942

Control_H-58_2000153935

Control_H-41_2000154026


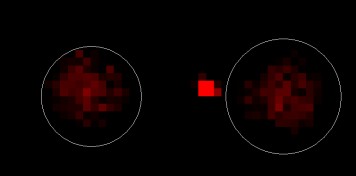

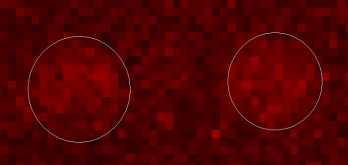

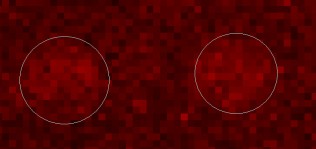

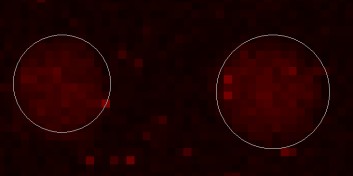


Control_H-59_2000154016 Control_HC 25_2000153932

Control_HV-71_2000144458

Control_HV-56_2000155735


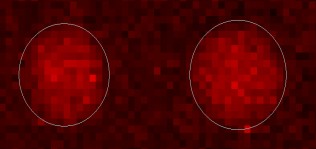

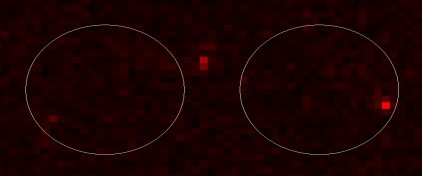

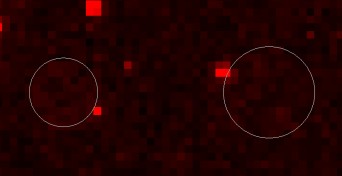


Control_HV-59_2000155740 Control_HV-64_2000144456 Control_HV-70_2000144457(REPEAT)

###### CONTROL


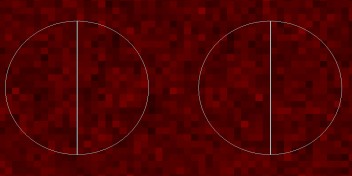

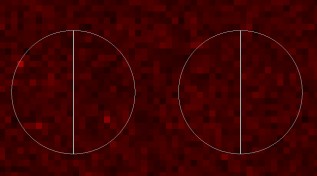

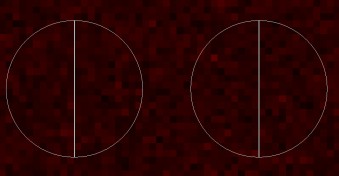


MG_Grade_I_CF 4450_2000153801 MG_Grade_I_CH 24953_2000153787 MG_Grade_I_CJ 4231_2000153790


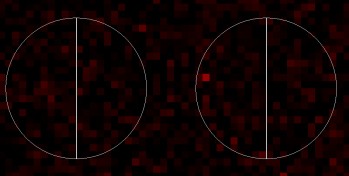

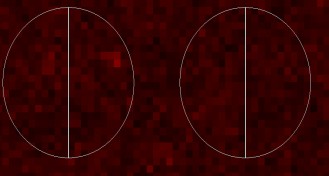

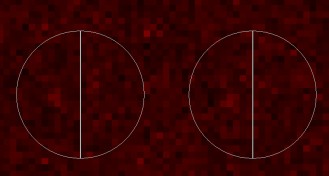


MG_Grade_I_CJ 9179_2000153906 MG_Grade_I_CJ 14742_2000153789 MG_Grade_I_CJ 20619_2000153788


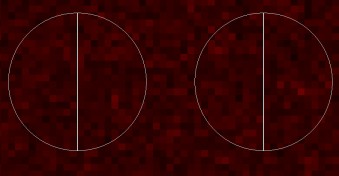

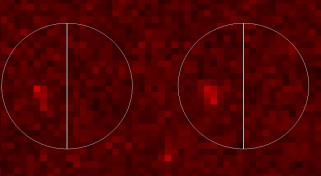

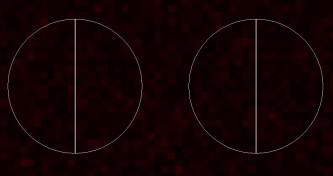


MG_Grade_I_CJ 26538_2000153915 MG_Grade_I_CJ 29452_2000153786 MG_Grade_I_CJ 29583_2000153908


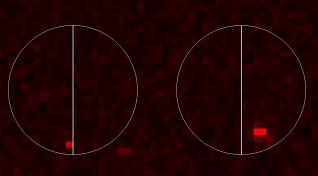


MG_Grade_I_CJ 29822_2000153909

###### GRADE I


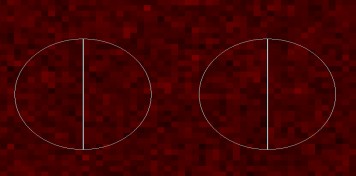

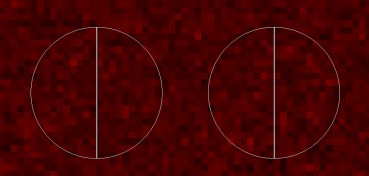

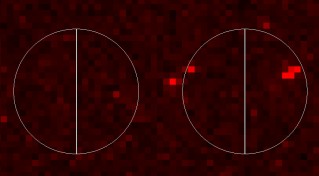


MG_Grade_II_CH 17967_2000153914 MG_Grade_II_CJ 3577_2000153910 MG_Grade_II_CJ 15491_2000153803


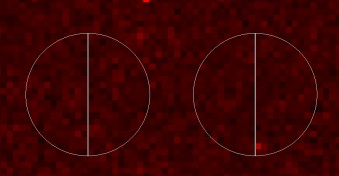

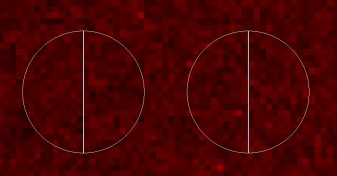


MG_Grade_II_CJ 15753_2000153802 MG_Grade_II_CK 7710_2000153907

###### GRADE II

NM_032328.1 EFCAB2


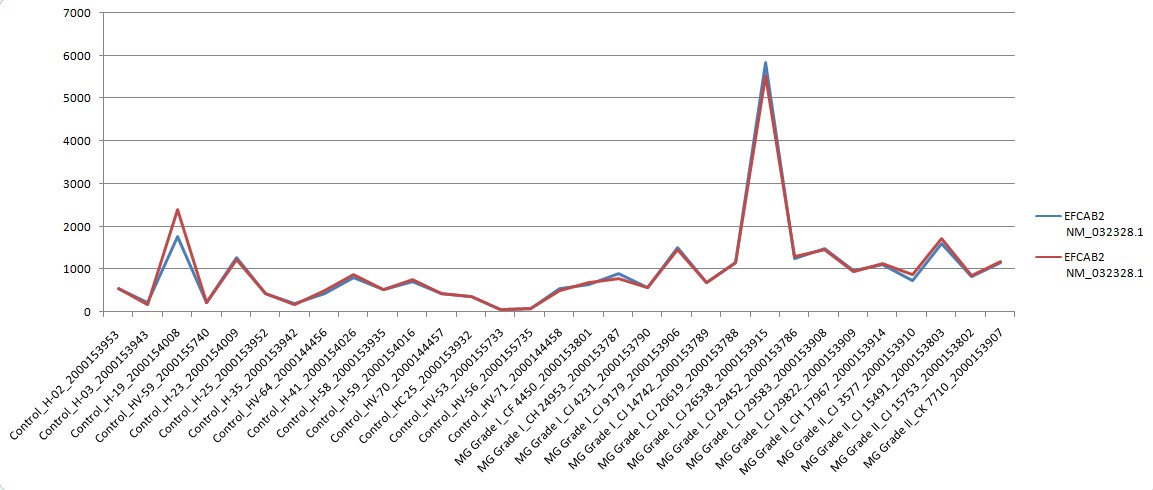


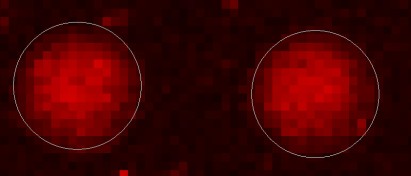

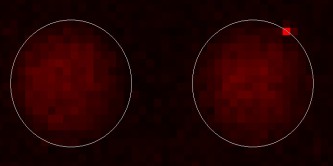

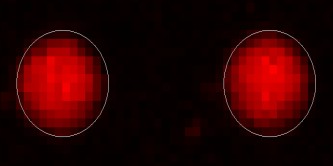

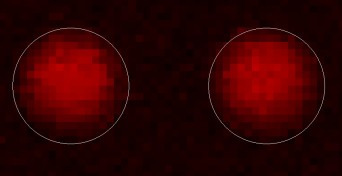


Control_H-02_2000153953 Control_H-03_2000153943 Control_H-19_2000154008 Control_H-25_2000153952


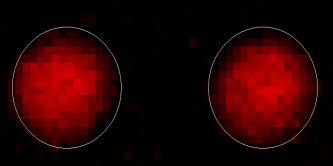

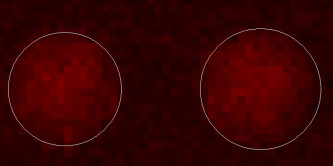

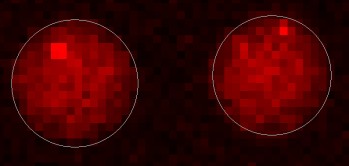

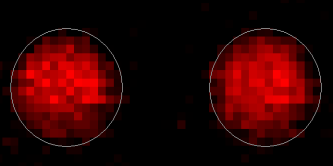


Control_H-23_2000154009

Control_H-35_2000153942

Control_H-58_2000153935

Control_H-41_2000154026


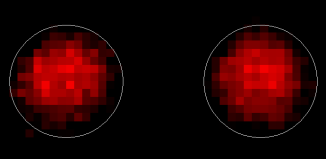

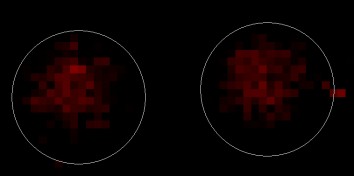

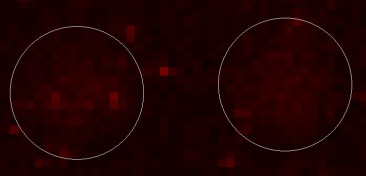

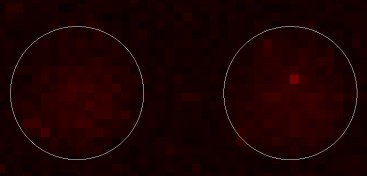


Control_H-59_2000154016 Control_HC 25_2000153932 Control_HV-53_2000155733 Control_HV-56_2000155735


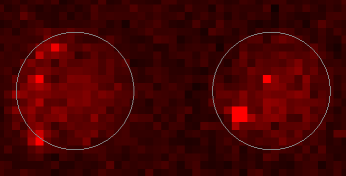

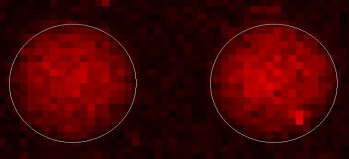

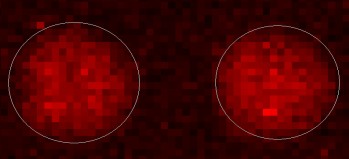

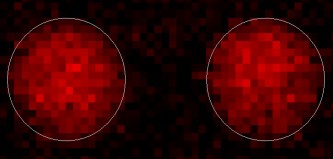


Control_HV-59_2000155740 Control_HV-64_2000144456 Control_HV-70_2000144457 Control_HV-71_2000144458

###### CONTROL


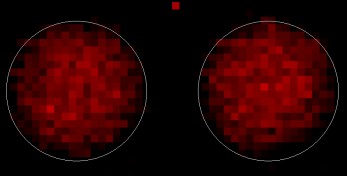

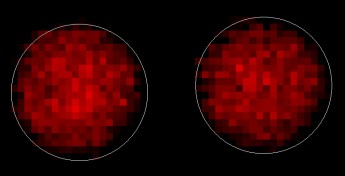

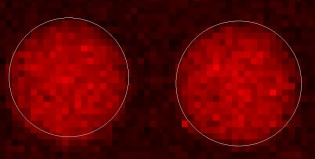


MG_Grade_I_CF 4450_2000153801 MG_Grade_I_CH 24953_2000153787 MG_Grade_I_CJ 4231_2000153790


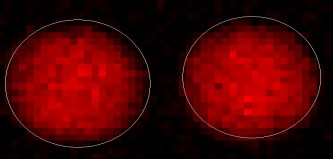

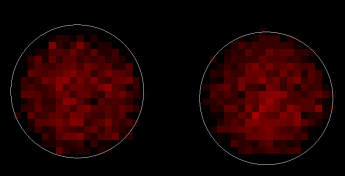

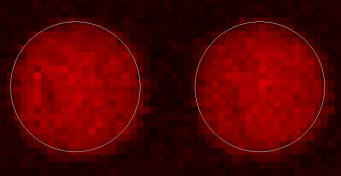


MG_Grade_I_CJ 9179_2000153906 MG_Grade_I_CJ 14742_2000153789 MG_Grade_I_CJ 20619_2000153788


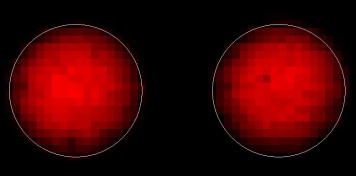

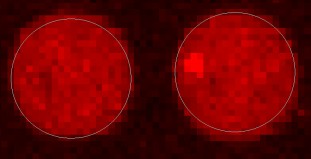

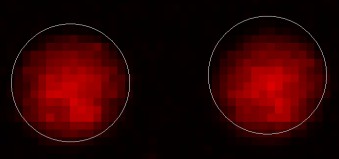


MG_Grade_I_CJ 26538_2000153915 MG_Grade_I_CJ 29452_2000153786 MG_Grade_I_CJ 29583_2000153908


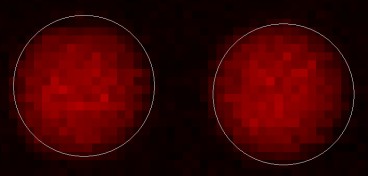


MG_Grade_I_CJ 29822_2000153909

###### GRADE I


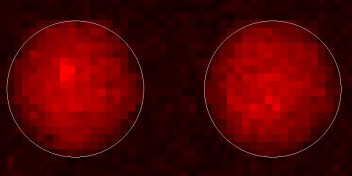

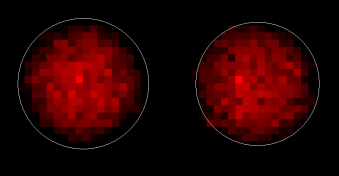

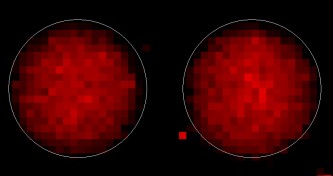


MG_Grade_II_CH 17967_2000153914 MG_Grade_II_CJ 3577_2000153910 MG_Grade_II_CJ 15491_2000153803


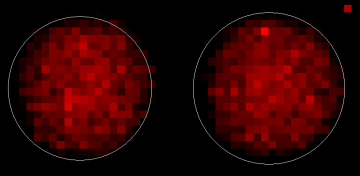

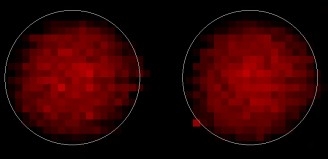


MG_Grade_II_CJ 15753_2000153802 MG_Grade_II_CK 7710_2000153907

###### GRADE II

NM_031304.2 DOHH


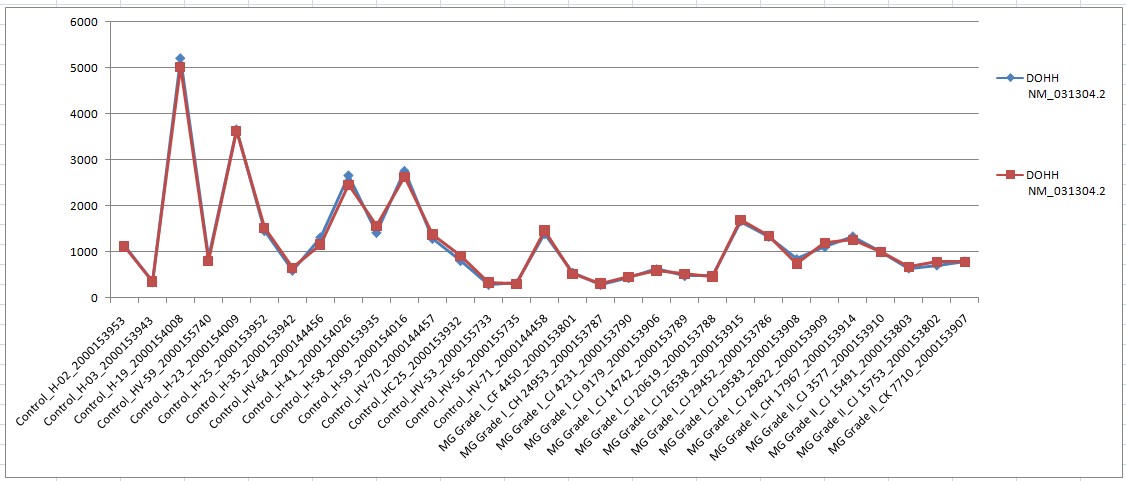


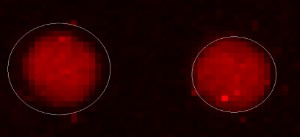

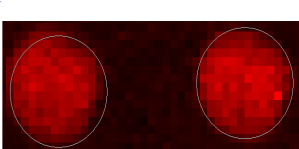

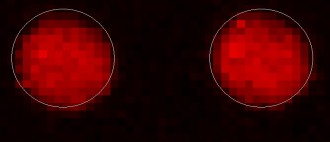

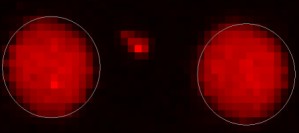


Control_H-02_2000153953 Control_H-03_2000153943 Control_H-19_2000154008 Control_H-25_2000153952

Control_H-23_2000154009

Control_H-35_2000153942

Control_H-58_2000153935

Control_H-41_2000154026

Control_H-59_2000154016 Control_HC 25_2000153932

Control_HV-71_2000144458

Control_HV-56_2000155735

Control_HV-59_2000155740 Control_HV-64_2000144456 Control_HV-70_2000144457

CONTROL

MG_Grade_I_CF 4450_2000153801 MG_Grade_I_CH 24953_2000153787 MG_Grade_I_CJ 4231_2000153790

MG_Grade_I_CJ 9179_2000153906 MG_Grade_I_CJ 14742_2000153789 MG_Grade_I_CJ 20619_2000153788

MG_Grade_I_CJ 26538_2000153915 MG_Grade_I_CJ 29452_2000153786 MG_Grade_I_CJ 29583_2000153908

MG_Grade_I_CJ 29822_2000153909

###### GRADE I

MG_Grade_II_CH 17967_2000153914 MG_Grade_II_CJ 3577_2000153910 MG_Grade_II_CJ 15491_2000153803

MG_Grade_II_CJ 15753_2000153802 MG_Grade_II_CK 7710_2000153907

###### GRADE II

NM_015726.2(WDR42A)

Control_H-02_2000153953 Control_H-03_2000153943 Control_H-19_2000154008 Control_H-25_2000153952

Control_H-23_2000154009

Control_H-35_2000153942

Control_H-58_2000153935

Control_H-41_2000154026

Control_H-59_2000154016 Control_HC 25_2000153932

Control_HV-71_2000144458

Control_HV-56_2000155735

Control_HV-59_2000155740 Control_HV-64_2000144456 Control_HV-70_2000144457

###### CONTROL

MG_Grade_I_CF 4450_2000153801 MG_Grade_I_CH 24953_2000153787 MG_Grade_I_CJ 4231_2000153790

MG_Grade_I_CJ 9179_2000153906 MG_Grade_I_CJ 14742_2000153789 MG_Grade_I_CJ 20619_2000153788

MG_Grade_I_CJ 26538_2000153915 MG_Grade_I_CJ 29452_2000153786 MG_Grade_I_CJ 29583_2000153908

MG_Grade_I_CJ 29822_2000153909

###### GRADE I

MG_Grade_II_CH 17967_2000153914 MG_Grade_II_CJ 3577_2000153910 MG_Grade_II_CJ 15491_2000153803

MG_Grade_II_CJ 15753_2000153802 MG_Grade_II_CK 7710_2000153907

###### GRADE II

BC065370.1 C20orf112

Control_H-02_2000153953 Control_H-03_2000153943 Control_H-19_2000154008 Control_H-25_2000153952

Control_H-23_2000154009

Control_H-35_2000153942

Control_H-58_2000153935

Control_H-41_2000154026

Control_H-59_2000154016 Control_HC 25_2000153932 Control_HV-53_2000155733 Control_HV-56_2000155735

Control_HV-59_2000155740 Control_HV-64_2000144456 Control_HV-70_2000144457 Control_HV-71_2000144458

###### CONTROL

MG_Grade_I_CF 4450_2000153801 MG_Grade_I_CH 24953_2000153787 MG_Grade_I_CJ 4231_2000153790

MG_Grade_I_CJ 9179_2000153906 MG_Grade_I_CJ 14742_2000153789 MG_Grade_I_CJ 20619_2000153788

MG_Grade_I_CJ 26538_2000153915 MG_Grade_I_CJ 29452_2000153786 MG_Grade_I _CJ 29583_2000153908

MG_Grade_I_CJ 29822_2000153909

###### GRADE I

MG_Grade_II_CH 17967_2000153914 MG_Grade_II_CJ 3577_2000153910 MG_Grade_II_CJ 15491_2000153803

MG_Grade_II_CJ 15753_2000153802 MG_Grade_II_CK 7710_2000153907

###### GRADE II

BC037876.1 C17orf57

Control_H-02_2000153953 Control_H-03_2000153943 Control_H-19_2000154008 Control_H-25_2000153952

Control_H-23_2000154009

Control_H-35_2000153942

Control_H-58_2000153935

Control_H-41_2000154026

Control_H-59_2000154016 Control_HC 25_2000153932 Control_HV-53_2000155733 Control_HV-56_2000155735

Control_HV-59_2000155740 Control_HV-64_2000144456 Control_HV-70_2000144457 Control_HV-71_2000144458

###### CONTROL

MG_Grade_I_CF 4450_2000153801 MG_Grade_I_CH 24953_2000153787 MG_Grade_I_CJ 4231_2000153790

MG_Grade_I_CJ 9179_2000153906 MG_Grade_I_CJ 14742_2000153789 MG_Grade_I_CJ 20619_2000153788

MG_Grade_I_CJ 26538_2000153915 MG_Grade_I_CJ 29452_2000153786 MG_Grade_I _CJ 29583_2000153908

MG_Grade_I_CJ 29822_2000153909

###### GRADE I

MG_Grade_II_CH 17967_2000153914 MG_Grade_II_CJ 3577_2000153910 MG_Grade_II_CJ 15491_2000153803

MG Grade II CJ 15753_2000153802 MG Grade II_CK 7710_2000153907

###### GRADE II

LOC389833 (NM_001033515.1)

Control_H-02_2000153953 Control_H-03_2000153943 Control_H-19_2000154008 Control_H-25_2000153952

Control_H-23_2000154009

Control_H-35_2000153942

Control_H-58_2000153935

Control_H-41_2000154026

Control_H-59_2000154016 Control_HC 25_2000153932

Control_HV-71_2000144458

Control_HV-56_2000155735

Control_HV-59_2000155740 Control_HV-64_2000144456 Control_HV-70_2000144457

###### CONTROL

MG_Grade_I_CF 4450_2000153801 MG_Grade_I_CH 24953_2000153787 MG_Grade_I_CJ 4231_2000153790

MG_Grade_I_CJ 9179_2000153906 MG_Grade_I_CJ 14742_2000153789 MG_Grade_I_CJ 20619_2000153788

MG_Grade_I_CJ 26538_2000153915 MG_Grade_I_CJ 29452_2000153786 MG_Grade_I_CJ 29583_2000153908

MG_Grade_I_CJ 29822_2000153909

###### GRADE I

MG_Grade_II_CH 17967_2000153914 MG_Grade_II_CJ 3577_2000153910 MG_Grade_II_CJ 15491_2000153803

MG_Grade_II_CJ 15753_2000153802 MG_Grade_II_CK 7710_2000153907

###### GRADE II

OR10G3 ( NM_001005465.1)

Control_H-02_2000153953 Control_H-03_2000153943 Control_H-19_2000154008

Control_H-25_2000153952

Control_H-23_2000154009

Control_H-35_2000153942

Control_H-58_2000153935

Control_H-41_2000154026

Control_H-59_2000154016 Control_HC 25_2000153932

Control_HV-71_2000144458

Control_HV-56_2000155735

Control_HV-59_2000155740 Control_HV-64_2000144456 Control_HV-70_2000144457

###### CONTROL

MG_Grade_I_CF 4450_2000153801 MG_Grade_I_CH 24953_2000153787 MG_Grade_I_CJ 4231_2000153790

MG_Grade_I_CJ 9179_2000153906 MG_Grade_I_CJ 14742_2000153789 MG_Grade_I_CJ 20619_2000153788

MG_Grade_I_CJ 26538_2000153915 MG_Grade_I_CJ 29452_2000153786 MG_Grade_I_CJ 29583_2000153908

MG_Grade_I_CJ 29822_2000153909

###### GRADE I

MG_Grade_II_CH 17967_2000153914 MG_Grade_II_CJ 3577_2000153910 MG_Grade_II_CJ 15491_2000153803

MG_Grade_II_CJ 15753_2000153802 MG_Grade_II_CK 7710_2000153907

###### GRADE II

NM_139204.1(EPS8L1)

Control_H-02_2000153953 Control_H-03_2000153943 Control_H-19_2000154008

Control_H-25_2000153952

Control_H-23_2000154009

Control_H-35_2000153942

Control_H-58_2000153935

Control_H-41_2000154026

Control_H-59_2000154016 Control_HC 25_2000153932

Control_HV-71_2000144458

Control_HV-56_2000155735

Control_HV-59_2000155740 Control_HV-64_2000144456 Control_HV-70_2000144457

###### CONTROL

MG_Grade_I_CF 4450_2000153801 MG_Grade_I_CH 24953_2000153787 MG_Grade_I_CJ 4231_2000153790

MG_Grade_I_CJ 9179_2000153906 MG_Grade_I_CJ 14742_2000153789 MG_Grade_I_CJ 20619_2000153788

MG_Grade_I_CJ 26538_2000153915 MG_Grade_I_CJ 29452_2000153786 MG_Grade_I_CJ 29583_2000153908

MG_Grade_I_CJ 29822_2000153909

###### GRADE I

MG_Grade_II_CH 17967_2000153914 MG_Grade_II_CJ 3577_2000153910 MG_Grade_II_CJ 15491_2000153803

MG_Grade_II_CJ 15753_2000153802 MG_Grade_II_CK 7710_2000153907

###### GRADE II

NM_001025266.1 (LOC285382)

Control_H-02_2000153953 Control_H-03_2000153943 Control_H-19_2000154008 Control_H-25_2000153952

Control_H-23_2000154009

Control_H-35_2000153942

Control_H-58_2000153935

Control_H-41_2000154026

Control_H-59_2000154016 Control_HC 25_2000153932

Control_HV-71_2000144458

Control_HV-56_2000155735

Control_HV-59_2000155740 Control_HV-64_2000144456 Control_HV-70_2000144457

###### CONTROL

MG_Grade_I_CF 4450_2000153801 MG_Grade_I_CH 24953_2000153787 MG_Grade_I_CJ 4231_2000153790

MG_Grade_I_CJ 9179_2000153906 MG_Grade_I_CJ 14742_2000153789 MG_Grade_I_CJ 20619_2000153788

MG_Grade_I_CJ 26538_2000153915 MG_Grade_I_CJ 29452_2000153786 MG_Grade_I _CJ 29583_2000153908

MG_Grade_I_CJ 29822_2000153909

###### GRADE I

MG_Grade_II_CH 17967_2000153914 MG_Grade_II_CJ 3577_2000153910 MG_Grade_II_CJ 15491_2000153803

MG_Grade_II_CJ 15753_2000153802 MG_Grade_II_CK 7710_2000153907

###### GRADE II

NM_148910.2 TIRAP

Control_H-02_2000153953 Control_H-03_2000153943 Control_H-19_2000154008 Control_H-25_2000153952

Control_H-23_2000154009

Control_H-35_2000153942

Control_H-58_2000153935

Control_H-41_2000154026

Control_H-59_2000154016 Control_HC 25_2000153932 Control_HV-53_2000155733 Control_HV-56_2000155735

Control_HV-59_2000155740 Control_HV-64_2000144456 Control_HV-70_2000144457 Control_HV-71_2000144458

###### CONTROL

MG_Grade_I_CF 4450_2000153801 MG_Grade_I_CH 24953_2000153787 MG_Grade_I_CJ 4231_2000153790

MG_Grade_I_CJ 9179_2000153906 MG_Grade_I_CJ 14742_2000153789 MG_Grade_I_CJ 20619_2000153788

MG_Grade_I_CJ 26538_2000153915 MG_Grade_I_CJ 29452_2000153786 MG_Grade_I_CJ 29583_2000153908

MG_Grade_I_CJ 29822_2000153909

###### GRADE I

MG_Grade_II_CH 17967_2000153914 MG_Grade_II_CJ 3577_2000153910 MG_Grade_II_CJ 15491_2000153803

MG_Grade_II_CJ 15753_2000153802 MG_Grade_II_CK 7710_2000153907

###### GRADE II

NM_002893.2 (RBBP7) Human

retinoblastoma binding protein 7

Control_H-02_2000153953 Control_H-03_2000153943 Control_H-19_2000154008 Control_H-25_2000153952

Control_H-23_2000154009

Control_H-35_2000153942

Control_H-58_2000153935

Control_H-41_2000154026

Control_H-59_2000154016 Control_HC 25_2000153932

Control_HV-71_2000144458

Control_HV-56_2000155735

Control_HV-59_2000155740 Control_HV-64_2000144456 Control_HV-70_2000144457

###### CONTROL

MG_Grade_I_CF 4450_2000153801 MG_Grade_I_CH 24953_2000153787 MG_Grade_I_CJ 4231_2000153790

MG_Grade_I_CJ 9179_2000153906 MG_Grade_I_CJ 14742_2000153789 MG_Grade_I_CJ 20619_2000153788

MG_Grade_I_CJ 26538_2000153915 MG_Grade_I_CJ 29452_2000153786 MG_Grade_I_CJ 29583_2000153908

MG_Grade_I_CJ 29822_2000153909

###### GRADE I

MG_Grade_II_CH 17967_2000153914 MG_Grade_II CJ 3577_2000153910 MG_Grade_II_CJ 15491_2000153803

MG_Grade_II_CJ 15753_2000153802 MG_Grade_II_CK 7710_2000153907

###### GRADE II

NM_021810.3(CDH26 )

Control_H-02_2000153953 Control_H-03_2000153943 Control_H-19_2000154008 Control_H-25_2000153952

Control_H-23_2000154009

Control_H-35_2000153942

Control_H-58_2000153935

Control_H-41_2000154026

Control_H-59_2000154016 Control_HC 25_2000153932

Control_HV-71_2000144458

Control_HV-56_2000155735

Control_HV-59_2000155740 Control_HV-64_2000144456 Control_HV-70_2000144457

###### CONTROL

MG_Grade_I_CF 4450_2000153801 MG_Grade_I_CH 24953_2000153787 MG_Grade_I_CJ 4231_2000153790

MG_Grade_I_CJ 9179_2000153906 MG_Grade_I_CJ 14742_2000153789 MG_Grade_I_CJ 20619_2000153788

MG_Grade_I_CJ 26538_2000153915 MG_Grade_I_CJ 29452_2000153786 MG_Grade_I_CJ 29583_2000153908

MG_Grade_I_CJ 29822_2000153909

###### GRADE I

MG_Grade_II_CH 17967_2000153914 MG_Grade_II CJ 3577_2000153910 MG_Grade_II_CJ 15491_2000153803

MG_Grade_II_CJ 15753_2000153802 MG_Grade_II_CK 7710_2000153907

###### GRADE II

XM_290842.4 (LRFN1)

Control_H-02_2000153953 Control_H-03_2000153943 Control_H-19_2000154008

Control_H-25_2000153952

Control_H-23_2000154009

Control_H-35_2000153942

Control_H-58_2000153935

Control_H-41_2000154026

Control_H-59_2000154016 Control_HC 25_2000153932 Control_HV-53_2000155733 Control_HV-56_2000155735

Control_HV-59_2000155740 Control_HV-64_2000144456 Control_HV-70_2000144457 Control_HV-71_2000144458

###### CONTROL

MG_Grade_I_CF 4450_2000153801 MG_Grade_I_CH 24953_2000153787 MG_Grade_I_CJ 4231_2000153790

MG_Grade_I_CJ 9179_2000153906 MG_Grade_I_CJ 14742_2000153789 MG_Grade_I_CJ 20619_2000153788

MG_Grade_I_CJ 26538_2000153915 MG_Grade_I_CJ 29452_2000153786 MG_Grade_I _CJ 29583_2000153908

MG_Grade_I_CJ 29822_2000153909

###### GRADE I

MG_Grade_II_CH 17967_2000153914 MG_Grade_II_CJ 3577_2000153910 MG_Grade_II_CJ 15491_2000153803

MG_Grade_II_CJ 15753_2000153802 MG_Grade_II_CK 7710_2000153907

###### GRADE II

NM_018584.4(CAMK2N1)

Control_H-02_2000153953 Control_H-03_2000153943 Control_H-19_2000154008 Control_H-25_2000153952

Control_H-23_2000154009

Control_H-35_2000153942

Control_H-58_2000153935

Control_H-41_2000154026

Control_H-59_2000154016 Control_HC 25_2000153932 Control_HV-53_2000155733 Control_HV-56_2000155735

Control_HV-59_2000155740 Control_HV-64_2000144456 Control_HV-70_2000144457 Control_HV-71_2000144458

###### CONTROL

MG_Grade_I_CF 4450_2000153801 MG_Grade_I_CH 24953_2000153787 MG_Grade_I_CJ 4231_2000153790

MG_Grade_I_CJ 9179_2000153906 MG_Grade_I_CJ 14742_2000153789 MG_Grade_I_CJ 20619_2000153788

MG_Grade_I_CJ 26538_2000153915 MG_Grade_I_CJ 29452_2000153786 MG_Grade_I _CJ 29583_2000153908

MG_Grade_I_CJ 29822_2000153909

###### GRADE I

MG_Grade_II_CH 17967_2000153914 MG_Grade_II_CJ 3577_2000153910 MG_Grade_II_CJ 15491_2000153803

MG_Grade_II_CJ 15753_2000153802 MG_Grade_II_CK 7710_2000153907

###### GRADE II

BC090880.1 (EIF3S3)

Control_H-02_2000153953 Control_H-03_2000153943 Control_H-19_2000154008 Control_H-25_2000153952

Control_H-23_2000154009

Control_H-35_2000153942

Control_H-58_2000153935

Control_H-41_2000154026

Control_H-59_2000154016 Control_HC 25_2000153932 Control_HV-53_2000155733 Control_HV-56_2000155735

Control_HV-59_2000155740 Control_HV-64_2000144456 Control_HV-70_2000144457 Control_HV-71_2000144458

CONTROL

MG_Grade_I_CF 4450_2000153801 MG_Grade_I_CH 24953_2000153787 MG_Grade_I_CJ 4231_2000153790

MG_Grade_I_CJ 9179_2000153906 MG_Grade_I_CJ 14742_2000153789 MG_Grade_I_CJ 20619_2000153788

MG_Grade_I_CJ 26538_2000153915 MG_Grade_I_CJ 29452_2000153786 MG_Grade_I _CJ 29583_2000153908

MG_Grade_I_CJ 29822_2000153909

###### GRADE I

MG_Grade_II_CH 17967_2000153914 MG_Grade_II_CJ 3577_2000153910 MG_Grade_II_CJ 15491_2000153803

MG_Grade_II_CJ 15753_2000153802 MG_Grade_II_CK 7710_2000153907

###### GRADE II

NM_198086.1 AJUBA

Control_H-02_2000153953 Control_H-03_2000153943 Control_H-19_2000154008 Control_H-25_2000153952

Control_H-23_2000154009

Control_H-35_2000153942

Control_H-58_2000153935

Control_H-41_2000154026

Control_H-59_2000154016 Control_HC 25_2000153932 Control_HV-53_2000155733 Control_HV-56_2000155735

Control_HV-59_2000155740 Control_HV-64_2000144456 Control_HV-70_2000144457 Control_HV-71_2000144458

###### CONTROL

MG_Grade_I_CF 4450_2000153801 MG_Grade_I_CH 24953_2000153787 MG_Grade_I_CJ 4231_2000153790

MG_Grade_I_CJ 9179_2000153906 MG_Grade_I_CJ 14742_2000153789 MG_Grade_I_CJ 20619_2000153788

MG_Grade_I_CJ 26538_2000153915 MG_Grade_I_CJ 29452_2000153786 MG_Grade_I_CJ 29583_2000153908

MG_Grade_I_CJ 29822_2000153909

###### GRADE I

MG_Grade_II_CH 17967_2000153914 MG_Grade_II_CJ 3577_2000153910 MG_Grade_II_CJ 15491_2000153803

MG_Grade_II_CJ 15753_2000153802 MG_Grade_II_CK 7710_2000153907

###### GRADE II

NM_016224.3 SNX9

Control_H-02_2000153953 Control_H-03_2000153943 Control_H-19_2000154008 Control_H-25_2000153952

Control_H-23_2000154009

Control_H-35_2000153942

Control_H-58_2000153935

Control_H-41_2000154026

Control_H-59_2000154016 Control_HC 25_2000153932

Control_HV-71_2000144458

Control_HV-56_2000155735

Control_HV-59_2000155740 Control_HV-64_2000144456 Control_HV-70_2000144457

###### CONTROL

MG_Grade_I_CF 4450_2000153801 MG_Grade_I_CH 24953_2000153787 MG_Grade_I_CJ 4231_2000153790

MG_Grade_I_CJ 9179_2000153906 MG_Grade_I_CJ 14742_2000153789 MG_Grade_I_CJ 20619_2000153788

MG_Grade_I_CJ 26538_2000153915 MG_Grade_I_CJ 29452_2000153786 MG_Grade_I_CJ 29583_2000153908

MG_Grade_I_CJ 29822_2000153909

###### GRADE I

MG_Grade_II_CH 17967_2000153914 MG_Grade_II_CJ 3577_2000153910 MG_Grade_II_CJ 15491_2000153803

MG_Grade_II_CJ 15753_2000153802 MG_Grade_II_CK 7710_2000153907

###### GRADE II

NM_005719.2 ARPC3

Control_H-02_2000153953 Control_H-03_2000153943 Control_H-19_2000154008 Control_H-25_2000153952

Control_H-23_2000154009

Control_H-35_2000153942

Control_H-58_2000153935

Control_H-41_2000154026

Control_H-59_2000154016 Control_HC 25_2000153932

Control_HV-71_2000144458

Control_HV-56_2000155735

Control_HV-59_2000155740 Control_HV-64_2000144456 Control_HV-70_2000144457

###### CONTROL

MG _Grade_I_CF 4450_2000153801 MG_Grade_I_CH 24953_2000153787 MG_Grade_I_CJ 4231_2000153790

MG_Grade_I_CJ 9179_2000153906 MG_Grade_I_CJ 14742_2000153789 MG_Grade_I_CJ 20619_2000153788

MG_Grade_I_CJ 26538_2000153915 MG_Grade_I_CJ 29452_2000153786 MG_Grade_I_CJ 29583_2000153908

MG_Grade_I_CJ 29822_2000153909

###### GRADE I

MG_Grade_II_CH 17967_2000153914 MG_Grade_II CJ 3577_2000153910 MG_Grade_II_CJ 15491_2000153803

MG-_ Grade_II_CJ 15753_2000153802 MG_Grade_II_CK 7710_2000153907

###### GRADE II

NM_182789 PAIP1

Control_H-02_2000153953 Control_H-03_2000153943 Control_H-19_2000154008 Control_H-25_2000153952

Control_H-23_2000154009

Control_H-35_2000153942

Control_H-58_2000153935

Control_H-41_2000154026

Control_H-59_2000154016 Control_HC 25_2000153932

Control_HV-71_2000144458

Control_HV-56_2000155735

Control_HV-59_2000155470 Control_HV-64_2000144456 Control_HV-70_2000144457

###### CONTROL

MG_Grade_I_CF 4450_2000153801 MG_Grade_I_CH 24953_2000153787 MG_Grade_I_CJ 4231_2000153790

MG_Grade_I_CJ 9179_2000153906 MG_Grade_I_CJ 14742_2000153789 MG_Grade_I_CJ 20619_2000153788

MG_Grade_I_CJ 26538_2000153915 MG_Grade_I_CJ 29452_2000153786 MG_Grade_I_CJ 29583_2000153908

MG_Grade_I_CJ 29822_2000153909

###### GRADE I

MG_Grade_II_CH 17967_2000153914 MG_Grade_II CJ 3577_2000153910 MG_Grade_II_CJ 15491_2000153803

MG_Grade_II_CJ 15753_2000153802 MG_Grade_II_CK 7710_2000153907

###### GRADE II

BC013992.1 MAPK3

Control_H-02_2000153953 Control_H-03_2000153943 Control_H-19_2000154008 Control_H-25_2000153952

Control_H-23_2000154009

Control_H-35_2000153942

Control_H-58_2000153935

Control_H-41_2000154026

Control_H-59_2000154016 Control_HC 25_2000153932

Control_HV-71_2000144458

Control_HV-56_2000155735

Control_HV-59_2000155470 Control_HV-64_2000144456 Control_HV-70_2000144457

###### CONTROL

MG_Grade_I_CF 4450_2000153801 MG_Grade_I_CH 24953_2000153787 MG_Grade_I_CJ 4231_2000153790

MG_Grade_I_CJ 9179_2000153906 MG_Grade_I_CJ 14742_2000153789 MG_Grade_I_CJ 20619_2000153788

MG_Grade_I_CJ 26538_2000153915 MG_Grade_I_CJ 29452_2000153786 MG_Grade_I_CJ 29583_2000153908

MG_Grade_I_CJ 29822_2000153909

###### GRADE I

MG_Grade_II_CH 17967_2000153914 MG_Grade_II CJ 3577_2000153910 MG_Grade_II_CJ 15491_2000153803

MG_Grade_II_CJ 15753_2000153802 MG_Grade_II_CK 7710_2000153907

###### GRADE II

NM_002767.2 PRPSAP2

Control_H-02_2000153953 Control_H-03_2000153943 Control_H-19_2000154008 Control_H-25_2000153952

Control_H-23_2000154009

Control_H-35_2000153942

Control_H-58_2000153935

Control_H-41_2000154026

Control_H-59_2000154016 Control_HC 25_2000153932 Control_HV-53_2000155733 Control_HV-56_2000155735

Control_HV-59_2000155740 Control_HV-64_2000144456 Control_HV-70_2000144457 Control_HV-71_2000144458

###### CONTROL

MG_Grade_I_CF 4450_2000153801 MG_Grade_I_CH 24953_2000153787 MG_Grade_I_CJ 4231_2000153790

MG_Grade_I_CJ 9179_2000153906 MG_Grade_I_CJ 14742_2000153789 MG_Grade_I_CJ 20619_2000153788

MG_Grade_I_CJ 26538_2000153915 MG_Grade_I_CJ 29452_2000153786 MG_Grade_I_CJ 29583_2000153908

MG_Grade_I_CJ 29822_2000153909

###### GRADE I

MG_Grade_II_CH 17967_2000153914 MG_Grade_II_CJ 3577_2000153910 MG_Grade_II_CJ 15491_2000153803

MG_Grade_II_CJ 15753_2000153802 MG_Grade_II_CK 7710_2000153907

###### GRADE II

NM_014372.3 RNF11

RNF11 (NM_014372.3) (down-

regulated)

Control_H-02_2000153953 Control_H-03_2000153943 Control_H-19_2000154008 Control_H-25_2000153952

Control_H-23_2000154009

Control_H-35_2000153942

Control_H-58_2000153935

Control_H-41_2000154026

Control_H-59_2000154016 Control_HC 25_2000153932 Control_HV-53_2000155733 Control_HV-56_2000155735

Control_HV-59_2000155740 Control_HV-64_2000144456 Control_HV-70_2000144457 Control_HV-71_2000144458

###### CONTROL

MG_Grade_I_CF 4450_2000153801 MG_Grade_I_CH 24953_2000153787 MG_Grade_I_CJ 4231_2000153790

MG_Grade_I_CJ 9179_2000153906 MG_Grade_I_CJ 14742_2000153789 MG_Grade_I_CJ 20619_2000153788

MG_Grade_I_CJ 26538_2000153915 MG_Grade_I_CJ 29452_2000153786 MG_Grade_I_CJ 29583_2000153908

MG_Grade_I_CJ 29822_2000153909

###### GRADE I

MG_Grade_II_CH 17967_2000153914 MG_Grade_II_CJ 3577_2000153910 MG_Grade_II_CJ 15491_2000153803

MG_Grade_II_CJ 15753_2000153802 MG_Grade_II_CK 7710_2000153907

###### GRADE II

NM_173809.2 BLOC1S2

Control_H-02_2000153953 Control_H-03_2000153943 Control_H-19_2000154008 Control_H-25_2000153952

Control_H-23_2000154009

Control_H-35_2000153942

Control_H-58_2000153935

Control_H-41_2000154026

Control_H-59_2000154016 Control_HC 25_2000153932

Control_HV-71_2000144458

Control_HV-56_2000155735

Control_HV-59_2000154016 Control_HV-64_2000144456 Control_HV-70_2000144457

###### CONTROL

MG_Grade_I_CF 4450_2000153801 MG_Grade_I_CH 24953_2000153787 MG_Grade_I_CJ 4231_2000153790

MG_Grade_I_CJ 9179_2000153906 MG_Grade_I_CJ 14742_2000153789 MG_Grade_I_CJ 20619_2000153788

MG_Grade_I_CJ 26538_2000153915 MG_Grade_I_CJ 29452_2000153786 MG_Grade_I_CJ 29583_2000153908

MG_Grade_I_CJ 29822_2000153909

###### GRADE I

MG_Grade_II_CH 17967_2000153914 MG_Grade_II CJ 3577_2000153910 MG_Grade_II_CJ 15491_2000153803

MG_Grade_II_CJ 15753_2000153802 MG_Grade_II_CK 7710_2000153907

###### GRADE II

NM_003099.3 SNX1

Control_H-02_2000153953 Control_H-03_2000153943 Control_H-19_2000154008 Control_H-25_2000153952

Control_H-23_2000154009

Control_H-35_2000153942

Control_H-58_2000153935

Control_H-41_2000154026

Control_H-59_2000154016 Control_HC 25_2000153932

Control_HV-71_2000144458

Control_HV-56_2000155735

Control_HV-59_2000154016 Control_HV-64_2000144456 Control_HV-70_2000144457

###### CONTROL

MG_Grade_I_CF 4450_2000153801 MG_Grade_I_CH 24953_2000153787 MG_Grade_I_CJ 4231_2000153790

MG_Grade_I_CJ 9179_2000153906 MG_Grade_I_CJ 14742_2000153789 MG_Grade_I_CJ 20619_2000153788

MG_Grade_I_CJ 26538_2000153915 MG_Grade_I_CJ 29452_2000153786 MG_Grade_I_CJ 29583_2000153908

MG_Grade_I_CJ 29822_2000153909

###### GRADE I

MG_Grade_II_CH 17967_2000153914 MG_Grade_II CJ 3577_2000153910 MG_Grade_II_CJ 15491_2000153803

MG_Grade_II_CJ 15753_2000153802 MG_Grade_II_CK 7710_2000153907

###### GRADE II

Nol3 :NOL3

Control_H-02_2000153953 Control_H-03_2000153943 Control_H-19_2000154008 Control_H-25_2000153952

Control_H-23_2000154009

Control_H-35_2000153942

Control_H-58_2000153935

Control_H-41_2000154026

Control_H-59_2000154016 Control_HC 25_2000153932 Control_HV-53_2000155733 Control_HV-56_2000155735

Control_HV-59_2000155740 Control_HV-64_2000144456 Control_HV-70_2000144457 Control_HV-71_2000144458

CONTROL

MG_Grade_I_CF 4450_2000153801 MG_Grade_I_CH 24953_2000153787 MG_Grade_I_CJ 4231_2000153790

MG_Grade_I_CJ 9179_2000153906 MG_Grade_I_CJ 14742_2000153789 MG_Grade_I_CJ 20619_2000153788

MG_Grade_I_CJ 26538_2000153915 MG_Grade_I_CJ 29452_2000153786

MG_Grade_I_CJ 29822_2000153909

MG_Grade_I _CJ 29583_2000153908

###### GRADE I

MG_Grade_II_CH 17967_2000153914 MG_Grade_II_CJ 3577_2000153910 MG_Grade_II_CJ 15491_2000153803

MG_Grade_II_CJ 15753_2000153802 MG_Grade_II_CK 7710_2000153907

###### GRADE II

NM_001033112.1

PAIP2

Control_H-02_2000153953 Control_H-03_2000153943 Control_H-19_2000154008 Control_H-25_2000153952

Control_H-23_2000154009

Control_H-35_2000153942

Control_H-58_2000153935

Control_H-41_2000154026

Control_H-59_2000154016 Control_HC 25_2000153932

Control_HV-71_2000144458

Control_HV-56_2000155735

Control_HV-59_2000154016 Control_HV-64_2000144456 Control_HV-70_2000144457

CONTROL

MG_Grade_I_CF 4450_2000153801 MG_Grade_I_CH 24953_2000153787 MG_Grade_I_CJ 4231_2000153790

MG_Grade_I_CJ 9179_2000153906 MG_Grade_I_CJ 14742_2000153789 MG_Grade_I_CJ 20619_2000153788

MG_Grade_I_CJ 26538_2000153915 MG_Grade_I_CJ 29452_2000153786 MG_Grade_I_CJ 29583_2000153908

MG_Grade_I_CJ 29822_2000153909

###### GRADE I

MG_Grade_II_CH 17967_2000153914 MG_Grade_II CJ 3577_2000153910 MG_Grade_II_CJ 15491_2000153803

MG_Grade_II_CJ 15753_2000153802 MG_Grade_II_CK 7710_2000153907

###### GRADE II

LHX1

Control_H-02_2000153953 Control_H-03_2000153943 Control_H-19_2000154008 Control_H-25_2000153952

Control_H-23_2000154009

Control_H-35_2000153942

Control_H-58_2000153935

Control_H-41_2000154026

Control_H-59_2000154016 Control_HC 25_2000153932

Control_HV-71_2000144458

Control_HV-56_2000155735

Control_HV-59_2000154016 Control_HV-64_2000144456 Control_HV-70_2000144457

CONTROL

MG_Grade_I_CF 4450_2000153801 MG_Grade_I_CH 24953_2000153787 MG_Grade_I_CJ 4231_2000153790

MG_Grade_I_CJ 9179_2000153906 MG_Grade_I_CJ 14742_2000153789 MG_Grade_I_CJ 20619_2000153788

MG_Grade_I_CJ 26538_2000153915 MG_Grade_I_CJ 29452_2000153786 MG_Grade_I_CJ 29583_2000153908

MG_Grade_I_CJ 29822_2000153909

###### GRADE I

MG_Grade_II_CH 17967_2000153914 MG_Grade_II CJ 3577_2000153910 MG_Grade_II_CJ 15491_2000153803

MG_Grade_II_CJ 15753_2000153802 MG_Grade_II_CK 7710_2000153907

###### GRADE II

**SCREENSHOTS FOR OTHER PROTEINS**

Tumor Protein D52/L2

**NM_003288.2**

Control_H-02_2000153953 Control_H-03_2000153943 Control_H-19_2000154008 Control_H-25_2000153952

Control_H-23_2000154009

Control_H-35_2000153942

Control_H-58_2000153935

Control_H-41_2000154026

Control_H-59_2000154016 Control_HC 25_2000153932

Control_HV-71_2000144458

Control_HV-56_2000155735

Control_HV-59_2000154016 Control_HV-64_2000144456 Control_HV-70_2000144457

CONTROL

MG_Grade_I_CF 4450_2000153801 MG_Grade_I_CH 24953_2000153787 MG_Grade_I_CJ 4231_2000153790

MG_Grade_I_CJ 9179_2000153906 MG_Grade_I_CJ 14742_2000153789 MG_Grade_I_CJ 20619_2000153788

MG_Grade_I_CJ 26538_2000153915 MG_Grade_I_CJ 29452_2000153786 MG_Grade_I_CJ 29583_2000153908

MG_Grade_I_CJ 29822_2000153909

GRADE I

MG_Grade_II_CH 17967_2000153914 MG_Grade_II CJ 3577_2000153910 MG_Grade_II_CJ 15491_2000153803

MG_Grade_II_CJ 15753_2000153802 MG_Grade_II_CK 7710_2000153907

###### GRADE II

BC069020.1 IGHG 1

Control_H-02_2000153953 Control_H-03_2000153943 Control_H-19_2000154008

Control_H-25_2000153952

Control_H-23_2000154009

Control_H-35_2000153942

Control_H-58_2000153935

Control_H-41_2000154026

Control_H-59_2000154016 Control_HC 25_2000153932

Control_HV-71_2000144458

Control_HV-56_2000155735

Control_HV-59_2000154016 Control_HV-64_2000144456 Control_HV-70_2000144457

CONTROL

MG_Grade_I_CF 4450_2000153801 MG_Grade_I_CH 24953_2000153787 MG_Grade_I_CJ 4231_2000153790

MG_Grade_I_CJ 9179_2000153906 MG_Grade_I_CJ 14742_2000153789 MG_Grade_I_CJ 20619_2000153788

MG_Grade_I_CJ 26538_2000153915 MG_Grade_I_CJ 29452_2000153786 MG_Grade_I_CJ 29583_2000153908

MG_Grade_I_CJ 29822_2000153909

###### GRADE I

MG_Grade_II_CH 17967_2000153914 MG_Grade_II CJ 3577_2000153910 MG_Grade_II_CJ 15491_2000153803

MG_Grade_II_CJ 15753_2000153802 MG_Grade_II_CK 7710_2000153907

###### GRADE II

NM_001005465.1

SPAG16

Control_H-02_2000153953 Control_H-03_2000153943 Control_H-19_2000154008 Control_H-25_2000153952

Control_H-23_2000154009

Control_H-35_2000153942

Control_H-58_2000153935

Control_H-41_2000154026

Control_H-59_2000154016 Control_HC 25_2000153932

Control_HV-71_2000144458

Control_HV-56_2000155735

Control_HV-59_20001557 Control_HV-64_2000144456 Control_HV-70_2000144457

CONTROL

MG_Grade_I_CF 4450_2000153801 MG_Grade_I_CH 24953_2000153787 MG_Grade_I_CJ 4231_2000153790

MG_Grade_I_CJ 9179_2000153906 MG_Grade_I_CJ 14742_2000153789 MG_Grade_I_CJ 20619_2000153788

MG_Grade_I_CJ 26538_2000153915 MG_Grade_I_CJ 29452_2000153786 MG_Grade_I_CJ 29583_2000153908

MG_Grade_I_CJ 29822_2000153909

###### GRADE I

MG_Grade_II_CH 17967_2000153914 MG_Grade_II CJ 3577_2000153910 MG_Grade_II_CJ 15491_2000153803

MG_Grade_II_CJ 15753_2000153802 MG_Grade_II_CK 7710_2000153907

###### GRADE II

SELENIUM BINDING PROTEIN (NM_003944.2)

Control_H-02_2000153953 Control_H-03_2000153943 Control_H-19_2000154008 Control_H-25_2000153952

Control_H-23_2000154009

Control_H-35_2000153942

Control_H-58_2000153935

Control_H-41_2000154026

Control_H-59_2000154016 Control_HC 25_2000153932

Control_HV-71_2000144458

Control_HV-56_2000155735

Control_HV-59_2000155740 Control_HV-64_2000144456 Control_HV-70_2000144457

CONTROL

MG_Grade_I_CF 4450_2000153801 MG_Grade_I_CH 24953_2000153787 MG_Grade_I_CJ 4231_2000153790

MG_Grade_I_CJ 9179_2000153906 MG_Grade_I_CJ 14742_2000153789 MG_Grade_I_CJ 20619_2000153788

MG_Grade_I_CJ 26538_2000153915 MG_Grade_I_CJ 29452_2000153786 MG_Grade_I_CJ 29583_2000153908

MG_Grade_I_CJ 29822_2000153909

###### GRADE I

MG_Grade_II_CH 17967_2000153914 MG_Grade_II CJ 3577_2000153910 MG_Grade_II_CJ 15491_2000153803

MG_Grade_II_CJ 15753_2000153802 MG_Grade_II_CK 7710_2000153907

###### GRADE II

**Calcium-activated potassium channel subunit**

**beta-3**

NM_171830.1 KCNMB3

Control_H-02_2000153953 Control_H-03_2000153943 Control_H-19_2000154008 Control_H-25_2000153952

Control_H-23_2000154009

Control_H-35_2000153942

Control_H-58_2000153935

Control_H-41_2000154026

Control_H-59_2000154016 Control_HC 25_2000153932

Control_HV-71_2000144458

Control_HV-56_2000155735

Control_HV-59_2000155740 Control_HV-64_2000144456 Control_HV-70_2000144457

CONTROL

MG_Grade_I_CF 4450_2000153801 MG_Grade_I_CH 24953_2000153787 MG_Grade_I_CJ 4231_2000153790

MG_Grade_I_CJ 9179_2000153906 MG_Grade_I_CJ 14742_2000153789 MG_Grade_I_CJ 20619_2000153788

MG_Grade_I_CJ 26538_2000153915 MG_Grade_I_CJ 29452_2000153786 MG_Grade_I_CJ 29583_2000153908

MG_Grade_I_CJ 29822_2000153909

###### GRADE I

MG_Grade_II_CH 17967_2000153914 MG_Grade_II CJ 3577_2000153910 MG_Grade_II_CJ 15491_2000153803

MG_Grade_II_CJ 15753_2000153802 MG_Grade_II_CK 7710_2000153907

###### GRADE II

NM_006857.1 RY1

Control_H-02_2000153953 Control_H-03_2000153943 Control_H-19_2000154008 Control_H-25_2000153952

Control_H-23_2000154009

Control_H-35_2000153942

Control_H-58_2000153935

Control_H-41_2000154026

Control_H-59_2000154016 Control_HC 25_2000153932

Control_HV-71_2000144458

Control_HV-56_2000155735

Control_HV-59_2000155740 Control_HV-64_2000144456 Control_HV-70_2000144457

CONTROL

MG_Grade_I_CF 4450_2000153801 MG_Grade_I_CH 24953_2000153787 MG_Grade_I_CJ 4231_2000153790

MG_Grade_I_CJ 9179_2000153906 MG_Grade_I_CJ 14742_2000153789 MG_Grade_I_CJ 20619_2000153788

MG_Grade_I_CJ 26538_2000153915 MG_Grade_I_CJ 29452_2000153786 MG_Grade_I_CJ 29583_2000153908

MG_Grade_I_CJ 29822_2000153909

###### GRADE I

MG_Grade_II_CH 17967_2000153914 MG_Grade_II CJ 3577_2000153910 MG_Grade_II_CJ 15491_2000153803

MG_Grade_II_CJ 15753_2000153802 MG_Grade_II_CK 7710_2000153907

###### GRADE II

NM_015004.2 (EXOSC7 )

Control_H-02_2000153953 Control_H-03_2000153943 Control_H-19_2000154008 Control_H-25_2000153952

Control_H-23_2000154009

Control_H-35_2000153942

Control_H-58_2000153935

Control_H-41_2000154026

Control_H-59_2000154016 Control_HC 25_2000153932 Control_HV-53_2000155733 Control_HV-56_2000155735

Control_HV-59_2000155740 Control_HV-64_2000144456 Control_HV-70_2000144457 Control_HV-71_2000144458

CONTROL

MG_Grade_I_CF 4450_2000153801 MG_Grade_I_CH 24953_2000153787 MG_Grade_I_CJ 4231_2000153790

MG_Grade_I_CJ 9179_2000153906 MG_Grade_I_CJ 14742_2000153789 MG_Grade_I_CJ 20619_2000153788

MG_Grade_I_CJ 26538_2000153915 MG_Grade_I_CJ 29452_2000153786 MG_Grade_I _CJ 29583_2000153908

MG_Grade_I_CJ 29822_2000153909

###### GRADE I

MG_Grade_II_CH 17967_2000153914 MG_Grade_II_CJ 3577_2000153910 MG_Grade_II_CJ 15491_2000153803

MG_Grade_II_CJ 15753_2000153802 MG_Grade_II_CK 7710_2000153907

###### GRADE II
