# Supplementary figures and images for: Evaluation of autoantibody signatures in meningioma patients using human proteome arrays
Source: Oncotarget. 2017 Apr 10;8(35):58443–56. doi: 10.18632/oncotarget.16997 (PMC5601665; doi:10.18632/oncotarget.16997)

**Supplementary Figure 2**: Box plots generated for all significant entities (Log FC≥0.5, adv. p-value ≤0.05).


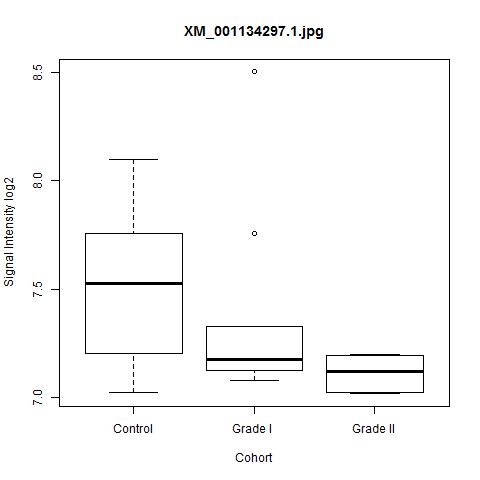

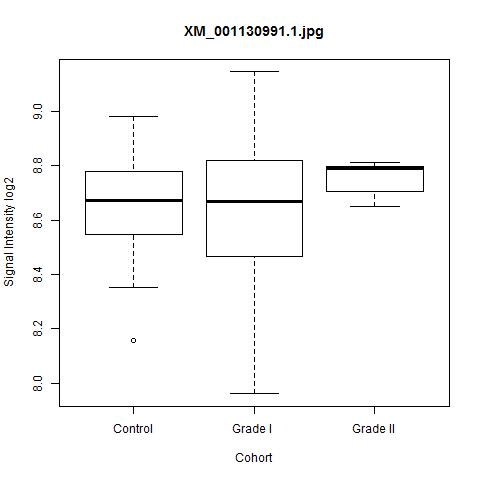

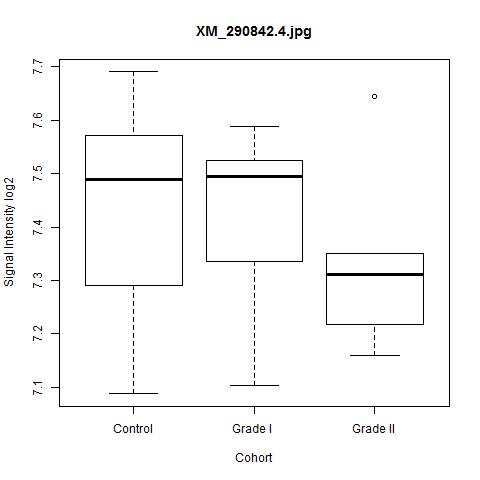

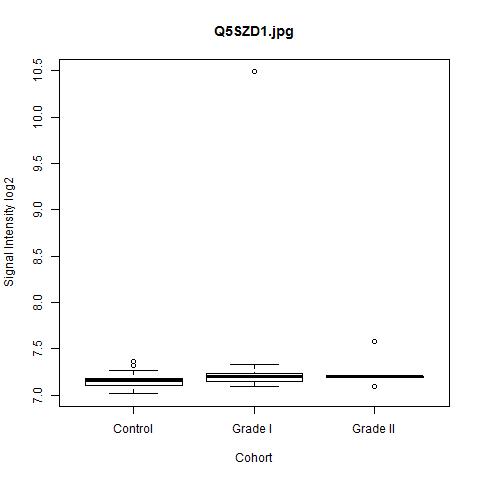

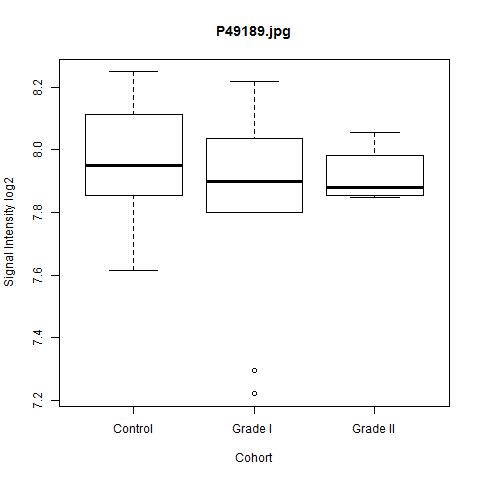

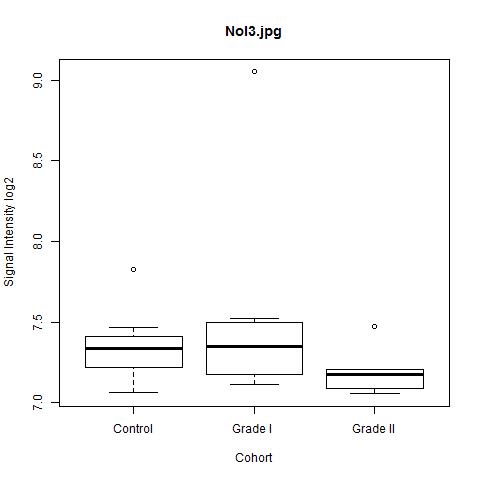

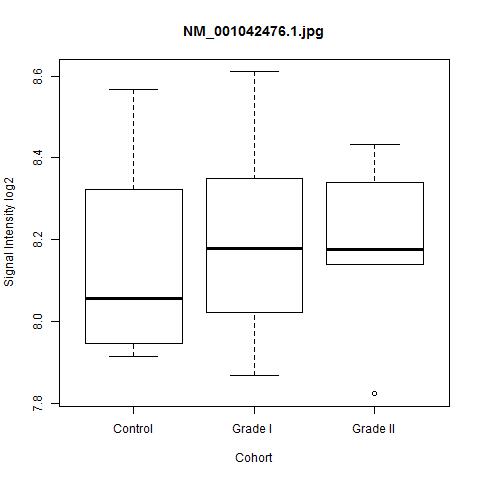

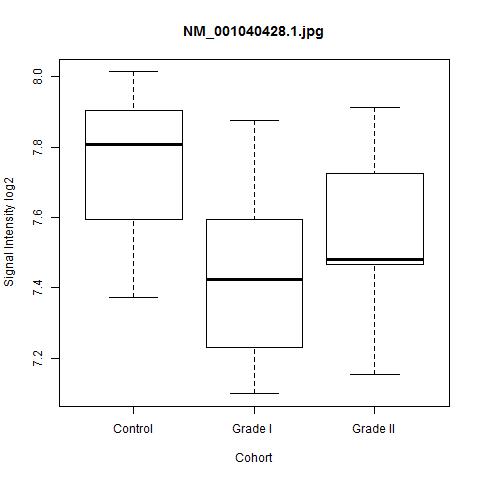

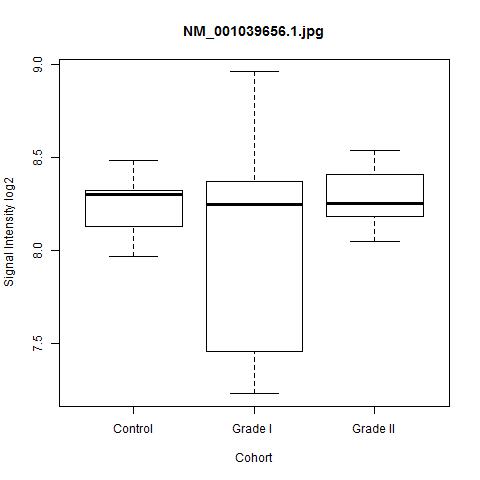

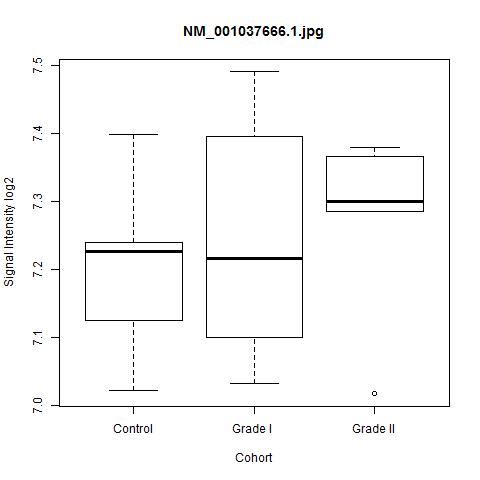

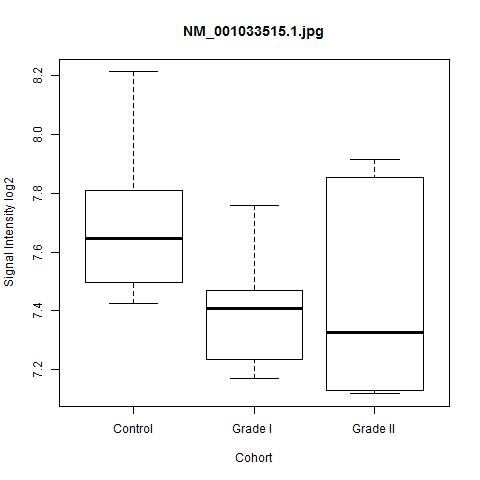

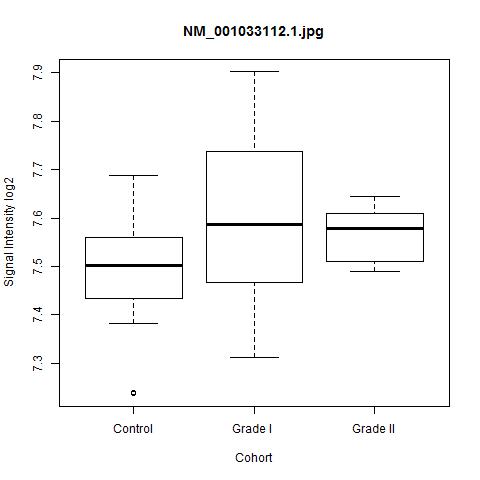

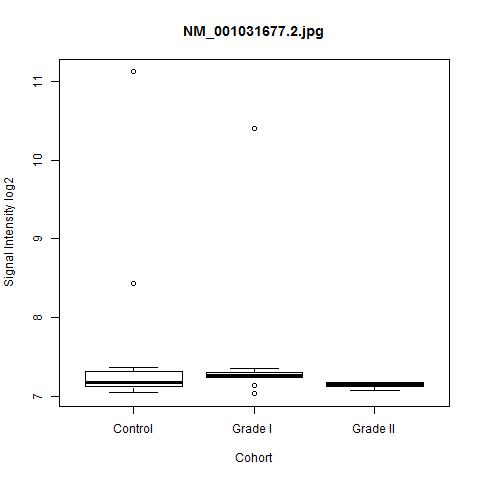

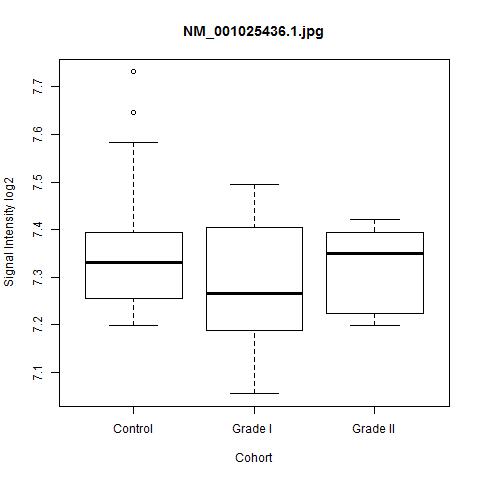

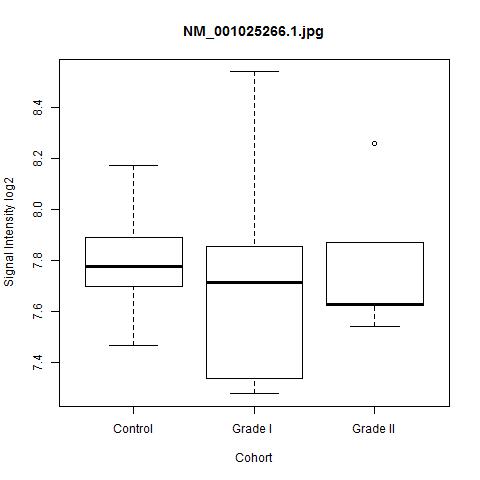

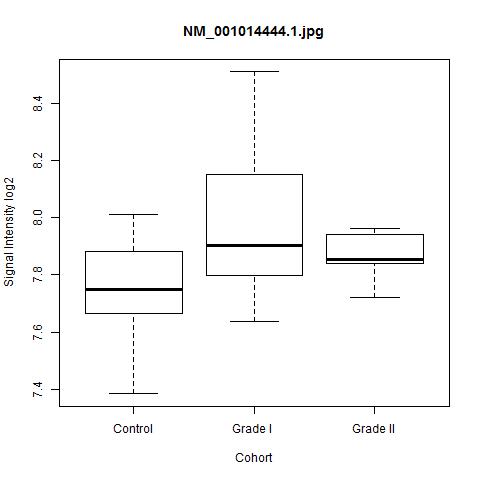

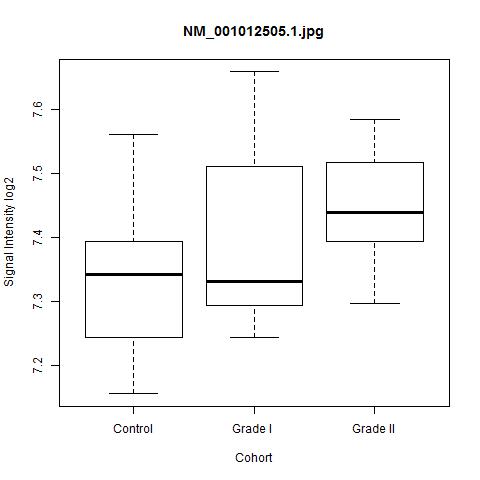

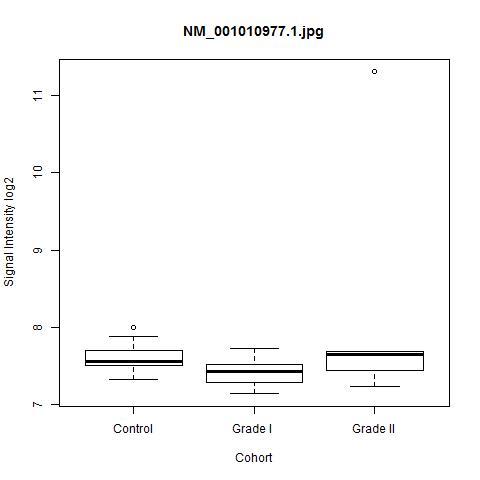

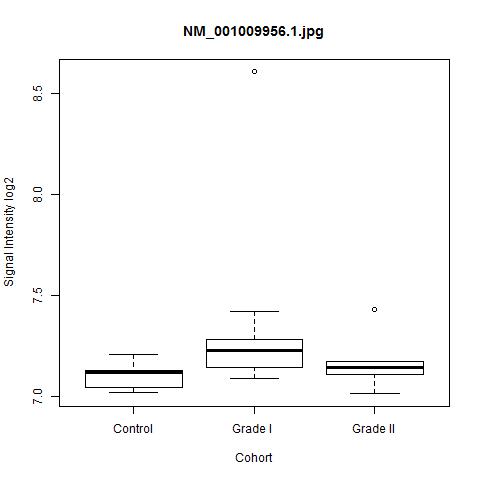

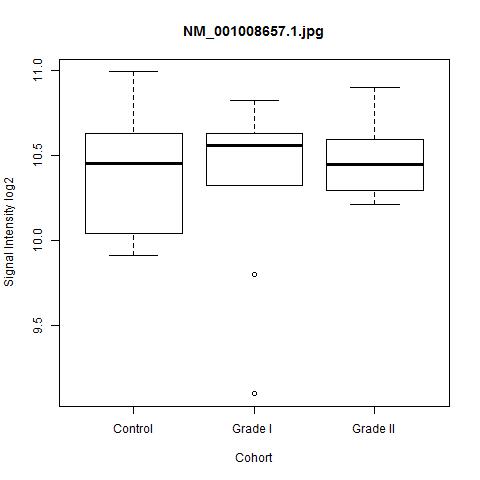

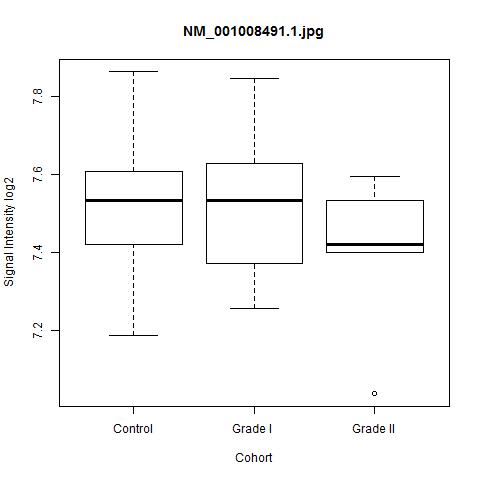

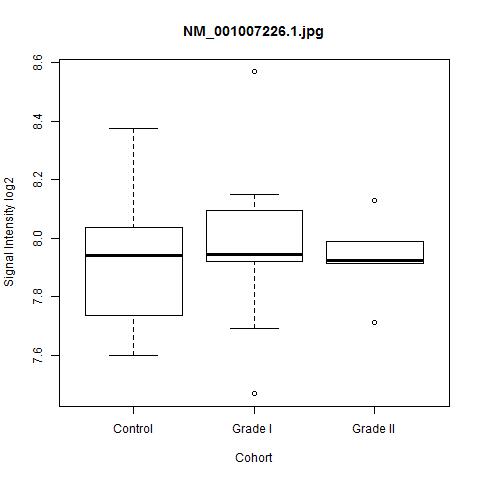

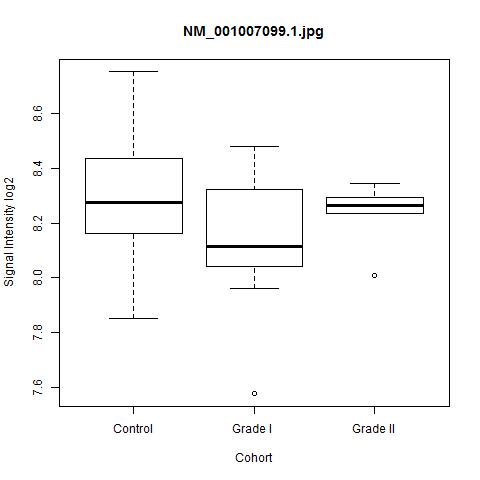

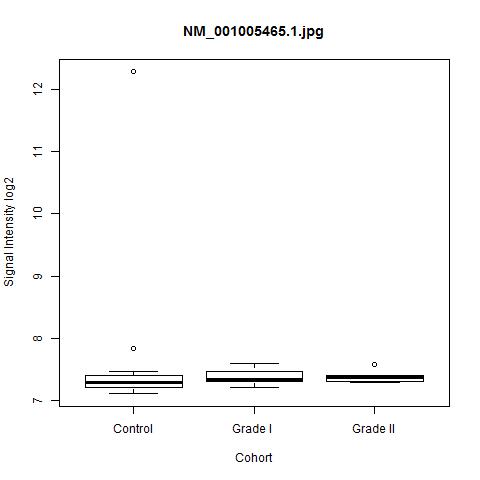

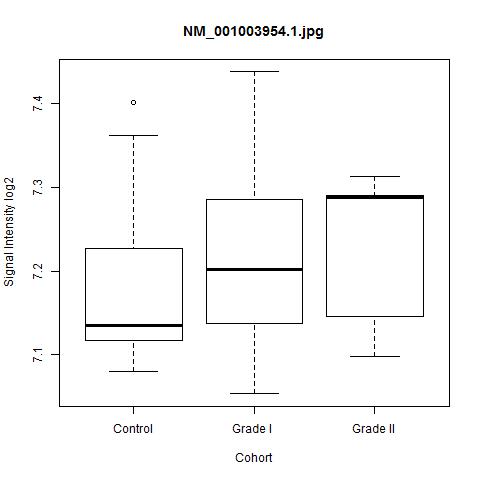

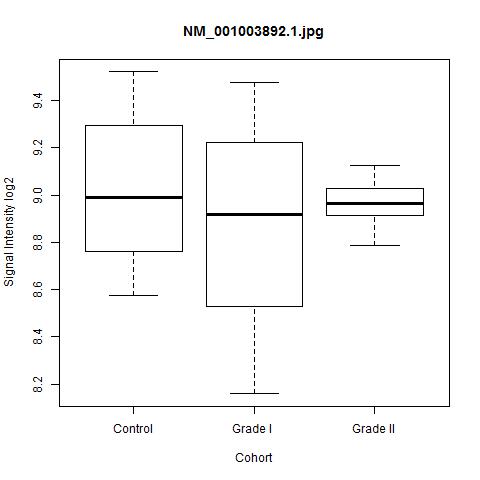

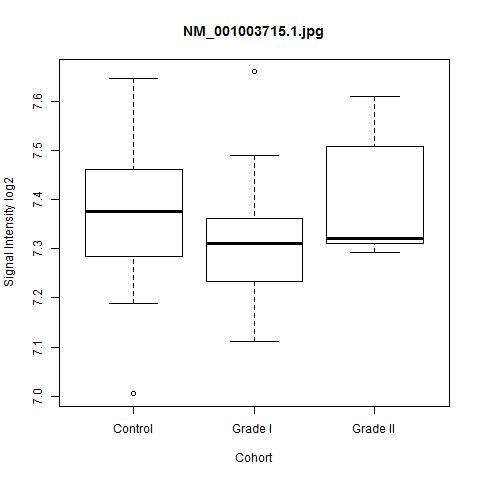

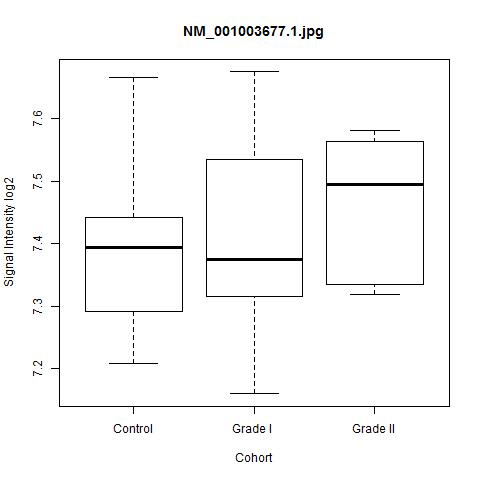

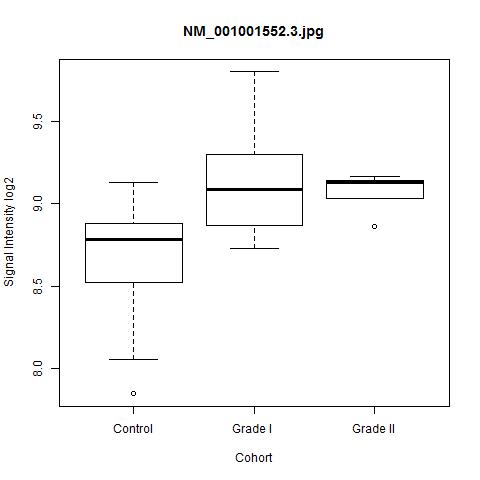

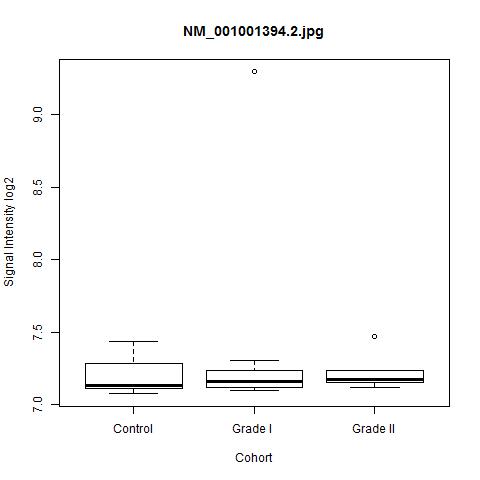

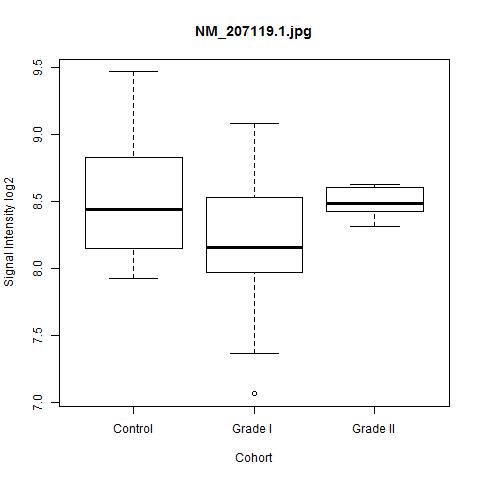

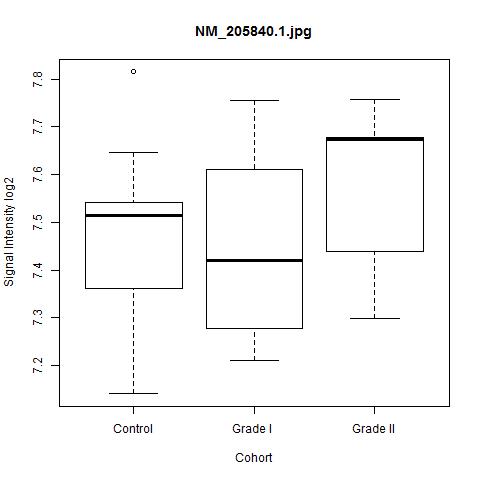

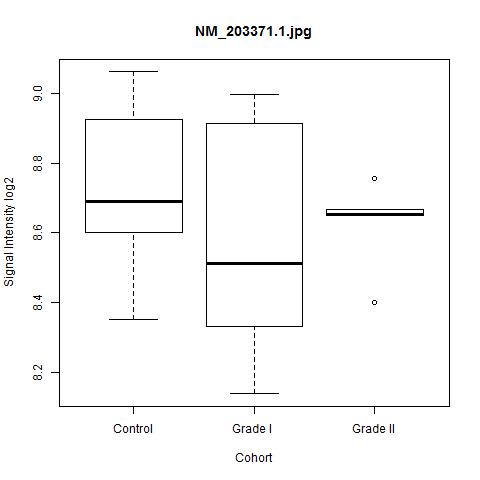

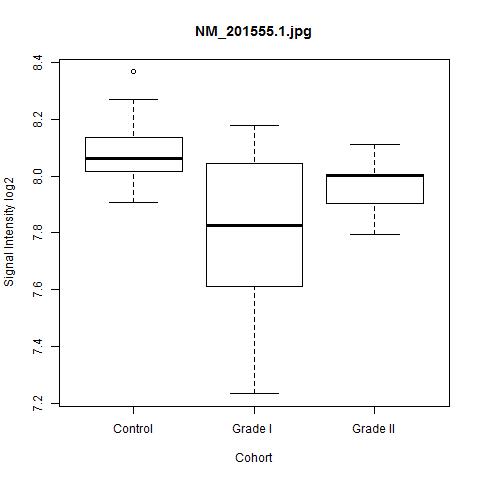

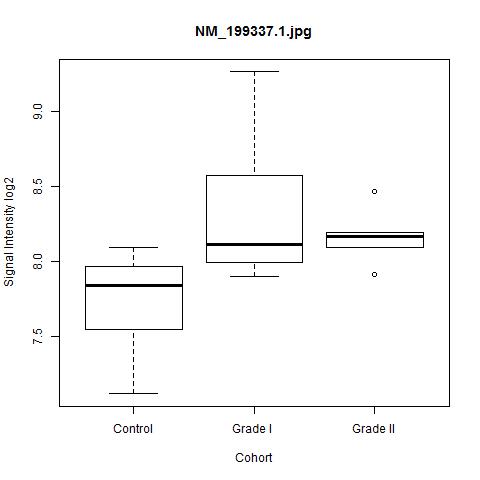

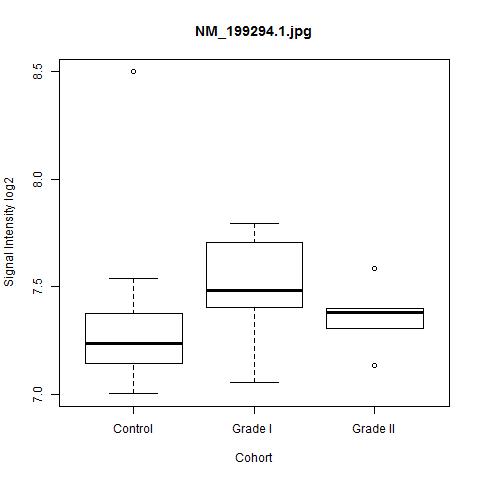

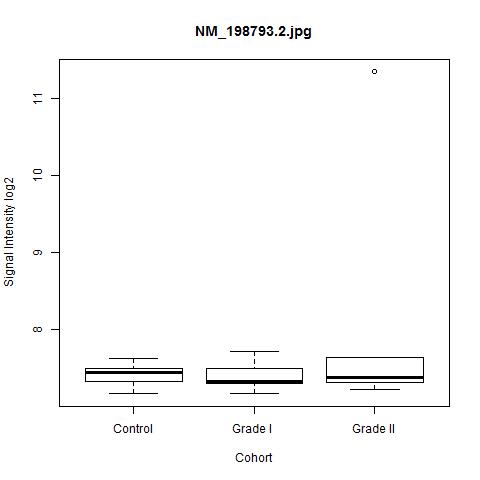

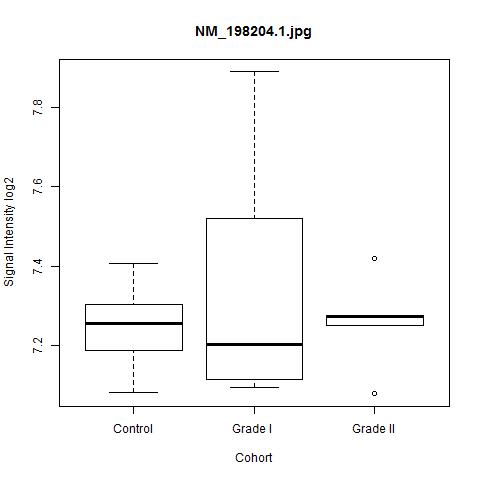

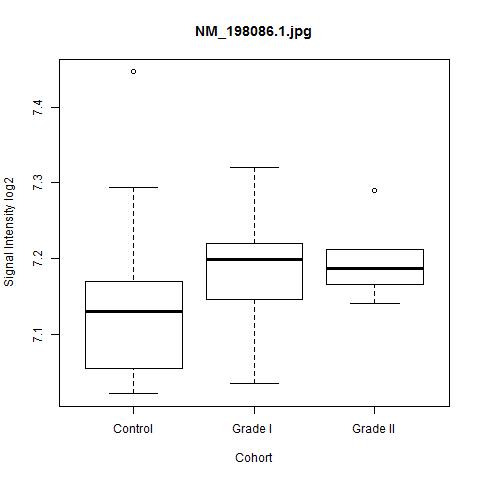

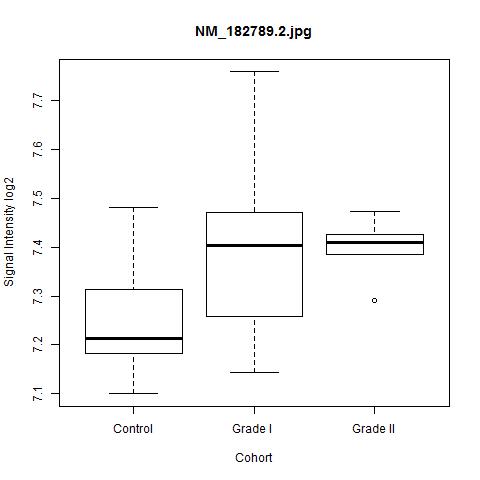

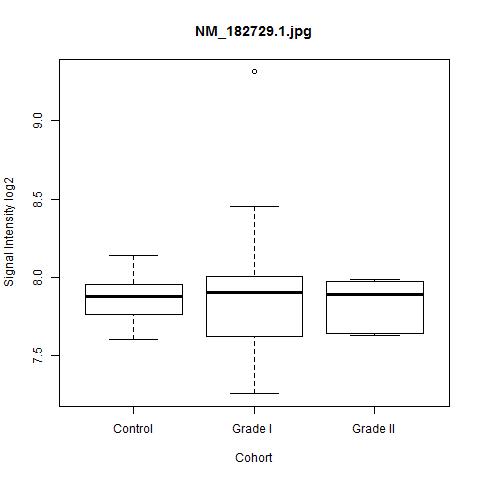

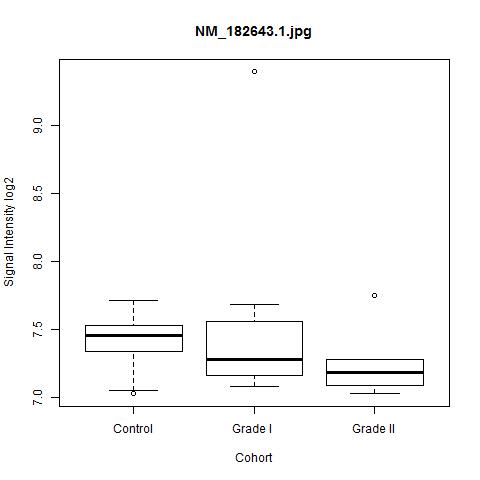

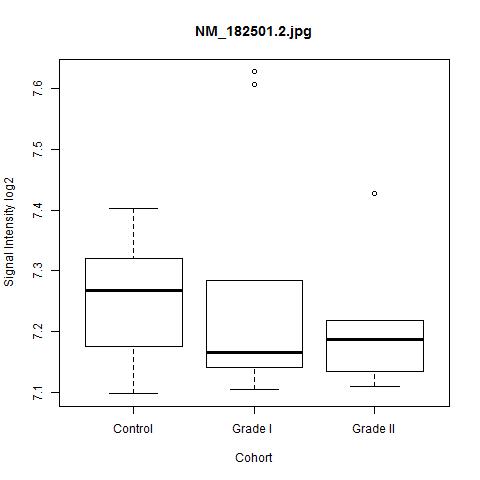

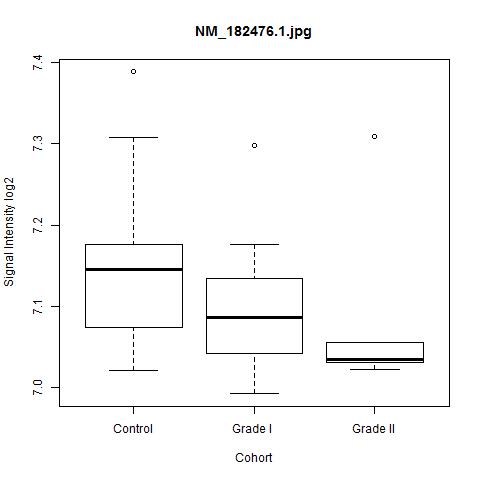

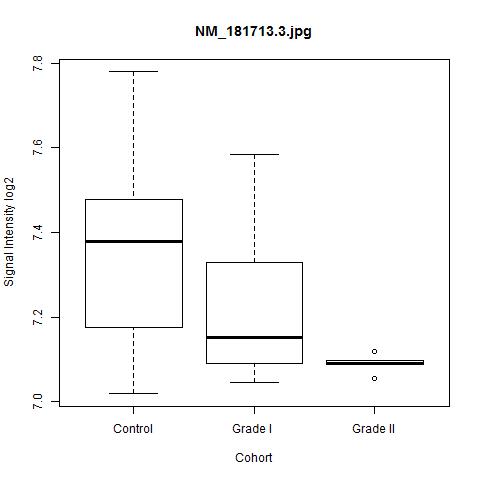

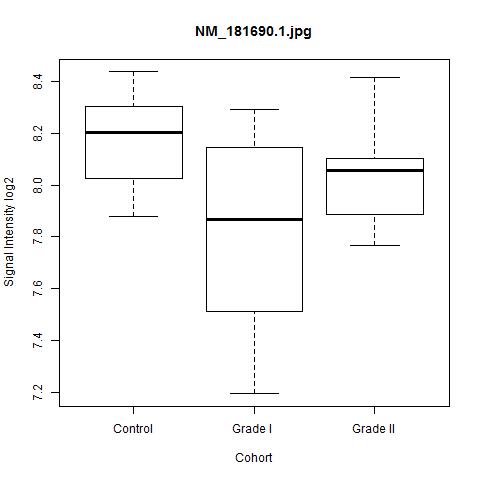

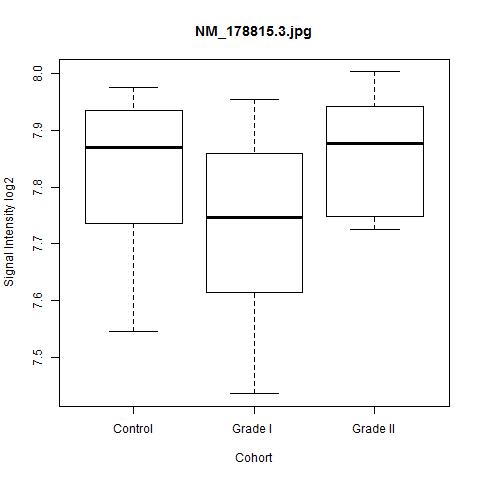

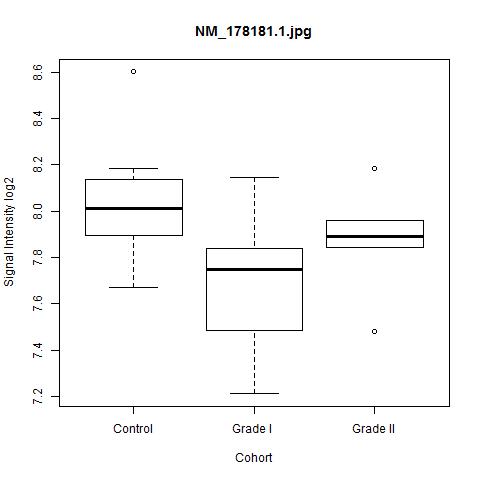

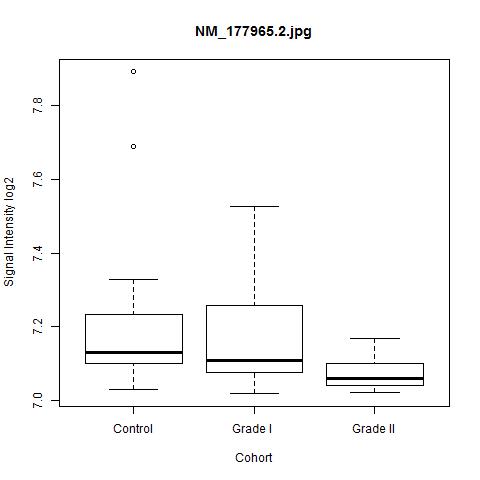

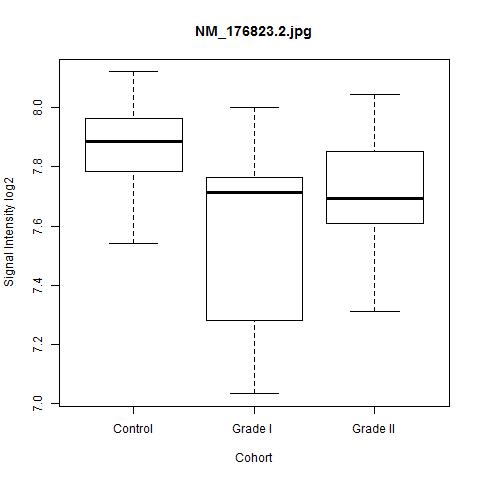

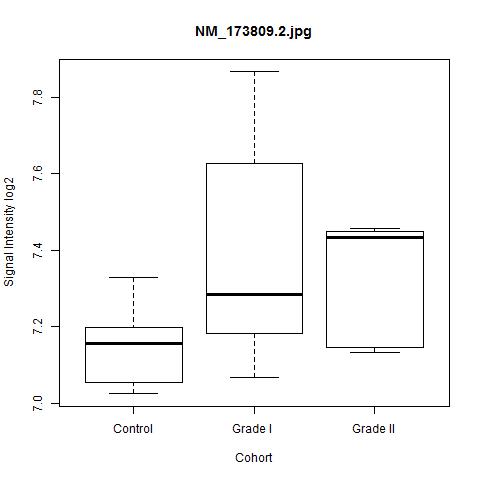

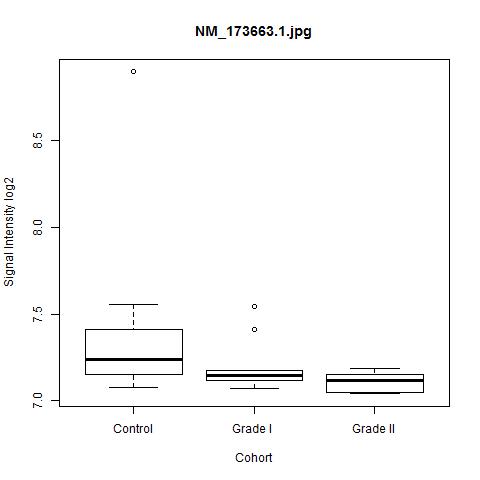

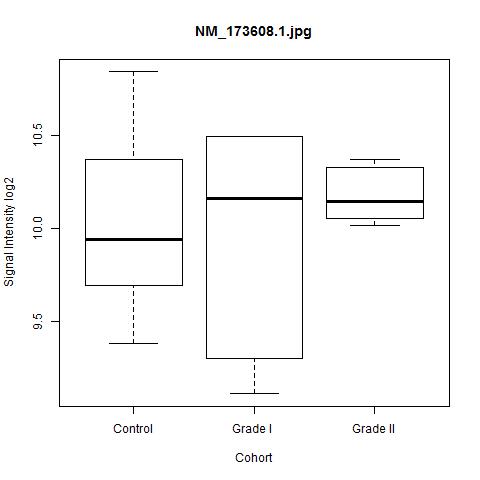

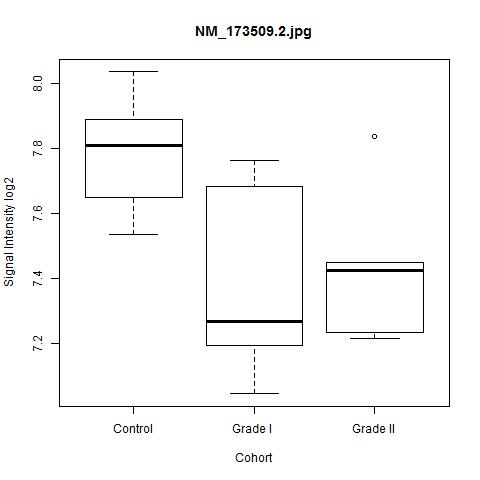

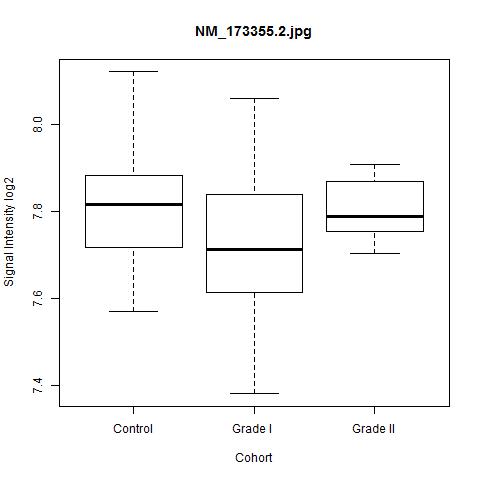

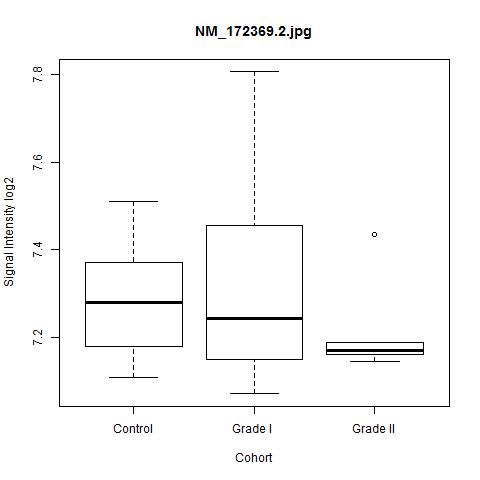

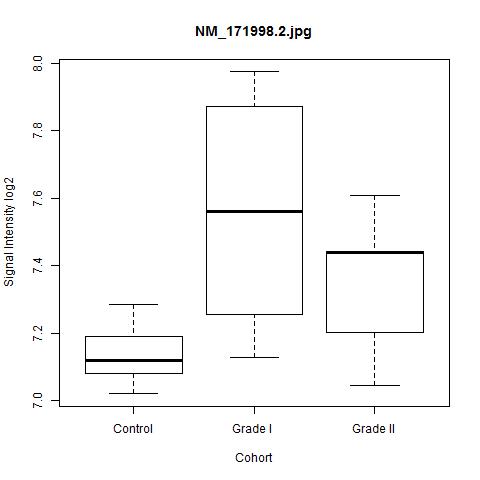

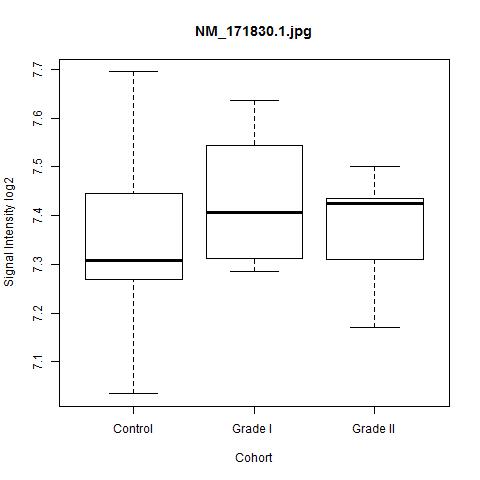

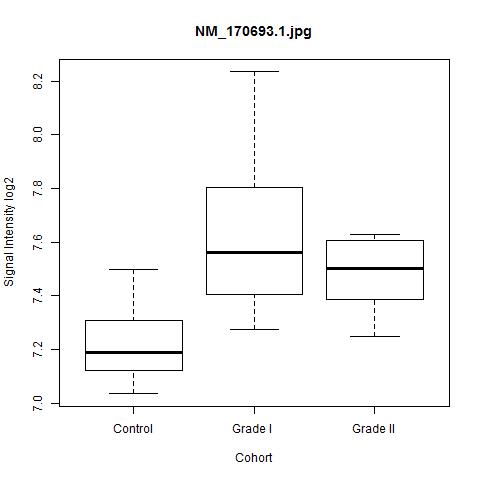

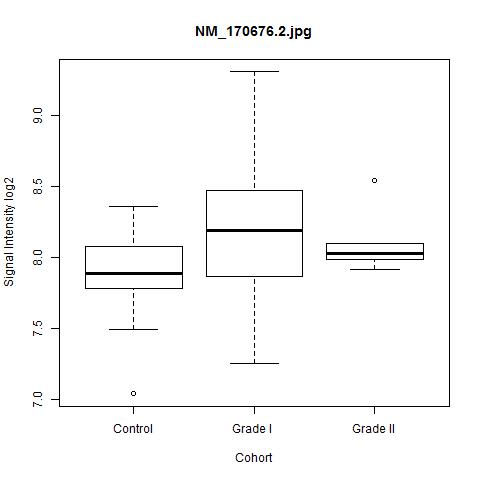

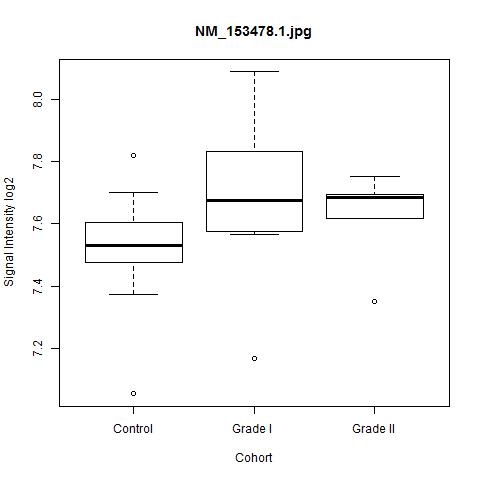

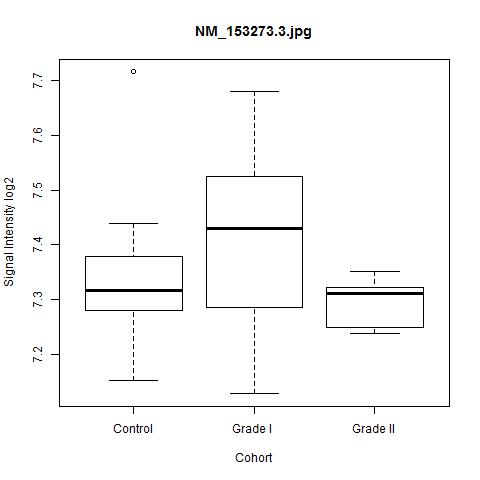

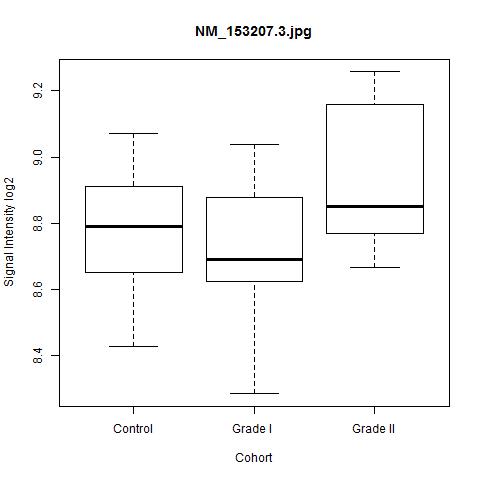

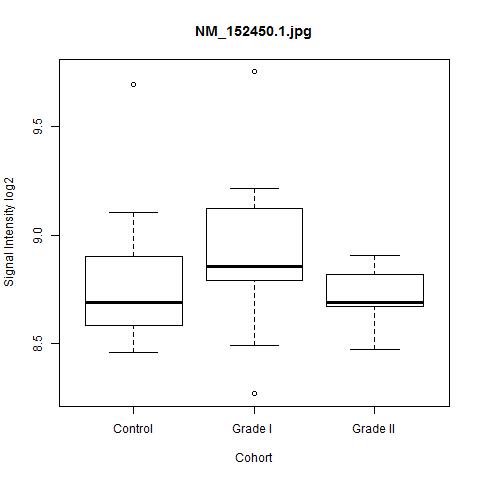

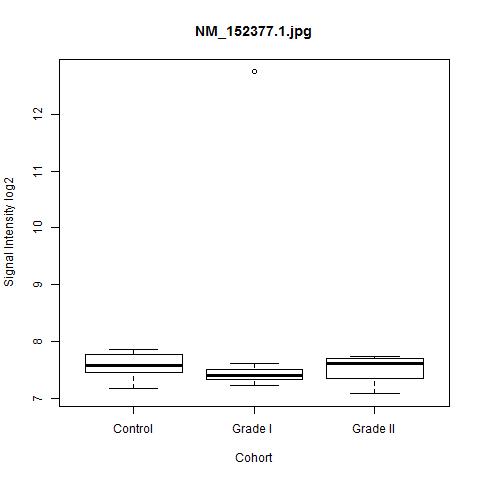

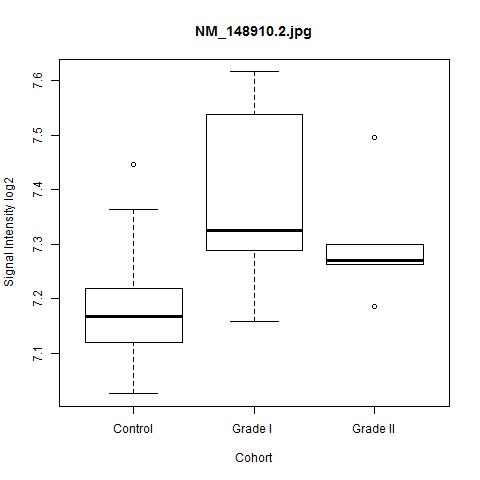

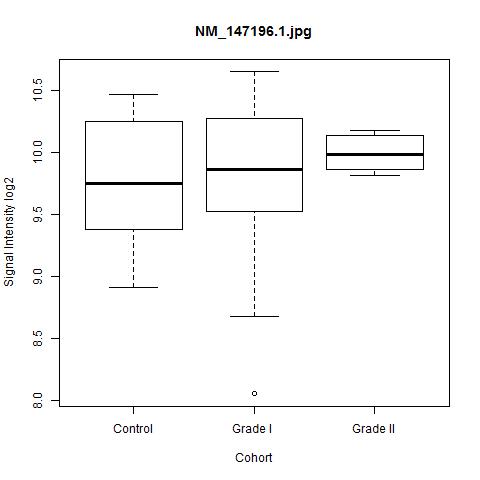

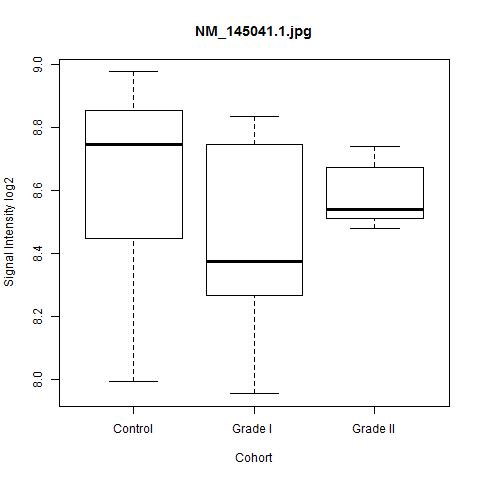

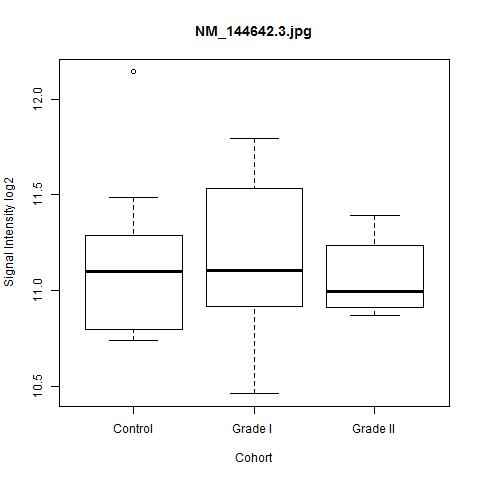

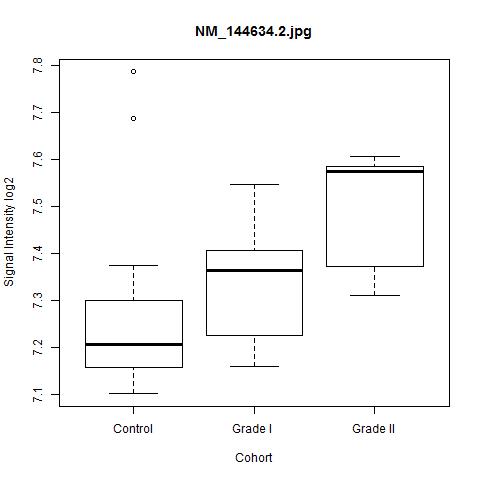

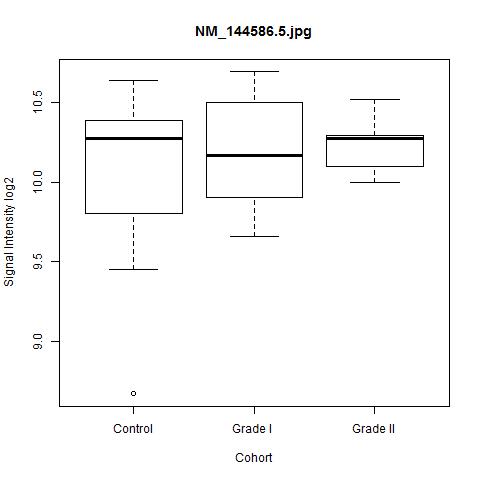

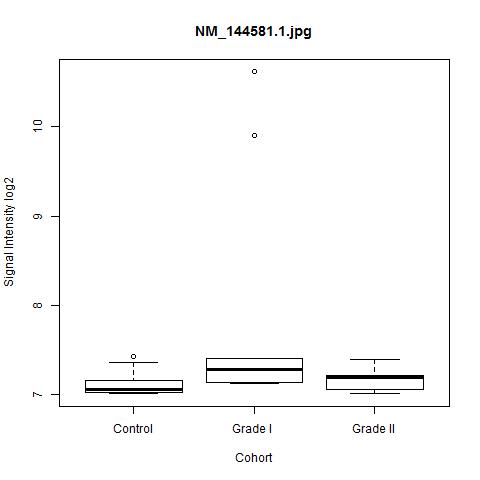

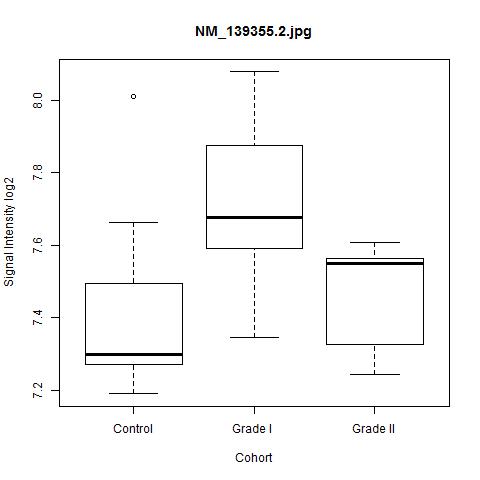

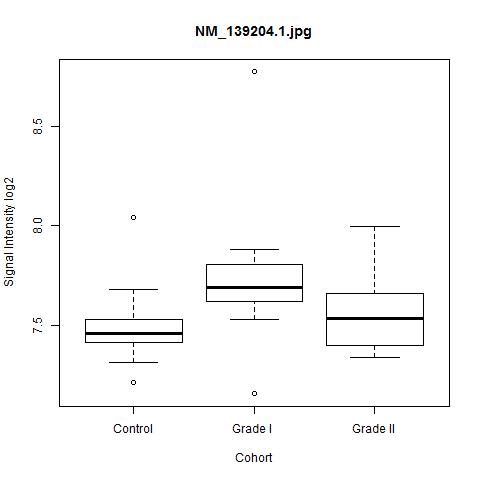

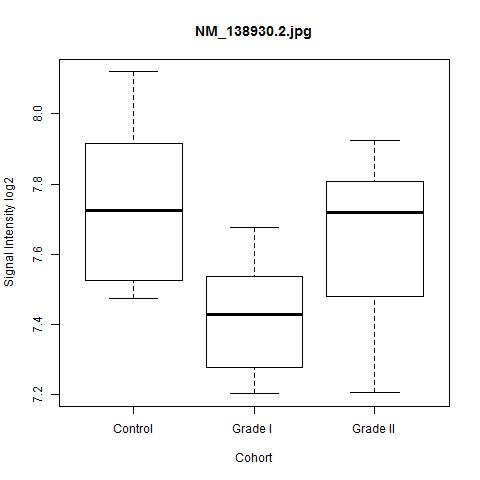

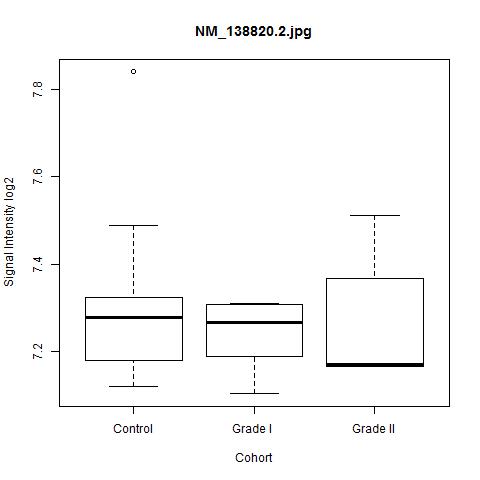

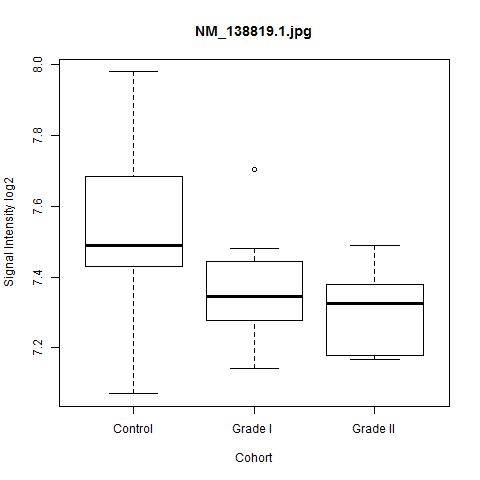

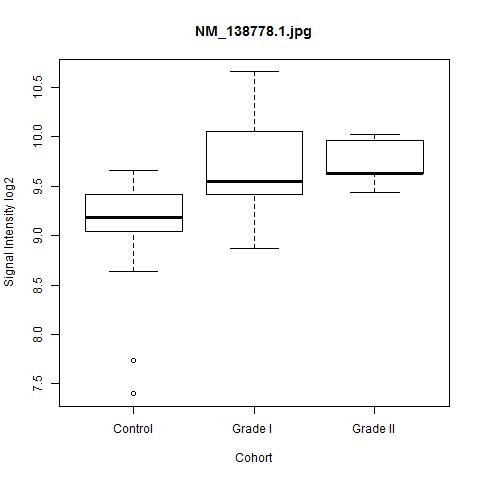

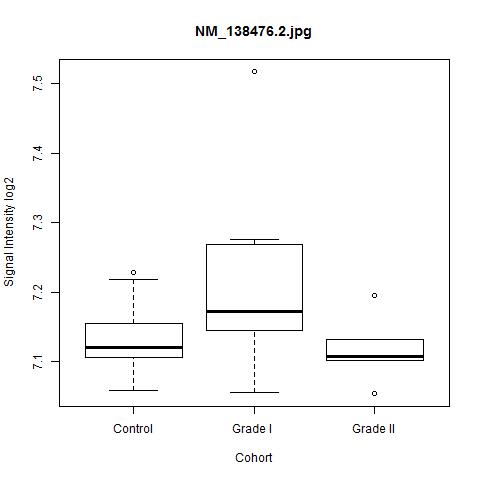

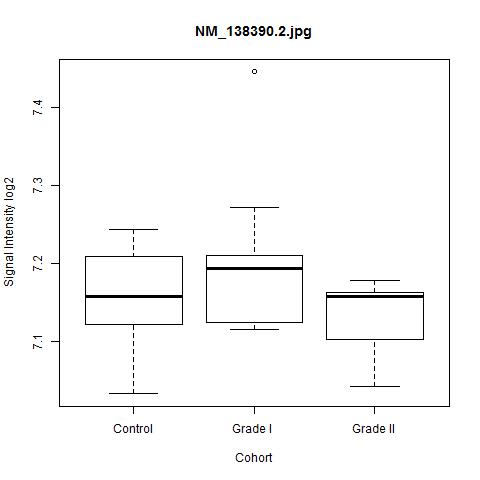

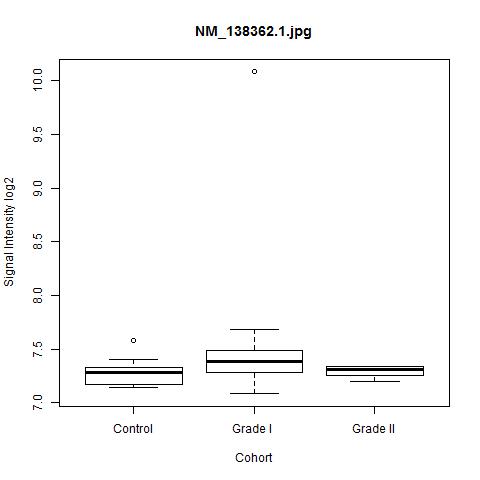

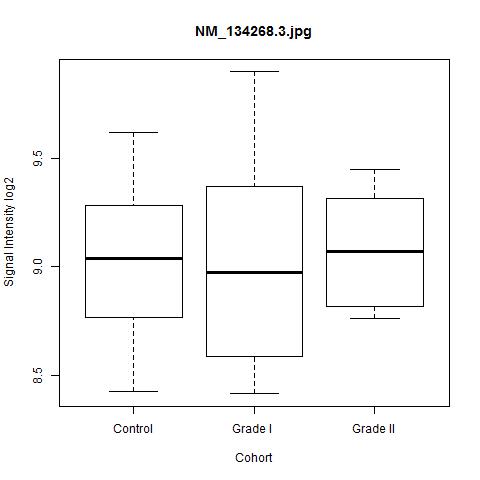

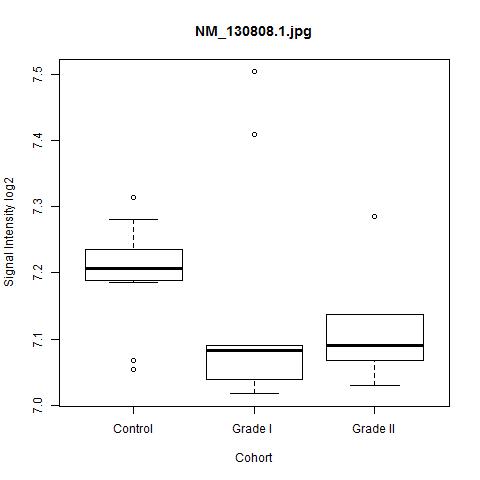

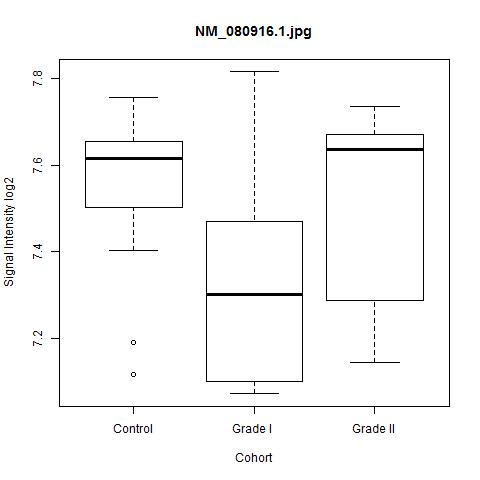

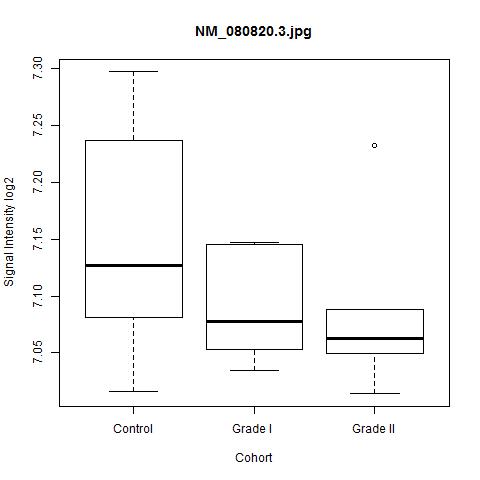

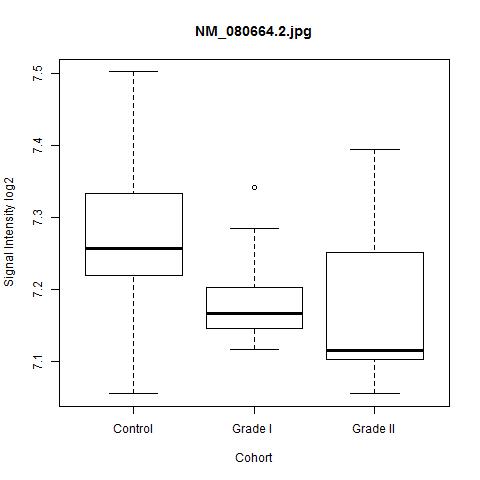

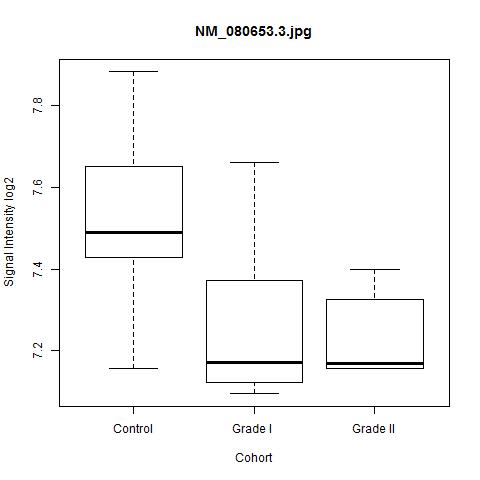

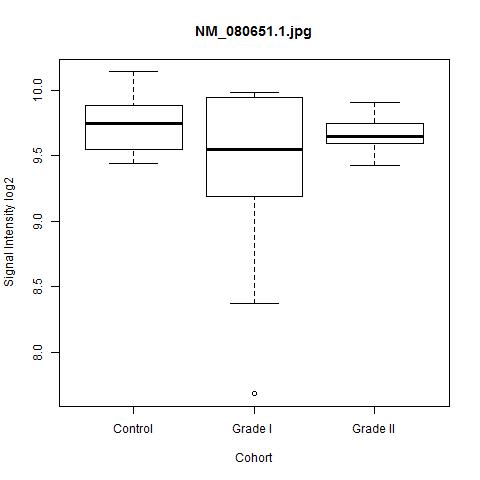

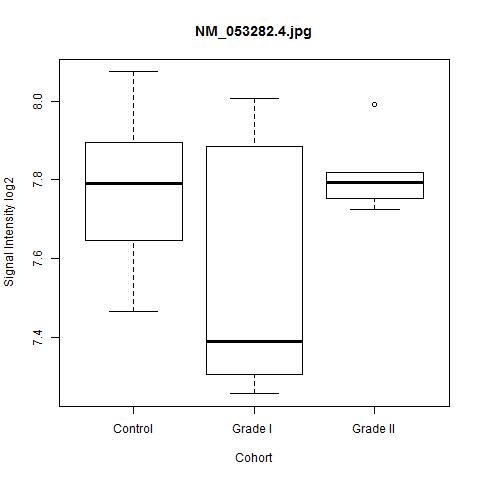

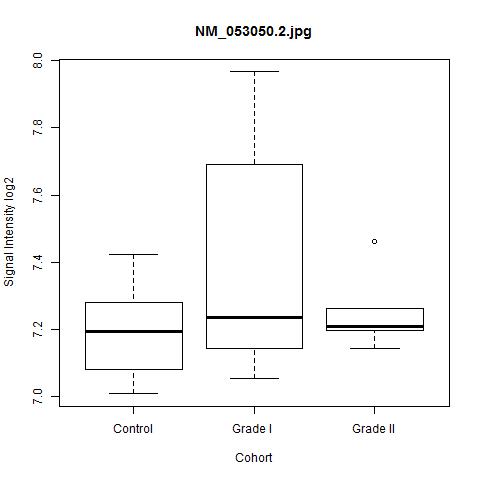

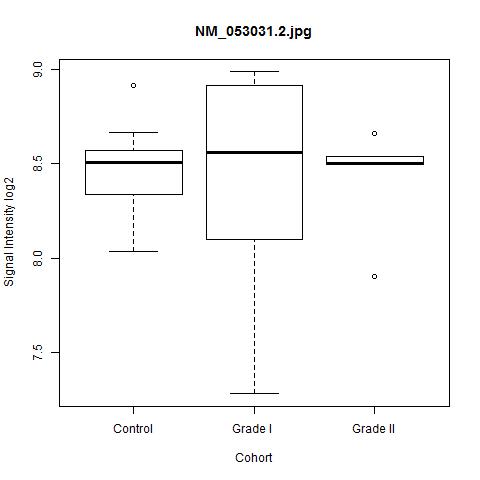

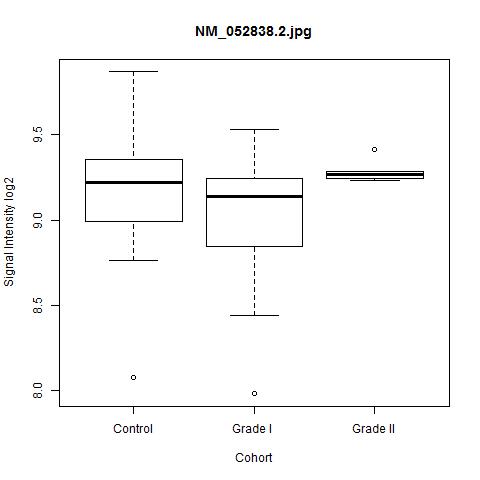

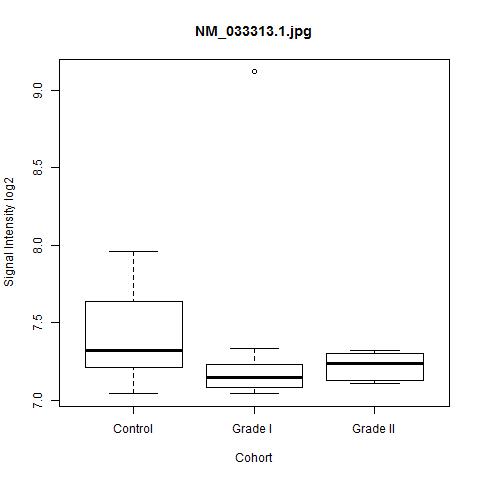

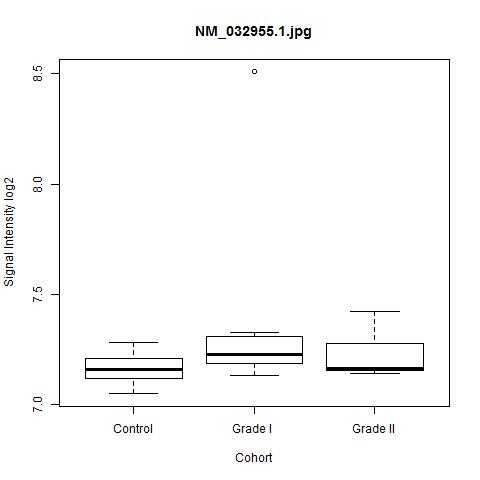

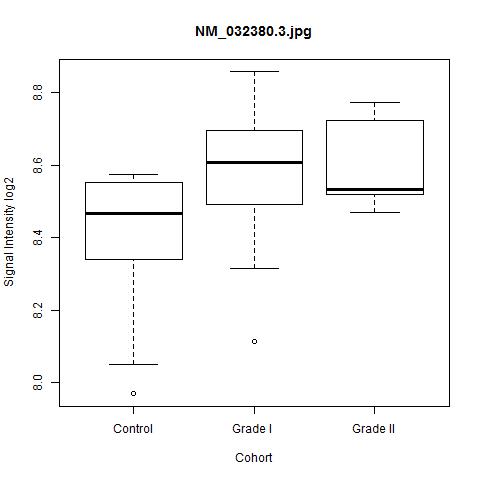

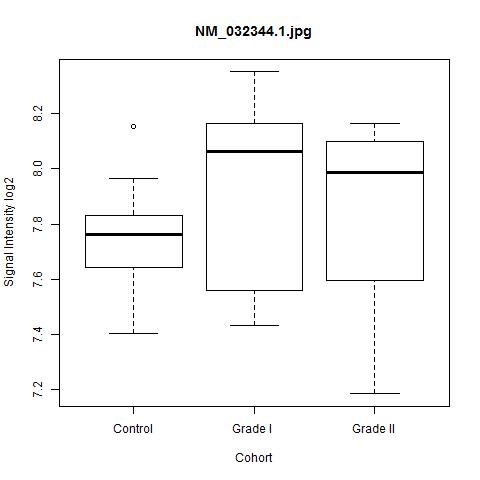

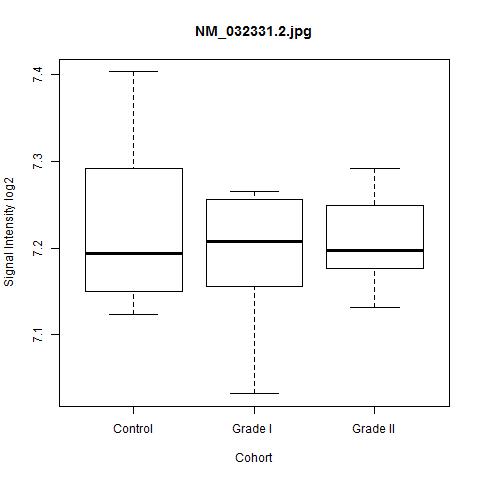

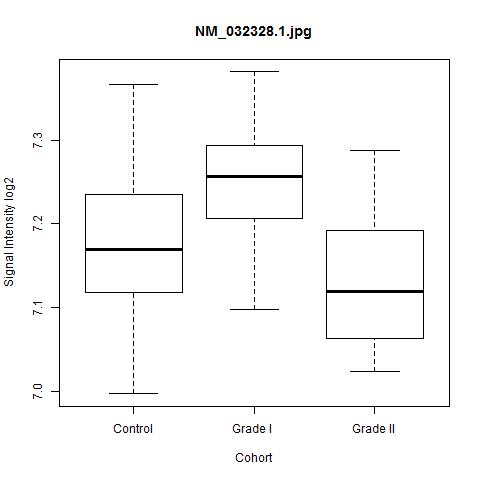

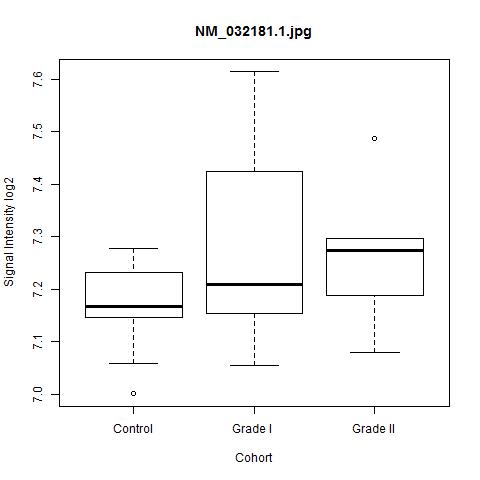

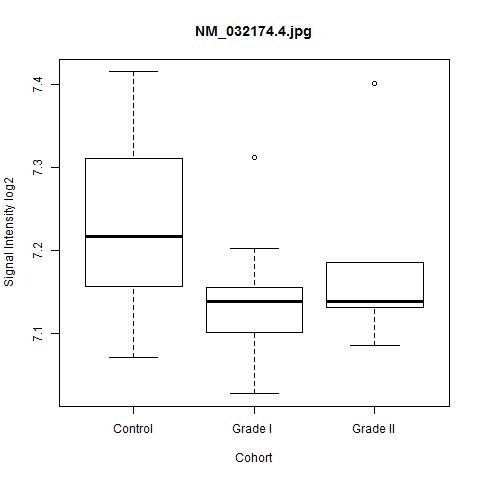

Supplement: Supplementary file 3 [file oncotarget-08-58443-s003.docx]
