# Supplementary material for: Evaluation of autoantibody signatures in meningioma patients using human proteome arrays
Source: Oncotarget. 2017 Apr 10;8(35):58443–56. doi: 10.18632/oncotarget.16997 (PMC5601665; doi:10.18632/oncotarget.16997)

**Supplementary Figure 4:** Immunoblotting images for SELENBP1 and TPD52L2.


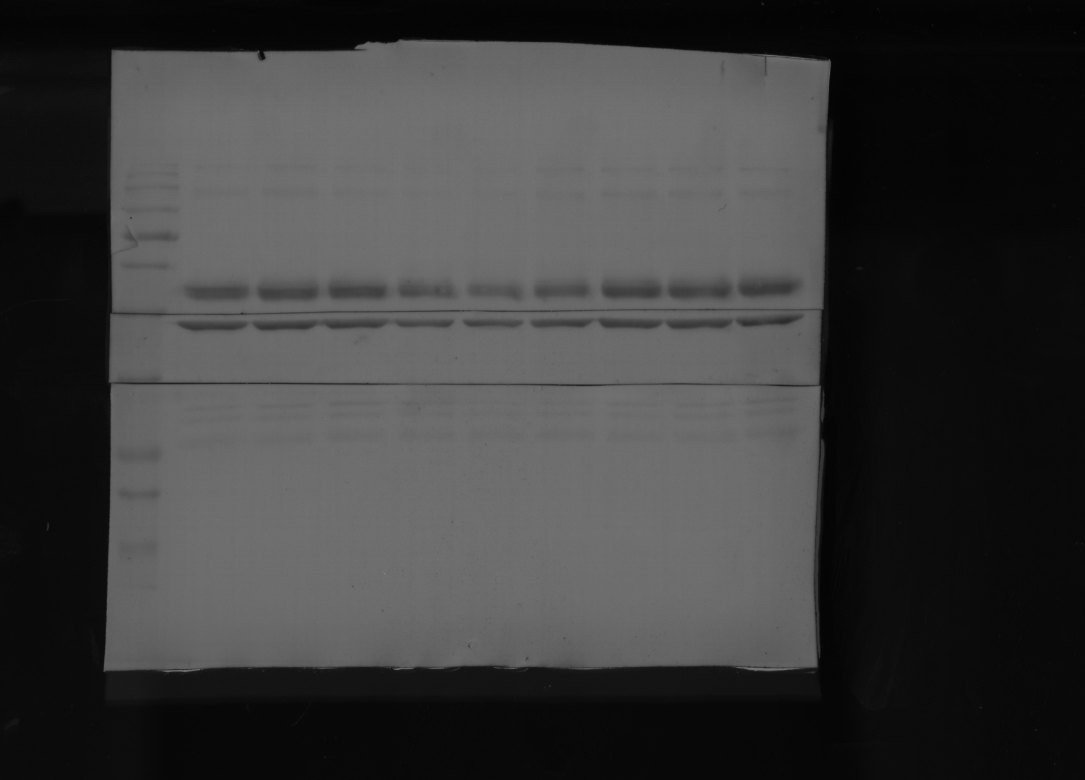


A. Raw image (TIFF) image of MG tissue lysates probed with antibodies specific

To SELENBP1 along with ACTB as loading control


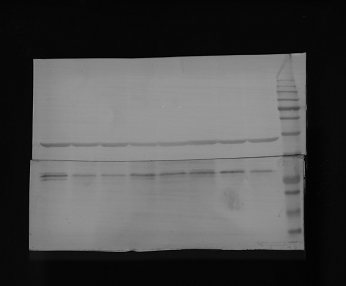


B.Raw image (TIFF) image of MG tissue lysates probed with antibodies specific

To TPD52L2 along with ACTB as loading control

**Information regarding Normal dura:**

**Additional information regarding normal dura:** The normal dura had fainter expression profiles and this is one of the clinical limitation of such samples as due to ethical reasons large amount of dura mater cannot be excised from the patients. As a support of this point the immunoblots wherein we have performed immunoblots of various samples including meningiomas, metastatic samples for a comparing expression profiles of not only beta actin but also other proteins like CLIC1 that were not part of the current study.


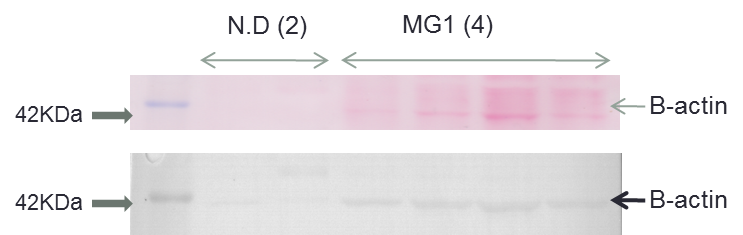


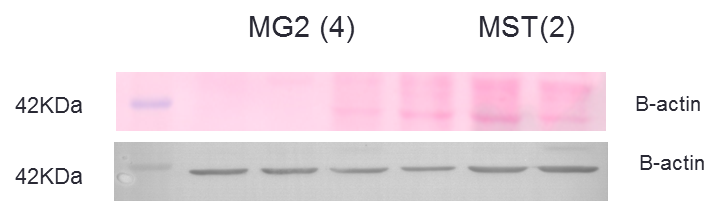


Quantitation of ACTB levels using IQTL

**Footnote:** ND: Refers to Normal Dura Mater, MST: Refers to highly aggressive tumor lysates that were also used to compare the expression profiles of all the proteins of interest along with the several grades of meningiomas as well as normal dura mater.

Antibody dilution used: Primary: 1:2000, Secondary: 1:6000
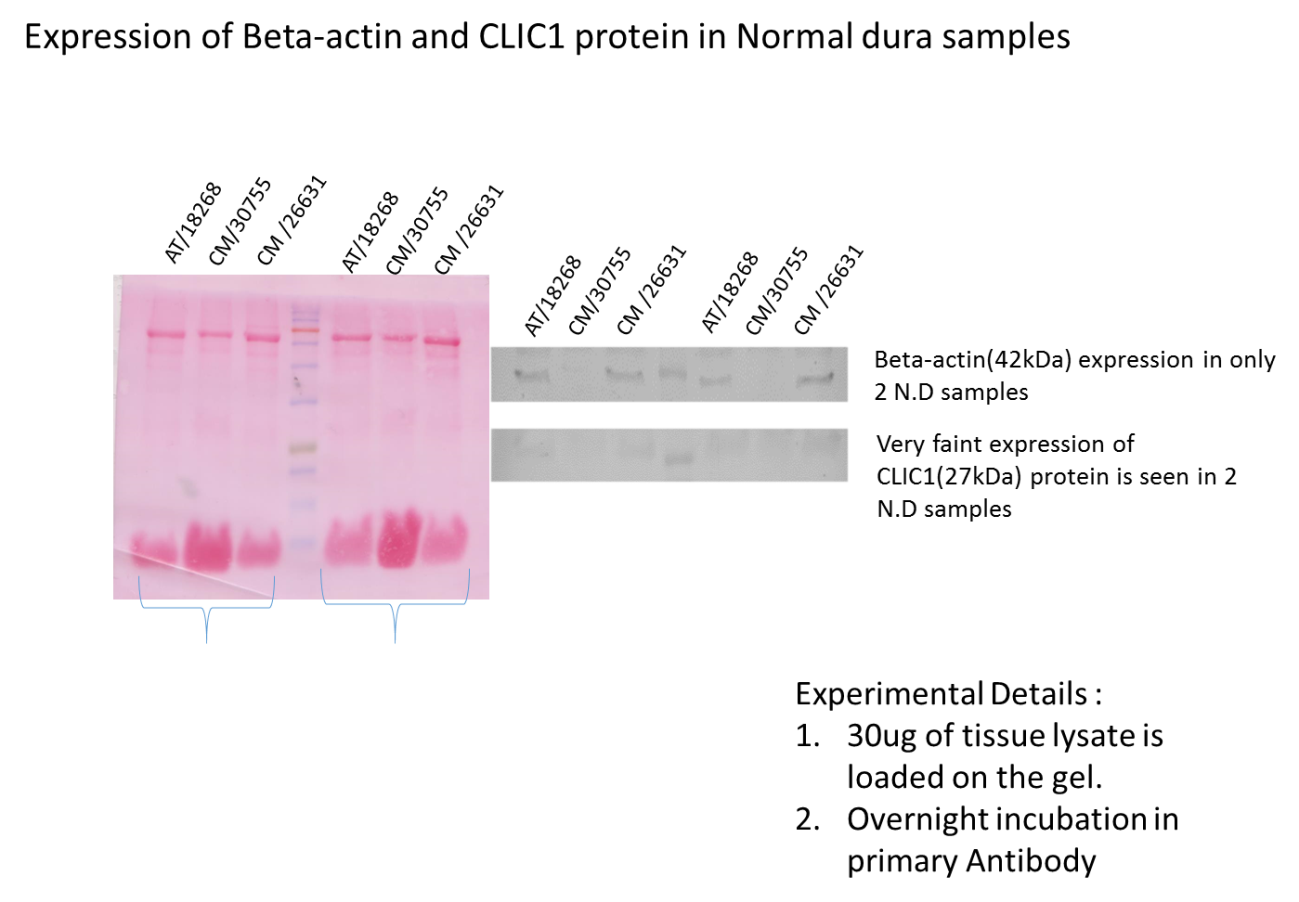

Supplement: Supplementary file 5 [file oncotarget-08-58443-s005.docx]
