# Supplementary material for: Evaluation of autoantibody signatures in meningioma patients using human proteome arrays
Source: Oncotarget. 2017 Apr 10;8(35):58443–56. doi: 10.18632/oncotarget.16997 (PMC5601665; doi:10.18632/oncotarget.16997)

**Supplementary Figure 5**: MCL clusters generated via STRING DB V 10.0

HC vs MG1_MCL Index 0.


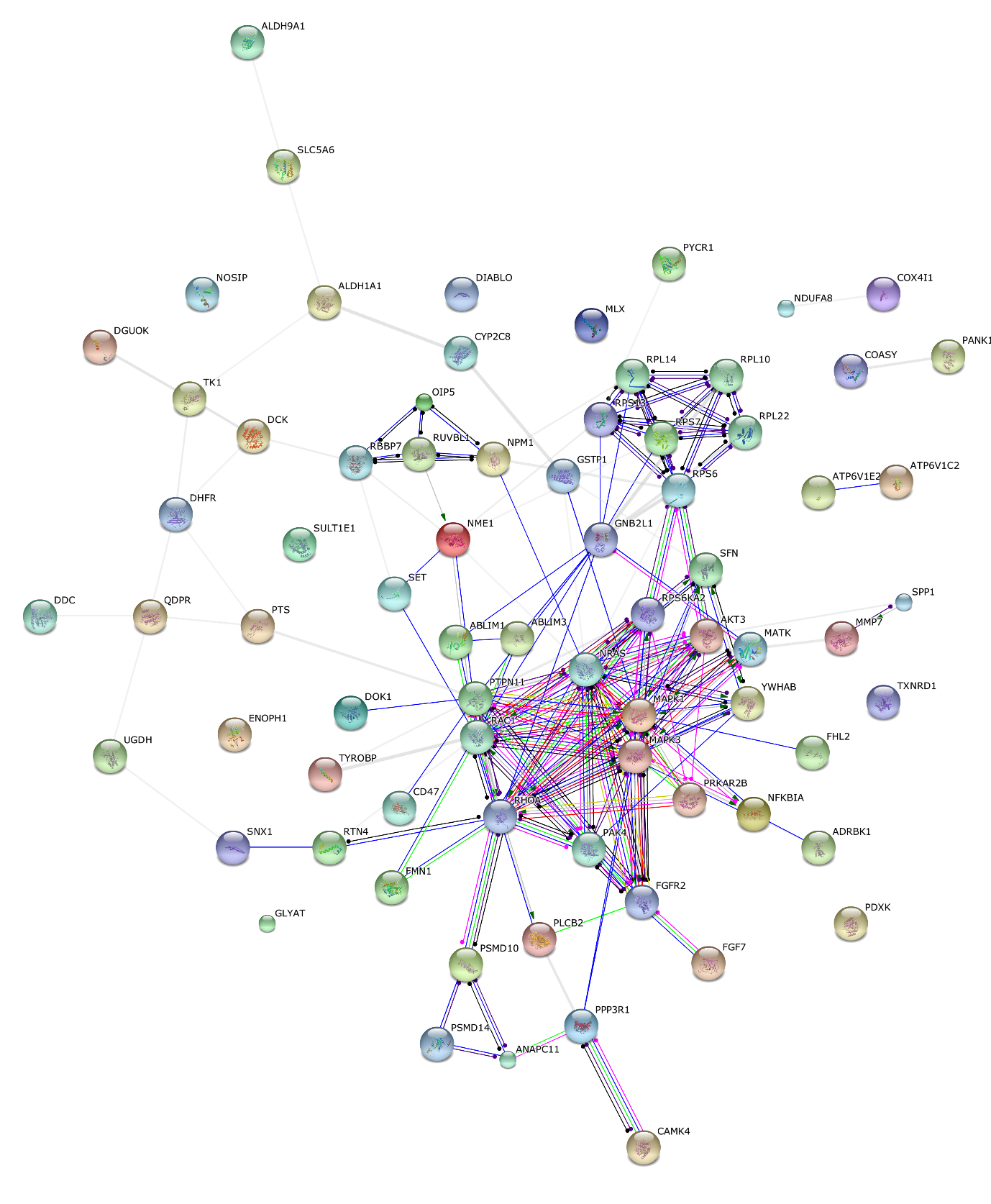


HC vs MG1_MCL Index 1


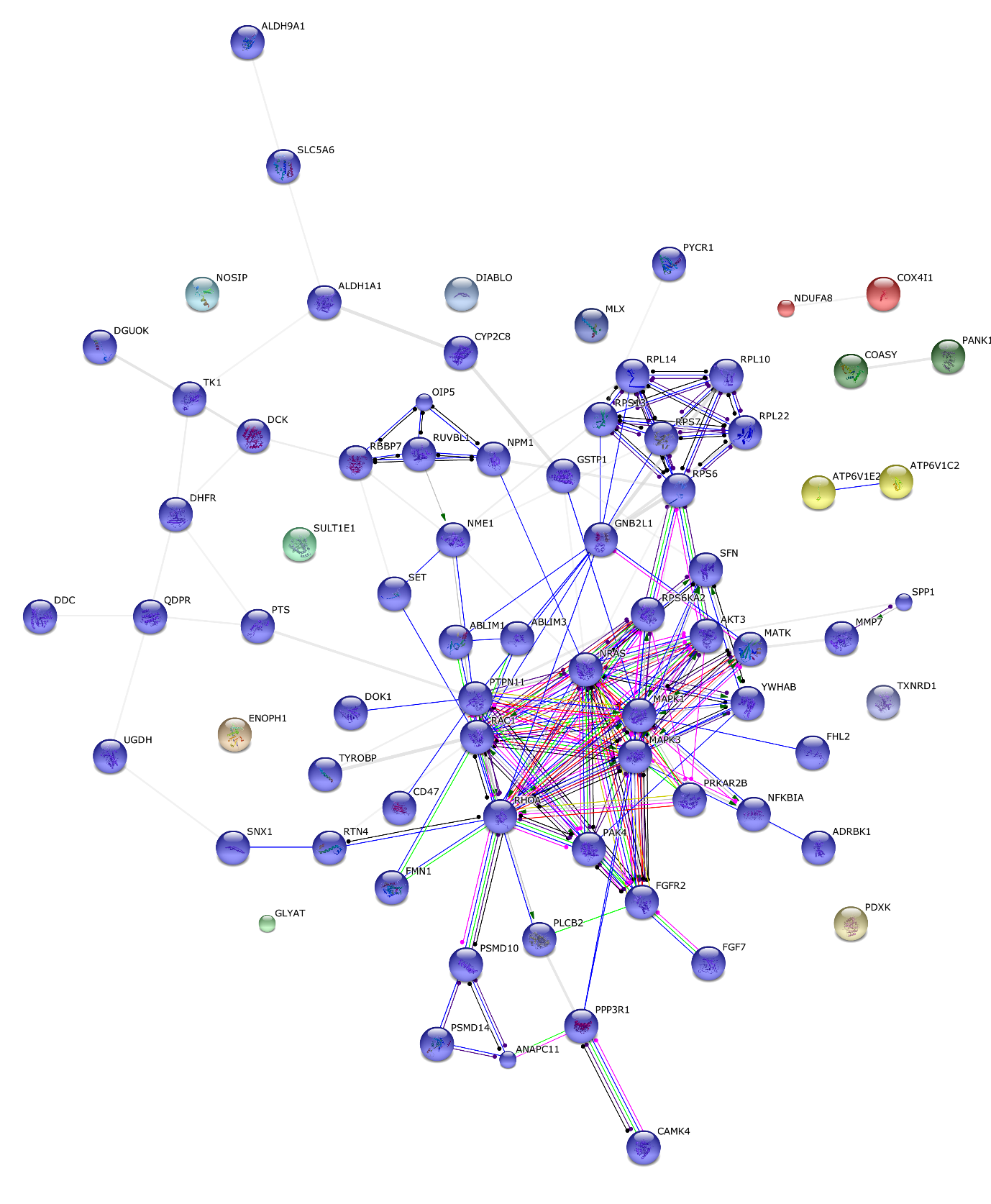


HC vs MG1_MCL Index 2


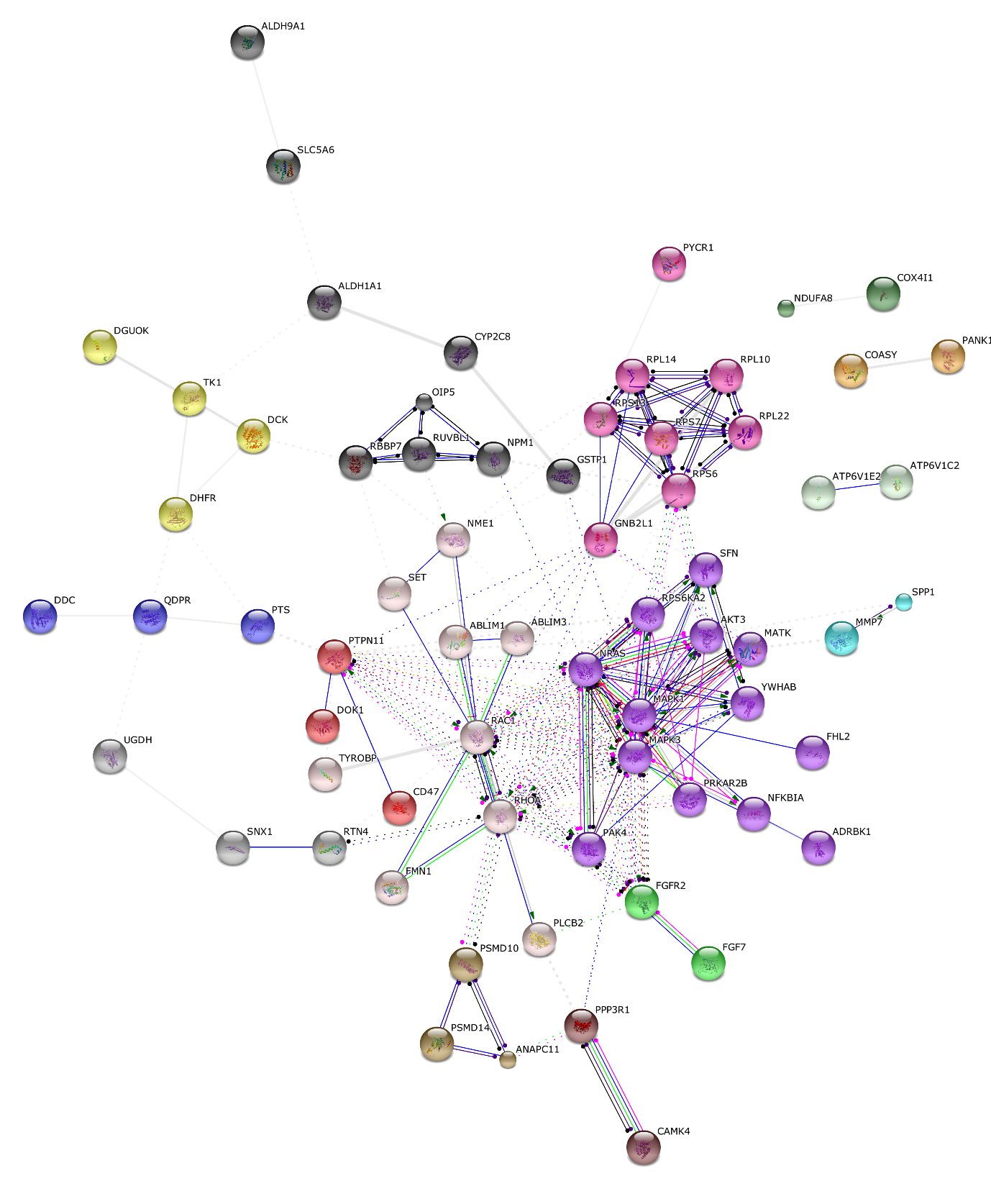


HC vs MG1_MCL Index 3


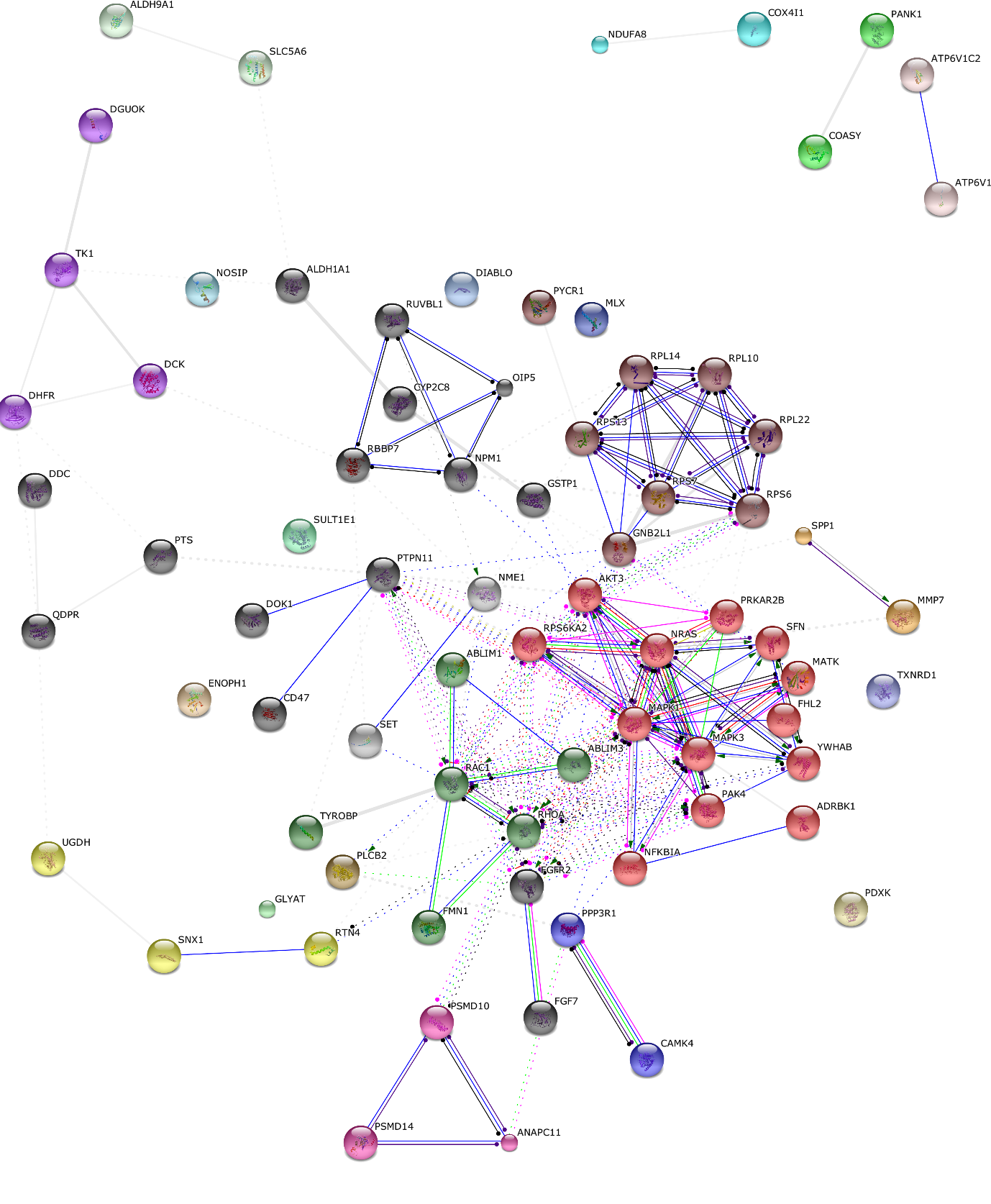


HC vs MG1_MCL Index 4


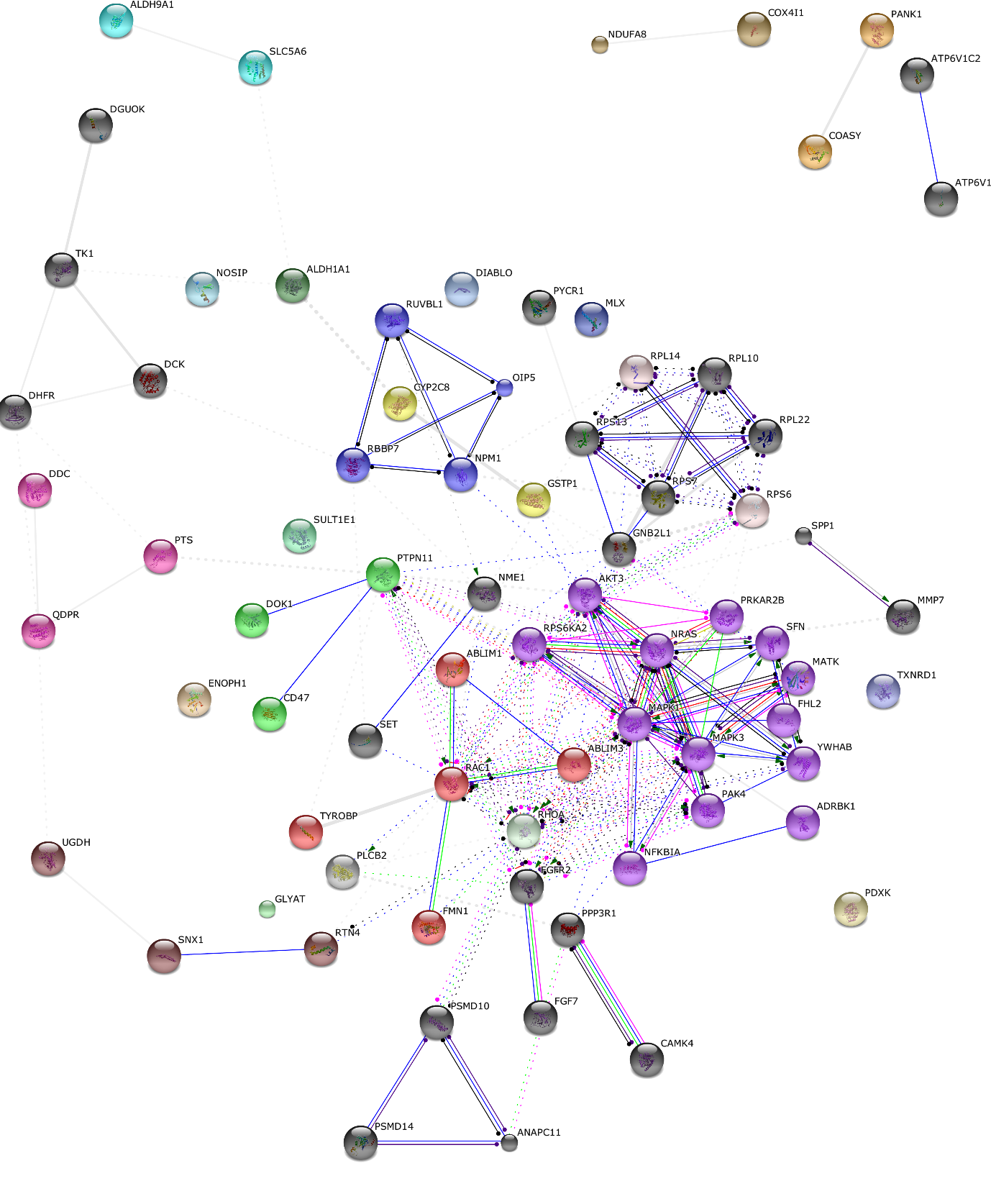


HC vs MG1_MCL Index 5


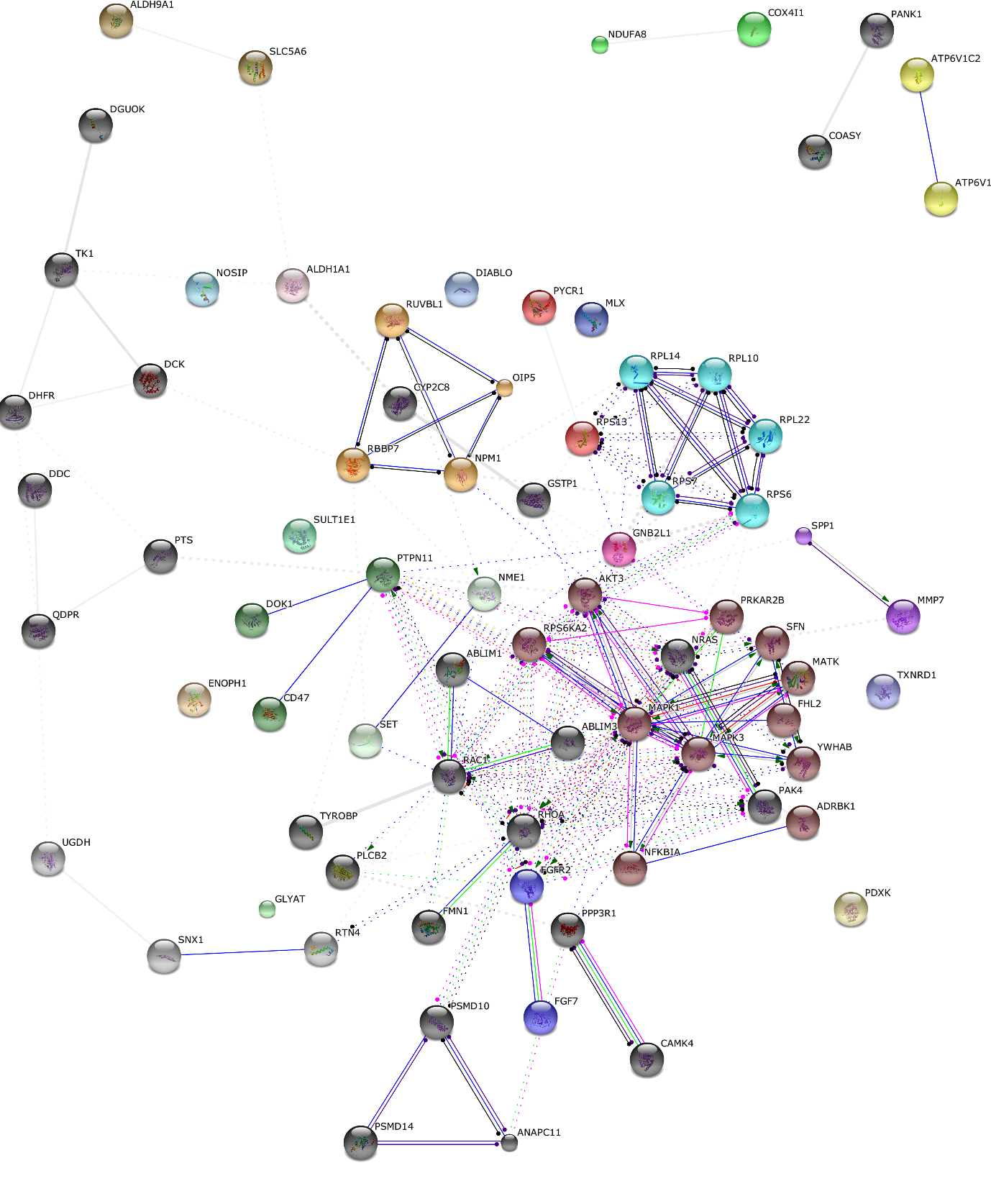


HC vs MG2_MCL Index 0


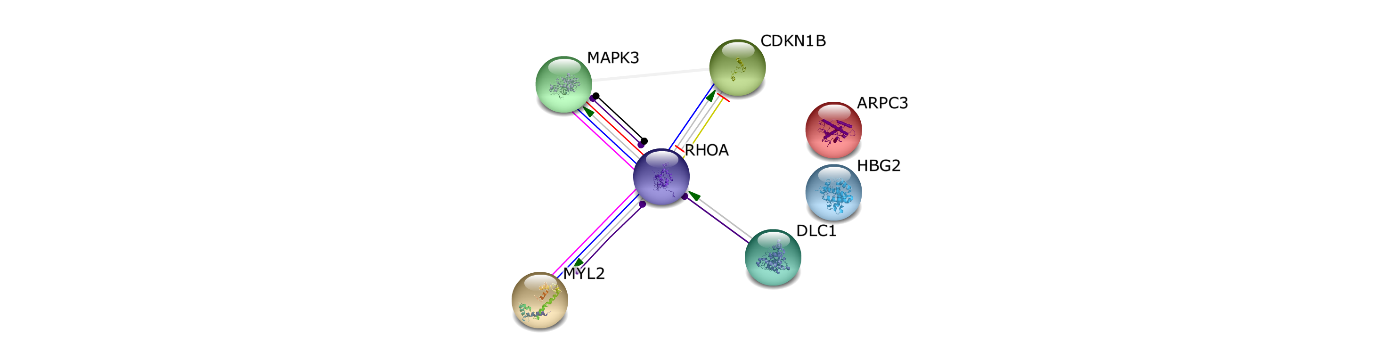


HC vs MG2_MCL Index 5


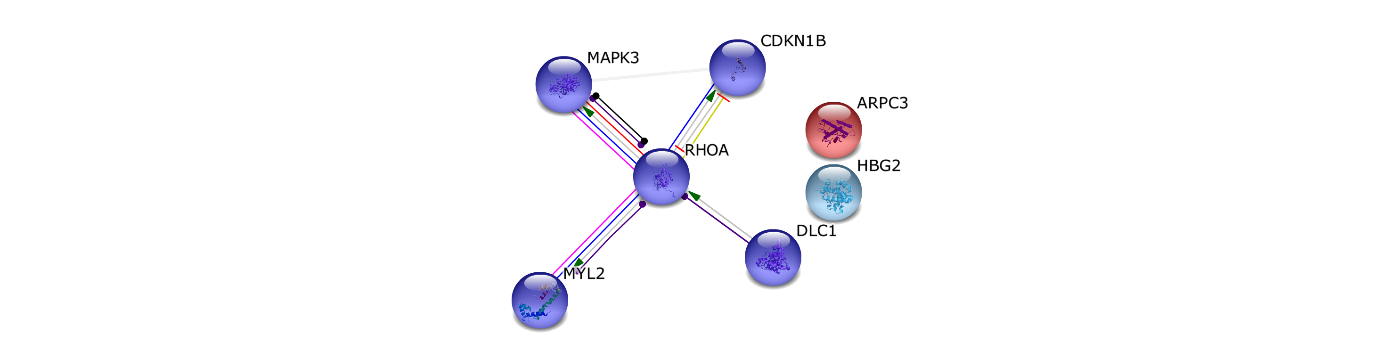

Supplement: Supplementary file 6 [file oncotarget-08-58443-s006.docx]
