# Supplementary material for: Evaluation of autoantibody signatures in meningioma patients using human proteome arrays
Source: Oncotarget. 2017 Apr 10;8(35):58443–56. doi: 10.18632/oncotarget.16997 (PMC5601665; doi:10.18632/oncotarget.16997)

**Supplementary Figure 6:** Raw scatter plot outputs of GO outputs from STRING DB V 10.0 using REVIGO.

HC vs MG1


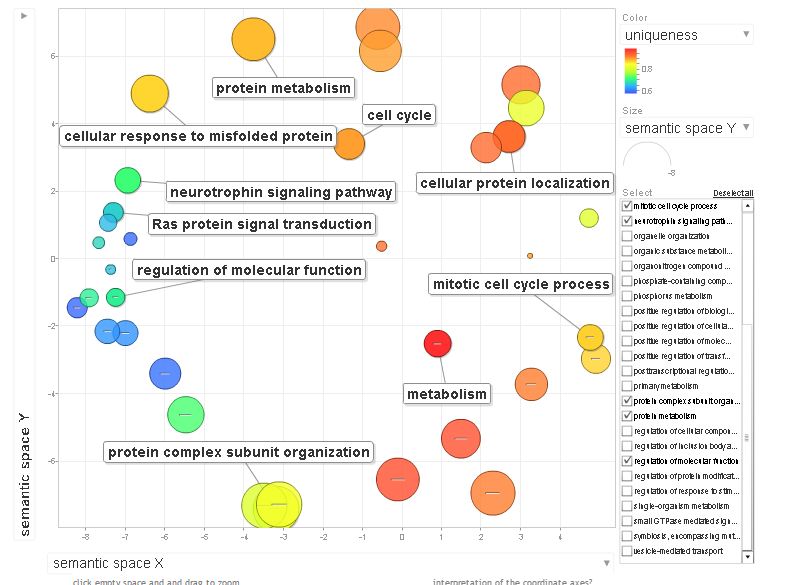


HC vs MG 2


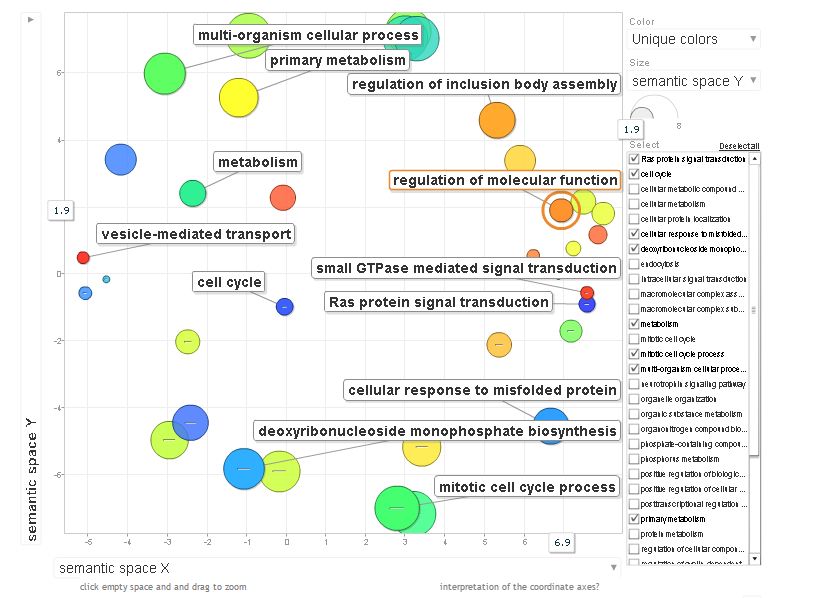

Supplement: Supplementary file 7 [file oncotarget-08-58443-s007.docx]
