# Supplementary material for: Evaluation of autoantibody signatures in meningioma patients using human proteome arrays
Source: Oncotarget. 2017 Apr 10;8(35):58443–56. doi: 10.18632/oncotarget.16997 (PMC5601665; doi:10.18632/oncotarget.16997)
Supplement: Supplementary file 8 [file oncotarget-08-58443-s008.docx]

**Supplementary Table 1.1**: Experimental details. The details of the healthy control and Grade I Meningioma samples.

| **Sample ID** | **Sample Type** | **File name** | **Age (years)** | **Sex** |
| --- | --- | --- | --- | --- |
| H-02 | Control | Control_H-02_2000153953.gpr | 23 | M |
| H-03 | Control | Control_H-03_2000153943.gpr | 27 | M |
| H-19 | Control | Control_H-19_2000154008.gpr | 18 | F |
| H-23 | Control | Control_H-23_2000154009.gpr | 20 | F |
| H-25 | Control | Control_H-25_2000153952.gpr | 19 | F |
| H-35 | Control | Control_H-35_2000153942.gpr | 20 | F |
| H-41 | Control | Control_H-41_2000154026.gpr | 19 | F |
| H-58 | Control | Control_H-58_2000153935.gpr | NA | NA |
| H-59 | Control | Control_H-59_2000154016.gpr | NA | NA |
| HC-25 | Control | Control_HC-25_2000153932.gpr | NA | NA |
| HV-56 | Control | Control_HV-56_2000155735.gpr | 35 | M |
| HV-59 | Control | Control_HV-59_2000155740.gpr | 42 | M |
| HV-64 | Control | Control_HV-64_2000144456.gpr | 31 | M |
| HV-70 | Control | Control_HV-70_2000144457.gpr | NA | NA |
| HV-71 | Control | Control_HV-71_2000144458.gpr | NA | NA |
| CF 4450 | MGGrade1 | MG_Grade_I_CF 4450_2000153801.gpr | 55 | F |
| CH 24953 | MGGrade1 | MG_Grade_I_CH 24953_2000153787.gpr | 41 | F |
| CJ 4231 | MGGrade1 | MG_Grade_I_CJ 4231_2000153790.gpr | 35 | F |
| CJ 9179 | MGGrade1 | MG_Grade_I_CJ 9179_2000153906.gpr | 57 | F |
| CJ 14742 | MGGrade1 | MG_Grade_I_CJ 14742_2000153789.gpr | 29 | M |
| CJ 20619 | MGGrade1 | MG_Grade_I_CJ 20619_2000153788.gpr | 41 | F |
| CJ 26538 | MGGrade1 | MG_Grade_I_CJ 26538_2000153915.gpr | 45 | F |
| CJ 29452 | MGGrade1 | MG_Grade_I_CJ 29452_2000153786.gpr | 42 | F |
| CJ 29583 | MGGrade1 | MG_Grade_I_CJ 29583_2000153908.gpr | 44 | F |
| CJ 29822 | MGGrade1 | MG_Grade_I_CJ 29822_2000153909.gpr | 50 | F |

'NA' indicates non-availability of age and sex details; 'M': Indicates Male; 'F' indicates Female.

Shortlisted protein abs Log FC >0.5

**1.2: Shortlisted proteins. List of proteins with p-value<0.05 and logFC >0.5 or <-0.5 (MG1vsHC)**

|  | | | | | | |  |  |  |  |  |
| --- | --- | --- | --- | --- | --- | --- | --- | --- | --- | --- | --- |
| **Block** | **Row** | **Column** | **ID** | **Name** | **logFC** | **AveExpr** | **t** | **P.Value** | **adj.P.Val** | **B** | **abs log FC** |
| 41 | 5 | 11 | BC025985.1 | IGHG4 | -3.15 | 8.68 | -30.04 | 8.02E-38 | 1.45E-33 | 68.66 | 3.149 |
| 10 | 18 | 29 | NM_001014444.1 | CRYM | -1.44 | 7.79 | -22.59 | 5.31E-31 | 4.80E-27 | 56.47 | 1.444 |
| 1 | 14 | 17 | NM_171830.1 | KCNMB3 | -0.72 | 7.53 | -10.77 | 1.24E-15 | 7.46E-12 | 24.94 | 0.716 |
| 3 | 4 | 19 | NM_019102.2 | HOXA5 | -0.83 | 7.62 | -9.14 | 5.81E-13 | 2.62E-09 | 19.13 | 0.829 |
| 3 | 3 | 21 | NM_032328.1 | EFCAB2 | 1.10 | 9.67 | 9.08 | 7.41E-13 | 2.68E-09 | 18.90 | 1.097 |
| 38 | 18 | 5 | NM_031304.2 | DOHH | -1.01 | 10.06 | -8.89 | 1.51E-12 | 4.55E-09 | 18.22 | 1.010 |
| 11 | 3 | 21 | BC006296.2 | ND | 0.60 | 7.53 | 8.45 | 8.45E-12 | 2.18E-08 | 16.58 | 0.601 |
| 14 | 20 | 19 | NM_015726.2 | WDR42A | -1.31 | 8.72 | -8.28 | 1.62E-11 | 3.66E-08 | 15.96 | 1.306 |
| 43 | 1 | 27 | NM_006857.1 | RY1 | 0.88 | 9.31 | 8.22 | 2.11E-11 | 4.23E-08 | 15.71 | 0.880 |
| 23 | 15 | 29 | NM_003153.3 | STAT6 | -0.63 | 7.38 | -7.93 | 6.59E-11 | 1.19E-07 | 14.63 | 0.627 |
| 43 | 2 | 3 | NM_003874.1 | CD84 | 0.50 | 7.44 | 7.86 | 8.66E-11 | 1.42E-07 | 14.37 | 0.500 |
| 3 | 3 | 11 | NM_001001394.2 | HCG3 | 0.90 | 9.07 | 7.80 | 1.09E-10 | 1.46E-07 | 14.15 | 0.901 |
| 38 | 20 | 13 | BC068569.1 | ZHX3 | -0.96 | 9.23 | -7.78 | 1.18E-10 | 1.46E-07 | 14.08 | 0.957 |
| 38 | 1 | 19 | NM_182476.1 | COQ6 | 0.62 | 7.63 | 7.73 | 1.43E-10 | 1.52E-07 | 13.89 | 0.616 |
| 27 | 13 | 15 | BC065370.1 | C20orf112 | -1.83 | 9.13 | -7.66 | 1.86E-10 | 1.87E-07 | 13.64 | 1.831 |
| 27 | 3 | 21 | BC006453.1 | HDAC7A | 1.00 | 8.08 | 7.62 | 2.23E-10 | 2.12E-07 | 13.46 | 1.005 |
| 11 | 1 | 19 | NM_031966.2 | CCNB1 | 0.92 | 8.99 | 7.56 | 2.77E-10 | 2.40E-07 | 13.26 | 0.917 |
| 27 | 22 | 1 | BC073856.1 | ADRB2 | -0.58 | 7.43 | -7.55 | 2.90E-10 | 2.40E-07 | 13.21 | 0.584 |
| 6 | 7 | 21 | BC033854.1 | ND | 0.81 | 8.87 | 7.50 | 3.47E-10 | 2.73E-07 | 13.04 | 0.810 |
| 27 | 21 | 1 | BC037876.1 | C17orf57 | -1.55 | 8.24 | -7.42 | 4.76E-10 | 3.58E-07 | 12.74 | 1.553 |
| 36 | 3 | 13 | NM_004147.3 | DRG1 | 0.55 | 7.65 | 7.35 | 6.39E-10 | 4.61E-07 | 12.46 | 0.548 |
| 16 | 2 | 1 | NM_007198.2 | PROSC | 0.81 | 8.52 | 7.28 | 8.23E-10 | 5.51E-07 | 12.22 | 0.814 |
| 24 | 1 | 7 | NM_007236.3 | CHP | 0.67 | 7.75 | 7.28 | 8.32E-10 | 5.51E-07 | 12.21 | 0.668 |
| 27 | 22 | 17 | NM_001033515.1 | LOC389833 | -1.14 | 8.32 | -7.27 | 8.70E-10 | 5.51E-07 | 12.16 | 1.139 |
| 36 | 1 | 17 | NM_014814.1 | ND | 0.55 | 7.82 | 7.27 | 8.84E-10 | 5.51E-07 | 12.15 | 0.555 |
| 38 | 18 | 17 | NM_012266.3 | DNAJB5 | -0.64 | 8.19 | -7.23 | 1.01E-09 | 5.87E-07 | 12.02 | 0.639 |
| 27 | 1 | 27 | BC017864.1 | GYPE | 0.83 | 7.96 | 7.22 | 1.07E-09 | 6.03E-07 | 11.97 | 0.825 |
| 26 | 13 | 9 | NM_007280.1 | OIP5 | 0.76 | 8.83 | 7.12 | 1.59E-09 | 8.10E-07 | 11.59 | 0.758 |
| 31 | 1 | 25 | BC020733.1 | SRPX2 | 0.68 | 7.46 | 7.11 | 1.61E-09 | 8.10E-07 | 11.57 | 0.678 |
| 16 | 3 | 15 | BC027881.1 | AFP | 0.56 | 7.81 | 7.08 | 1.84E-09 | 8.77E-07 | 11.45 | 0.559 |
| 38 | 24 | 23 | BC031231.1 | STK33 | -0.54 | 7.76 | -6.98 | 2.74E-09 | 1.22E-06 | 11.07 | 0.544 |
| 11 | 24 | 19 | BC029498.1 | N.D. | -0.73 | 8.00 | -6.98 | 2.76E-09 | 1.22E-06 | 11.06 | 0.730 |
| 7 | 3 | 11 | XM_001134297.1 | PRIM2A | 0.55 | 7.47 | 6.96 | 2.99E-09 | 1.29E-06 | 10.99 | 0.554 |
| 36 | 3 | 5 | NM_201555.1 | FHL2 | 0.54 | 8.28 | 6.89 | 3.85E-09 | 1.58E-06 | 10.74 | 0.539 |
| 38 | 21 | 1 | BC066896.1 | KARCA1 | -0.64 | 7.97 | -6.87 | 4.14E-09 | 1.66E-06 | 10.68 | 0.641 |
| 27 | 3 | 25 | NM_006163.1 | NFE2 | 0.69 | 7.44 | 6.86 | 4.33E-09 | 1.66E-06 | 10.63 | 0.692 |
| 11 | 1 | 9 | NM_002867.2 | RAB3B | 0.86 | 9.26 | 6.84 | 4.63E-09 | 1.71E-06 | 10.57 | 0.857 |
| 26 | 3 | 23 | BC008369.1 | STAU2 | 0.75 | 7.70 | 6.79 | 5.75E-09 | 2.01E-06 | 10.36 | 0.748 |
| 27 | 1 | 25 | NM_015971.2 | MRPS7 | 0.69 | 7.94 | 6.78 | 5.89E-09 | 2.01E-06 | 10.34 | 0.692 |
| 8 | 3 | 27 | NM_014764.2 | DAZAP2 | 0.58 | 7.57 | 6.75 | 6.62E-09 | 2.17E-06 | 10.23 | 0.576 |
| 42 | 19 | 25 | BC039725.1 | NA | -0.54 | 8.34 | -6.74 | 7.00E-09 | 2.26E-06 | 10.17 | 0.540 |
| 15 | 3 | 27 | BC004130.2 | CALCOCO2 | 0.71 | 9.36 | 6.70 | 8.32E-09 | 2.64E-06 | 10.01 | 0.709 |
| 3 | 13 | 9 | NM_013409.1 | FST | 0.62 | 9.64 | 6.69 | 8.62E-09 | 2.66E-06 | 9.98 | 0.621 |
| 11 | 1 | 21 | NM_002524.2 | NRAS | 0.64 | 7.54 | 6.68 | 8.70E-09 | 2.66E-06 | 9.97 | 0.642 |
| 20 | 1 | 15 | NM_003332.2 | TYROBP | 0.56 | 7.40 | 6.66 | 9.73E-09 | 2.84E-06 | 9.86 | 0.562 |
| 7 | 20 | 25 | BC071918.1 | RPL10 | -0.70 | 8.57 | -6.62 | 1.10E-08 | 3.13E-06 | 9.75 | 0.699 |
| 38 | 19 | 9 | NM_002257.2 | KLK1 | -0.83 | 8.27 | -6.62 | 1.11E-08 | 3.13E-06 | 9.73 | 0.833 |
| 29 | 6 | 31 | NM_004264.2 | SURB7 | -0.92 | 7.64 | -6.61 | 1.17E-08 | 3.20E-06 | 9.68 | 0.925 |
| 38 | 20 | 27 | NM_025203.1 | C2orf44 | -0.68 | 8.10 | -6.57 | 1.34E-08 | 3.63E-06 | 9.55 | 0.681 |
| 22 | 3 | 5 | BC026107.2 | KRR1 | 0.62 | 8.58 | 6.55 | 1.48E-08 | 3.89E-06 | 9.46 | 0.624 |
| 11 | 1 | 25 | NM_004078.1 | CSRP1 | 0.67 | 8.04 | 6.54 | 1.54E-08 | 3.96E-06 | 9.42 | 0.671 |
| 7 | 2 | 21 | BC010136.2 | P15RS | 0.52 | 7.48 | 6.46 | 2.05E-08 | 4.91E-06 | 9.15 | 0.525 |
| 15 | 1 | 19 | NM_005702.2 | ERAL1 | 0.58 | 7.47 | 6.46 | 2.06E-08 | 4.91E-06 | 9.15 | 0.579 |
| 35 | 20 | 15 | NM_017671.3 | C20orf42 | -0.56 | 7.98 | -6.46 | 2.06E-08 | 4.91E-06 | 9.14 | 0.561 |
| 8 | 3 | 25 | NM_015464.2 | ND | 0.52 | 7.92 | 6.39 | 2.75E-08 | 6.13E-06 | 8.87 | 0.524 |
| 25 | 21 | 21 | BC098119.1 | OGFOD2 | -0.54 | 7.48 | -6.37 | 2.98E-08 | 6.56E-06 | 8.79 | 0.535 |
| 23 | 1 | 17 | NM_017614.3 | BHMT2 | 0.83 | 8.11 | 6.35 | 3.25E-08 | 7.06E-06 | 8.71 | 0.833 |
| 14 | 1 | 17 | NM_022107.1 | GPSM3 | 0.76 | 8.71 | 6.34 | 3.28E-08 | 7.06E-06 | 8.70 | 0.761 |
| 7 | 2 | 19 | NM_138778.1 | ND | 0.57 | 7.41 | 6.31 | 3.76E-08 | 7.64E-06 | 8.57 | 0.572 |
| 11 | 3 | 15 | NM_144634.2 | ND | 0.51 | 7.83 | 6.30 | 3.89E-08 | 7.72E-06 | 8.54 | 0.510 |
| 38 | 17 | 29 | BC003557.1 | APOE | -0.54 | 8.04 | -6.30 | 3.91E-08 | 7.72E-06 | 8.53 | 0.542 |
| 12 | 3 | 1 | NM_002125.3 | HLA-DRB5 | 0.82 | 7.78 | 6.29 | 3.96E-08 | 7.72E-06 | 8.52 | 0.818 |
| 10 | 21 | 5 | BC062437.1 | COX4I1 | -0.51 | 7.63 | -6.29 | 4.08E-08 | 7.75E-06 | 8.49 | 0.512 |
| 15 | 1 | 13 | NM_002149.2 | HPCAL1 | 0.87 | 9.11 | 6.28 | 4.23E-08 | 7.85E-06 | 8.46 | 0.865 |
| 18 | 3 | 13 | NM_198793.2 | CD47 | 0.53 | 7.83 | 6.27 | 4.31E-08 | 7.86E-06 | 8.44 | 0.532 |
| 18 | 3 | 17 | NM_024770.1 | METTL8 | 0.58 | 8.05 | 6.25 | 4.78E-08 | 8.54E-06 | 8.34 | 0.584 |
| 38 | 24 | 25 | NM_173355.2 | UPP2 | -0.57 | 7.89 | -6.24 | 4.95E-08 | 8.77E-06 | 8.31 | 0.567 |
| 7 | 3 | 13 | NM_153207.3 | AEBP2 | 0.50 | 7.44 | 6.23 | 5.07E-08 | 8.89E-06 | 8.28 | 0.502 |
| 11 | 2 | 1 | BC010405.2 | TSPAN17 | 0.57 | 8.35 | 6.23 | 5.15E-08 | 8.95E-06 | 8.27 | 0.575 |
| 38 | 18 | 7 | NM_021979.2 | HSPA2 | -0.70 | 9.30 | -6.21 | 5.46E-08 | 9.30E-06 | 8.21 | 0.703 |
| 5 | 3 | 27 | BC001103.2 | GULP1 | 0.63 | 9.15 | 6.20 | 5.61E-08 | 9.47E-06 | 8.19 | 0.629 |
| 38 | 20 | 15 | NM_014220.2 | TM4SF1 | -0.60 | 7.92 | -6.17 | 6.52E-08 | 1.09E-05 | 8.04 | 0.595 |
| 10 | 2 | 19 | BC015045.2 | GALT | 0.71 | 7.69 | 6.11 | 7.94E-08 | 1.29E-05 | 7.86 | 0.711 |
| 19 | 1 | 19 | BC019059.2 | SP110 | 0.57 | 8.09 | 6.11 | 8.23E-08 | 1.32E-05 | 7.82 | 0.572 |
| 8 | 3 | 13 | NM_015485.3 | RWDD3 | 0.54 | 7.53 | 6.10 | 8.46E-08 | 1.33891E-05 | 7.80 | 0.542 |
| 20 | 1 | 31 | NM_080651.1 | THRAP6 | 0.52 | 7.46 | 6.08 | 8.97E-08 | 1.40E-05 | 7.74 | 0.517 |
| 27 | 3 | 23 | NM_022821.2 | ELOVL1 | 0.62 | 7.44 | 6.08 | 9.23E-08 | 1.41365E-05 | 7.71 | 0.617 |
| 38 | 19 | 15 | NM_000114.2 | EDN3 | -0.59 | 9.17 | -6.06 | 9.65E-08 | 1.47E-05 | 7.67 | 0.586 |
| 15 | 1 | 15 | BC001387.2 | HRASLS3 | 0.51 | 7.92 | 6.06 | 9.99E-08 | 1.50E-05 | 7.64 | 0.513 |
| 7 | 1 | 19 | NM_144586.5 | LYPD1 | 0.62 | 7.57 | 6.05 | 1.01E-07 | 1.50E-05 | 7.62 | 0.617 |
| 38 | 3 | 5 | NM_014078.4 | ND | 0.68 | 7.78 | 6.04 | 1.07E-07 | 1.56E-05 | 7.57 | 0.681 |
| 27 | 12 | 25 | NM_001005465.1 | ND | -1.44 | 10.01 | -6.04 | 1.08E-07 | 1.56E-05 | 7.56 | 1.445 |
| 27 | 3 | 9 | NM_207119.1 | LRRC20 | 0.55 | 7.37 | 6.01 | 1.18E-07 | 1.67E-05 | 7.48 | 0.548 |
| 39 | 6 | 27 | NM_001042476.1 | CARHSP1 | -0.81 | 8.05 | -6.01 | 1.18E-07 | 1.67E-05 | 7.48 | 0.812 |
| 35 | 1 | 31 | BC017842.1 | NAPSA | 0.53 | 7.54 | 6.01 | 1.19E-07 | 1.67E-05 | 7.47 | 0.526 |
| 38 | 18 | 13 | BC008368.1 | C12orf11 | -0.54 | 8.00 | -6.00 | 1.22E-07 | 1.70E-05 | 7.44 | 0.542 |
| 12 | 1 | 15 | NM_001827.1 | CKS2 | 0.87 | 8.34 | 6.00 | 1.25E-07 | 1.72E-05 | 7.42 | 0.872 |
| 6 | 3 | 19 | BC007874.2 | FUBP3 | 0.52 | 7.95 | 5.99 | 1.30E-07 | 1.78E-05 | 7.39 | 0.517 |
| 11 | 3 | 5 | BC022362 | IGKC | 0.51 | 7.68 | 5.97 | 1.41E-07 | 1.90E-05 | 7.31 | 0.512 |
| 38 | 1 | 21 | NM_020070.2 | IGLL1 | 0.57 | 7.43 | 5.95 | 1.50E-07 | 1.99E-05 | 7.25 | 0.567 |
| 16 | 2 | 3 | BC010915.1 | GSTP1 | 0.51 | 8.81 | 5.91 | 1.73E-07 | 2.24E-05 | 7.11 | 0.507 |
| 14 | 1 | 7 | BC001028.1 | MRS2L | 0.57 | 7.40 | 5.89 | 1.91E-07 | 2.36E-05 | 7.02 | 0.573 |
| 7 | 1 | 21 | BC020630.1 | CAMK2N1 | 0.65 | 7.52 | 5.88 | 1.96E-07 | 2.41E-05 | 7.00 | 0.652 |
| 8 | 1 | 7 | NM_021135.4 | RPS6KA2 | 0.76 | 7.51 | 5.87 | 2.03E-07 | 2.46E-05 | 6.96 | 0.756 |
| 11 | 1 | 31 | NM_000397.2 | CYBB | 0.56 | 7.50 | 5.84 | 2.29E-07 | 2.72277E-05 | 6.85 | 0.563 |
| 25 | 23 | 17 | BC110374 | CORO1A | -0.71 | 7.66 | -5.83 | 2.36E-07 | 2.78E-05 | 6.82 | 0.708 |
| 15 | 2 | 19 | BC017247.2 | FUBP1 | 0.50 | 7.80 | 5.83 | 2.37E-07 | 2.78E-05 | 6.81 | 0.501 |
| 16 | 3 | 5 | BC019909.1 | ATAD2 | 0.53 | 7.93 | 5.81 | 2.53E-07 | 2.93E-05 | 6.75 | 0.531 |
| 26 | 19 | 17 | NM_018379.3 | FAM63A | -0.89 | 8.62 | -5.79 | 2.81E-07 | 3.17E-05 | 6.65 | 0.891 |
| 1 | 3 | 31 | BC007520.1 | CAPRIN2 | 0.67 | 8.05 | 5.79 | 2.81E-07 | 3.17E-05 | 6.65 | 0.672 |
| 4 | 3 | 13 | BC012748.1 | FBXO31 | 0.53 | 8.71 | 5.78 | 2.83E-07 | 3.18E-05 | 6.64 | 0.533 |
| 11 | 3 | 1 | NM_138819.1 | FAM122C | 0.51 | 7.96 | 5.75 | 3.27E-07 | 3.52E-05 | 6.51 | 0.512 |
| 19 | 1 | 17 | NM_001011.3 | RPS7 | 0.64 | 8.24 | 5.73 | 3.46E-07 | 3.67E-05 | 6.45 | 0.641 |
| 16 | 3 | 25 | BC007097.1 | TIMP1 | 0.54 | 8.04 | 5.71 | 3.71E-07 | 3.90E-05 | 6.39 | 0.540 |
| 19 | 9 | 19 | ENST00000435033 | NA | 0.54 | 9.27 | 5.71 | 3.74E-07 | 3.91E-05 | 6.38 | 0.540 |
| 1 | 23 | 1 | NM_003154.2 | STATH | -0.55 | 7.85 | -5.69 | 3.97E-07 | 4.09838E-05 | 6.32 | 0.549 |
| 15 | 1 | 29 | NM_002543.2 | OLR1 | 0.51 | 7.54 | 5.68 | 4.23E-07 | 4.29E-05 | 6.26 | 0.511 |
| 3 | 3 | 1 | NM_002801.2 | ND | 0.52 | 7.51 | 5.67 | 4.31E-07 | 4.35E-05 | 6.24 | 0.518 |
| 11 | 3 | 29 | NM_032344.1 | NUDT22 | 0.53 | 7.52 | 5.67 | 4.42E-07 | 4.44E-05 | 6.22 | 0.533 |
| 25 | 23 | 23 | BC035199 | NA | -0.55 | 7.80 | -5.66 | 4.45E-07 | 4.44E-05 | 6.21 | 0.549 |
| 24 | 1 | 15 | NM_021131.3 | PPP2R4 | 0.69 | 8.19 | 5.66 | 4.55E-07 | 4.49E-05 | 6.19 | 0.687 |
| 13 | 22 | 9 | BC064383.1 | SLC39A9 | -0.52 | 7.68 | -5.65 | 4.75E-07 | 4.61E-05 | 6.15 | 0.523 |
| 30 | 20 | 9 | BC037963.1 | ADRBK1 | -0.53 | 8.03 | -5.63 | 5.14E-07 | 4.82E-05 | 6.08 | 0.533 |
| 38 | 18 | 15 | BC000576.2 | QDPR | -0.52 | 8.77 | -5.59 | 5.84E-07 | 5.37E-05 | 5.95 | 0.520 |
| 39 | 12 | 19 | NM_199294.1 | APITD1 | -0.59 | 10.57 | -5.59 | 5.91E-07 | 5.39E-05 | 5.94 | 0.589 |
| 5 | 1 | 15 | NM_001549.2 | IFIT3 | 0.74 | 9.13 | 5.58 | 6.01E-07 | 5.46E-05 | 5.93 | 0.739 |
| 20 | 2 | 7 | BC014607.2 | ROPN1L | 0.58 | 8.00 | 5.58 | 6.21E-07 | 5.54E-05 | 5.90 | 0.584 |
| 11 | 1 | 13 | BC001360.2 | RHOA | 0.69 | 8.88 | 5.55 | 6.79E-07 | 5.89E-05 | 5.81 | 0.691 |
| 27 | 1 | 19 | BC020749.1 | CD96 | 0.53 | 7.39 | 5.55 | 6.83E-07 | 5.90E-05 | 5.81 | 0.528 |
| 26 | 23 | 13 | BC025407.1 | LAYN | -0.70 | 9.36 | -5.55 | 6.89E-07 | 5.90E-05 | 5.80 | 0.704 |
| 12 | 1 | 19 | NM_138390.2 | ND | 0.50 | 7.78 | 5.55 | 6.90E-07 | 5.90E-05 | 5.80 | 0.501 |
| 26 | 23 | 17 | Dlx5 | Dlx5 | -0.63 | 7.83 | -5.54 | 7.11E-07 | 6.06E-05 | 5.77 | 0.632 |
| 26 | 3 | 1 | BC011757.2 | GADD45A | 0.55 | 8.70 | 5.54 | 7.14E-07 | 6.06E-05 | 5.76 | 0.548 |
| 38 | 21 | 31 | BC098334.1 | RAP1GDS1 | -0.76 | 8.87 | -5.53 | 7.28E-07 | 6.11E-05 | 5.75 | 0.763 |
| 4 | 3 | 5 | NM_022488.3 | ATG3 | 0.65 | 7.82 | 5.53 | 7.33E-07 | 6.12654E-05 | 5.74 | 0.646 |
| 15 | 1 | 21 | BC020691.1 | PBEF1 | 0.53 | 7.43 | 5.53 | 7.46E-07 | 6.21E-05 | 5.72 | 0.533 |
| 20 | 16 | 3 | ENST00000362035 | NA | 0.54 | 8.39 | 5.51 | 8.00E-07 | 6.51E-05 | 5.66 | 0.538 |
| 20 | 10 | 3 | BC000870.1 | TIPIN | 0.72 | 8.99 | 5.51 | 8.02E-07 | 6.51E-05 | 5.65 | 0.716 |
| 20 | 1 | 27 | BC008253.1 | C8orf43 | 0.74 | 8.39 | 5.51 | 8.04E-07 | 6.51E-05 | 5.65 | 0.741 |
| 12 | 3 | 23 | BC005395.1 | HPX | 0.51 | 7.35 | 5.49 | 8.72E-07 | 6.91E-05 | 5.57 | 0.505 |
| 8 | 1 | 9 | NM_170693.1 | SGK2 | 0.66 | 8.50 | 5.47 | 9.16E-07 | 7.13E-05 | 5.53 | 0.660 |
| 4 | 3 | 15 | NM_018090.3 | NECAP2 | 0.55 | 8.84 | 5.45 | 9.83E-07 | 7.45894E-05 | 5.46 | 0.545 |
| 4 | 1 | 17 | NM_000854.2 | ND | 0.65 | 7.55 | 5.44 | 1.03E-06 | 7.71E-05 | 5.42 | 0.648 |
| 7 | 1 | 27 | BC019064.2 | FAM40B | 0.63 | 7.52 | 5.43 | 1.08E-06 | 7.91E-05 | 5.37 | 0.627 |
| 15 | 4 | 3 | NM_024096.1 | XTP3TPA | 0.54 | 9.02 | 5.43 | 1.08E-06 | 7.91E-05 | 5.37 | 0.541 |
| 25 | 3 | 21 | NM_002120.2 | HLA-DOB | 0.64 | 7.50 | 5.42 | 1.14E-06 | 8.23E-05 | 5.32 | 0.639 |
| 30 | 2 | 25 | NM_001025436.1 | SPAG16 | 0.77 | 8.51 | 5.41 | 1.15E-06 | 8.28536E-05 | 5.31 | 0.767 |
| 44 | 24 | 1 | BC038596 | CHD4 | -0.69 | 9.51 | -5.41 | 1.17E-06 | 8.37E-05 | 5.29 | 0.690 |
| 6 | 3 | 23 | NM_013443.3 | ST6GALNAC6 | 0.57 | 7.54 | 5.41 | 1.18E-06 | 8.41E-05 | 5.28 | 0.569 |
| 4 | 1 | 23 | NM_001007226.1 | ND | 0.59 | 7.99 | 5.38 | 1.32E-06 | 9.09E-05 | 5.18 | 0.588 |
| 11 | 1 | 27 | NM_001381.2 | DOK1 | 0.56 | 9.10 | 5.37 | 1.36E-06 | 9.23E-05 | 5.15 | 0.564 |
| 3 | 1 | 25 | NM_022497.3 | MRPS25 | 0.54 | 7.58 | 5.36 | 1.38E-06 | 9.30E-05 | 5.14 | 0.539 |
| 31 | 24 | 9 | NM_005805.2 | PSMD14 | -0.62 | 7.91 | -5.35 | 1.46E-06 | 9.74E-05 | 5.09 | 0.624 |
| 7 | 3 | 5 | BC015631.2 | SLC5A6 | 0.52 | 7.45 | 5.33 | 1.57E-06 | 0.000102505 | 5.01 | 0.523 |
| 11 | 13 | 9 | NM_000884.2 | IMPDH2 | 0.63 | 8.80 | 5.32 | 1.61E-06 | 0.000104404 | 4.99 | 0.627 |
| 15 | 1 | 25 | NM_002059.3 | GH2 | 0.52 | 7.40 | 5.32 | 1.62E-06 | 0.000104404 | 4.99 | 0.518 |
| 7 | 3 | 3 | BC026296.1 | PANK1 | 0.50 | 7.49 | 5.32 | 1.63E-06 | 0.000104713 | 4.98 | 0.503 |
| 12 | 3 | 15 | BC009785.1 | GLYAT | 0.51 | 7.33 | 5.31 | 1.69E-06 | 0.00010698 | 4.94 | 0.511 |
| 25 | 18 | 25 | NM_004736.2 | XPR1 | -0.53 | 8.07 | -5.29 | 1.82E-06 | 0.000112883 | 4.87 | 0.528 |
| 27 | 1 | 21 | NM_030588.1 | DHX9 | 0.61 | 7.44 | 5.29 | 1.83E-06 | 0.000112883 | 4.87 | 0.608 |
| 12 | 1 | 9 | NM_006098.4 | GNB2L1 | 0.73 | 7.95 | 5.29 | 1.85E-06 | 0.000113041 | 4.86 | 0.726 |
| 3 | 2 | 21 | NM_001012505.1 | ND | 0.51 | 7.61 | 5.28 | 1.87E-06 | 0.000113303 | 4.85 | 0.512 |
| 34 | 24 | 23 | NM_016183.2 | MRTO4 | -0.58 | 8.35 | -5.28 | 1.92171E-06 | 0.000114953 | 4.82 | 0.575 |
| 38 | 20 | 25 | NM_001801.2 | CDO1 | -0.78 | 9.27 | -5.27 | 1.93E-06 | 0.000115345 | 4.82 | 0.776 |
| 12 | 3 | 19 | NM_005872.2 | BCAS2 | 0.54 | 7.33 | 5.27 | 1.99E-06 | 0.000117314 | 4.79 | 0.535 |
| 47 | 20 | 21 | BC007232.1 | FAM58A | -0.58 | 8.32 | -5.26 | 2.07E-06 | 0.000120726 | 4.75 | 0.581 |
| 8 | 1 | 31 | NM_203371.1 | LOC387758 | 0.50 | 7.46 | 5.25 | 2.07E-06 | 0.000120726 | 4.75 | 0.503 |
| 27 | 2 | 21 | NM_016243.2 | CYB5R1 | 0.58 | 7.35 | 5.25 | 2.14E-06 | 0.000123619 | 4.72 | 0.580 |
| 11 | 1 | 29 | NM_002009.2 | FGF7 | 0.52 | 7.83 | 5.24 | 2.21E-06 | 0.000126911 | 4.69 | 0.519 |
| 8 | 1 | 27 | ENST00000331450 | NA | 0.54 | 7.75 | 5.24 | 2.21E-06 | 0.000126911 | 4.69 | 0.543 |
| 25 | 1 | 21 | NM_004359.1 | CDC34 | 0.68 | 8.46 | 5.24 | 2.22E-06 | 0.000126911 | 4.68 | 0.676 |
| 3 | 2 | 1 | NM_032181.1 | TMEM166 | 0.53 | 7.61 | 5.21 | 2.49E-06 | 0.000138983 | 4.57 | 0.533 |
| 27 | 1 | 17 | NM_001305.3 | CLDN4 | 0.65 | 7.42 | 5.16 | 2.90E-06 | 0.000155147 | 4.43 | 0.646 |
| 1 | 1 | 21 | NM_012106.3 | ARL2BP | 0.86 | 8.81 | 5.16 | 2.92E-06 | 0.000155147 | 4.42 | 0.857 |
| 7 | 1 | 17 | ENST00000368135 | NA | 0.57 | 7.49 | 5.16 | 2.99E-06 | 0.00015762 | 4.40 | 0.575 |
| 23 | 2 | 11 | BC016854.2 | C11orf67 | 0.52 | 8.89 | 5.15 | 3.01E-06 | 0.000157861 | 4.40 | 0.517 |
| 14 | 1 | 23 | NM_144642.3 | SYNPR | 0.52 | 7.45 | 5.15 | 3.03E-06 | 0.000158514 | 4.39 | 0.519 |
| 35 | 21 | 23 | NM_001010977.1 | LOC196541 | -0.66 | 9.45 | -5.15 | 3.05E-06 | 0.000158855 | 4.38 | 0.660 |
| 10 | 2 | 29 | NM_171998.2 | RAB39B | 0.57 | 8.24 | 5.14 | 3.15E-06 | 0.000162237 | 4.35 | 0.573 |
| 31 | 2 | 1 | NM_000229.1 | LCAT | 0.52 | 7.35 | 5.14 | 3.16E-06 | 0.000162237 | 4.35 | 0.521 |
| 38 | 20 | 11 | NM_003385.4 | VSNL1 | -0.71 | 10.62 | -5.13 | 3.32E-06 | 0.000167048 | 4.30 | 0.708 |
| 38 | 24 | 19 | BC029896.1 | N.D. | -0.74 | 8.70 | -5.13 | 3.33E-06 | 0.000167048 | 4.30 | 0.739 |
| 27 | 1 | 7 | BC002862.2 | ND | 0.61 | 7.39 | 5.12 | 3.37E-06 | 0.000168154 | 4.29 | 0.611 |
| 8 | 16 | 11 | NM_139204.1 | EPS8L1 | 1.22 | 8.43 | 5.11 | 3.48E-06 | 0.000170906 | 4.26 | 1.216 |
| 15 | 3 | 21 | NM_016172.2 | UBADC1 | 0.79 | 8.08 | 5.11 | 3.52E-06 | 0.000171238 | 4.25 | 0.792 |
| 6 | 2 | 19 | NM_002825.5 | PTN | 0.59 | 7.52 | 5.11 | 3.53E-06 | 0.000171238 | 4.24 | 0.593 |
| 3 | 8 | 3 | NM_006064.3 | RRAGB | 0.51 | 9.47 | 5.10 | 3.62E-06 | 0.000173903 | 4.22 | 0.506 |
| 47 | 15 | 11 | NM_003798.1 | CTNNAL1 | -0.57 | 10.05 | -5.10 | 3.66E-06 | 0.000174609 | 4.21 | 0.570 |
| 27 | 1 | 23 | ENST00000321023 | NA | 0.52 | 7.36 | 5.10 | 3.66E-06 | 0.000174609 | 4.21 | 0.521 |
| 43 | 22 | 1 | BC071725.1 | IGL@ | -0.52 | 7.53 | -5.10 | 3.70E-06 | 0.000175177 | 4.20 | 0.523 |
| 3 | 1 | 15 | NM_018969.3 | ND | 0.59 | 7.55 | 5.10 | 3.72E-06 | 0.000175405 | 4.20 | 0.589 |
| 12 | 3 | 11 | BC024256.1 | FBXL18 | 0.55 | 7.33 | 5.10 | 3.73E-06 | 0.000175589 | 4.19 | 0.548 |
| 23 | 1 | 13 | BC001550.1 | SFN | 0.64 | 8.29 | 5.09 | 3.84E-06 | 0.000178421 | 4.16 | 0.643 |
| 31 | 6 | 29 | NM_030920.2 | ANP32E | 0.59 | 8.29 | 5.08 | 3.97E-06 | 0.000182373 | 4.13 | 0.588 |
| 34 | 20 | 11 | NM_022158.2 | FN3K | -0.67 | 8.99 | -5.08 | 3.98E-06 | 0.000182373 | 4.13 | 0.669 |
| 12 | 2 | 9 | NM_052838.2 | SEPT1 | 0.69 | 7.96 | 5.06 | 4.30E-06 | 0.000193539 | 4.06 | 0.694 |
| 15 | 1 | 27 | NM_024782.1 | NHEJ1 | 0.56 | 8.14 | 5.05 | 4.44E-06 | 0.000199595 | 4.03 | 0.562 |
| 4 | 3 | 25 | BC014441.1 | ND | 0.51 | 7.47 | 5.05 | 4.47E-06 | 0.000200398 | 4.02 | 0.511 |
| 4 | 1 | 7 | BC000293.2 | NME1 | 0.69 | 7.94 | 5.04 | 4.53E-06 | 0.000201591 | 4.01 | 0.694 |
| 34 | 13 | 21 | NM_006645.2 | STARD10 | -0.51 | 9.29 | -5.03 | 4.67E-06 | 0.000205997 | 3.98 | 0.507 |
| 25 | 1 | 7 | NM_020677.2 | NMRAL1 | 0.67 | 8.10 | 5.02 | 4.84E-06 | 0.000210256 | 3.94 | 0.675 |
| 45 | 1 | 31 | BC013393.2 | ADD1 | 0.58 | 8.84 | 5.02 | 4.95E-06 | 0.000213527 | 3.92 | 0.577 |
| 3 | 1 | 31 | NM_016188.3 | ACTL6B | 0.53 | 7.81 | 5.00 | 5.39E-06 | 0.000227677 | 3.84 | 0.527 |
| 15 | 1 | 11 | NM_016049.3 | C14orf122 | 0.72 | 9.28 | 4.99 | 5.52E-06 | 0.000230409 | 3.82 | 0.719 |
| 35 | 15 | 13 | NM_001025266.1 | LOC285382 | 1.32 | 8.63 | 4.99 | 5.53E-06 | 0.000230409 | 3.82 | 1.316 |
| 6 | 1 | 13 | NM_001744.3 | CAMK4 | 0.57 | 9.04 | 4.99 | 5.54E-06 | 0.000230409 | 3.82 | 0.573 |
| 3 | 1 | 17 | NM_001018.3 | RPS15 | 0.55 | 7.59 | 4.98 | 5.64E-06 | 0.000232688 | 3.80 | 0.545 |
| 23 | 3 | 25 | NM_015926.3 | TEX264 | 0.51 | 8.32 | 4.95 | 6.30E-06 | 0.000251892 | 3.69 | 0.515 |
| 11 | 15 | 1 | NM_000788.1 | DCK | 0.50 | 9.82 | 4.94 | 6.53E-06 | 0.000258341 | 3.66 | 0.504 |
| 14 | 1 | 19 | BC018090.1 | ZNF641 | 0.58 | 7.50 | 4.94 | 6.58E-06 | 0.000258785 | 3.65 | 0.576 |
| 42 | 22 | 25 | BC070280.1 | DHFR | -0.56 | 9.31 | -4.93 | 6.72E-06 | 0.000261752 | 3.63 | 0.556 |
| 20 | 13 | 9 | NM_012175.3 | FBXO3 | 0.54 | 8.64 | 4.91 | 7.37E-06 | 0.000277102 | 3.55 | 0.542 |
| 7 | 1 | 25 | BC022405.1 | TMEM185A | 0.58 | 7.47 | 4.91 | 7.38E-06 | 0.000277102 | 3.55 | 0.578 |
| 35 | 23 | 5 | Q5SZD1 | C6orf141 | -0.68 | 9.64 | -4.91 | 7.44E-06 | 0.00027883 | 3.54 | 0.676 |
| 31 | 1 | 3 | NM_016185.2 | HN1 | 0.67 | 8.03 | 4.89 | 7.98E-06 | 0.000292415 | 3.47 | 0.671 |
| 3 | 1 | 7 | BC000607.1 | ANAPC11 | 0.54 | 7.55 | 4.89 | 8.00E-06 | 0.00029254 | 3.47 | 0.535 |
| 31 | 20 | 5 | BC132786.1 | DDX58 | -0.53 | 7.67 | -4.88 | 8.19E-06 | 0.000297769 | 3.45 | 0.527 |
| 21 | 18 | 15 | NM_005719.2 | ARPC3 | -0.83 | 8.35 | -4.88 | 8.22E-06 | 0.000297769 | 3.44 | 0.827 |
| 34 | 22 | 7 | BC093033.1 | SPP1 | -0.66 | 8.93 | -4.88 | 8.23E-06 | 0.000297769 | 3.44 | 0.658 |
| 27 | 3 | 11 | NM_004362.1 | CLGN | 0.57 | 7.70 | 4.88 | 8.24E-06 | 0.000297769 | 3.44 | 0.575 |
| 38 | 3 | 1 | BC026196.2 | ND | 0.57 | 7.55 | 4.88 | 8.31E-06 | 0.000299725 | 3.43 | 0.569 |
| 47 | 2 | 21 | NM_001031677.2 | RAB24 | 0.55 | 8.99 | 4.88 | 8.35E-06 | 0.000300497 | 3.43 | 0.555 |
| 27 | 21 | 7 | NM_001039656.1 | MTL5 | -0.99 | 7.81 | -4.87 | 8.65E-06 | 0.000309467 | 3.39 | 0.986 |
| 47 | 12 | 27 | BC036107.1 | HSPA2 | -0.71 | 9.86 | -4.86 | 8.93E-06 | 0.000317608 | 3.36 | 0.709 |
| 22 | 3 | 7 | BC027956.1 | SULT1E1 | 0.57 | 8.65 | 4.85 | 9.13E-06 | 0.000322822 | 3.34 | 0.573 |
| 7 | 1 | 29 | NM_053282.4 | SH2D1B | 0.62 | 7.49 | 4.84 | 9.60E-06 | 0.000333013 | 3.30 | 0.617 |
| 27 | 20 | 1 | NM_003597.4 | KLF11 | -0.72 | 7.63 | -4.84 | 9.64E-06 | 0.00033361 | 3.29 | 0.721 |
| 12 | 1 | 21 | BC001023.2 | HLA-DRB3 | 0.72 | 7.49 | 4.83 | 9.86E-06 | 0.000337416 | 3.27 | 0.715 |
| 12 | 3 | 13 | NM_015953.3 | NOSIP | 0.56 | 7.38 | 4.83 | 9.86E-06 | 0.000337416 | 3.27 | 0.560 |
| 34 | 3 | 5 | NM_003732.2 | EIF4EBP3 | 0.55 | 9.51 | 4.83 | 9.92E-06 | 0.000338226 | 3.27 | 0.549 |
| 47 | 19 | 7 | BC018029.1 | PTS | -0.59 | 9.68 | -4.82 | 1.03E-05 | 0.000346944 | 3.23 | 0.587 |
| 24 | 1 | 9 | NM_022128.1 | RBKS | 0.51 | 7.40 | 4.81 | 1.06E-05 | 0.000351169 | 3.21 | 0.514 |
| 12 | 1 | 7 | NM_001239.2 | CCNH | 0.70 | 7.40 | 4.80 | 1.08E-05 | 0.000357766 | 3.18 | 0.697 |
| 6 | 2 | 27 | NM_001007099.1 | ND | 0.52 | 7.54 | 4.80 | 1.11E-05 | 0.000363062 | 3.16 | 0.518 |
| 20 | 1 | 13 | BC002695.2 | AAK1 | 0.58 | 7.99 | 4.80 | 1.11E-05 | 0.000363737 | 3.16 | 0.577 |
| 40 | 19 | 9 | NM_001176.2 | ARHGDIG | -0.50 | 8.78 | -4.79 | 1.12E-05 | 0.000365064 | 3.15 | 0.504 |
| 12 | 1 | 23 | BC008145.1 | APEX1 | 0.61 | 7.40 | 4.76 | 1.29E-05 | 0.000406795 | 3.02 | 0.607 |
| 25 | 22 | 29 | BC071639 | ZNF761 | -0.51 | 7.83 | -4.75 | 1.30E-05 | 0.000408698 | 3.01 | 0.513 |
| 27 | 23 | 25 | BC106934 | CCT4 | -0.53 | 7.44 | -4.75 | 1.30E-05 | 0.000408698 | 3.01 | 0.535 |
| 19 | 1 | 21 | NM_013368.2 | SERTAD3 | 0.83 | 8.72 | 4.75 | 1.30E-05 | 0.000408698 | 3.01 | 0.831 |
| 8 | 1 | 13 | BC021992.1 | ND | 0.58 | 7.44 | 4.75 | 1.33E-05 | 0.000414492 | 2.99 | 0.585 |
| 12 | 3 | 7 | ENST00000302517 | CXXC5 | 0.57 | 7.36 | 4.74 | 1.35E-05 | 0.0004189 | 2.98 | 0.570 |
| 3 | 1 | 13 | BC001956.1 | FNIP1 | 0.52 | 7.53 | 4.74 | 1.36E-05 | 0.000420684 | 2.97 | 0.523 |
| 1 | 2 | 3 | NM_015433.2 | FAM119B | 0.66 | 9.23 | 4.73 | 1.42E-05 | 0.000431861 | 2.93 | 0.663 |
| 6 | 1 | 15 | NM_033313.1 | ND | 0.57 | 7.84 | 4.73 | 1.42E-05 | 0.000433079 | 2.92 | 0.568 |
| 12 | 3 | 9 | NM_173509.2 | C1orf76 | 0.51 | 7.34 | 4.72 | 1.48E-05 | 0.000446551 | 2.89 | 0.508 |
| 12 | 3 | 3 | BC017219.2 | ZNF587 | 0.55 | 7.61 | 4.71 | 1.49E-05 | 0.000446949 | 2.88 | 0.545 |
| 46 | 21 | 27 | BC098132.1 | CCR10 | -0.65 | 8.07 | -4.71 | 1.49E-05 | 0.000446949 | 2.88 | 0.652 |
| 20 | 2 | 1 | NM_053050.2 | MRPL53 | 0.56 | 7.79 | 4.71 | 1.52E-05 | 0.000453595 | 2.86 | 0.556 |
| 25 | 13 | 9 | BC002448.2 | ABLIM1 | 0.57 | 8.38 | 4.70 | 1.54E-05 | 0.000458295 | 2.85 | 0.569 |
| 34 | 1 | 23 | NM_006321.2 | ARIH2 | 0.52 | 8.73 | 4.67 | 1.74E-05 | 0.000501422 | 2.73 | 0.520 |
| 4 | 2 | 1 | BC013418.2 | C13orf3 | 0.64 | 7.57 | 4.67 | 1.77E-05 | 0.000507303 | 2.72 | 0.643 |
| 39 | 10 | 31 | NM_004873.2 | BAG5 | -0.61 | 9.37 | -4.66 | 1.81E-05 | 0.000513671 | 2.70 | 0.614 |
| 36 | 1 | 15 | BC013992.1 | MAPK3 | 0.73 | 9.26 | 4.66 | 1.82E-05 | 0.0005161 | 2.69 | 0.729 |
| 10 | 1 | 23 | NM_022555.3 | HLA-DRB3 | 0.51 | 7.41 | 4.65 | 1.88E-05 | 0.000530628 | 2.66 | 0.513 |
| 27 | 14 | 17 | NM_148910.2 | TIRAP | -1.05 | 8.43 | -4.64 | 1.93E-05 | 0.000537739 | 2.64 | 1.052 |
| 4 | 2 | 5 | BC009510.2 | C9orf123 | 0.54 | 7.54 | 4.63 | 2.04E-05 | 0.000559939 | 2.59 | 0.544 |
| 16 | 1 | 21 | NM_178181.1 | CDCP1 | 0.51 | 7.79 | 4.63 | 2.04E-05 | 0.000561336 | 2.58 | 0.512 |
| 7 | 1 | 9 | NM_006790.1 | MYOT | 0.58 | 7.48 | 4.62 | 2.06E-05 | 0.000564264 | 2.58 | 0.581 |
| 6 | 1 | 25 | NM_001037666.1 | LOC652968 | 0.58 | 8.27 | 4.62 | 2.10E-05 | 0.00057205 | 2.56 | 0.577 |
| 3 | 1 | 29 | NM_001662.2 | ARF5 | 0.52 | 8.07 | 4.61 | 2.19E-05 | 0.000588607 | 2.52 | 0.522 |
| 4 | 1 | 9 | NM_003404.3 | YWHAB | 0.67 | 7.87 | 4.60 | 2.20442E-05 | 0.000590843 | 2.51 | 0.675 |
| 9 | 2 | 25 | NM_006360.3 | PCID1 | 0.72 | 9.90 | 4.60 | 2.22E-05 | 0.000593341 | 2.51 | 0.715 |
| 24 | 12 | 29 | NM_014063.5 | DBNL | -0.56 | 10.53 | -4.60 | 2.25E-05 | 0.000598938 | 2.49 | 0.564 |
| 8 | 2 | 17 | NM_080664.2 | C14orf126 | 0.59 | 7.67 | 4.60 | 2.27E-05 | 0.000601034 | 2.48 | 0.589 |
| 30 | 12 | 15 | NM_001008657.1 | TCOF1 | -0.56 | 8.64 | -4.59 | 2.36E-05 | 0.0006142 | 2.45 | 0.558 |
| 4 | 14 | 25 | BC063111.1 | GGT6 | 0.52 | 8.47 | 4.58 | 2.36815E-05 | 0.0006142 | 2.44 | 0.523 |
| 18 | 1 | 17 | BC000606.2 | RPL14 | 0.52 | 7.38 | 4.58 | 2.37E-05 | 0.0006142 | 2.44 | 0.522 |
| 8 | 1 | 25 | NM_031468.2 | CALN1 | 0.58 | 8.58 | 4.58 | 2.41E-05 | 0.000623352 | 2.43 | 0.581 |
| 10 | 1 | 27 | NM_172369.2 | C1QC | 0.53 | 7.48 | 4.57 | 2.49E-05 | 0.000636554 | 2.39 | 0.530 |
| 4 | 3 | 1 | NM_030806.3 | C1orf21 | 0.61 | 8.32 | 4.57 | 2.53E-05 | 0.000643354 | 2.38 | 0.606 |
| 6 | 11 | 3 | BC014258.1 | IGHG1 | 0.59 | 9.62 | 4.56 | 2.62E-05 | 0.000658931 | 2.35 | 0.590 |
| 12 | 2 | 3 | BC022476.1 | C20orf28 | 0.60 | 7.37 | 4.55 | 2.66E-05 | 0.000663261 | 2.33 | 0.600 |
| 18 | 1 | 27 | BC008685.1 | ND | 0.52 | 7.60 | 4.55 | 2.66E-05 | 0.000663261 | 2.33 | 0.516 |
| 37 | 22 | 1 | BC068458.1 | VIL2 | -0.55 | 8.87 | -4.55 | 2.71E-05 | 0.000670165 | 2.32 | 0.550 |
| 7 | 2 | 1 | BC015416.1 | RNF32 | 0.51 | 7.50 | 4.55 | 2.71E-05 | 0.000670165 | 2.32 | 0.512 |
| 47 | 20 | 11 | NM_003314.1 | TTC1 | -0.76 | 9.84 | -4.54 | 2.75E-05 | 0.000676537 | 2.30 | 0.761 |
| 25 | 1 | 27 | ENST00000312635 | USP15 | 0.84 | 8.42 | 4.53 | 2.87E-05 | 0.000698122 | 2.26 | 0.835 |
| 12 | 3 | 5 | BC009313 | MORC4 | 0.57 | 7.36 | 4.53 | 2.90E-05 | 0.000704651 | 2.25 | 0.573 |
| 27 | 20 | 9 | NM_003359.2 | UGDH | -0.79 | 7.75 | -4.51 | 3.10E-05 | 0.000734574 | 2.19 | 0.794 |
| 14 | 2 | 1 | NM_014034.1 | ASF1A | 0.56 | 8.84 | 4.51 | 3.11E-05 | 0.000735667 | 2.19 | 0.564 |
| 46 | 21 | 9 | BC036124.1 | MIPOL1 | -0.58 | 8.13 | -4.50 | 3.20E-05 | 0.000748676 | 2.16 | 0.579 |
| 20 | 2 | 31 | BC007566.1 | M6PRBP1 | 0.53 | 8.68 | 4.50 | 3.24E-05 | 0.000752754 | 2.15 | 0.528 |
| 4 | 1 | 19 | BC001317.1 | ENOPH1 | 0.51 | 8.06 | 4.49 | 3.31035E-05 | 0.000764724 | 2.13 | 0.508 |
| 24 | 1 | 3 | BC008692.1 | PTPN11 | 0.53 | 7.34 | 4.48 | 3.42E-05 | 0.000786301 | 2.10 | 0.535 |
| 40 | 7 | 13 | BC015738.1 | ZFYVE19 | 0.51 | 8.92 | 4.48 | 3.44E-05 | 0.000786816 | 2.09 | 0.514 |
| 47 | 9 | 9 | NM_004537.3 | ND | -0.72 | 9.86 | -4.47 | 3.53E-05 | 0.000803897 | 2.07 | 0.721 |
| 3 | 19 | 5 | NM_003690.3 | PRKRA | -0.52 | 7.94 | -4.46 | 3.66E-05 | 0.000824874 | 2.03 | 0.522 |
| 39 | 23 | 5 | NM_022743.1 | SMYD3 | -0.66 | 10.45 | -4.46 | 3.71E-05 | 0.000830482 | 2.02 | 0.659 |
| 34 | 23 | 11 | NM_006739.2 | MCM5 | -0.56 | 8.39 | -4.46 | 3.71E-05 | 0.000830482 | 2.02 | 0.559 |
| 31 | 6 | 23 | NM_017924.2 | C14orf119 | 0.54 | 9.20 | 4.45 | 3.74E-05 | 0.000834151 | 2.01 | 0.545 |
| 4 | 3 | 21 | NM_000136.2 | ND | 0.51 | 7.48 | 4.45 | 3.77E-05 | 0.000838582 | 2.00 | 0.508 |
| 39 | 24 | 3 | NM_138930.2 | DIABLO | -0.63 | 10.51 | -4.44 | 3.86822E-05 | 0.000848973 | 1.98 | 0.625 |
| 27 | 2 | 9 | BC015350.1 | QTRT1 | 0.50 | 7.33 | 4.44 | 3.91E-05 | 0.000854843 | 1.97 | 0.504 |
| 12 | 2 | 1 | NM_023070.1 | ZNF643 | 0.66 | 7.40 | 4.43 | 4.02E-05 | 0.000864808 | 1.94 | 0.660 |
| 14 | 3 | 11 | BC027870.1 | PDLIM3 | 0.59 | 9.34 | 4.43 | 4.03E-05 | 0.000865393 | 1.94 | 0.592 |
| 27 | 15 | 13 | NM_002893.2 | RBBP7 | -1.00 | 8.12 | -4.43 | 4.05E-05 | 0.000866432 | 1.94 | 1.004 |
| 12 | 21 | 5 | BC022399.1 | PI16 | -0.53 | 7.80 | -4.42 | 4.19165E-05 | 0.000887715 | 1.90 | 0.529 |
| 25 | 2 | 29 | BC020233 | CKAP2 | 0.53 | 8.23 | 4.42 | 4.26E-05 | 0.000896359 | 1.89 | 0.527 |
| 27 | 1 | 15 | NM_000810.2 | GABRA5 | 0.58 | 7.47 | 4.41 | 4.33E-05 | 0.000908095 | 1.87 | 0.575 |
| 4 | 1 | 13 | NM_017567.2 | NAGK | 0.59 | 8.19 | 4.41 | 4.33E-05 | 0.000908334 | 1.87 | 0.585 |
| 43 | 20 | 31 | BC095488.1 | PRH2 | -0.55 | 7.89 | -4.41 | 4.34E-05 | 0.000909379 | 1.87 | 0.546 |
| 42 | 24 | 31 | NM_130808.1 | CPNE4 | -0.84 | 9.53 | -4.41 | 4.35E-05 | 0.000909632 | 1.87 | 0.839 |
| 47 | 10 | 31 | NM_170676.2 | MEIS2 | -0.56 | 9.23 | -4.40 | 4.57E-05 | 0.000943943 | 1.82 | 0.563 |
| 2 | 1 | 25 | NM_014060.1 | MCTS1 | 0.52 | 7.58 | 4.40 | 4.60E-05 | 0.000948826 | 1.82 | 0.519 |
| 12 | 1 | 25 | NM_024295.3 | DERL1 | 0.56 | 7.37 | 4.39 | 4.64E-05 | 0.000955045 | 1.81 | 0.558 |
| 10 | 1 | 29 | NM_138820.2 | HIGD2A | 0.53 | 7.45 | 4.38 | 4.86E-05 | 0.000993164 | 1.77 | 0.531 |
| 10 | 20 | 9 | NM_000983.3 | RPL22 | -0.52 | 7.78 | -4.38 | 4.89E-05 | 0.000996561 | 1.76 | 0.517 |
| 24 | 2 | 19 | BC003587.1 | C21orf33 | 0.50 | 8.09 | 4.37 | 5.02E-05 | 0.001018968 | 1.74 | 0.501 |
| 40 | 22 | 7 | BC068547.1 | SRPK2 | -0.66 | 8.17 | -4.36 | 5.16E-05 | 0.001036154 | 1.71 | 0.665 |
| 12 | 2 | 7 | NM_152450.1 | FAM81A | 0.58 | 7.38 | 4.36 | 5.27E-05 | 0.001050206 | 1.69 | 0.581 |
| 18 | 1 | 9 | NM_014365.2 | HSPB8 | 0.51 | 7.51 | 4.35 | 5.29E-05 | 0.001052293 | 1.69 | 0.511 |
| 4 | 1 | 31 | NM_080653.3 | ATP6V1E2 | 0.60 | 7.99 | 4.35 | 5.45E-05 | 0.00107654 | 1.66 | 0.604 |
| 28 | 13 | 9 | NM_173608.1 | C14orf80 | 0.57 | 8.50 | 4.34 | 5.66E-05 | 0.001101463 | 1.62 | 0.569 |
| 42 | 3 | 7 | NM_000184.2 | HBG2 | 0.68 | 9.36 | 4.33 | 5.68E-05 | 0.001103072 | 1.62 | 0.677 |
| 24 | 2 | 1 | NM_001003677.1 | C11orf49 | 0.51 | 7.60 | 4.33 | 5.69E-05 | 0.001103884 | 1.62 | 0.508 |
| 12 | 9 | 19 | NM_138476.2 | MDP-1 | 0.52 | 8.31 | 4.33 | 5.81E-05 | 0.001121568 | 1.60 | 0.522 |
| 47 | 23 | 5 | NM_144581.1 | C14orf149 | -0.65 | 9.42 | -4.32 | 5.91E-05 | 0.001133675 | 1.58 | 0.651 |
| 23 | 1 | 7 | BC002488.2 | SERBP1 | 0.57 | 7.35 | 4.31 | 6.08E-05 | 0.001152873 | 1.56 | 0.571 |
| 2 | 1 | 23 | BC000849.1 | ND | 0.50 | 7.57 | 4.31 | 6.16E-05 | 0.001166885 | 1.54 | 0.500 |
| 21 | 21 | 25 | NM_001040428.1 | SPATA7 | -0.58 | 8.70 | -4.30 | 6.35E-05 | 0.001197006 | 1.51 | 0.582 |
| 4 | 1 | 3 | BC007872.2 | TK1 | 0.53 | 7.97 | 4.29 | 6.60E-05 | 0.001224824 | 1.48 | 0.533 |
| 26 | 22 | 19 | BC066915.1 | MARCKSL1 | -0.64 | 7.42 | -4.29 | 6.64E-05 | 0.001229341 | 1.47 | 0.641 |
| 23 | 1 | 29 | NM_020675.3 | SPC25 | 0.51 | 7.33 | 4.29 | 6.72E-05 | 0.001239743 | 1.46 | 0.509 |
| 31 | 1 | 21 | BC020596.1 | CYP2C8 | 0.59 | 7.42 | 4.28 | 6.74672E-05 | 0.001240862 | 1.46 | 0.591 |
| 37 | 19 | 13 | NM_005861.2 | STUB1 | -0.50 | 9.39 | -4.28 | 6.83E-05 | 0.001248493 | 1.45 | 0.503 |
| 27 | 14 | 19 | NM_002767.2 | PRPSAP2 | -0.94 | 8.56 | -4.27 | 7.01803E-05 | 0.001276745 | 1.42 | 0.941 |
| 46 | 21 | 7 | BC031276.1 | LOC158381 | -0.56 | 9.15 | -4.26 | 7.22E-05 | 0.001307946 | 1.39 | 0.562 |
| 27 | 20 | 21 | NM_173663.1 | NY-REN-7 | -0.79 | 7.89 | -4.26 | 7.36E-05 | 0.001322226 | 1.38 | 0.792 |
| 38 | 18 | 11 | NM_017876.3 | RNF126 | -0.53 | 7.96 | -4.26 | 7.45E-05 | 0.001334261 | 1.36 | 0.531 |
| 27 | 1 | 13 | NM_016530.2 | RAB8B | 0.64 | 7.49 | 4.25 | 7.53E-05 | 0.001340674 | 1.35 | 0.636 |
| 12 | 1 | 13 | NM_139355.2 | MATK | 0.59 | 7.37 | 4.25 | 7.58E-05 | 0.001345977 | 1.35 | 0.591 |
| 12 | 3 | 21 | NM_032174.4 | ND | 0.52 | 7.32 | 4.23 | 8.02E-05 | 0.001400986 | 1.30 | 0.516 |
| 23 | 1 | 25 | NM_001737.2 | C9 | 0.51 | 7.42 | 4.23 | 8.19E-05 | 0.001424327 | 1.28 | 0.513 |
| 36 | 22 | 3 | BC099907.1 | GTF2I | -0.52 | 8.28 | -4.23 | 8.24E-05 | 0.001430688 | 1.27 | 0.518 |
| 15 | 2 | 31 | NM_004765.2 | BCL7C | 0.52 | 7.64 | 4.21 | 8.6384E-05 | 0.001486606 | 1.23 | 0.516 |
| 10 | 1 | 19 | NM_016192.2 | ND | 0.50 | 7.41 | 4.21 | 8.74404E-05 | 0.00149306 | 1.21 | 0.501 |
| 38 | 24 | 27 | BC022244.1 | PYCR1 | -0.67 | 8.52 | -4.20 | 8.85E-05 | 0.0015067 | 1.20 | 0.669 |
| 27 | 20 | 11 | NM_021810.3 | CDH26 | -1.04 | 8.49 | -4.20 | 8.86E-05 | 0.0015067 | 1.20 | 1.045 |
| 10 | 1 | 25 | NM_014222.2 | NDUFA8 | 0.54 | 7.95 | 4.20 | 8.94E-05 | 0.001514163 | 1.19 | 0.536 |
| 26 | 23 | 15 | BC012924.1 | DAPP1 | -0.51 | 9.61 | -4.18 | 9.53E-05 | 0.001577262 | 1.13 | 0.507 |
| 40 | 18 | 27 | NM_005488.1 | TOM1 | -0.57 | 10.41 | -4.18 | 9.55E-05 | 0.001579074 | 1.13 | 0.571 |
| 1 | 1 | 31 | NM_012158.1 | FBXL3 | 0.54 | 7.44 | 4.18 | 9.62E-05 | 0.001583806 | 1.12 | 0.538 |
| 27 | 18 | 13 | BC028026.1 | PCGF3 | -0.71 | 7.79 | -4.17 | 9.80E-05 | 0.001604231 | 1.11 | 0.707 |
| 6 | 1 | 7 | NM_021643.1 | ND | 0.61 | 7.91 | 4.17 | 9.91E-05 | 0.001620652 | 1.10 | 0.608 |
| 4 | 1 | 27 | BC009009.1 | PLCB2 | 0.57 | 7.62 | 4.16 | 0.000103337 | 0.001653614 | 1.06 | 0.573 |
| 4 | 2 | 21 | NM_017444.3 | CHRAC1 | 0.51 | 8.36 | 4.15 | 0.000105008 | 0.001668588 | 1.04 | 0.506 |
| 8 | 1 | 3 | NM_020168.3 | PAK6 | 0.57 | 7.45 | 4.14 | 0.000108518 | 0.00170467 | 1.01 | 0.566 |
| 37 | 24 | 9 | NM_014059.1 | C13orf15 | -0.51 | 7.32 | -4.12 | 0.000116717 | 0.00180832 | 0.94 | 0.510 |
| 34 | 23 | 9 | NM_001155.3 | ANXA6 | -0.61 | 8.98 | -4.12 | 0.000117217 | 0.001812617 | 0.94 | 0.610 |
| 1 | 1 | 17 | NM_001017.2 | RPS13 | 0.51 | 7.39 | 4.12 | 0.000117296 | 0.001812617 | 0.94 | 0.510 |
| 7 | 3 | 29 | NM_005151.3 | USP14 | 0.51 | 8.43 | 4.11 | 0.000120505 | 0.001847974 | 0.91 | 0.509 |
| 43 | 23 | 31 | BC110491 | KBTBD5 | -0.54 | 8.13 | -4.10 | 0.000125171 | 0.001901773 | 0.88 | 0.539 |
| 46 | 23 | 31 | BC000446 | CLP1 | -0.68 | 9.64 | -4.10 | 0.000128037 | 0.001936708 | 0.86 | 0.677 |
| 25 | 2 | 19 | NM_002904.5 | ND | 0.52 | 7.50 | 4.09 | 0.000131706 | 0.001972856 | 0.83 | 0.516 |
| 8 | 3 | 3 | BC027877.1 | TEAD3 | 0.51 | 9.63 | 4.08 | 0.00013644 | 0.002025303 | 0.80 | 0.508 |
| 42 | 15 | 31 | BC067254.1 | COASY | -0.71 | 9.54 | -4.07 | 0.000140225 | 0.002072967 | 0.77 | 0.705 |
| 4 | 2 | 9 | NM_145041.1 | TMEM106A | 0.55 | 7.55 | 4.06 | 0.000144462 | 0.00211624 | 0.74 | 0.553 |
| 20 | 1 | 7 | BC013575.2 | ND | 0.54 | 7.34 | 4.05 | 0.000151265 | 0.002180839 | 0.70 | 0.537 |
| 38 | 2 | 1 | NM_001003715.1 | RECQL5 | 0.50 | 7.77 | 4.04 | 0.000154188 | 0.002206277 | 0.68 | 0.503 |
| 44 | 7 | 13 | NM_001157.2 | ANXA11 | 0.86 | 10.19 | 4.03 | 0.000157909 | 0.002242581 | 0.66 | 0.855 |
| 42 | 22 | 17 | BC070274 | PDS5B | -0.50 | 9.61 | -4.03 | 0.000161595 | 0.002274748 | 0.64 | 0.500 |
| 27 | 2 | 15 | NM_014372.3 | RNF11 | -0.99 | 8.59 | -4.02 | 0.000164937 | 0.002309752 | 0.62 | 0.986 |
| 6 | 1 | 17 | BC001665.2 | ABLIM3 | 0.56 | 7.71 | 4.01 | 0.000168509 | 0.00235248 | 0.60 | 0.562 |
| 39 | 21 | 17 | BC099905.1 | MAPK1 | -0.62 | 10.52 | -3.99 | 0.000180405 | 0.002461494 | 0.54 | 0.621 |
| 42 | 19 | 23 | NM_182729.1 | TXNRD1 | -0.58 | 9.41 | -3.99 | 0.000184978 | 0.002509107 | 0.51 | 0.577 |
| 47 | 24 | 9 | NM_182501.2 | MTERFD2 | -0.60 | 9.34 | -3.99 | 0.000185006 | 0.002509107 | 0.51 | 0.600 |
| 18 | 24 | 25 | BC003166.1 | POLE3 | -0.59 | 9.60 | -3.98 | 0.000186156 | 0.002521749 | 0.51 | 0.595 |
| 12 | 1 | 27 | NM_002985.2 | CCL5 | 0.56 | 7.37 | 3.98 | 0.000187461 | 0.002532898 | 0.50 | 0.559 |
| 33 | 1 | 3 | NM_003130.2 | SRI | 0.58 | 8.56 | 3.98 | 0.000187758 | 0.00253501 | 0.50 | 0.576 |
| 38 | 13 | 11 | NM_198204.1 | MLX | -0.79 | 9.42 | -3.98 | 0.000188664 | 0.002545343 | 0.49 | 0.792 |
| 38 | 14 | 29 | BC001487.2 | TARDBP | -0.56 | 8.92 | -3.98 | 0.000190038 | 0.002561972 | 0.49 | 0.561 |
| 45 | 21 | 9 | BC074796.2 | HCRTR1 | -0.55 | 8.12 | -3.97 | 0.00019378 | 0.002598845 | 0.47 | 0.549 |
| 45 | 20 | 19 | BC063384.1 | IGHD | -0.61 | 8.44 | -3.96 | 0.000200074 | 0.00265955 | 0.44 | 0.614 |
| 46 | 24 | 25 | NM_020927.1 | KIAA1576 | -0.68 | 9.86 | -3.96 | 0.000204064 | 0.002700678 | 0.42 | 0.676 |
| 27 | 1 | 9 | BC022548.1 | RASGEF1A | 0.58 | 7.39 | 3.95 | 0.000207619 | 0.002737416 | 0.40 | 0.576 |
| 24 | 1 | 21 | BC018756.1 | MOXD1 | 0.53 | 7.39 | 3.95 | 0.000208621 | 0.0027429 | 0.40 | 0.526 |
| 12 | 1 | 11 | BC007462.1 | CKM | 0.60 | 7.58 | 3.95 | 0.000210691 | 0.002764081 | 0.39 | 0.598 |
| 4 | 2 | 11 | NM_019863.2 | F8 | 0.50 | 7.43 | 3.93 | 0.000225976 | 0.002897276 | 0.33 | 0.504 |
| 11 | 1 | 3 | BC022511.1 | EDNRA | 0.52 | 7.40 | 3.92 | 0.000229381 | 0.002932602 | 0.31 | 0.525 |
| 38 | 23 | 7 | NM_018990.2 | CXorf9 | -0.54 | 8.99 | -3.90 | 0.000243053 | 0.003049047 | 0.26 | 0.540 |
| 4 | 1 | 11 | NM_006748.1 | SLA | 0.54 | 7.93 | 3.90 | 0.000244884 | 0.003061474 | 0.25 | 0.545 |
| 23 | 1 | 9 | BC015684.2 | TRIML1 | 0.57 | 7.78 | 3.89 | 0.000252602 | 0.0031341 | 0.22 | 0.574 |
| 40 | 24 | 11 | NM_004365.2 | CETN3 | -0.54 | 8.75 | -3.89 | 0.00025358 | 0.003141923 | 0.22 | 0.538 |
| 36 | 13 | 5 | NM_002814.2 | PSMD10 | -0.67 | 9.79 | -3.88 | 0.000260065 | 0.003193795 | 0.19 | 0.668 |
| 5 | 20 | 29 | BC080607.1 | TMEM185B | -0.50 | 7.88 | -3.87 | 0.000269662 | 0.003273826 | 0.16 | 0.502 |
| 43 | 20 | 23 | BC094878.1 | ARL2BP | -0.65 | 10.54 | -3.87 | 0.000271164 | 0.003285436 | 0.16 | 0.655 |
| 27 | 15 | 7 | XM_290842.4 | LRFN1 | -1.05 | 8.33 | -3.87 | 0.000271622 | 0.003285968 | 0.15 | 1.047 |
| 4 | 1 | 29 | BC000642.2 | LEPROTL1 | 0.54 | 7.53 | 3.86 | 0.000277877 | 0.00332001 | 0.13 | 0.544 |
| 38 | 19 | 7 | NM_000689.3 | ALDH1A1 | -0.56 | 9.77 | -3.86 | 0.000278812 | 0.003328977 | 0.13 | 0.565 |
| 38 | 23 | 13 | BC006141.1 | EPB41L3 | -0.53 | 7.72 | -3.86 | 0.000280574 | 0.003345585 | 0.12 | 0.526 |
| 3 | 1 | 1 | NM_016103.2 | SAR1B | 0.55 | 8.21 | 3.85 | 0.000287806 | 0.003418291 | 0.10 | 0.546 |
| 9 | 1 | 23 | NM_003944.2 | SELENBP1 | 0.51 | 8.64 | 3.85 | 0.000290558 | 0.003435169 | 0.09 | 0.506 |
| 40 | 19 | 13 | NM_002423.3 | MMP7 | -0.50 | 8.16 | -3.84 | 0.000303377 | 0.003558772 | 0.05 | 0.505 |
| 47 | 1 | 19 | BC009253.2 | ABCF3 | 0.57 | 8.41 | 3.84 | 0.000303646 | 0.003559617 | 0.05 | 0.569 |
| 35 | 21 | 31 | NM_024781.1 | CCDC102B | -0.55 | 9.29 | -3.83 | 0.000309923 | 0.003591246 | 0.03 | 0.550 |
| 27 | 18 | 11 | NM_004663.3 | RAB11A | -0.55 | 8.16 | -3.82 | 0.000319341 | 0.003676479 | 0.00 | 0.552 |
| 4 | 4 | 1 | BC002957.1 | ND | 0.50 | 7.42 | 3.82 | 0.000319517 | 0.003676479 | 0.00 | 0.500 |
| 26 | 24 | 25 | NM_003707.1 | RUVBL1 | -0.51 | 8.99 | -3.80 | 0.000341184 | 0.003830879 | -0.06 | 0.512 |
| 25 | 6 | 19 | NM_003288.2 | TPD52L2 | 0.60 | 9.02 | 3.80 | 0.000342884 | 0.003840531 | -0.06 | 0.596 |
| 27 | 14 | 23 | NM_018584.4 | CAMK2N1 | -1.04 | 9.36 | -3.79 | 0.000351914 | 0.003914615 | -0.09 | 1.036 |
| 36 | 4 | 17 | NM_003099.3 | SNX1 | 0.84 | 9.41 | 3.79 | 0.000353424 | 0.003924159 | -0.09 | 0.843 |
| 23 | 21 | 25 | BC090880.1 | EIF3S3 | -1.05 | 8.29 | -3.79 | 0.000354412 | 0.00393271 | -0.09 | 1.048 |
| 11 | 17 | 7 | NM_022562.2 | ND | 0.53 | 8.68 | 3.78 | 0.000364902 | 0.00401949 | -0.12 | 0.526 |
| 20 | 1 | 29 | NM_005884.3 | PAK4 | 0.51 | 7.45 | 3.77 | 0.000379626 | 0.004138773 | -0.16 | 0.509 |
| 4 | 21 | 7 | BC008897.1 | FLJ22222 | -0.65 | 8.26 | -3.75 | 0.000398257 | 0.004279898 | -0.20 | 0.652 |
| 12 | 1 | 3 | NM_006374.3 | STK25 | 0.68 | 7.36 | 3.75 | 0.000406124 | 0.004338632 | -0.22 | 0.681 |
| 27 | 21 | 31 | NM_001003892.1 | DUPD1 | -0.94 | 8.00 | -3.73 | 0.000425998 | 0.004474214 | -0.26 | 0.940 |
| 20 | 22 | 23 | BC075842.1 | IGHG1 | 0.61 | 10.63 | 3.72 | 0.000440679 | 0.004593691 | -0.30 | 0.605 |
| 8 | 1 | 11 | NM_023031.1 | FGFR2 | 0.67 | 7.51 | 3.71 | 0.000448547 | 0.004651553 | -0.31 | 0.671 |
| 44 | 18 | 27 | BC015797.1 | SLC25A10 | -0.58 | 8.10 | -3.70 | 0.000464196 | 0.004761895 | -0.34 | 0.580 |
| 46 | 15 | 25 | NM_176823.2 | S100A7A | -0.78 | 7.45 | -3.69 | 0.000490045 | 0.004951152 | -0.39 | 0.776 |
| 31 | 1 | 17 | BC020764.1 | FGA | 0.53 | 7.57 | 3.68 | 0.000500199 | 0.005017265 | -0.41 | 0.530 |
| 27 | 19 | 25 | NM_001001552.3 | LEMD1 | -0.87 | 7.86 | -3.68 | 0.000504446 | 0.005048286 | -0.42 | 0.875 |
| 1 | 4 | 7 | NM_000790.2 | DDC | 0.50 | 8.91 | 3.67 | 0.000520351 | 0.005170596 | -0.45 | 0.504 |
| 27 | 15 | 29 | NM_198086.1 | JUB | -1.07 | 8.84 | -3.67 | 0.000524596 | 0.005193906 | -0.46 | 1.072 |
| 12 | 21 | 17 | BC103692.1 | FMN1 | -0.83 | 8.90 | -3.66 | 0.000536419 | 0.005272259 | -0.48 | 0.833 |
| 25 | 23 | 13 | NM_138362.1 | FAM104B | -0.61 | 8.54 | -3.65 | 0.000549409 | 0.005341801 | -0.50 | 0.607 |
| 17 | 2 | 1 | NM_032331.2 | MGC2408 | 0.62 | 10.20 | 3.65 | 0.000549944 | 0.005344132 | -0.50 | 0.617 |
| 37 | 21 | 19 | BC086875.1 | FAM13A1 | -0.51 | 9.07 | -3.65 | 0.000555685 | 0.005385435 | -0.51 | 0.509 |
| 12 | 2 | 11 | BC051822.1 | PECAM1 | 0.56 | 7.42 | 3.65 | 0.000556652 | 0.005391909 | -0.51 | 0.557 |
| 39 | 24 | 31 | NM_001798.2 | N.D. | -0.51 | 8.92 | -3.64 | 0.000577393 | 0.005524684 | -0.55 | 0.514 |
| 36 | 20 | 11 | NM_005642.2 | TAF7 | -0.55 | 8.76 | -3.63 | 0.000591971 | 0.005623351 | -0.57 | 0.547 |
| 48 | 17 | 25 | NM_019088.2 | PAF1 | -0.73 | 9.38 | -3.62 | 0.000604081 | 0.005713466 | -0.59 | 0.726 |
| 47 | 22 | 17 | NM_024610.3 | HSPBAP1 | -0.62 | 10.34 | -3.61 | 0.000617897 | 0.005819761 | -0.61 | 0.621 |
| 16 | 19 | 21 | NM_016224.3 | SNX9 | -1.14 | 7.66 | -3.61 | 0.000618974 | 0.005823837 | -0.61 | 1.137 |
| 1 | 14 | 7 | NM_000507.2 | FBP1 | -0.61 | 8.18 | -3.60 | 0.000646577 | 0.006006038 | -0.65 | 0.607 |
| 46 | 17 | 27 | NM_001009956.1 | ZNF655 | -0.62 | 9.77 | -3.59 | 0.000662399 | 0.006120837 | -0.67 | 0.619 |
| 39 | 16 | 5 | NM_205840.1 | LST1 | -0.71 | 7.44 | -3.59 | 0.000667957 | 0.006159595 | -0.68 | 0.712 |
| 28 | 20 | 27 | NM_003011.2 | SET | -0.53 | 8.75 | -3.58 | 0.000694111 | 0.006337218 | -0.72 | 0.531 |
| 22 | 3 | 3 | NM_000945.3 | PPP3R1 | 0.58 | 10.44 | 3.57 | 0.000701217 | 0.006364357 | -0.73 | 0.576 |
| 12 | 22 | 5 | BC075800.1 | PRKAR2B | -0.66 | 9.28 | -3.57 | 0.000709115 | 0.00641002 | -0.74 | 0.661 |
| 25 | 19 | 11 | NM_020706.1 | SFRS15 | -0.53 | 7.98 | -3.56 | 0.000726882 | 0.006519924 | -0.76 | 0.527 |
| 25 | 1 | 29 | NM_001010.2 | RPS6 | 0.53 | 7.94 | 3.55 | 0.00075249 | 0.00666359 | -0.79 | 0.534 |
| 38 | 11 | 5 | BC012142.1 | ATP6V1C2 | -0.51 | 8.36 | -3.54 | 0.000770746 | 0.006785863 | -0.81 | 0.506 |
| 42 | 20 | 29 | NM_053031.2 | MYLK | -0.63 | 9.43 | -3.54 | 0.000771212 | 0.006786137 | -0.81 | 0.626 |
| 4 | 2 | 13 | NM_003123.3 | SPN | 0.84 | 7.88 | 3.54 | 0.00077546 | 0.00681688 | -0.82 | 0.840 |
| 48 | 20 | 31 | NM_007126.2 | VCP | -0.61 | 9.68 | -3.54 | 0.000779242 | 0.006836818 | -0.82 | 0.609 |
| 3 | 1 | 5 | BC004247.1 | RAC1 | 0.53 | 7.49 | 3.54 | 0.000785924 | 0.006882851 | -0.83 | 0.532 |
| 42 | 22 | 15 | XM_001130991.1 | LOC729447 | -0.54 | 9.15 | -3.53 | 0.000797287 | 0.006934516 | -0.85 | 0.538 |
| 46 | 20 | 31 | NM_031431.2 | COG3 | -0.53 | 7.35 | -3.53 | 0.000803146 | 0.006969639 | -0.85 | 0.533 |
| 4 | 1 | 15 | BC005197.1 | MBIP | 0.51 | 7.74 | 3.53 | 0.000815556 | 0.00704295 | -0.87 | 0.512 |
| 40 | 4 | 19 | NM_012267.3 | HSPBP1 | -0.56 | 9.32 | -3.52 | 0.000820711 | 0.007076916 | -0.87 | 0.563 |
| 41 | 18 | 21 | NM_002664.1 | PLEK | -0.58 | 9.20 | -3.52 | 0.000833195 | 0.007160642 | -0.89 | 0.579 |
| 48 | 15 | 11 | NM_181690.1 | AKT3 | -0.61 | 9.72 | -3.51 | 0.000849757 | 0.007240975 | -0.90 | 0.607 |
| 4 | 1 | 25 | NM_003136.2 | SRP54 | 0.58 | 7.85 | 3.50 | 0.000878951 | 0.007416275 | -0.94 | 0.580 |
| 7 | 3 | 19 | NM_020368.1 | SAS10 | 0.51 | 7.66 | 3.50 | 0.000889773 | 0.007486608 | -0.95 | 0.515 |
| 42 | 18 | 3 | NM_024815.3 | NUDT18 | -0.55 | 9.48 | -3.50 | 0.000898125 | 0.007542828 | -0.96 | 0.548 |
| 8 | 1 | 5 | NM_025211.2 | GKAP1 | 0.59 | 7.73 | 3.49 | 0.000903255 | 0.007575352 | -0.96 | 0.593 |
| 12 | 22 | 3 | BC072461.1 | CHORDC1 | -0.58 | 9.10 | -3.49 | 0.00091299 | 0.007636544 | -0.97 | 0.576 |
| 12 | 1 | 5 | NM_000294.1 | PHKG2 | 0.71 | 7.36 | 3.45 | 0.001040395 | 0.008436387 | -1.09 | 0.707 |
| 6 | 1 | 21 | BC021093.1 | HIATL1 | 0.55 | 7.85 | 3.43 | 0.001112365 | 0.008915208 | -1.15 | 0.555 |
| 12 | 2 | 19 | NM_032380.3 | GFM2 | 0.54 | 7.44 | 3.41 | 0.001176489 | 0.009280902 | -1.20 | 0.536 |
| 6 | 3 | 3 | NM_152377.1 | C1orf87 | 0.53 | 8.09 | 3.39 | 0.001226966 | 0.00959115 | -1.24 | 0.529 |
| 36 | 23 | 23 | Lhx1 | Lhx1 | -0.97 | 8.05 | -3.38 | 0.001262824 | 0.009807788 | -1.27 | 0.965 |
| 40 | 8 | 25 | BC039814.1 | ZRANB2 | -0.53 | 9.35 | -3.37 | 0.001308658 | 0.010045069 | -1.30 | 0.525 |
| 15 | 1 | 5 | NM_153273.3 | IHPK1 | 0.51 | 7.40 | 3.37 | 0.001315567 | 0.010083039 | -1.31 | 0.510 |
| 39 | 22 | 17 | BC012098.1 | GBE1 | -0.63 | 10.04 | -3.35 | 0.001383111 | 0.010476272 | -1.35 | 0.630 |
| 43 | 1 | 9 | NM_003141.3 | ND | 0.52 | 7.53 | 3.34 | 0.001428653 | 0.010762558 | -1.38 | 0.518 |
| 41 | 23 | 27 | BC121798 | EYA1 | -0.66 | 10.07 | -3.34 | 0.001433725 | 0.010791766 | -1.39 | 0.659 |
| 17 | 2 | 5 | NM_001444.1 | FABP5 | 0.55 | 10.18 | 3.34 | 0.001439071 | 0.010818845 | -1.39 | 0.554 |
| 27 | 1 | 29 | NM_005086.3 | SSPN | 0.51 | 7.49 | 3.34 | 0.001439119 | 0.010818845 | -1.39 | 0.509 |
| 39 | 1 | 31 | BC013009.2 | ZMYM3 | 0.52 | 10.12 | 3.32 | 0.001516125 | 0.011251766 | -1.44 | 0.517 |
| 44 | 24 | 27 | NM_002520.5 | NPM1 | -0.51 | 7.69 | -3.32 | 0.001540562 | 0.011350023 | -1.45 | 0.514 |
| 14 | 1 | 27 | BC026335.1 | CORO2B | 0.53 | 7.49 | 3.31 | 0.001602145 | 0.011679886 | -1.49 | 0.532 |
| 12 | 1 | 1 | NM_005627.2 | SGK | 0.70 | 7.38 | 3.30 | 0.001622062 | 0.011758646 | -1.50 | 0.704 |
| 31 | 1 | 9 | BC011707.1 | NRBF2 | 0.52 | 7.63 | 3.30 | 0.001656081 | 0.011962054 | -1.52 | 0.516 |
| 4 | 2 | 17 | NM_016618.1 | KRCC1 | 0.58 | 7.41 | 3.29 | 0.001701289 | 0.012191106 | -1.54 | 0.583 |
| 7 | 1 | 7 | NM_004804.2 | CIAO1 | 0.56 | 7.52 | 3.27 | 0.001766344 | 0.012500968 | -1.58 | 0.556 |
| 22 | 19 | 29 | NM_018290.2 | PGM2 | -0.52 | 9.64 | -3.27 | 0.001780872 | 0.012581715 | -1.58 | 0.520 |
| 8 | 1 | 1 | BC022483.1 | ARHGAP29 | 0.52 | 7.70 | 3.21 | 0.002142826 | 0.014509052 | -1.75 | 0.521 |
| 27 | 2 | 1 | NM_016072.2 | GOLT1B | 0.52 | 7.40 | 3.19 | 0.002248938 | 0.014991535 | -1.80 | 0.522 |
| 30 | 20 | 31 | NM_020805.1 | KLHL14 | -0.58 | 9.42 | -3.18 | 0.002361686 | 0.015523129 | -1.84 | 0.575 |
| 30 | 20 | 29 | NM_134268.3 | CYGB | -0.58 | 9.72 | -3.18 | 0.002362197 | 0.015523129 | -1.84 | 0.583 |
| 46 | 20 | 29 | NM_020532.4 | RTN4 | -0.62 | 7.41 | -3.11 | 0.002845426 | 0.017860536 | -2.01 | 0.621 |
| 27 | 13 | 1 | NM_024928.3 | OBFC1 | -0.58 | 10.50 | -3.10 | 0.002925634 | 0.018281417 | -2.04 | 0.583 |
| 7 | 1 | 1 | BC005825.2 | PDXK | 0.53 | 7.78 | 3.09 | 0.003016424 | 0.01862964 | -2.07 | 0.529 |
| 11 | 6 | 5 | BC002769.1 | C20orf43 | 0.62 | 9.68 | 3.08 | 0.003167228 | 0.019323193 | -2.11 | 0.623 |
| 33 | 2 | 17 | BC018747.1 | IGHG1 | 0.85 | 10.00 | 3.02 | 0.003723916 | 0.02182048 | -2.26 | 0.852 |
| 41 | 24 | 3 | BC014953.1 | C6orf115 | -0.60 | 9.71 | -3.00 | 0.003890442 | 0.022456071 | -2.30 | 0.601 |
| 48 | 24 | 17 | NM_020216.3 | RNPEP | -0.52 | 10.43 | -2.98 | 0.004163066 | 0.023601019 | -2.36 | 0.523 |
| 27 | 20 | 27 | BC082258.1 | RP11-56A21.1 | -0.73 | 7.66 | -2.94 | 0.004614261 | 0.025348949 | -2.45 | 0.731 |
| 2 | 15 | 9 | NM_032531.2 | ND | 0.90 | 9.62 | 2.94 | 0.004630953 | 0.025404847 | -2.45 | 0.902 |
| 25 | 24 | 15 | NM_174901.3 | FAM9C | -0.55 | 8.84 | -2.94 | 0.004646601 | 0.025475218 | -2.46 | 0.552 |
| 46 | 11 | 31 | BC013426.1 | PLEKHG2 | -0.83 | 9.03 | -2.90 | 0.005138291 | 0.027325057 | -2.55 | 0.827 |
| 40 | 18 | 21 | NM_001003954.1 | ANXA13 | -0.55 | 9.29 | -2.90 | 0.005210425 | 0.027549005 | -2.56 | 0.548 |
| 37 | 1 | 15 | BC004983.1 | NFKBIA | 0.52 | 8.62 | 2.87 | 0.005699403 | 0.029313154 | -2.64 | 0.518 |
| 16 | 1 | 1 | NM_080916.1 | DGUOK | 0.53 | 7.73 | 2.84 | 0.006227691 | 0.031233548 | -2.72 | 0.528 |
| 27 | 18 | 31 | NM_006541.1 | TXNL2 | -0.60 | 8.27 | -2.83 | 0.006249894 | 0.031299065 | -2.72 | 0.604 |
| 46 | 18 | 23 | P49189 | ALDH9A1 | -0.63 | 9.93 | -2.80 | 0.006871265 | 0.033503215 | -2.81 | 0.628 |
| 24 | 15 | 25 | NM_005465.3 | AKT3 | -0.73 | 9.44 | -2.77 | 0.00748369 | 0.035657653 | -2.88 | 0.729 |
| 27 | 24 | 31 | BC009388.1 | LOC554174 | -0.56 | 7.96 | -2.74 | 0.008060719 | 0.037757433 | -2.95 | 0.555 |
| 24 | 1 | 1 | NM_024779.3 | PIP5K2C | 0.53 | 8.10 | 2.72 | 0.008575062 | 0.039537648 | -3.01 | 0.527 |
| 30 | 21 | 27 | NM_001106.3 | ACVR2B | -0.54 | 7.76 | -2.69 | 0.00929213 | 0.042094827 | -3.08 | 0.543 |
| 23 | 14 | 29 | NM_177965.2 | C8orf37 | -0.62 | 10.29 | -2.69 | 0.009346783 | 0.042244091 | -3.08 | 0.621 |
| 36 | 10 | 5 | BC051868.2 | UBE2O | -0.61 | 8.46 | -2.68 | 0.009427724 | 0.042516534 | -3.09 | 0.609 |
| 31 | 4 | 3 | NM_002857.2 | PEX19 | 0.57 | 9.83 | 2.66 | 0.01000431 | 0.044431931 | -3.14 | 0.567 |
| 41 | 14 | 19 | NM_024826.1 | ASAP | 0.59 | 10.25 | 2.62 | 0.011063329 | 0.047778874 | -3.23 | 0.591 |
| 27 | 12 | 27 | NM_199337.1 | LOC374395 | -0.71 | 8.32 | -2.60 | 0.011609619 | 0.04948744 | -3.28 | 0.711 |

**1.3: Shortlisted proteins. List of proteins with p-value<0.05 and logFC >1 or <-1**

**(MG1vsHC)**

|  | | | | | | |  |  |  |  |  |
| --- | --- | --- | --- | --- | --- | --- | --- | --- | --- | --- | --- |
| **Block** | **Row** | **Column** | **ID** | **Name** | **logFC** | **AveExpr** | **t** | **P.Value** | **adj.P.Val** | **B** | **abs log FC** |
| 41 | 5 | 11 | BC025985.1 | IGHG4 | -3.15 | 8.68 | -30.04 | 8.02E-38 | 1.45E-33 | 68.7 | 3.15 |
| 10 | 18 | 29 | NM_001014444.1 | CRYM | -1.44 | 7.79 | -22.59 | 5.31E-31 | 4.80E-27 | 56.5 | 1.44 |
| 3 | 3 | 21 | NM_032328.1 | EFCAB2 | 1.10 | 9.67 | 9.08 | 7.41E-13 | 2.68E-09 | 18.9 | 1.10 |
| 38 | 18 | 5 | NM_031304.2 | DOHH | -1.01 | 10.06 | -8.89 | 1.51E-12 | 4.55E-09 | 18.2 | 1.01 |
| 14 | 20 | 19 | NM_015726.2 | WDR42A | -1.31 | 8.72 | -8.28 | 1.62E-11 | 3.66E-08 | 16.0 | 1.31 |
| 27 | 13 | 15 | BC065370.1 | C20orf112 | -1.83 | 9.13 | -7.66 | 1.86E-10 | 1.87E-07 | 13.6 | 1.83 |
| 27 | 3 | 21 | BC006453.1 | HDAC7A | 1.00 | 8.08 | 7.62 | 2.23E-10 | 2.12E-07 | 13.5 | 1.00 |
| 27 | 21 | 1 | BC037876.1 | C17orf57 | -1.55 | 8.24 | -7.42 | 4.76E-10 | 3.58E-07 | 12.7 | 1.55 |
| 27 | 22 | 17 | NM_001033515.1 | LOC389833 | -1.14 | 8.32 | -7.27 | 8.70E-10 | 5.51E-07 | 12.2 | 1.14 |
| 27 | 12 | 25 | NM_001005465.1 | ND | -1.44 | 10.01 | -6.04 | 1.08E-07 | 1.56E-05 | 7.6 | 1.44 |
| 8 | 16 | 11 | NM_139204.1 | EPS8L1 | 1.22 | 8.43 | 5.11 | 3.48E-06 | 0.000170906 | 4.3 | 1.22 |
| 35 | 15 | 13 | NM_001025266.1 | LOC285382 | 1.32 | 8.63 | 4.99 | 5.53E-06 | 0.000230409 | 3.8 | 1.32 |
| 27 | 14 | 17 | NM_148910.2 | TIRAP | -1.05 | 8.43 | -4.64 | 1.93E-05 | 0.000537739 | 2.6 | 1.05 |
| 27 | 15 | 13 | NM_002893.2 | RBBP7 | -1.00 | 8.12 | -4.43 | 4.05E-05 | 0.000866432 | 1.9 | 1.00 |
| 27 | 20 | 11 | NM_021810.3 | CDH26 | -1.04 | 8.49 | -4.20 | 8.86E-05 | 0.0015067 | 1.2 | 1.04 |
| 27 | 15 | 7 | XM_290842.4 | LRFN1 | -1.05 | 8.33 | -3.87 | 0.000271622 | 0.003285968 | 0.2 | 1.05 |
| 27 | 14 | 23 | NM_018584.4 | CAMK2N1 | -1.04 | 9.36 | -3.79 | 0.000351914 | 0.003914615 | -0.1 | 1.04 |
| 23 | 21 | 25 | BC090880.1 | EIF3S3 | -1.05 | 8.29 | -3.79 | 0.000354412 | 0.00393271 | -0.1 | 1.05 |
| 27 | 15 | 29 | NM_198086.1 | JUB | -1.07 | 8.84 | -3.67 | 0.000524596 | 0.005193906 | -0.5 | 1.07 |
| 16 | 19 | 21 | NM_016224.3 | SNX9 | -1.14 | 7.66 | -3.61 | 0.000618974 | 0.005823837 | -0.6 | 1.14 |
