# Supplementary material for: Evaluation of autoantibody signatures in meningioma patients using human proteome arrays
Source: Oncotarget. 2017 Apr 10;8(35):58443–56. doi: 10.18632/oncotarget.16997 (PMC5601665; doi:10.18632/oncotarget.16997)
Supplement: Supplementary file 9 [file oncotarget-08-58443-s009.docx]

**Supplementary Table 2.1**:

**Experimental details.** The details of the healthy control and Grade II Meningioma samples.

|  | | | |  |
| --- | --- | --- | --- | --- |
| **Sample ID** | **Sample Type** | **File name** | **Age (years)** | **Sex** |
| H-02 | Control | Control_H-02_2000153953.gpr | 23 | M |
| H-03 | Control | Control_H-03_2000153943.gpr | 27 | M |
| H-19 | Control | Control_H-19_2000154008.gpr | 18 | F |
| H-23 | Control | Control_H-23_2000154009.gpr | 20 | F |
| H-25 | Control | Control_H-25_2000153952.gpr | 19 | F |
| H-35 | Control | Control_H-35_2000153942.gpr | 20 | F |
| H-41 | Control | Control_H-41_2000154026.gpr | 19 | F |
| H-58 | Control | Control_H-58_2000153935.gpr | NA | NA |
| H-59 | Control | Control_H-59_2000154016.gpr | NA | NA |
| HC-25 | Control | Control_HC-25_2000153932.gpr | NA | NA |
| HV-56 | Control | Control_HV-56_2000155735.gpr | 35 | M |
| HV-59 | Control | Control_HV-59_2000155740.gpr | 42 | M |
| HV-64 | Control | Control_HV-64_2000144456.gpr | 31 | M |
| HV-70 | Control | Control_HV-70_2000144457.gpr | NA | NA |
| HV-71 | Control | Control_HV-71_2000144458.gpr | NA | NA |
| CH 17967 | MGGrade2 | MG_Grade_II_CH 17967_2000153914.gpr | 58 | M |
| CJ 3577 | MGGrade2 | MG_Grade_II_CJ 3577_2000153910.gpr | 43 | **F** |
| CJ 15491 | MGGrade2 | MG_Grade_II_CJ 15491_2000153803.gpr | 67 | M |
| CJ 15753 | MGGrade2 | MG_Grade_II_CJ 15753_2000153802.gpr | 46 | F |
| CK 7710 | MGGrade2 | MG_Grade_II_CK 7710_2000153907.gpr | 54 | F |

'NA' indicates not availability of age and sex details; 'M': Indicates Male; 'F' indicates Female.

**Supplementary Table 2.2**: **Shortlisted proteins.** List of proteins with p-value<0.05 and logFC >0.5 or <-0.5 MGIIvsHC

|  | | | | | | |  |  |  |  |  |
| --- | --- | --- | --- | --- | --- | --- | --- | --- | --- | --- | --- |
| **Block** | **Row** | **Column** | **ID** | **Name** | **logFC** | **AveExpr** | **t** | **P.Value** | **adj.P.Val** | **B** | **abs log FC** |
| 41 | 5 | 11 | BC025985.1 | IGHG4 | -3.146 | 8.676 | -23.728035 | 3.74E-32 | 6.76E-28 | 54.06 | 3.15 |
| 10 | 18 | 29 | NM_001014444.1 | CRYM | -1.427 | 7.793 | -17.6457552 | 2.09E-25 | 1.89E-21 | 42.78 | 1.43 |
| 1 | 14 | 17 | NM_171830.1 | KCNMB3 | -0.758 | 7.526 | -9.00953511 | 9.65E-13 | 5.81E-09 | 18.08 | 0.76 |
| 27 | 13 | 15 | BC065370.1 | C20orf112 | -2.184 | 9.126 | -7.22644395 | 1.04E-09 | 4.68E-06 | 11.74 | 2.18 |
| 3 | 3 | 21 | NM_032328.1 | EFCAB2 | 1.060 | 9.673 | 6.935432576 | 3.25E-09 | 1.03E-05 | 10.70 | 1.06 |
| 21 | 18 | 15 | NM_005719.2 | ARPC3 | -1.484 | 8.351 | -6.92092978 | 3.44E-09 | 1.03E-05 | 10.65 | 1.48 |
| 39 | 6 | 27 | NM_001042476.1 | CARHSP1 | -1.125 | 8.050 | -6.58832321 | 1.26E-08 | 3.26E-05 | 9.45 | 1.13 |
| 43 | 1 | 27 | NM_006857.1 | RY1 | 0.877 | 9.307 | 6.470773417 | 2.00E-08 | 4.01E-05 | 9.03 | 0.88 |
| 27 | 21 | 1 | BC037876.1 | C17orf57 | -1.669 | 8.240 | -6.31064334 | 3.72E-08 | 6.72E-05 | 8.46 | 1.67 |
| 27 | 22 | 1 | BC073856.1 | ADRB2 | -0.603 | 7.429 | -6.16136288 | 6.63E-08 | 0.00010889 | 7.93 | 0.60 |
| 3 | 4 | 19 | NM_019102.2 | HOXA5 | -0.703 | 7.622 | -6.12465994 | 7.64E-08 | 0.000115013 | 7.80 | 0.70 |
| 10 | 21 | 5 | BC062437.1 | COX4I1 | -0.617 | 7.627 | -5.99949017 | 1.24E-07 | 0.000159651 | 7.36 | 0.62 |
| 44 | 3 | 9 | BC013019.2 | CCDC28A | 0.652 | 8.904 | 5.941490839 | 1.55E-07 | 0.000186179 | 7.15 | 0.65 |
| 29 | 6 | 31 | NM_004264.2 | SURB7 | -1.043 | 7.641 | -5.8900679 | 1.88E-07 | 0.000212566 | 6.97 | 1.04 |
| 23 | 15 | 29 | NM_003153.3 | STAT6 | -0.583 | 7.383 | -5.83013296 | 2.37E-07 | 0.000251605 | 6.76 | 0.58 |
| 40 | 3 | 21 | NM_182789.2 | PAIP1 | 1.065 | 9.130 | 5.576329014 | 6.21E-07 | 0.000590618 | 5.87 | 1.06 |
| 36 | 1 | 17 | NM_014814.1 | PSMD6 | 0.536 | 7.822 | 5.556508111 | 6.69E-07 | 0.000604708 | 5.81 | 0.54 |
| 27 | 22 | 17 | NM_001033515.1 | LOC389833 | -1.091 | 8.321 | -5.50595543 | 8.10E-07 | 0.000644278 | 5.63 | 1.09 |
| 36 | 1 | 15 | BC013992.1 | MAPK3 | 1.088 | 9.260 | 5.50263171 | 8.20E-07 | 0.000644278 | 5.62 | 1.09 |
| 27 | 3 | 21 | BC006453.1 | HDAC7A | 0.913 | 8.076 | 5.474183355 | 9.13E-07 | 0.000687197 | 5.52 | 0.91 |
| 36 | 3 | 13 | NM_004147.3 | DRG1 | 0.508 | 7.652 | 5.391519691 | 1.24E-06 | 0.000876425 | 5.24 | 0.51 |
| 6 | 7 | 21 | BC033854.1 | GSG1 | 0.730 | 8.869 | 5.348621857 | 1.46E-06 | 0.000977935 | 5.09 | 0.73 |
| 27 | 14 | 19 | NM_002767.2 | PRPSAP2 | -1.464 | 8.555 | -5.25497106 | 2.07E-06 | 0.001290634 | 4.77 | 1.46 |
| 22 | 3 | 5 | BC026107.2 | KRR1 | 0.624 | 8.579 | 5.17552586 | 2.78E-06 | 0.001674838 | 4.50 | 0.62 |
| 40 | 7 | 13 | BC015738.1 | ZFYVE19 | 0.748 | 8.915 | 5.156706683 | 2.98E-06 | 0.00173755 | 4.43 | 0.75 |
| 11 | 1 | 19 | NM_031966.2 | CCNB1 | 0.780 | 8.988 | 5.085598956 | 3.87E-06 | 0.002058848 | 4.19 | 0.78 |
| 25 | 23 | 17 | BC110374 | CORO1A | -0.776 | 7.658 | -5.04892929 | 4.43E-06 | 0.002288326 | 4.07 | 0.78 |
| 3 | 13 | 9 | NM_013409.1 | FST | 0.590 | 9.641 | 5.019093376 | 4.95E-06 | 0.002414696 | 3.97 | 0.59 |
| 38 | 3 | 5 | NM_014078.4 | MRPL13 | 0.712 | 7.784 | 4.992491891 | 5.45E-06 | 0.002591376 | 3.88 | 0.71 |
| 27 | 2 | 15 | NM_014372.3 | RNF11 | -1.535 | 8.585 | -4.9463538 | 6.45E-06 | 0.002987741 | 3.73 | 1.53 |
| 16 | 2 | 1 | NM_007198.2 | PROSC | 0.694 | 8.519 | 4.911427252 | 7.32E-06 | 0.003194506 | 3.61 | 0.69 |
| 2 | 22 | 17 | BC078671.1 | IGHG1 | 0.614 | 12.151 | 4.895710646 | 7.75E-06 | 0.003194506 | 3.56 | 0.61 |
| 34 | 3 | 5 | NM_003732.2 | EIF4EBP3 | 0.696 | 9.512 | 4.843614368 | 9.36E-06 | 0.003524178 | 3.38 | 0.70 |
| 26 | 13 | 9 | NM_007280.1 | OIP5 | 0.651 | 8.831 | 4.830257902 | 9.83E-06 | 0.003622918 | 3.34 | 0.65 |
| 7 | 13 | 5 | BC062688.1 | PRKG1 | 0.636 | 10.451 | 4.818017993 | 1.03E-05 | 0.003663871 | 3.30 | 0.64 |
| 36 | 12 | 9 | ENST00000228872 | CDKN1B | 0.577 | 9.857 | 4.810673006 | 1.05E-05 | 0.003663871 | 3.28 | 0.58 |
| 30 | 3 | 21 | BC006011.1 | DDI2 | 0.639 | 8.789 | 4.787188632 | 1.15E-05 | 0.003839614 | 3.20 | 0.64 |
| 19 | 9 | 19 | ENST00000435033 | NA | 0.572 | 9.268 | 4.77600359 | 1.19E-05 | 0.00385449 | 3.16 | 0.57 |
| 38 | 20 | 29 | NM_080820.3 | DTD1 | -0.529 | 7.556 | -4.73324236 | 1.39E-05 | 0.004342601 | 3.02 | 0.53 |
| 13 | 22 | 9 | BC064383.1 | SLC39A9 | -0.554 | 7.683 | -4.72509213 | 1.43E-05 | 0.004342601 | 2.99 | 0.55 |
| 40 | 5 | 27 | BC020637.1 | IL1F7 | 0.697 | 8.999 | 4.723544376 | 1.44E-05 | 0.004342601 | 2.99 | 0.70 |
| 7 | 13 | 9 | NM_002297.2 | LCN1 | 0.693 | 9.512 | 4.625645953 | 2.04E-05 | 0.004954568 | 2.67 | 0.69 |
| 11 | 1 | 9 | NM_002867.2 | RAB3B | 0.731 | 9.261 | 4.614865705 | 2.12E-05 | 0.005050338 | 2.63 | 0.73 |
| 11 | 1 | 27 | NM_001381.2 | DOK1 | 0.609 | 9.103 | 4.58067627 | 2.40E-05 | 0.005417872 | 2.52 | 0.61 |
| 47 | 3 | 25 | NM_016246.2 | HSD17B14 | 0.527 | 8.604 | 4.580557659 | 2.40E-05 | 0.005417872 | 2.52 | 0.53 |
| 43 | 3 | 11 | NM_003350.2 | UBE2V2 | 0.657 | 8.639 | 4.56729435 | 2.51E-05 | 0.005607862 | 2.48 | 0.66 |
| 28 | 15 | 31 | NM_173809.2 | BLOC1S2 | -2.597 | 9.020 | -4.5153262 | 3.02E-05 | 0.006572942 | 2.31 | 2.60 |
| 40 | 16 | 9 | NM_032955.1 | AIF1 | 0.694 | 9.538 | 4.448130144 | 3.82E-05 | 0.007846226 | 2.10 | 0.69 |
| 15 | 3 | 27 | BC004130.2 | CALCOCO2 | 0.593 | 9.362 | 4.433326982 | 4.02E-05 | 0.008078891 | 2.05 | 0.59 |
| 36 | 12 | 5 | NM_000231.1 | SGCG | 0.514 | 9.500 | 4.298704772 | 6.42E-05 | 0.011257445 | 1.62 | 0.51 |
| 8 | 16 | 11 | NM_139204.1 | EPS8L1 | 1.292 | 8.431 | 4.295865147 | 6.48E-05 | 0.011258731 | 1.62 | 1.29 |
| 35 | 15 | 13 | NM_001025266.1 | LOC285382 | 1.429 | 8.631 | 4.284013809 | 6.75E-05 | 0.011398435 | 1.58 | 1.43 |
| 26 | 20 | 5 | NM_002899.2 | RBP1 | -0.508 | 7.542 | -4.22287797 | 8.32E-05 | 0.013480022 | 1.39 | 0.51 |
| 38 | 19 | 9 | NM_002257.2 | KLK1 | -0.665 | 8.266 | -4.17625999 | 9.76E-05 | 0.015463125 | 1.24 | 0.66 |
| 2 | 21 | 3 | BC062336.1 | IGHG1 | 0.570 | 12.500 | 4.172995011 | 9.87E-05 | 0.015473454 | 1.23 | 0.57 |
| 27 | 15 | 29 | NM_198086.1 | JUB | -1.533 | 8.843 | -4.14439052 | 0.000108723 | 0.016644712 | 1.15 | 1.53 |
| 46 | 21 | 9 | BC036124.1 | MIPOL1 | -0.671 | 8.130 | -4.12398365 | 0.000116493 | 0.017318239 | 1.08 | 0.67 |
| 39 | 3 | 15 | NM_022453.2 | RNF25 | 0.670 | 9.319 | 4.122807563 | 0.000116957 | 0.017318239 | 1.08 | 0.67 |
| 22 | 10 | 25 | NM_182643.1 | DLC1 | 0.552 | 8.814 | 4.111841979 | 0.000121368 | 0.017825316 | 1.05 | 0.55 |
| 27 | 23 | 21 | BC109063 | TRIM68 | -0.581 | 7.415 | -4.06067231 | 0.000144168 | 0.020579118 | 0.89 | 0.58 |
| 3 | 3 | 11 | NM_001001394.2 | HCG3 | 0.593 | 9.068 | 4.058501498 | 0.000145221 | 0.020579118 | 0.88 | 0.59 |
| 27 | 23 | 25 | BC106934 | CCT4 | -0.574 | 7.440 | -4.03410615 | 0.000157582 | 0.021809658 | 0.81 | 0.57 |
| 15 | 1 | 13 | NM_002149.2 | HPCAL1 | 0.703 | 9.111 | 4.033021655 | 0.000158155 | 0.021809658 | 0.80 | 0.70 |
| 11 | 1 | 13 | BC001360.2 | RHOA | 0.630 | 8.875 | 4.002985591 | 0.00017483 | 0.023362349 | 0.71 | 0.63 |
| 9 | 5 | 9 | NM_031911.3 | C1QTNF7 | 0.636 | 8.487 | 4.000278821 | 0.000176414 | 0.023362349 | 0.71 | 0.64 |
| 3 | 19 | 5 | NM_003690.3 | PRKRA | -0.589 | 7.938 | -3.97606643 | 0.000191206 | 0.024154737 | 0.63 | 0.59 |
| 33 | 15 | 25 | BC007706.2 | FAM105B | -0.767 | 7.910 | -3.96045001 | 0.000201372 | 0.024579632 | 0.59 | 0.77 |
| 40 | 1 | 29 | NM_018147.2 | FAIM | 0.569 | 8.574 | 3.952737574 | 0.000206583 | 0.024879479 | 0.56 | 0.57 |
| 27 | 14 | 17 | NM_148910.2 | TIRAP | -1.129 | 8.429 | -3.94128527 | 0.000214561 | 0.025333628 | 0.53 | 1.13 |
| 38 | 18 | 5 | NM_031304.2 | DOHH | -0.564 | 10.057 | -3.93032913 | 0.00022247 | 0.025928566 | 0.50 | 0.56 |
| 22 | 3 | 7 | BC027956.1 | SULT1E1 | 0.587 | 8.650 | 3.925411782 | 0.000226111 | 0.025967312 | 0.48 | 0.59 |
| 24 | 1 | 15 | NM_021131.3 | PPP2R4 | 0.600 | 8.189 | 3.908998865 | 0.000238681 | 0.026615888 | 0.43 | 0.60 |
| 12 | 1 | 15 | NM_001827.1 | CKS2 | 0.714 | 8.339 | 3.885540305 | 0.00025782 | 0.027319456 | 0.36 | 0.71 |
| 14 | 1 | 17 | NM_022107.1 | GPSM3 | 0.589 | 8.712 | 3.885233638 | 0.00025808 | 0.027319456 | 0.36 | 0.59 |
| 27 | 1 | 27 | BC017864.1 | GYPE | 0.560 | 7.959 | 3.871662637 | 0.000269828 | 0.027816208 | 0.32 | 0.56 |
| 5 | 20 | 29 | BC080607.1 | TMEM185B | -0.635 | 7.880 | -3.87033736 | 0.000271002 | 0.027816208 | 0.32 | 0.64 |
| 16 | 10 | 29 | BC046238.1 | POLR3B | 0.504 | 8.775 | 3.867405598 | 0.000273618 | 0.027925994 | 0.31 | 0.50 |
| 25 | 13 | 9 | BC002448.2 | ABLIM1 | 0.592 | 8.383 | 3.863746985 | 0.000276915 | 0.027982317 | 0.30 | 0.59 |
| 44 | 3 | 11 | NM_000432.2 | MYL2 | 0.650 | 8.150 | 3.821410703 | 0.000317964 | 0.029608337 | 0.17 | 0.65 |
| 27 | 20 | 9 | NM_003359.2 | UGDH | -0.851 | 7.751 | -3.81913263 | 0.000320331 | 0.029675762 | 0.17 | 0.85 |
| 27 | 12 | 25 | NM_001005465.1 | OR10G3 | -1.152 | 10.012 | -3.80399528 | 0.000336492 | 0.030700661 | 0.12 | 1.15 |
| 36 | 4 | 17 | NM_003099.3 | SNX1 | 1.067 | 9.411 | 3.790649875 | 0.000351387 | 0.031225561 | 0.08 | 1.07 |
| 27 | 18 | 11 | NM_004663.3 | RAB11A | -0.691 | 8.160 | -3.77899982 | 0.000364904 | 0.031845336 | 0.05 | 0.69 |
| 37 | 5 | 29 | BC014969.1 | ATF6 | 0.866 | 9.351 | 3.762983224 | 0.000384303 | 0.032791621 | 0.00 | 0.87 |
| 21 | 12 | 11 | BC020719.1 | HBG1 | 0.590 | 10.422 | 3.754984049 | 0.000394357 | 0.032829797 | -0.02 | 0.59 |
| 44 | 2 | 25 | NM_001902.4 | CTH | 0.579 | 8.855 | 3.742551906 | 0.000410486 | 0.033113875 | -0.06 | 0.58 |
| 25 | 1 | 7 | NM_020677.2 | NMRAL1 | 0.635 | 8.102 | 3.74180184 | 0.000411479 | 0.033113875 | -0.06 | 0.64 |
| 13 | 1 | 7 | NM_002970.1 | SAT1 | 0.548 | 8.436 | 3.740336556 | 0.000413425 | 0.033113875 | -0.07 | 0.55 |
| 11 | 10 | 3 | BC016967.1 | RPUSD2 | 0.605 | 8.228 | 3.739704842 | 0.000414267 | 0.033113875 | -0.07 | 0.60 |
| 44 | 1 | 17 | NM_006520.1 | DYNLT3 | 0.530 | 8.007 | 3.728291698 | 0.000429763 | 0.034051197 | -0.10 | 0.53 |
| 27 | 21 | 7 | NM_001039656.1 | MTL5 | -0.948 | 7.811 | -3.69775258 | 0.000474002 | 0.035687485 | -0.19 | 0.95 |
| 8 | 3 | 3 | BC027877.1 | TEAD3 | 0.579 | 9.626 | 3.67711253 | 0.000506333 | 0.037037479 | -0.25 | 0.58 |
| 42 | 3 | 7 | NM_000184.2 | HBG2 | 0.726 | 9.361 | 3.677066125 | 0.000506408 | 0.037037479 | -0.25 | 0.73 |
| 20 | 11 | 3 | BC008254.1 | LOC339803 | 0.544 | 8.960 | 3.661619318 | 0.000531975 | 0.038103831 | -0.29 | 0.54 |
| 26 | 23 | 17 | Dlx5 | Dlx5 | -0.527 | 7.830 | -3.65127696 | 0.000549777 | 0.038676499 | -0.32 | 0.53 |
| 38 | 3 | 3 | BC028123.1 | MRVI1-AS1 | 0.561 | 8.426 | 3.647327652 | 0.000556724 | 0.038981439 | -0.33 | 0.56 |
| 25 | 1 | 21 | NM_004359.1 | CDC34 | 0.592 | 8.465 | 3.627608611 | 0.000592681 | 0.040100337 | -0.39 | 0.59 |
| 36 | 23 | 21 | Nol3 | Nol3 | -1.876 | 11.320 | -3.58637095 | 0.00067517 | 0.043296146 | -0.51 | 1.88 |
| 20 | 10 | 3 | BC000870.1 | TIPIN | 0.589 | 8.995 | 3.583533646 | 0.00068123 | 0.04333248 | -0.51 | 0.59 |
| 27 | 15 | 7 | XM_290842.4 | LRFN1 | -1.224 | 8.326 | -3.57514752 | 0.000699448 | 0.043721551 | -0.54 | 1.22 |
| 33 | 1 | 11 | BC011913.2 | P2RX7 | 0.514 | 8.274 | 3.567254338 | 0.000717018 | 0.044359358 | -0.56 | 0.51 |
| 15 | 4 | 5 | BC000116.1 | KIAA0174 | 0.606 | 9.334 | 3.543055328 | 0.000773542 | 0.046644735 | -0.63 | 0.61 |
| 15 | 6 | 31 | NM_001033112.1 | PAIP2 | 1.041 | 9.637 | 3.531553929 | 0.00080187 | 0.047807833 | -0.66 | 1.04 |
| 38 | 20 | 13 | BC068569.1 | ZHX3 | -0.548 | 9.233 | -3.52568633 | 0.000816699 | 0.048531791 | -0.68 | 0.55 |

**Supplementary Table 2.3**: Shortlisted proteins. List of proteins with p-value<0.05 and logFC >1 or <-1 MGII vs HC

|  | | | | | | |  |  |  |  |  |
| --- | --- | --- | --- | --- | --- | --- | --- | --- | --- | --- | --- |
| Block | Row | Column | ID | Name | logFC | AveExpr | t | P.Value | adj.P.Val | B | abs log FC |
| 41 | 5 | 11 | BC025985.1 | IGHG4 | -3.14597518 | 8.676403265 | -23.728035 | 3.74027E-32 | 6.7568E-28 | 54.05858942 | 3.14597518 |
| 10 | 18 | 29 | NM_001014444.1 | CRYM | -1.42663612 | 7.793195613 | -17.6457552 | 2.08748E-25 | 1.88552E-21 | 42.77963427 | 1.426636123 |
| 27 | 13 | 15 | BC065370.1 | C20orf112 | -2.18422628 | 9.126461982 | -7.22644395 | 1.03591E-09 | 4.67842E-06 | 11.74396557 | 2.184226275 |
| 3 | 3 | 21 | NM_032328.1 | EFCAB2 | 1.060258749 | 9.673042049 | 6.935432576 | 3.24732E-09 | 1.03492E-05 | 10.69817974 | 1.060258749 |
| 21 | 18 | 15 | NM_005719.2 | ARPC3 | -1.48427948 | 8.351040673 | -6.92092978 | 3.43733E-09 | 1.03492E-05 | 10.64608692 | 1.484279478 |
| 39 | 6 | 27 | NM_001042476.1 | CARHSP1 | -1.12522164 | 8.05024838 | -6.58832321 | 1.26278E-08 | 3.25887E-05 | 9.45311122 | 1.125221637 |
| 27 | 21 | 1 | BC037876.1 | C17orf57 | -1.6692423 | 8.240190755 | -6.31064334 | 3.72019E-08 | 6.72053E-05 | 8.461338025 | 1.669242303 |
| 29 | 6 | 31 | NM_004264.2 | SURB7 | -1.04259446 | 7.640933032 | -5.8900679 | 1.88268E-07 | 0.000212566 | 6.971671483 | 1.042594462 |
| 40 | 3 | 21 | NM_182789.2 | PAIP1 | 1.064745486 | 9.129997192 | 5.576329014 | 6.21187E-07 | 0.000590618 | 5.874663697 | 1.064745486 |
| 27 | 22 | 17 | NM_001033515.1 | LOC389833 | -1.09108686 | 8.320760318 | -5.50595543 | 8.10078E-07 | 0.000644278 | 5.630719737 | 1.091086856 |
| 36 | 1 | 15 | BC013992.1 | MAPK3 | 1.088388385 | 9.260262113 | 5.50263171 | 8.20281E-07 | 0.000644278 | 5.619219657 | 1.088388385 |
| 27 | 14 | 19 | NM_002767.2 | PRPSAP2 | -1.46374973 | 8.555111061 | -5.25497106 | 2.07187E-06 | 0.001290634 | 4.768174532 | 1.463749729 |
| 27 | 2 | 15 | NM_014372.3 | RNF11 | -1.53453961 | 8.585034731 | -4.9463538 | 6.45015E-06 | 0.002987741 | 3.726132726 | 1.534539615 |
| 28 | 15 | 31 | NM_173809.2 | BLOC1S2 | -2.59703456 | 9.019927151 | -4.5153262 | 3.01995E-05 | 0.006572942 | 2.312994338 | 2.59703456 |
| 8 | 16 | 11 | NM_139204.1 | EPS8L1 | 1.292200418 | 8.431421288 | 4.295865147 | 6.48164E-05 | 0.011258731 | 1.616061144 | 1.292200418 |
| 35 | 15 | 13 | NM_001025266.1 | LOC285382 | 1.429112141 | 8.63066804 | 4.284013809 | 6.75136E-05 | 0.011398435 | 1.57890986 | 1.429112141 |
| 27 | 15 | 29 | NM_198086.1 | JUB | -1.53304835 | 8.843021035 | -4.14439052 | 0.000108723 | 0.016644712 | 1.145187718 | 1.533048348 |
| 27 | 14 | 17 | NM_148910.2 | TIRAP | -1.12935444 | 8.42893404 | -3.94128527 | 0.000214561 | 0.025333628 | 0.528049891 | 1.129354442 |
| 27 | 12 | 25 | NM_001005465.1 | OR10G3 | -1.15204997 | 10.01249218 | -3.80399528 | 0.000336492 | 0.030700661 | 0.120820834 | 1.152049971 |
| 36 | 4 | 17 | NM_003099.3 | SNX1 | 1.067016442 | 9.410595537 | 3.790649875 | 0.000351387 | 0.031225561 | 0.081684513 | 1.067016442 |
| 36 | 23 | 21 | Nol3 | Nol3 | -1.87647287 | 11.3195451 | -3.58637095 | 0.00067517 | 0.043296146 | -0.5069646 | 1.876472872 |
| 27 | 15 | 7 | XM_290842.4 | LRFN1 | -1.22388227 | 8.326399746 | -3.57514752 | 0.000699448 | 0.043721551 | -0.53872221 | 1.223882265 |
| 15 | 6 | 31 | NM_001033112.1 | PAIP2 | 1.040697871 | 9.637179898 | 3.531553929 | 0.00080187 | 0.047807833 | -0.66147766 | 1.040697871 |
