# Supplementary material for: Evaluation of autoantibody signatures in meningioma patients using human proteome arrays
Source: Oncotarget. 2017 Apr 10;8(35):58443–56. doi: 10.18632/oncotarget.16997 (PMC5601665; doi:10.18632/oncotarget.16997)
Supplement: Supplementary file 10 [file oncotarget-08-58443-s010.docx]

| **Table 3Sa: Experimental details.** The details of the healthy control and Meningioma samples. | | | |  |
| --- | --- | --- | --- | --- |
| **Sample ID** | **Sample Type** | **File name** | **Age (years)** | **Sex** |
| H-02 | Control | Control_H-02_2000153953.gpr | 23 | M |
| H-03 | Control | Control_H-03_2000153943.gpr | 27 | M |
| H-19 | Control | Control_H-19_2000154008.gpr | 18 | F |
| H-23 | Control | Control_H-23_2000154009.gpr | 20 | F |
| H-25 | Control | Control_H-25_2000153952.gpr | 19 | F |
| H-35 | Control | Control_H-35_2000153942.gpr | 20 | F |
| H-41 | Control | Control_H-41_2000154026.gpr | 19 | F |
| H-58 | Control | Control_H-58_2000153935.gpr | NA | NA |
| H-59 | Control | Control_H-59_2000154016.gpr | NA | NA |
| HC-25 | Control | Control_HC-25_2000153932.gpr | NA | NA |
| HV-56 | Control | Control_HV-56_2000155735.gpr | 35 | M |
| HV-59 | Control | Control_HV-59_2000155740.gpr | 42 | M |
| HV-64 | Control | Control_HV-64_2000144456.gpr | 31 | M |
| HV-70 | Control | Control_HV-70_2000144457.gpr | NA | NA |
| HV-71 | Control | Control_HV-71_2000144458.gpr | NA | NA |
| CF 4450 | MGGrade1 | MG_Grade_I_CF 4450_2000153801.gpr | 55 | F |
| CH 24953 | MGGrade1 | MG_Grade_I_CH 24953_2000153787.gpr | 41 | F |
| CJ 4231 | MGGrade1 | MG_Grade_I_CJ 4231_2000153790.gpr | 35 | F |
| CJ 9179 | MGGrade1 | MG_Grade_I_CJ 9179_2000153906.gpr | 57 | F |
| CJ 14742 | MGGrade1 | MG_Grade_I_CJ 14742_2000153789.gpr | 29 | M |
| CJ 20619 | MGGrade1 | MG_Grade_I_CJ 20619_2000153788.gpr | 41 | F |
| CJ 26538 | MGGrade1 | MG_Grade_I_CJ 26538_2000153915.gpr | 45 | F |
| CJ 29452 | MGGrade1 | MG_Grade_I_CJ 29452_2000153786.gpr | 42 | F |
| CJ 29583 | MGGrade1 | MG_Grade_I_CJ 29583_2000153908.gpr | 44 | F |
| CJ 29822 | MGGrade1 | MG_Grade_I_CJ 29822_2000153909.gpr | 50 | F |
| CH 17967 | MGGrade2 | MG_Grade_II_CH 17967_2000153914.gpr | 58 | M |
| CJ 3577 | MGGrade2 | MG_Grade_II_CJ 3577_2000153910.gpr | 43 | F |
| CJ 15491 | MGGrade2 | MG_Grade_II_CJ 15491_2000153803.gpr | 67 | M |
| CJ 15753 | MGGrade2 | MG_Grade_II_CJ 15753_2000153802.gpr | 46 | F |
| CK 7710 | MGGrade2 | MG_Grade_II_CK 7710_2000153907.gpr | 54 | F |

'NA' indicates not availability of age and sex details; 'M': Indicates Male; 'F' indicates Female.

**Supplementary Table 3.2: Shortlisted proteins.** List of proteins with p-value<0.05 and logFC >0.5 or <-0.5 (MGvsHC)

**Supplementary Table 3.1**: Experimental details. The details of the healthy control and Meningioma samples.

| **Table 3Sb: Shortlisted proteins.** List of proteins with p-value<0.05 and logFC >0.5 or <-0.5 | | | | | | |  |  |  |  |  |
| --- | --- | --- | --- | --- | --- | --- | --- | --- | --- | --- | --- |
| **Block** | **Row** | **Column** | **ID** | **Name** | **logFC** | **AveExpr** | **t** | **P.Value** | **adj.P.Val** | **B** | **abslog FC** |
| 41 | 5 | 11 | BC025985.1 | IGHG4 | -3.15 | 8.676 | -32.57 | 8.37E-40 | 1.51E-35 | 71.29 | 3.15 |
| 10 | 18 | 29 | NM_001014444.1 | CRYM | -1.44 | 7.793 | -24.36 | 9.04E-33 | 8.17E-29 | 59.38 | 1.44 |
| 1 | 14 | 17 | NM_171830.1 | KCNMB3 | -0.74 | 7.526 | -12.02 | 1.32E-17 | 7.96E-14 | 29.10 | 0.74 |
| 3 | 3 | 21 | NM_032328.1 | EFCAB2 | 1.08 | 9.673 | 9.68 | 7.32E-14 | 3.30E-10 | 21.05 | 1.08 |
| 3 | 4 | 19 | NM_019102.2 | HOXA5 | -0.77 | 7.622 | -9.16 | 5.42E-13 | 1.95E-09 | 19.16 | 0.77 |
| 27 | 13 | 15 | BC065370.1 | C20orf112 | -2.01 | 9.126 | -9.11 | 6.47E-13 | 1.95E-09 | 18.99 | 2.01 |
| 43 | 1 | 27 | NM_006857.1 | RY1 | 0.88 | 9.307 | 8.90 | 1.50E-12 | 3.87E-09 | 18.19 | 0.88 |
| 27 | 21 | 1 | BC037876.1 | C17orf57 | -1.61 | 8.240 | -8.36 | 1.23E-11 | 2.22E-08 | 16.20 | 1.61 |
| 27 | 22 | 1 | BC073856.1 | ADRB2 | -0.59 | 7.429 | -8.32 | 1.41E-11 | 2.31E-08 | 16.07 | 0.59 |
| 23 | 15 | 29 | NM_003153.3 | STAT6 | -0.60 | 7.383 | -8.30 | 1.54E-11 | 2.31E-08 | 15.99 | 0.60 |
| 27 | 3 | 21 | BC006453.1 | HDAC7A | 0.96 | 8.076 | 7.89 | 7.74E-11 | 1.08E-07 | 14.45 | 0.96 |
| 39 | 6 | 27 | NM_001042476.1 | CARHSP1 | -0.97 | 8.050 | -7.78 | 1.18E-10 | 1.52E-07 | 14.06 | 0.97 |
| 36 | 1 | 17 | NM_014814.1 | PSMD6 | 0.55 | 7.822 | 7.75 | 1.31E-10 | 1.56E-07 | 13.96 | 0.55 |
| 6 | 7 | 21 | BC033854.1 | GSG1 | 0.77 | 8.869 | 7.74 | 1.38E-10 | 1.56E-07 | 13.90 | 0.77 |
| 27 | 22 | 17 | NM_001033515.1 | LOC389833 | -1.12 | 8.321 | -7.72 | 1.49E-10 | 1.58E-07 | 13.83 | 1.12 |
| 36 | 3 | 13 | NM_004147.3 | DRG1 | 0.53 | 7.652 | 7.68 | 1.71E-10 | 1.63E-07 | 13.70 | 0.53 |
| 29 | 6 | 31 | NM_004264.2 | SURB7 | -0.98 | 7.641 | -7.62 | 2.17E-10 | 1.96E-07 | 13.48 | 0.98 |
| 11 | 1 | 19 | NM_031966.2 | CCNB1 | 0.85 | 8.988 | 7.59 | 2.48E-10 | 2.14E-07 | 13.35 | 0.85 |
| 10 | 21 | 5 | BC062437.1 | COX4I1 | -0.56 | 7.627 | -7.53 | 3.20E-10 | 2.46E-07 | 13.11 | 0.56 |
| 38 | 18 | 5 | NM_031304.2 | DOHH | -0.79 | 10.057 | -7.52 | 3.27E-10 | 2.46E-07 | 13.08 | 0.79 |
| 38 | 1 | 19 | NM_182476.1 | COQ6 | 0.54 | 7.633 | 7.41 | 5.05E-10 | 3.38E-07 | 12.67 | 0.54 |
| 21 | 18 | 15 | NM_005719.2 | ARPC3 | -1.16 | 8.351 | -7.39 | 5.36E-10 | 3.46E-07 | 12.61 | 1.16 |
| 16 | 2 | 1 | NM_007198.2 | PROSC | 0.75 | 8.519 | 7.32 | 7.17E-10 | 4.18E-07 | 12.34 | 0.75 |
| 26 | 13 | 9 | NM_007280.1 | OIP5 | 0.70 | 8.831 | 7.17 | 1.28E-09 | 6.98E-07 | 11.79 | 0.70 |
| 22 | 3 | 5 | BC026107.2 | KRR1 | 0.62 | 8.579 | 7.10 | 1.70E-09 | 8.76E-07 | 11.52 | 0.62 |
| 3 | 13 | 9 | NM_013409.1 | FST | 0.61 | 9.641 | 7.07 | 1.92E-09 | 9.62E-07 | 11.40 | 0.61 |
| 3 | 3 | 11 | NM_001001394.2 | HCG3 | 0.75 | 9.068 | 7.01 | 2.39E-09 | 1.10E-06 | 11.19 | 0.75 |
| 11 | 1 | 9 | NM_002867.2 | RAB3B | 0.79 | 9.261 | 6.88 | 4.07E-09 | 1.84E-06 | 10.69 | 0.79 |
| 44 | 3 | 9 | BC013019.2 | CCDC28A | 0.54 | 8.904 | 6.80 | 5.42E-09 | 2.28E-06 | 10.41 | 0.54 |
| 38 | 3 | 5 | NM_014078.4 | MRPL13 | 0.70 | 7.784 | 6.70 | 8.21E-09 | 3.37E-06 | 10.02 | 0.70 |
| 15 | 3 | 27 | BC004130.2 | CALCOCO2 | 0.65 | 9.362 | 6.67 | 9.10E-09 | 3.65E-06 | 9.92 | 0.65 |
| 38 | 20 | 13 | BC068569.1 | ZHX3 | -0.75 | 9.233 | -6.64 | 1.04E-08 | 4.09E-06 | 9.79 | 0.75 |
| 25 | 23 | 17 | BC110374 | CORO1A | -0.74 | 7.658 | -6.63 | 1.09E-08 | 4.11E-06 | 9.75 | 0.74 |
| 27 | 1 | 27 | BC017864.1 | GYPE | 0.69 | 7.959 | 6.57 | 1.35E-08 | 4.99E-06 | 9.54 | 0.69 |
| 24 | 1 | 7 | NM_007236.3 | CHP | 0.55 | 7.751 | 6.46 | 2.08E-08 | 7.08E-06 | 9.13 | 0.55 |
| 38 | 19 | 9 | NM_002257.2 | KLK1 | -0.75 | 8.266 | -6.46 | 2.12E-08 | 7.08E-06 | 9.12 | 0.75 |
| 19 | 9 | 19 | ENST00000435033 | NA | 0.56 | 9.268 | 6.37 | 2.92E-08 | 9.25E-06 | 8.81 | 0.56 |
| 38 | 18 | 17 | NM_012266.3 | DNAJB5 | -0.52 | 8.192 | -6.33 | 3.42E-08 | 1.03E-05 | 8.66 | 0.52 |
| 13 | 22 | 9 | BC064383.1 | SLC39A9 | -0.54 | 7.683 | -6.30 | 3.82E-08 | 1.12E-05 | 8.56 | 0.54 |
| 36 | 1 | 15 | BC013992.1 | MAPK3 | 0.91 | 9.260 | 6.30 | 3.86E-08 | 1.12E-05 | 8.55 | 0.91 |
| 27 | 1 | 25 | NM_015971.2 | MRPS7 | 0.58 | 7.939 | 6.20 | 5.68E-08 | 1.58E-05 | 8.18 | 0.58 |
| 38 | 20 | 29 | NM_080820.3 | DTD1 | -0.51 | 7.556 | -6.20 | 5.70E-08 | 1.58E-05 | 8.17 | 0.51 |
| 15 | 1 | 13 | NM_002149.2 | HPCAL1 | 0.78 | 9.111 | 6.17 | 6.39E-08 | 1.72E-05 | 8.07 | 0.78 |
| 14 | 1 | 17 | NM_022107.1 | GPSM3 | 0.68 | 8.712 | 6.11 | 8.23E-08 | 2.10E-05 | 7.82 | 0.68 |
| 11 | 1 | 27 | NM_001381.2 | DOK1 | 0.59 | 9.103 | 6.05 | 1.01E-07 | 2.49E-05 | 7.63 | 0.59 |
| 38 | 20 | 27 | NM_025203.1 | C2orf44 | -0.58 | 8.099 | -6.04 | 1.05E-07 | 2.49E-05 | 7.60 | 0.58 |
| 40 | 7 | 13 | BC015738.1 | ZFYVE19 | 0.63 | 8.915 | 5.97 | 1.41E-07 | 3.13E-05 | 7.32 | 0.63 |
| 34 | 3 | 5 | NM_003732.2 | EIF4EBP3 | 0.62 | 9.512 | 5.94 | 1.55E-07 | 3.33E-05 | 7.22 | 0.62 |
| 38 | 21 | 1 | BC066896.1 | KARCA1 | -0.51 | 7.966 | -5.93 | 1.61E-07 | 3.41E-05 | 7.19 | 0.51 |
| 27 | 14 | 19 | NM_002767.2 | PRPSAP2 | -1.20 | 8.555 | -5.92 | 1.67E-07 | 3.44E-05 | 7.15 | 1.20 |
| 12 | 1 | 15 | NM_001827.1 | CKS2 | 0.79 | 8.339 | 5.92 | 1.69E-07 | 3.44E-05 | 7.14 | 0.79 |
| 18 | 3 | 17 | NM_024770.1 | METTL8 | 0.51 | 8.051 | 5.91 | 1.71E-07 | 3.44E-05 | 7.13 | 0.51 |
| 27 | 12 | 25 | NM_001005465.1 | OR10G3 | -1.30 | 10.012 | -5.88 | 1.94E-07 | 3.76E-05 | 7.01 | 1.30 |
| 11 | 2 | 1 | BC010405.2 | TSPAN17 | 0.50 | 8.353 | 5.88 | 1.98E-07 | 3.76E-05 | 6.99 | 0.50 |
| 16 | 3 | 25 | BC007097.1 | TIMP1 | 0.51 | 8.040 | 5.87 | 2.01E-07 | 3.76E-05 | 6.97 | 0.51 |
| 12 | 3 | 1 | NM_002125.3 | HLA-DRB5 | 0.69 | 7.778 | 5.77 | 3.03E-07 | 5.22E-05 | 6.58 | 0.69 |
| 11 | 1 | 13 | BC001360.2 | RHOA | 0.66 | 8.875 | 5.76 | 3.12E-07 | 5.32E-05 | 6.56 | 0.66 |
| 24 | 1 | 15 | NM_021131.3 | PPP2R4 | 0.64 | 8.189 | 5.75 | 3.21E-07 | 5.42E-05 | 6.53 | 0.64 |
| 14 | 20 | 19 | NM_015726.2 | WDR42A | -0.83 | 8.716 | -5.73 | 3.49E-07 | 5.83E-05 | 6.45 | 0.83 |
| 8 | 16 | 11 | NM_139204.1 | EPS8L1 | 1.25 | 8.431 | 5.72 | 3.59E-07 | 5.96E-05 | 6.42 | 1.25 |
| 26 | 3 | 23 | BC008369.1 | STAU2 | 0.58 | 7.704 | 5.69 | 4.05E-07 | 6.41E-05 | 6.31 | 0.58 |
| 35 | 15 | 13 | NM_001025266.1 | LOC285382 | 1.37 | 8.631 | 5.64 | 4.81E-07 | 7.12E-05 | 6.15 | 1.37 |
| 5 | 3 | 27 | BC001103.2 | GULP1 | 0.53 | 9.151 | 5.62 | 5.23E-07 | 7.38E-05 | 6.07 | 0.53 |
| 26 | 3 | 1 | BC011757.2 | GADD45A | 0.51 | 8.701 | 5.62 | 5.31E-07 | 7.38E-05 | 6.05 | 0.51 |
| 27 | 2 | 15 | NM_014372.3 | RNF11 | -1.26 | 8.585 | -5.57 | 6.28E-07 | 8.40E-05 | 5.89 | 1.26 |
| 26 | 23 | 17 | Dlx5 | DLX5 | -0.58 | 7.830 | -5.51 | 7.99E-07 | 9.82E-05 | 5.66 | 0.58 |
| 23 | 1 | 17 | NM_017614.3 | BHMT2 | 0.66 | 8.109 | 5.45 | 9.89E-07 | 0.000117493 | 5.46 | 0.66 |
| 20 | 10 | 3 | BC000870.1 | TIPIN | 0.65 | 8.995 | 5.45 | 1.02E-06 | 0.000119987 | 5.44 | 0.65 |
| 7 | 13 | 9 | NM_002297.2 | LCN1 | 0.59 | 9.512 | 5.42 | 1.13E-06 | 0.00012651 | 5.34 | 0.59 |
| 5 | 1 | 15 | NM_001549.2 | IFIT3 | 0.66 | 9.131 | 5.38 | 1.29E-06 | 0.000139106 | 5.21 | 0.66 |
| 20 | 1 | 27 | BC008253.1 | C8orf43 | 0.67 | 8.390 | 5.37 | 1.37E-06 | 0.000143366 | 5.15 | 0.67 |
| 27 | 23 | 25 | BC106934 | CCT4 | -0.55 | 7.440 | -5.34 | 1.48E-06 | 0.000151699 | 5.08 | 0.55 |
| 30 | 3 | 21 | BC006011.1 | DDI2 | 0.52 | 8.789 | 5.33 | 1.55E-06 | 0.000154276 | 5.04 | 0.52 |
| 25 | 1 | 21 | NM_004359.1 | CDC34 | 0.63 | 8.465 | 5.33 | 1.58E-06 | 0.000154787 | 5.02 | 0.63 |
| 22 | 3 | 7 | BC027956.1 | SULT1E1 | 0.58 | 8.650 | 5.32 | 1.61E-06 | 0.000155964 | 5.00 | 0.58 |
| 11 | 24 | 19 | BC029498.1 | LTC4S | -0.51 | 7.999 | -5.30 | 1.78E-06 | 0.000170792 | 4.91 | 0.51 |
| 25 | 1 | 7 | NM_020677.2 | NMRAL1 | 0.66 | 8.102 | 5.29 | 1.81E-06 | 0.000172562 | 4.89 | 0.66 |
| 38 | 18 | 7 | NM_021979.2 | HSPA2 | -0.55 | 9.299 | -5.27 | 1.96E-06 | 0.000182139 | 4.81 | 0.55 |
| 46 | 21 | 9 | BC036124.1 | MIPOL1 | -0.62 | 8.130 | -5.27 | 1.97E-06 | 0.000182139 | 4.81 | 0.62 |
| 10 | 2 | 19 | BC015045.2 | GALT | 0.56 | 7.694 | 5.22 | 2.33E-06 | 0.000204097 | 4.65 | 0.56 |
| 27 | 14 | 17 | NM_148910.2 | TIRAP | -1.09 | 8.429 | -5.22 | 2.35E-06 | 0.000204097 | 4.64 | 1.09 |
| 40 | 5 | 27 | BC020637.1 | IL1F7 | 0.56 | 8.999 | 5.21 | 2.46E-06 | 0.000206767 | 4.60 | 0.56 |
| 25 | 13 | 9 | BC002448.2 | ABLIM1 | 0.58 | 8.383 | 5.20 | 2.52E-06 | 0.000210952 | 4.57 | 0.58 |
| 27 | 21 | 7 | NM_001039656.1 | MTL5 | -0.97 | 7.811 | -5.18 | 2.78E-06 | 0.000228695 | 4.48 | 0.97 |
| 3 | 19 | 5 | NM_003690.3 | PRKRA | -0.56 | 7.938 | -5.15 | 3.09E-06 | 0.000248339 | 4.38 | 0.56 |
| 40 | 3 | 21 | NM_182789.2 | PAIP1 | 0.71 | 9.130 | 5.11 | 3.54E-06 | 0.000269837 | 4.25 | 0.71 |
| 27 | 23 | 21 | BC109063 | TRIM68 | -0.53 | 7.415 | -5.09 | 3.78E-06 | 0.000278909 | 4.19 | 0.53 |
| 6 | 1 | 13 | NM_001744.3 | CAMK4 | 0.54 | 9.038 | 5.08 | 3.98E-06 | 0.000285163 | 4.14 | 0.54 |
| 27 | 20 | 9 | NM_003359.2 | UGDH | -0.82 | 7.751 | -5.06 | 4.19E-06 | 0.000297432 | 4.09 | 0.82 |
| 4 | 1 | 23 | NM_001007226.1 | SPOP | 0.51 | 7.994 | 5.05 | 4.44E-06 | 0.000309092 | 4.04 | 0.51 |
| 1 | 3 | 31 | BC007520.1 | CAPRIN2 | 0.54 | 8.048 | 5.05 | 4.45E-06 | 0.000309092 | 4.04 | 0.54 |
| 30 | 2 | 25 | NM_001025436.1 | SPAG16 | 0.66 | 8.507 | 5.04 | 4.61E-06 | 0.000316003 | 4.00 | 0.66 |
| 47 | 2 | 21 | NM_001031677.2 | RAB24 | 0.53 | 8.995 | 5.01 | 5.17E-06 | 0.000343066 | 3.90 | 0.53 |
| 11 | 13 | 9 | NM_000884.2 | IMPDH2 | 0.54 | 8.802 | 4.99 | 5.49E-06 | 0.000355016 | 3.84 | 0.54 |
| 31 | 6 | 29 | NM_030920.2 | ANP32E | 0.53 | 8.290 | 4.97 | 5.95E-06 | 0.000366273 | 3.76 | 0.53 |
| 43 | 3 | 11 | NM_003350.2 | UBE2V2 | 0.52 | 8.639 | 4.96 | 6.12E-06 | 0.000371362 | 3.74 | 0.52 |
| 40 | 1 | 29 | NM_018147.2 | FAIM | 0.51 | 8.574 | 4.88 | 8.28E-06 | 0.00047017 | 3.45 | 0.51 |
| 42 | 3 | 7 | NM_000184.2 | HBG2 | 0.70 | 9.361 | 4.87 | 8.42E-06 | 0.000476628 | 3.43 | 0.70 |
| 4 | 3 | 5 | NM_022488.3 | ATG3 | 0.52 | 7.816 | 4.85 | 9.03E-06 | 0.000500303 | 3.37 | 0.52 |
| 27 | 15 | 29 | NM_198086.1 | JUB | -1.30 | 8.843 | -4.83 | 9.79E-06 | 0.000530996 | 3.29 | 1.30 |
| 27 | 3 | 11 | NM_004362.1 | CLGN | 0.52 | 7.703 | 4.82 | 1.02E-05 | 0.00054291 | 3.25 | 0.52 |
| 23 | 1 | 13 | BC001550.1 | SFN | 0.56 | 8.291 | 4.80 | 1.10E-05 | 0.00057175 | 3.19 | 0.56 |
| 27 | 20 | 1 | NM_003597.4 | KLF11 | -0.66 | 7.634 | -4.79 | 1.14E-05 | 0.000586729 | 3.15 | 0.66 |
| 33 | 1 | 11 | BC011913.2 | P2RX7 | 0.50 | 8.274 | 4.78 | 1.19E-05 | 0.000605315 | 3.10 | 0.50 |
| 5 | 20 | 29 | BC080607.1 | TMEM185B | -0.57 | 7.880 | -4.75 | 1.29E-05 | 0.000638103 | 3.03 | 0.57 |
| 8 | 3 | 3 | BC027877.1 | TEAD3 | 0.54 | 9.626 | 4.73 | 1.39E-05 | 0.000672148 | 2.96 | 0.54 |
| 6 | 11 | 3 | BC014258.1 | IGHG1 | 0.56 | 9.619 | 4.71 | 1.52E-05 | 0.000719969 | 2.88 | 0.56 |
| 20 | 2 | 31 | BC007566.1 | M6PRBP1 | 0.51 | 8.680 | 4.69 | 1.60E-05 | 0.00074757 | 2.83 | 0.51 |
| 8 | 1 | 25 | NM_031468.2 | CALN1 | 0.55 | 8.585 | 4.67 | 1.74E-05 | 0.000783769 | 2.75 | 0.55 |
| 14 | 2 | 1 | NM_014034.1 | ASF1A | 0.54 | 8.843 | 4.67 | 1.77E-05 | 0.000791789 | 2.74 | 0.54 |
| 27 | 18 | 11 | NM_004663.3 | RAB11A | -0.62 | 8.160 | -4.66 | 1.78E-05 | 0.000795519 | 2.73 | 0.62 |
| 36 | 4 | 17 | NM_003099.3 | SNX1 | 0.96 | 9.411 | 4.66 | 1.84E-05 | 0.000817213 | 2.70 | 0.96 |
| 26 | 19 | 17 | NM_018379.3 | FAM63A | -0.66 | 8.616 | -4.63 | 2.01E-05 | 0.000860842 | 2.61 | 0.66 |
| 27 | 15 | 13 | NM_002893.2 | RBBP7 | -0.96 | 8.123 | -4.61 | 2.15E-05 | 0.000899981 | 2.55 | 0.96 |
| 27 | 18 | 13 | BC028026.1 | PCGF3 | -0.72 | 7.791 | -4.61 | 2.19E-05 | 0.000911881 | 2.53 | 0.72 |
| 25 | 2 | 29 | BC020233 | CKAP2 | 0.50 | 8.228 | 4.58 | 2.41461E-05 | 0.000978356 | 2.44 | 0.50 |
| 14 | 3 | 11 | BC027870.1 | PDLIM3 | 0.56 | 9.343 | 4.56 | 2.54E-05 | 0.001013945 | 2.39 | 0.56 |
| 27 | 15 | 7 | XM_290842.4 | LRFN1 | -1.14 | 8.326 | -4.55 | 2.66E-05 | 0.001050001 | 2.35 | 1.14 |
| 26 | 22 | 19 | BC066915.1 | MARCKSL1 | -0.62 | 7.415 | -4.53 | 2.84E-05 | 0.00110008 | 2.29 | 0.62 |
| 31 | 6 | 23 | NM_017924.2 | C14orf119 | 0.51 | 9.199 | 4.49 | 3.26E-05 | 0.001209322 | 2.16 | 0.51 |
| 33 | 15 | 25 | BC007706.2 | FAM105B | -0.63 | 7.910 | -4.43 | 4.03E-05 | 0.001391074 | 1.96 | 0.63 |
| 38 | 20 | 25 | NM_001801.2 | CDO1 | -0.60 | 9.269 | -4.43 | 4.07E-05 | 0.001397189 | 1.95 | 0.60 |
| 46 | 21 | 27 | BC098132.1 | CCR10 | -0.56 | 8.069 | -4.42 | 4.15E-05 | 0.001408809 | 1.93 | 0.56 |
| 40 | 16 | 9 | NM_032955.1 | AIF1 | 0.50 | 9.538 | 4.42 | 4.17E-05 | 0.001409394 | 1.93 | 0.50 |
| 12 | 2 | 9 | NM_052838.2 | SEPT1 | 0.56 | 7.960 | 4.40 | 4.47E-05 | 0.001476956 | 1.86 | 0.56 |
| 27 | 20 | 11 | NM_021810.3 | CDH26 | -1.01 | 8.487 | -4.40 | 4.56E-05 | 0.001499112 | 1.84 | 1.01 |
| 8 | 1 | 7 | NM_021135.4 | RPS6KA2 | 0.52 | 7.515 | 4.39 | 4.70E-05 | 0.001527058 | 1.82 | 0.52 |
| 27 | 20 | 21 | NM_173663.1 | NY-REN-7 | -0.75 | 7.890 | -4.39 | 4.73E-05 | 0.001531184 | 1.81 | 0.75 |
| 1 | 1 | 21 | NM_012106.3 | ARL2BP | 0.67 | 8.811 | 4.37 | 5.01E-05 | 0.001603851 | 1.76 | 0.67 |
| 23 | 21 | 25 | BC090880.1 | EIF3S3 | -1.11 | 8.291 | -4.37 | 5.02E-05 | 0.001603851 | 1.75 | 1.11 |
| 15 | 1 | 11 | NM_016049.3 | C14orf122 | 0.58 | 9.276 | 4.36 | 5.23E-05 | 0.001649492 | 1.71 | 0.58 |
| 4 | 1 | 7 | BC000293.2 | NME1 | 0.55 | 7.940 | 4.32 | 6.07E-05 | 0.001831889 | 1.58 | 0.55 |
| 28 | 13 | 9 | NM_173608.1 | C14orf80 | 0.52 | 8.500 | 4.31 | 6.09E-05 | 0.001835052 | 1.57 | 0.52 |
| 38 | 21 | 31 | BC098334.1 | RAP1GDS1 | -0.55 | 8.867 | -4.31 | 6.20E-05 | 0.001852525 | 1.56 | 0.55 |
| 31 | 1 | 3 | NM_016185.2 | HN1 | 0.54 | 8.030 | 4.31 | 6.27E-05 | 0.001863382 | 1.54 | 0.54 |
| 12 | 1 | 9 | NM_006098.4 | GNB2L1 | 0.54 | 7.954 | 4.28 | 6.76E-05 | 0.001942117 | 1.47 | 0.54 |
| 34 | 20 | 11 | NM_022158.2 | FN3K | -0.52 | 8.989 | -4.27 | 6.97E-05 | 0.00195886 | 1.45 | 0.52 |
| 38 | 20 | 11 | NM_003385.4 | VSNL1 | -0.54 | 10.623 | -4.23 | 8.15E-05 | 0.002193808 | 1.30 | 0.54 |
| 47 | 1 | 19 | BC009253.2 | ABCF3 | 0.57 | 8.413 | 4.19 | 9.46E-05 | 0.002386098 | 1.16 | 0.57 |
| 12 | 1 | 21 | BC001023.2 | HLA-DRB3 | 0.57 | 7.492 | 4.17 | 9.82513E-05 | 0.002453453 | 1.12 | 0.57 |
| 1 | 2 | 3 | NM_015433.2 | FAM119B | 0.54 | 9.234 | 4.16 | 0.000102515 | 0.002526522 | 1.08 | 0.54 |
| 4 | 1 | 31 | NM_080653.3 | ATP6V1E2 | 0.53 | 7.985 | 4.15 | 0.000106027 | 0.002581383 | 1.05 | 0.53 |
| 47 | 9 | 9 | NM_004537.3 | NAP1L1 | -0.62 | 9.859 | -4.15 | 0.000107545 | 0.002601735 | 1.04 | 0.62 |
| 17 | 2 | 1 | NM_032331.2 | MGC2408 | 0.65 | 10.204 | 4.14 | 0.000108645 | 0.002612758 | 1.03 | 0.65 |
| 27 | 24 | 25 | NM_022782.2 | MPHOSPH9 | -0.52 | 7.412 | -4.11 | 0.000123668 | 0.0028581 | 0.91 | 0.52 |
| 17 | 2 | 5 | NM_001444.1 | FABP5 | 0.63 | 10.175 | 4.10 | 0.000126498 | 0.002888992 | 0.89 | 0.63 |
| 44 | 7 | 13 | NM_001157.2 | ANXA11 | 0.80 | 10.190 | 4.10 | 0.000128169 | 0.002912429 | 0.88 | 0.80 |
| 27 | 21 | 31 | NM_001003892.1 | DUPD1 | -0.95 | 7.999 | -4.09 | 0.000130028 | 0.002942651 | 0.86 | 0.95 |
| 28 | 15 | 31 | NM_173809.2 | BLOC1S2 | -1.72 | 9.020 | -4.09 | 0.000130151 | 0.002942651 | 0.86 | 1.72 |
| 38 | 14 | 29 | BC001487.2 | TARDBP | -0.53 | 8.918 | -4.09 | 0.000132754 | 0.00298202 | 0.84 | 0.53 |
| 47 | 20 | 11 | NM_003314.1 | TTC1 | -0.63 | 9.839 | -4.07 | 0.000137519 | 0.003051949 | 0.81 | 0.63 |
| 22 | 3 | 3 | NM_000945.3 | PPP3R1 | 0.60 | 10.437 | 4.05 | 0.000147659 | 0.003237218 | 0.74 | 0.60 |
| 46 | 15 | 25 | NM_176823.2 | S100A7A | -0.77 | 7.451 | -3.98 | 0.000191712 | 0.003865261 | 0.50 | 0.77 |
| 47 | 12 | 27 | BC036107.1 | HSPA2 | -0.53 | 9.857 | -3.97 | 0.000196691 | 0.003917562 | 0.48 | 0.53 |
| 39 | 1 | 31 | BC013009.2 | ZMYM3 | 0.57 | 10.122 | 3.96 | 0.00020075 | 0.003972123 | 0.46 | 0.57 |
| 11 | 17 | 7 | NM_022562.2 | GH1 | 0.51 | 8.680 | 3.94 | 0.000212644 | 0.00414392 | 0.40 | 0.51 |
| 9 | 2 | 25 | NM_006360.3 | PCID1 | 0.56 | 9.902 | 3.94 | 0.000216682 | 0.004195452 | 0.39 | 0.56 |
| 38 | 11 | 5 | BC012142.1 | ATP6V1C2 | -0.52 | 8.361 | -3.93 | 0.000225193 | 0.004300541 | 0.35 | 0.52 |
| 16 | 19 | 21 | NM_016224.3 | SNX9 | -1.13 | 7.660 | -3.88 | 0.000259839 | 0.004775173 | 0.22 | 1.13 |
| 27 | 17 | 3 | ENST00000369196 | NA | -0.57 | 7.682 | -3.88 | 0.000260485 | 0.004775335 | 0.21 | 0.57 |
| 38 | 24 | 19 | BC029896.1 | SIGLEC5 | -0.51 | 8.696 | -3.87 | 0.000267027 | 0.004872575 | 0.19 | 0.51 |
| 39 | 16 | 5 | NM_205840.1 | LST1 | -0.71 | 7.439 | -3.86 | 0.000282711 | 0.005086826 | 0.14 | 0.71 |
| 46 | 20 | 31 | NM_031431.2 | COG3 | -0.54 | 7.352 | -3.86 | 0.000283331 | 0.005087853 | 0.14 | 0.54 |
| 15 | 3 | 21 | NM_016172.2 | UBADC1 | 0.55 | 8.079 | 3.84 | 0.000297521 | 0.005238523 | 0.09 | 0.55 |
| 33 | 1 | 13 | NM_022777.1 | RABL5 | 0.52 | 9.670 | 3.83 | 0.00030948 | 0.005388004 | 0.05 | 0.52 |
| 27 | 19 | 25 | NM_001001552.3 | LEMD1 | -0.84 | 7.860 | -3.83 | 0.000312756 | 0.005427406 | 0.04 | 0.84 |
| 36 | 23 | 23 | Lhx1 | Lhx1 | -1.01 | 8.053 | -3.82 | 0.000314844 | 0.005447945 | 0.04 | 1.01 |
| 33 | 1 | 3 | NM_003130.2 | SRI | 0.51 | 8.560 | 3.82 | 0.000317751 | 0.005474472 | 0.03 | 0.51 |
| 4 | 1 | 9 | NM_003404.3 | YWHAB | 0.51 | 7.872 | 3.81 | 0.000327508 | 0.005578863 | 0.00 | 0.51 |
| 34 | 23 | 9 | NM_001155.3 | ANXA6 | -0.51 | 8.979 | -3.72 | 0.000436859 | 0.00671648 | -0.27 | 0.51 |
| 20 | 22 | 23 | BC075842.1 | IGHG1 | 0.56 | 10.629 | 3.71 | 0.000461581 | 0.006942929 | -0.32 | 0.56 |
| 25 | 6 | 19 | NM_003288.2 | TPD52L2 | 0.53 | 9.024 | 3.65 | 0.000552289 | 0.007868376 | -0.48 | 0.53 |
| 36 | 23 | 21 | Nol3 | Nol3 | -1.39 | 11.320 | -3.64 | 0.000563246 | 0.007995503 | -0.50 | 1.39 |
| 40 | 22 | 7 | BC068547.1 | SRPK2 | -0.51 | 8.174 | -3.64 | 0.000578767 | 0.008142848 | -0.53 | 0.51 |
| 27 | 18 | 31 | NM_006541.1 | TXNL2 | -0.71 | 8.268 | -3.60 | 0.000646999 | 0.008821154 | -0.63 | 0.71 |
| 46 | 23 | 31 | BC000446 | CLP1 | -0.55 | 9.636 | -3.58 | 0.000678484 | 0.009082664 | -0.67 | 0.55 |
| 25 | 1 | 27 | ENST00000312635 | USP15 | 0.61 | 8.422 | 3.57 | 0.000720305 | 0.009429212 | -0.73 | 0.61 |
| 27 | 17 | 29 | NM_153478.1 | CSAG1 | -0.57 | 7.538 | -3.55 | 0.000758049 | 0.009767588 | -0.77 | 0.57 |
| 41 | 18 | 21 | NM_002664.1 | PLEK | -0.54 | 9.203 | -3.54 | 0.000770421 | 0.00985669 | -0.79 | 0.54 |
| 38 | 13 | 11 | NM_198204.1 | MLX | -0.65 | 9.421 | -3.54 | 0.000774894 | 0.009892902 | -0.80 | 0.65 |
| 12 | 21 | 17 | BC103692.1 | FMN1 | -0.74 | 8.899 | -3.54 | 0.000779647 | 0.009925532 | -0.80 | 0.74 |
| 12 | 22 | 5 | BC075800.1 | PRKAR2B | -0.60 | 9.276 | -3.51 | 0.000848505 | 0.010581909 | -0.88 | 0.60 |
| 30 | 21 | 27 | NM_001106.3 | ACVR2B | -0.64 | 7.759 | -3.45 | 0.001048574 | 0.012088379 | -1.07 | 0.64 |
| 42 | 15 | 31 | BC067254.1 | COASY | -0.54 | 9.536 | -3.40 | 0.001218039 | 0.013591028 | -1.21 | 0.54 |
| 27 | 14 | 21 | NM_147196.1 | TMIE | -0.55 | 7.541 | -3.39 | 0.001238414 | 0.013767223 | -1.23 | 0.55 |
| 19 | 1 | 21 | NM_013368.2 | SERTAD3 | 0.54 | 8.724 | 3.36 | 0.001354172 | 0.014763501 | -1.31 | 0.54 |
| 43 | 20 | 23 | BC094878.1 | ARL2BP | -0.52 | 10.540 | -3.34 | 0.001434934 | 0.015338509 | -1.36 | 0.52 |
| 27 | 12 | 27 | NM_199337.1 | LOC374395 | -0.84 | 8.316 | -3.34 | 0.001465095 | 0.015587127 | -1.38 | 0.84 |
| 27 | 14 | 23 | NM_018584.4 | CAMK2N1 | -0.83 | 9.363 | -3.30 | 0.001647557 | 0.016843868 | -1.49 | 0.83 |
| 46 | 17 | 27 | NM_001009956.1 | ZNF655 | -0.52 | 9.766 | -3.29 | 0.001699564 | 0.017152938 | -1.52 | 0.52 |
| 25 | 23 | 13 | NM_138362.1 | FAM104B | -0.50 | 8.540 | -3.29 | 0.001699627 | 0.017152938 | -1.52 | 0.50 |
| 12 | 20 | 23 | NM_181713.3 | UBXD4 | -0.55 | 9.106 | -3.27 | 0.00175885 | 0.017585926 | -1.55 | 0.55 |
| 46 | 19 | 9 | NM_001008491.1 | SEPT2 | -0.52 | 10.010 | -3.25 | 0.001894515 | 0.018528607 | -1.62 | 0.52 |
| 27 | 15 | 21 | NM_005678.3 | SNURF | -0.56 | 7.642 | -3.24 | 0.001932071 | 0.018764985 | -1.63 | 0.56 |
| 27 | 24 | 31 | BC009388.1 | LOC554174 | -0.60 | 7.963 | -3.19 | 0.002257188 | 0.020751196 | -1.78 | 0.60 |
| 27 | 20 | 27 | BC082258.1 | RP11-56A21.1 | -0.71 | 7.661 | -3.08 | 0.003097285 | 0.026039981 | -2.06 | 0.71 |
| 33 | 2 | 17 | BC018747.1 | IGHG1 | 0.80 | 9.996 | 3.08 | 0.003123215 | 0.026191366 | -2.07 | 0.80 |
| 46 | 11 | 31 | BC013426.1 | PLEKHG2 | -0.80 | 9.032 | -3.04 | 0.00355486 | 0.028592408 | -2.19 | 0.80 |
| 15 | 6 | 31 | NM_001033112.1 | PAIP2 | 0.64 | 9.637 | 2.96 | 0.004419206 | 0.033130599 | -2.38 | 0.64 |
| 46 | 20 | 29 | NM_020532.4 | RTN4 | -0.53 | 7.413 | -2.90 | 0.005216796 | 0.037030029 | -2.53 | 0.53 |
| 39 | 22 | 17 | BC012098.1 | GBE1 | -0.50 | 10.037 | -2.89 | 0.005304928 | 0.037464236 | -2.55 | 0.50 |
| 48 | 1 | 19 | NM_178815.3 | ARL5B | 0.79 | 8.401 | 2.85 | 0.006015628 | 0.041210586 | -2.66 | 0.79 |
| 11 | 6 | 5 | BC002769.1 | C20orf43 | 0.53 | 9.675 | 2.81 | 0.006595709 | 0.04400288 | -2.74 | 0.53 |

**Supplementary Table 3.3: Shortlisted proteins.** List of proteins with p-value<0.05 and logFC >0.5 or <-0.5 (MGvsHC)

| **Table 3Sc: Shortlisted proteins.** List of proteins with p-value<0.05 and logFC >1 or <-1 | | | | | |  |  |  |  |  |
| --- | --- | --- | --- | --- | --- | --- | --- | --- | --- | --- |
| **Row** | **Column** | **ID** | **Name** | **logFC** | **AveExpr** | **t** | **P.Value** | **adj.P.Val** | **B** | **abslog FC** |
| 5 | 11 | BC025985.1 | IGHG4 | -3.15 | 8.68 | -32.57 | 8E-40 | 1.51E-35 | 71.29 | 3.15 |
| 18 | 29 | NM_001014444.1 | CRYM | -1.44 | 7.79 | -24.36 | 9E-33 | 8.17E-29 | 59.38 | 1.44 |
| 3 | 21 | NM_032328.1 | EFCAB2 | 1.08 | 9.67 | 9.68 | 7E-14 | 3.30E-10 | 21.05 | 1.08 |
| 13 | 15 | BC065370.1 | C20orf112 | -2.01 | 9.13 | -9.11 | 6E-13 | 1.95E-09 | 18.99 | 2.01 |
| 21 | 1 | BC037876.1 | C17orf57 | -1.61 | 8.24 | -8.36 | 1E-11 | 2.22E-08 | 16.20 | 1.61 |
| 22 | 17 | NM_001033515.1 | LOC389833 | -1.12 | 8.32 | -7.72 | 1E-10 | 1.58E-07 | 13.83 | 1.12 |
| 18 | 15 | NM_005719.2 | ARPC3 | -1.16 | 8.35 | -7.39 | 5E-10 | 3.46E-07 | 12.61 | 1.16 |
| 14 | 19 | NM_002767.2 | PRPSAP2 | -1.20 | 8.56 | -5.92 | 2E-07 | 3.44E-05 | 7.15 | 1.20 |
| 12 | 25 | NM_001005465.1 | OR10G3 | -1.30 | 10.01 | -5.88 | 2E-07 | 3.76E-05 | 7.01 | 1.30 |
| 16 | 11 | NM_139204.1 | EPS8L1 | 1.25 | 8.43 | 5.72 | 4E-07 | 5.96E-05 | 6.42 | 1.25 |
| 15 | 13 | NM_001025266.1 | LOC285382 | 1.37 | 8.63 | 5.64 | 5E-07 | 7.12E-05 | 6.15 | 1.37 |
| 2 | 15 | NM_014372.3 | RNF11 | -1.26 | 8.59 | -5.57 | 6E-07 | 8.40E-05 | 5.89 | 1.26 |
| 14 | 17 | NM_148910.2 | TIRAP | -1.09 | 8.43 | -5.22 | 2E-06 | 0.000204097 | 4.64 | 1.09 |
| 15 | 29 | NM_198086.1 | JUB | -1.30 | 8.84 | -4.83 | 1E-05 | 0.000530996 | 3.29 | 1.30 |
| 15 | 7 | XM_290842.4 | LRFN1 | -1.14 | 8.33 | -4.55 | 3E-05 | 0.001050001 | 2.35 | 1.14 |
| 20 | 11 | NM_021810.3 | CDH26 | -1.01 | 8.49 | -4.40 | 5E-05 | 0.001499112 | 1.84 | 1.01 |
| 21 | 25 | BC090880.1 | EIF3S3 | -1.11 | 8.29 | -4.37 | 5E-05 | 0.001603851 | 1.75 | 1.11 |
| 15 | 31 | NM_173809.2 | BLOC1S2 | -1.72 | 9.02 | -4.09 | 1E-04 | 0.002942651 | 0.86 | 1.72 |
| 19 | 21 | NM_016224.3 | SNX9 | -1.13 | 7.66 | -3.88 | 3E-04 | 0.004775173 | 0.22 | 1.13 |
| 23 | 23 | Lhx1 | Lhx1 | -1.01 | 8.05 | -3.82 | 3E-04 | 0.005447945 | 0.04 | 1.01 |
| 23 | 21 | Nol3 | Nol3 | -1.39 | 11.32 | -3.64 | 6E-04 | 0.007995503 | -0.50 | 1.39 |

**Supplementary Table 3.4: Full extended tables for main Table 1 and Table 2**

| **Table 1: Significantly dysregulated proteins across all comparisons (absolute logFC>1, p<0.05)** | | | | | | | | | | | | |
| --- | --- | --- | --- | --- | --- | --- | --- | --- | --- | --- | --- | --- |
| **HC vs MG** | | | | **HC vs MG1** | | | | | **HC vs MG2** | | | |
| **ID** | **Symbol** | **Name** | **logFC** | **ID** | **Symbol** | | **Name** | **logFC** | **ID** | **Symbol** | **Name** | **logFC** |
| BC025985.1 | IGHG4 | immunoglobulin heavy constant gamma 4 (G4m marker) | -3.15 | BC025985.1 | IGHG4 | immunoglobulin heavy constant gamma 4 (G4m marker) | | -3.15 | BC025985.1 | IGHG4 | immunoglobulin heavy constant gamma 4 (G4m marker) | -3.15 |
| NM_001014444.1 | CRYM | crystallin, mu | -1.44 | NM_001014444.1 | CRYM | crystallin, mu | | -1.44 | NM_001014444.1 | CRYM | crystallin, mu | -1.43 |
| NM_032328.1 | EFCAB2 | EF-hand calcium binding domain 2 | 1.08 | NM_032328.1 | EFCAB2 | EF-hand calcium binding domain 2 | | 1.10 | BC065370.1 | C20orf112 | Chromosome 20 open reading frame 112 | -2.18 |
| BC065370.1 | C20orf112 | Chromosome 20 open reading frame 112 | -2.01 | NM_031304.2 | DOHH | deoxyhypusine hydroxylase/monooxygenase | | -1.01 | NM_032328.1 | EFCAB2 | EF-hand calcium binding domain 2 | 1.06 |
| BC037876.1 | C17orf57 | Chromosome 20 open reading frame 112 | -1.61 | NM_015726.2 | WDR42A | DDB1 and CUL4 associated factor 8 | | -1.31 | NM_005719.2 | ARPC3 | actin related protein 2/3 complex, subunit 3, 21kDa | -1.48 |
| NM_001033515.1 | LOC389833 | EF-hand calcium binding domain 13 | -1.12 | BC065370.1 | C20orf112 | Chromosome 20 open reading frame 112 | | -1.83 | NM_001042476.1 | CARHSP1 | calcium regulated heat stable protein 1, 24kDa | -1.13 |
| NM_005719.2 | ARPC3 | actin related protein 2/3 complex, subunit 3, 21kDa | -1.16 | BC006453.1 | HDAC7A | Histone deacetylase 7 | | 1.00 | BC037876.1 | C17orf57 | EF-hand calcium binding domain 13 | -1.67 |
| NM_002767.2 | PRPSAP2 | phosphoribosyl pyrophosphate synthetase-associated protein 2 | -1.20 | BC037876.1 | C17orf57 | EF-hand calcium binding domain 13 | | -1.55 | NM_004264.2 | SURB7 | Mediator complex subunit 21 | -1.04 |
| NM_001005465.1 | OR10G3 | olfactory receptor, family 10, subfamily G, member 3 | -1.30 | NM_001033515.1 | LOC389833 |  | | -1.14 | NM_182789.2 | PAIP1 | poly(A) binding protein interacting protein 1 | 1.06 |
| NM_139204.1 | EPS8L1 | EPS8-like 1 | 1.25 | NM_001005465.1 | ND |  | | -1.44 | NM_001033515.1 | LOC389833 |  | -1.09 |
| NM_001025266.1 | LOC285382 |  | 1.37 | NM_139204.1 | EPS8L1 | EPS8-like 1 | | 1.22 | BC013992.1 | MAPK3 | mitogen-activated protein kinase 3 | 1.09 |
| NM_014372.3 | RNF11 | ring finger protein 11 | -1.26 | NM_001025266.1 | LOC285382 |  | | 1.32 | NM_002767.2 | PRPSAP2 | phosphoribosyl pyrophosphate synthetase-associated protein 2 | -1.46 |
| NM_148910.2 | TIRAP | toll-interleukin 1 receptor (TIR) domain containing adaptor protein | -1.09 | NM_148910.2 | TIRAP | toll-interleukin 1 receptor (TIR) domain containing adaptor protein | | -1.05 | NM_014372.3 | RNF11 | ring finger protein 11 | -1.53 |
| NM_198086.1 | JUB | Ajuba LIM protein | -1.30 | NM_002893.2 | RBBP7 | retinoblastoma binding protein 7 | | -1.00 | NM_173809.2 | BLOC1S2 | biogenesis of lysosomal organelles complex-1, subunit 2 | -2.60 |
| XM_290842.4 | LRFN1 | leucine rich repeat and fibronectin type III domain containing 1 | -1.14 | NM_021810.3 | CDH26 | cadherin 26 | | -1.04 | NM_139204.1 | EPS8L1 | EPS8-like 1 | 1.29 |
| NM_021810.3 | CDH26 | cadherin 26 | -1.01 | XM_290842.4 | LRFN1 | leucine rich repeat and fibronectin type III domain containing 1 | | -1.05 | NM_001025266.1 | LOC285382 |  | 1.43 |
| BC090880.1 | EIF3S3 | Eukaryotic translation initiation factor 3, subunit H | -1.11 | NM_018584.4 | CAMK2N1 | calcium/calmodulin-dependent protein kinase II inhibitor 1 | | -1.04 | NM_198086.1 | JUB | Ajuba LIM protein | -1.53 |
| NM_173809.2 | BLOC1S2 | biogenesis of lysosomal organelles complex-1, subunit 2 | -1.72 | BC090880.1 | EIF3S3 | Eukaryotic translation initiation factor 3, subunit H | | -1.05 | NM_148910.2 | TIRAP | toll-interleukin 1 receptor (TIR) domain containing adaptor protein | -1.13 |
| NM_016224.3 | SNX9 | sorting nexin 9 | -1.13 | NM_198086.1 | JUB | Ajuba LIM protein | | -1.07 | NM_001005465.1 | OR10G3 | olfactory receptor, family 10, subfamily G, member 3 | -1.15 |
| Lhx1 | Lhx1 | LIM homeobox 1 | -1.01 | NM_016224.3 | SNX9 | sorting nexin 9 | | -1.14 | NM_003099.3 | SNX1 | sorting nexin 1 | 1.07 |
| Nol3 | Nol3 | nucleolar protein 3 (apoptosis repressor with CARD domain) | -1.39 |  |  |  | |  | Nol3 | Nol3 | nucleolar protein 3 (apoptosis repressor with CARD domain) | -1.88 |
|  |  |  |  |  |  |  | |  | XM_290842.4 | LRFN1 | leucine rich repeat and fibronectin type III domain containing 1 | -1.22 |
|  |  |  |  |  |  |  | |  | NM_001033112.1 | PAIP2 | poly(A) binding protein interacting protein 2 | 1.04 |

| **Table 2: Trends of proteins common in mass spectrometric analysis and corresponding autoantibody response** | | | | | | | |
| --- | --- | --- | --- | --- | --- | --- | --- |
| **Proteins up-regulated in MS with elevated autoantibody response** | | | | | | | |
| **Gene Symbol** | **Uniprot Accession** | **Protein name** | **Fold Change in MS Orbitrap_MGI** | **Fold Change in MA_MGI** | **mRNA data from GEO (GSE43290)** | **Fold Change in MS Orbitrap_MGII** | **Fold Change in MA_MGII** |
| GSTP1 | P09211 | Glutathione S-transferase P | 1.62 | 0.51 | No data available | 2.32 | Not significant |
| C11orf67 | Q9H7C9 | Mth938 domain-containing protein | 1.77 | 0.52 | No data available | 2.38 | Not significant |
| RPS13 | P62277 | 40S ribosomal protein S13gi\|51316609\|sp\|Q6ITC7.3\|RS13_CHICK RecName | 1.81 | 0.51 | No data available | 1.46 | Not significant |
| SELENBP1 | Q13228 | Selenium-binding protein 1 | 1.55 | 0.51 | No data available | 2.14 | Not significant |
| FABP5 | Q01469 | Fatty acid-binding protein, epidermal | 1.79 | 0.55 | No data available | 3.83 | Not significant |
|  |  |  |  |  |  |  |  |
| TPD52L2 | O43399 | Tumor protein D54 | 1.35 | 0.60 | No data available | 2.54 | Not significant |
| PDXK | O00764 | Pyridoxal kinase | 0.83 | 0.53 | No data available | 0.87 | Not significant |
| **Proteins down-regulated in MS with downregulated autoantibody response** | | | | | | | |
| **Gene Symbol** | **Uniprot Accesion** | **Protein name** | **Fold Change in MS_Orbitrap_MGI** | **Fold Change in MA_MGI** | **mRNA data from GEO (GSE43290)** | **Fold Change in MS_Orbitrap_MGII** | **Fold Change in MA_MGII** |
| CRYM | Q14894 | Ketimine reductase mu-crystallin | 0.10 | -1.44 | down-regulated | 0.13 | -1.43 |
| APOE | P02649 | Apolipoprotein E | 0.53 | -0.54 | No data available | 0.61 | Not significant |
| COX4I1 | P13073 | Cytochrome c oxidase subunit 4 isoform 1, mitochondrial | 0.17 | -0.51 | No data available | 0.25 | -0.62 |
| MARCKSL1 | P49006 | MARCKS-related protein | 0.30 | -0.64 | No data available | 0.42 | Not significant |
| EPB41L3 | Q9Y2J2 | Band 4.1-like protein 3 | 0.48 | -0.53 | No data available | 0.47 | Not significant |
| RTN4 | Q9NQC3 | Reticulon-4 | 0.60 | -0.62 | No data available | 0.65 | Not significant |
| QDPR | P09417 | Dihydropteridine reductase | 0.21 | -0.52 | No data available | 0.23 | Not significant |
| HSPA2 | P54652 | Heat shock-related 70 kDa protein 2 | 0.28 | -0.70 | No data available | 0.32 | Not significant |
| **Proteins with opposite trends in MS and autoantibody response** | | | | | | | |
| **Gene Symbol** | **Uniprot Accesion** | **Protein name** | **Fold Change in MS_Orbitrap_MGI** | **Fold Change in MA_MGI** | **mRNA data from GEO (GSE43290)** | **Fold Change in MS_Orbitrap_MGII** | **Fold Change in MA_MGII** |
| PPP2R4 | Q15257 | Serine/threonine-protein phosphatase 2A activator | 0.581 | 0.69 | No data available | 0.55 | 0.60 |
| NME1 | P15531 | Nucleoside diphosphate kinase A | 0.624 | 0.69 | No data available | 1.40 | Not significant |
| ACO2 | Q99798 | Aconitate hydratase, mitochondrial | 0.602 | 0.57 | No data available | 0.53 | Not significant |
| YWHAB | P31946 | 14-3-3 protein beta/alpha | 0.393 | 0.67 | No data available | 0.37 | Not significant |
| C21orf33 | P30042 | ES1 protein homolog, mitochondrial | 0.516 | 0.50 | No data available | 0.80 | Not significant |
| VCP | P55072 | Transitional endoplasmic reticulum ATPase | 1.558 | -0.61 | No data available | 1.13 | Not significant |
| RNPEP | Q9H4A4 | Aminopeptidase B | 2.060 | -0.52 | No data available | 1.76 | Not significant |
| ALDH9A1 | P49189 | 4-trimethylaminobutyraldehyde dehydrogenase | 1.396 | -0.63 | No data available | 1.54 | Not significant |
| CARHSP1 | Q9Y2V2 | Calcium-regulated heat stable protein 1 | 1.191 | -0.81 | up-regulated | 2.21 | -1.13 |
| UBE2V2 | Q15819 | Ubiquitin-conjugating enzyme E2 variant 2 | 0.37 | Not significant | No data available | 0.40 | 0.66 |

**Supplementary Table 3.5: Comparison with serum proteomics study**

|  |  | **Present in Grade** | **Microarray analysis** | | | | | **Mass Spectrometric analysis** | | |
| --- | --- | --- | --- | --- | --- | --- | --- | --- | --- | --- |
| **Name** | **Gene symbol** | **MG/MG1/MG2** | **Log FC** | **Trend** | **p-value** | **Adjusted p-value** | **Abs log FC** | **Fold change in MS_MG1** | **Fold change in MS_MG2** | **Fold change in MS_MG3** |
| **Ig gamma-4 chain C region** | **IGHG4** | HCvsMG | -3.15 | Downregulated | 8.4E-40 | 1.5E-35 | 3.1E+00 | 0.6 | 1.1 | 2.6 |
|  |  | HCvsMG1 | -3.15 |  | 8.0E-38 | 1.4E-33 | 3.1E+00 | Downregulated | Not significant | Downregulated |
|  |  | HCvsMG2 | -3.15 |  | 3.7E-32 | 6.8E-28 | 3.1E+00 |  |  |  |
| **immunoglobulin heavy constant gamma 1 (G1m marker)(IGHG1)** | **IGHG1** | HCvsMG | 0.56 | Upregulated | 1.5E-05 | 7.2E-04 | 5.6E-01 | 0.4 | 0.5 | 0.6 |
|  |  | HCvsMG1 | 0.59 |  | 2.6E-05 | 6.6E-04 | 5.9E-01 | Downregulated | Downregulated | Downregulated |
|  |  | HCvsMG2 | 0.61 |  | 7.8E-06 | 3.2E-03 | 6.1E-01 | NA | NA | NA |
| **Apolipoprotein E** | **APOE** | HCvsMG | NA | NA | NA | NA | NA | NA | NA | NA |
|  |  | HCvsMG1 | -0.54 | Downregulated | 3.9E-08 | 7.7E-06 | 5.4E-01 | 2.5 | 1.5 | 2.0 |
|  |  | HCvsMG2 | NA | NA | NA | NA | NA | Upregulated | Not significant | Upregulated |
| **Ig delta chain C region** | **IGHD** | HCvsMG | NA | NA | NA | NA | NA | 0.3 | 0.7 | 0.3 |
|  |  | HCvsMG1 | -0.61 | Downregulated | 2.0E-04 | 2.7E-03 | 6.1E-01 | Downregulated | Not significant | Downregulated |
|  |  | HCvsMG2 | NA | NA | NA | NA | NA | NA | NA | NA |
| **fibrinogen alpha chain(FGA)** | **FGA** | HCvsMG | NA | NA | NA | NA | NA | 1.2 | 1.7 | 1.0 |
|  |  | HCvsMG1 | 0.53 | Upregulated | 5.0E-04 | 5.0E-03 | 5.3E-01 | Not significant | Upregulated | Not signifIcant |
|  |  | HCvsMG2 | NA | NA | NA | NA | NA | NA | NA | NA |

Yellow: Indicates common trend in both mass spectrometric study as well as autoantibody screening study. Red: Upregulated in mass spectrometry studies; Green: Down regulated in mass spectrometric studies. For autoantibody screening data Log FC>=0.5 indicates upregulation; while Log FC<=-0.5 indicates significant downregulation. (Data procured from Sharma *et al*., 2014^1^ ‘NA’ indicates not applicable, ‘NS’ indicates values below the threshold as mentioned in the manuscript.

1. Sharma, S., Ray, S., Moiyadi, A., Sridhar, E. & Srivastava, S. Quantitative proteomic analysis of meningiomas for the identification of surrogate protein markers. *Sci. Rep.* **4,** 7140 (2014).
