# Supplementary material for: Evaluation of autoantibody signatures in meningioma patients using human proteome arrays
Source: Oncotarget. 2017 Apr 10;8(35):58443–56. doi: 10.18632/oncotarget.16997 (PMC5601665; doi:10.18632/oncotarget.16997)
Supplement: Supplementary file 11 [file oncotarget-08-58443-s011.docx]

**Supplementary Table 4:**

**Supplementary 4.1: Full Extended Tables for Main Table 1 and Table 2**

| **Table 1: Significantly dysregulated proteins across all comparisons (absolute logFC>1, p<0.05)** | | | | | | | | | | | | |
| --- | --- | --- | --- | --- | --- | --- | --- | --- | --- | --- | --- | --- |
| **HC vs MG** | | | | **HC vs MG1** | | | | | **HC vs MG2** | | | |
| **ID** | **Symbol** | **Name** | **logFC** | **ID** | **Symbol** | | **Name** | **logFC** | **ID** | **Symbol** | **Name** | **logFC** |
| BC025985.1 | IGHG4 | immunoglobulin heavy constant gamma 4 (G4m marker) | -3.15 | BC025985.1 | IGHG4 | immunoglobulin heavy constant gamma 4 (G4m marker) | | -3.15 | BC025985.1 | IGHG4 | immunoglobulin heavy constant gamma 4 (G4m marker) | -3.15 |
| NM_001014444.1 | CRYM | crystallin, mu | -1.44 | NM_001014444.1 | CRYM | crystallin, mu | | -1.44 | NM_001014444.1 | CRYM | crystallin, mu | -1.43 |
| NM_032328.1 | EFCAB2 | EF-hand calcium binding domain 2 | 1.08 | NM_032328.1 | EFCAB2 | EF-hand calcium binding domain 2 | | 1.10 | BC065370.1 | C20orf112 | Chromosome 20 open reading frame 112 | -2.18 |
| BC065370.1 | C20orf112 | Chromosome 20 open reading frame 112 | -2.01 | NM_031304.2 | DOHH | deoxyhypusine hydroxylase/monooxygenase | | -1.01 | NM_032328.1 | EFCAB2 | EF-hand calcium binding domain 2 | 1.06 |
| BC037876.1 | C17orf57 | Chromosome 20 open reading frame 112 | -1.61 | NM_015726.2 | WDR42A | DDB1 and CUL4 associated factor 8 | | -1.31 | NM_005719.2 | ARPC3 | actin related protein 2/3 complex, subunit 3, 21kDa | -1.48 |
| NM_001033515.1 | LOC389833 | EF-hand calcium binding domain 13 | -1.12 | BC065370.1 | C20orf112 | Chromosome 20 open reading frame 112 | | -1.83 | NM_001042476.1 | CARHSP1 | calcium regulated heat stable protein 1, 24kDa | -1.13 |
| NM_005719.2 | ARPC3 | actin related protein 2/3 complex, subunit 3, 21kDa | -1.16 | BC006453.1 | HDAC7A | Histone deacetylase 7 | | 1.00 | BC037876.1 | C17orf57 | EF-hand calcium binding domain 13 | -1.67 |
| NM_002767.2 | PRPSAP2 | phosphoribosyl pyrophosphate synthetase-associated protein 2 | -1.20 | BC037876.1 | C17orf57 | EF-hand calcium binding domain 13 | | -1.55 | NM_004264.2 | SURB7 | Mediator complex subunit 21 | -1.04 |
| NM_001005465.1 | OR10G3 | olfactory receptor, family 10, subfamily G, member 3 | -1.30 | NM_001033515.1 | LOC389833 |  | | -1.14 | NM_182789.2 | PAIP1 | poly(A) binding protein interacting protein 1 | 1.06 |
| NM_139204.1 | EPS8L1 | EPS8-like 1 | 1.25 | NM_001005465.1 | ND |  | | -1.44 | NM_001033515.1 | LOC389833 |  | -1.09 |
| NM_001025266.1 | LOC285382 |  | 1.37 | NM_139204.1 | EPS8L1 | EPS8-like 1 | | 1.22 | BC013992.1 | MAPK3 | mitogen-activated protein kinase 3 | 1.09 |
| NM_014372.3 | RNF11 | ring finger protein 11 | -1.26 | NM_001025266.1 | LOC285382 |  | | 1.32 | NM_002767.2 | PRPSAP2 | phosphoribosyl pyrophosphate synthetase-associated protein 2 | -1.46 |
| NM_148910.2 | TIRAP | toll-interleukin 1 receptor (TIR) domain containing adaptor protein | -1.09 | NM_148910.2 | TIRAP | toll-interleukin 1 receptor (TIR) domain containing adaptor protein | | -1.05 | NM_014372.3 | RNF11 | ring finger protein 11 | -1.53 |
| NM_198086.1 | JUB | Ajuba LIM protein | -1.30 | NM_002893.2 | RBBP7 | retinoblastoma binding protein 7 | | -1.00 | NM_173809.2 | BLOC1S2 | biogenesis of lysosomal organelles complex-1, subunit 2 | -2.60 |
| XM_290842.4 | LRFN1 | leucine rich repeat and fibronectin type III domain containing 1 | -1.14 | NM_021810.3 | CDH26 | cadherin 26 | | -1.04 | NM_139204.1 | EPS8L1 | EPS8-like 1 | 1.29 |
| NM_021810.3 | CDH26 | cadherin 26 | -1.01 | XM_290842.4 | LRFN1 | leucine rich repeat and fibronectin type III domain containing 1 | | -1.05 | NM_001025266.1 | LOC285382 |  | 1.43 |
| BC090880.1 | EIF3S3 | Eukaryotic translation initiation factor 3, subunit H | -1.11 | NM_018584.4 | CAMK2N1 | calcium/calmodulin-dependent protein kinase II inhibitor 1 | | -1.04 | NM_198086.1 | JUB | Ajuba LIM protein | -1.53 |
| NM_173809.2 | BLOC1S2 | biogenesis of lysosomal organelles complex-1, subunit 2 | -1.72 | BC090880.1 | EIF3S3 | Eukaryotic translation initiation factor 3, subunit H | | -1.05 | NM_148910.2 | TIRAP | toll-interleukin 1 receptor (TIR) domain containing adaptor protein | -1.13 |
| NM_016224.3 | SNX9 | sorting nexin 9 | -1.13 | NM_198086.1 | JUB | Ajuba LIM protein | | -1.07 | NM_001005465.1 | OR10G3 | olfactory receptor, family 10, subfamily G, member 3 | -1.15 |
| Lhx1 | Lhx1 | LIM homeobox 1 | -1.01 | NM_016224.3 | SNX9 | sorting nexin 9 | | -1.14 | NM_003099.3 | SNX1 | sorting nexin 1 | 1.07 |
| Nol3 | Nol3 | nucleolar protein 3 (apoptosis repressor with CARD domain) | -1.39 |  |  |  | |  | Nol3 | Nol3 | nucleolar protein 3 (apoptosis repressor with CARD domain) | -1.88 |
|  |  |  |  |  |  |  | |  | XM_290842.4 | LRFN1 | leucine rich repeat and fibronectin type III domain containing 1 | -1.22 |
|  |  |  |  |  |  |  | |  | NM_001033112.1 | PAIP2 | poly(A) binding protein interacting protein 2 | 1.04 |

| **Table 2: Trends of proteins common in mass spectrometric analysis and corresponding autoantibody response** | | | | | | | |
| --- | --- | --- | --- | --- | --- | --- | --- |
| **Proteins up-regulated in MS with elevated autoantibody response** | | | | | | | |
| **Gene Symbol** | **Uniprot Accession** | **Protein name** | **Fold Change in MS Orbitrap_MGI** | **Fold Change in MA_MGI** | **mRNA data from GEO (GSE43290)** | **Fold Change in MS Orbitrap_MGII** | **Fold Change in MA_MGII** |
| GSTP1 | P09211 | Glutathione S-transferase P | 1.62 | 0.51 | No data available | 2.32 | Not significant |
| C11orf67 | Q9H7C9 | Mth938 domain-containing protein | 1.77 | 0.52 | No data available | 2.38 | Not significant |
| RPS13 | P62277 | 40S ribosomal protein S13gi\|51316609\|sp\|Q6ITC7.3\|RS13_CHICK RecName | 1.81 | 0.51 | No data available | 1.46 | Not significant |
| SELENBP1 | Q13228 | Selenium-binding protein 1 | 1.55 | 0.51 | No data available | 2.14 | Not significant |
| FABP5 | Q01469 | Fatty acid-binding protein, epidermal | 1.79 | 0.55 | No data available | 3.83 | Not significant |
|  |  |  |  |  |  |  |  |
| TPD52L2 | O43399 | Tumor protein D54 | 1.35 | 0.60 | No data available | 2.54 | Not significant |
| PDXK | O00764 | Pyridoxal kinase | 0.83 | 0.53 | No data available | 0.87 | Not significant |
| **Proteins down-regulated in MS with downregulated autoantibody response** | | | | | | | |
| **Gene Symbol** | **Uniprot Accesion** | **Protein name** | **Fold Change in MS_Orbitrap_MGI** | **Fold Change in MA_MGI** | **mRNA data from GEO (GSE43290)** | **Fold Change in MS_Orbitrap_MGII** | **Fold Change in MA_MGII** |
| CRYM | Q14894 | Ketimine reductase mu-crystallin | 0.10 | -1.44 | down-regulated | 0.13 | -1.43 |
| APOE | P02649 | Apolipoprotein E | 0.53 | -0.54 | No data available | 0.61 | Not significant |
| COX4I1 | P13073 | Cytochrome c oxidase subunit 4 isoform 1, mitochondrial | 0.17 | -0.51 | No data available | 0.25 | -0.62 |
| MARCKSL1 | P49006 | MARCKS-related protein | 0.30 | -0.64 | No data available | 0.42 | Not significant |
| EPB41L3 | Q9Y2J2 | Band 4.1-like protein 3 | 0.48 | -0.53 | No data available | 0.47 | Not significant |
| RTN4 | Q9NQC3 | Reticulon-4 | 0.60 | -0.62 | No data available | 0.65 | Not significant |
| QDPR | P09417 | Dihydropteridine reductase | 0.21 | -0.52 | No data available | 0.23 | Not significant |
| HSPA2 | P54652 | Heat shock-related 70 kDa protein 2 | 0.28 | -0.70 | No data available | 0.32 | Not significant |
| **Proteins with opposite trends in MS and autoantibody response** | | | | | | | |
| **Gene Symbol** | **Uniprot Accesion** | **Protein name** | **Fold Change in MS_Orbitrap_MGI** | **Fold Change in MA_MGI** | **mRNA data from GEO (GSE43290)** | **Fold Change in MS_Orbitrap_MGII** | **Fold Change in MA_MGII** |
| PPP2R4 | Q15257 | Serine/threonine-protein phosphatase 2A activator | 0.581 | 0.69 | No data available | 0.55 | 0.60 |
| NME1 | P15531 | Nucleoside diphosphate kinase A | 0.624 | 0.69 | No data available | 1.40 | Not significant |
| ACO2 | Q99798 | Aconitate hydratase, mitochondrial | 0.602 | 0.57 | No data available | 0.53 | Not significant |
| YWHAB | P31946 | 14-3-3 protein beta/alpha | 0.393 | 0.67 | No data available | 0.37 | Not significant |
| C21orf33 | P30042 | ES1 protein homolog, mitochondrial | 0.516 | 0.50 | No data available | 0.80 | Not significant |
| VCP | P55072 | Transitional endoplasmic reticulum ATPase | 1.558 | -0.61 | No data available | 1.13 | Not significant |
| RNPEP | Q9H4A4 | Aminopeptidase B | 2.060 | -0.52 | No data available | 1.76 | Not significant |
| ALDH9A1 | P49189 | 4-trimethylaminobutyraldehyde dehydrogenase | 1.396 | -0.63 | No data available | 1.54 | Not significant |
| CARHSP1 | Q9Y2V2 | Calcium-regulated heat stable protein 1 | 1.191 | -0.81 | up-regulated | 2.21 | -1.13 |
| UBE2V2 | Q15819 | Ubiquitin-conjugating enzyme E2 variant 2 | 0.37 | Not significant | No data available | 0.40 | 0.66 |

**Supplementary 4.2: Comparison with serum proteomics study**

|  |  | **Present in Grade** | **Microarray analysis** | | | | | **Mass Spectrometric analysis** | | |
| --- | --- | --- | --- | --- | --- | --- | --- | --- | --- | --- |
| **Name** | **Gene symbol** | **MG/MG1/MG2** | **Log FC** | **Trend** | **p-value** | **Adjusted p-value** | **Abs log FC** | **Fold change in MS_MG1** | **Fold change in MS_MG2** | **Fold change in MS_MG3** |
| **Ig gamma-4 chain C region** | **IGHG4** | HCvsMG | -3.15 | Downregulated | 8.4E-40 | 1.5E-35 | 3.1E+00 | 0.6 | 1.1 | 2.6 |
|  |  | HCvsMG1 | -3.15 |  | 8.0E-38 | 1.4E-33 | 3.1E+00 | Downregulated | Not significant | Downregulated |
|  |  | HCvsMG2 | -3.15 |  | 3.7E-32 | 6.8E-28 | 3.1E+00 |  |  |  |
| **immunoglobulin heavy constant gamma 1 (G1m marker)(IGHG1)** | **IGHG1** | HCvsMG | 0.56 | Upregulated | 1.5E-05 | 7.2E-04 | 5.6E-01 | 0.4 | 0.5 | 0.6 |
|  |  | HCvsMG1 | 0.59 |  | 2.6E-05 | 6.6E-04 | 5.9E-01 | Downregulated | Downregulated | Downregulated |
|  |  | HCvsMG2 | 0.61 |  | 7.8E-06 | 3.2E-03 | 6.1E-01 | NA | NA | NA |
| **Apolipoprotein E** | **APOE** | HCvsMG | NA | NA | NA | NA | NA | NA | NA | NA |
|  |  | HCvsMG1 | -0.54 | Downregulated | 3.9E-08 | 7.7E-06 | 5.4E-01 | 2.5 | 1.5 | 2.0 |
|  |  | HCvsMG2 | NA | NA | NA | NA | NA | Upregulated | Not significant | Upregulated |
| **Ig delta chain C region** | **IGHD** | HCvsMG | NA | NA | NA | NA | NA | 0.3 | 0.7 | 0.3 |
|  |  | HCvsMG1 | -0.61 | Downregulated | 2.0E-04 | 2.7E-03 | 6.1E-01 | Downregulated | Not significant | Downregulated |
|  |  | HCvsMG2 | NA | NA | NA | NA | NA | NA | NA | NA |
| **fibrinogen alpha chain(FGA)** | **FGA** | HCvsMG | NA | NA | NA | NA | NA | 1.2 | 1.7 | 1.0 |
|  |  | HCvsMG1 | 0.53 | Upregulated | 5.0E-04 | 5.0E-03 | 5.3E-01 | Not significant | Upregulated | Not signifIcant |
|  |  | HCvsMG2 | NA | NA | NA | NA | NA | NA | NA | NA |

Yellow: Indicates common trend in both mass spectrometric study as well as autoantibody screening study. Red: Upregulated in mass spectrometry studies; Green: Down regulated in mass spectrometric studies. For autoantibody screening data Log FC>=0.5 indicates upregulation; while Log FC<=-0.5 indicates significant downregulation. (Data procured from Sharma *et al*., 2014^1^ ‘NA’ indicates not applicable, ‘NS’ indicates values below the threshold as mentioned in the manuscript.

1. Sharma, S., Ray, S., Moiyadi, A., Sridhar, E. & Srivastava, S. Quantitative proteomic analysis of meningiomas for the identification of surrogate protein markers. *Sci. Rep.* **4,** 7140 (2014).

**Supplementary Table 4.3: IQTL analysis for SELENBP1**

| IQTL data for SELENBP1 | | | |  |  |  |  |  |  |  |  |  |  |  |  |  |
| --- | --- | --- | --- | --- | --- | --- | --- | --- | --- | --- | --- | --- | --- | --- | --- | --- |
| **GRADE** | **SAMPLE ID** | **Selenium Binding Protein 1(SBP1) 57kDa** | | | | **Beta-Actin(42 kDa)** | | | |  |  |  |  |  |  |  |
| **MGI** | CM/14915 | 15004596 | 18646501 | 186250066 | **73300388** | 11206924 | 9506826 | 91371354 | **37361701** |  |  |  |  |  |  |  |
| **MGI** | CM/20619 | 16257976 | 17964287 | 165440058 | **66554107** | 10986264 | 6449491.4 | 92867742 | **36767832** |  |  |  |  |  |  |  |
| **MGI** | CM/29552 | 17637459 | 20363061.06 | 144517920 | **60839480** | 10173879 | 6232340 | 83382892 | **33263037** |  |  |  |  |  |  |  |
| **MGI** | CM/29822 | 0 | 11900739.45 | 145799226 | **52566655** | 0 | 4622464.9 | 102829174 | **35817213** |  |  |  |  |  |  |  |
| **MGII** | CM/20453 | 11434945 | 6518872 | 78971500 | **32308439** | 11434945 | 7229281 | 85200690 | **34621639** |  |  |  |  |  |  |  |
| **MGII** | CM/16399 | 8267592 | 7286946.97 | 59082356 | **24878965** | 8267592 | 6100602 | 57584194 | **23984129** |  |  |  |  |  |  |  |
| **MGII** | CM/22287 | 11002064 | 6513319.08 | 71531986 | **29682456** | 11002064 | 9066574.9 | 86155522 | **35408054** |  |  |  |  |  |  |  |
| **MGII** | CM/5736 | 0 | 9304592.73 | 74157208 | **27820600** | 0 | 6131576 | 64320584 | **23484053** |  |  |  |  |  |  |  |
| **MGII** | CM/10571 | 0 | 4754500.33 | 76177360 | **26977287** | 0 | 9021775.8 | 82856166 | **30625981** |  |  |  |  |  |  |  |
|  |  |  |  |  |  |  |  |  |  |  |  |  |  |  |  |  |
| **Average values** | | | |  |  |  |  |  |  |  |  |  |  |  |  |  |
| **GRADE** | **SAMPLE ID** | **SBP1** | **B-actin** |  |  |  |  |  |  |  |  |  |  |  |  |  |
| **MGI** | CM/14915 | **73300388** | **37361701.33** |  |  |  |  |  |  |  |  |  |  |  |  |  |
| **MGI** | CM/20619 | **66554107** | **36767832.48** |  |  |  |  |  |  |  |  |  |  |  |  |  |
| **MGI** | CM/29552 | **60839480** | **33263037** |  |  |  |  |  |  |  |  |  |  |  |  |  |
| **MGI** | CM/29822 | **52566655** | **35817212.97** |  |  |  |  |  |  |  |  |  |  |  |  |  |
| **MGII** | CM/20453 | **32308439** | **34621638.67** |  |  |  |  |  |  |  |  |  |  |  |  |  |
| **MGII** | CM/16399 | **24878965** | **23984129.33** |  |  |  |  |  |  |  |  |  |  |  |  |  |
| **MGII** | CM/22287 | **29682456** | **35408053.64** |  |  |  |  |  |  |  |  |  |  |  |  |  |
| **MGII** | CM/5736 | **27820600** | **23484053.33** |  |  |  |  |  |  |  |  |  |  |  |  |  |
| **MGII** | CM/10571 | **26977287** | **30625980.58** |  |  |  |  |  |  |  |  |  |  |  |  |  |
|  |  |  |  |  |  |  |  |  |  |  |  |  |  |  |  |  |
| **Beta-actin normalization** | | | |  | SBP1(normalization) | |  |  |  |  |  |  |  |  |  |  |
| **GRADE** | **SAMPLE ID** | **B-actin** | Median Value | **n B-Actin** | avg | **n SBP1** |  |  |  |  |  |  |  |  |  |  |
| **MGI** | CM/14915 | **37361701** | 34621638.67 | 1.08 | **73300388** | 79101605 |  |  |  |  |  |  |  |  |  |  |
| **MGI** | CM/20619 | **36767832** |  | 1.06 | **66554107** | 70679793 |  |  |  |  |  |  |  |  |  |  |
| **MGI** | CM/29552 | **33263037** |  | 0.96 | **60839480** | 58452053 |  |  |  |  |  |  |  |  |  |  |
| **MGI** | CM/29822 | **35817213** |  | 1.03 | **52566655** | 54381917 |  |  |  |  |  |  |  |  |  |  |
| **MGII** | CM/20453 | **34621639** |  | 1.00 | **32308439** | 32308439 |  |  |  |  |  |  |  |  |  |  |
| **MGII** | CM/16399 | **23984129** |  | 0.69 | **24878965** | 17234895 |  |  |  |  |  |  |  |  |  |  |
| **MGII** | CM/22287 | **35408054** |  | 1.02 | **29682456** | 30356680 |  |  |  |  |  |  |  |  |  |  |
| **MGII** | CM/5736 | **23484053** |  | 0.68 | **27820600** | 18870870 |  |  |  |  |  |  |  |  |  |  |
| **MGII** | CM/10571 | **30625981** |  | 0.88 | **26977287** | 23863858 |  |  |  |  |  |  |  |  |  |  |
|  |  |  |  |  |  |  |  |  |  |  |  |  |  |  |  |  |
|  |  |  |  |  |  |  |  |  |  |  |  |  |  |  |  |  |
|  |  |  | | |  |  |  |  |  |  |  |  |  |  |  |  |
| **GRADE** | **SAMPLE ID** | **n SBP1** | avg |  | **GRADE** | **n SBP1** | st dev |  |  |  |  |  |  |  |  |  |
| **MGI** | CM/14915 | 79101605 | 65653842.19 |  | **MGI** | 65653842 | 11328792 |  |  |  |  |  |  |  |  |  |
| **MGI** | CM/20619 | 70679793 |  |  | **MGII** | 24526949 | 6710855.3 |  |  |  |  |  |  |  |  |  |
| **MGI** | CM/29552 | 58452053 |  |  |  |  |  |  |  |  |  |  |  |  |  |  |
| **MGI** | CM/29822 | 54381917 |  |  |  |  |  |  |  |  |  |  |  |  |  |  |
| **MGII** | CM/20453 | 32308439 | 24526948.5 |  |  |  |  |  |  |  |  |  |  |  |  |  |
| **MGII** | CM/16399 | 17234895 |  |  |  |  |  |  |  |  |  |  |  |  |  |  |
| **MGII** | CM/22287 | 30356680 |  |  |  |  |  |  |  |  |  |  |  |  |  |  |
| **MGII** | CM/5736 | 18870871 |  |  |  |  |  |  |  |  |  |  |  |  |  |  |
| **MGII** | CM/10571 | 23863858 |  |  |  |  |  |  |  |  |  |  |  |  |  |  |
|  |  |  |  |  |  |  |  |  |  |  |  |  |  |  |  |  |

p-value: 0.000248

**Supplementary Table 4.4: IQTL data for TPD52**

| IQTL data for TPD52 | | | |  |  |  |  |  |  |  |  |  |  |
| --- | --- | --- | --- | --- | --- | --- | --- | --- | --- | --- | --- | --- | --- |
| **GRADE** | **SAMPLE ID** | **TPD52L2 (27KDa)** | | | |  | **Beta-Actin(42 kDa)** |  |  |  |  |  |  |
| **MGI** | CM/14915 | 1579.29 | 61667084 | 30834331.65 |  | 1490.94 | 57658341 | **28829915.97** |  |  |  |  |  |
| **MGI** | CM/30755 | 1678.21 | 65135435 | 32568556.61 |  | 1592.47 | 61588252 | **30794922.24** |  |  |  |  |  |
| **MGI** | CM/20619 | 1561.74 | 62297465 | 31149513.37 |  | 1474.36 | 59963394 | **29982434.18** |  |  |  |  |  |
| **MGI** | CM/13944 | 1543.24 | 62803870 | 31402706.62 |  | 1330.44 | 59152477 | **29576903.72** |  |  |  |  |  |
| **MGII** | CM/10571 | 2257.87 | 65526765 | 32764511.44 |  | 1705.34 | 64666016 | **32333860.67** |  |  |  |  |  |
| **MGII** | CM/20453 | 1582.01 | 45900648 | 22951115.01 |  | 1761.84 | 48988358 | **24495059.92** |  |  |  |  |  |
| **MGII** | CM/16399 | 1296.53 | 48856544 | 24428920.27 |  | 1767.1 | 49213888 | **24607827.55** |  |  |  |  |  |
| **MGII** | CM/22287 | 1879.54 | 52193953 | 26097916.27 |  | 1792.24 | 65427976 | **32714884.12** |  |  |  |  |  |
|  |  |  |  |  |  |  |  |  |  |  |  |  |  |
| **Average values** | | | | |  |  |  |  |  |  |  |  |  |
| **GRADE** | **SAMPLE ID** | **TPD52L2** | **B-actin** |  |  |  |  |  |  |  |  |  |  |
| **MGI** | CM/14915 | **30834331.65** | **28829915.97** |  |  |  |  |  |  |  |  |  |  |
| **MGI** | CM/30755 | **32568556.61** | **30794922.24** |  |  |  |  |  |  |  |  |  |  |
| **MGI** | CM/20619 | **31149513.37** | **29982434.18** |  |  |  |  |  |  |  |  |  |  |
| **MGI** | CM/13944 | **31402706.62** | **29576903.72** |  |  |  |  |  |  |  |  |  |  |
| **MGII** | CM/10571 | **32764511.44** | **32333860.67** |  |  |  |  |  |  |  |  |  |  |
| **MGII** | CM/20453 | **22951115.01** | **24495059.92** |  |  |  |  |  |  |  |  |  |  |
| **MGII** | CM/16399 | **24428920.27** | **24607827.55** |  |  |  |  |  |  |  |  |  |  |
| **MGII** | CM/22287 | **26097916.27** | **32714884.12** |  |  |  |  |  |  |  |  |  |  |
|  |  |  |  |  |  |  |  |  |  |  |  |  |  |
|  |  |  |  |  |  |  |  |  |  |  |  |  |  |
| **Beta-actin normalization** | | | |  | TPD52L2(normalization) | |  |  |  |  |  |  |  |
| **GRADE** | **SAMPLE ID** | **B-actin** | Median Value | **n B-Actin** | AVG | **NTPD52L2** |  |  |  |  |  |  |  |
| **MGI** | CM/14915 | **28829915.97** |  | 1.01 | **30834331.6** | 31194826.01 |  |  |  |  |  |  |  |
| **MGI** | CM/30755 | **30794922.24** |  | 0.95 | **32568556.6** | 30846848.81 |  |  |  |  |  |  |  |
| **MGI** | CM/20619 | **29982434.18** |  | 0.97 | **31149513.4** | 30302313.18 |  |  |  |  |  |  |  |
| **MGI** | CM/13944 | **29576903.72** | 29166976.05 | 0.99 | **31402706.6** | 30967473.82 |  |  |  |  |  |  |  |
| **MGII** | CM/10571 | **32333860.67** |  | 0.90 | **32764511.4** | 29555447.46 |  |  |  |  |  |  |  |
| **MGII** | CM/20453 | **24495059.92** |  | 1.19 | **22951115** | 27328556.2 |  |  |  |  |  |  |  |
| **MGII** | CM/16399 | **24607827.55** |  | 1.19 | **24428920.3** | 28954922.2 |  |  |  |  |  |  |  |
| **MGII** | CM/22287 | **32714884.12** |  | 0.89 | **26097916.3** | 23267614.09 |  |  |  |  |  |  |  |
|  |  |  |  |  |  |  |  |  |  |  |  |  |  |
|  |  |  |  |  |  |  |  |  |  |  |  |  |  |
|  |  |  | | |  |  |  |  |  |  |  |  |  |
| **GRADE** | **SAMPLE ID** | **n TPD52L2** | avg |  | **GRADE** | **n TPD52L2** |  |  |  |  |  |  |  |
| **MGI** | CM/14915 | 31194826.01 | 30827865.45 |  | **MGI** | 30827865.45 | st dev | st err | Ttest |  |  |  |  |
| **MGI** | CM/30755 | 30846848.81 |  |  | **MGII** | 27276634.99 | 378909.01 | 189454.5041 | 0.047510413 |  |  |  |  |
| **MGI** | CM/20619 | 30302313.18 |  |  |  |  | 2833406.6 | 1416703.292 |  |  |  |  |  |
| **MGI** | CM/13944 | 30967473.82 |  |  |  |  |  |  |  |  |  |  |  |
| **MGII** | CM/10571 | 29555447.46 | 27276634.99 |  | \|  \| \| --- \| |  |  |  |  |  |  |  |  |
| **MGII** | CM/20453 | 27328556.2 |  |  |  |  |  |  | p-value=0.047510413 |  |  |  |  |
| **MGII** | CM/16399 | 28954922.2 |  |  |  |  |  |  |  |  |  |  |  |
| **MGII** | CM/22287 | 23267614.09 |  |  |  |  |  |  |  |  |  |  |  |
|  |  |  |  |  |  |  |  |  |  |  |  |  |  |
|  |  |  |  |  |  |  |  |  |  |  |  |  |  |
|  |  |  |  |  |  |  |  |  |  |  |  |  |  |
|  |  |  |  |  |  |  |  |  |  |  |  |  |  |
|  |  |  |  |  |  |  |  |  |  |  |  |  |  |
|  |  |  |  |  |  |  |  |  |  |  |  |  |  |
|  |  |  |  |  |  |  |  |  |  |  |  |  |  |
|  |  |  |  |  |  |  |  |  |  |  |  |  |  |
|  |  |  |  |  |  |  |  |  |  |  |  |  |  |
|  |  |  |  |  |  |  |  |  |  |  |  |  |  |
|  |  |  |  |  |  |  |  |  |  |  |  |  |  |
|  |  |  |  |  |  |  |  |  |  |  |  |  |  |
|  |  |  |  |  |  |  |  |  |  |  |  |  |  |
|  |  |  |  |  |  |  |  |  |  |  |  |  |  |
|  |  |  |  |  |  |  |  |  |  |  |  |  |  |
