# Supplementary material for: Evaluation of autoantibody signatures in meningioma patients using human proteome arrays
Source: Oncotarget. 2017 Apr 10;8(35):58443–56. doi: 10.18632/oncotarget.16997 (PMC5601665; doi:10.18632/oncotarget.16997)
Supplement: Supplementary file 12 [file oncotarget-08-58443-s012.docx]

**Supplementary Table 5: Gene set enrichment analysis of MGI vs HC**

**Supplementary Table 5.1: Gene details**

| Name of data set: MG1 vs HC |  |  |  |  |  |  |
| --- | --- | --- | --- | --- | --- | --- |
| No. of mapped items: 365 |  |  |  |  |  |  |
| No. of unmapped items: 119 |  |  |  |  |  |  |
| No. of redundant items: 5 |  |  |  |  |  |  |
| Is data set quantitative :NO |  |  |  |  |  |  |
|  |  |  |  |  |  |  |
| Mapped items: |  |  |  |  |  |  |
| Search Term | Enrtez GeneID | Gene Symbol | Description | Alternate names | Chromosome | Map location |
| CRYM | [1428](http://www.ncbi.nlm.nih.gov/gene/1428) | CRYM | crystallin, mu | DFNA40\|THBP | 16 | 16p12.2 |
| KCNMB3 | [27094](http://www.ncbi.nlm.nih.gov/gene/27094) | KCNMB3 | potassium large conductance calcium-activated channel, subfamily M beta member 3 | BKBETA3\|HBETA3\|KCNMB2\|KCNMBL\|SLOBETA3 | 3 | 3q26.3-q27 |
| HOXA5 | [3202](http://www.ncbi.nlm.nih.gov/gene/3202) | HOXA5 | homeobox A5 | HOX1\|HOX1.3\|HOX1C | 7 | 7p15.2 |
| EFCAB2 | [84288](http://www.ncbi.nlm.nih.gov/gene/84288) | EFCAB2 | EF-hand calcium binding domain 2 | - | 1 | 1q44 |
| DOHH | [83475](http://www.ncbi.nlm.nih.gov/gene/83475) | DOHH | deoxyhypusine hydroxylase/monooxygenase | HLRC1\|hDOHH | 19 | 19p13.3 |
| STAT6 | [6778](http://www.ncbi.nlm.nih.gov/gene/6778) | STAT6 | signal transducer and activator of transcription 6, interleukin-4 induced | D12S1644\|IL-4-STAT\|STAT6B\|STAT6C | 12 | 12q13 |
| CD84 | [8832](http://www.ncbi.nlm.nih.gov/gene/8832) | CD84 | CD84 molecule | LY9B\|SLAMF5\|hCD84\|mCD84 | 1 | 1q24 |
| ZHX3 | [23051](http://www.ncbi.nlm.nih.gov/gene/23051) | ZHX3 | zinc fingers and homeoboxes 3 | TIX1 | 20 | 20q12 |
| COQ6 | [51004](http://www.ncbi.nlm.nih.gov/gene/51004) | COQ6 | coenzyme Q6 monooxygenase | CGI10\|COQ10D6 | 14 | 14q24.3 |
| CCNB1 | [891](http://www.ncbi.nlm.nih.gov/gene/891) | CCNB1 | cyclin B1 | CCNB | 5 | 5q12 |
| ADRB2 | [154](http://www.ncbi.nlm.nih.gov/gene/154) | ADRB2 | adrenoceptor beta 2, surface | ADRB2R\|ADRBR\|B2AR\|BAR\|BETA2AR | 5 | 5q31-q32 |
| DRG1 | [4733](http://www.ncbi.nlm.nih.gov/gene/4733) | DRG1 | developmentally regulated GTP binding protein 1 | NEDD3 | 22 | 22q12.2 |
| PROSC | [11212](http://www.ncbi.nlm.nih.gov/gene/11212) | PROSC | proline synthetase co-transcribed homolog (bacterial) | - | 8 | 8p11.2 |
| DNAJB5 | [25822](http://www.ncbi.nlm.nih.gov/gene/25822) | DNAJB5 | DnaJ (Hsp40) homolog, subfamily B, member 5 | Hsc40 | 9 | 9p13.3 |
| GYPE | [2996](http://www.ncbi.nlm.nih.gov/gene/2996) | GYPE | glycophorin E (MNS blood group) | GPE\|MNS\|MiIX | 4 | 4q31.1 |
| OIP5 | [11339](http://www.ncbi.nlm.nih.gov/gene/11339) | OIP5 | Opa interacting protein 5 | 5730547N13Rik\|CT86\|LINT-25\|MIS18B\|MIS18beta\|hMIS18beta | 15 | 15q15.1 |
| SRPX2 | [27286](http://www.ncbi.nlm.nih.gov/gene/27286) | SRPX2 | sushi-repeat containing protein, X-linked 2 | BPP\|CBPS\|PMGX\|RESDX\|SRPUL | X | Xq21.33-q23 |
| AFP | [174](http://www.ncbi.nlm.nih.gov/gene/174) | AFP | alpha-fetoprotein | FETA\|HPAFP | 4 | 4q13.3 |
| STK33 | [65975](http://www.ncbi.nlm.nih.gov/gene/65975) | STK33 | serine/threonine kinase 33 | - | 11 | 11p15.3 |
| FHL2 | [2274](http://www.ncbi.nlm.nih.gov/gene/2274) | FHL2 | four and a half LIM domains 2 | AAG11\|DRAL\|FHL-2\|SLIM-3\|SLIM3 | 2 | 2q12.2 |
| NFE2 | [4778](http://www.ncbi.nlm.nih.gov/gene/4778) | NFE2 | nuclear factor, erythroid 2 | NF-E2\|p45 | 12 | 12q13 |
| RAB3B | [5865](http://www.ncbi.nlm.nih.gov/gene/5865) | RAB3B | RAB3B, member RAS oncogene family | - | 1 | 1p32-p31 |
| STAU2 | [27067](http://www.ncbi.nlm.nih.gov/gene/27067) | STAU2 | staufen double-stranded RNA binding protein 2 | 39K2\|39K3 | 8 | 8q21.11 |
| MRPS7 | [51081](http://www.ncbi.nlm.nih.gov/gene/51081) | MRPS7 | mitochondrial ribosomal protein S7 | MRP-S\|MRP-S7\|RP-S7\|RPMS7\|S7mt\|bMRP27a | 17 | 17q25 |
| DAZAP2 | [9802](http://www.ncbi.nlm.nih.gov/gene/9802) | DAZAP2 | DAZ associated protein 2 | PRTB | 12 | 12q12 |
| CALCOCO2 | [10241](http://www.ncbi.nlm.nih.gov/gene/10241) | CALCOCO2 | calcium binding and coiled-coil domain 2 | NDP52 | 17 | 17q21.32 |
| FST | [10468](http://www.ncbi.nlm.nih.gov/gene/10468) | FST | follistatin | FS | 5 | 5q11.2 |
| NRAS | [4893](http://www.ncbi.nlm.nih.gov/gene/4893) | NRAS | neuroblastoma RAS viral (v-ras) oncogene homolog | ALPS4\|CMNS\|N-ras\|NCMS\|NRAS1\|NS6 | 1 | 1p13.2 |
| TYROBP | [7305](http://www.ncbi.nlm.nih.gov/gene/7305) | TYROBP | TYRO protein tyrosine kinase binding protein | DAP12\|KARAP\|PLOSL | 19 | 19q13.1 |
| RPL10 | [6134](http://www.ncbi.nlm.nih.gov/gene/6134) | RPL10 | ribosomal protein L10 | AUTSX5\|DXS648\|DXS648E\|L10\|NOV\|QM | X | Xq28 |
| KLK1 | [3816](http://www.ncbi.nlm.nih.gov/gene/3816) | KLK1 | kallikrein 1 | KLKR\|Klk6\|hK1 | 19 | 19q13.3 |
| C2orf44 | [80304](http://www.ncbi.nlm.nih.gov/gene/80304) | C2orf44 | chromosome 2 open reading frame 44 | - | 2 | 2p23.3 |
| KRR1 | [11103](http://www.ncbi.nlm.nih.gov/gene/11103) | KRR1 | KRR1, small subunit (SSU) processome component, homolog (yeast) | HRB2\|RIP-1 | 12 | 12q21.2 |
| CSRP1 | [1465](http://www.ncbi.nlm.nih.gov/gene/1465) | CSRP1 | cysteine and glycine-rich protein 1 | CRP\|CRP1\|CSRP\|CYRP\|D1S181E\|HEL-141 | 1 | 1q32 |
| ERAL1 | [26284](http://www.ncbi.nlm.nih.gov/gene/26284) | ERAL1 | Era-like 12S mitochondrial rRNA chaperone 1 | ERA\|ERAL1A\|H-ERA\|HERA-A\|HERA-B | 17 | 17q11.2 |
| OGFOD2 | [79676](http://www.ncbi.nlm.nih.gov/gene/79676) | OGFOD2 | 2-oxoglutarate and iron-dependent oxygenase domain containing 2 | - | 12 | 12q24.31 |
| BHMT2 | [23743](http://www.ncbi.nlm.nih.gov/gene/23743) | BHMT2 | betaine--homocysteine S-methyltransferase 2 | - | 5 | 5q13 |
| GPSM3 | [63940](http://www.ncbi.nlm.nih.gov/gene/63940) | GPSM3 | G-protein signaling modulator 3 | AGS4\|C6orf9\|G18\|G18.1a\|G18.1b\|G18.2\|NG1 | 6 | 6p21.3 |
| APOE | [348](http://www.ncbi.nlm.nih.gov/gene/348) | APOE | apolipoprotein E | AD2\|LDLCQ5\|LPG | 19 | 19q13.2 |
| HLA-DRB5 | [3127](http://www.ncbi.nlm.nih.gov/gene/3127) | HLA-DRB5 | major histocompatibility complex, class II, DR beta 5 | HLA-DRB | 6 | 6p21.3 |
| COX4I1 | [1327](http://www.ncbi.nlm.nih.gov/gene/1327) | COX4I1 | cytochrome c oxidase subunit IV isoform 1 | COX4\|COX4-1\|COXIV | 16 | 16q24.1 |
| HPCAL1 | [3241](http://www.ncbi.nlm.nih.gov/gene/3241) | HPCAL1 | hippocalcin-like 1 | BDR1\|HLP2\|VILIP-3 | 2 | 2p25.1 |
| CD47 | [961](http://www.ncbi.nlm.nih.gov/gene/961) | CD47 | CD47 molecule | IAP\|MER6\|OA3 | 3 | 3q13.1-q13.2 |
| METTL8 | [79828](http://www.ncbi.nlm.nih.gov/gene/79828) | METTL8 | methyltransferase like 8 | TIP | 2 | 2q31.1 |
| UPP2 | [151531](http://www.ncbi.nlm.nih.gov/gene/151531) | UPP2 | uridine phosphorylase 2 | UDRPASE2\|UP2\|UPASE2 | 2 | 2q24.1 |
| AEBP2 | [121536](http://www.ncbi.nlm.nih.gov/gene/121536) | AEBP2 | AE binding protein 2 | - | 12 | 12p12.3 |
| TSPAN17 | [26262](http://www.ncbi.nlm.nih.gov/gene/26262) | TSPAN17 | tetraspanin 17 | FBX23\|FBXO23\|TM4SF17 | 5 | 5q35.3 |
| HSPA2 | [3306](http://www.ncbi.nlm.nih.gov/gene/3306) | HSPA2 | heat shock 70kDa protein 2 | HSP70-2\|HSP70-3 | 14 | 14q24.1 |
| GULP1 | [51454](http://www.ncbi.nlm.nih.gov/gene/51454) | GULP1 | GULP, engulfment adaptor PTB domain containing 1 | CED-6\|CED6\|GULP | 2 | 2q32.3-q33 |
| TM4SF1 | [4071](http://www.ncbi.nlm.nih.gov/gene/4071) | TM4SF1 | transmembrane 4 L six family member 1 | H-L6\|L6\|M3S1\|TAAL6 | 3 | 3q21-q25 |
| GALT | [2592](http://www.ncbi.nlm.nih.gov/gene/2592) | GALT | galactose-1-phosphate uridylyltransferase | - | 9 | 9p13 |
| SP110 | [3431](http://www.ncbi.nlm.nih.gov/gene/3431) | SP110 | SP110 nuclear body protein | IFI41\|IFI75\|IPR1\|VODI | 2 | 2q37.1 |
| RWDD3 | [25950](http://www.ncbi.nlm.nih.gov/gene/25950) | RWDD3 | RWD domain containing 3 | RSUME | 1 | 1p21.3 |
| ELOVL1 | [64834](http://www.ncbi.nlm.nih.gov/gene/64834) | ELOVL1 | ELOVL fatty acid elongase 1 | Ssc1 | 1 | 1p34.2 |
| EDN3 | [1908](http://www.ncbi.nlm.nih.gov/gene/1908) | EDN3 | endothelin 3 | ET-3\|ET3\|HSCR4\|PPET3\|WS4B | 20 | 20q13.2-q13.3 |
| LYPD1 | [116372](http://www.ncbi.nlm.nih.gov/gene/116372) | LYPD1 | LY6/PLAUR domain containing 1 | LYPDC1\|PHTS | 2 | 2q21.2 |
| LRRC20 | [55222](http://www.ncbi.nlm.nih.gov/gene/55222) | LRRC20 | leucine rich repeat containing 20 | - | 10 | 10q22.1 |
| CARHSP1 | [23589](http://www.ncbi.nlm.nih.gov/gene/23589) | CARHSP1 | calcium regulated heat stable protein 1, 24kDa | CRHSP-24\|CSDC1 | 16 | 16p13.2 |
| NAPSA | [9476](http://www.ncbi.nlm.nih.gov/gene/9476) | NAPSA | napsin A aspartic peptidase | KAP\|Kdap\|NAP1\|NAPA\|SNAPA | 19 | 19q13.33 |
| CKS2 | [1164](http://www.ncbi.nlm.nih.gov/gene/1164) | CKS2 | CDC28 protein kinase regulatory subunit 2 | CKSHS2 | 9 | 9q22 |
| FUBP3 | [8939](http://www.ncbi.nlm.nih.gov/gene/8939) | FUBP3 | far upstream element (FUSE) binding protein 3 | FBP3 | 9 | 9q34.11 |
| IGLL1 | [3543](http://www.ncbi.nlm.nih.gov/gene/3543) | IGLL1 | immunoglobulin lambda-like polypeptide 1 | 14.1\|AGM2\|CD179b\|IGL1\|IGL5\|IGLJ14.1\|IGLL\|IGO\|IGVPB\|VPREB2 | 22 | 22q11.23 |
| GSTP1 | [2950](http://www.ncbi.nlm.nih.gov/gene/2950) | GSTP1 | glutathione S-transferase pi 1 | DFN7\|FAEES3\|GST3\|GSTP\|HEL-S-22\|PI | 11 | 11q13 |
| CAMK2N1 | [55450](http://www.ncbi.nlm.nih.gov/gene/55450) | CAMK2N1 | calcium/calmodulin-dependent protein kinase II inhibitor 1 | PRO1489 | 1 | 1p36.12 |
| RPS6KA2 | [6196](http://www.ncbi.nlm.nih.gov/gene/6196) | RPS6KA2 | ribosomal protein S6 kinase, 90kDa, polypeptide 2 | HU-2\|MAPKAPK1C\|RSK\|RSK3\|S6K-alpha\|S6K-alpha2\|p90-RSK3\|pp90RSK3 | 6 | 6q27 |
| CYBB | [1536](http://www.ncbi.nlm.nih.gov/gene/1536) | CYBB | cytochrome b-245, beta polypeptide | AMCBX2\|CGD\|GP91-1\|GP91-PHOX\|GP91PHOX\|NOX2\|p91-PHOX | X | Xp21.1 |
| CORO1A | [11151](http://www.ncbi.nlm.nih.gov/gene/11151) | CORO1A | coronin, actin binding protein, 1A | CLABP\|CLIPINA\|HCORO1\|IMD8\|TACO\|p57 | 16 | 16p11.2 |
| FUBP1 | [8880](http://www.ncbi.nlm.nih.gov/gene/8880) | FUBP1 | far upstream element (FUSE) binding protein 1 | FBP\|FUBP | 1 | 1p31.1 |
| ATAD2 | [29028](http://www.ncbi.nlm.nih.gov/gene/29028) | ATAD2 | ATPase family, AAA domain containing 2 | ANCCA\|CT137\|PRO2000 | 8 | 8q24.13 |
| FAM63A | [55793](http://www.ncbi.nlm.nih.gov/gene/55793) | FAM63A | family with sequence similarity 63, member A | - | 1 | 1q21.3 |
| CAPRIN2 | [65981](http://www.ncbi.nlm.nih.gov/gene/65981) | CAPRIN2 | caprin family member 2 | C1QDC1\|EEG-1\|EEG1\|RNG140 | 12 | 12p11 |
| FBXO31 | [79791](http://www.ncbi.nlm.nih.gov/gene/79791) | FBXO31 | F-box protein 31 | FBX14\|FBXO14\|Fbx31\|pp2386 | 16 | 16q24.2 |
| FAM122C | [159091](http://www.ncbi.nlm.nih.gov/gene/159091) | FAM122C | family with sequence similarity 122C | - | X | Xq26.3 |
| RPS7 | [6201](http://www.ncbi.nlm.nih.gov/gene/6201) | RPS7 | ribosomal protein S7 | DBA8\|S7 | 2 | 2p25 |
| TIMP1 | [7076](http://www.ncbi.nlm.nih.gov/gene/7076) | TIMP1 | TIMP metallopeptidase inhibitor 1 | CLGI\|EPA\|EPO\|HCI\|TIMP | X | Xp11.3-p11.23 |
| STATH | [6779](http://www.ncbi.nlm.nih.gov/gene/6779) | STATH | statherin | STR | 4 | 4q13.3 |
| OLR1 | [4973](http://www.ncbi.nlm.nih.gov/gene/4973) | OLR1 | oxidized low density lipoprotein (lectin-like) receptor 1 | CLEC8A\|LOX1\|LOXIN\|SCARE1\|SLOX1 | 12 | 12p13.2-p12.3 |
| NUDT22 | [84304](http://www.ncbi.nlm.nih.gov/gene/84304) | NUDT22 | nudix (nucleoside diphosphate linked moiety X)-type motif 22 | - | 11 | 11q13.1 |
| PPP2R4 | [5524](http://www.ncbi.nlm.nih.gov/gene/5524) | PPP2R4 | protein phosphatase 2A activator, regulatory subunit 4 | PP2A\|PR53\|PTPA | 9 | 9q34 |
| SLC39A9 | [55334](http://www.ncbi.nlm.nih.gov/gene/55334) | SLC39A9 | solute carrier family 39, member 9 | ZIP-9\|ZIP9 | 14 | 14q24.1 |
| ADRBK1 | [156](http://www.ncbi.nlm.nih.gov/gene/156) | ADRBK1 | adrenergic, beta, receptor kinase 1 | BARK1\|BETA-ARK1\|GRK2 | 11 | 11q13.1 |
| QDPR | [5860](http://www.ncbi.nlm.nih.gov/gene/5860) | QDPR | quinoid dihydropteridine reductase | DHPR\|PKU2\|SDR33C1 | 4 | 4p15.31 |
| IFIT3 | [3437](http://www.ncbi.nlm.nih.gov/gene/3437) | IFIT3 | interferon-induced protein with tetratricopeptide repeats 3 | CIG-49\|GARG-49\|IFI60\|IFIT4\|IRG2\|ISG60\|P60\|RIG-G | 10 | 10q24 |
| ROPN1L | [83853](http://www.ncbi.nlm.nih.gov/gene/83853) | ROPN1L | rhophilin associated tail protein 1-like | ASP\|RSPH11 | 5 | 5p15.2 |
| RHOA | [387](http://www.ncbi.nlm.nih.gov/gene/387) | RHOA | ras homolog family member A | ARH12\|ARHA\|RHO12\|RHOH12 | 3 | 3p21.3 |
| CD96 | [10225](http://www.ncbi.nlm.nih.gov/gene/10225) | CD96 | CD96 molecule | TACTILE | 3 | 3q13.13-q13.2 |
| LAYN | [143903](http://www.ncbi.nlm.nih.gov/gene/143903) | LAYN | layilin | - | 11 | 11q23.1 |
| Dlx5 | [1749](http://www.ncbi.nlm.nih.gov/gene/1749) | DLX5 | distal-less homeobox 5 | SHFM1D | 7 | 7q22 |
| GADD45A | [1647](http://www.ncbi.nlm.nih.gov/gene/1647) | GADD45A | growth arrest and DNA-damage-inducible, alpha | DDIT1\|GADD45 | 1 | 1p31.2 |
| RAP1GDS1 | [5910](http://www.ncbi.nlm.nih.gov/gene/5910) | RAP1GDS1 | RAP1, GTP-GDP dissociation stimulator 1 | GDS1\|SmgGDS | 4 | 4q23-q25 |
| ATG3 | [64422](http://www.ncbi.nlm.nih.gov/gene/64422) | ATG3 | autophagy related 3 | APG3\|APG3-LIKE\|APG3L\|PC3-96 | 3 | 3q13.2 |
| TIPIN | [54962](http://www.ncbi.nlm.nih.gov/gene/54962) | TIPIN | TIMELESS interacting protein | - | 15 | 15q22.31 |
| HPX | [3263](http://www.ncbi.nlm.nih.gov/gene/3263) | HPX | hemopexin | HX | 11 | 11p15.5-p15.4 |
| SGK2 | [10110](http://www.ncbi.nlm.nih.gov/gene/10110) | SGK2 | serum/glucocorticoid regulated kinase 2 | H-SGK2\|dJ138B7.2 | 20 | 20q13.2 |
| NECAP2 | [55707](http://www.ncbi.nlm.nih.gov/gene/55707) | NECAP2 | NECAP endocytosis associated 2 | - | 1 | 1p36.13 |
| HLA-DOB | [3112](http://www.ncbi.nlm.nih.gov/gene/3112) | HLA-DOB | major histocompatibility complex, class II, DO beta | DOB | 6 | 6p21.3 |
| SPAG16 | [79582](http://www.ncbi.nlm.nih.gov/gene/79582) | SPAG16 | sperm associated antigen 16 | PF20\|WDR29 | 2 | 2q34 |
| CHD4 | [1108](http://www.ncbi.nlm.nih.gov/gene/1108) | CHD4 | chromodomain helicase DNA binding protein 4 | Mi-2b\|Mi2-BETA | 12 | 12p13 |
| ST6GALNAC6 | [30815](http://www.ncbi.nlm.nih.gov/gene/30815) | ST6GALNAC6 | ST6 (alpha-N-acetyl-neuraminyl-2,3-beta-galactosyl-1,3)-N-acetylgalactosaminide alpha-2,6-sialyltransferase 6 | SIAT7-F\|SIAT7F\|ST6GALNACVI | 9 | 9q34.11 |
| DOK1 | [1796](http://www.ncbi.nlm.nih.gov/gene/1796) | DOK1 | docking protein 1, 62kDa (downstream of tyrosine kinase 1) | P62DOK | 2 | 2p13 |
| MRPS25 | [64432](http://www.ncbi.nlm.nih.gov/gene/64432) | MRPS25 | mitochondrial ribosomal protein S25 | MRP-S25\|RPMS25 | 3 | 3p25 |
| PSMD14 | [10213](http://www.ncbi.nlm.nih.gov/gene/10213) | PSMD14 | proteasome (prosome, macropain) 26S subunit, non-ATPase, 14 | PAD1\|POH1\|RPN11 | 2 | 2q24.2 |
| SLC5A6 | [8884](http://www.ncbi.nlm.nih.gov/gene/8884) | SLC5A6 | solute carrier family 5 (sodium/multivitamin and iodide cotransporter), member 6 | SMVT | 2 | 2p23 |
| IMPDH2 | [3615](http://www.ncbi.nlm.nih.gov/gene/3615) | IMPDH2 | IMP (inosine 5'-monophosphate) dehydrogenase 2 | IMPD2\|IMPDH-II | 3 | 3p21.2 |
| GH2 | [2689](http://www.ncbi.nlm.nih.gov/gene/2689) | GH2 | growth hormone 2 | GH-V\|GHL\|GHV\|hGH-V | 17 | 17q24.2 |
| PANK1 | [53354](http://www.ncbi.nlm.nih.gov/gene/53354) | PANK1 | pantothenate kinase 1 | PANK | 10 | 10q23.31 |
| GLYAT | [10249](http://www.ncbi.nlm.nih.gov/gene/10249) | GLYAT | glycine-N-acyltransferase | ACGNAT\|CAT\|GAT | 11 | 11q12.1 |
| XPR1 | [9213](http://www.ncbi.nlm.nih.gov/gene/9213) | XPR1 | xenotropic and polytropic retrovirus receptor 1 | SYG1\|X3 | 1 | 1q25.1 |
| DHX9 | [1660](http://www.ncbi.nlm.nih.gov/gene/1660) | DHX9 | DEAH (Asp-Glu-Ala-His) box helicase 9 | DDX9\|LKP\|NDH2\|NDHII\|RHA | 1 | 1q25 |
| GNB2L1 | [10399](http://www.ncbi.nlm.nih.gov/gene/10399) | GNB2L1 | guanine nucleotide binding protein (G protein), beta polypeptide 2-like 1 | Gnb2-rs1\|H12.3\|HLC-7\|PIG21\|RACK1 | 5 | 5q35.3 |
| MRTO4 | [51154](http://www.ncbi.nlm.nih.gov/gene/51154) | MRTO4 | mRNA turnover 4 homolog (S. cerevisiae) | C1orf33\|MRT4\|dJ657E11.4 | 1 | 1p36.13 |
| CDO1 | [1036](http://www.ncbi.nlm.nih.gov/gene/1036) | CDO1 | cysteine dioxygenase type 1 | - | 5 | 5q23.2 |
| BCAS2 | [10286](http://www.ncbi.nlm.nih.gov/gene/10286) | BCAS2 | breast carcinoma amplified sequence 2 | DAM1\|SPF27\|Snt309 | 1 | 1p13.2 |
| FAM58A | [92002](http://www.ncbi.nlm.nih.gov/gene/92002) | FAM58A | family with sequence similarity 58, member A | STAR | X | Xq28 |
| CYB5R1 | [51706](http://www.ncbi.nlm.nih.gov/gene/51706) | CYB5R1 | cytochrome b5 reductase 1 | B5R.1\|B5R1\|B5R2\|NQO3A2\|humb5R2 | 1 | 1q32.1 |
| FGF7 | [2252](http://www.ncbi.nlm.nih.gov/gene/2252) | FGF7 | fibroblast growth factor 7 | HBGF-7\|KGF | 15 | 15q21.2 |
| CDC34 | [997](http://www.ncbi.nlm.nih.gov/gene/997) | CDC34 | cell division cycle 34 | E2-CDC34\|UBC3\|UBCH3\|UBE2R1 | 19 | 19p13.3 |
| CLDN4 | [1364](http://www.ncbi.nlm.nih.gov/gene/1364) | CLDN4 | claudin 4 | CPE-R\|CPER\|CPETR\|CPETR1\|WBSCR8\|hCPE-R | 7 | 7q11.23 |
| ARL2BP | [23568](http://www.ncbi.nlm.nih.gov/gene/23568) | ARL2BP | ADP-ribosylation factor-like 2 binding protein | BART\|BART1\|RP66 | 16 | 16q13 |
| SYNPR | [132204](http://www.ncbi.nlm.nih.gov/gene/132204) | SYNPR | synaptoporin | SPO | 3 | 3p14.2 |
| RAB39B | [116442](http://www.ncbi.nlm.nih.gov/gene/116442) | RAB39B | RAB39B, member RAS oncogene family | MRX72 | X | Xq28 |
| LCAT | [3931](http://www.ncbi.nlm.nih.gov/gene/3931) | LCAT | lecithin-cholesterol acyltransferase | - | 16 | 16q22.1 |
| VSNL1 | [7447](http://www.ncbi.nlm.nih.gov/gene/7447) | VSNL1 | visinin-like 1 | HLP3\|HPCAL3\|HUVISL1\|VILIP\|VILIP-1 | 2 | 2p24.3 |
| EPS8L1 | [54869](http://www.ncbi.nlm.nih.gov/gene/54869) | EPS8L1 | EPS8-like 1 | DRC3\|EPS8R1 | 19 | 19q13.42 |
| PTN | [5764](http://www.ncbi.nlm.nih.gov/gene/5764) | PTN | pleiotrophin | HARP\|HBGF8\|HBNF\|NEGF1 | 7 | 7q33 |
| RRAGB | [10325](http://www.ncbi.nlm.nih.gov/gene/10325) | RRAGB | Ras-related GTP binding B | RAGB\|bA465E19.1 | X | Xp11.21 |
| CTNNAL1 | [8727](http://www.ncbi.nlm.nih.gov/gene/8727) | CTNNAL1 | catenin (cadherin-associated protein), alpha-like 1 | ACRP\|CLLP\|alpha-CATU | 9 | 9q31.2 |
| FBXL18 | [80028](http://www.ncbi.nlm.nih.gov/gene/80028) | FBXL18 | F-box and leucine-rich repeat protein 18 | Fbl18 | 7 | 7p22.2 |
| SFN | [2810](http://www.ncbi.nlm.nih.gov/gene/2810) | SFN | stratifin | YWHAS | 1 | 1p36.11 |
| ANP32E | [81611](http://www.ncbi.nlm.nih.gov/gene/81611) | ANP32E | acidic (leucine-rich) nuclear phosphoprotein 32 family, member E | LANP-L\|LANPL | 1 | 1q21.2 |
| FN3K | [64122](http://www.ncbi.nlm.nih.gov/gene/64122) | FN3K | fructosamine 3 kinase | - | 17 | 17q25.3 |
| SEPT1 | [1731](http://www.ncbi.nlm.nih.gov/gene/1731) | SEPT1 | septin 1 | DIFF6\|LARP\|PNUTL3\|SEP1 | 16 | 16p11.1 |
| NHEJ1 | [79840](http://www.ncbi.nlm.nih.gov/gene/79840) | NHEJ1 | nonhomologous end-joining factor 1 | XLF | 2 | 2q35 |
| NME1 | [4830](http://www.ncbi.nlm.nih.gov/gene/4830) | NME1 | NME/NM23 nucleoside diphosphate kinase 1 | AWD\|GAAD\|NB\|NBS\|NDKA\|NDPK-A\|NDPKA\|NM23\|NM23-H1 | 17 | 17q21.3 |
| STARD10 | [10809](http://www.ncbi.nlm.nih.gov/gene/10809) | STARD10 | StAR-related lipid transfer (START) domain containing 10 | NY-CO-28\|PCTP2\|SDCCAG28 | 11 | 11q13 |
| NMRAL1 | [57407](http://www.ncbi.nlm.nih.gov/gene/57407) | NMRAL1 | NmrA-like family domain containing 1 | HSCARG\|SDR48A1 | 16 | 16p13.3 |
| ADD1 | [118](http://www.ncbi.nlm.nih.gov/gene/118) | ADD1 | adducin 1 (alpha) | ADDA | 4 | 4p16.3 |
| ACTL6B | [51412](http://www.ncbi.nlm.nih.gov/gene/51412) | ACTL6B | actin-like 6B | ACTL6\|BAF53B | 7 | 7q22 |
| CAMK4 | [814](http://www.ncbi.nlm.nih.gov/gene/814) | CAMK4 | calcium/calmodulin-dependent protein kinase IV | CaMK IV\|CaMK-GR\|IV\|caMK | 5 | 5q21.3 |
| RPS15 | [6209](http://www.ncbi.nlm.nih.gov/gene/6209) | RPS15 | ribosomal protein S15 | RIG\|S15 | 19 | 19p13.3 |
| TEX264 | [51368](http://www.ncbi.nlm.nih.gov/gene/51368) | TEX264 | testis expressed 264 | ZSIG11 | 3 | 3p21.31 |
| DCK | [1633](http://www.ncbi.nlm.nih.gov/gene/1633) | DCK | deoxycytidine kinase | - | 4 | 4q13.3-q21.1 |
| ZNF641 | [121274](http://www.ncbi.nlm.nih.gov/gene/121274) | ZNF641 | zinc finger protein 641 | - | 12 | 12q13.11 |
| DHFR | [1719](http://www.ncbi.nlm.nih.gov/gene/1719) | DHFR | dihydrofolate reductase | DHFRP1\|DYR | 5 | 5q11.2-q13.2 |
| FBXO3 | [26273](http://www.ncbi.nlm.nih.gov/gene/26273) | FBXO3 | F-box protein 3 | FBA\|FBX3 | 11 | 11p13 |
| TMEM185A | [84548](http://www.ncbi.nlm.nih.gov/gene/84548) | TMEM185A | transmembrane protein 185A | CXorf13\|FAM11A\|FRAXF\|ee3 | X | Xq28 |
| C6orf141 | [135398](http://www.ncbi.nlm.nih.gov/gene/135398) | C6orf141 | chromosome 6 open reading frame 141 | - | 6 | 6p12.3 |
| HN1 | [51155](http://www.ncbi.nlm.nih.gov/gene/51155) | HN1 | hematological and neurological expressed 1 | ARM2\|HN1A | 17 | 17q25.1 |
| ANAPC11 | [51529](http://www.ncbi.nlm.nih.gov/gene/51529) | ANAPC11 | anaphase promoting complex subunit 11 | APC11\|Apc11p | 17 | 17q25.3 |
| DDX58 | [23586](http://www.ncbi.nlm.nih.gov/gene/23586) | DDX58 | DEAD (Asp-Glu-Ala-Asp) box polypeptide 58 | RIG-I\|RIGI\|RLR-1 | 9 | 9p12 |
| ARPC3 | [10094](http://www.ncbi.nlm.nih.gov/gene/10094) | ARPC3 | actin related protein 2/3 complex, subunit 3, 21kDa | ARC21\|p21-Arc | 12 | 12q24.11 |
| SPP1 | [6696](http://www.ncbi.nlm.nih.gov/gene/6696) | SPP1 | secreted phosphoprotein 1 | BNSP\|BSPI\|ETA-1\|OPN | 4 | 4q22.1 |
| CLGN | [1047](http://www.ncbi.nlm.nih.gov/gene/1047) | CLGN | calmegin | - | 4 | 4q28.3-q31.1 |
| RAB24 | [53917](http://www.ncbi.nlm.nih.gov/gene/53917) | RAB24 | RAB24, member RAS oncogene family | - | 5 | 5q35.3 |
| MTL5 | [9633](http://www.ncbi.nlm.nih.gov/gene/9633) | MTL5 | metallothionein-like 5, testis-specific (tesmin) | CXCDC2\|MTLT\|TESMIN | 11 | 11q13.2-q13.3 |
| SULT1E1 | [6783](http://www.ncbi.nlm.nih.gov/gene/6783) | SULT1E1 | sulfotransferase family 1E, estrogen-preferring, member 1 | EST\|EST-1\|ST1E1\|STE | 4 | 4q13.1 |
| SH2D1B | [117157](http://www.ncbi.nlm.nih.gov/gene/117157) | SH2D1B | SH2 domain containing 1B | EAT2 | 1 | 1q23.3 |
| KLF11 | [8462](http://www.ncbi.nlm.nih.gov/gene/8462) | KLF11 | Kruppel-like factor 11 | FKLF\|FKLF1\|MODY7\|TIEG2\|Tieg3 | 2 | 2p25 |
| HLA-DRB3 | [3125](http://www.ncbi.nlm.nih.gov/gene/3125) | HLA-DRB3 | major histocompatibility complex, class II, DR beta 3 | HLA-DR3B | 6 | 6p21.3 |
| NOSIP | [51070](http://www.ncbi.nlm.nih.gov/gene/51070) | NOSIP | nitric oxide synthase interacting protein | - | 19 | 19q13.33 |
| EIF4EBP3 | [8637](http://www.ncbi.nlm.nih.gov/gene/8637) | EIF4EBP3 | eukaryotic translation initiation factor 4E binding protein 3 | 4E-BP3\|4EBP3 | 5 | 5q31.3 |
| PTS | [5805](http://www.ncbi.nlm.nih.gov/gene/5805) | PTS | 6-pyruvoyltetrahydropterin synthase | PTPS | 11 | 11q22.3 |
| RBKS | [64080](http://www.ncbi.nlm.nih.gov/gene/64080) | RBKS | ribokinase | RBSK | 2 | 2p23.3 |
| CCNH | [902](http://www.ncbi.nlm.nih.gov/gene/902) | CCNH | cyclin H | CAK\|CycH\|p34\|p37 | 5 | 5q13.3-q14 |
| AAK1 | [22848](http://www.ncbi.nlm.nih.gov/gene/22848) | AAK1 | AP2 associated kinase 1 | - | 2 | 2p14 |
| ARHGDIG | [398](http://www.ncbi.nlm.nih.gov/gene/398) | ARHGDIG | Rho GDP dissociation inhibitor (GDI) gamma | RHOGDI-3 | 16 | 16p13.3 |
| APEX1 | [328](http://www.ncbi.nlm.nih.gov/gene/328) | APEX1 | APEX nuclease (multifunctional DNA repair enzyme) 1 | APE\|APE1\|APEN\|APEX\|APX\|HAP1\|REF1 | 14 | 14q11.2 |
| ZNF761 | [388561](http://www.ncbi.nlm.nih.gov/gene/388561) | ZNF761 | zinc finger protein 761 | ZNF468 | 19 | 19q13.42 |
| CCT4 | [10575](http://www.ncbi.nlm.nih.gov/gene/10575) | CCT4 | chaperonin containing TCP1, subunit 4 (delta) | CCT-DELTA\|Cctd\|SRB | 2 | 2p15 |
| SERTAD3 | [29946](http://www.ncbi.nlm.nih.gov/gene/29946) | SERTAD3 | SERTA domain containing 3 | RBT1 | 19 | 19q13.2 |
| CXXC5 | [51523](http://www.ncbi.nlm.nih.gov/gene/51523) | CXXC5 | CXXC finger protein 5 | CF5\|RINF\|WID | 5 | 5q31.2 |
| FNIP1 | [96459](http://www.ncbi.nlm.nih.gov/gene/96459) | FNIP1 | folliculin interacting protein 1 | - | 5 | 5q23.3 |
| ZNF587 | [84914](http://www.ncbi.nlm.nih.gov/gene/84914) | ZNF587 | zinc finger protein 587 | ZF6 | 19 | 19q13.43 |
| CCR10 | [2826](http://www.ncbi.nlm.nih.gov/gene/2826) | CCR10 | chemokine (C-C motif) receptor 10 | GPR2 | 17 | 17q21.1-q21.3 |
| MRPL53 | [116540](http://www.ncbi.nlm.nih.gov/gene/116540) | MRPL53 | mitochondrial ribosomal protein L53 | L53MT | 2 | 2p13.1 |
| ABLIM1 | [3983](http://www.ncbi.nlm.nih.gov/gene/3983) | ABLIM1 | actin binding LIM protein 1 | ABLIM\|LIMAB1\|LIMATIN\|abLIM-1 | 10 | 10q25 |
| ARIH2 | [10425](http://www.ncbi.nlm.nih.gov/gene/10425) | ARIH2 | ariadne RBR E3 ubiquitin protein ligase 2 | ARI2\|TRIAD1 | 3 | 3p21 |
| BAG5 | [9529](http://www.ncbi.nlm.nih.gov/gene/9529) | BAG5 | BCL2-associated athanogene 5 | BAG-5 | 14 | 14q32.33 |
| MAPK3 | [5595](http://www.ncbi.nlm.nih.gov/gene/5595) | MAPK3 | mitogen-activated protein kinase 3 | ERK-1\|ERK1\|ERT2\|HS44KDAP\|HUMKER1A\|P44ERK1\|P44MAPK\|PRKM3\|p44-ERK1\|p44-MAPK | 16 | 16p11.2 |
| TIRAP | [114609](http://www.ncbi.nlm.nih.gov/gene/114609) | TIRAP | toll-interleukin 1 receptor (TIR) domain containing adaptor protein | BACTS1\|Mal\|MyD88-2\|wyatt | 11 | 11q24.2 |
| CDCP1 | [64866](http://www.ncbi.nlm.nih.gov/gene/64866) | CDCP1 | CUB domain containing protein 1 | CD318\|SIMA135\|TRASK | 3 | 3p21.31 |
| MYOT | [9499](http://www.ncbi.nlm.nih.gov/gene/9499) | MYOT | myotilin | LGMD1\|LGMD1A\|MFM3\|TTID\|TTOD | 5 | 5q31 |
| ARF5 | [381](http://www.ncbi.nlm.nih.gov/gene/381) | ARF5 | ADP-ribosylation factor 5 | - | 7 | 7q31.3 |
| YWHAB | [7529](http://www.ncbi.nlm.nih.gov/gene/7529) | YWHAB | tyrosine 3-monooxygenase/tryptophan 5-monooxygenase activation protein, beta | GW128\|HEL-S-1\|HS1\|KCIP-1\|YWHAA | 20 | 20q13.1 |
| DBNL | [28988](http://www.ncbi.nlm.nih.gov/gene/28988) | DBNL | drebrin-like | ABP1\|HIP-55\|HIP55\|SH3P7 | 7 | 7p13 |
| TCOF1 | [6949](http://www.ncbi.nlm.nih.gov/gene/6949) | TCOF1 | Treacher Collins-Franceschetti syndrome 1 | MFD1\|TCS\|TCS1\|treacle | 5 | 5q32 |
| GGT6 | [124975](http://www.ncbi.nlm.nih.gov/gene/124975) | GGT6 | gamma-glutamyltransferase 6 | - | 17 | 17p13.2 |
| RPL14 | [9045](http://www.ncbi.nlm.nih.gov/gene/9045) | RPL14 | ribosomal protein L14 | CAG-ISL-7\|CTG-B33\|L14\|RL14\|hRL14 | 3 | 3p22-p21.2 |
| CALN1 | [83698](http://www.ncbi.nlm.nih.gov/gene/83698) | CALN1 | calneuron 1 | CABP8 | 7 | 7q11 |
| C1QC | [714](http://www.ncbi.nlm.nih.gov/gene/714) | C1QC | complement component 1, q subcomponent, C chain | C1Q-C\|C1QG | 1 | 1p36.11 |
| C1orf21 | [81563](http://www.ncbi.nlm.nih.gov/gene/81563) | C1orf21 | chromosome 1 open reading frame 21 | PIG13 | 1 | 1q25 |
| RNF32 | [140545](http://www.ncbi.nlm.nih.gov/gene/140545) | RNF32 | ring finger protein 32 | FKSG33\|HSD15\|LMBR2 | 7 | 7q36 |
| TTC1 | [7265](http://www.ncbi.nlm.nih.gov/gene/7265) | TTC1 | tetratricopeptide repeat domain 1 | TPR1 | 5 | 5q33.3 |
| USP15 | [9958](http://www.ncbi.nlm.nih.gov/gene/9958) | USP15 | ubiquitin specific peptidase 15 | UNPH-2\|UNPH4 | 12 | 12q14 |
| MORC4 | [79710](http://www.ncbi.nlm.nih.gov/gene/79710) | MORC4 | MORC family CW-type zinc finger 4 | ZCW4\|ZCWCC2\|dJ75H8.2 | X | Xq22.3 |
| UGDH | [7358](http://www.ncbi.nlm.nih.gov/gene/7358) | UGDH | UDP-glucose 6-dehydrogenase | GDH\|UDP-GlcDH\|UDPGDH\|UGD | 4 | 4p15.1 |
| ASF1A | [25842](http://www.ncbi.nlm.nih.gov/gene/25842) | ASF1A | anti-silencing function 1A histone chaperone | CGI-98\|CIA\|HSPC146 | 6 | 6q22.31 |
| MIPOL1 | [145282](http://www.ncbi.nlm.nih.gov/gene/145282) | MIPOL1 | mirror-image polydactyly 1 | - | 14 | 14q13.3 |
| ENOPH1 | [58478](http://www.ncbi.nlm.nih.gov/gene/58478) | ENOPH1 | enolase-phosphatase 1 | E1\|MASA\|MST145\|mtnC | 4 | 4q21.22 |
| PTPN11 | [5781](http://www.ncbi.nlm.nih.gov/gene/5781) | PTPN11 | protein tyrosine phosphatase, non-receptor type 11 | BPTP3\|CFC\|NS1\|PTP-1D\|PTP2C\|SH-PTP2\|SH-PTP3\|SHP2 | 12 | 12q24 |
| ZFYVE19 | [84936](http://www.ncbi.nlm.nih.gov/gene/84936) | ZFYVE19 | zinc finger, FYVE domain containing 19 | MPFYVE | 15 | 15q15.1 |
| PRKRA | [8575](http://www.ncbi.nlm.nih.gov/gene/8575) | PRKRA | protein kinase, interferon-inducible double stranded RNA dependent activator | DYT16\|PACT\|RAX | 2 | 2q31.2 |
| SMYD3 | [64754](http://www.ncbi.nlm.nih.gov/gene/64754) | SMYD3 | SET and MYND domain containing 3 | KMT3E\|ZMYND1\|ZNFN3A1\|bA74P14.1 | 1 | 1q44 |
| MCM5 | [4174](http://www.ncbi.nlm.nih.gov/gene/4174) | MCM5 | minichromosome maintenance complex component 5 | CDC46\|P1-CDC46 | 22 | 22q13.1 |
| C14orf119 | [55017](http://www.ncbi.nlm.nih.gov/gene/55017) | C14orf119 | chromosome 14 open reading frame 119 | - | 14 | 14q11.2 |
| DIABLO | [56616](http://www.ncbi.nlm.nih.gov/gene/56616) | DIABLO | diablo, IAP-binding mitochondrial protein | DFNA64\|SMAC | 12 | 12q24.31 |
| QTRT1 | [81890](http://www.ncbi.nlm.nih.gov/gene/81890) | QTRT1 | queuine tRNA-ribosyltransferase 1 | FP3235\|TGT\|TGUT | 19 | 19p13.3 |
| PDLIM3 | [27295](http://www.ncbi.nlm.nih.gov/gene/27295) | PDLIM3 | PDZ and LIM domain 3 | ALP | 4 | 4q35 |
| RBBP7 | [5931](http://www.ncbi.nlm.nih.gov/gene/5931) | RBBP7 | retinoblastoma binding protein 7 | RbAp46 | X | Xp22.2 |
| PI16 | [221476](http://www.ncbi.nlm.nih.gov/gene/221476) | PI16 | peptidase inhibitor 16 | CRISP9\|MSMBBP\|PSPBP | 6 | 6p21.2 |
| CKAP2 | [26586](http://www.ncbi.nlm.nih.gov/gene/26586) | CKAP2 | cytoskeleton associated protein 2 | LB1\|TMAP\|se20-10 | 13 | 13q14 |
| GABRA5 | [2558](http://www.ncbi.nlm.nih.gov/gene/2558) | GABRA5 | gamma-aminobutyric acid (GABA) A receptor, alpha 5 | - | 15 | 15q12 |
| NAGK | [55577](http://www.ncbi.nlm.nih.gov/gene/55577) | NAGK | N-acetylglucosamine kinase | GNK\|HSA242910 | 2 | 2p13.3 |
| PRH2 | [5555](http://www.ncbi.nlm.nih.gov/gene/5555) | PRH2 | proline-rich protein HaeIII subfamily 2 | PIF-S\|PRH1\|PRP-1/PRP-2\|Pr\|db-s\|pa\|pr1/Pr2 | 12 | 12p13.2 |
| CPNE4 | [131034](http://www.ncbi.nlm.nih.gov/gene/131034) | CPNE4 | copine IV | COPN4\|CPN4 | 3 | 3q22.1 |
| MEIS2 | [4212](http://www.ncbi.nlm.nih.gov/gene/4212) | MEIS2 | Meis homeobox 2 | HsT18361\|MRG1 | 15 | 15q14 |
| MCTS1 | [28985](http://www.ncbi.nlm.nih.gov/gene/28985) | MCTS1 | malignant T cell amplified sequence 1 | MCT-1\|MCT1 | X | Xq24 |
| DERL1 | [79139](http://www.ncbi.nlm.nih.gov/gene/79139) | DERL1 | derlin 1 | DER-1\|DER1 | 8 | 8q24.13 |
| HIGD2A | [192286](http://www.ncbi.nlm.nih.gov/gene/192286) | HIGD2A | HIG1 hypoxia inducible domain family, member 2A | RCF1b | 5 | 5q35.2 |
| RPL22 | [6146](http://www.ncbi.nlm.nih.gov/gene/6146) | RPL22 | ribosomal protein L22 | EAP\|HBP15\|HBP15/L22\|L22 | 1 | 1p36.31 |
| SRPK2 | [6733](http://www.ncbi.nlm.nih.gov/gene/6733) | SRPK2 | SRSF protein kinase 2 | SFRSK2 | 7 | 7q22-q31.1 |
| FAM81A | [145773](http://www.ncbi.nlm.nih.gov/gene/145773) | FAM81A | family with sequence similarity 81, member A | - | 15 | 15q22.2 |
| HSPB8 | [26353](http://www.ncbi.nlm.nih.gov/gene/26353) | HSPB8 | heat shock 22kDa protein 8 | CMT2L\|DHMN2\|E2IG1\|H11\|HMN2\|HMN2A\|HSP22 | 12 | 12q24.23 |
| ATP6V1E2 | [90423](http://www.ncbi.nlm.nih.gov/gene/90423) | ATP6V1E2 | ATPase, H+ transporting, lysosomal 31kDa, V1 subunit E2 | ATP6E1\|ATP6EL2\|ATP6V1EL2\|VMA4 | 2 | 2p21 |
| C14orf80 | [283643](http://www.ncbi.nlm.nih.gov/gene/283643) | C14orf80 | chromosome 14 open reading frame 80 | - | 14 | 14q32.33 |
| HBG2 | [3048](http://www.ncbi.nlm.nih.gov/gene/3048) | HBG2 | hemoglobin, gamma G | HBG-T1\|TNCY | 11 | 11p15.5 |
| C11orf49 | [79096](http://www.ncbi.nlm.nih.gov/gene/79096) | C11orf49 | chromosome 11 open reading frame 49 | - | 11 | 11p11.2 |
| SERBP1 | [26135](http://www.ncbi.nlm.nih.gov/gene/26135) | SERBP1 | SERPINE1 mRNA binding protein 1 | CHD3IP\|HABP4L\|PAI-RBP1\|PAIRBP1 | 1 | 1p31 |
| SPATA7 | [55812](http://www.ncbi.nlm.nih.gov/gene/55812) | SPATA7 | spermatogenesis associated 7 | HEL-S-296\|HSD-3.1\|HSD3\|LCA3 | 14 | 14q31.3 |
| TK1 | [7083](http://www.ncbi.nlm.nih.gov/gene/7083) | TK1 | thymidine kinase 1, soluble | TK2 | 17 | 17q23.2-q25.3 |
| MARCKSL1 | [65108](http://www.ncbi.nlm.nih.gov/gene/65108) | MARCKSL1 | MARCKS-like 1 | F52\|MACMARCKS\|MLP\|MLP1\|MRP | 1 | 1p35.1 |
| SPC25 | [57405](http://www.ncbi.nlm.nih.gov/gene/57405) | SPC25 | SPC25, NDC80 kinetochore complex component | SPBC25\|hSpc25 | 2 | 2q31.1 |
| CYP2C8 | [1558](http://www.ncbi.nlm.nih.gov/gene/1558) | CYP2C8 | cytochrome P450, family 2, subfamily C, polypeptide 8 | CPC8\|CYPIIC8\|MP-12/MP-20 | 10 | 10q23.33 |
| STUB1 | [10273](http://www.ncbi.nlm.nih.gov/gene/10273) | STUB1 | STIP1 homology and U-box containing protein 1, E3 ubiquitin protein ligase | CHIP\|HSPABP2\|NY-CO-7\|SCAR16\|SDCCAG7\|UBOX1 | 16 | 16p13.3 |
| PRPSAP2 | [5636](http://www.ncbi.nlm.nih.gov/gene/5636) | PRPSAP2 | phosphoribosyl pyrophosphate synthetase-associated protein 2 | PAP41 | 17 | 17p11.2-p12 |
| RNF126 | [55658](http://www.ncbi.nlm.nih.gov/gene/55658) | RNF126 | ring finger protein 126 | - | 19 | 19p13.3 |
| RAB8B | [51762](http://www.ncbi.nlm.nih.gov/gene/51762) | RAB8B | RAB8B, member RAS oncogene family | - | 15 | 15q22.2 |
| MATK | [4145](http://www.ncbi.nlm.nih.gov/gene/4145) | MATK | megakaryocyte-associated tyrosine kinase | CHK\|CTK\|HHYLTK\|HYL\|HYLTK\|Lsk | 19 | 19p13.3 |
| C9 | [735](http://www.ncbi.nlm.nih.gov/gene/735) | C9 | complement component 9 | ARMD15\|C9D | 5 | 5p14-p12 |
| GTF2I | [2969](http://www.ncbi.nlm.nih.gov/gene/2969) | GTF2I | general transcription factor IIi | BAP135\|BTKAP1\|DIWS\|GTFII-I\|IB291\|SPIN\|TFII-I\|WBS\|WBSCR6 | 7 | 7q11.23 |
| BCL7C | [9274](http://www.ncbi.nlm.nih.gov/gene/9274) | BCL7C | B-cell CLL/lymphoma 7C | - | 16 | 16p11 |
| PYCR1 | [5831](http://www.ncbi.nlm.nih.gov/gene/5831) | PYCR1 | pyrroline-5-carboxylate reductase 1 | ARCL2B\|ARCL3B\|P5C\|P5CR\|PIG45\|PP222\|PRO3\|PYCR | 17 | 17q25.3 |
| CDH26 | [60437](http://www.ncbi.nlm.nih.gov/gene/60437) | CDH26 | cadherin 26 | VR20 | 20 | 20q13.33 |
| NDUFA8 | [4702](http://www.ncbi.nlm.nih.gov/gene/4702) | NDUFA8 | NADH dehydrogenase (ubiquinone) 1 alpha subcomplex, 8, 19kDa | CI-19KD\|CI-PGIV\|PGIV | 9 | 9q33.2 |
| DAPP1 | [27071](http://www.ncbi.nlm.nih.gov/gene/27071) | DAPP1 | dual adaptor of phosphotyrosine and 3-phosphoinositides | BAM32 | 4 | 4q25-q27 |
| TOM1 | [10043](http://www.ncbi.nlm.nih.gov/gene/10043) | TOM1 | target of myb1 (chicken) | - | 22 | 22q13.1 |
| FBXL3 | [26224](http://www.ncbi.nlm.nih.gov/gene/26224) | FBXL3 | F-box and leucine-rich repeat protein 3 | FBL3\|FBL3A\|FBXL3A | 13 | 13q22 |
| PCGF3 | [10336](http://www.ncbi.nlm.nih.gov/gene/10336) | PCGF3 | polycomb group ring finger 3 | DONG1\|RNF3\|RNF3A | 4 | 4p16.3 |
| PLCB2 | [5330](http://www.ncbi.nlm.nih.gov/gene/5330) | PLCB2 | phospholipase C, beta 2 | PLC-beta-2 | 15 | 15q15 |
| CHRAC1 | [54108](http://www.ncbi.nlm.nih.gov/gene/54108) | CHRAC1 | chromatin accessibility complex 1 | CHARC1\|CHARC15\|CHRAC-1\|CHRAC-15\|CHRAC15\|YCL1 | 8 | 8q24.3 |
| PAK6 | [56924](http://www.ncbi.nlm.nih.gov/gene/56924) | PAK6 | p21 protein (Cdc42/Rac)-activated kinase 6 | PAK5 | 15 | 15q14 |
| ANXA6 | [309](http://www.ncbi.nlm.nih.gov/gene/309) | ANXA6 | annexin A6 | ANX6\|CBP68 | 5 | 5q33.1 |
| RPS13 | [6207](http://www.ncbi.nlm.nih.gov/gene/6207) | RPS13 | ribosomal protein S13 | S13 | 11 | 11p15 |
| USP14 | [9097](http://www.ncbi.nlm.nih.gov/gene/9097) | USP14 | ubiquitin specific peptidase 14 (tRNA-guanine transglycosylase) | TGT | 18 | 18p11.32 |
| CLP1 | [10978](http://www.ncbi.nlm.nih.gov/gene/10978) | CLP1 | cleavage and polyadenylation factor I subunit 1 | HEAB\|PCH10\|hClp1 | 11 | 11q12 |
| TEAD3 | [7005](http://www.ncbi.nlm.nih.gov/gene/7005) | TEAD3 | TEA domain family member 3 | DTEF-1\|ETFR-1\|TEAD-3\|TEAD5\|TEF-5\|TEF5 | 6 | 6p21.2 |
| COASY | [80347](http://www.ncbi.nlm.nih.gov/gene/80347) | COASY | CoA synthase | DPCK\|NBIA6\|NBP\|PPAT\|UKR1\|pOV-2 | 17 | 17q12-q21 |
| TMEM106A | [113277](http://www.ncbi.nlm.nih.gov/gene/113277) | TMEM106A | transmembrane protein 106A | - | 17 | 17q21.31 |
| RECQL5 | [9400](http://www.ncbi.nlm.nih.gov/gene/9400) | RECQL5 | RecQ protein-like 5 | RECQ5 | 17 | 17q25 |
| ANXA11 | [311](http://www.ncbi.nlm.nih.gov/gene/311) | ANXA11 | annexin A11 | ANX11\|CAP50 | 10 | 10q23 |
| PDS5B | [23047](http://www.ncbi.nlm.nih.gov/gene/23047) | PDS5B | PDS5, regulator of cohesion maintenance, homolog B (S. cerevisiae) | APRIN\|AS3\|CG008 | 13 | 13q12.3 |
| RNF11 | [26994](http://www.ncbi.nlm.nih.gov/gene/26994) | RNF11 | ring finger protein 11 | SID1669 | 1 | 1p32 |
| ABLIM3 | [22885](http://www.ncbi.nlm.nih.gov/gene/22885) | ABLIM3 | actin binding LIM protein family, member 3 | - | 5 | 5q32 |
| MAPK1 | [5594](http://www.ncbi.nlm.nih.gov/gene/5594) | MAPK1 | mitogen-activated protein kinase 1 | ERK\|ERK-2\|ERK2\|ERT1\|MAPK2\|P42MAPK\|PRKM1\|PRKM2\|p38\|p40\|p41\|p41mapk\|p42-MAPK | 22 | 22q11.21 |
| TXNRD1 | [7296](http://www.ncbi.nlm.nih.gov/gene/7296) | TXNRD1 | thioredoxin reductase 1 | GRIM-12\|TR\|TR1\|TRXR1\|TXNR | 12 | 12q23-q24.1 |
| POLE3 | [54107](http://www.ncbi.nlm.nih.gov/gene/54107) | POLE3 | polymerase (DNA directed), epsilon 3, accessory subunit | CHARAC17\|CHRAC17\|YBL1\|p17 | 9 | 9q33 |
| CCL5 | [6352](http://www.ncbi.nlm.nih.gov/gene/6352) | CCL5 | chemokine (C-C motif) ligand 5 | D17S136E\|RANTES\|SCYA5\|SIS-delta\|SISd\|TCP228\|eoCP | 17 | 17q12 |
| SRI | [6717](http://www.ncbi.nlm.nih.gov/gene/6717) | SRI | sorcin | CP-22\|CP22\|SCN\|V19 | 7 | 7q21.1 |
| MLX | [6945](http://www.ncbi.nlm.nih.gov/gene/6945) | MLX | MLX, MAX dimerization protein | MAD7\|MXD7\|TCFL4\|bHLHd13 | 17 | 17q21.1 |
| TARDBP | [23435](http://www.ncbi.nlm.nih.gov/gene/23435) | TARDBP | TAR DNA binding protein | ALS10\|TDP-43 | 1 | 1p36.22 |
| HCRTR1 | [3061](http://www.ncbi.nlm.nih.gov/gene/3061) | HCRTR1 | hypocretin (orexin) receptor 1 | OX1R | 1 | 1p33 |
| RASGEF1A | [221002](http://www.ncbi.nlm.nih.gov/gene/221002) | RASGEF1A | RasGEF domain family, member 1A | CG4853 | 10 | 10q11.21 |
| MOXD1 | [26002](http://www.ncbi.nlm.nih.gov/gene/26002) | MOXD1 | monooxygenase, DBH-like 1 | MOX\|PRO5780\|dJ248E1.1 | 6 | 6q23.2 |
| CKM | [1158](http://www.ncbi.nlm.nih.gov/gene/1158) | CKM | creatine kinase, muscle | CKMM\|M-CK | 19 | 19q13.32 |
| F8 | [2157](http://www.ncbi.nlm.nih.gov/gene/2157) | F8 | coagulation factor VIII, procoagulant component | AHF\|DXS1253E\|F8B\|F8C\|FVIII\|HEMA | X | Xq28 |
| EDNRA | [1909](http://www.ncbi.nlm.nih.gov/gene/1909) | EDNRA | endothelin receptor type A | ET-A\|ETA\|ETA-R\|ETAR\|ETRA\|hET-AR | 4 | 4q31.22 |
| SLA | [6503](http://www.ncbi.nlm.nih.gov/gene/6503) | SLA | Src-like-adaptor | SLA1\|SLAP | 8 | 8q24 |
| TRIML1 | [339976](http://www.ncbi.nlm.nih.gov/gene/339976) | TRIML1 | tripartite motif family-like 1 | RNF209 | 4 | 4q35.2 |
| CETN3 | [1070](http://www.ncbi.nlm.nih.gov/gene/1070) | CETN3 | centrin, EF-hand protein, 3 | CDC31\|CEN3 | 5 | 5q14.3 |
| PSMD10 | [5716](http://www.ncbi.nlm.nih.gov/gene/5716) | PSMD10 | proteasome (prosome, macropain) 26S subunit, non-ATPase, 10 | dJ889N15.2\|p28\|p28(GANK) | X | Xq22.3 |
| TMEM185B | [79134](http://www.ncbi.nlm.nih.gov/gene/79134) | TMEM185B | transmembrane protein 185B | FAM11B | 2 | 2q14.2 |
| LRFN1 | [57622](http://www.ncbi.nlm.nih.gov/gene/57622) | LRFN1 | leucine rich repeat and fibronectin type III domain containing 1 | SALM2 | 19 | 19q13.2 |
| LEPROTL1 | [23484](http://www.ncbi.nlm.nih.gov/gene/23484) | LEPROTL1 | leptin receptor overlapping transcript-like 1 | HSPC112\|Vps55\|my047 | 8 | 8p21 |
| ALDH1A1 | [216](http://www.ncbi.nlm.nih.gov/gene/216) | ALDH1A1 | aldehyde dehydrogenase 1 family, member A1 | ALDC\|ALDH-E1\|ALDH1\|ALDH11\|HEL-9\|HEL-S-53e\|HEL12\|PUMB1\|RALDH1 | 9 | 9q21.13 |
| EPB41L3 | [23136](http://www.ncbi.nlm.nih.gov/gene/23136) | EPB41L3 | erythrocyte membrane protein band 4.1-like 3 | 4.1B\|DAL-1\|DAL1 | 18 | 18p11.32 |
| SAR1B | [51128](http://www.ncbi.nlm.nih.gov/gene/51128) | SAR1B | secretion associated, Ras related GTPase 1B | ANDD\|CMRD\|GTBPB\|SARA2 | 5 | 5q31.1 |
| SELENBP1 | [8991](http://www.ncbi.nlm.nih.gov/gene/8991) | SELENBP1 | selenium binding protein 1 | HEL-S-134P\|LPSB\|SBP56\|SP56\|hSBP | 1 | 1q21.3 |
| MMP7 | [4316](http://www.ncbi.nlm.nih.gov/gene/4316) | MMP7 | matrix metallopeptidase 7 (matrilysin, uterine) | MMP-7\|MPSL1\|PUMP-1 | 11 | 11q21-q22 |
| ABCF3 | [55324](http://www.ncbi.nlm.nih.gov/gene/55324) | ABCF3 | ATP-binding cassette, sub-family F (GCN20), member 3 | EST201864 | 3 | 3q27.1 |
| CCDC102B | [79839](http://www.ncbi.nlm.nih.gov/gene/79839) | CCDC102B | coiled-coil domain containing 102B | ACY1L\|AN\|C18orf14\|HsT1731 | 18 | 18q22.1 |
| RAB11A | [8766](http://www.ncbi.nlm.nih.gov/gene/8766) | RAB11A | RAB11A, member RAS oncogene family | YL8 | 15 | 15q22.31 |
| RUVBL1 | [8607](http://www.ncbi.nlm.nih.gov/gene/8607) | RUVBL1 | RuvB-like AAA ATPase 1 | ECP54\|INO80H\|NMP238\|PONTIN\|Pontin52\|RVB1\|TIH1\|TIP49\|TIP49A | 3 | 3q21 |
| TPD52L2 | [7165](http://www.ncbi.nlm.nih.gov/gene/7165) | TPD52L2 | tumor protein D52-like 2 | D54 | 20 | 20q13.2-q13.3 |
| SNX1 | [6642](http://www.ncbi.nlm.nih.gov/gene/6642) | SNX1 | sorting nexin 1 | HsT17379\|VPS5 | 15 | 15q22.31 |
| PAK4 | [10298](http://www.ncbi.nlm.nih.gov/gene/10298) | PAK4 | p21 protein (Cdc42/Rac)-activated kinase 4 | - | 19 | 19q13.2 |
| STK25 | [10494](http://www.ncbi.nlm.nih.gov/gene/10494) | STK25 | serine/threonine kinase 25 | SOK1\|YSK1 | 2 | 2q37.3 |
| DUPD1 | [338599](http://www.ncbi.nlm.nih.gov/gene/338599) | DUPD1 | dual specificity phosphatase and pro isomerase domain containing 1 | DUSP27\|FMDSP | 10 | 10q22.2 |
| FGFR2 | [2263](http://www.ncbi.nlm.nih.gov/gene/2263) | FGFR2 | fibroblast growth factor receptor 2 | BBDS\|BEK\|BFR-1\|CD332\|CEK3\|CFD1\|ECT1\|JWS\|K-SAM\|KGFR\|TK14\|TK25 | 10 | 10q26 |
| SLC25A10 | [1468](http://www.ncbi.nlm.nih.gov/gene/1468) | SLC25A10 | solute carrier family 25 (mitochondrial carrier; dicarboxylate transporter), member 10 | DIC | 17 | 17q25.3 |
| S100A7A | [338324](http://www.ncbi.nlm.nih.gov/gene/338324) | S100A7A | S100 calcium binding protein A7A | NICE-2\|S100A15\|S100A7L1\|S100A7f | 1 | 1q21.3 |
| FGA | [2243](http://www.ncbi.nlm.nih.gov/gene/2243) | FGA | fibrinogen alpha chain | Fib2 | 4 | 4q28 |
| LEMD1 | [93273](http://www.ncbi.nlm.nih.gov/gene/93273) | LEMD1 | LEM domain containing 1 | CT50\|LEMP-1 | 1 | 1q32.1 |
| DDC | [1644](http://www.ncbi.nlm.nih.gov/gene/1644) | DDC | dopa decarboxylase (aromatic L-amino acid decarboxylase) | AADC | 7 | 7p12.2 |
| FMN1 | [342184](http://www.ncbi.nlm.nih.gov/gene/342184) | FMN1 | formin 1 | FMN\|LD | 15 | 15q13.3 |
| FAM104B | [90736](http://www.ncbi.nlm.nih.gov/gene/90736) | FAM104B | family with sequence similarity 104, member B | CXorf44 | X | Xp11.21 |
| PECAM1 | [5175](http://www.ncbi.nlm.nih.gov/gene/5175) | PECAM1 | platelet/endothelial cell adhesion molecule 1 | CD31\|CD31/EndoCAM\|GPIIA'\|PECA1\|PECAM-1\|endoCAM | 17 | 17q23.3 |
| TAF7 | [6879](http://www.ncbi.nlm.nih.gov/gene/6879) | TAF7 | TAF7 RNA polymerase II, TATA box binding protein (TBP)-associated factor, 55kDa | TAF2F\|TAFII55 | 5 | 5q31 |
| PAF1 | [54623](http://www.ncbi.nlm.nih.gov/gene/54623) | PAF1 | Paf1, RNA polymerase II associated factor, homolog (S. cerevisiae) | F23149_1\|PD2 | 19 | 19q13.1 |
| HSPBAP1 | [79663](http://www.ncbi.nlm.nih.gov/gene/79663) | HSPBAP1 | HSPB (heat shock 27kDa) associated protein 1 | PASS1 | 3 | 3q21.1 |
| SNX9 | [51429](http://www.ncbi.nlm.nih.gov/gene/51429) | SNX9 | sorting nexin 9 | SDP1\|SH3PX1\|SH3PXD3A\|WISP | 6 | 6q25.1-q26 |
| FBP1 | [2203](http://www.ncbi.nlm.nih.gov/gene/2203) | FBP1 | fructose-1,6-bisphosphatase 1 | FBP | 9 | 9q22.3 |
| ZNF655 | [79027](http://www.ncbi.nlm.nih.gov/gene/79027) | ZNF655 | zinc finger protein 655 | VIK\|VIK-1 | 7 | 7q22.1 |
| LST1 | [7940](http://www.ncbi.nlm.nih.gov/gene/7940) | LST1 | leukocyte specific transcript 1 | B144\|D6S49E\|LST-1 | 6 | 6p21.3 |
| SET | [6418](http://www.ncbi.nlm.nih.gov/gene/6418) | SET | SET nuclear proto-oncogene | 2PP2A\|I2PP2A\|IGAAD\|IPP2A2\|PHAPII\|TAF-I\|TAF-IBETA | 9 | 9q34 |
| PPP3R1 | [5534](http://www.ncbi.nlm.nih.gov/gene/5534) | PPP3R1 | protein phosphatase 3, regulatory subunit B, alpha | CALNB1\|CNB\|CNB1 | 2 | 2p15 |
| PRKAR2B | [5577](http://www.ncbi.nlm.nih.gov/gene/5577) | PRKAR2B | protein kinase, cAMP-dependent, regulatory, type II, beta | PRKAR2\|RII-BETA | 7 | 7q22 |
| RPS6 | [6194](http://www.ncbi.nlm.nih.gov/gene/6194) | RPS6 | ribosomal protein S6 | S6 | 9 | 9p21 |
| ATP6V1C2 | [245973](http://www.ncbi.nlm.nih.gov/gene/245973) | ATP6V1C2 | ATPase, H+ transporting, lysosomal 42kDa, V1 subunit C2 | ATP6C2\|VMA5 | 2 | - |
| MYLK | [4638](http://www.ncbi.nlm.nih.gov/gene/4638) | MYLK | myosin light chain kinase | AAT7\|KRP\|MLCK\|MLCK1\|MLCK108\|MLCK210\|MSTP083\|MYLK1\|smMLCK | 3 | 3q21 |
| VCP | [7415](http://www.ncbi.nlm.nih.gov/gene/7415) | VCP | valosin containing protein | ALS14\|HEL-220\|HEL-S-70\|IBMPFD\|IBMPFD1\|TERA\|p97 | 9 | 9p13.3 |
| RAC1 | [5879](http://www.ncbi.nlm.nih.gov/gene/5879) | RAC1 | ras-related C3 botulinum toxin substrate 1 (rho family, small GTP binding protein Rac1) | Rac-1\|TC-25\|p21-Rac1 | 7 | 7p22 |
| COG3 | [83548](http://www.ncbi.nlm.nih.gov/gene/83548) | COG3 | component of oligomeric golgi complex 3 | SEC34 | 13 | 13q14.13 |
| MBIP | [51562](http://www.ncbi.nlm.nih.gov/gene/51562) | MBIP | MAP3K12 binding inhibitory protein 1 | - | 14 | 14q13.3 |
| HSPBP1 | [23640](http://www.ncbi.nlm.nih.gov/gene/23640) | HSPBP1 | HSPA (heat shock 70kDa) binding protein, cytoplasmic cochaperone 1 | FES1 | 19 | 19q13.42 |
| PLEK | [5341](http://www.ncbi.nlm.nih.gov/gene/5341) | PLEK | pleckstrin | P47 | 2 | 2p13.3 |
| AKT3 | [10000](http://www.ncbi.nlm.nih.gov/gene/10000) | AKT3 | v-akt murine thymoma viral oncogene homolog 3 | MPPH\|PKB-GAMMA\|PKBG\|PRKBG\|RAC-PK-gamma\|RAC-gamma\|STK-2 | 1 | 1q44 |
| SRP54 | [6729](http://www.ncbi.nlm.nih.gov/gene/6729) | SRP54 | signal recognition particle 54kDa | - | 14 | 14q13.2 |
| NUDT18 | [79873](http://www.ncbi.nlm.nih.gov/gene/79873) | NUDT18 | nudix (nucleoside diphosphate linked moiety X)-type motif 18 | MTH3 | 8 | 8p21.3 |
| GKAP1 | [80318](http://www.ncbi.nlm.nih.gov/gene/80318) | GKAP1 | G kinase anchoring protein 1 | GKAP42 | 9 | 9q21.32 |
| CHORDC1 | [26973](http://www.ncbi.nlm.nih.gov/gene/26973) | CHORDC1 | cysteine and histidine-rich domain (CHORD) containing 1 | CHP1 | 11 | 11q14.3 |
| PHKG2 | [5261](http://www.ncbi.nlm.nih.gov/gene/5261) | PHKG2 | phosphorylase kinase, gamma 2 (testis) | GSD9C | 16 | 16p11.2 |
| HIATL1 | [84641](http://www.ncbi.nlm.nih.gov/gene/84641) | HIATL1 | hippocampus abundant transcript-like 1 | - | 9 | 9q22.32 |
| GFM2 | [84340](http://www.ncbi.nlm.nih.gov/gene/84340) | GFM2 | G elongation factor, mitochondrial 2 | EF-G2mt\|EFG2\|MRRF2\|MST027\|RRF2\|RRF2mt\|hEFG2\|mEF-G 2 | 5 | 5q13 |
| C1orf87 | [127795](http://www.ncbi.nlm.nih.gov/gene/127795) | C1orf87 | chromosome 1 open reading frame 87 | CREF | 1 | 1p32.1 |
| Lhx1 | [3975](http://www.ncbi.nlm.nih.gov/gene/3975) | LHX1 | LIM homeobox 1 | LIM-1\|LIM1 | 17 | 17q12 |
| ZRANB2 | [9406](http://www.ncbi.nlm.nih.gov/gene/9406) | ZRANB2 | zinc finger, RAN-binding domain containing 2 | ZIS\|ZIS1\|ZIS2\|ZNF265 | 1 | 1p31 |
| GBE1 | [2632](http://www.ncbi.nlm.nih.gov/gene/2632) | GBE1 | glucan (1,4-alpha-), branching enzyme 1 | APBD\|GBE\|GSD4 | 3 | 3p12.3 |
| EYA1 | [2138](http://www.ncbi.nlm.nih.gov/gene/2138) | EYA1 | EYA transcriptional coactivator and phosphatase 1 | BOP\|BOR\|BOS1\|OFC1 | 8 | 8q13.3 |
| FABP5 | [2171](http://www.ncbi.nlm.nih.gov/gene/2171) | FABP5 | fatty acid binding protein 5 (psoriasis-associated) | E-FABP\|EFABP\|KFABP\|PA-FABP\|PAFABP | 8 | 8q21.13 |
| SSPN | [8082](http://www.ncbi.nlm.nih.gov/gene/8082) | SSPN | sarcospan | DAGA5\|KRAG\|NSPN\|SPN1\|SPN2 | 12 | 12p11.2 |
| ZMYM3 | [9203](http://www.ncbi.nlm.nih.gov/gene/9203) | ZMYM3 | zinc finger, MYM-type 3 | DXS6673E\|MYM\|XFIM\|ZNF198L2\|ZNF261 | X | Xq13.1 |
| NPM1 | [4869](http://www.ncbi.nlm.nih.gov/gene/4869) | NPM1 | nucleophosmin (nucleolar phosphoprotein B23, numatrin) | B23\|NPM | 5 | 5q35.1 |
| CORO2B | [10391](http://www.ncbi.nlm.nih.gov/gene/10391) | CORO2B | coronin, actin binding protein, 2B | CLIPINC | 15 | 15q23 |
| NRBF2 | [29982](http://www.ncbi.nlm.nih.gov/gene/29982) | NRBF2 | nuclear receptor binding factor 2 | COPR1\|COPR2\|NRBF-2 | 10 | 10q21.3 |
| KRCC1 | [51315](http://www.ncbi.nlm.nih.gov/gene/51315) | KRCC1 | lysine-rich coiled-coil 1 | CHBP2 | 2 | 2p11.2 |
| CIAO1 | [9391](http://www.ncbi.nlm.nih.gov/gene/9391) | CIAO1 | cytosolic iron-sulfur assembly component 1 | CIA1\|WDR39 | 2 | 2q11.2 |
| PGM2 | [55276](http://www.ncbi.nlm.nih.gov/gene/55276) | PGM2 | phosphoglucomutase 2 | - | 4 | 4p14 |
| ARHGAP29 | [9411](http://www.ncbi.nlm.nih.gov/gene/9411) | ARHGAP29 | Rho GTPase activating protein 29 | PARG1 | 1 | 1p22.1 |
| GOLT1B | [51026](http://www.ncbi.nlm.nih.gov/gene/51026) | GOLT1B | golgi transport 1B | GCT2\|GOT1\|GOT1B\|YMR292W | 12 | 12p12.1 |
| KLHL14 | [57565](http://www.ncbi.nlm.nih.gov/gene/57565) | KLHL14 | kelch-like family member 14 | - | 18 | 18q12.1 |
| CYGB | [114757](http://www.ncbi.nlm.nih.gov/gene/114757) | CYGB | cytoglobin | HGB\|STAP | 17 | 17q25 |
| RTN4 | [57142](http://www.ncbi.nlm.nih.gov/gene/57142) | RTN4 | reticulon 4 | ASY\|NI220/250\|NOGO\|NOGO-A\|NOGOC\|NSP\|NSP-CL\|Nbla00271\|Nbla10545\|Nogo-B\|Nogo-C\|RTN-X\|RTN4-A\|RTN4-B1\|RTN4-B2\|RTN4-C | 2 | 2p16.3 |
| OBFC1 | [79991](http://www.ncbi.nlm.nih.gov/gene/79991) | OBFC1 | oligonucleotide/oligosaccharide-binding fold containing 1 | AAF-44\|AAF44\|RPA-32\|STN1\|bA541N10.2 | 10 | 10q24.33 |
| PDXK | [8566](http://www.ncbi.nlm.nih.gov/gene/8566) | PDXK | pyridoxal (pyridoxine, vitamin B6) kinase | C21orf124\|C21orf97\|HEL-S-1a\|PKH\|PNK | 21 | 21q22.3 |
| RNPEP | [6051](http://www.ncbi.nlm.nih.gov/gene/6051) | RNPEP | arginyl aminopeptidase (aminopeptidase B) | - | 1 | 1q32 |
| FAM9C | [171484](http://www.ncbi.nlm.nih.gov/gene/171484) | FAM9C | family with sequence similarity 9, member C | TEX39C | X | Xp22.2 |
| PLEKHG2 | [64857](http://www.ncbi.nlm.nih.gov/gene/64857) | PLEKHG2 | pleckstrin homology domain containing, family G (with RhoGef domain) member 2 | ARHGEF42\|CLG | 19 | 19q13.2 |
| ANXA13 | [312](http://www.ncbi.nlm.nih.gov/gene/312) | ANXA13 | annexin A13 | ANX13\|ISA | 8 | 8q24.13 |
| NFKBIA | [4792](http://www.ncbi.nlm.nih.gov/gene/4792) | NFKBIA | nuclear factor of kappa light polypeptide gene enhancer in B-cells inhibitor, alpha | IKBA\|MAD-3\|NFKBI | 14 | 14q13 |
| DGUOK | [1716](http://www.ncbi.nlm.nih.gov/gene/1716) | DGUOK | deoxyguanosine kinase | MTDPS3\|dGK | 2 | 2p13 |
| ALDH9A1 | [223](http://www.ncbi.nlm.nih.gov/gene/223) | ALDH9A1 | aldehyde dehydrogenase 9 family, member A1 | ALDH4\|ALDH7\|ALDH9\|E3\|TMABADH | 1 | 1q23.1 |
| ACVR2B | [93](http://www.ncbi.nlm.nih.gov/gene/93) | ACVR2B | activin A receptor, type IIB | ACTRIIB\|ActR-IIB\|HTX4 | 3 | 3p22 |
| C8orf37 | [157657](http://www.ncbi.nlm.nih.gov/gene/157657) | C8orf37 | chromosome 8 open reading frame 37 | CORD16\|RP64\|smalltalk | 8 | 8q22.1 |
| UBE2O | [63893](http://www.ncbi.nlm.nih.gov/gene/63893) | UBE2O | ubiquitin-conjugating enzyme E2O | E2-230K | 17 | 17q25.1 |
| PEX19 | [5824](http://www.ncbi.nlm.nih.gov/gene/5824) | PEX19 | peroxisomal biogenesis factor 19 | D1S2223E\|HK33\|PBD12A\|PMP1\|PMPI\|PXF\|PXMP1 | 1 | 1q23.2 |
|  |  |  |  |  |  |  |
| Redundant entries |  |  |  |  |  |  |
|  | Entry "HSPA2" ignored. Both "HSPA2" and "HSPA2" mappes to "HSPA2" | | |  |  |  |
|  | Entry "HLA-DRB3" ignored. Both "HLA-DRB3" and "HLA-DRB3" mappes to "HLA-DRB3" | | |  |  |  |
|  | Entry "ARL2BP" ignored. Both "ARL2BP" and "ARL2BP" mappes to "ARL2BP" | | |  |  |  |
|  | Entry "CAMK2N1" ignored. Both "CAMK2N1" and "CAMK2N1" mappes to "CAMK2N1" | | |  |  |  |
|  | Entry "AKT3" ignored. Both "AKT3" and "AKT3" mappes to "AKT3" | | |  |  |  |
|  |  |  |  |  |  |  |
| Unmapped Entries |  |  |  |  |  |  |
|  | IGHG4 |  |  |  |  |  |
|  | ND |  |  |  |  |  |
|  | WDR42A |  |  |  |  |  |
|  | RY1 |  |  |  |  |  |
|  | HCG3 |  |  |  |  |  |
|  | C20orf112 |  |  |  |  |  |
|  | HDAC7A |  |  |  |  |  |
|  | ND |  |  |  |  |  |
|  | C17orf57 |  |  |  |  |  |
|  | CHP |  |  |  |  |  |
|  | LOC389833 |  |  |  |  |  |
|  | ND |  |  |  |  |  |
|  | N.D. |  |  |  |  |  |
|  | PRIM2A |  |  |  |  |  |
|  | KARCA1 |  |  |  |  |  |
|  | NA |  |  |  |  |  |
|  | SURB7 |  |  |  |  |  |
|  | P15RS |  |  |  |  |  |
|  | C20orf42 |  |  |  |  |  |
|  | ND |  |  |  |  |  |
|  | ND |  |  |  |  |  |
|  | ND |  |  |  |  |  |
|  | THRAP6 |  |  |  |  |  |
|  | HRASLS3 |  |  |  |  |  |
|  | ND |  |  |  |  |  |
|  | ND |  |  |  |  |  |
|  | C12orf11 |  |  |  |  |  |
|  | IGKC |  |  |  |  |  |
|  | MRS2L |  |  |  |  |  |
|  | NA |  |  |  |  |  |
|  | ND |  |  |  |  |  |
|  | NA |  |  |  |  |  |
|  | APITD1 |  |  |  |  |  |
|  | ND |  |  |  |  |  |
|  | PBEF1 |  |  |  |  |  |
|  | NA |  |  |  |  |  |
|  | C8orf43 |  |  |  |  |  |
|  | ND |  |  |  |  |  |
|  | FAM40B |  |  |  |  |  |
|  | XTP3TPA |  |  |  |  |  |
|  | ND |  |  |  |  |  |
|  | ND |  |  |  |  |  |
|  | LOC387758 |  |  |  |  |  |
|  | NA |  |  |  |  |  |
|  | TMEM166 |  |  |  |  |  |
|  | NA |  |  |  |  |  |
|  | C11orf67 |  |  |  |  |  |
|  | LOC196541 |  |  |  |  |  |
|  | N.D. |  |  |  |  |  |
|  | ND |  |  |  |  |  |
|  | UBADC1 |  |  |  |  |  |
|  | NA |  |  |  |  |  |
|  | IGL@ |  |  |  |  |  |
|  | ND |  |  |  |  |  |
|  | ND |  |  |  |  |  |
|  | C14orf122 |  |  |  |  |  |
|  | LOC285382 |  |  |  |  |  |
|  | ND |  |  |  |  |  |
|  | ND |  |  |  |  |  |
|  | ND |  |  |  |  |  |
|  | FAM119B |  |  |  |  |  |
|  | ND |  |  |  |  |  |
|  | C1orf76 |  |  |  |  |  |
|  | C13orf3 |  |  |  |  |  |
|  | C9orf123 |  |  |  |  |  |
|  | LOC652968 |  |  |  |  |  |
|  | PCID1 |  |  |  |  |  |
|  | C14orf126 |  |  |  |  |  |
|  | IGHG1 |  |  |  |  |  |
|  | C20orf28 |  |  |  |  |  |
|  | ND |  |  |  |  |  |
|  | VIL2 |  |  |  |  |  |
|  | M6PRBP1 |  |  |  |  |  |
|  | ND |  |  |  |  |  |
|  | ND |  |  |  |  |  |
|  | ZNF643 |  |  |  |  |  |
|  | C21orf33 |  |  |  |  |  |
|  | MDP-1 |  |  |  |  |  |
|  | C14orf149 |  |  |  |  |  |
|  | ND |  |  |  |  |  |
|  | LOC158381 |  |  |  |  |  |
|  | NY-REN-7 |  |  |  |  |  |
|  | ND |  |  |  |  |  |
|  | ND |  |  |  |  |  |
|  | ND |  |  |  |  |  |
|  | C13orf15 |  |  |  |  |  |
|  | KBTBD5 |  |  |  |  |  |
|  | ND |  |  |  |  |  |
|  | ND |  |  |  |  |  |
|  | MTERFD2 |  |  |  |  |  |
|  | IGHD |  |  |  |  |  |
|  | KIAA1576 |  |  |  |  |  |
|  | CXorf9 |  |  |  |  |  |
|  | ND |  |  |  |  |  |
|  | EIF3S3 |  |  |  |  |  |
|  | ND |  |  |  |  |  |
|  | FLJ22222 |  |  |  |  |  |
|  | IGHG1 |  |  |  |  |  |
|  | JUB |  |  |  |  |  |
|  | MGC2408 |  |  |  |  |  |
|  | FAM13A1 |  |  |  |  |  |
|  | N.D. |  |  |  |  |  |
|  | SFRS15 |  |  |  |  |  |
|  | SPN |  |  |  |  |  |
|  | LOC729447 |  |  |  |  |  |
|  | SAS10 |  |  |  |  |  |
|  | IHPK1 |  |  |  |  |  |
|  | ND |  |  |  |  |  |
|  | SGK |  |  |  |  |  |
|  | C20orf43 |  |  |  |  |  |
|  | IGHG1 |  |  |  |  |  |
|  | C6orf115 |  |  |  |  |  |
|  | RP11-56A21.1 |  |  |  |  |  |
|  | ND |  |  |  |  |  |
|  | TXNL2 |  |  |  |  |  |
|  | LOC554174 |  |  |  |  |  |
|  | PIP5K2C |  |  |  |  |  |
|  | ASAP |  |  |  |  |  |
|  | LOC374395 |  |  |  |  |  |

**Supplementary Table 5.2: Cellular component**

| Analysis:Cellular component |  |  |  |  |  |  |  |  |  |
| --- | --- | --- | --- | --- | --- | --- | --- | --- | --- |
| Name of data set: MG1 vs HC |  |  |  |  |  |  |  |  |  |
| Number of gene in data set: 365 |  |  |  |  |  |  |  |  |  |
| Number of gene mapped to Cellular component : 319 |  |  |  |  |  |  |  |  |  |
|  |  |  |  |  |  |  |  |  |  |
| Cellular component | No. of genes  in the data set | No. of genes in the background data set | Percentage of genes | Fold Enrichment | Uncorrected  p-value  (Hypergeometric test) | Corrected  p-value  (Bonferroni method) | Corrected  p-value  (BH method) | Storey and Tibshirani method  q-value | Genes mapped from  input data set |
| Cytoplasm | 188 | 5632 | 58.93416928 | 1.51041898 | 3.9E-13 | 5.46E-11 | 5.46E-11 | 1.15E-11 | CRYM,STAT6,ZHX3,CCNB1,DRG1,PROSC,DNAJB5,SRPX2,AFP,STK33,FHL2,RAB3B,STAU2,CALCOCO2,TYROBP,RPL10,CSRP1,APOE,COX4I1,HPCAL1,UPP2,AEBP2,HSPA2,RWDD3,CARHSP1,NAPSA,IGLL1,GSTP1,RPS6KA2,CORO1A,FUBP1,FAM63A,CAPRIN2,FBXO31,PPP2R4,ADRBK1,QDPR,IFIT3,RHOA,DLX5,RAP1GDS1,ATG3,NECAP2,CHD4,DOK1,PSMD14,IMPDH2,PANK1,DHX9,GNB2L1,FAM58A,CYB5R1,CDC34,RAB39B,VSNL1,RRAGB,CTNNAL1,SFN,FN3K,SEPT1,NME1,ADD1,CAMK4,DCK,DHFR,FBXO3,HN1,DDX58,ARPC3,CLGN,MTL5,SULT1E1,SH2D1B,NOSIP,EIF4EBP3,PTS,AAK1,ARHGDIG,APEX1,CCT4,ABLIM1,ARIH2,MAPK3,TIRAP,CDCP1,ARF5,YWHAB,DBNL,TCOF1,RPL14,C1QC,RNF32,USP15,PTPN11,ZFYVE19,PRKRA,SMYD3,DIABLO,PDLIM3,CKAP2,CPNE4,MEIS2,MCTS1,RPL22,SRPK2,HSPB8,C11orf49,SERBP1,SPATA7,TK1,CYP2C8,STUB1,PRPSAP2,RAB8B,MATK,GTF2I,BCL7C,PYCR1,DAPP1,TOM1,PLCB2,PAK6,ANXA6,USP14,COASY,RECQL5,ANXA11,RNF11,ABLIM3,MAPK1,TXNRD1,CCL5,SRI,MLX,CKM,SLA,PSMD10,ALDH1A1,SELENBP1,RAB11A,RUVBL1,TPD52L2,SNX1,PAK4,FGFR2,S100A7A,FGA,DDC,FMN1,TAF7,FBP1,ZNF655,LST1,SET,PPP3R1,PRKAR2B,MYLK,VCP,RAC1,COG3,MBIP,PLEK,AKT3,SRP54,PHKG2,GFM2,ZRANB2,EYA1,FABP5,ZMYM3,NPM1,CORO2B,NRBF2,CIAO1,PGM2,ARHGAP29,KLHL14,CYGB,RTN4,PDXK,FAM9C,PLEKHG2,NFKBIA,DGUOK,ALDH9A1,ACVR2B,UBE2O,PEX19 |
| Exosomes | 74 | 2001 | 23.19749216 | 1.689696769 | 4.2E-06 | 0.000589 | 0.000294 | 4.23E-05 | CRYM,RAB3B,NRAS,RPL10,KLK1,CSRP1,BHMT2,APOE,HLA-DRB5,COX4I1,HPCAL1,HSPA2,NAPSA,GSTP1,CORO1A,ATAD2,RPS7,QDPR,RHOA,RAP1GDS1,HPX,SLC5A6,IMPDH2,DHX9,GNB2L1,CYB5R1,CLDN4,EPS8L1,SFN,NME1,CAMK4,ARPC3,CCT4,ARF5,YWHAB,DBNL,RPL14,C1QC,UGDH,NAGK,RPL22,HSPB8,SERBP1,MARCKSL1,RAB8B,C9,TOM1,ANXA6,RPS13,COASY,ANXA11,RNF11,TXNRD1,SRI,ALDH1A1,SELENBP1,MMP7,RAB11A,RUVBL1,FGA,DDC,PECAM1,SNX9,FBP1,ATP6V1C2,VCP,RAC1,GBE1,NPM1,RTN4,PDXK,RNPEP,ANXA13,ALDH9A1 |
| Cytosol | 50 | 1165 | 15.67398119 | 1.975708119 | 4.29E-06 | 0.000601 | 0.0002 | 4.23E-05 | CCNB1,RPL10,UPP2,GALT,RPS6KA2,RPS7,ADRBK1,ATG3,DOK1,IMPDH2,PANK1,CDO1,CTNNAL1,ADD1,RPS15,ANAPC11,SULT1E1,NOSIP,CCT4,MAPK3,YWHAB,DBNL,RPL14,UGDH,PTPN11,DIABLO,RPL22,TOM1,PLCB2,RPS13,USP14,MAPK1,TXNRD1,CKM,PAK4,STK25,SET,PPP3R1,PRKAR2B,RPS6,MYLK,VCP,RAC1,PLEK,AKT3,GBE1,PGM2,PDXK,PLEKHG2,NFKBIA |
| Nucleolus | 46 | 1227 | 14.4200627 | 1.729559916 | 0.000284 | 0.03969 | 0.009923 | 0.002092 | STAT6,CCNB1,NFE2,RPL10,KRR1,CSRP1,HSPA2,SP110,FUBP1,RPS7,DLX5,CHD4,DHX9,GNB2L1,MRTO4,ANP32E,CAMK4,ARPC3,NOSIP,APEX1,CCT4,MAPK3,TCOF1,RPL14,C1QC,USP15,MCM5,PDLIM3,RBBP7,MEIS2,RPL22,SRPK2,SERBP1,GTF2I,RPS13,MAPK1,TARDBP,RUVBL1,TAF7,ZNF655,RPS6,VCP,MBIP,SRP54,ZMYM3,NPM1 |
| Nucleus | 156 | 5766 | 48.90282132 | 1.225864442 | 0.000738 | 0.103278 | 0.020656 | 0.004355 | HOXA5,STAT6,ZHX3,CCNB1,DRG1,PROSC,DNAJB5,OIP5,STK33,FHL2,NFE2,STAU2,CALCOCO2,KRR1,CSRP1,ERAL1,APOE,COX4I1,AEBP2,HSPA2,SP110,RWDD3,CARHSP1,FUBP3,GSTP1,RPS6KA2,CORO1A,FUBP1,ATAD2,FBXO31,STATH,PPP2R4,QDPR,RHOA,DLX5,GADD45A,TIPIN,HPX,NECAP2,CHD4,PSMD14,DHX9,GNB2L1,BCAS2,FAM58A,CDC34,RAB39B,SFN,ANP32E,NHEJ1,NME1,ADD1,ACTL6B,CAMK4,RPS15,DCK,ZNF641,DHFR,FBXO3,HN1,ARPC3,RAB24,MTL5,KLF11,NOSIP,EIF4EBP3,PTS,CCNH,AAK1,APEX1,ZNF761,SERTAD3,CXXC5,ZNF587,ABLIM1,ARIH2,MAPK3,YWHAB,DBNL,TCOF1,C1QC,MORC4,ASF1A,SMYD3,MCM5,PDLIM3,RBBP7,MEIS2,RPL22,SRPK2,HSPB8,C11orf49,SERBP1,SPATA7,SPC25,STUB1,PRPSAP2,MATK,GTF2I,BCL7C,PYCR1,FBXL3,PLCB2,CHRAC1,PAK6,RPS13,TEAD3,RECQL5,ANXA11,PDS5B,RNF11,MAPK1,TXNRD1,POLE3,CCL5,MLX,TARDBP,SLA,PSMD10,EPB41L3,RUVBL1,TPD52L2,PAK4,FGFR2,FGA,LEMD1,FMN1,PECAM1,TAF7,PAF1,ZNF655,SET,PPP3R1,PRKAR2B,RPS6,VCP,MBIP,PLEK,AKT3,SRP54,LHX1,ZRANB2,EYA1,ZMYM3,NPM1,NRBF2,CIAO1,CYGB,RTN4,OBFC1,RNPEP,FAM9C,PLEKHG2,NFKBIA,ALDH9A1,PEX19 |
| Ribosome | 10 | 141 | 3.134796238 | 3.555664854 | 0.001182 | 0.165544 | 0.027591 | 0.005817 | STAU2,RPL10,RPS7,RPS15,APEX1,RPL14,QTRT1,RPL22,RPS13,RPS6 |
| Centrosome | 26 | 629 | 8.150470219 | 1.943901361 | 0.001714 | 0.239947 | 0.034278 | 0.007227 | CCNB1,RPL10,CARHSP1,CORO1A,RPS7,IMPDH2,DHX9,GNB2L1,ANP32E,SEPT1,RPS15,APEX1,CCT4,YWHAB,RPL22,SERBP1,PYCR1,TOM1,ANXA6,RPS13,MAPK1,CETN3,PRKAR2B,RPS6,VCP,NPM1 |
| Actin cytoskeleton | 9 | 130 | 2.821316614 | 3.511115572 | 0.002429 | 0.34 | 0.0425 | 0.008961 | FHL2,NFE2,RHOA,ARPC3,ABLIM1,MYOT,DBNL,PSMD10,CORO2B |
| Phagocytic vesicle | 2 | 4 | 0.626959248 | 27.83201368 | 0.002851 | 0.399162 | 0.044351 | 0.009351 | CORO1A,ANXA11 |

**Supplementary Table 5.3: Molecular function**

| Analysis:Molecular function |  |  |  |  |  |  |  |  |  |
| --- | --- | --- | --- | --- | --- | --- | --- | --- | --- |
| Name of data set: MG1 vs HC |  |  |  |  |  |  |  |  |  |
| Number of gene in data set: 365 |  |  |  |  |  |  |  |  |  |
| Number of gene mapped to Molecular function : 363 |  |  |  |  |  |  |  |  |  |
|  |  |  |  |  |  |  |  |  |  |
| Molecular function | No. of genes  in the data set | No. of genes in the background data set | Percentage of genes | Fold Enrichment | Uncorrected  p-value  (Hypergeometric test) | Corrected  p-value  (Bonferroni method) | Corrected  p-value  (BH method) | Storey and Tibshirani method  q-value | Genes mapped from  input data set |
| Calcium ion binding | 12 | 182 | 3.305785124 | 3.556472447 | 0.000348 | 0.025775 | 0.025775 | 0.01327 | EFCAB2,HPCAL1,CARHSP1,VSNL1,CALN1,ANXA6,ANXA11,SRI,CETN3,S100A7A,PLEK,ANXA13 |
| Structural constituent of ribosome | 10 | 150 | 2.754820937 | 3.658513758 | 0.000981 | 0.072573 | 0.036287 | 0.018681 | MRPS7,RPL10,RPS7,MRPS25,RPS15,MRPL53,RPL14,RPL22,RPS13,RPS6 |
| GTPase activity | 12 | 222 | 3.305785124 | 2.919254539 | 0.001973 | 0.145968 | 0.048656 | 0.02505 | RAB3B,NRAS,ERAL1,RHOA,RAB39B,SEPT1,RAB24,ARF5,RAB8B,SAR1B,RAB11A,RAC1 |

**Supplementary Table 5.4: Biological process**

| Analysis:Biological process |  |  |  |  |  |  |  |  |  |
| --- | --- | --- | --- | --- | --- | --- | --- | --- | --- |
| Name of data set: MG1 vs HC |  |  |  |  |  |  |  |  |  |
| Number of gene in data set: 365 |  |  |  |  |  |  |  |  |  |
| Number of gene mapped to Biological process : 363 |  |  |  |  |  |  |  |  |  |
|  |  |  |  |  |  |  |  |  |  |
| Biological process | No. of genes  in the data set | No. of genes in the background data set | Percentage of genes | Fold Enrichment | Uncorrected  p-value  (Hypergeometric test) | Corrected  p-value  (Bonferroni method) | Corrected  p-value  (BH method) | Storey and Tibshirani method  q-value | Genes mapped from  input data set |
| Protein metabolism | 44 | 1314 | 12.12121212 | 1.692223908 | 0.000695 | 0.023626 | 0.023626 | 0.010499 | DNAJB5,MRPS7,RPL10,KLK1,HSPA2,NAPSA,RPS7,MRPS25,PSMD14,CDC34,FN3K,FBXO3,CLGN,EIF4EBP3,CCT4,MRPL53,ARIH2,GGT6,RPL14,RNF32,USP15,ASF1A,ZFYVE19,PI16,RPL22,HSPB8,RNF126,FBXL3,PCGF3,RPS13,USP14,RNF11,F8,TRIML1,PSMD10,SELENBP1,MMP7,FGA,RPS6,HSPBP1,SRP54,NPM1,RNPEP,UBE2O |

**Supplementary Table 5.5: Biological pathway**

| Analysis:Biological pathway |  |  |  |  |  |  |  |  |  |
| --- | --- | --- | --- | --- | --- | --- | --- | --- | --- |
| Name of data set: MG1 vs HC |  |  |  |  |  |  |  |  |  |
| Number of gene in data set: 365 |  |  |  |  |  |  |  |  |  |
| Number of gene mapped to Biological pathway : 159 |  |  |  |  |  |  |  |  |  |
|  |  |  |  |  |  |  |  |  |  |
| Biological pathway | No. of genes  in the data set | No. of genes in the background data set | Percentage of genes | Fold Enrichment | Uncorrected  p-value  (Hypergeometric test) | Corrected  p-value  (Bonferroni method) | Corrected  p-value  (BH method) | Storey and Tibshirani method  q-value | Genes mapped from  input data set |
| RAF/MAP kinase cascade | 5 | 11 | 3.144654088 | 19.10041708 | 4.99E-06 | 0.003755 | 0.003755 | 0.000442 | NRAS,MAPK3,YWHAB,PTPN11,MAPK1 |
| SHC-mediated signalling | 5 | 13 | 3.144654088 | 16.41965679 | 1.33E-05 | 0.010019 | 0.005009 | 0.000469 | NRAS,MAPK3,YWHAB,PTPN11,MAPK1 |
| FRS2-mediated cascade | 5 | 15 | 3.144654088 | 14.39877595 | 2.98E-05 | 0.022391 | 0.007464 | 0.000469 | NRAS,MAPK3,YWHAB,PTPN11,MAPK1 |
| SOS-mediated signalling | 5 | 15 | 3.144654088 | 14.39877595 | 2.98E-05 | 0.022391 | 0.005598 | 0.000469 | NRAS,MAPK3,YWHAB,PTPN11,MAPK1 |
| GRB2 events in EGFR signaling | 5 | 15 | 3.144654088 | 14.39877595 | 2.98E-05 | 0.022391 | 0.004478 | 0.000469 | NRAS,MAPK3,YWHAB,PTPN11,MAPK1 |
| SHC1 events in EGFR signaling | 5 | 16 | 3.144654088 | 13.5640643 | 4.24E-05 | 0.031875 | 0.005312 | 0.000469 | NRAS,MAPK3,YWHAB,PTPN11,MAPK1 |
| Signalling to p38 via RIT and RIN | 5 | 16 | 3.144654088 | 13.5640643 | 4.24E-05 | 0.031875 | 0.004554 | 0.000469 | NRAS,MAPK3,YWHAB,PTPN11,MAPK1 |
| SHC-related events | 5 | 16 | 3.144654088 | 13.5640643 | 4.24E-05 | 0.031875 | 0.003984 | 0.000469 | NRAS,MAPK3,YWHAB,PTPN11,MAPK1 |
| ARMS-mediated activation | 5 | 18 | 3.144654088 | 12.15481087 | 7.96E-05 | 0.05989 | 0.006654 | 0.00078 | NRAS,MAPK3,YWHAB,PTPN11,MAPK1 |
| Signalling by NGF | 13 | 142 | 8.176100629 | 3.724081319 | 8.82E-05 | 0.066297 | 0.00663 | 0.00078 | NRAS,RPS6KA2,ADRBK1,RHOA,CAMK4,MAPK3,YWHAB,PTPN11,MAPK1,PRKAR2B,RAC1,RTN4,NFKBIA |
| Frs2-mediated activation | 5 | 19 | 3.144654088 | 11.55457329 | 0.000106 | 0.07955 | 0.007232 | 0.000851 | NRAS,MAPK3,YWHAB,PTPN11,MAPK1 |
| Prolonged ERK activation events | 5 | 20 | 3.144654088 | 11.01082867 | 0.000138 | 0.103811 | 0.008651 | 0.001018 | NRAS,MAPK3,YWHAB,PTPN11,MAPK1 |
| Signaling by FGFR | 10 | 94 | 6.289308176 | 4.421671355 | 0.000172 | 0.129315 | 0.009947 | 0.001121 | NRAS,ADRBK1,FGF7,CAMK4,MAPK3,YWHAB,PTPN11,MAPK1,FGFR2,PRKAR2B |
| Signalling to RAS | 5 | 21 | 3.144654088 | 10.51595996 | 0.000177 | 0.133357 | 0.009525 | 0.001121 | NRAS,MAPK3,YWHAB,PTPN11,MAPK1 |
| Signaling by Insulin receptor | 9 | 78 | 5.660377358 | 4.841985856 | 0.000196 | 0.147704 | 0.009847 | 0.001159 | NRAS,DOK1,MAPK3,YWHAB,PTPN11,ATP6V1E2,MAPK1,RPS6,ATP6V1C2 |
| p75(NTR)-mediated signaling | 14 | 178 | 8.805031447 | 3.185001208 | 0.000241 | 0.181224 | 0.011326 | 0.001333 | NRAS,RHOA,SFN,CAMK4,MAPK3,YWHAB,PTPN11,DIABLO,MATK,MAPK1,MMP7,RAC1,RTN4,NFKBIA |
| Downstream signaling of activated FGFR | 8 | 65 | 5.031446541 | 5.227027345 | 0.000286 | 0.214919 | 0.012642 | 0.001488 | NRAS,ADRBK1,CAMK4,MAPK3,YWHAB,PTPN11,MAPK1,PRKAR2B |
| IRS-mediated signalling | 7 | 50 | 4.402515723 | 6.02641452 | 0.000309 | 0.232096 | 0.012894 | 0.001518 | NRAS,DOK1,MAPK3,YWHAB,PTPN11,MAPK1,RPS6 |
| ERK activation | 3 | 6 | 1.886792453 | 21.94572059 | 0.000345 | 0.25927 | 0.013646 | 0.001606 | MAPK3,PTPN11,MAPK1 |
| IRS-related events | 7 | 52 | 4.402515723 | 5.800070313 | 0.000395 | 0.297365 | 0.014868 | 0.00175 | NRAS,DOK1,MAPK3,YWHAB,PTPN11,MAPK1,RPS6 |
| NGF signalling via TRKA from the plasma membrane | 10 | 106 | 6.289308176 | 3.926938896 | 0.000461 | 0.346875 | 0.016518 | 0.001944 | NRAS,RPS6KA2,ADRBK1,RHOA,CAMK4,MAPK3,YWHAB,PTPN11,MAPK1,PRKAR2B |
| Metabolism | 37 | 813 | 23.27044025 | 1.758622374 | 0.000526 | 0.395545 | 0.017979 | 0.002116 | RPL10,COX4I1,GSTP1,RPS7,QDPR,PSMD14,SLC5A6,PANK1,GLYAT,DCK,DHFR,ANAPC11,SULT1E1,NOSIP,PTS,YWHAB,RPL14,UGDH,ENOPH1,RPL22,TK1,CYP2C8,PYCR1,NDUFA8,RPS13,COASY,TXNRD1,MLX,PSMD10,ALDH1A1,DDC,SET,PRKAR2B,RPS6,PDXK,DGUOK,ALDH9A1 |
| Insulin receptor signalling cascade | 7 | 55 | 4.402515723 | 5.490733229 | 0.000561 | 0.422006 | 0.018348 | 0.00216 | NRAS,DOK1,MAPK3,YWHAB,PTPN11,MAPK1,RPS6 |
| Downstream signal transduction | 8 | 74 | 5.031446541 | 4.601867929 | 0.000697 | 0.52451 | 0.021855 | 0.002573 | NRAS,ADRBK1,CAMK4,MAPK3,YWHAB,PTPN11,MAPK1,PRKAR2B |
| Interleukin-2 signaling | 5 | 28 | 3.144654088 | 7.999319973 | 0.000737 | 0.554343 | 0.022174 | 0.00261 | NRAS,MAPK3,YWHAB,PTPN11,MAPK1 |
| S1P3 pathway | 5 | 29 | 3.144654088 | 7.734879643 | 0.000872 | 0.655658 | 0.025218 | 0.002779 | RHOA,MAPK3,MAPK1,RAC1,AKT3 |
| Osteopontin-mediated events | 5 | 29 | 3.144654088 | 7.734879643 | 0.000872 | 0.655658 | 0.024284 | 0.002779 | SPP1,MAPK3,MAPK1,RAC1,NFKBIA |
| Syndecan-2-mediated signaling events | 8 | 77 | 5.031446541 | 4.425438487 | 0.000911 | 0.684737 | 0.024455 | 0.002779 | RHOA,GNB2L1,SPP1,MAPK3,PTPN11,MAPK1,PAK4,FGFR2 |
| Signaling by PDGF | 8 | 77 | 5.031446541 | 4.425438487 | 0.000911 | 0.684737 | 0.023612 | 0.002779 | NRAS,ADRBK1,CAMK4,MAPK3,YWHAB,PTPN11,MAPK1,PRKAR2B |
| Signalling to ERKs | 5 | 30 | 3.144654088 | 7.487363495 | 0.001024 | 0.770152 | 0.025672 | 0.003022 | NRAS,MAPK3,YWHAB,PTPN11,MAPK1 |
| Neurotrophic factor-mediated Trk receptor signaling | 9 | 101 | 5.660377358 | 3.752835003 | 0.001338 | 1 | 0.032467 | 0.003776 | NRAS,SFN,CAMK4,MAPK3,YWHAB,PTPN11,MATK,MAPK1,RAC1 |
| Vitamin B5 (pantothenate) metabolism | 3 | 9 | 1.886792453 | 15.52258285 | 0.001365 | 1 | 0.032085 | 0.003776 | SLC5A6,PANK1,COASY |
| Integrins in angiogenesis | 7 | 64 | 4.402515723 | 4.733390715 | 0.001407 | 1 | 0.032074 | 0.003776 | RHOA,SPP1,MAPK3,PTPN11,MAPK1,RAC1,NFKBIA |
| FGF signaling pathway | 6 | 48 | 3.773584906 | 5.511003587 | 0.001542 | 1 | 0.034112 | 0.004016 | SPP1,MAPK3,PTPN11,MAPK1,PAK4,FGFR2 |
| DCC mediated attractive signaling | 3 | 10 | 1.886792453 | 14.14279771 | 0.001913 | 1 | 0.041095 | 0.004837 | ABLIM1,ABLIM3,RAC1 |
| ERKs are inactivated | 2 | 3 | 1.257861635 | 28.62815454 | 0.002059 | 1 | 0.043003 | 0.005017 | MAPK3,MAPK1 |
| Signal transduction by L1 | 5 | 35 | 3.144654088 | 6.454623702 | 0.002097 | 1 | 0.042619 | 0.005017 | RPS6KA2,RHOA,MAPK3,MAPK1,RAC1 |
| Signaling events regulated by Ret tyrosine kinase | 7 | 69 | 4.402515723 | 4.396494579 | 0.002189 | 1 | 0.043315 | 0.005048 | RHOA,DOK1,MAPK3,PTPN11,MAPK1,RAC1,NFKBIA |
| Opioid Signalling | 6 | 52 | 3.773584906 | 5.097031487 | 0.002345 | 1 | 0.045223 | 0.005048 | ADRBK1,CAMK4,PLCB2,MAPK1,PPP3R1,PRKAR2B |
| N-cadherin signaling events | 15 | 249 | 9.433962264 | 2.430962174 | 0.002353 | 1 | 0.044236 | 0.005048 | FHL2,RHOA,SFN,NME1,CAMK4,SPP1,MAPK3,YWHAB,PTPN11,MAPK1,MMP7,SNX1,PAK4,FGFR2,RAC1 |
| Deposition of New CENPA-containing Nucleosomes at the Centromere | 4 | 22 | 2.51572327 | 8.453474913 | 0.002395 | 1 | 0.043933 | 0.005048 | OIP5,RBBP7,RUVBL1,NPM1 |
| Nucleosome assembly | 4 | 22 | 2.51572327 | 8.453474913 | 0.002395 | 1 | 0.042887 | 0.005048 | OIP5,RBBP7,RUVBL1,NPM1 |
| Signal regulatory protein (SIRP) family interactions | 3 | 11 | 1.886792453 | 12.98828361 | 0.002579 | 1 | 0.045102 | 0.005309 | TYROBP,CD47,PTPN11 |
| E-cadherin signaling events | 16 | 279 | 10.06289308 | 2.304317935 | 0.002787 | 1 | 0.047624 | 0.005588 | FHL2,RHOA,SFN,NME1,CAMK4,SPP1,MAPK3,YWHAB,PTPN11,MAPK1,MMP7,SNX1,PAK4,FGFR2,FMN1,RAC1 |
| Signaling events mediated by PRL | 4 | 23 | 2.51572327 | 8.10487801 | 0.00284 | 1 | 0.047467 | 0.005588 | RHOA,MAPK3,MAPK1,RAC1 |
| Posttranslational regulation of adherens junction stability and dissassembly | 14 | 230 | 8.805031447 | 2.468806341 | 0.002984 | 1 | 0.048789 | 0.005609 | FHL2,SFN,NME1,CAMK4,SPP1,MAPK3,YWHAB,PTPN11,MAPK1,MMP7,SNX1,PAK4,FGFR2,RAC1 |
| ErbB2/ErbB3 signaling events | 5 | 38 | 3.144654088 | 5.961276668 | 0.003042 | 1 | 0.048667 | 0.005609 | NRAS,MAPK3,PTPN11,MAPK1,RAC1 |
| PLC beta mediated events | 5 | 38 | 3.144654088 | 5.961276668 | 0.003042 | 1 | 0.047653 | 0.005609 | ADRBK1,CAMK4,PLCB2,MAPK1,PRKAR2B |

**Supplementary Table 5.6: Protein domain**

| Analysis:Protein domain |  |  |  |  |  |  |  |  |  |
| --- | --- | --- | --- | --- | --- | --- | --- | --- | --- |
| Name of data set: MG1 vs HC |  |  |  |  |  |  |  |  |  |
| Number of gene in data set: 365 |  |  |  |  |  |  |  |  |  |
| Number of gene mapped to Protein domain : 103 |  |  |  |  |  |  |  |  |  |
|  |  |  |  |  |  |  |  |  |  |
| Protein domain | No. of genes  in the data set | No. of genes in the background data set | Percentage of genes | Fold Enrichment | Uncorrected  p-value  (Hypergeometric test) | Corrected  p-value  (Bonferroni method) | Corrected  p-value  (BH method) | Storey and Tibshirani method  q-value | Genes mapped from  input data set |
| ANX | 3 | 8 | 2.912621359 | 25.09618251 | 0.000302 | 0.031363 | 0.031363 | 0.00648 | ANXA6,ANXA11,ANXA13 |

**Supplementary Table 5.7: Site of expression**

| Analysis:Site of expression |  |  |  |  |  |  |  |  |  |
| --- | --- | --- | --- | --- | --- | --- | --- | --- | --- |
| Name of data set: MG1 vs HC |  |  |  |  |  |  |  |  |  |
| Number of gene in data set: 365 |  |  |  |  |  |  |  |  |  |
| Number of gene mapped to Site of expression : 362 |  |  |  |  |  |  |  |  |  |
|  |  |  |  |  |  |  |  |  |  |
| Site of expression | No. of genes  in the data set | No. of genes in the background data set | Percentage of genes | Fold Enrichment | Uncorrected  p-value  (Hypergeometric test) | Corrected  p-value  (Bonferroni method) | Corrected  p-value  (BH method) | Storey and Tibshirani method  q-value | Genes mapped from  input data set |
| CD8 | 138 | 3259 | 38.12154696 | 2.121550842 | 8.64E-20 | 3.42E-17 | 3.42E-17 | 7.95E-18 | DOHH,STAT6,CCNB1,DRG1,PROSC,AFP,MRPS7,NRAS,CSRP1,OGFOD2,GPSM3,APOE,HLA-DRB5,COX4I1,HPCAL1,HSPA2,CARHSP1,FUBP3,GSTP1,CYBB,CORO1A,FUBP1,ATAD2,RPS7,PPP2R4,ADRBK1,QDPR,IFIT3,RAP1GDS1,ATG3,HPX,NECAP2,HLA-DOB,CHD4,ST6GALNAC6,MRPS25,PSMD14,IMPDH2,GNB2L1,MRTO4,BCAS2,CYB5R1,ARL2BP,ANP32E,NME1,ADD1,DCK,DHFR,HN1,DDX58,ARPC3,CLGN,HLA-DRB3,NOSIP,EIF4EBP3,AAK1,APEX1,CCT4,MAPK3,ARF5,YWHAB,DBNL,TCOF1,RPL14,C1QC,TTC1,USP15,UGDH,ASF1A,PTPN11,MCM5,DIABLO,RBBP7,CKAP2,NAGK,PRH2,MCTS1,DERL1,RPL22,SRPK2,HBG2,SERBP1,TK1,MARCKSL1,SPC25,PRPSAP2,RNF126,RAB8B,C9,GTF2I,PYCR1,NDUFA8,TOM1,CHRAC1,ANXA6,RPS13,USP14,COASY,ANXA11,PDS5B,MAPK1,TXNRD1,POLE3,CCL5,SRI,MLX,TARDBP,PSMD10,ALDH1A1,SAR1B,ABCF3,RUVBL1,TPD52L2,SNX1,PAK4,SLC25A10,FGA,SNX9,SET,PRKAR2B,RPS6,VCP,RAC1,HSPBP1,PLEK,SRP54,CHORDC1,ZRANB2,FABP5,ZMYM3,NPM1,PGM2,RTN4,PDXK,RNPEP,ALDH9A1,UBE2O,PEX19 |
| B Cell | 106 | 2194 | 29.28176796 | 2.426735585 | 1.11E-18 | 4.38E-16 | 2.19E-16 | 5.09E-17 | DOHH,CD84,DRG1,PROSC,AFP,RAB3B,NRAS,RPL10,CSRP1,GPSM3,APOE,HLA-DRB5,COX4I1,HPCAL1,HSPA2,GALT,IGLL1,GSTP1,CYBB,CORO1A,FUBP1,RPS7,TIMP1,STATH,PPP2R4,QDPR,IFIT3,RHOA,CD96,HPX,NECAP2,HLA-DOB,CHD4,PSMD14,IMPDH2,PANK1,DHX9,GNB2L1,CYB5R1,RAB39B,SFN,ANP32E,NME1,ADD1,RPS15,DCK,ARPC3,SH2D1B,HLA-DRB3,NOSIP,APEX1,CCT4,MAPK3,ARF5,YWHAB,DBNL,TCOF1,RPL14,C1QC,UGDH,ENOPH1,MCM5,DIABLO,QTRT1,RBBP7,NAGK,PRH2,MCTS1,DERL1,RPL22,HBG2,SERBP1,TK1,MARCKSL1,RAB8B,GTF2I,NDUFA8,DAPP1,ANXA6,RPS13,USP14,COASY,ANXA11,MAPK1,TXNRD1,SRI,TARDBP,PSMD10,ALDH1A1,SAR1B,RUVBL1,TPD52L2,FGA,SET,RPS6,VCP,RAC1,HSPBP1,PLEK,SRP54,CHORDC1,FABP5,NPM1,PGM2,RTN4,ALDH9A1 |
| K-562 | 109 | 2483 | 30.11049724 | 2.204410359 | 3.36E-16 | 1.33E-13 | 4.43E-14 | 1.03E-14 | CCNB1,DRG1,PROSC,ERAL1,OGFOD2,COX4I1,HPCAL1,ELOVL1,CARHSP1,FUBP3,GSTP1,RPS6KA2,FUBP1,RPS7,PPP2R4,QDPR,RHOA,GADD45A,NECAP2,CHD4,DOK1,PSMD14,SLC5A6,IMPDH2,DHX9,GNB2L1,BCAS2,ANP32E,FN3K,NME1,ADD1,RPS15,TEX264,DCK,DHFR,HN1,ARPC3,NOSIP,CCNH,AAK1,APEX1,CCT4,MAPK3,CDCP1,ARF5,YWHAB,DBNL,TCOF1,RPL14,TTC1,USP15,ASF1A,PTPN11,MCM5,DIABLO,RBBP7,MCTS1,RPL22,SRPK2,HBG2,SERBP1,TK1,MARCKSL1,SPC25,PRPSAP2,GTF2I,PYCR1,NDUFA8,TOM1,CHRAC1,RPS13,USP14,ANXA11,PDS5B,MAPK1,TXNRD1,POLE3,SRI,TARDBP,PSMD10,ALDH1A1,SELENBP1,ABCF3,RUVBL1,TPD52L2,SNX1,PAK4,PAF1,SNX9,SET,PRKAR2B,RPS6,VCP,HSPBP1,SRP54,CHORDC1,PHKG2,ZRANB2,FABP5,ZMYM3,NPM1,CIAO1,PGM2,RTN4,PDXK,RNPEP,ACVR2B,UBE2O,PEX19 |
| HCT116 | 111 | 2596 | 30.66298343 | 2.146751319 | 1.06E-15 | 4.18E-13 | 1.04E-13 | 2.42E-14 | DOHH,DRG1,PROSC,RAB3B,MRPS7,NRAS,RPL10,KRR1,CSRP1,COX4I1,HSPA2,ELOVL1,CARHSP1,FUBP3,GSTP1,FUBP1,RPS7,PPP2R4,QDPR,RHOA,RAP1GDS1,ATG3,SGK2,CHD4,PSMD14,IMPDH2,DHX9,GNB2L1,MRTO4,BCAS2,CLDN4,SFN,ANP32E,NME1,RPS15,DCK,DHFR,ARPC3,APEX1,CCT4,MRPL53,MAPK3,ARF5,YWHAB,TCOF1,RPL14,USP15,UGDH,ENOPH1,PRKRA,MCM5,DIABLO,RBBP7,CPNE4,MCTS1,DERL1,RPL22,HBG2,SERBP1,TK1,MARCKSL1,STUB1,PRPSAP2,RAB8B,GTF2I,PYCR1,NDUFA8,ANXA6,RPS13,USP14,ANXA11,PDS5B,MAPK1,TXNRD1,POLE3,SRI,TARDBP,CKM,PSMD10,ALDH1A1,SAR1B,ABCF3,RAB11A,RUVBL1,TPD52L2,SNX1,STK25,FGFR2,SLC25A10,LST1,SET,PRKAR2B,RPS6,ATP6V1C2,VCP,RAC1,COG3,HSPBP1,CHORDC1,ZRANB2,GBE1,FABP5,NPM1,CIAO1,PGM2,GOLT1B,RTN4,PDXK,RNPEP,ALDH9A1,UBE2O |
| Erythrocytes | 78 | 1492 | 21.54696133 | 2.63618504 | 2.22E-15 | 8.78E-13 | 1.76E-13 | 3.99E-14 | DOHH,STAT6,PROSC,NFE2,CSRP1,HPCAL1,HSPA2,GALT,CARHSP1,GSTP1,FUBP1,RPS7,PPP2R4,QDPR,ATG3,NECAP2,PSMD14,CDC34,SFN,ANP32E,FN3K,NME1,ADD1,APEX1,CCT4,ARIH2,BAG5,YWHAB,DBNL,USP15,ENOPH1,PTPN11,ZFYVE19,RBBP7,NAGK,MCTS1,RPL22,HBG2,PRPSAP2,RNF126,RAB8B,TOM1,RPS13,USP14,COASY,ANXA11,MAPK1,TXNRD1,SRI,TARDBP,PSMD10,ALDH1A1,SAR1B,SELENBP1,RUVBL1,TPD52L2,SNX1,FGA,SNX9,SET,PRKAR2B,RPS6,VCP,RAC1,HSPBP1,CHORDC1,PHKG2,GBE1,FABP5,ZMYM3,NPM1,NRBF2,CIAO1,PGM2,PDXK,RNPEP,ALDH9A1,PEX19 |
| Dendritic cells | 91 | 1923 | 25.13812155 | 2.381299513 | 2.61E-15 | 1.03E-12 | 1.72E-13 | 3.99E-14 | CD84,DRG1,PROSC,MRPS7,NRAS,TYROBP,RPL10,CSRP1,GPSM3,COX4I1,HPCAL1,CARHSP1,GSTP1,RPS6KA2,CYBB,CORO1A,FUBP1,RPS7,TIMP1,PPP2R4,IFIT3,RHOA,RAP1GDS1,ATG3,NECAP2,HLA-DOB,PSMD14,DHX9,GNB2L1,ANP32E,NME1,RPS15,DCK,HN1,ARPC3,HLA-DRB3,PTS,APEX1,CCT4,MRPL53,MAPK3,ARF5,YWHAB,DBNL,RPL14,C1QC,USP15,ENOPH1,DIABLO,RBBP7,NAGK,RPL22,ATP6V1E2,RAB8B,NDUFA8,TOM1,PLCB2,ANXA6,RPS13,USP14,ANXA11,MAPK1,TXNRD1,CCL5,SRI,TARDBP,PSMD10,EPB41L3,SAR1B,ABCF3,RAB11A,TPD52L2,SNX1,PECAM1,FBP1,LST1,SET,RPS6,VCP,RAC1,PLEK,CHORDC1,FABP5,NPM1,PGM2,GOLT1B,RTN4,PDXK,RNPEP,ALDH9A1,UBE2O |
| OVCAR3 | 132 | 3644 | 36.4640884 | 1.815720691 | 3.33E-13 | 1.32E-10 | 1.89E-11 | 4.38E-12 | DOHH,CCNB1,ADRB2,DRG1,PROSC,MRPS7,FST,NRAS,RPL10,CSRP1,ERAL1,APOE,COX4I1,HPCAL1,CD47,HSPA2,GALT,LRRC20,CARHSP1,CKS2,FUBP3,GSTP1,CORO1A,RPS7,TIMP1,PPP2R4,SLC39A9,ADRBK1,QDPR,IFIT3,RAP1GDS1,ATG3,NECAP2,CHD4,PSMD14,IMPDH2,GNB2L1,MRTO4,BCAS2,CYB5R1,EPS8L1,FBXL18,SFN,ANP32E,FN3K,NME1,ADD1,DCK,DHFR,HN1,ARPC3,CLGN,NOSIP,APEX1,CCT4,MRPL53,ABLIM1,ARIH2,BAG5,MAPK3,ARF5,YWHAB,DBNL,TCOF1,TTC1,USP15,UGDH,ENOPH1,PTPN11,PRKRA,DIABLO,RBBP7,NAGK,PRH2,MCTS1,DERL1,RPL22,SRPK2,SERBP1,TK1,MARCKSL1,STUB1,PRPSAP2,C9,GTF2I,BCL7C,PYCR1,NDUFA8,TOM1,CHRAC1,ANXA6,RPS13,USP14,COASY,ANXA11,MAPK1,TXNRD1,POLE3,SRI,TARDBP,PSMD10,ALDH1A1,SAR1B,SELENBP1,RAB11A,RUVBL1,TPD52L2,SNX1,PAK4,FGA,PAF1,SET,RPS6,VCP,RAC1,HSPBP1,SRP54,CHORDC1,GFM2,GBE1,FABP5,NPM1,NRBF2,PGM2,ARHGAP29,GOLT1B,RTN4,PDXK,RNPEP,PLEKHG2,ALDH9A1,PEX19 |
| CD4 | 84 | 1854 | 23.20441989 | 2.282449046 | 4.74E-13 | 1.88E-10 | 2.35E-11 | 5.45E-12 | PROSC,NRAS,CSRP1,GPSM3,APOE,HLA-DRB5,COX4I1,HPCAL1,GALT,CARHSP1,FUBP3,GSTP1,CYBB,CORO1A,FUBP1,RPS7,TIMP1,PPP2R4,QDPR,HPX,NECAP2,CHD4,PSMD14,IMPDH2,GNB2L1,BCAS2,CYB5R1,ARL2BP,LCAT,SFN,ANP32E,NME1,ADD1,HN1,ARPC3,HLA-DRB3,AAK1,APEX1,CCT4,MAPK3,ARF5,YWHAB,DBNL,TCOF1,RPL14,C1QC,ENOPH1,DIABLO,RBBP7,NAGK,MCTS1,RPL22,SERBP1,PRPSAP2,RAB8B,C9,NDUFA8,ANXA6,RPS13,USP14,ANXA11,PDS5B,MAPK1,CCL5,SRI,TARDBP,PSMD10,RUVBL1,TPD52L2,SNX1,FGA,SET,RPS6,VCP,RAC1,PLEK,SRP54,CHORDC1,FABP5,NPM1,PGM2,RTN4,PDXK,ALDH9A1 |
| H293 | 201 | 6698 | 55.52486188 | 1.499586386 | 5.39E-13 | 2.13E-10 | 2.37E-11 | 5.5E-12 | HOXA5,DOHH,STAT6,ZHX3,COQ6,CCNB1,DRG1,PROSC,OIP5,AFP,FHL2,STAU2,MRPS7,NRAS,C2orf44,KRR1,CSRP1,ERAL1,OGFOD2,APOE,HLA-DRB5,COX4I1,HPCAL1,HSPA2,GULP1,GALT,SP110,ELOVL1,CARHSP1,CKS2,FUBP3,GSTP1,CORO1A,FUBP1,ATAD2,RPS7,TIMP1,STATH,PPP2R4,SLC39A9,ADRBK1,QDPR,RAP1GDS1,ATG3,TIPIN,HPX,NECAP2,CHD4,MRPS25,PSMD14,SLC5A6,IMPDH2,PANK1,XPR1,GNB2L1,MRTO4,BCAS2,CYB5R1,CDC34,SYNPR,LCAT,CTNNAL1,SFN,ANP32E,NME1,STARD10,ADD1,ACTL6B,CAMK4,TEX264,DCK,DHFR,FBXO3,HN1,ARPC3,CLGN,RAB24,HLA-DRB3,NOSIP,EIF4EBP3,CCNH,AAK1,APEX1,CCT4,FNIP1,MRPL53,ABLIM1,ARIH2,BAG5,MAPK3,ARF5,YWHAB,DBNL,TCOF1,RPL14,C1QC,TTC1,USP15,MORC4,UGDH,ASF1A,ENOPH1,PTPN11,ZFYVE19,PRKRA,MCM5,DIABLO,QTRT1,PDLIM3,RBBP7,PI16,CKAP2,NAGK,MEIS2,MCTS1,DERL1,RPL22,SRPK2,HSPB8,HBG2,SERBP1,TK1,MARCKSL1,SPC25,STUB1,PRPSAP2,RNF126,C9,GTF2I,BCL7C,PYCR1,NDUFA8,TOM1,CHRAC1,ANXA6,RPS13,USP14,COASY,RECQL5,ANXA11,PDS5B,RNF11,ABLIM3,MAPK1,TXNRD1,POLE3,CCL5,SRI,MLX,TARDBP,MOXD1,CKM,CETN3,PSMD10,ALDH1A1,EPB41L3,SAR1B,ABCF3,RAB11A,RUVBL1,TPD52L2,SNX1,PAK4,FGFR2,SLC25A10,FGA,TAF7,PAF1,SNX9,SET,PPP3R1,PRKAR2B,RPS6,VCP,RAC1,COG3,HSPBP1,PLEK,AKT3,SRP54,GKAP1,CHORDC1,PHKG2,GFM2,ZRANB2,GBE1,FABP5,ZMYM3,NPM1,CORO2B,NRBF2,CIAO1,PGM2,GOLT1B,RTN4,PDXK,RNPEP,ANXA13,DGUOK,ALDH9A1,PEX19 |
| CRC | 144 | 4170 | 39.77900552 | 1.729653321 | 6.5E-13 | 2.57E-10 | 2.57E-11 | 5.97E-12 | DRG1,PROSC,SRPX2,AFP,RAB3B,NRAS,RPL10,KLK1,CSRP1,ERAL1,APOE,COX4I1,HPCAL1,HSPA2,ELOVL1,LRRC20,CARHSP1,FUBP3,GSTP1,CORO1A,FUBP1,ATAD2,RPS7,TIMP1,PPP2R4,ADRBK1,QDPR,RHOA,LAYN,ATG3,HPX,CHD4,MRPS25,PSMD14,IMPDH2,DHX9,GNB2L1,MRTO4,BCAS2,CLDN4,ARL2BP,RAB39B,VSNL1,PTN,SFN,ANP32E,NME1,STARD10,ADD1,ACTL6B,RPS15,DCK,DHFR,HN1,ARPC3,SPP1,RBKS,APEX1,CCT4,MRPL53,MAPK3,CDCP1,ARF5,YWHAB,DBNL,TCOF1,GGT6,RPL14,TTC1,UGDH,ASF1A,ENOPH1,PTPN11,PRKRA,MCM5,DIABLO,RBBP7,CKAP2,MCTS1,DERL1,HIGD2A,RPL22,ATP6V1E2,HBG2,SERBP1,MARCKSL1,RNF126,RAB8B,GTF2I,PYCR1,NDUFA8,CHRAC1,ANXA6,RPS13,USP14,TEAD3,COASY,ANXA11,PDS5B,MAPK1,TXNRD1,POLE3,SRI,TARDBP,RASGEF1A,CKM,PSMD10,ALDH1A1,EPB41L3,SAR1B,SELENBP1,MMP7,RAB11A,RUVBL1,TPD52L2,SNX1,STK25,FGFR2,SLC25A10,DDC,PECAM1,FBP1,SET,PPP3R1,RPS6,MYLK,VCP,RAC1,COG3,HSPBP1,SRP54,CHORDC1,GBE1,FABP5,ZMYM3,NPM1,CIAO1,PGM2,RTN4,PDXK,RNPEP,ANXA13,ALDH9A1,UBE2O |
| 031003_BALF2 | 60 | 1090 | 16.57458564 | 2.787988512 | 7.91E-13 | 3.13E-10 | 2.85E-11 | 6.61E-12 | DOHH,AFP,CSRP1,APOE,HSPA2,GALT,GSTP1,CORO1A,FUBP1,TIMP1,PPP2R4,QDPR,IFIT3,HPX,IMPDH2,PANK1,GNB2L1,LCAT,SFN,ANP32E,NME1,SPP1,APEX1,CCT4,MAPK3,YWHAB,DBNL,USP15,UGDH,ENOPH1,RBBP7,NAGK,PRH2,MCTS1,HBG2,MARCKSL1,C9,TOM1,ANXA6,USP14,MAPK1,TXNRD1,SRI,TARDBP,PSMD10,ALDH1A1,FGA,FBP1,SET,VCP,CHORDC1,GBE1,FABP5,NPM1,CIAO1,PGM2,RTN4,PDXK,RNPEP,ALDH9A1 |
| global_SCX_fractionated | 99 | 2427 | 27.3480663 | 2.050690538 | 1.3E-12 | 5.16E-10 | 4.3E-11 | 9.98E-12 | DOHH,PROSC,FHL2,NRAS,CSRP1,ERAL1,COX4I1,HPCAL1,GALT,ELOVL1,CARHSP1,FUBP3,GSTP1,CORO1A,FUBP1,RPS7,PPP2R4,QDPR,RAP1GDS1,ATG3,CHD4,MRPS25,PSMD14,IMPDH2,GNB2L1,MRTO4,BCAS2,CYB5R1,FBXL18,SFN,ANP32E,NHEJ1,NME1,HN1,ARPC3,SULT1E1,NOSIP,EIF4EBP3,APEX1,CCT4,ARF5,YWHAB,DBNL,TCOF1,RPL14,USP15,UGDH,ENOPH1,PTPN11,ZFYVE19,PRKRA,SMYD3,MCM5,QTRT1,RBBP7,MCTS1,RPL22,SERBP1,STUB1,PRPSAP2,GTF2I,PYCR1,NDUFA8,TOM1,RPS13,USP14,ANXA11,MAPK1,TXNRD1,SRI,TARDBP,PSMD10,EPB41L3,SAR1B,ABCF3,RAB11A,RUVBL1,TPD52L2,SNX1,PAK4,SET,RPS6,VCP,RAC1,COG3,HSPBP1,CHORDC1,GFM2,GBE1,FABP5,NPM1,CIAO1,PGM2,RTN4,PDXK,RNPEP,NFKBIA,ALDH9A1,PEX19 |
| R | 84 | 1894 | 23.20441989 | 2.234277057 | 1.5E-12 | 5.95E-10 | 4.58E-11 | 1.06E-11 | DOHH,CCNB1,DRG1,CSRP1,OGFOD2,HPCAL1,CARHSP1,FUBP3,GSTP1,FUBP1,RPS7,TIMP1,PPP2R4,QDPR,RAP1GDS1,ATG3,NECAP2,CHD4,PSMD14,IMPDH2,GNB2L1,MRTO4,BCAS2,ANP32E,NME1,ADD1,HN1,ARPC3,NOSIP,EIF4EBP3,APEX1,CCT4,ARIH2,YWHAB,DBNL,TCOF1,TTC1,USP15,UGDH,ASF1A,PTPN11,MCM5,DIABLO,QTRT1,RBBP7,NAGK,MCTS1,RPL22,HBG2,SERBP1,TK1,MARCKSL1,STUB1,PRPSAP2,GTF2I,BCL7C,PYCR1,ANXA6,RPS13,USP14,COASY,ANXA11,PDS5B,TXNRD1,ABCF3,RUVBL1,TPD52L2,SNX1,PAF1,SNX9,SET,PRKAR2B,RPS6,VCP,RAC1,SRP54,CHORDC1,ZRANB2,NPM1,PGM2,RTN4,RNPEP,DGUOK,PEX19 |
| Jurkat | 107 | 2815 | 29.55801105 | 1.909263385 | 9.9E-12 | 3.92E-09 | 2.8E-10 | 6.5E-11 | DOHH,CCNB1,DRG1,PROSC,STK33,CALCOCO2,NRAS,RPL10,COX4I1,CD47,AEBP2,HSPA2,CKS2,FUBP3,GSTP1,RPS6KA2,CORO1A,FUBP1,RPS7,SLC39A9,QDPR,CD96,RAP1GDS1,CHD4,DOK1,IMPDH2,DHX9,GNB2L1,MRTO4,BCAS2,FAM58A,ANP32E,NME1,ADD1,CAMK4,RPS15,TEX264,DCK,HN1,ARPC3,CCNH,ARHGDIG,APEX1,CCT4,BAG5,MAPK3,YWHAB,DBNL,TCOF1,RPL14,USP15,ASF1A,PTPN11,PRKRA,MCM5,DIABLO,QTRT1,RBBP7,DERL1,SERBP1,MARCKSL1,SPC25,PRPSAP2,GTF2I,NDUFA8,TOM1,CHRAC1,ANXA6,RPS13,USP14,ANXA11,PDS5B,MAPK1,TXNRD1,SRI,TARDBP,F8,LEPROTL1,SAR1B,ABCF3,RUVBL1,TPD52L2,SNX1,PAK4,STK25,FGFR2,SLC25A10,PAF1,SET,RPS6,VCP,COG3,SRP54,CHORDC1,PHKG2,LHX1,ZRANB2,FABP5,NPM1,CIAO1,RTN4,PDXK,RNPEP,NFKBIA,ALDH9A1,ACVR2B,PEX19 |
| CaOV3 | 119 | 3291 | 32.87292818 | 1.814267924 | 1.11E-11 | 4.4E-09 | 2.93E-10 | 6.81E-11 | DOHH,DRG1,PROSC,RAB3B,MRPS7,NRAS,RPL10,KRR1,CSRP1,ERAL1,APOE,HLA-DRB5,HPCAL1,HSPA2,CARHSP1,CKS2,FUBP3,GSTP1,RPS7,TIMP1,OLR1,PPP2R4,QDPR,IFIT3,LAYN,ATG3,NECAP2,CHD4,PSMD14,IMPDH2,GNB2L1,MRTO4,BCAS2,EPS8L1,SFN,ANP32E,FN3K,NME1,ADD1,DHFR,HN1,ARPC3,HLA-DRB3,NOSIP,APEX1,CCT4,MRPL53,ARIH2,BAG5,MAPK3,CDCP1,ARF5,YWHAB,DBNL,TCOF1,TTC1,UGDH,ENOPH1,PTPN11,PRKRA,MCM5,DIABLO,RBBP7,NAGK,PRH2,MCTS1,RPL22,SERBP1,TK1,MARCKSL1,STUB1,MATK,GTF2I,BCL7C,PYCR1,NDUFA8,TOM1,ANXA6,RPS13,USP14,ANXA11,PDS5B,MAPK1,TXNRD1,POLE3,SRI,TARDBP,PSMD10,ALDH1A1,SAR1B,SELENBP1,ABCF3,RAB11A,RUVBL1,TPD52L2,SNX1,PAK4,SLC25A10,FGA,SNX9,FBP1,SET,RPS6,VCP,RAC1,HSPBP1,SRP54,CHORDC1,ZRANB2,GBE1,FABP5,NPM1,NRBF2,PGM2,GOLT1B,RTN4,PDXK,RNPEP,PEX19 |
| 041803_BALF3 | 57 | 1072 | 15.74585635 | 2.695902467 | 1.29E-11 | 5.13E-09 | 3.2E-10 | 7.44E-11 | DOHH,DRG1,CSRP1,APOE,HLA-DRB5,GSTP1,CORO1A,FUBP1,RPS7,IFIT3,RAP1GDS1,ATG3,HPX,IMPDH2,GNB2L1,LCAT,ANP32E,NME1,HLA-DRB3,EIF4EBP3,APEX1,CCT4,DBNL,USP15,UGDH,PRKRA,MCM5,RBBP7,NAGK,PRH2,RPL22,SERBP1,MARCKSL1,STUB1,RNF126,C9,TOM1,ANXA6,USP14,ANXA11,SRI,PSMD10,ALDH1A1,RUVBL1,TPD52L2,FGA,PAF1,SET,RPS6,VCP,PLEK,CHORDC1,GBE1,FABP5,PGM2,RTN4,RNPEP |
| ES2 | 123 | 3469 | 33.97790055 | 1.77846277 | 1.58E-11 | 6.25E-09 | 3.67E-10 | 8.53E-11 | HOXA5,DOHH,CCNB1,DRG1,PROSC,RAB3B,MRPS7,FST,NRAS,RPL10,KRR1,CSRP1,APOE,COX4I1,HPCAL1,TM4SF1,CARHSP1,CKS2,FUBP3,GSTP1,RPS7,TIMP1,PPP2R4,SLC39A9,QDPR,RAP1GDS1,ATG3,CHD4,MRPS25,PSMD14,IMPDH2,GNB2L1,MRTO4,BCAS2,CYB5R1,CTNNAL1,FBXL18,SFN,ANP32E,NHEJ1,NME1,ADD1,TEX264,DHFR,HN1,ARPC3,NOSIP,EIF4EBP3,CCNH,AAK1,APEX1,CCT4,MRPL53,ARIH2,MAPK3,ARF5,YWHAB,DBNL,TCOF1,TTC1,UGDH,PTPN11,ZFYVE19,PRKRA,MCM5,C14orf119,RBBP7,NAGK,MCTS1,DERL1,HIGD2A,RPL22,HBG2,SERBP1,MARCKSL1,STUB1,RNF126,RAB8B,C9,GTF2I,PYCR1,NDUFA8,TOM1,ANXA6,RPS13,USP14,COASY,ANXA11,MAPK1,TXNRD1,POLE3,SRI,TARDBP,PSMD10,SAR1B,RAB11A,RUVBL1,TPD52L2,SNX1,PAK4,FGA,SNX9,SET,RPS6,MYLK,VCP,RAC1,HSPBP1,SRP54,CHORDC1,ZRANB2,GBE1,FABP5,NPM1,PGM2,ARHGAP29,GOLT1B,RTN4,PDXK,RNPEP,ANXA13,ALDH9A1,PEX19 |
| Ascites cancer cell | 122 | 3445 | 33.70165746 | 1.776434882 | 2.18E-11 | 8.64E-09 | 4.8E-10 | 1.11E-10 | CD84,DRG1,PROSC,STAU2,MRPS7,FST,NRAS,TYROBP,RPL10,CSRP1,APOE,HLA-DRB5,COX4I1,HPCAL1,CD47,TSPAN17,HSPA2,ELOVL1,CARHSP1,FUBP3,GSTP1,CYBB,CORO1A,RPS7,TIMP1,OLR1,QDPR,IFIT3,HPX,NECAP2,CHD4,PSMD14,IMPDH2,GNB2L1,BCAS2,CYB5R1,EPS8L1,RRAGB,SFN,ANP32E,NME1,ADD1,TEX264,HN1,SPP1,HLA-DRB3,NOSIP,EIF4EBP3,CCNH,AAK1,APEX1,CCT4,CXXC5,MRPL53,ABLIM1,BAG5,MAPK3,CDCP1,YWHAB,DBNL,TCOF1,C1QC,TTC1,UGDH,PTPN11,MCM5,DIABLO,RBBP7,NAGK,MCTS1,DERL1,RPL22,SRPK2,SERBP1,TK1,MARCKSL1,STUB1,RAB8B,C9,GTF2I,BCL7C,PYCR1,NDUFA8,TOM1,ANXA6,RPS13,USP14,ANXA11,PDS5B,TXNRD1,SRI,TARDBP,ALDH1A1,EPB41L3,SELENBP1,MMP7,RAB11A,RUVBL1,TPD52L2,SNX1,PAK4,SLC25A10,FGA,PAF1,FBP1,LST1,SET,RPS6,VCP,RAC1,PLEK,CHORDC1,ZRANB2,FABP5,ZMYM3,NPM1,PGM2,RTN4,PDXK,RNPEP,ALDH9A1,PEX19 |
| Monocyte | 105 | 2786 | 29.00552486 | 1.893486261 | 2.85E-11 | 1.13E-08 | 5.93E-10 | 1.38E-10 | ZHX3,DRG1,PROSC,RAB3B,NRAS,CSRP1,HLA-DRB5,COX4I1,HPCAL1,ELOVL1,GSTP1,CYBB,CORO1A,FUBP1,RPS7,PPP2R4,ADRBK1,QDPR,HPX,NECAP2,CHD4,PSMD14,IMPDH2,PANK1,GNB2L1,BCAS2,CYB5R1,ARL2BP,LCAT,SFN,ANP32E,NME1,ADD1,TEX264,DDX58,ARPC3,CLGN,RAB24,HLA-DRB3,EIF4EBP3,APEX1,CCT4,MRPL53,CDCP1,ARF5,YWHAB,DBNL,TCOF1,RPL14,USP15,PRKRA,MCM5,DIABLO,RBBP7,PI16,NAGK,MCTS1,DERL1,HIGD2A,RPL22,SERBP1,MARCKSL1,STUB1,PRPSAP2,RAB8B,C9,GTF2I,PYCR1,NDUFA8,ANXA6,RPS13,USP14,COASY,ANXA11,PDS5B,MAPK1,CCL5,SRI,MLX,TARDBP,PSMD10,ALDH1A1,SAR1B,RUVBL1,TPD52L2,SLC25A10,FGA,FBP1,SET,PPP3R1,RPS6,VCP,RAC1,PLEK,SRP54,GFM2,FABP5,NPM1,PGM2,GOLT1B,RTN4,PDXK,RNPEP,ALDH9A1,PEX19 |
| HEK293 | 144 | 4377 | 39.77900552 | 1.647876758 | 3.04E-11 | 1.2E-08 | 6.02E-10 | 1.4E-10 | HOXA5,DOHH,CCNB1,DRG1,OIP5,FHL2,STAU2,MRPS7,NRAS,KRR1,CSRP1,ERAL1,OGFOD2,APOE,COX4I1,HSPA2,CARHSP1,FUBP3,GSTP1,FUBP1,ATAD2,RPS7,PPP2R4,ADRBK1,QDPR,RAP1GDS1,NECAP2,CHD4,MRPS25,PSMD14,IMPDH2,XPR1,GNB2L1,MRTO4,BCAS2,CYB5R1,CDC34,CTNNAL1,SFN,ANP32E,NME1,ADD1,DCK,DHFR,HN1,ARPC3,CLGN,RAB24,NOSIP,EIF4EBP3,CCNH,AAK1,APEX1,CCT4,MRPL53,ABLIM1,ARIH2,BAG5,ARF5,YWHAB,DBNL,TCOF1,RPL14,TTC1,USP15,UGDH,ASF1A,ENOPH1,PTPN11,ZFYVE19,PRKRA,MCM5,DIABLO,PDLIM3,RBBP7,CKAP2,NAGK,MCTS1,HIGD2A,RPL22,SRPK2,HSPB8,SERBP1,TK1,MARCKSL1,SPC25,STUB1,PRPSAP2,RNF126,RAB8B,GTF2I,PYCR1,NDUFA8,TOM1,CHRAC1,ANXA6,RPS13,USP14,COASY,ANXA11,PDS5B,MAPK1,TXNRD1,SRI,TARDBP,MOXD1,PSMD10,EPB41L3,SAR1B,ABCF3,RUVBL1,TPD52L2,SNX1,PAK4,SLC25A10,TAF7,PAF1,SNX9,SET,PPP3R1,PRKAR2B,RPS6,VCP,RAC1,COG3,HSPBP1,SRP54,CHORDC1,PHKG2,GFM2,ZRANB2,GBE1,FABP5,ZMYM3,NPM1,NRBF2,CIAO1,PGM2,GOLT1B,RTN4,PDXK,RNPEP,ALDH9A1,PEX19 |
| T cells | 74 | 1694 | 20.44198895 | 2.204864448 | 9.3E-11 | 3.68E-08 | 1.75E-09 | 4.07E-10 | STAT6,DRG1,PROSC,MRPS7,NRAS,RPL10,CSRP1,COX4I1,HPCAL1,FUBP3,GSTP1,CORO1A,FUBP1,RPS7,ADRBK1,QDPR,NECAP2,CHD4,IMPDH2,DHX9,GNB2L1,MRTO4,BCAS2,SEPT1,NHEJ1,NME1,ADD1,RPS15,DCK,ARPC3,NOSIP,EIF4EBP3,PTS,AAK1,APEX1,CCT4,CCR10,ABLIM1,YWHAB,DBNL,TCOF1,RPL14,DIABLO,RBBP7,MCTS1,RPL22,HBG2,SERBP1,RAB8B,MATK,GTF2I,BCL7C,PYCR1,NDUFA8,ANXA6,RPS13,USP14,ANXA11,PDS5B,MAPK1,CCL5,TARDBP,SLA,RUVBL1,PAF1,SNX9,SET,PRKAR2B,RPS6,VCP,SRP54,ZRANB2,NPM1,NFKBIA |
| Lens | 53 | 1028 | 14.64088398 | 2.618110729 | 2.31E-10 | 9.16E-08 | 4.16E-09 | 9.66E-10 | DOHH,DRG1,HLA-DRB5,LRRC20,CARHSP1,GSTP1,CYBB,CORO1A,FUBP1,IFIT3,RAP1GDS1,PSMD14,IMPDH2,GNB2L1,SFN,ANP32E,NME1,DDX58,HLA-DRB3,APEX1,CCT4,ARIH2,YWHAB,DBNL,UGDH,MCM5,QTRT1,RBBP7,SRPK2,NDUFA8,ANXA6,USP14,ANXA11,MAPK1,SRI,TARDBP,PSMD10,ALDH1A1,RUVBL1,RPS6,VCP,RAC1,HSPBP1,PLEK,ZRANB2,GBE1,FABP5,NPM1,CIAO1,PGM2,RNPEP,ALDH9A1,PEX19 |
| Neutrophil | 80 | 1979 | 22.09944751 | 2.038039004 | 5.72E-10 | 2.26E-07 | 9.84E-09 | 2.28E-09 | NRAS,CSRP1,OGFOD2,APOE,HLA-DRB5,COX4I1,HPCAL1,SP110,FUBP3,IGLL1,GSTP1,CYBB,CORO1A,FUBP1,RPS7,TIMP1,STATH,PPP2R4,HPX,CHD4,IMPDH2,GNB2L1,MRTO4,BCAS2,CYB5R1,LCAT,SFN,ANP32E,NME1,ADD1,DDX58,ARPC3,HLA-DRB3,EIF4EBP3,CCNH,APEX1,CCT4,YWHAB,DBNL,TCOF1,C1QC,PRKRA,RBBP7,PI16,NAGK,RPL22,HBG2,SERBP1,MARCKSL1,RAB8B,C9,GTF2I,NDUFA8,ANXA6,USP14,RECQL5,ANXA11,MAPK1,SRI,MLX,TARDBP,ALDH1A1,RUVBL1,FGA,PAF1,SET,RPS6,MYLK,VCP,RAC1,PLEK,GKAP1,ZRANB2,GBE1,FABP5,NPM1,PGM2,RTN4,PDXK,ALDH9A1 |
| MDA-MB-468 | 41 | 744 | 11.32596685 | 2.816007922 | 4.76E-09 | 1.88E-06 | 7.85E-08 | 1.82E-08 | PROSC,FHL2,RPL10,COX4I1,FUBP3,GSTP1,CORO1A,FUBP1,RPS7,RHOA,CHD4,IMPDH2,GNB2L1,SFN,NME1,EIF4EBP3,APEX1,CCT4,TTC1,PTPN11,RBBP7,NAGK,SERBP1,RPS13,USP14,MAPK1,TXNRD1,PSMD10,ALDH1A1,SELENBP1,RAB11A,PAK4,S100A7A,SET,VCP,RAC1,HSPBP1,CHORDC1,NPM1,PDXK,RNPEP |
| Melanoma | 72 | 1795 | 19.88950276 | 2.025582758 | 7.24E-09 | 2.87E-06 | 1.15E-07 | 2.66E-08 | DRG1,CALCOCO2,RPL10,APOE,COX4I1,HPCAL1,HSPA2,GSTP1,CORO1A,FUBP1,RPS7,PPP2R4,QDPR,RHOA,RAP1GDS1,HPX,NECAP2,IMPDH2,DHX9,GNB2L1,BCAS2,CYB5R1,SFN,NME1,ADD1,RPS15,ARPC3,HLA-DRB3,APEX1,CCT4,ARIH2,ARF5,YWHAB,DBNL,TCOF1,RPL14,C1QC,UGDH,ENOPH1,PRKRA,DIABLO,NAGK,RPL22,RAB8B,GTF2I,TOM1,ANXA6,RPS13,USP14,MAPK1,TXNRD1,PSMD10,ALDH1A1,SELENBP1,RAB11A,RUVBL1,TPD52L2,SNX1,FGFR2,FGA,FBP1,SET,RPS6,VCP,RAC1,FABP5,NPM1,PGM2,RTN4,PDXK,RNPEP,ALDH9A1 |
|  | 53 | 1163 | 14.64088398 | 2.314529068 | 1.69E-08 | 6.69E-06 | 2.57E-07 | 5.97E-08 | PROSC,FHL2,RAB3B,CSRP1,APOE,HSPA2,RPS6KA2,RHOA,HPX,SLC5A6,GNB2L1,CYB5R1,CLDN4,EPS8L1,NME1,TEX264,DCK,ARPC3,CCNH,AAK1,CCT4,MAPK3,ARF5,YWHAB,DBNL,C1QC,DIABLO,NAGK,MCTS1,DERL1,RPL22,RPS13,ANXA11,MAPK1,TXNRD1,SRI,PSMD10,EPB41L3,SAR1B,RUVBL1,TPD52L2,SNX1,PAK4,FGA,SET,MYLK,RAC1,NUDT18,FABP5,CIAO1,RTN4,PDXK,ACVR2B |
| Platelets | 79 | 2138 | 21.82320442 | 1.863342304 | 4.29E-08 | 1.7E-05 | 6.29E-07 | 1.42E-07 | CD84,RAB3B,CALCOCO2,NRAS,CSRP1,APOE,COX4I1,HPCAL1,CD47,HSPA2,ELOVL1,GSTP1,CORO1A,FAM63A,TIMP1,PPP2R4,QDPR,RHOA,RAP1GDS1,HPX,NECAP2,CHD4,GNB2L1,BCAS2,CYB5R1,SFN,FN3K,NME1,ADD1,TEX264,ARPC3,PTS,CCT4,ARF5,YWHAB,DBNL,C1QC,RNF32,DIABLO,PRH2,MCTS1,FAM81A,HBG2,SERBP1,CYP2C8,RAB8B,NDUFA8,DAPP1,USP14,COASY,ANXA11,MAPK1,TXNRD1,POLE3,CCL5,SRI,TARDBP,SAR1B,MMP7,RAB11A,TPD52L2,STK25,FGA,PECAM1,PPP3R1,PRKAR2B,MYLK,VCP,RAC1,PLEK,CHORDC1,GFM2,C1orf87,GBE1,FABP5,NPM1,PGM2,RTN4,UBE2O |
| Ramos | 63 | 1546 | 17.40331492 | 2.062632157 | 4.34E-08 | 1.72E-05 | 6.13E-07 | 1.42E-07 | CCNB1,PROSC,CALCOCO2,ERAL1,COX4I1,HPCAL1,ELOVL1,CORO1A,ADRBK1,RHOA,HLA-DOB,DOK1,DHX9,GNB2L1,FAM58A,SFN,ANP32E,NME1,CAMK4,TEX264,DCK,ARPC3,CCNH,AAK1,CCT4,MAPK3,YWHAB,DBNL,TCOF1,MCM5,DIABLO,CKAP2,DERL1,RPL22,SERBP1,TK1,GTF2I,PYCR1,RPS13,COASY,MAPK1,SRI,TARDBP,LEPROTL1,SAR1B,SNX1,PAK4,SLC25A10,S100A7A,PAF1,SET,COG3,HSPBP1,NUDT18,PHKG2,ZRANB2,FABP5,NPM1,CIAO1,GOLT1B,PDXK,ACVR2B,UBE2O |
| A549 | 46 | 965 | 12.70718232 | 2.428966077 | 4.48E-08 | 1.77E-05 | 6.11E-07 | 1.42E-07 | DRG1,HSPA2,CARHSP1,GSTP1,RPS6KA2,FUBP1,RPS7,QDPR,CHD4,PSMD14,DHX9,GNB2L1,EPS8L1,SFN,NME1,RPS15,HN1,ARPC3,APEX1,CCT4,MAPK3,ARF5,YWHAB,RPL14,PRH2,SERBP1,SPATA7,GTF2I,ANXA6,CLP1,ANXA11,MAPK1,TXNRD1,POLE3,ALDH1A1,RPS6,VCP,COG3,ZRANB2,GBE1,FABP5,NPM1,CIAO1,RTN4,PDXK,ALDH9A1 |
| Ovary | 221 | 8506 | 61.04972376 | 1.29766239 | 5.6E-08 | 2.22E-05 | 7.4E-07 | 1.72E-07 | CRYM,KCNMB3,EFCAB2,STAT6,ZHX3,DRG1,PROSC,DNAJB5,SRPX2,AFP,STK33,FHL2,NFE2,RAB3B,STAU2,MRPS7,DAZAP2,CALCOCO2,FST,NRAS,RPL10,CSRP1,ERAL1,APOE,HLA-DRB5,COX4I1,HPCAL1,CD47,HSPA2,GULP1,GALT,SP110,ELOVL1,LYPD1,CARHSP1,NAPSA,FUBP3,GSTP1,RPS6KA2,CYBB,CORO1A,FUBP1,RPS7,TIMP1,OLR1,PPP2R4,SLC39A9,ADRBK1,QDPR,RHOA,LAYN,DLX5,RAP1GDS1,ATG3,HPX,SGK2,NECAP2,SPAG16,CHD4,ST6GALNAC6,MRPS25,PSMD14,IMPDH2,XPR1,DHX9,GNB2L1,MRTO4,BCAS2,CYB5R1,FGF7,CDC34,CLDN4,RAB39B,LCAT,VSNL1,CTNNAL1,SFN,ANP32E,NHEJ1,NME1,STARD10,ADD1,CAMK4,RPS15,ANAPC11,DDX58,ARPC3,SPP1,CLGN,SULT1E1,HLA-DRB3,EIF4EBP3,RBKS,AAK1,APEX1,CCT4,FNIP1,CCR10,ABLIM1,BAG5,MAPK3,ARF5,YWHAB,DBNL,TCOF1,GGT6,RPL14,C1QC,C1orf21,RNF32,USP15,MORC4,UGDH,ASF1A,MIPOL1,PTPN11,PRKRA,SMYD3,C14orf119,DIABLO,PDLIM3,RBBP7,PI16,CKAP2,NAGK,CPNE4,MEIS2,DERL1,RPL22,SRPK2,HSPB8,HBG2,SERBP1,TK1,MARCKSL1,CYP2C8,PRPSAP2,RAB8B,MATK,C9,GTF2I,BCL7C,PYCR1,NDUFA8,TOM1,FBXL3,PCGF3,PLCB2,PAK6,ANXA6,RPS13,USP14,CLP1,COASY,RECQL5,ANXA11,RNF11,ABLIM3,MAPK1,TXNRD1,SRI,MLX,TARDBP,F8,EDNRA,PSMD10,LRFN1,LEPROTL1,ALDH1A1,EPB41L3,SAR1B,SELENBP1,MMP7,ABCF3,RAB11A,RUVBL1,TPD52L2,SNX1,PAK4,STK25,SLC25A10,FGA,DDC,FMN1,PECAM1,HSPBAP1,SNX9,ZNF655,SET,PPP3R1,PRKAR2B,RPS6,MYLK,VCP,RAC1,COG3,HSPBP1,AKT3,SRP54,HIATL1,LHX1,GBE1,FABP5,SSPN,ZMYM3,NPM1,CORO2B,CIAO1,ARHGAP29,GOLT1B,KLHL14,CYGB,RTN4,PDXK,RNPEP,ANXA13,NFKBIA,ALDH9A1,ACVR2B,C8orf37,PEX19 |
| 17_wcx | 58 | 1408 | 16.02209945 | 2.08837966 | 1.14E-07 | 4.51E-05 | 1.46E-06 | 3.38E-07 | STAU2,CSRP1,APOE,HLA-DRB5,FUBP3,CORO1A,FUBP1,STATH,HPX,CHD4,IMPDH2,PANK1,GNB2L1,LCAT,ANP32E,NME1,ADD1,TEX264,DCK,HLA-DRB3,NOSIP,EIF4EBP3,APEX1,CCT4,DBNL,C1QC,ENOPH1,MCM5,RBBP7,RPL22,SERBP1,MARCKSL1,C9,GTF2I,PYCR1,NDUFA8,ANXA6,USP14,ANXA11,MAPK1,SRI,TARDBP,F8,PSMD10,RUVBL1,SLC25A10,FGA,RPS6,VCP,COG3,PLEK,CHORDC1,ZRANB2,GBE1,NPM1,PGM2,ALDH9A1,UBE2O |
| Parathyroid | 39 | 776 | 10.77348066 | 2.572256644 | 1.35E-07 | 5.34E-05 | 1.67E-06 | 3.88E-07 | STAT6,DRG1,NFE2,COX4I1,HSPA2,TM4SF1,RHOA,IMPDH2,GNB2L1,CLDN4,RAB39B,RRAGB,NME1,CAMK4,SPP1,PTS,APEX1,MAPK3,C1QC,MORC4,MIPOL1,C14orf119,MEIS2,GTF2I,TOM1,ANXA6,USP14,ANXA11,TXNRD1,TARDBP,PSMD10,FGFR2,PPP3R1,RPS6,RAC1,HSPBP1,LHX1,ZMYM3,NFKBIA |
| Leukocytes | 94 | 2827 | 25.96685083 | 1.672847425 | 2.52E-07 | 9.98E-05 | 3.03E-06 | 7.02E-07 | KCNMB3,CD84,DRG1,PROSC,STK33,NFE2,DAZAP2,CSRP1,SP110,GSTP1,CYBB,CORO1A,FUBP1,OLR1,PPP2R4,RHOA,RAP1GDS1,ATG3,DOK1,IMPDH2,GH2,DHX9,GNB2L1,BCAS2,RAB39B,ANP32E,NME1,ADD1,HN1,ARPC3,SPP1,RAB24,EIF4EBP3,APEX1,CCT4,ABLIM1,CDCP1,YWHAB,DBNL,C1orf21,USP15,UGDH,ASF1A,PTPN11,DIABLO,CKAP2,NAGK,CPNE4,SERBP1,MARCKSL1,PRPSAP2,GTF2I,NDUFA8,FBXL3,ANXA6,RPS13,USP14,CLP1,COASY,RECQL5,ANXA11,MAPK1,TXNRD1,CCL5,SRI,MLX,TARDBP,PSMD10,LEPROTL1,SAR1B,TPD52L2,SNX1,FGA,HSPBAP1,SNX9,FBP1,LST1,SET,RPS6,VCP,COG3,PLEK,AKT3,GFM2,GBE1,SSPN,PGM2,RTN4,PDXK,RNPEP,DGUOK,ALDH9A1,UBE2O,PEX19 |
| Brain | 192 | 7215 | 53.03867403 | 1.330200405 | 2.74E-07 | 0.000108 | 3.19E-06 | 7.3E-07 | CRYM,KCNMB3,ZHX3,DRG1,PROSC,DNAJB5,FHL2,RAB3B,STAU2,CALCOCO2,NRAS,RPL10,CSRP1,ERAL1,BHMT2,APOE,COX4I1,HPCAL1,CD47,HSPA2,GULP1,SP110,CARHSP1,GSTP1,RPS6KA2,CORO1A,CAPRIN2,RPS7,OLR1,PPP2R4,ADRBK1,QDPR,IFIT3,RHOA,LAYN,RAP1GDS1,ATG3,HPX,SGK2,SPAG16,MRPS25,PSMD14,SLC5A6,IMPDH2,PANK1,XPR1,DHX9,GNB2L1,CDO1,BCAS2,CYB5R1,CLDN4,SYNPR,RAB39B,LCAT,VSNL1,PTN,CTNNAL1,SFN,ANP32E,FN3K,SEPT1,NHEJ1,NME1,ADD1,ACTL6B,CAMK4,RPS15,DCK,HN1,DDX58,ARPC3,SPP1,RAB24,SULT1E1,KLF11,NOSIP,EIF4EBP3,PTS,AAK1,ARHGDIG,APEX1,CCT4,FNIP1,ABLIM1,BAG5,MAPK3,TIRAP,ARF5,YWHAB,DBNL,TCOF1,RPL14,CALN1,TTC1,USP15,UGDH,ASF1A,MIPOL1,ENOPH1,PTPN11,PRKRA,MCM5,DIABLO,RBBP7,CKAP2,GABRA5,NAGK,CPNE4,MEIS2,MCTS1,RPL22,SRPK2,HSPB8,HBG2,SERBP1,MARCKSL1,CYP2C8,STUB1,PRPSAP2,RAB8B,MATK,GTF2I,NDUFA8,DAPP1,TOM1,FBXL3,PAK6,ANXA6,RPS13,USP14,TEAD3,COASY,RECQL5,ANXA11,PDS5B,RNF11,ABLIM3,MAPK1,TXNRD1,POLE3,SRI,MLX,TARDBP,HCRTR1,MOXD1,CKM,PSMD10,LEPROTL1,ALDH1A1,EPB41L3,SAR1B,SELENBP1,RAB11A,RUVBL1,TPD52L2,SNX1,FGFR2,SLC25A10,FGA,SNX9,SET,PPP3R1,PRKAR2B,RPS6,MYLK,VCP,RAC1,COG3,MBIP,HSPBP1,AKT3,SRP54,GFM2,LHX1,ZRANB2,GBE1,FABP5,SSPN,ZMYM3,NPM1,CORO2B,ARHGAP29,RTN4,PDXK,RNPEP,FAM9C,DGUOK,ALDH9A1,ACVR2B,UBE2O,PEX19 |
| 050603_BALF11 | 45 | 994 | 12.43093923 | 2.308281094 | 2.78E-07 | 0.00011 | 3.14E-06 | 7.3E-07 | MRPS7,NRAS,HLA-DRB5,COX4I1,HPCAL1,GSTP1,CORO1A,RPS7,TIMP1,HPX,MRPS25,IMPDH2,XPR1,GNB2L1,CYB5R1,RAB39B,NME1,TEX264,ARPC3,SPP1,HLA-DRB3,APEX1,CCT4,YWHAB,RBBP7,PRH2,SERBP1,MARCKSL1,RAB8B,PYCR1,NDUFA8,ANXA6,RPS13,ANXA11,POLE3,SLC25A10,FGA,FBP1,SET,RPS6,VCP,NPM1,CIAO1,RTN4,PEX19 |
| H358 | 58 | 1448 | 16.02209945 | 2.030739373 | 2.92E-07 | 0.000116 | 3.22E-06 | 7.46E-07 | SRPX2,AFP,FHL2,APOE,COX4I1,HPCAL1,GALT,CARHSP1,CKS2,GSTP1,FUBP1,RPS7,TIMP1,PPP2R4,RHOA,CHD4,IMPDH2,DHX9,GNB2L1,MRTO4,BCAS2,SFN,NME1,RPS15,HN1,ARPC3,SPP1,APEX1,CCT4,YWHAB,DBNL,TCOF1,USP15,UGDH,ENOPH1,RBBP7,SERBP1,MARCKSL1,PDS5B,TXNRD1,SRI,TARDBP,PSMD10,MMP7,RAB11A,RUVBL1,TPD52L2,SNX9,SET,VCP,GBE1,FABP5,NPM1,PGM2,RTN4,RNPEP,UBE2O,PEX19 |
| Sy5y | 84 | 2447 | 23.20441989 | 1.729608329 | 3.65E-07 | 0.000144 | 3.9E-06 | 9.06E-07 | DRG1,PROSC,MRPS7,NRAS,RPL10,CSRP1,APOE,COX4I1,GSTP1,CORO1A,FUBP1,RPS7,RHOA,HPX,CHD4,PSMD14,IMPDH2,GNB2L1,BCAS2,VSNL1,SFN,NHEJ1,NME1,ADD1,DHFR,ARPC3,NOSIP,APEX1,CCT4,MRPL53,ABLIM1,MAPK3,ARF5,YWHAB,DBNL,TCOF1,RPL14,TTC1,UGDH,PTPN11,ZFYVE19,MCM5,RBBP7,CKAP2,PRH2,MCTS1,HIGD2A,RPL22,SRPK2,C14orf80,SERBP1,TK1,STUB1,PRPSAP2,GTF2I,BCL7C,PYCR1,NDUFA8,TOM1,CHRAC1,ANXA6,RPS13,COASY,ANXA11,MAPK1,TARDBP,ALDH1A1,RAB11A,RUVBL1,TPD52L2,SNX1,PAK4,FGA,SET,RPS6,VCP,RAC1,HSPBP1,SRP54,GKAP1,CHORDC1,FABP5,NPM1,RTN4 |
| Skeletal muscles | 227 | 8978 | 62.70718232 | 1.262640939 | 3.76E-07 | 0.000149 | 3.92E-06 | 9.1E-07 | CRYM,KCNMB3,HOXA5,EFCAB2,ZHX3,COQ6,DRG1,PROSC,DNAJB5,SRPX2,AFP,STK33,FHL2,NFE2,STAU2,MRPS7,CALCOCO2,FST,NRAS,TYROBP,RPL10,CSRP1,ERAL1,BHMT2,APOE,COX4I1,CD47,UPP2,HSPA2,GULP1,TM4SF1,GALT,SP110,ELOVL1,LYPD1,LRRC20,CARHSP1,GSTP1,RPS6KA2,CYBB,CORO1A,FUBP1,ATAD2,FAM63A,CAPRIN2,RPS7,PPP2R4,SLC39A9,QDPR,IFIT3,RHOA,DLX5,RAP1GDS1,ATG3,HPX,SGK2,SPAG16,CHD4,ST6GALNAC6,DOK1,MRPS25,PSMD14,SLC5A6,IMPDH2,PANK1,XPR1,DHX9,GNB2L1,BCAS2,CYB5R1,CDC34,CLDN4,RAB39B,RRAGB,CTNNAL1,ANP32E,FN3K,SEPT1,NHEJ1,NME1,STARD10,ADD1,CAMK4,TEX264,ZNF641,TMEM185A,HN1,ANAPC11,DDX58,ARPC3,SPP1,CLGN,RAB24,SULT1E1,KLF11,NOSIP,EIF4EBP3,AAK1,APEX1,CCT4,FNIP1,CCR10,ABLIM1,BAG5,MAPK3,TIRAP,CDCP1,MYOT,ARF5,YWHAB,DBNL,TCOF1,GGT6,RPL14,C1QC,C1orf21,USP15,UGDH,ASF1A,MIPOL1,ZFYVE19,PRKRA,SMYD3,C14orf119,DIABLO,PDLIM3,CKAP2,NAGK,CPNE4,MEIS2,MCTS1,DERL1,SRPK2,HSPB8,HBG2,SERBP1,STUB1,PRPSAP2,RAB8B,MATK,C9,GTF2I,BCL7C,NDUFA8,DAPP1,TOM1,FBXL3,PCGF3,PLCB2,PAK6,ANXA6,RPS13,USP14,CLP1,TEAD3,COASY,RECQL5,ANXA11,RNF11,ABLIM3,MAPK1,TXNRD1,SRI,MLX,TARDBP,RASGEF1A,MOXD1,CKM,F8,EDNRA,SLA,CETN3,PSMD10,LRFN1,LEPROTL1,ALDH1A1,SAR1B,SELENBP1,MMP7,ABCF3,RAB11A,TPD52L2,SNX1,PAK4,DUPD1,FGFR2,SLC25A10,FGA,FMN1,PECAM1,TAF7,HSPBAP1,SNX9,ZNF655,SET,PPP3R1,RPS6,MYLK,VCP,RAC1,COG3,MBIP,HSPBP1,AKT3,NUDT18,HIATL1,GFM2,LHX1,ZRANB2,GBE1,FABP5,SSPN,ZMYM3,NPM1,CIAO1,ARHGAP29,GOLT1B,KLHL14,CYGB,RTN4,PDXK,RNPEP,ANXA13,NFKBIA,ALDH9A1,C8orf37,UBE2O |
| Pancreatic cancer | 212 | 8226 | 58.56353591 | 1.287488887 | 3.86E-07 | 0.000153 | 3.92E-06 | 9.1E-07 | CRYM,KCNMB3,HOXA5,EFCAB2,STAT6,ZHX3,CCNB1,ADRB2,DRG1,PROSC,DNAJB5,AFP,STK33,FHL2,NFE2,RAB3B,STAU2,MRPS7,CALCOCO2,FST,NRAS,TYROBP,RPL10,CSRP1,ERAL1,APOE,HLA-DRB5,COX4I1,CD47,UPP2,HSPA2,GULP1,TM4SF1,GALT,ELOVL1,LYPD1,CARHSP1,GSTP1,RPS6KA2,CORO1A,FUBP1,ATAD2,FAM63A,RPS7,TIMP1,PPP2R4,SLC39A9,QDPR,RHOA,DLX5,RAP1GDS1,HPX,NECAP2,CHD4,ST6GALNAC6,DOK1,PSMD14,IMPDH2,PANK1,XPR1,DHX9,GNB2L1,MRTO4,BCAS2,CYB5R1,CDC34,CLDN4,RAB39B,EPS8L1,RRAGB,SFN,ANP32E,NHEJ1,NME1,STARD10,ADD1,CAMK4,TEX264,ANAPC11,DDX58,ARPC3,SPP1,CLGN,SULT1E1,HLA-DRB3,PTS,RBKS,CCNH,AAK1,APEX1,CCT4,CCR10,BAG5,MAPK3,CDCP1,ARF5,YWHAB,DBNL,TCOF1,GGT6,RPL14,C1QC,C1orf21,USP15,MORC4,UGDH,MIPOL1,ENOPH1,PTPN11,PRKRA,SMYD3,MCM5,C14orf119,DIABLO,PDLIM3,CKAP2,NAGK,CPNE4,MEIS2,MCTS1,DERL1,RPL22,SRPK2,HSPB8,SERBP1,TK1,MARCKSL1,CYP2C8,PRPSAP2,MATK,C9,GTF2I,BCL7C,CDH26,NDUFA8,TOM1,PCGF3,PLCB2,PAK6,ANXA6,RPS13,USP14,TEAD3,COASY,RECQL5,ANXA11,PDS5B,ABLIM3,MAPK1,TXNRD1,SRI,MLX,TARDBP,RASGEF1A,MOXD1,F8,EDNRA,SLA,CETN3,PSMD10,ALDH1A1,EPB41L3,SAR1B,SELENBP1,MMP7,ABCF3,RAB11A,RUVBL1,TPD52L2,SNX1,PAK4,FGFR2,SLC25A10,FGA,DDC,FMN1,PECAM1,TAF7,SNX9,FBP1,ZNF655,SET,PPP3R1,PRKAR2B,RPS6,MYLK,VCP,RAC1,MBIP,HSPBP1,AKT3,NUDT18,HIATL1,C1orf87,LHX1,GBE1,ZMYM3,NPM1,CORO2B,CIAO1,PGM2,ARHGAP29,KLHL14,CYGB,RTN4,PDXK,RNPEP,ANXA13,NFKBIA,ALDH9A1,ACVR2B,C8orf37 |
| Endometrium | 50 | 1192 | 13.8121547 | 2.133396461 | 5.68E-07 | 0.000225 | 5.63E-06 | 1.31E-06 | STAT6,CCNB1,DRG1,NFE2,KLK1,COX4I1,HSPA2,TM4SF1,TIMP1,RHOA,IMPDH2,GNB2L1,CLDN4,RAB39B,RRAGB,SFN,NME1,SPP1,SULT1E1,PTS,APEX1,MAPK3,YWHAB,C1QC,MORC4,MIPOL1,MCM5,C14orf119,DIABLO,MEIS2,GTF2I,TOM1,ANXA6,USP14,ANXA11,MAPK1,TXNRD1,SRI,TARDBP,PSMD10,PECAM1,PPP3R1,RPS6,RAC1,MBIP,HSPBP1,LHX1,ZMYM3,NPM1,NFKBIA |
| Nuclear | 53 | 1305 | 14.64088398 | 2.062920932 | 6.75E-07 | 0.000267 | 6.52E-06 | 1.51E-06 | KRR1,CSRP1,OGFOD2,GULP1,FUBP3,FUBP1,PPP2R4,ADRBK1,CHD4,IMPDH2,GNB2L1,BCAS2,ANP32E,NME1,ADD1,APEX1,CCT4,MAPK3,DBNL,USP15,UGDH,PRKRA,RBBP7,MCTS1,SRPK2,SERBP1,MARCKSL1,STUB1,GTF2I,PYCR1,ANXA6,USP14,PDS5B,MAPK1,TXNRD1,TARDBP,PSMD10,SAR1B,RUVBL1,PAK4,PPP3R1,RPS6,VCP,COG3,HSPBP1,CHORDC1,GBE1,NPM1,PGM2,RTN4,RNPEP,ALDH9A1,PEX19 |
| HeLa | 158 | 5699 | 43.64640884 | 1.387697102 | 7.72E-07 | 0.000306 | 7.27E-06 | 1.69E-06 | DOHH,COQ6,CCNB1,PROSC,RAB3B,STAU2,MRPS7,CALCOCO2,NRAS,TYROBP,RPL10,KRR1,CSRP1,COX4I1,HPCAL1,CD47,HSPA2,SP110,ELOVL1,CARHSP1,FUBP3,GSTP1,FUBP1,ATAD2,CAPRIN2,FBXO31,RPS7,PPP2R4,CD96,RAP1GDS1,ATG3,TIPIN,CHD4,MRPS25,PSMD14,IMPDH2,GH2,PANK1,DHX9,GNB2L1,CDO1,BCAS2,FAM58A,RAB39B,CTNNAL1,SFN,ANP32E,FN3K,NME1,STARD10,NMRAL1,CAMK4,RPS15,TEX264,DCK,DHFR,FBXO3,HN1,ANAPC11,ARPC3,MTL5,NOSIP,EIF4EBP3,CCNH,AAK1,APEX1,ZNF761,CCT4,MRPL53,MAPK3,ARF5,YWHAB,DBNL,TCOF1,RPL14,TTC1,USP15,MORC4,UGDH,ASF1A,MIPOL1,ZFYVE19,PRKRA,SMYD3,MCM5,DIABLO,QTRT1,RBBP7,CKAP2,NAGK,RPL22,SRPK2,FAM81A,HBG2,SERBP1,TK1,MARCKSL1,SPC25,STUB1,RAB8B,GTF2I,BCL7C,PYCR1,PLCB2,CHRAC1,ANXA6,RPS13,USP14,COASY,TMEM106A,RECQL5,ANXA11,PDS5B,MAPK1,TXNRD1,POLE3,SRI,TARDBP,CKM,CETN3,PSMD10,LEPROTL1,SAR1B,SELENBP1,ABCF3,CCDC102B,RAB11A,RUVBL1,TPD52L2,SNX1,PAK4,FGFR2,SLC25A10,S100A7A,DDC,TAF7,ZNF655,SET,RPS6,ATP6V1C2,VCP,RAC1,HSPBP1,PHKG2,GFM2,ZRANB2,GBE1,FABP5,ZMYM3,NPM1,CIAO1,ARHGAP29,PDXK,RNPEP,ANXA13,ALDH9A1,ACVR2B,UBE2O |
| Thyroid | 40 | 882 | 11.04972376 | 2.319794158 | 1.3E-06 | 0.000513 | 1.19E-05 | 2.77E-06 | STAT6,DRG1,NFE2,COX4I1,HSPA2,TM4SF1,RHOA,IMPDH2,GNB2L1,CLDN4,RAB39B,RRAGB,NME1,SPP1,PTS,APEX1,MAPK3,YWHAB,C1QC,MORC4,MIPOL1,C14orf119,DIABLO,MEIS2,GTF2I,TOM1,ANXA6,USP14,ANXA11,MAPK1,TXNRD1,TARDBP,PSMD10,PPP3R1,RPS6,MBIP,HSPBP1,LHX1,ZMYM3,NFKBIA |
| Colorectal cancer cells | 29 | 542 | 8.011049724 | 2.765889258 | 1.74E-06 | 0.00069 | 1.57E-05 | 3.64E-06 | NRAS,CSRP1,COX4I1,HPCAL1,HSPA2,GSTP1,RPS7,RHOA,GNB2L1,CLDN4,SFN,NME1,ARPC3,CCT4,ARF5,YWHAB,UGDH,MARCKSL1,RAB8B,ANXA6,RPS13,ANXA11,ALDH1A1,MMP7,RAB11A,RUVBL1,VCP,RAC1,PDXK |
| Placenta | 258 | 10796 | 71.27071823 | 1.192654776 | 3.17E-06 | 0.001254 | 2.79E-05 | 6.47E-06 | KCNMB3,HOXA5,EFCAB2,STAT6,ZHX3,COQ6,CCNB1,ADRB2,DRG1,PROSC,DNAJB5,SRPX2,AFP,STK33,FHL2,NFE2,RAB3B,STAU2,MRPS7,CALCOCO2,FST,NRAS,TYROBP,RPL10,CSRP1,ERAL1,APOE,HLA-DRB5,COX4I1,HPCAL1,CD47,UPP2,HSPA2,GULP1,TM4SF1,GALT,SP110,ELOVL1,EDN3,LYPD1,CARHSP1,FUBP3,GSTP1,RPS6KA2,CYBB,CORO1A,FUBP1,ATAD2,FAM63A,CAPRIN2,RPS7,STATH,OLR1,PPP2R4,SLC39A9,QDPR,IFIT3,RHOA,DLX5,RAP1GDS1,ATG3,HPX,SGK2,NECAP2,CHD4,ST6GALNAC6,DOK1,MRPS25,PSMD14,SLC5A6,IMPDH2,GH2,PANK1,XPR1,DHX9,GNB2L1,MRTO4,BCAS2,CYB5R1,CDC34,CLDN4,RAB39B,EPS8L1,RRAGB,CTNNAL1,SFN,ANP32E,FN3K,NHEJ1,NME1,STARD10,ADD1,CAMK4,TEX264,DCK,ZNF641,TMEM185A,HN1,ANAPC11,DDX58,ARPC3,SPP1,CLGN,SULT1E1,KLF11,EIF4EBP3,PTS,RBKS,AAK1,APEX1,CCT4,CCR10,ABLIM1,BAG5,MAPK3,TIRAP,CDCP1,ARF5,YWHAB,DBNL,TCOF1,GGT6,RPL14,C1QC,C1orf21,TTC1,USP15,MORC4,UGDH,ASF1A,MIPOL1,ENOPH1,PTPN11,ZFYVE19,PRKRA,SMYD3,MCM5,C14orf119,DIABLO,PDLIM3,RBBP7,CKAP2,NAGK,PRH2,CPNE4,MEIS2,MCTS1,DERL1,RPL22,SRPK2,HSPB8,HBG2,SERBP1,TK1,MARCKSL1,STUB1,PRPSAP2,RAB8B,MATK,C9,GTF2I,BCL7C,PYCR1,CDH26,NDUFA8,DAPP1,TOM1,FBXL3,PCGF3,PLCB2,PAK6,ANXA6,RPS13,USP14,CLP1,TEAD3,COASY,TMEM106A,RECQL5,ANXA11,PDS5B,RNF11,ABLIM3,MAPK1,TXNRD1,POLE3,SRI,MLX,TARDBP,RASGEF1A,F8,EDNRA,SLA,CETN3,PSMD10,LEPROTL1,ALDH1A1,EPB41L3,SAR1B,SELENBP1,ABCF3,RAB11A,RUVBL1,TPD52L2,SNX1,PAK4,FGFR2,SLC25A10,FGA,DDC,FMN1,PECAM1,TAF7,HSPBAP1,SNX9,ZNF655,SET,PPP3R1,PRKAR2B,RPS6,ATP6V1C2,MYLK,VCP,RAC1,COG3,MBIP,HSPBP1,PLEK,AKT3,SRP54,NUDT18,CHORDC1,GFM2,LHX1,ZRANB2,GBE1,FABP5,SSPN,ZMYM3,NPM1,CORO2B,CIAO1,PGM2,ARHGAP29,GOLT1B,KLHL14,CYGB,RTN4,PDXK,RNPEP,ANXA13,NFKBIA,DGUOK,ALDH9A1,ACVR2B,C8orf37,UBE2O,PEX19 |
| Serum | 194 | 7543 | 53.59116022 | 1.28553562 | 3.35E-06 | 0.001329 | 2.89E-05 | 6.7E-06 | DOHH,STAT6,CD84,CCNB1,ADRB2,DRG1,PROSC,DNAJB5,OIP5,NFE2,RAB3B,STAU2,MRPS7,KLK1,KRR1,CSRP1,ERAL1,OGFOD2,APOE,COX4I1,HSPA2,ELOVL1,LRRC20,CARHSP1,IGLL1,GSTP1,CAMK2N1,RPS6KA2,CORO1A,FUBP1,ATAD2,CAPRIN2,FBXO31,RPS7,TIMP1,STATH,PPP2R4,ADRBK1,QDPR,RAP1GDS1,ATG3,HPX,SGK2,NECAP2,CHD4,ST6GALNAC6,DOK1,MRPS25,PSMD14,SLC5A6,IMPDH2,GLYAT,MRTO4,BCAS2,CDC34,LCAT,RRAGB,CTNNAL1,FBXL18,SFN,ANP32E,SEPT1,NME1,STARD10,NMRAL1,ADD1,CAMK4,RPS15,DCK,FBXO3,TMEM185A,HN1,ARPC3,SPP1,RAB24,SULT1E1,NOSIP,EIF4EBP3,CCNH,AAK1,APEX1,CCT4,SERTAD3,CXXC5,ZNF587,ARIH2,CDCP1,YWHAB,DBNL,TCOF1,C1QC,TTC1,USP15,MORC4,ASF1A,ENOPH1,PTPN11,SMYD3,MCM5,C14orf119,DIABLO,PDLIM3,RBBP7,PI16,CKAP2,NAGK,PRH2,MEIS2,MCTS1,DERL1,RPL22,SRPK2,FAM81A,ATP6V1E2,HBG2,SERBP1,TK1,MARCKSL1,SPC25,CYP2C8,STUB1,PRPSAP2,MATK,C9,GTF2I,PYCR1,NDUFA8,DAPP1,TOM1,ANXA6,RPS13,USP14,CLP1,COASY,TMEM106A,PDS5B,ABLIM3,POLE3,MLX,MOXD1,CKM,F8,EDNRA,CETN3,PSMD10,ALDH1A1,EPB41L3,SELENBP1,ABCF3,CCDC102B,RUVBL1,TPD52L2,SNX1,PAK4,STK25,FGFR2,FGA,FMN1,PAF1,HSPBAP1,SNX9,ZNF655,SET,PRKAR2B,RPS6,MYLK,VCP,RAC1,MBIP,HSPBP1,AKT3,SRP54,GKAP1,CHORDC1,PHKG2,HIATL1,GFM2,C1orf87,LHX1,ZRANB2,EYA1,ZMYM3,NPM1,CIAO1,PGM2,ARHGAP29,RTN4,PDXK,RNPEP,ANXA13,DGUOK,ALDH9A1,C8orf37,PEX19 |
| Cortex | 32 | 662 | 8.839779006 | 2.490138681 | 4.34E-06 | 0.001719 | 3.66E-05 | 8.49E-06 | CRYM,CSRP1,APOE,COX4I1,HPCAL1,CD47,HSPA2,GSTP1,QDPR,RAP1GDS1,VSNL1,NME1,ADD1,ARPC3,AAK1,CCT4,MAPK3,YWHAB,DBNL,NDUFA8,TOM1,ANXA6,MAPK1,SRI,EPB41L3,RAB11A,PRKAR2B,RAC1,NPM1,RTN4,PDXK,ALDH9A1 |
| Pancreas | 239 | 9835 | 66.02209945 | 1.213229556 | 4.56E-06 | 0.001806 | 3.76E-05 | 8.74E-06 | CRYM,KCNMB3,HOXA5,EFCAB2,ZHX3,ADRB2,DRG1,PROSC,DNAJB5,AFP,STK33,FHL2,NFE2,RAB3B,STAU2,MRPS7,CALCOCO2,FST,NRAS,TYROBP,RPL10,KLK1,CSRP1,ERAL1,APOE,HLA-DRB5,COX4I1,CD47,UPP2,HSPA2,GULP1,TM4SF1,GALT,SP110,ELOVL1,LYPD1,GSTP1,RPS6KA2,FUBP1,ATAD2,FAM63A,CAPRIN2,RPS7,TIMP1,OLR1,PPP2R4,SLC39A9,QDPR,IFIT3,RHOA,DLX5,RAP1GDS1,HPX,SGK2,SPAG16,CHD4,ST6GALNAC6,DOK1,MRPS25,PSMD14,SLC5A6,IMPDH2,PANK1,XPR1,DHX9,GNB2L1,MRTO4,BCAS2,CYB5R1,FGF7,CDC34,CLDN4,RAB39B,PTN,RRAGB,CTNNAL1,SFN,ANP32E,FN3K,NHEJ1,NME1,STARD10,ADD1,CAMK4,TEX264,ZNF641,ANAPC11,DDX58,ARPC3,SPP1,CLGN,KLF11,HLA-DRB3,EIF4EBP3,PTS,RBKS,CCNH,AAK1,ARHGDIG,APEX1,CCT4,FNIP1,CCR10,ABLIM1,BAG5,MAPK3,CDCP1,ARF5,YWHAB,DBNL,TCOF1,GGT6,RPL14,C1QC,C1orf21,USP15,MORC4,UGDH,ASF1A,MIPOL1,PTPN11,PRKRA,SMYD3,MCM5,DIABLO,PDLIM3,PI16,CKAP2,NAGK,CPNE4,MEIS2,MCTS1,DERL1,RPL22,SRPK2,HSPB8,HBG2,SERBP1,MARCKSL1,CYP2C8,STUB1,PRPSAP2,RAB8B,MATK,C9,GTF2I,BCL7C,PYCR1,CDH26,NDUFA8,DAPP1,TOM1,FBXL3,PCGF3,PLCB2,PAK6,ANXA6,RPS13,USP14,CLP1,TEAD3,COASY,RECQL5,ANXA11,PDS5B,RNF11,ABLIM3,MAPK1,TXNRD1,POLE3,SRI,MLX,TARDBP,RASGEF1A,MOXD1,F8,EDNRA,SLA,CETN3,PSMD10,LRFN1,LEPROTL1,ALDH1A1,EPB41L3,SAR1B,SELENBP1,MMP7,ABCF3,RAB11A,RUVBL1,TPD52L2,SNX1,PAK4,FGFR2,SLC25A10,FGA,DDC,FMN1,TAF7,HSPBAP1,SNX9,FBP1,ZNF655,SET,PPP3R1,PRKAR2B,RPS6,MYLK,VCP,RAC1,COG3,MBIP,HSPBP1,AKT3,SRP54,NUDT18,HIATL1,GFM2,C1orf87,LHX1,ZRANB2,FABP5,SSPN,ZMYM3,NPM1,CORO2B,CIAO1,PGM2,ARHGAP29,KLHL14,CYGB,RTN4,PDXK,RNPEP,ANXA13,NFKBIA,ALDH9A1,ACVR2B,C8orf37 |
| Vulva | 37 | 832 | 10.22099448 | 2.280159675 | 5.21E-06 | 0.002061 | 4.21E-05 | 9.76E-06 | STAT6,CCNB1,DRG1,NFE2,COX4I1,HSPA2,IMPDH2,GNB2L1,CLDN4,RRAGB,NME1,SPP1,PTS,APEX1,MAPK3,YWHAB,MORC4,MCM5,C14orf119,DIABLO,MEIS2,GTF2I,TOM1,ANXA6,USP14,ANXA11,MAPK1,TXNRD1,TARDBP,F8,PSMD10,PPP3R1,RPS6,HSPBP1,LHX1,ZMYM3,NFKBIA |
| Oesophagus | 39 | 914 | 10.77348066 | 2.184415707 | 7.54E-06 | 0.002985 | 5.97E-05 | 1.39E-05 | KCNMB3,STAT6,CCNB1,DRG1,STK33,NFE2,COX4I1,HSPA2,TM4SF1,GSTP1,IMPDH2,GNB2L1,CLDN4,NME1,SPP1,APEX1,MAPK3,YWHAB,MORC4,MIPOL1,MCM5,C14orf119,DIABLO,MEIS2,GTF2I,TOM1,PAK6,USP14,MAPK1,TXNRD1,TARDBP,F8,MMP7,PPP3R1,RPS6,MBIP,HSPBP1,LHX1,ZMYM3 |
| HTB75 | 48 | 1241 | 13.25966851 | 1.969275025 | 9.07E-06 | 0.003591 | 7.04E-05 | 1.63E-05 | PROSC,FHL2,CSRP1,APOE,HSPA2,CARHSP1,GSTP1,FUBP1,RPS7,TIMP1,OLR1,RHOA,IMPDH2,GNB2L1,SFN,NME1,APEX1,CCT4,MRPL53,YWHAB,DBNL,RPL14,TTC1,UGDH,RBBP7,RPL22,SERBP1,MARCKSL1,ANXA6,USP14,ANXA11,MAPK1,TXNRD1,SRI,TARDBP,RAB11A,RUVBL1,TPD52L2,SNX1,FBP1,SET,RPS6,VCP,FABP5,NPM1,PGM2,RTN4,RNPEP |
| Bone marrow | 196 | 7744 | 54.14364641 | 1.265000552 | 9.42E-06 | 0.003729 | 7.17E-05 | 1.66E-05 | KCNMB3,HOXA5,EFCAB2,DOHH,STAT6,CD84,ZHX3,CCNB1,ADRB2,DRG1,PROSC,DNAJB5,AFP,STK33,FHL2,NFE2,STAU2,MRPS7,CALCOCO2,FST,NRAS,TYROBP,RPL10,CSRP1,ERAL1,APOE,COX4I1,UPP2,HSPA2,GULP1,TM4SF1,GALT,ELOVL1,LYPD1,FUBP3,IGLL1,GSTP1,RPS6KA2,CORO1A,FUBP1,ATAD2,FAM63A,CAPRIN2,TIMP1,OLR1,PPP2R4,SLC39A9,QDPR,RHOA,DLX5,RAP1GDS1,NECAP2,CHD4,ST6GALNAC6,DOK1,PSMD14,IMPDH2,XPR1,DHX9,GNB2L1,BCAS2,CYB5R1,CDC34,CLDN4,RAB39B,PTN,RRAGB,FBXL18,ANP32E,FN3K,NHEJ1,NME1,STARD10,ADD1,CAMK4,RPS15,TEX264,ANAPC11,DDX58,ARPC3,SPP1,SULT1E1,KLF11,HLA-DRB3,PTS,AAK1,APEX1,CCT4,CCR10,MRPL53,BAG5,MAPK3,CDCP1,YWHAB,DBNL,TCOF1,GGT6,RPL14,C1QC,C1orf21,USP15,MORC4,UGDH,PTPN11,PRKRA,SMYD3,MCM5,C14orf119,DIABLO,PDLIM3,CKAP2,NAGK,CPNE4,MEIS2,DERL1,SRPK2,SERBP1,TK1,CYP2C8,PRPSAP2,MATK,C9,GTF2I,PYCR1,NDUFA8,DAPP1,TOM1,PCGF3,PLCB2,ANXA6,USP14,TEAD3,COASY,RECQL5,ANXA11,ABLIM3,MAPK1,TXNRD1,SRI,MLX,TARDBP,F8,EDNRA,SLA,CETN3,PSMD10,ALDH1A1,SAR1B,SELENBP1,ABCF3,RAB11A,RUVBL1,TPD52L2,SNX1,PAK4,FGFR2,SLC25A10,FGA,FMN1,PECAM1,TAF7,SNX9,FBP1,ZNF655,LST1,SET,PPP3R1,RPS6,MYLK,VCP,RAC1,MBIP,HSPBP1,PLEK,AKT3,SRP54,CHORDC1,GFM2,LHX1,ZRANB2,FABP5,ZMYM3,NPM1,CORO2B,CIAO1,ARHGAP29,KLHL14,RTN4,PDXK,RNPEP,ANXA13,NFKBIA,DGUOK,ALDH9A1,ACVR2B,C8orf37 |
| Urine | 97 | 3202 | 26.79558011 | 1.5235295 | 1.02E-05 | 0.004046 | 7.63E-05 | 1.77E-05 | CRYM,CD84,RAB3B,NRAS,RPL10,KLK1,CSRP1,BHMT2,APOE,METTL8,UPP2,HSPA2,GALT,EDN3,LYPD1,CARHSP1,NAPSA,IGLL1,GSTP1,CYBB,CORO1A,ATAD2,RPS7,TIMP1,OLR1,PPP2R4,QDPR,IFIT3,RHOA,LAYN,RAP1GDS1,TIPIN,HPX,IMPDH2,GNB2L1,LCAT,EPS8L1,SFN,NME1,ADD1,CAMK4,DCK,ARPC3,SPP1,ABLIM1,MAPK3,ARF5,YWHAB,DBNL,TCOF1,GGT6,C1QC,UGDH,ENOPH1,SMYD3,PI16,NAGK,PRH2,RPL22,FAM81A,HBG2,PRPSAP2,RAB8B,C9,GTF2I,TOM1,ANXA6,RPS13,COASY,TMEM106A,ANXA11,RNF11,SRI,ALDH1A1,SELENBP1,MMP7,CCDC102B,RAB11A,FGFR2,FGA,DDC,SNX9,FBP1,PRKAR2B,ATP6V1C2,MYLK,VCP,RAC1,GBE1,FABP5,NPM1,PGM2,RTN4,PDXK,RNPEP,ANXA13,ALDH9A1 |
| Uterine cervix | 38 | 893 | 10.49723757 | 2.180167473 | 1.06E-05 | 0.004186 | 7.75E-05 | 1.8E-05 | STAT6,CCNB1,DRG1,NFE2,COX4I1,HSPA2,TM4SF1,RHOA,IMPDH2,GNB2L1,CLDN4,RAB39B,RRAGB,NME1,CAMK4,SPP1,PTS,APEX1,MAPK3,YWHAB,MIPOL1,C14orf119,DIABLO,MEIS2,GTF2I,TOM1,USP14,ANXA11,MAPK1,TXNRD1,TARDBP,PSMD10,PPP3R1,RPS6,MBIP,HSPBP1,LHX1,ZMYM3 |
| CPE_SCX_fractionated | 75 | 2302 | 20.71823204 | 1.64440168 | 1.24E-05 | 0.004917 | 8.94E-05 | 2.08E-05 | DOHH,DRG1,FHL2,CSRP1,ERAL1,HPCAL1,GALT,FUBP3,GSTP1,CORO1A,FUBP1,RPS7,QDPR,RAP1GDS1,ATG3,CHD4,MRPS25,IMPDH2,GNB2L1,CYB5R1,SFN,ANP32E,NME1,NOSIP,EIF4EBP3,AAK1,APEX1,CCT4,MRPL53,BAG5,MAPK3,ARF5,YWHAB,DBNL,USP15,UGDH,ENOPH1,PRKRA,SMYD3,QTRT1,RBBP7,RPL22,SRPK2,SERBP1,RNF126,NDUFA8,RPS13,USP14,ANXA11,MAPK1,TXNRD1,SRI,TARDBP,PSMD10,SAR1B,RUVBL1,PAK4,SLC25A10,SET,PPP3R1,RPS6,VCP,COG3,HSPBP1,CHORDC1,GFM2,GBE1,FABP5,NPM1,CIAO1,PGM2,RTN4,RNPEP,NFKBIA,ALDH9A1 |
| Adrenal glands | 190 | 7492 | 52.48618785 | 1.267769692 | 1.4E-05 | 0.005559 | 9.93E-05 | 2.3E-05 | CRYM,KCNMB3,HOXA5,EFCAB2,STAT6,ZHX3,CCNB1,DRG1,PROSC,DNAJB5,AFP,STK33,FHL2,NFE2,RAB3B,STAU2,MRPS7,CALCOCO2,FST,NRAS,TYROBP,RPL10,ERAL1,APOE,COX4I1,UPP2,HSPA2,GULP1,TM4SF1,GALT,ELOVL1,LYPD1,GSTP1,RPS6KA2,FUBP1,ATAD2,FAM63A,TIMP1,OLR1,PPP2R4,SLC39A9,RHOA,DLX5,RAP1GDS1,HPX,SGK2,NECAP2,SPAG16,CHD4,ST6GALNAC6,DOK1,PSMD14,IMPDH2,PANK1,DHX9,GNB2L1,MRTO4,CYB5R1,CDC34,CLDN4,RAB39B,RRAGB,SFN,ANP32E,NHEJ1,NME1,STARD10,ADD1,CAMK4,TEX264,ZNF641,ANAPC11,DDX58,ARPC3,SPP1,CLGN,SULT1E1,KLF11,PTS,RBKS,CCNH,AAK1,APEX1,CCT4,CCR10,BAG5,MAPK3,CDCP1,YWHAB,DBNL,TCOF1,GGT6,RPL14,C1QC,C1orf21,USP15,MORC4,MIPOL1,PTPN11,PRKRA,SMYD3,MCM5,C14orf119,DIABLO,PDLIM3,CKAP2,NAGK,CPNE4,MEIS2,DERL1,SRPK2,HSPB8,SERBP1,TK1,MARCKSL1,CYP2C8,PRPSAP2,MATK,C9,GTF2I,BCL7C,CDH26,NDUFA8,TOM1,PCGF3,PLCB2,PAK6,ANXA6,USP14,TEAD3,COASY,ANXA11,ABLIM3,MAPK1,TXNRD1,MLX,TARDBP,RASGEF1A,MOXD1,F8,EDNRA,SLA,CETN3,PSMD10,LEPROTL1,ALDH1A1,EPB41L3,SELENBP1,MMP7,ABCF3,RAB11A,RUVBL1,SNX1,PAK4,FGFR2,SLC25A10,FGA,DDC,FMN1,TAF7,SNX9,FBP1,ZNF655,SET,PPP3R1,PRKAR2B,RPS6,MYLK,VCP,MBIP,HSPBP1,AKT3,NUDT18,HIATL1,C1orf87,LHX1,ZMYM3,NPM1,CORO2B,CIAO1,ARHGAP29,KLHL14,CYGB,RTN4,PDXK,ANXA13,NFKBIA,ALDH9A1,ACVR2B,C8orf37 |
| TOV21G | 37 | 873 | 10.22099448 | 2.17322625 | 1.52E-05 | 0.006015 | 0.000106 | 2.45E-05 | PROSC,CSRP1,APOE,GSTP1,FUBP1,RPS7,TIMP1,RHOA,IMPDH2,GNB2L1,SFN,ANP32E,NME1,HN1,SPP1,APEX1,CCT4,YWHAB,DBNL,TTC1,UGDH,RBBP7,SERBP1,USP14,TXNRD1,TARDBP,RAB11A,RUVBL1,TPD52L2,SET,MYLK,VCP,FABP5,NPM1,PGM2,RTN4,RNPEP |
| H1688 | 39 | 951 | 10.77348066 | 2.099539487 | 1.86E-05 | 0.007383 | 0.000127 | 2.96E-05 | FHL2,FST,CSRP1,CARHSP1,GSTP1,RPS7,TIMP1,RHOA,IMPDH2,GNB2L1,SFN,ANP32E,NME1,HN1,SPP1,APEX1,CCT4,YWHAB,RPL14,USP15,UGDH,ENOPH1,PTPN11,RBBP7,RPL22,SERBP1,RPS13,USP14,TXNRD1,TARDBP,ALDH1A1,DDC,SET,VCP,SRP54,FABP5,NPM1,PGM2,ALDH9A1 |
| Stomach cancer | 186 | 7333 | 51.38121547 | 1.268163533 | 1.97E-05 | 0.007808 | 0.000132 | 3.07E-05 | CRYM,KCNMB3,HOXA5,EFCAB2,STAT6,ZHX3,CCNB1,DRG1,PROSC,DNAJB5,AFP,STK33,FHL2,NFE2,RAB3B,STAU2,MRPS7,CALCOCO2,FST,NRAS,TYROBP,RPL10,ERAL1,APOE,COX4I1,CD47,UPP2,HSPA2,GULP1,TM4SF1,GALT,ELOVL1,LYPD1,GSTP1,RPS6KA2,FUBP1,ATAD2,FAM63A,TIMP1,PPP2R4,SLC39A9,RHOA,DLX5,RAP1GDS1,HPX,SGK2,NECAP2,HLA-DOB,CHD4,ST6GALNAC6,DOK1,PSMD14,IMPDH2,PANK1,XPR1,DHX9,GNB2L1,MRTO4,CYB5R1,CDC34,CLDN4,RAB39B,RRAGB,SFN,ANP32E,NHEJ1,NME1,STARD10,ADD1,CAMK4,TEX264,ZNF641,ANAPC11,DDX58,ARPC3,SPP1,CLGN,SULT1E1,PTS,RBKS,CCNH,AAK1,APEX1,CCT4,CCR10,BAG5,MAPK3,CDCP1,YWHAB,DBNL,TCOF1,GGT6,RPL14,C1QC,C1orf21,USP15,MORC4,MIPOL1,PTPN11,PRKRA,SMYD3,MCM5,C14orf119,DIABLO,PDLIM3,CKAP2,NAGK,MEIS2,DERL1,SRPK2,HSPB8,SERBP1,TK1,MARCKSL1,CYP2C8,PRPSAP2,MATK,C9,GTF2I,BCL7C,CDH26,TOM1,PCGF3,PLCB2,PAK6,ANXA6,USP14,TEAD3,COASY,RECQL5,ANXA11,ABLIM3,MAPK1,TXNRD1,MLX,TARDBP,RASGEF1A,MOXD1,F8,EDNRA,SLA,CETN3,PSMD10,ALDH1A1,SELENBP1,MMP7,ABCF3,RAB11A,RUVBL1,PAK4,FGFR2,SLC25A10,FGA,DDC,FMN1,PECAM1,TAF7,SNX9,FBP1,ZNF655,SET,PPP3R1,PRKAR2B,RPS6,MYLK,VCP,RAC1,MBIP,HSPBP1,AKT3,NUDT18,HIATL1,LHX1,ZMYM3,NPM1,CIAO1,ARHGAP29,KLHL14,CYGB,RTN4,PDXK,ANXA13,NFKBIA,ALDH9A1,ACVR2B,C8orf37 |
| TOV112D | 47 | 1244 | 12.98342541 | 1.924641787 | 2.07E-05 | 0.008196 | 0.000137 | 3.17E-05 | DOHH,PROSC,FHL2,CSRP1,APOE,HPCAL1,CARHSP1,GSTP1,FUBP1,RPS7,TIMP1,PPP2R4,RHOA,GNB2L1,SFN,ANP32E,NME1,HN1,SPP1,APEX1,CCT4,YWHAB,TCOF1,TTC1,UGDH,PTPN11,RBBP7,SERBP1,GTF2I,USP14,MAPK1,TXNRD1,SRI,TARDBP,RAB11A,TPD52L2,SET,RPS6,VCP,CHORDC1,GBE1,FABP5,NPM1,PGM2,RTN4,RNPEP,ALDH9A1 |
| Malignant glioma | 181 | 7111 | 50 | 1.272827842 | 2.39E-05 | 0.009445 | 0.000155 | 3.59E-05 | CRYM,KCNMB3,HOXA5,EFCAB2,STAT6,ZHX3,CCNB1,DRG1,PROSC,DNAJB5,AFP,STK33,FHL2,NFE2,RAB3B,STAU2,MRPS7,CALCOCO2,FST,NRAS,TYROBP,RPL10,ERAL1,APOE,COX4I1,CD47,UPP2,HSPA2,GULP1,TM4SF1,GALT,ELOVL1,LYPD1,GSTP1,RPS6KA2,FUBP1,ATAD2,FAM63A,TIMP1,PPP2R4,SLC39A9,RHOA,DLX5,RAP1GDS1,HPX,SGK2,NECAP2,CHD4,ST6GALNAC6,DOK1,PSMD14,IMPDH2,PANK1,XPR1,DHX9,GNB2L1,CYB5R1,CDC34,CLDN4,RAB39B,PTN,RRAGB,ANP32E,NHEJ1,NME1,STARD10,ADD1,CAMK4,TEX264,ANAPC11,DDX58,ARPC3,SPP1,CLGN,SULT1E1,PTS,RBKS,CCNH,AAK1,APEX1,CCT4,CCR10,BAG5,MAPK3,CDCP1,YWHAB,DBNL,TCOF1,GGT6,RPL14,C1QC,C1orf21,USP15,MORC4,MIPOL1,PTPN11,PRKRA,SMYD3,MCM5,C14orf119,DIABLO,PDLIM3,CKAP2,NAGK,MEIS2,DERL1,SRPK2,HSPB8,SERBP1,TK1,MARCKSL1,CYP2C8,PRPSAP2,MATK,C9,GTF2I,BCL7C,CDH26,TOM1,PCGF3,PLCB2,PAK6,ANXA6,USP14,TEAD3,COASY,RECQL5,ANXA11,ABLIM3,MAPK1,TXNRD1,MLX,TARDBP,HCRTR1,RASGEF1A,F8,EDNRA,SLA,CETN3,PSMD10,ALDH1A1,SELENBP1,ABCF3,RAB11A,RUVBL1,PAK4,FGFR2,SLC25A10,FGA,DDC,FMN1,TAF7,SNX9,FBP1,ZNF655,SET,PPP3R1,PRKAR2B,RPS6,MYLK,VCP,RAC1,MBIP,HSPBP1,AKT3,NUDT18,HIATL1,LHX1,ZMYM3,NPM1,CORO2B,CIAO1,KLHL14,CYGB,RTN4,PDXK,ANXA13,NFKBIA,ALDH9A1,ACVR2B,C8orf37 |
| Testis cancer | 182 | 7183 | 50.27624309 | 1.266985633 | 2.98E-05 | 0.0118 | 0.00019 | 4.42E-05 | CRYM,KCNMB3,HOXA5,EFCAB2,STAT6,ZHX3,CCNB1,ADRB2,DRG1,PROSC,DNAJB5,AFP,STK33,FHL2,NFE2,RAB3B,STAU2,MRPS7,CALCOCO2,FST,NRAS,TYROBP,RPL10,ERAL1,APOE,COX4I1,CD47,HSPA2,GULP1,TM4SF1,GALT,ELOVL1,LYPD1,GSTP1,RPS6KA2,FUBP1,ATAD2,FAM63A,TIMP1,PPP2R4,SLC39A9,RHOA,DLX5,RAP1GDS1,HPX,NECAP2,CHD4,ST6GALNAC6,DOK1,PSMD14,IMPDH2,PANK1,DHX9,GNB2L1,MRTO4,CYB5R1,CDC34,CLDN4,RAB39B,PTN,RRAGB,SFN,ANP32E,NHEJ1,NME1,STARD10,ADD1,CAMK4,TEX264,ZNF641,ANAPC11,DDX58,ARPC3,SPP1,CLGN,SULT1E1,PTS,RBKS,CCNH,AAK1,APEX1,CCT4,CCR10,BAG5,MAPK3,CDCP1,YWHAB,DBNL,TCOF1,GGT6,RPL14,C1QC,C1orf21,USP15,MORC4,MIPOL1,PTPN11,PRKRA,SMYD3,MCM5,C14orf119,DIABLO,PDLIM3,CKAP2,NAGK,CPNE4,MEIS2,DERL1,SRPK2,HSPB8,SERBP1,TK1,MARCKSL1,CYP2C8,PRPSAP2,MATK,C9,GTF2I,BCL7C,CDH26,TOM1,PCGF3,PLCB2,PAK6,ANXA6,USP14,TEAD3,COASY,RECQL5,ANXA11,ABLIM3,MAPK1,TXNRD1,MLX,TARDBP,RASGEF1A,F8,EDNRA,CETN3,PSMD10,EPB41L3,SELENBP1,ABCF3,RAB11A,RUVBL1,PAK4,FGFR2,SLC25A10,FGA,DDC,FMN1,TAF7,SNX9,FBP1,ZNF655,SET,PPP3R1,PRKAR2B,RPS6,MYLK,VCP,RAC1,MBIP,HSPBP1,AKT3,HIATL1,C1orf87,LHX1,ZMYM3,NPM1,CORO2B,CIAO1,ARHGAP29,KLHL14,CYGB,RTN4,PDXK,ANXA13,NFKBIA,ALDH9A1,ACVR2B,C8orf37 |
| Smooth muscle cells | 137 | 5073 | 37.84530387 | 1.353325993 | 3.28E-05 | 0.013007 | 0.000206 | 4.79E-05 | CRYM,KCNMB3,EFCAB2,STAT6,ZHX3,DRG1,PROSC,DNAJB5,AFP,STK33,FHL2,NFE2,STAU2,MRPS7,CALCOCO2,FST,NRAS,TYROBP,RPL10,ERAL1,COX4I1,UPP2,HSPA2,GULP1,ELOVL1,LYPD1,GSTP1,FUBP1,ATAD2,FAM63A,PPP2R4,SLC39A9,ADRBK1,DLX5,CHD4,ST6GALNAC6,IMPDH2,DHX9,GNB2L1,CYB5R1,CDC34,RRAGB,SFN,NHEJ1,STARD10,CAMK4,ZNF641,ANAPC11,DDX58,SPP1,SULT1E1,PTS,RBKS,AAK1,APEX1,CCR10,BAG5,MAPK3,CDCP1,YWHAB,TCOF1,GGT6,RPL14,C1QC,C1orf21,USP15,MORC4,MIPOL1,PTPN11,PRKRA,SMYD3,C14orf119,DIABLO,PDLIM3,CKAP2,NAGK,CPNE4,MEIS2,SRPK2,HSPB8,SERBP1,PRPSAP2,C9,GTF2I,TOM1,PCGF3,PLCB2,PAK6,ANXA6,USP14,TEAD3,COASY,RECQL5,ANXA11,ABLIM3,MAPK1,TXNRD1,MLX,TARDBP,RASGEF1A,MOXD1,F8,EDNRA,CETN3,PSMD10,ALDH1A1,SELENBP1,RAB11A,RUVBL1,PAK4,FGFR2,SLC25A10,DDC,FMN1,TAF7,SNX9,ZNF655,SET,PPP3R1,RPS6,MYLK,VCP,RAC1,HSPBP1,AKT3,LHX1,ZMYM3,NPM1,CORO2B,CIAO1,ARHGAP29,KLHL14,RTN4,ANXA13,NFKBIA,ALDH9A1,ACVR2B |
| Renal cancer | 179 | 7062 | 49.44751381 | 1.267593019 | 3.72E-05 | 0.014744 | 0.00023 | 5.35E-05 | CRYM,KCNMB3,HOXA5,EFCAB2,STAT6,ZHX3,CCNB1,ADRB2,DRG1,PROSC,DNAJB5,AFP,STK33,FHL2,NFE2,RAB3B,STAU2,MRPS7,CALCOCO2,FST,NRAS,TYROBP,RPL10,ERAL1,APOE,COX4I1,CD47,HSPA2,GULP1,TM4SF1,GALT,ELOVL1,LYPD1,NAPSA,GSTP1,RPS6KA2,FUBP1,ATAD2,FAM63A,PPP2R4,SLC39A9,RHOA,DLX5,RAP1GDS1,HPX,SGK2,NECAP2,CHD4,ST6GALNAC6,DOK1,PSMD14,IMPDH2,DHX9,GNB2L1,MRTO4,CYB5R1,CDC34,CLDN4,RAB39B,RRAGB,SFN,ANP32E,NHEJ1,NME1,STARD10,ADD1,CAMK4,ANAPC11,DDX58,ARPC3,SPP1,CLGN,SULT1E1,PTS,RBKS,CCNH,AAK1,APEX1,CCT4,CCR10,BAG5,MAPK3,CDCP1,YWHAB,DBNL,TCOF1,GGT6,RPL14,C1QC,C1orf21,USP15,MORC4,MIPOL1,PTPN11,PRKRA,SMYD3,MCM5,C14orf119,DIABLO,CKAP2,NAGK,CPNE4,MEIS2,DERL1,SRPK2,HSPB8,SERBP1,MARCKSL1,PRPSAP2,MATK,C9,GTF2I,BCL7C,CDH26,TOM1,PCGF3,PLCB2,PAK6,ANXA6,USP14,TEAD3,COASY,RECQL5,ANXA11,ABLIM3,MAPK1,TXNRD1,MLX,TARDBP,MOXD1,F8,EDNRA,SLA,CETN3,PSMD10,ALDH1A1,EPB41L3,SELENBP1,ABCF3,RAB11A,RUVBL1,PAK4,FGFR2,SLC25A10,FGA,DDC,FMN1,TAF7,SNX9,FBP1,ZNF655,SET,PPP3R1,PRKAR2B,RPS6,MYLK,VCP,RAC1,MBIP,HSPBP1,AKT3,NUDT18,HIATL1,C1orf87,LHX1,ZMYM3,NPM1,CORO2B,CIAO1,ARHGAP29,KLHL14,CYGB,RTN4,PDXK,ANXA13,NFKBIA,ALDH9A1,ACVR2B,C8orf37 |
| 15_clinprot_c3 | 32 | 740 | 8.839779006 | 2.228107225 | 4E-05 | 0.01584 | 0.000244 | 5.66E-05 | CSRP1,APOE,HLA-DRB5,FUBP3,GSTP1,CORO1A,FUBP1,HPX,CHD4,PSMD14,IMPDH2,GNB2L1,SFN,ANP32E,HLA-DRB3,APEX1,C1QC,RBBP7,MCTS1,SERBP1,C9,GTF2I,TARDBP,ALDH1A1,RUVBL1,FGA,SET,RPS6,VCP,PLEK,ZRANB2,NPM1 |
| Lymph nodes | 170 | 6641 | 46.96132597 | 1.280632462 | 4.13E-05 | 0.016349 | 0.000248 | 5.75E-05 | CRYM,KCNMB3,HOXA5,EFCAB2,STAT6,CD84,ZHX3,CCNB1,DRG1,PROSC,DNAJB5,AFP,STK33,NFE2,STAU2,MRPS7,CALCOCO2,FST,NRAS,TYROBP,RPL10,ERAL1,APOE,COX4I1,CD47,UPP2,GALT,ELOVL1,LYPD1,GSTP1,RPS6KA2,CORO1A,FUBP1,ATAD2,FAM63A,CAPRIN2,OLR1,PPP2R4,SLC39A9,RHOA,LAYN,DLX5,RAP1GDS1,NECAP2,HLA-DOB,CHD4,ST6GALNAC6,DOK1,IMPDH2,XPR1,DHX9,GNB2L1,CYB5R1,CDC34,RAB39B,RRAGB,FN3K,NHEJ1,NME1,STARD10,ADD1,CAMK4,ANAPC11,DDX58,ARPC3,SPP1,KLF11,PTS,RBKS,AAK1,ARHGDIG,APEX1,CCT4,CCR10,BAG5,MAPK3,YWHAB,DBNL,TCOF1,GGT6,RPL14,C1QC,C1orf21,USP15,MORC4,MIPOL1,PTPN11,PRKRA,SMYD3,MCM5,C14orf119,DIABLO,PDLIM3,CKAP2,NAGK,CPNE4,MEIS2,DERL1,SRPK2,HSPB8,SERBP1,TK1,MARCKSL1,CYP2C8,PRPSAP2,MATK,C9,GTF2I,BCL7C,NDUFA8,DAPP1,TOM1,PCGF3,PLCB2,ANXA6,USP14,TEAD3,COASY,RECQL5,ANXA11,ABLIM3,MAPK1,TXNRD1,TARDBP,RASGEF1A,F8,EDNRA,SLA,CETN3,PSMD10,LEPROTL1,MMP7,ABCF3,RAB11A,RUVBL1,PAK4,FGFR2,DDC,FMN1,PECAM1,TAF7,SNX9,ZNF655,LST1,SET,PPP3R1,RPS6,MYLK,VCP,RAC1,MBIP,HSPBP1,PLEK,AKT3,HIATL1,GFM2,LHX1,ZMYM3,NPM1,CORO2B,CIAO1,ARHGAP29,KLHL14,RTN4,PDXK,ANXA13,NFKBIA,DGUOK,ALDH9A1,C8orf37 |
| PC3 | 46 | 1244 | 12.70718232 | 1.884752838 | 4.33E-05 | 0.017135 | 0.000256 | 5.94E-05 | CCNB1,SRPX2,RAB3B,FST,CSRP1,APOE,HPCAL1,HSPA2,CARHSP1,FUBP3,GSTP1,TIMP1,RHOA,IMPDH2,GNB2L1,RAB39B,SFN,NME1,HN1,APEX1,CCT4,CDCP1,ARF5,YWHAB,TTC1,UGDH,PTPN11,RBBP7,NAGK,MCTS1,SERBP1,MARCKSL1,RAB8B,USP14,ANXA11,TXNRD1,TARDBP,EPB41L3,SET,VCP,RAC1,CHORDC1,GBE1,FABP5,NPM1,RTN4 |
| Head & neck cancer | 177 | 6998 | 48.89502762 | 1.264990244 | 5.02E-05 | 0.01987 | 0.000292 | 6.78E-05 | CRYM,KCNMB3,HOXA5,EFCAB2,STAT6,ZHX3,CCNB1,DRG1,PROSC,DNAJB5,AFP,STK33,FHL2,NFE2,RAB3B,STAU2,MRPS7,CALCOCO2,FST,NRAS,TYROBP,RPL10,ERAL1,APOE,COX4I1,CD47,UPP2,HSPA2,GULP1,TM4SF1,GALT,ELOVL1,LYPD1,GSTP1,RPS6KA2,FUBP1,ATAD2,FAM63A,PPP2R4,SLC39A9,RHOA,DLX5,RAP1GDS1,NECAP2,CHD4,ST6GALNAC6,DOK1,PSMD14,IMPDH2,PANK1,XPR1,DHX9,GNB2L1,MRTO4,CYB5R1,CDC34,CLDN4,RAB39B,RRAGB,SFN,ANP32E,NHEJ1,NME1,STARD10,ADD1,CAMK4,TEX264,ZNF641,ANAPC11,DDX58,ARPC3,SPP1,CLGN,SULT1E1,PTS,RBKS,CCNH,AAK1,APEX1,CCT4,BAG5,MAPK3,CDCP1,YWHAB,DBNL,TCOF1,GGT6,RPL14,C1QC,C1orf21,USP15,MORC4,MIPOL1,PTPN11,PRKRA,SMYD3,MCM5,C14orf119,DIABLO,CKAP2,NAGK,MEIS2,DERL1,SRPK2,HSPB8,SERBP1,TK1,MARCKSL1,CYP2C8,PRPSAP2,MATK,C9,GTF2I,BCL7C,CDH26,TOM1,PCGF3,PLCB2,PAK6,ANXA6,USP14,TEAD3,COASY,RECQL5,ANXA11,ABLIM3,MAPK1,TXNRD1,MLX,TARDBP,F8,EDNRA,CETN3,PSMD10,ALDH1A1,SELENBP1,MMP7,ABCF3,RAB11A,RUVBL1,PAK4,FGFR2,SLC25A10,FGA,DDC,FMN1,PECAM1,TAF7,SNX9,FBP1,ZNF655,SET,PPP3R1,PRKAR2B,RPS6,MYLK,VCP,MBIP,HSPBP1,PLEK,AKT3,NUDT18,HIATL1,LHX1,ZMYM3,NPM1,CORO2B,CIAO1,KLHL14,CYGB,RTN4,PDXK,ANXA13,NFKBIA,ALDH9A1,ACVR2B,C8orf37 |
| Bronchus | 174 | 6862 | 48.06629834 | 1.268344855 | 5.42E-05 | 0.021446 | 0.000311 | 7.2E-05 | CRYM,KCNMB3,HOXA5,EFCAB2,STAT6,ZHX3,CCNB1,ADRB2,DRG1,PROSC,DNAJB5,AFP,STK33,FHL2,NFE2,RAB3B,STAU2,MRPS7,CALCOCO2,FST,NRAS,TYROBP,RPL10,ERAL1,APOE,COX4I1,CD47,UPP2,HSPA2,GULP1,TM4SF1,GALT,ELOVL1,LYPD1,GSTP1,RPS6KA2,FUBP1,ATAD2,FAM63A,TIMP1,STATH,PPP2R4,SLC39A9,RHOA,DLX5,RAP1GDS1,CHD4,ST6GALNAC6,DOK1,PSMD14,IMPDH2,DHX9,GNB2L1,MRTO4,CYB5R1,CDC34,CLDN4,RAB39B,RRAGB,SFN,ANP32E,NHEJ1,NME1,STARD10,ADD1,CAMK4,TEX264,ZNF641,ANAPC11,DDX58,ARPC3,SPP1,CLGN,SULT1E1,PTS,RBKS,CCNH,AAK1,APEX1,CCT4,CCR10,BAG5,MAPK3,CDCP1,YWHAB,DBNL,TCOF1,GGT6,RPL14,C1QC,C1orf21,USP15,MIPOL1,PTPN11,SMYD3,MCM5,C14orf119,DIABLO,PDLIM3,CKAP2,MEIS2,DERL1,SRPK2,HSPB8,SERBP1,TK1,MARCKSL1,CYP2C8,MATK,C9,GTF2I,BCL7C,CDH26,TOM1,PCGF3,PLCB2,PAK6,ANXA6,USP14,TEAD3,COASY,RECQL5,ANXA11,ABLIM3,MAPK1,TXNRD1,MLX,TARDBP,RASGEF1A,F8,EDNRA,CETN3,PSMD10,ALDH1A1,SELENBP1,ABCF3,RAB11A,RUVBL1,PAK4,FGFR2,SLC25A10,FGA,DDC,FMN1,TAF7,SNX9,ZNF655,SET,PPP3R1,PRKAR2B,RPS6,MYLK,VCP,RAC1,MBIP,HSPBP1,AKT3,NUDT18,HIATL1,C1orf87,LHX1,ZMYM3,NPM1,CORO2B,CIAO1,ARHGAP29,KLHL14,CYGB,RTN4,PDXK,ANXA13,NFKBIA,ACVR2B,C8orf37 |
| Rectum | 178 | 7056 | 49.17127072 | 1.261632319 | 5.48E-05 | 0.021717 | 0.00031 | 7.2E-05 | CRYM,KCNMB3,HOXA5,EFCAB2,STAT6,ZHX3,CCNB1,ADRB2,DRG1,PROSC,DNAJB5,AFP,STK33,FHL2,NFE2,RAB3B,STAU2,MRPS7,CALCOCO2,FST,NRAS,TYROBP,RPL10,ERAL1,APOE,COX4I1,UPP2,HSPA2,GULP1,GALT,ELOVL1,LYPD1,GSTP1,RPS6KA2,FUBP1,ATAD2,FAM63A,TIMP1,PPP2R4,SLC39A9,RHOA,DLX5,RAP1GDS1,SGK2,NECAP2,HLA-DOB,CHD4,ST6GALNAC6,DOK1,PSMD14,IMPDH2,PANK1,DHX9,GNB2L1,MRTO4,CYB5R1,CDC34,CLDN4,RAB39B,RRAGB,SFN,ANP32E,NHEJ1,NME1,STARD10,ADD1,CAMK4,TEX264,ZNF641,ANAPC11,DDX58,ARPC3,SPP1,CLGN,SULT1E1,RBKS,CCNH,AAK1,APEX1,CCT4,CCR10,BAG5,MAPK3,CDCP1,YWHAB,DBNL,TCOF1,GGT6,RPL14,C1QC,C1orf21,USP15,MORC4,MIPOL1,PTPN11,PRKRA,SMYD3,MCM5,C14orf119,DIABLO,PDLIM3,CKAP2,NAGK,MEIS2,DERL1,SRPK2,HSPB8,SERBP1,TK1,MARCKSL1,CYP2C8,PRPSAP2,MATK,C9,GTF2I,BCL7C,CDH26,TOM1,PCGF3,PLCB2,PAK6,ANXA6,USP14,TEAD3,COASY,RECQL5,ANXA11,ABLIM3,MAPK1,TXNRD1,MLX,TARDBP,RASGEF1A,F8,EDNRA,SLA,CETN3,PSMD10,EPB41L3,SELENBP1,ABCF3,RAB11A,RUVBL1,PAK4,FGFR2,SLC25A10,DDC,FMN1,TAF7,SNX9,FBP1,ZNF655,SET,PPP3R1,RPS6,MYLK,VCP,RAC1,MBIP,HSPBP1,AKT3,NUDT18,HIATL1,C1orf87,LHX1,ZMYM3,NPM1,CORO2B,CIAO1,ARHGAP29,KLHL14,CYGB,PDXK,ANXA13,NFKBIA,ALDH9A1,ACVR2B,C8orf37 |
| SH-SY5Y | 25 | 524 | 6.906077348 | 2.482403588 | 6.15E-05 | 0.024341 | 0.000343 | 7.96E-05 | C2orf44,OGFOD2,HSPA2,GSTP1,FUBP1,ATG3,BCAS2,ANP32E,NME1,RPS15,HN1,ARPC3,APEX1,CCT4,YWHAB,QTRT1,RBBP7,SERBP1,POLE3,SRI,RUVBL1,SET,COG3,FABP5,RTN4 |
| Skin | 181 | 7229 | 50 | 1.252054883 | 7.04E-05 | 0.027895 | 0.000387 | 8.99E-05 | CRYM,KCNMB3,HOXA5,EFCAB2,STAT6,ZHX3,CCNB1,ADRB2,DRG1,PROSC,DNAJB5,AFP,FHL2,NFE2,RAB3B,STAU2,MRPS7,CALCOCO2,FST,NRAS,TYROBP,RPL10,CSRP1,ERAL1,APOE,COX4I1,UPP2,HSPA2,TM4SF1,GALT,ELOVL1,LYPD1,GSTP1,RPS6KA2,CORO1A,FUBP1,ATAD2,CAPRIN2,TIMP1,PPP2R4,SLC39A9,QDPR,RHOA,CD96,DLX5,HPX,SGK2,NECAP2,CHD4,ST6GALNAC6,DOK1,PSMD14,IMPDH2,DHX9,GNB2L1,MRTO4,CYB5R1,FGF7,CDC34,CLDN4,PTN,RRAGB,SFN,ANP32E,SEPT1,NHEJ1,NME1,STARD10,ADD1,ZNF641,C6orf141,ANAPC11,DDX58,ARPC3,SPP1,SULT1E1,PTS,AAK1,APEX1,CCR10,BAG5,MAPK3,CDCP1,ARF5,YWHAB,DBNL,TCOF1,GGT6,RPL14,C1QC,C1orf21,MORC4,UGDH,ENOPH1,PRKRA,SMYD3,MCM5,C14orf119,DIABLO,PDLIM3,PI16,CKAP2,NAGK,CPNE4,MEIS2,MCTS1,DERL1,SRPK2,HSPB8,HBG2,SERBP1,SPATA7,TK1,CYP2C8,PRPSAP2,RAB8B,MATK,C9,GTF2I,BCL7C,TOM1,PCGF3,PLCB2,PAK6,ANXA6,USP14,COASY,RECQL5,ANXA11,ABLIM3,MAPK1,TXNRD1,CCL5,MLX,TARDBP,RASGEF1A,MOXD1,F8,EDNRA,SLA,CETN3,PSMD10,ALDH1A1,SELENBP1,MMP7,ABCF3,RAB11A,RUVBL1,TPD52L2,SNX1,PAK4,FGFR2,SLC25A10,S100A7A,FGA,FMN1,SNX9,ZNF655,SET,PPP3R1,RPS6,MYLK,VCP,RAC1,HSPBP1,AKT3,NUDT18,C1orf87,LHX1,FABP5,ZMYM3,NPM1,CIAO1,PGM2,KLHL14,RTN4,PDXK,ANXA13,NFKBIA,DGUOK,ALDH9A1 |
| Endometrial cancer | 184 | 7393 | 50.82872928 | 1.244437903 | 8.28E-05 | 0.032785 | 0.000449 | 0.000103 | CRYM,KCNMB3,HOXA5,EFCAB2,STAT6,ZHX3,CCNB1,DRG1,PROSC,DNAJB5,AFP,STK33,FHL2,NFE2,RAB3B,STAU2,MRPS7,CALCOCO2,FST,NRAS,TYROBP,RPL10,ERAL1,APOE,COX4I1,CD47,UPP2,HSPA2,GULP1,TM4SF1,GALT,ELOVL1,LYPD1,GSTP1,RPS6KA2,FUBP1,ATAD2,FAM63A,TIMP1,PPP2R4,SLC39A9,RHOA,DLX5,RAP1GDS1,HPX,NECAP2,CHD4,ST6GALNAC6,DOK1,PSMD14,IMPDH2,PANK1,XPR1,DHX9,GNB2L1,MRTO4,CYB5R1,CDC34,CLDN4,RAB39B,RRAGB,SFN,ANP32E,NHEJ1,NME1,STARD10,ADD1,CAMK4,TEX264,ANAPC11,DDX58,ARPC3,SPP1,CLGN,PTS,RBKS,CCNH,AAK1,APEX1,CCT4,CCR10,BAG5,MAPK3,CDCP1,YWHAB,DBNL,TCOF1,GGT6,RPL14,C1QC,C1orf21,USP15,MORC4,MIPOL1,PTPN11,PRKRA,SMYD3,MCM5,C14orf119,DIABLO,PDLIM3,CKAP2,NAGK,CPNE4,MEIS2,DERL1,SRPK2,HSPB8,SERBP1,TK1,MARCKSL1,CYP2C8,PRPSAP2,MATK,C9,GTF2I,BCL7C,CDH26,TOM1,PCGF3,PLCB2,ANXA6,USP14,TEAD3,COASY,RECQL5,ANXA11,ABLIM3,MAPK1,TXNRD1,MLX,TARDBP,MOXD1,F8,EDNRA,SLA,CETN3,PSMD10,ALDH1A1,SELENBP1,MMP7,ABCF3,RAB11A,RUVBL1,PAK4,FGFR2,SLC25A10,FGA,DDC,FMN1,PECAM1,TAF7,SNX9,FBP1,ZNF655,SET,PPP3R1,PRKAR2B,RPS6,MYLK,VCP,RAC1,MBIP,HSPBP1,PLEK,AKT3,NUDT18,HIATL1,C1orf87,LHX1,ZMYM3,NPM1,CORO2B,CIAO1,ARHGAP29,KLHL14,CYGB,RTN4,PDXK,ANXA13,NFKBIA,ALDH9A1,ACVR2B,C8orf37 |
| Testis | 239 | 10152 | 66.02209945 | 1.175350672 | 8.32E-05 | 0.032959 | 0.000445 | 0.000103 | CRYM,KCNMB3,HOXA5,EFCAB2,STAT6,ZHX3,CCNB1,ADRB2,DRG1,PROSC,DNAJB5,AFP,STK33,FHL2,NFE2,STAU2,MRPS7,DAZAP2,CALCOCO2,FST,NRAS,TYROBP,RPL10,CSRP1,ERAL1,APOE,HLA-DRB5,COX4I1,CD47,UPP2,HSPA2,GULP1,TM4SF1,GALT,SP110,ELOVL1,LYPD1,CARHSP1,GSTP1,RPS6KA2,FUBP1,ATAD2,FAM63A,RPS7,OLR1,PPP2R4,SLC39A9,QDPR,ROPN1L,RHOA,DLX5,RAP1GDS1,ATG3,HPX,SGK2,NECAP2,HLA-DOB,SPAG16,CHD4,ST6GALNAC6,DOK1,MRPS25,PSMD14,IMPDH2,GH2,PANK1,DHX9,GNB2L1,MRTO4,BCAS2,CYB5R1,CDC34,CLDN4,RAB39B,LCAT,PTN,RRAGB,CTNNAL1,SFN,ANP32E,FN3K,SEPT1,NHEJ1,NME1,STARD10,ADD1,CAMK4,TEX264,ZNF641,HN1,ANAPC11,DDX58,ARPC3,SPP1,CLGN,MTL5,SULT1E1,HLA-DRB3,EIF4EBP3,PTS,RBKS,CCNH,AAK1,APEX1,CCT4,FNIP1,CCR10,ABLIM1,BAG5,MAPK3,CDCP1,YWHAB,DBNL,TCOF1,GGT6,RPL14,C1QC,C1orf21,RNF32,USP15,MORC4,UGDH,ASF1A,MIPOL1,ENOPH1,PTPN11,PRKRA,SMYD3,MCM5,C14orf119,DIABLO,PDLIM3,PI16,CKAP2,NAGK,CPNE4,MEIS2,DERL1,SRPK2,HSPB8,ATP6V1E2,SERBP1,SPATA7,MARCKSL1,CYP2C8,STUB1,PRPSAP2,RAB8B,MATK,C9,GTF2I,BCL7C,CDH26,NDUFA8,TOM1,FBXL3,PCGF3,PLCB2,PAK6,ANXA6,USP14,CLP1,TEAD3,COASY,RECQL5,ANXA11,RNF11,ABLIM3,MAPK1,TXNRD1,SRI,MLX,TARDBP,HCRTR1,RASGEF1A,MOXD1,F8,EDNRA,SLA,CETN3,PSMD10,LRFN1,LEPROTL1,ALDH1A1,EPB41L3,SELENBP1,ABCF3,RAB11A,RUVBL1,TPD52L2,SNX1,PAK4,FGFR2,SLC25A10,FGA,LEMD1,DDC,FMN1,TAF7,HSPBAP1,SNX9,FBP1,ZNF655,SET,PPP3R1,PRKAR2B,RPS6,MYLK,VCP,RAC1,COG3,MBIP,HSPBP1,AKT3,NUDT18,PHKG2,HIATL1,C1orf87,LHX1,FABP5,SSPN,ZMYM3,NPM1,CORO2B,CIAO1,ARHGAP29,KLHL14,CYGB,RTN4,PDXK,RNPEP,FAM9C,ANXA13,NFKBIA,DGUOK,ALDH9A1,ACVR2B,C8orf37,UBE2O |
| Prostate cancer | 179 | 7152 | 49.44751381 | 1.251644552 | 8.4E-05 | 0.033257 | 0.000443 | 0.000103 | CRYM,KCNMB3,HOXA5,EFCAB2,STAT6,ZHX3,CCNB1,ADRB2,DRG1,PROSC,DNAJB5,AFP,STK33,FHL2,NFE2,RAB3B,STAU2,MRPS7,CALCOCO2,FST,NRAS,TYROBP,RPL10,ERAL1,APOE,COX4I1,CD47,HSPA2,GULP1,TM4SF1,GALT,ELOVL1,LYPD1,GSTP1,RPS6KA2,FUBP1,ATAD2,FAM63A,TIMP1,PPP2R4,SLC39A9,RHOA,DLX5,RAP1GDS1,NECAP2,CHD4,ST6GALNAC6,DOK1,PSMD14,IMPDH2,PANK1,DHX9,GNB2L1,MRTO4,CYB5R1,CDC34,CLDN4,RAB39B,RRAGB,SFN,ANP32E,NHEJ1,NME1,STARD10,ADD1,CAMK4,TEX264,ZNF641,ANAPC11,DDX58,ARPC3,SPP1,CLGN,SULT1E1,PTS,RBKS,CCNH,AAK1,APEX1,CCT4,CCR10,BAG5,MAPK3,CDCP1,YWHAB,DBNL,TCOF1,GGT6,RPL14,C1QC,C1orf21,USP15,MORC4,MIPOL1,PTPN11,SMYD3,MCM5,C14orf119,DIABLO,PDLIM3,CKAP2,NAGK,MEIS2,DERL1,SRPK2,HSPB8,SERBP1,TK1,MARCKSL1,CYP2C8,PRPSAP2,MATK,C9,GTF2I,BCL7C,CDH26,TOM1,PCGF3,PLCB2,PAK6,ANXA6,USP14,TEAD3,COASY,RECQL5,ANXA11,ABLIM3,MAPK1,TXNRD1,MLX,TARDBP,RASGEF1A,F8,EDNRA,SLA,CETN3,PSMD10,ALDH1A1,SELENBP1,MMP7,ABCF3,RAB11A,RUVBL1,PAK4,FGFR2,SLC25A10,FGA,DDC,FMN1,TAF7,SNX9,FBP1,ZNF655,SET,PPP3R1,PRKAR2B,RPS6,MYLK,VCP,RAC1,MBIP,HSPBP1,AKT3,HIATL1,C1orf87,LHX1,ZMYM3,NPM1,CIAO1,ARHGAP29,KLHL14,CYGB,RTN4,PDXK,ANXA13,NFKBIA,ALDH9A1,ACVR2B,C8orf37 |
| Tonsils | 180 | 7207 | 49.72375691 | 1.248985275 | 8.9E-05 | 0.035236 | 0.000464 | 0.000108 | CRYM,KCNMB3,HOXA5,EFCAB2,STAT6,ZHX3,CCNB1,DRG1,PROSC,DNAJB5,AFP,STK33,FHL2,NFE2,RAB3B,STAU2,MRPS7,CALCOCO2,FST,NRAS,TYROBP,RPL10,ERAL1,APOE,COX4I1,CD47,UPP2,HSPA2,GULP1,TM4SF1,GALT,ELOVL1,LYPD1,GSTP1,RPS6KA2,FUBP1,ATAD2,FAM63A,CAPRIN2,TIMP1,PPP2R4,SLC39A9,RHOA,DLX5,RAP1GDS1,NECAP2,HLA-DOB,CHD4,ST6GALNAC6,DOK1,PSMD14,IMPDH2,DHX9,GNB2L1,MRTO4,CYB5R1,CDC34,CLDN4,RAB39B,RRAGB,SFN,ANP32E,NHEJ1,NME1,STARD10,ADD1,CAMK4,ZNF641,ANAPC11,DDX58,ARPC3,SPP1,CLGN,SULT1E1,PTS,RBKS,AAK1,APEX1,CCT4,CCR10,BAG5,MAPK3,CDCP1,YWHAB,DBNL,TCOF1,GGT6,RPL14,C1QC,C1orf21,USP15,MORC4,MIPOL1,PTPN11,PRKRA,SMYD3,MCM5,C14orf119,DIABLO,PDLIM3,PI16,CKAP2,NAGK,CPNE4,MEIS2,DERL1,SRPK2,HSPB8,SERBP1,TK1,MARCKSL1,CYP2C8,PRPSAP2,MATK,C9,GTF2I,BCL7C,CDH26,TOM1,PCGF3,PLCB2,PAK6,ANXA6,USP14,TEAD3,COASY,RECQL5,ANXA11,ABLIM3,MAPK1,TXNRD1,MLX,TARDBP,RASGEF1A,F8,EDNRA,CETN3,PSMD10,ALDH1A1,EPB41L3,MMP7,ABCF3,RAB11A,RUVBL1,PAK4,FGFR2,SLC25A10,DDC,FMN1,PECAM1,TAF7,SNX9,FBP1,ZNF655,PPP3R1,PRKAR2B,RPS6,MYLK,VCP,RAC1,MBIP,HSPBP1,PLEK,AKT3,NUDT18,HIATL1,C1orf87,LHX1,ZMYM3,NPM1,CORO2B,CIAO1,ARHGAP29,KLHL14,RTN4,PDXK,ANXA13,NFKBIA,ALDH9A1,C8orf37 |
| LNCaP | 49 | 1398 | 13.5359116 | 1.783815339 | 9.12E-05 | 0.036131 | 0.000469 | 0.000109 | CSRP1,HPCAL1,HSPA2,CARHSP1,FUBP3,FUBP1,PPP2R4,RHOA,SGK2,IMPDH2,DHX9,GNB2L1,NME1,HN1,ARPC3,ARHGDIG,APEX1,CCT4,CDCP1,ARF5,YWHAB,DBNL,TTC1,UGDH,ENOPH1,PTPN11,DIABLO,RBBP7,SERBP1,MARCKSL1,PYCR1,NDUFA8,USP14,TXNRD1,TARDBP,PSMD10,SELENBP1,MMP7,RAB11A,SET,VCP,SRP54,CHORDC1,ZRANB2,NPM1,PGM2,RTN4,RNPEP,ALDH9A1 |
| Breast cancer | 184 | 7408 | 50.82872928 | 1.241918543 | 9.44E-05 | 0.037368 | 0.000479 | 0.000111 | CRYM,KCNMB3,HOXA5,EFCAB2,STAT6,ZHX3,CCNB1,ADRB2,DRG1,PROSC,DNAJB5,AFP,STK33,FHL2,NFE2,RAB3B,STAU2,MRPS7,CALCOCO2,FST,NRAS,TYROBP,RPL10,ERAL1,APOE,COX4I1,CD47,UPP2,HSPA2,GULP1,TM4SF1,GALT,ELOVL1,LYPD1,GSTP1,RPS6KA2,FUBP1,ATAD2,FAM63A,TIMP1,PPP2R4,SLC39A9,RHOA,DLX5,RAP1GDS1,SGK2,NECAP2,CHD4,ST6GALNAC6,DOK1,PSMD14,IMPDH2,PANK1,XPR1,DHX9,GNB2L1,MRTO4,CYB5R1,CDC34,CLDN4,RAB39B,RRAGB,SFN,ANP32E,NHEJ1,NME1,STARD10,ADD1,CAMK4,TEX264,ZNF641,ANAPC11,DDX58,ARPC3,SPP1,CLGN,SULT1E1,PTS,RBKS,CCNH,AAK1,APEX1,CCT4,CCR10,BAG5,MAPK3,CDCP1,YWHAB,DBNL,TCOF1,GGT6,RPL14,C1QC,C1orf21,USP15,MORC4,MIPOL1,PTPN11,PRKRA,SMYD3,MCM5,C14orf119,DIABLO,PDLIM3,CKAP2,NAGK,CPNE4,MEIS2,DERL1,SRPK2,HSPB8,SERBP1,TK1,MARCKSL1,CYP2C8,PRPSAP2,MATK,C9,GTF2I,BCL7C,TOM1,PCGF3,PLCB2,ANXA6,USP14,TEAD3,COASY,RECQL5,ANXA11,ABLIM3,MAPK1,TXNRD1,MLX,TARDBP,RASGEF1A,MOXD1,F8,EDNRA,CETN3,PSMD10,ALDH1A1,EPB41L3,SELENBP1,MMP7,ABCF3,RAB11A,RUVBL1,PAK4,FGFR2,SLC25A10,FGA,DDC,FMN1,TAF7,SNX9,FBP1,ZNF655,SET,PPP3R1,PRKAR2B,RPS6,MYLK,VCP,RAC1,MBIP,HSPBP1,AKT3,NUDT18,HIATL1,LHX1,ZMYM3,NPM1,CORO2B,CIAO1,ARHGAP29,KLHL14,CYGB,RTN4,PDXK,ANXA13,NFKBIA,ALDH9A1,ACVR2B,C8orf37 |
| Lung cancer | 183 | 7372 | 50.55248619 | 1.241245482 | 0.000105 | 0.041698 | 0.000528 | 0.000122 | CRYM,KCNMB3,HOXA5,EFCAB2,STAT6,ZHX3,CCNB1,DRG1,PROSC,DNAJB5,AFP,STK33,FHL2,NFE2,RAB3B,STAU2,MRPS7,CALCOCO2,FST,NRAS,TYROBP,RPL10,ERAL1,APOE,COX4I1,CD47,UPP2,HSPA2,GULP1,TM4SF1,GALT,ELOVL1,LYPD1,NAPSA,GSTP1,RPS6KA2,FUBP1,ATAD2,FAM63A,TIMP1,PPP2R4,SLC39A9,RHOA,DLX5,RAP1GDS1,HPX,NECAP2,HLA-DOB,CHD4,ST6GALNAC6,DOK1,PSMD14,IMPDH2,XPR1,DHX9,GNB2L1,MRTO4,CYB5R1,CDC34,CLDN4,RAB39B,PTN,RRAGB,SFN,ANP32E,NHEJ1,NME1,STARD10,ADD1,CAMK4,TEX264,ANAPC11,DDX58,ARPC3,SPP1,CLGN,SULT1E1,PTS,RBKS,CCNH,AAK1,APEX1,CCT4,CCR10,BAG5,MAPK3,CDCP1,YWHAB,DBNL,TCOF1,GGT6,RPL14,C1QC,C1orf21,USP15,MORC4,MIPOL1,PTPN11,PRKRA,SMYD3,MCM5,C14orf119,DIABLO,PDLIM3,CKAP2,NAGK,MEIS2,DERL1,SRPK2,HSPB8,SERBP1,TK1,MARCKSL1,CYP2C8,PRPSAP2,MATK,C9,GTF2I,BCL7C,CDH26,TOM1,PCGF3,PLCB2,PAK6,ANXA6,USP14,TEAD3,COASY,RECQL5,ANXA11,ABLIM3,MAPK1,TXNRD1,MLX,TARDBP,MOXD1,F8,EDNRA,SLA,CETN3,PSMD10,ALDH1A1,SELENBP1,MMP7,ABCF3,RAB11A,RUVBL1,PAK4,FGFR2,SLC25A10,FGA,DDC,FMN1,PECAM1,TAF7,SNX9,FBP1,ZNF655,SET,PPP3R1,RPS6,MYLK,VCP,RAC1,MBIP,HSPBP1,AKT3,NUDT18,HIATL1,LHX1,ZMYM3,NPM1,CORO2B,CIAO1,KLHL14,CYGB,RTN4,PDXK,ANXA13,NFKBIA,ALDH9A1,ACVR2B,C8orf37 |
| Corpus, uterine | 174 | 6937 | 48.06629834 | 1.254634501 | 0.000106 | 0.041944 | 0.000524 | 0.000122 | CRYM,KCNMB3,HOXA5,EFCAB2,STAT6,ZHX3,CCNB1,DRG1,PROSC,DNAJB5,AFP,STK33,FHL2,NFE2,STAU2,MRPS7,CALCOCO2,FST,NRAS,TYROBP,RPL10,ERAL1,APOE,COX4I1,CD47,UPP2,HSPA2,GULP1,TM4SF1,GALT,ELOVL1,LYPD1,GSTP1,RPS6KA2,FUBP1,ATAD2,FAM63A,TIMP1,PPP2R4,SLC39A9,RHOA,DLX5,RAP1GDS1,NECAP2,CHD4,DOK1,PSMD14,IMPDH2,XPR1,DHX9,GNB2L1,MRTO4,CYB5R1,CDC34,CLDN4,RAB39B,RRAGB,SFN,ANP32E,NHEJ1,NME1,STARD10,ADD1,CAMK4,TEX264,ZNF641,ANAPC11,DDX58,ARPC3,SPP1,CLGN,SULT1E1,PTS,RBKS,AAK1,APEX1,CCT4,CCR10,BAG5,MAPK3,CDCP1,YWHAB,DBNL,TCOF1,GGT6,RPL14,C1QC,C1orf21,USP15,MORC4,MIPOL1,PTPN11,PRKRA,SMYD3,MCM5,C14orf119,DIABLO,PDLIM3,CKAP2,NAGK,MEIS2,DERL1,SRPK2,HSPB8,SERBP1,TK1,MARCKSL1,PRPSAP2,MATK,C9,GTF2I,BCL7C,CDH26,TOM1,PCGF3,PLCB2,ANXA6,USP14,TEAD3,COASY,ANXA11,ABLIM3,MAPK1,TXNRD1,MLX,TARDBP,MOXD1,F8,EDNRA,SLA,CETN3,PSMD10,ALDH1A1,SELENBP1,MMP7,ABCF3,RAB11A,RUVBL1,PAK4,FGFR2,SLC25A10,FGA,DDC,FMN1,TAF7,SNX9,FBP1,ZNF655,SET,PRKAR2B,RPS6,MYLK,VCP,RAC1,MBIP,HSPBP1,PLEK,AKT3,HIATL1,C1orf87,LHX1,ZMYM3,NPM1,CORO2B,CIAO1,ARHGAP29,KLHL14,CYGB,PDXK,ANXA13,NFKBIA,ALDH9A1,ACVR2B,C8orf37 |
| Ovarian cancer | 187 | 7578 | 51.65745856 | 1.233723667 | 0.000116 | 0.045824 | 0.000566 | 0.000131 | CRYM,KCNMB3,HOXA5,EFCAB2,STAT6,ZHX3,CCNB1,DRG1,PROSC,DNAJB5,AFP,STK33,FHL2,NFE2,RAB3B,STAU2,MRPS7,CALCOCO2,FST,NRAS,RPL10,ERAL1,APOE,COX4I1,CD47,UPP2,HSPA2,GULP1,TM4SF1,GALT,ELOVL1,LYPD1,GSTP1,RPS6KA2,FUBP1,ATAD2,FAM63A,TIMP1,PPP2R4,SLC39A9,RHOA,DLX5,RAP1GDS1,HPX,SGK2,NECAP2,HLA-DOB,CHD4,ST6GALNAC6,DOK1,PSMD14,IMPDH2,PANK1,XPR1,DHX9,GNB2L1,MRTO4,CYB5R1,CDC34,CLDN4,RAB39B,RRAGB,SFN,ANP32E,NHEJ1,NME1,STARD10,ADD1,CAMK4,TEX264,ZNF641,ANAPC11,DDX58,ARPC3,SPP1,CLGN,SULT1E1,PTS,RBKS,CCNH,AAK1,APEX1,CCT4,CCR10,BAG5,MAPK3,CDCP1,YWHAB,DBNL,TCOF1,GGT6,RPL14,C1QC,C1orf21,USP15,MORC4,MIPOL1,PTPN11,PRKRA,SMYD3,MCM5,C14orf119,DIABLO,PDLIM3,CKAP2,NAGK,CPNE4,MEIS2,DERL1,SRPK2,HSPB8,SERBP1,TK1,MARCKSL1,CYP2C8,PRPSAP2,MATK,C9,GTF2I,BCL7C,CDH26,TOM1,PCGF3,PLCB2,PAK6,ANXA6,USP14,TEAD3,COASY,RECQL5,ANXA11,ABLIM3,MAPK1,TXNRD1,MLX,TARDBP,MOXD1,F8,EDNRA,SLA,CETN3,PSMD10,ALDH1A1,SELENBP1,MMP7,ABCF3,RAB11A,RUVBL1,TPD52L2,PAK4,FGFR2,SLC25A10,FGA,DDC,FMN1,TAF7,SNX9,FBP1,ZNF655,SET,PPP3R1,PRKAR2B,RPS6,MYLK,VCP,RAC1,MBIP,HSPBP1,AKT3,NUDT18,HIATL1,C1orf87,LHX1,ZMYM3,NPM1,CORO2B,CIAO1,ARHGAP29,KLHL14,CYGB,RTN4,PDXK,ANXA13,NFKBIA,ALDH9A1,ACVR2B,C8orf37 |
| H460 | 30 | 714 | 8.287292818 | 2.170207694 | 0.000117 | 0.046262 | 0.000564 | 0.000131 | SRPX2,FST,CSRP1,GSTP1,FUBP1,RPS7,TIMP1,RHOA,IMPDH2,GNB2L1,SFN,ANP32E,NME1,HN1,SPP1,APEX1,CCT4,YWHAB,UGDH,RBBP7,SERBP1,TXNRD1,EPB41L3,SET,VCP,GBE1,FABP5,NPM1,PGM2,RNPEP |
| H23 | 34 | 855 | 9.392265193 | 2.044879308 | 0.000119 | 0.04712 | 0.000568 | 0.000132 | DOHH,FHL2,CSRP1,APOE,CARHSP1,GSTP1,FUBP1,RPS7,TIMP1,IMPDH2,GNB2L1,SFN,ANP32E,NME1,HN1,APEX1,CCT4,YWHAB,ENOPH1,RBBP7,SERBP1,USP14,ANXA11,TXNRD1,TARDBP,RUVBL1,TPD52L2,SET,VCP,FABP5,NPM1,PGM2,RTN4,RNPEP |
| Red blood cells | 12 | 165 | 3.314917127 | 3.958798234 | 0.000128 | 0.050714 | 0.000604 | 0.000138 | CD47,GALT,GSTP1,QDPR,FN3K,NAGK,HBG2,PYCR1,TXNRD1,PHKG2,PGM2,PDXK |
| H520 | 33 | 823 | 9.116022099 | 2.064005088 | 0.000129 | 0.050986 | 0.0006 | 0.000138 | PROSC,CSRP1,CARHSP1,GSTP1,RPS7,IMPDH2,GNB2L1,VSNL1,SFN,NME1,HN1,SPP1,APEX1,CCT4,YWHAB,RBBP7,RPL22,HBG2,SERBP1,MARCKSL1,RPS13,MAPK1,TXNRD1,TARDBP,ALDH1A1,TPD52L2,SET,VCP,CHORDC1,FABP5,NPM1,PGM2,RNPEP |
| Malignant lymphoma | 165 | 6528 | 45.5801105 | 1.264758188 | 0.000129 | 0.051155 | 0.000595 | 0.000138 | CRYM,KCNMB3,HOXA5,EFCAB2,STAT6,ZHX3,CCNB1,ADRB2,DRG1,PROSC,DNAJB5,AFP,STK33,FHL2,NFE2,STAU2,MRPS7,CALCOCO2,FST,NRAS,TYROBP,RPL10,ERAL1,APOE,COX4I1,CD47,UPP2,HSPA2,TM4SF1,GALT,ELOVL1,LYPD1,GSTP1,RPS6KA2,FUBP1,ATAD2,FAM63A,TIMP1,PPP2R4,SLC39A9,RHOA,DLX5,RAP1GDS1,NECAP2,HLA-DOB,CHD4,DOK1,PSMD14,IMPDH2,PANK1,DHX9,GNB2L1,MRTO4,CYB5R1,CDC34,RAB39B,RRAGB,ANP32E,NHEJ1,NME1,STARD10,ADD1,CAMK4,TEX264,ANAPC11,DDX58,ARPC3,SPP1,CLGN,PTS,RBKS,CCNH,AAK1,APEX1,CCT4,CCR10,BAG5,MAPK3,CDCP1,YWHAB,DBNL,TCOF1,GGT6,RPL14,C1QC,C1orf21,MORC4,MIPOL1,PTPN11,PRKRA,SMYD3,MCM5,DIABLO,PDLIM3,CKAP2,NAGK,MEIS2,DERL1,SRPK2,HSPB8,SERBP1,TK1,MARCKSL1,PRPSAP2,MATK,C9,GTF2I,BCL7C,CDH26,TOM1,PCGF3,PLCB2,ANXA6,USP14,TEAD3,COASY,ANXA11,ABLIM3,MAPK1,TXNRD1,MLX,TARDBP,F8,EDNRA,SLA,CETN3,PSMD10,MMP7,ABCF3,RAB11A,RUVBL1,PAK4,SLC25A10,DDC,FMN1,PECAM1,TAF7,SNX9,FBP1,SET,PPP3R1,PRKAR2B,RPS6,MYLK,VCP,RAC1,MBIP,HSPBP1,PLEK,AKT3,HIATL1,LHX1,ZMYM3,NPM1,CORO2B,CIAO1,KLHL14,CYGB,RTN4,PDXK,ANXA13,NFKBIA,ALDH9A1,ACVR2B,C8orf37 |
| Colorectal cancer | 184 | 7446 | 50.82872928 | 1.235581585 | 0.000131 | 0.051802 | 0.000595 | 0.000138 | CRYM,KCNMB3,HOXA5,EFCAB2,STAT6,ZHX3,CCNB1,ADRB2,DRG1,PROSC,DNAJB5,AFP,STK33,FHL2,NFE2,RAB3B,STAU2,MRPS7,CALCOCO2,FST,NRAS,TYROBP,RPL10,ERAL1,APOE,COX4I1,UPP2,HSPA2,GULP1,TM4SF1,GALT,ELOVL1,LYPD1,GSTP1,RPS6KA2,FUBP1,ATAD2,FAM63A,TIMP1,PPP2R4,SLC39A9,RHOA,DLX5,RAP1GDS1,HPX,NECAP2,HLA-DOB,CHD4,ST6GALNAC6,DOK1,PSMD14,IMPDH2,PANK1,DHX9,GNB2L1,MRTO4,CYB5R1,CDC34,CLDN4,RAB39B,RRAGB,SFN,ANP32E,NHEJ1,NME1,STARD10,ADD1,CAMK4,TEX264,ZNF641,ANAPC11,DDX58,ARPC3,SPP1,CLGN,SULT1E1,PTS,RBKS,CCNH,AAK1,APEX1,CCT4,CCR10,BAG5,MAPK3,CDCP1,YWHAB,DBNL,TCOF1,GGT6,RPL14,C1QC,C1orf21,USP15,MORC4,MIPOL1,PTPN11,PRKRA,SMYD3,MCM5,C14orf119,DIABLO,PDLIM3,CKAP2,NAGK,CPNE4,MEIS2,DERL1,SRPK2,HSPB8,SERBP1,TK1,MARCKSL1,CYP2C8,PRPSAP2,MATK,C9,GTF2I,BCL7C,CDH26,TOM1,PCGF3,PLCB2,ANXA6,USP14,TEAD3,COASY,RECQL5,ANXA11,ABLIM3,MAPK1,TXNRD1,MLX,TARDBP,RASGEF1A,MOXD1,F8,EDNRA,CETN3,PSMD10,ALDH1A1,SELENBP1,MMP7,ABCF3,RAB11A,RUVBL1,PAK4,FGFR2,SLC25A10,FGA,DDC,FMN1,TAF7,SNX9,FBP1,ZNF655,SET,PPP3R1,PRKAR2B,RPS6,MYLK,VCP,RAC1,MBIP,HSPBP1,PLEK,AKT3,NUDT18,HIATL1,LHX1,ZMYM3,NPM1,CORO2B,CIAO1,ARHGAP29,KLHL14,CYGB,RTN4,PDXK,ANXA13,NFKBIA,ALDH9A1,ACVR2B,C8orf37 |
| Small intestine | 201 | 8289 | 55.52486188 | 1.211797484 | 0.000139 | 0.054993 | 0.000625 | 0.000144 | CRYM,KCNMB3,HOXA5,EFCAB2,STAT6,ZHX3,CCNB1,ADRB2,DRG1,PROSC,DNAJB5,SRPX2,AFP,STK33,FHL2,NFE2,RAB3B,STAU2,MRPS7,DAZAP2,CALCOCO2,FST,NRAS,TYROBP,RPL10,ERAL1,COX4I1,UPP2,HSPA2,GULP1,TM4SF1,GALT,SP110,ELOVL1,LYPD1,GSTP1,RPS6KA2,FUBP1,ATAD2,FAM63A,PPP2R4,SLC39A9,RHOA,DLX5,RAP1GDS1,ATG3,SGK2,NECAP2,HLA-DOB,CHD4,ST6GALNAC6,DOK1,PSMD14,IMPDH2,PANK1,DHX9,GNB2L1,BCAS2,CYB5R1,CDC34,CLDN4,RAB39B,RRAGB,CTNNAL1,SFN,ANP32E,FN3K,NHEJ1,NME1,ADD1,CAMK4,TEX264,ZNF641,TMEM185A,ANAPC11,DDX58,ARPC3,SPP1,SULT1E1,EIF4EBP3,RBKS,CCNH,AAK1,ARHGDIG,APEX1,CCR10,ABLIM1,BAG5,MAPK3,TIRAP,CDCP1,YWHAB,DBNL,TCOF1,GGT6,RPL14,C1QC,C1orf21,USP15,MORC4,UGDH,ASF1A,MIPOL1,PTPN11,PRKRA,SMYD3,MCM5,C14orf119,DIABLO,PDLIM3,PI16,CKAP2,CPNE4,MEIS2,DERL1,HSPB8,SERBP1,TK1,MARCKSL1,CYP2C8,PRPSAP2,MATK,C9,GTF2I,BCL7C,CDH26,NDUFA8,TOM1,FBXL3,PCGF3,PLCB2,PAK6,ANXA6,USP14,CLP1,TEAD3,COASY,RECQL5,ANXA11,RNF11,ABLIM3,MAPK1,TXNRD1,MLX,TARDBP,HCRTR1,F8,EDNRA,SLA,CETN3,PSMD10,LEPROTL1,ALDH1A1,EPB41L3,SAR1B,SELENBP1,MMP7,ABCF3,RAB11A,RUVBL1,SNX1,PAK4,FGFR2,SLC25A10,DDC,FMN1,PECAM1,TAF7,HSPBAP1,SNX9,FBP1,ZNF655,SET,PPP3R1,RPS6,MYLK,VCP,RAC1,COG3,MBIP,HSPBP1,AKT3,NUDT18,HIATL1,C1orf87,LHX1,FABP5,ZMYM3,NPM1,CIAO1,ARHGAP29,KLHL14,CYGB,RTN4,PDXK,ANXA13,NFKBIA,ALDH9A1,ACVR2B,C8orf37,UBE2O |
| Urothelial cancer | 184 | 7455 | 50.82872928 | 1.234090188 | 0.000141 | 0.055912 | 0.000628 | 0.000144 | KCNMB3,HOXA5,EFCAB2,STAT6,ZHX3,CCNB1,ADRB2,DRG1,PROSC,DNAJB5,AFP,STK33,FHL2,NFE2,RAB3B,STAU2,MRPS7,CALCOCO2,FST,NRAS,TYROBP,RPL10,ERAL1,APOE,COX4I1,CD47,UPP2,HSPA2,GULP1,TM4SF1,GALT,ELOVL1,LYPD1,GSTP1,RPS6KA2,FUBP1,ATAD2,FAM63A,PPP2R4,SLC39A9,RHOA,DLX5,RAP1GDS1,HPX,SGK2,NECAP2,CHD4,ST6GALNAC6,DOK1,PSMD14,IMPDH2,XPR1,DHX9,GNB2L1,MRTO4,CYB5R1,CDC34,CLDN4,RAB39B,PTN,RRAGB,SFN,ANP32E,NHEJ1,NME1,STARD10,ADD1,CAMK4,TEX264,ANAPC11,DDX58,ARPC3,SPP1,CLGN,SULT1E1,PTS,RBKS,CCNH,AAK1,APEX1,CCT4,CCR10,BAG5,MAPK3,CDCP1,YWHAB,DBNL,TCOF1,GGT6,RPL14,C1QC,C1orf21,USP15,MORC4,MIPOL1,PTPN11,PRKRA,SMYD3,MCM5,C14orf119,DIABLO,PDLIM3,CKAP2,NAGK,MEIS2,DERL1,SRPK2,HSPB8,SERBP1,TK1,MARCKSL1,CYP2C8,PRPSAP2,MATK,C9,GTF2I,BCL7C,CDH26,TOM1,PCGF3,PLCB2,PAK6,ANXA6,USP14,TEAD3,COASY,RECQL5,ANXA11,ABLIM3,MAPK1,TXNRD1,MLX,TARDBP,MOXD1,F8,EDNRA,SLA,CETN3,PSMD10,EPB41L3,SELENBP1,MMP7,ABCF3,RAB11A,RUVBL1,PAK4,FGFR2,SLC25A10,FGA,DDC,FMN1,TAF7,SNX9,FBP1,ZNF655,SET,PPP3R1,PRKAR2B,RPS6,MYLK,VCP,RAC1,MBIP,HSPBP1,PLEK,AKT3,NUDT18,HIATL1,C1orf87,LHX1,ZMYM3,NPM1,CORO2B,CIAO1,ARHGAP29,KLHL14,CYGB,RTN4,PDXK,ANXA13,NFKBIA,ALDH9A1,ACVR2B,C8orf37 |
| Ubiquitous | 39 | 1045 | 10.77348066 | 1.910907026 | 0.000142 | 0.056351 | 0.000626 | 0.000144 | STAT6,ZHX3,PROSC,STAU2,DAZAP2,ATG3,BCAS2,SEPT1,NHEJ1,ADD1,KLF11,APEX1,MAPK3,ARF5,DBNL,TCOF1,TTC1,PTPN11,PRKRA,MCTS1,GTF2I,NDUFA8,TOM1,CLP1,RECQL5,TARDBP,SLA,ABCF3,RAB11A,RUVBL1,SNX1,DDC,MYLK,RAC1,COG3,GFM2,ZRANB2,CYGB,RNPEP |
| Cervical cancer | 182 | 7359 | 50.27624309 | 1.23668918 | 0.000143 | 0.056569 | 0.000622 | 0.000144 | CRYM,KCNMB3,HOXA5,EFCAB2,STAT6,ZHX3,CCNB1,ADRB2,DRG1,PROSC,DNAJB5,AFP,STK33,FHL2,NFE2,RAB3B,STAU2,MRPS7,CALCOCO2,FST,NRAS,TYROBP,RPL10,ERAL1,APOE,COX4I1,CD47,UPP2,HSPA2,GULP1,TM4SF1,GALT,ELOVL1,LYPD1,GSTP1,RPS6KA2,FUBP1,ATAD2,FAM63A,TIMP1,PPP2R4,SLC39A9,RHOA,DLX5,RAP1GDS1,NECAP2,HLA-DOB,CHD4,ST6GALNAC6,DOK1,PSMD14,IMPDH2,PANK1,XPR1,DHX9,GNB2L1,MRTO4,CYB5R1,CDC34,CLDN4,RAB39B,RRAGB,SFN,ANP32E,NHEJ1,NME1,STARD10,ADD1,CAMK4,TEX264,ZNF641,ANAPC11,DDX58,ARPC3,SPP1,CLGN,SULT1E1,PTS,RBKS,CCNH,AAK1,APEX1,CCT4,CCR10,BAG5,MAPK3,CDCP1,YWHAB,DBNL,TCOF1,GGT6,RPL14,C1QC,C1orf21,USP15,MORC4,MIPOL1,PTPN11,PRKRA,SMYD3,MCM5,C14orf119,DIABLO,PDLIM3,CKAP2,NAGK,MEIS2,DERL1,SRPK2,HSPB8,SERBP1,TK1,MARCKSL1,PRPSAP2,MATK,C9,GTF2I,BCL7C,CDH26,TOM1,PCGF3,PLCB2,ANXA6,USP14,TEAD3,COASY,RECQL5,ANXA11,ABLIM3,MAPK1,TXNRD1,MLX,TARDBP,MOXD1,F8,EDNRA,CETN3,PSMD10,ALDH1A1,SELENBP1,MMP7,ABCF3,RAB11A,RUVBL1,PAK4,FGFR2,SLC25A10,FGA,DDC,FMN1,TAF7,SNX9,FBP1,ZNF655,SET,PPP3R1,PRKAR2B,RPS6,MYLK,VCP,RAC1,MBIP,HSPBP1,AKT3,NUDT18,HIATL1,C1orf87,LHX1,ZMYM3,NPM1,CORO2B,CIAO1,ARHGAP29,KLHL14,CYGB,RTN4,PDXK,ANXA13,NFKBIA,ALDH9A1,ACVR2B,C8orf37 |
| Thyroid cancer | 179 | 7218 | 49.44751381 | 1.240201738 | 0.000149 | 0.058872 | 0.00064 | 0.000149 | KCNMB3,HOXA5,EFCAB2,STAT6,ZHX3,CCNB1,DRG1,PROSC,DNAJB5,AFP,STK33,FHL2,NFE2,RAB3B,STAU2,MRPS7,CALCOCO2,FST,NRAS,TYROBP,RPL10,ERAL1,APOE,COX4I1,CD47,UPP2,HSPA2,GULP1,TM4SF1,GALT,ELOVL1,LYPD1,NAPSA,GSTP1,RPS6KA2,FUBP1,ATAD2,FAM63A,PPP2R4,SLC39A9,RHOA,DLX5,RAP1GDS1,NECAP2,HLA-DOB,CHD4,ST6GALNAC6,DOK1,PSMD14,IMPDH2,PANK1,XPR1,DHX9,GNB2L1,MRTO4,CYB5R1,CDC34,CLDN4,RAB39B,RRAGB,SFN,ANP32E,NHEJ1,NME1,STARD10,ADD1,CAMK4,TEX264,ZNF641,ANAPC11,DDX58,ARPC3,SPP1,CLGN,SULT1E1,PTS,RBKS,CCNH,AAK1,APEX1,CCT4,CCR10,BAG5,MAPK3,CDCP1,YWHAB,DBNL,TCOF1,GGT6,RPL14,C1QC,C1orf21,USP15,MORC4,MIPOL1,PTPN11,PRKRA,SMYD3,MCM5,C14orf119,DIABLO,PDLIM3,CKAP2,NAGK,MEIS2,DERL1,SRPK2,HSPB8,SERBP1,TK1,MARCKSL1,CYP2C8,PRPSAP2,MATK,C9,GTF2I,BCL7C,CDH26,TOM1,PCGF3,PLCB2,ANXA6,USP14,TEAD3,COASY,RECQL5,ANXA11,ABLIM3,MAPK1,TXNRD1,MLX,TARDBP,MOXD1,F8,EDNRA,CETN3,PSMD10,ALDH1A1,SELENBP1,MMP7,ABCF3,RAB11A,RUVBL1,PAK4,FGFR2,SLC25A10,FGA,DDC,FMN1,TAF7,SNX9,FBP1,ZNF655,SET,PPP3R1,PRKAR2B,RPS6,MYLK,VCP,RAC1,MBIP,HSPBP1,AKT3,HIATL1,C1orf87,LHX1,ZMYM3,NPM1,CORO2B,CIAO1,KLHL14,CYGB,RTN4,PDXK,ANXA13,NFKBIA,ALDH9A1,ACVR2B,C8orf37 |
| Liver cancer | 187 | 7629 | 51.65745856 | 1.225477554 | 0.000178 | 0.0704 | 0.000757 | 0.000176 | CRYM,KCNMB3,HOXA5,EFCAB2,STAT6,ZHX3,CCNB1,DRG1,PROSC,DNAJB5,AFP,STK33,FHL2,NFE2,RAB3B,STAU2,MRPS7,CALCOCO2,FST,NRAS,TYROBP,RPL10,ERAL1,APOE,COX4I1,CD47,UPP2,HSPA2,GULP1,TM4SF1,GALT,ELOVL1,LYPD1,GSTP1,RPS6KA2,FUBP1,ATAD2,FAM63A,TIMP1,PPP2R4,SLC39A9,RHOA,DLX5,RAP1GDS1,HPX,SGK2,NECAP2,CHD4,ST6GALNAC6,DOK1,PSMD14,IMPDH2,PANK1,XPR1,DHX9,GNB2L1,MRTO4,CYB5R1,CDC34,CLDN4,RAB39B,RRAGB,SFN,ANP32E,NHEJ1,NME1,STARD10,ADD1,CAMK4,TEX264,ZNF641,ANAPC11,DDX58,ARPC3,SPP1,CLGN,SULT1E1,PTS,RBKS,CCNH,AAK1,APEX1,CCT4,CCR10,BAG5,MAPK3,CDCP1,YWHAB,DBNL,TCOF1,GGT6,RPL14,C1QC,C1orf21,USP15,MORC4,MIPOL1,PTPN11,PRKRA,SMYD3,MCM5,C14orf119,DIABLO,PDLIM3,CKAP2,NAGK,MEIS2,DERL1,SRPK2,HSPB8,SERBP1,TK1,MARCKSL1,CYP2C8,PRPSAP2,MATK,C9,GTF2I,BCL7C,CDH26,TOM1,PCGF3,PLCB2,PAK6,ANXA6,USP14,TEAD3,COASY,RECQL5,ANXA11,ABLIM3,MAPK1,TXNRD1,MLX,TARDBP,HCRTR1,RASGEF1A,MOXD1,F8,EDNRA,CETN3,PSMD10,ALDH1A1,SELENBP1,MMP7,ABCF3,RAB11A,RUVBL1,PAK4,FGFR2,SLC25A10,FGA,DDC,FMN1,PECAM1,TAF7,SNX9,FBP1,ZNF655,SET,PPP3R1,PRKAR2B,RPS6,MYLK,VCP,RAC1,MBIP,HSPBP1,AKT3,NUDT18,HIATL1,C1orf87,LHX1,ZMYM3,NPM1,CORO2B,CIAO1,ARHGAP29,KLHL14,CYGB,RTN4,PDXK,ANXA13,NFKBIA,ALDH9A1,ACVR2B,C8orf37 |
| Nipple aspirate fluid | 30 | 735 | 8.287292818 | 2.108307033 | 0.000193 | 0.076548 | 0.000814 | 0.000189 | FST,APOE,HLA-DRB5,TM4SF1,GSTP1,CORO1A,TIMP1,STATH,RHOA,HPX,SFN,NME1,SPP1,HLA-DRB3,YWHAB,C1QC,HBG2,MARCKSL1,C9,SELENBP1,MMP7,RAB11A,S100A7A,FGA,FBP1,SET,VCP,RAC1,NPM1,PDXK |
| Skin cancer | 173 | 6970 | 47.79005525 | 1.241570179 | 0.000212 | 0.084052 | 0.000885 | 0.000205 | KCNMB3,HOXA5,EFCAB2,STAT6,ZHX3,CCNB1,DRG1,PROSC,DNAJB5,AFP,STK33,FHL2,NFE2,RAB3B,STAU2,MRPS7,CALCOCO2,FST,NRAS,TYROBP,RPL10,ERAL1,APOE,COX4I1,CD47,UPP2,HSPA2,GULP1,TM4SF1,GALT,ELOVL1,LYPD1,GSTP1,RPS6KA2,FUBP1,ATAD2,FAM63A,PPP2R4,SLC39A9,DLX5,RAP1GDS1,NECAP2,CHD4,ST6GALNAC6,DOK1,PSMD14,IMPDH2,PANK1,XPR1,DHX9,GNB2L1,MRTO4,CYB5R1,CDC34,CLDN4,RAB39B,RRAGB,SFN,ANP32E,NHEJ1,NME1,STARD10,ADD1,CAMK4,TEX264,ZNF641,ANAPC11,DDX58,ARPC3,SPP1,CLGN,SULT1E1,PTS,RBKS,CCNH,AAK1,APEX1,CCT4,CCR10,BAG5,MAPK3,CDCP1,YWHAB,DBNL,TCOF1,GGT6,RPL14,C1QC,C1orf21,USP15,MORC4,MIPOL1,PTPN11,PRKRA,SMYD3,MCM5,C14orf119,DIABLO,PDLIM3,CKAP2,NAGK,CPNE4,MEIS2,DERL1,SRPK2,HSPB8,SERBP1,TK1,MARCKSL1,PRPSAP2,MATK,C9,GTF2I,BCL7C,TOM1,PCGF3,PLCB2,PAK6,ANXA6,USP14,COASY,RECQL5,ANXA11,ABLIM3,MAPK1,TXNRD1,MLX,TARDBP,RASGEF1A,F8,EDNRA,SLA,CETN3,PSMD10,ALDH1A1,SELENBP1,ABCF3,RAB11A,RUVBL1,PAK4,FGFR2,SLC25A10,FGA,DDC,FMN1,TAF7,SNX9,ZNF655,SET,PPP3R1,PRKAR2B,RPS6,MYLK,VCP,RAC1,MBIP,HSPBP1,AKT3,NUDT18,C1orf87,LHX1,ZMYM3,NPM1,CORO2B,CIAO1,KLHL14,CYGB,PDXK,ANXA13,NFKBIA,ALDH9A1,ACVR2B,C8orf37 |
| Malignant melanoma | 181 | 7395 | 50 | 1.223954007 | 0.000288 | 0.114049 | 0.001188 | 0.000276 | CRYM,KCNMB3,HOXA5,EFCAB2,STAT6,ZHX3,CCNB1,DRG1,PROSC,DNAJB5,AFP,STK33,FHL2,NFE2,RAB3B,STAU2,MRPS7,CALCOCO2,FST,NRAS,TYROBP,RPL10,ERAL1,APOE,COX4I1,CD47,UPP2,HSPA2,GULP1,TM4SF1,GALT,ELOVL1,LYPD1,GSTP1,RPS6KA2,FUBP1,ATAD2,FAM63A,PPP2R4,SLC39A9,RHOA,DLX5,RAP1GDS1,SGK2,HLA-DOB,CHD4,ST6GALNAC6,DOK1,PSMD14,IMPDH2,PANK1,DHX9,GNB2L1,MRTO4,CYB5R1,CDC34,CLDN4,RAB39B,RRAGB,SFN,ANP32E,NHEJ1,NME1,STARD10,ADD1,CAMK4,TEX264,ANAPC11,DDX58,ARPC3,SPP1,CLGN,SULT1E1,PTS,RBKS,CCNH,AAK1,APEX1,CCT4,CCR10,BAG5,MAPK3,CDCP1,YWHAB,DBNL,TCOF1,GGT6,RPL14,C1QC,C1orf21,USP15,MORC4,MIPOL1,PTPN11,PRKRA,SMYD3,MCM5,C14orf119,DIABLO,PDLIM3,CKAP2,NAGK,CPNE4,MEIS2,DERL1,SRPK2,HSPB8,HBG2,SERBP1,TK1,MARCKSL1,CYP2C8,PRPSAP2,MATK,C9,GTF2I,BCL7C,CDH26,TOM1,PCGF3,PLCB2,PAK6,ANXA6,USP14,TEAD3,COASY,RECQL5,ANXA11,ABLIM3,MAPK1,TXNRD1,MLX,TARDBP,HCRTR1,RASGEF1A,F8,EDNRA,CETN3,PSMD10,ALDH1A1,EPB41L3,SELENBP1,MMP7,ABCF3,RAB11A,RUVBL1,PAK4,FGFR2,SLC25A10,FGA,DDC,FMN1,TAF7,SNX9,ZNF655,SET,PPP3R1,PRKAR2B,RPS6,MYLK,VCP,RAC1,MBIP,HSPBP1,AKT3,HIATL1,LHX1,ZMYM3,NPM1,CORO2B,CIAO1,ARHGAP29,KLHL14,CYGB,RTN4,PDXK,ANXA13,NFKBIA,ALDH9A1,ACVR2B,C8orf37 |
| hupo46_reference_cam | 37 | 1011 | 10.22099448 | 1.876950406 | 0.00031 | 0.12279 | 0.001266 | 0.000294 | CRYM,NRAS,CSRP1,APOE,COX4I1,CORO1A,QDPR,RAP1GDS1,ATG3,HPX,SYNPR,LCAT,NME1,ADD1,ARPC3,CCT4,MAPK3,YWHAB,C1QC,MARCKSL1,RAB8B,C9,NDUFA8,ANXA6,RPS13,USP14,MAPK1,SRI,ALDH1A1,SLC25A10,FGA,VCP,RAC1,NPM1,CORO2B,GOLT1B,RTN4 |
| Epididymis | 173 | 7025 | 47.79005525 | 1.23185143 | 0.000334 | 0.132149 | 0.001348 | 0.000313 | CRYM,KCNMB3,HOXA5,EFCAB2,STAT6,ZHX3,ADRB2,DRG1,PROSC,DNAJB5,AFP,STK33,FHL2,NFE2,STAU2,MRPS7,CALCOCO2,FST,NRAS,TYROBP,RPL10,ERAL1,APOE,COX4I1,CD47,UPP2,HSPA2,GULP1,TM4SF1,GALT,ELOVL1,LYPD1,NAPSA,GSTP1,RPS6KA2,FUBP1,ATAD2,FAM63A,PPP2R4,SLC39A9,RHOA,DLX5,RAP1GDS1,HPX,SGK2,CHD4,ST6GALNAC6,DOK1,PSMD14,IMPDH2,DHX9,GNB2L1,MRTO4,CYB5R1,CDC34,CLDN4,RAB39B,RRAGB,SFN,ANP32E,NHEJ1,NME1,STARD10,ADD1,CAMK4,TEX264,ANAPC11,DDX58,ARPC3,SPP1,CLGN,SULT1E1,PTS,RBKS,CCNH,AAK1,APEX1,CCR10,BAG5,MAPK3,CDCP1,YWHAB,DBNL,TCOF1,GGT6,RPL14,C1orf21,USP15,MORC4,MIPOL1,PTPN11,PRKRA,SMYD3,MCM5,C14orf119,DIABLO,CKAP2,NAGK,CPNE4,MEIS2,DERL1,SRPK2,HSPB8,SERBP1,MARCKSL1,CYP2C8,PRPSAP2,MATK,C9,GTF2I,BCL7C,CDH26,TOM1,PCGF3,PLCB2,ANXA6,USP14,TEAD3,COASY,RECQL5,ANXA11,ABLIM3,MAPK1,TXNRD1,MLX,TARDBP,F8,EDNRA,SLA,CETN3,PSMD10,ALDH1A1,EPB41L3,SELENBP1,ABCF3,RAB11A,RUVBL1,PAK4,FGFR2,SLC25A10,DDC,FMN1,TAF7,SNX9,FBP1,ZNF655,SET,PPP3R1,PRKAR2B,RPS6,MYLK,VCP,RAC1,MBIP,HSPBP1,PLEK,AKT3,HIATL1,LHX1,ZMYM3,NPM1,CORO2B,CIAO1,ARHGAP29,KLHL14,CYGB,RTN4,PDXK,ANXA13,NFKBIA,ALDH9A1,ACVR2B,C8orf37 |
| MDA468 | 29 | 725 | 8.011049724 | 2.068942292 | 0.000345 | 0.136487 | 0.001379 | 0.00032 | GSTP1,FUBP1,TIMP1,RHOA,IMPDH2,GNB2L1,SFN,NME1,HN1,APEX1,CCT4,TTC1,SERBP1,MARCKSL1,RPS13,USP14,ANXA11,MAPK1,TXNRD1,ALDH1A1,MMP7,TPD52L2,SET,VCP,CHORDC1,NPM1,PGM2,PDXK,RNPEP |
| Appendix | 171 | 6943 | 47.23756906 | 1.232091685 | 0.000376 | 0.148698 | 0.001487 | 0.000345 | CRYM,KCNMB3,HOXA5,EFCAB2,STAT6,ZHX3,CCNB1,DRG1,PROSC,DNAJB5,AFP,STK33,FHL2,NFE2,STAU2,MRPS7,CALCOCO2,FST,NRAS,TYROBP,RPL10,ERAL1,APOE,COX4I1,CD47,UPP2,HSPA2,GULP1,TM4SF1,GALT,ELOVL1,LYPD1,GSTP1,RPS6KA2,FUBP1,ATAD2,FAM63A,SLC39A9,RHOA,DLX5,RAP1GDS1,SGK2,NECAP2,HLA-DOB,CHD4,ST6GALNAC6,PSMD14,IMPDH2,PANK1,DHX9,GNB2L1,CYB5R1,CDC34,CLDN4,RAB39B,RRAGB,SFN,ANP32E,NHEJ1,NME1,STARD10,ADD1,CAMK4,TEX264,ZNF641,ANAPC11,DDX58,ARPC3,SPP1,CLGN,SULT1E1,RBKS,CCNH,AAK1,APEX1,CCR10,BAG5,MAPK3,CDCP1,YWHAB,DBNL,TCOF1,GGT6,RPL14,C1QC,C1orf21,MORC4,MIPOL1,PTPN11,PRKRA,SMYD3,MCM5,DIABLO,PDLIM3,CKAP2,NAGK,MEIS2,DERL1,HSPB8,SERBP1,TK1,MARCKSL1,CYP2C8,PRPSAP2,MATK,C9,GTF2I,BCL7C,CDH26,TOM1,PCGF3,PLCB2,PAK6,ANXA6,USP14,TEAD3,COASY,RECQL5,ANXA11,ABLIM3,MAPK1,TXNRD1,MLX,TARDBP,RASGEF1A,F8,EDNRA,CETN3,PSMD10,ALDH1A1,EPB41L3,SELENBP1,ABCF3,RAB11A,RUVBL1,PAK4,FGFR2,SLC25A10,FGA,DDC,FMN1,PECAM1,TAF7,SNX9,FBP1,ZNF655,SET,PPP3R1,RPS6,MYLK,VCP,MBIP,HSPBP1,PLEK,AKT3,NUDT18,HIATL1,LHX1,ZMYM3,NPM1,CORO2B,CIAO1,ARHGAP29,KLHL14,CYGB,PDXK,ANXA13,NFKBIA,ALDH9A1,ACVR2B,C8orf37 |
| Parathyroid gland | 150 | 5945 | 41.43646409 | 1.263459608 | 0.000392 | 0.155273 | 0.001537 | 0.000354 | CRYM,KCNMB3,HOXA5,EFCAB2,STAT6,ZHX3,ADRB2,DRG1,PROSC,DNAJB5,AFP,STK33,FHL2,NFE2,RAB3B,STAU2,MRPS7,CALCOCO2,FST,NRAS,TYROBP,RPL10,ERAL1,COX4I1,HSPA2,TM4SF1,GALT,ELOVL1,LYPD1,GSTP1,RPS6KA2,FUBP1,ATAD2,FAM63A,TIMP1,PPP2R4,SLC39A9,RHOA,DLX5,RAP1GDS1,NECAP2,CHD4,ST6GALNAC6,PSMD14,IMPDH2,DHX9,GNB2L1,CYB5R1,CDC34,CLDN4,RAB39B,PTN,RRAGB,SFN,ANP32E,NHEJ1,NME1,STARD10,ADD1,CAMK4,TEX264,ZNF641,ANAPC11,DDX58,SPP1,SULT1E1,PTS,RBKS,CCNH,AAK1,APEX1,CCR10,BAG5,MAPK3,CDCP1,YWHAB,DBNL,TCOF1,GGT6,RPL14,C1QC,C1orf21,USP15,MORC4,MIPOL1,PTPN11,C14orf119,DIABLO,CKAP2,MEIS2,DERL1,SRPK2,HSPB8,SERBP1,MARCKSL1,PRPSAP2,MATK,GTF2I,BCL7C,CDH26,TOM1,PCGF3,PLCB2,ANXA6,USP14,TEAD3,COASY,ANXA11,ABLIM3,MAPK1,TXNRD1,MLX,TARDBP,HCRTR1,RASGEF1A,F8,EDNRA,CETN3,PSMD10,EPB41L3,ABCF3,RAB11A,RUVBL1,PAK4,FGFR2,SLC25A10,FMN1,TAF7,SNX9,FBP1,SET,RPS6,MYLK,VCP,RAC1,HSPBP1,AKT3,LHX1,ZMYM3,NPM1,CORO2B,CIAO1,KLHL14,CYGB,PDXK,ANXA13,NFKBIA,ALDH9A1,ACVR2B,C8orf37 |
| Stomach | 192 | 7973 | 53.03867403 | 1.203756927 | 0.000393 | 0.155496 | 0.001524 | 0.000354 | KCNMB3,HOXA5,EFCAB2,STAT6,ZHX3,CCNB1,ADRB2,DRG1,PROSC,DNAJB5,AFP,STK33,FHL2,NFE2,RAB3B,STAU2,MRPS7,CALCOCO2,FST,NRAS,TYROBP,RPL10,ERAL1,APOE,COX4I1,CD47,UPP2,HSPA2,GULP1,TM4SF1,GALT,ELOVL1,LYPD1,GSTP1,RPS6KA2,FUBP1,ATAD2,FAM63A,TIMP1,OLR1,PPP2R4,SLC39A9,RHOA,DLX5,RAP1GDS1,HPX,SGK2,NECAP2,CHD4,ST6GALNAC6,DOK1,PSMD14,IMPDH2,PANK1,XPR1,DHX9,GNB2L1,MRTO4,BCAS2,CYB5R1,CDC34,CLDN4,RAB39B,RRAGB,SFN,ANP32E,FN3K,NHEJ1,NME1,STARD10,ADD1,CAMK4,TEX264,ZNF641,ANAPC11,DDX58,ARPC3,SPP1,SULT1E1,KLF11,PTS,RBKS,CCNH,AAK1,ARHGDIG,APEX1,CCT4,CCR10,BAG5,MAPK3,CDCP1,YWHAB,DBNL,TCOF1,GGT6,RPL14,C1QC,C1orf21,USP15,MORC4,MIPOL1,PTPN11,PRKRA,SMYD3,MCM5,C14orf119,DIABLO,PDLIM3,CKAP2,NAGK,MEIS2,DERL1,HSPB8,SERBP1,TK1,MARCKSL1,CYP2C8,PRPSAP2,MATK,C9,GTF2I,BCL7C,CDH26,NDUFA8,TOM1,PCGF3,PLCB2,PAK6,ANXA6,USP14,TEAD3,COASY,RECQL5,ANXA11,ABLIM3,MAPK1,TXNRD1,MLX,TARDBP,RASGEF1A,MOXD1,F8,EDNRA,SLA,CETN3,PSMD10,LEPROTL1,ALDH1A1,SELENBP1,MMP7,ABCF3,RAB11A,RUVBL1,SNX1,PAK4,FGFR2,SLC25A10,FGA,DDC,FMN1,TAF7,SNX9,FBP1,ZNF655,SET,PPP3R1,PRKAR2B,RPS6,MYLK,VCP,RAC1,MBIP,HSPBP1,AKT3,NUDT18,HIATL1,C1orf87,LHX1,ZMYM3,NPM1,CORO2B,CIAO1,ARHGAP29,KLHL14,CYGB,RTN4,PDXK,ANXA13,NFKBIA,ALDH9A1,ACVR2B,C8orf37 |
| Jurkat A3 | 111 | 4147 | 30.66298343 | 1.344096875 | 0.000414 | 0.16405 | 0.001593 | 0.000366 | CRYM,DOHH,CD84,DRG1,STAU2,MRPS7,CALCOCO2,RPL10,ERAL1,OGFOD2,GPSM3,HLA-DRB5,CD47,LRRC20,CKS2,ATAD2,CAPRIN2,RPS7,PPP2R4,QDPR,ROPN1L,RHOA,RAP1GDS1,ATG3,NECAP2,CHD4,MRPS25,PSMD14,XPR1,DHX9,MRTO4,BCAS2,CYB5R1,ARL2BP,EPS8L1,RRAGB,CTNNAL1,FBXL18,NME1,NMRAL1,ADD1,CAMK4,RPS15,DCK,DHFR,HN1,DDX58,NOSIP,AAK1,CCT4,MRPL53,ABLIM1,ARIH2,BAG5,MAPK3,MYOT,ARF5,DBNL,TCOF1,RPL14,TTC1,UGDH,ASF1A,ENOPH1,ZFYVE19,PRKRA,MCM5,QTRT1,PI16,CKAP2,NAGK,MCTS1,HIGD2A,RPL22,SRPK2,TK1,MARCKSL1,SPC25,STUB1,PRPSAP2,C9,TOM1,PDS5B,MAPK1,MLX,F8,SELENBP1,ABCF3,SNX1,PAK4,SLC25A10,PAF1,SET,PRKAR2B,RPS6,VCP,COG3,HSPBP1,NUDT18,CHORDC1,HIATL1,GBE1,NPM1,NRBF2,PGM2,RTN4,OBFC1,PDXK,RNPEP,C8orf37,UBE2O |
| Thyroid gland | 177 | 7246 | 48.89502762 | 1.221702434 | 0.000415 | 0.1642 | 0.001579 | 0.000366 | CRYM,KCNMB3,HOXA5,EFCAB2,STAT6,ZHX3,ADRB2,DRG1,PROSC,DNAJB5,AFP,STK33,FHL2,NFE2,STAU2,MRPS7,CALCOCO2,FST,NRAS,RPL10,ERAL1,APOE,COX4I1,HSPA2,GULP1,TM4SF1,GALT,ELOVL1,LYPD1,GSTP1,RPS6KA2,FUBP1,ATAD2,FAM63A,OLR1,PPP2R4,SLC39A9,ADRBK1,RHOA,LAYN,DLX5,RAP1GDS1,SGK2,NECAP2,HLA-DOB,SPAG16,CHD4,ST6GALNAC6,DOK1,PSMD14,IMPDH2,PANK1,XPR1,DHX9,GNB2L1,MRTO4,BCAS2,CYB5R1,CDC34,CLDN4,RAB39B,RRAGB,SFN,NHEJ1,NME1,STARD10,ADD1,CAMK4,TEX264,ZNF641,ANAPC11,DDX58,ARPC3,SPP1,CLGN,SULT1E1,KLF11,PTS,RBKS,AAK1,APEX1,CCR10,BAG5,MAPK3,CDCP1,YWHAB,DBNL,TCOF1,GGT6,RPL14,C1QC,C1orf21,USP15,MORC4,MIPOL1,PTPN11,PRKRA,SMYD3,C14orf119,DIABLO,PDLIM3,PI16,CKAP2,NAGK,CPNE4,MEIS2,DERL1,SRPK2,HSPB8,SERBP1,MARCKSL1,CYP2C8,PRPSAP2,MATK,C9,GTF2I,BCL7C,CDH26,NDUFA8,TOM1,FBXL3,PCGF3,PLCB2,PAK6,ANXA6,USP14,TEAD3,COASY,ANXA11,ABLIM3,MAPK1,TXNRD1,MLX,TARDBP,F8,EDNRA,SLA,CETN3,PSMD10,LEPROTL1,ALDH1A1,SELENBP1,ABCF3,RAB11A,RUVBL1,SNX1,PAK4,FGFR2,SLC25A10,DDC,FMN1,TAF7,SNX9,FBP1,ZNF655,SET,RPS6,MYLK,VCP,MBIP,HSPBP1,AKT3,HIATL1,LHX1,ZMYM3,NPM1,CORO2B,CIAO1,ARHGAP29,KLHL14,CYGB,PDXK,ANXA13,NFKBIA,ALDH9A1,ACVR2B,C8orf37 |
| Soft tissue | 170 | 6908 | 46.96132597 | 1.231143897 | 0.000418 | 0.165386 | 0.001575 | 0.000366 | KCNMB3,HOXA5,EFCAB2,STAT6,ZHX3,DRG1,PROSC,DNAJB5,AFP,STK33,FHL2,NFE2,STAU2,MRPS7,CALCOCO2,FST,NRAS,TYROBP,RPL10,ERAL1,APOE,COX4I1,CD47,UPP2,HSPA2,GULP1,TM4SF1,GALT,ELOVL1,LYPD1,GSTP1,RPS6KA2,FUBP1,ATAD2,FAM63A,PPP2R4,SLC39A9,RHOA,DLX5,RAP1GDS1,HPX,SGK2,NECAP2,CHD4,ST6GALNAC6,DOK1,PSMD14,IMPDH2,PANK1,XPR1,DHX9,GNB2L1,MRTO4,CYB5R1,CDC34,CLDN4,SFN,ANP32E,NHEJ1,NME1,STARD10,ADD1,CAMK4,ZNF641,ANAPC11,DDX58,ARPC3,SPP1,CLGN,SULT1E1,PTS,AAK1,APEX1,CCR10,BAG5,MAPK3,CDCP1,YWHAB,DBNL,TCOF1,GGT6,RPL14,C1QC,C1orf21,USP15,MORC4,MIPOL1,PTPN11,PRKRA,SMYD3,C14orf119,DIABLO,PDLIM3,CKAP2,NAGK,CPNE4,MEIS2,DERL1,SRPK2,HSPB8,SERBP1,MARCKSL1,CYP2C8,PRPSAP2,MATK,C9,GTF2I,BCL7C,CDH26,TOM1,PCGF3,PLCB2,PAK6,ANXA6,USP14,TEAD3,COASY,RECQL5,ANXA11,ABLIM3,MAPK1,TXNRD1,MLX,TARDBP,RASGEF1A,F8,EDNRA,SLA,CETN3,PSMD10,ALDH1A1,EPB41L3,SELENBP1,ABCF3,RAB11A,RUVBL1,PAK4,FGFR2,SLC25A10,FGA,DDC,FMN1,TAF7,SNX9,ZNF655,SET,PRKAR2B,RPS6,MYLK,VCP,RAC1,MBIP,HSPBP1,AKT3,HIATL1,LHX1,ZMYM3,NPM1,CORO2B,CIAO1,ARHGAP29,KLHL14,CYGB,RTN4,PDXK,ANXA13,NFKBIA,ALDH9A1,ACVR2B,C8orf37 |
| Malignant carcinoid | 172 | 7020 | 47.51381215 | 1.225654178 | 0.000472 | 0.186874 | 0.001763 | 0.000409 | CRYM,KCNMB3,HOXA5,EFCAB2,STAT6,ZHX3,CCNB1,ADRB2,DRG1,PROSC,DNAJB5,AFP,STK33,FHL2,NFE2,RAB3B,STAU2,MRPS7,CALCOCO2,FST,NRAS,TYROBP,RPL10,ERAL1,APOE,COX4I1,CD47,HSPA2,GULP1,TM4SF1,GALT,ELOVL1,LYPD1,GSTP1,RPS6KA2,FUBP1,ATAD2,FAM63A,TIMP1,PPP2R4,SLC39A9,RHOA,DLX5,RAP1GDS1,HPX,SGK2,CHD4,ST6GALNAC6,DOK1,PSMD14,IMPDH2,PANK1,DHX9,GNB2L1,CYB5R1,CDC34,CLDN4,RAB39B,RRAGB,ANP32E,NHEJ1,NME1,STARD10,ADD1,CAMK4,ANAPC11,DDX58,ARPC3,SPP1,CLGN,PTS,RBKS,CCNH,AAK1,APEX1,CCR10,BAG5,MAPK3,CDCP1,YWHAB,DBNL,TCOF1,GGT6,RPL14,C1QC,C1orf21,USP15,MORC4,MIPOL1,PTPN11,PRKRA,SMYD3,DIABLO,CKAP2,NAGK,MEIS2,DERL1,SRPK2,HSPB8,HBG2,SERBP1,TK1,MARCKSL1,PRPSAP2,MATK,C9,GTF2I,BCL7C,CDH26,TOM1,PCGF3,PLCB2,ANXA6,USP14,TEAD3,COASY,RECQL5,ANXA11,ABLIM3,MAPK1,TXNRD1,MLX,TARDBP,HCRTR1,RASGEF1A,MOXD1,F8,EDNRA,CETN3,PSMD10,ALDH1A1,SELENBP1,MMP7,ABCF3,RAB11A,RUVBL1,PAK4,FGFR2,SLC25A10,FGA,DDC,FMN1,TAF7,SNX9,ZNF655,SET,PPP3R1,PRKAR2B,RPS6,MYLK,VCP,RAC1,MBIP,HSPBP1,AKT3,NUDT18,HIATL1,LHX1,ZMYM3,NPM1,CORO2B,CIAO1,ARHGAP29,KLHL14,CYGB,RTN4,PDXK,ANXA13,NFKBIA,ALDH9A1,ACVR2B,C8orf37 |
| Breast | 158 | 6357 | 43.64640884 | 1.244087667 | 0.00051 | 0.201866 | 0.001887 | 0.000438 | CRYM,KCNMB3,HOXA5,EFCAB2,STAT6,ZHX3,CCNB1,ADRB2,DRG1,PROSC,DNAJB5,AFP,STK33,FHL2,NFE2,STAU2,MRPS7,CALCOCO2,FST,NRAS,TYROBP,RPL10,ERAL1,APOE,COX4I1,UPP2,HSPA2,GULP1,TM4SF1,GALT,ELOVL1,GSTP1,RPS6KA2,FUBP1,ATAD2,FAM63A,PPP2R4,SLC39A9,RHOA,DLX5,RAP1GDS1,NECAP2,CHD4,ST6GALNAC6,DOK1,PSMD14,IMPDH2,DHX9,GNB2L1,MRTO4,CYB5R1,CDC34,CLDN4,RAB39B,RRAGB,SFN,NHEJ1,NME1,STARD10,CAMK4,ANAPC11,DDX58,ARPC3,SPP1,CLGN,AAK1,APEX1,CCT4,CCR10,BAG5,MAPK3,CDCP1,YWHAB,DBNL,TCOF1,GGT6,RPL14,C1orf21,USP15,MORC4,MIPOL1,PTPN11,PRKRA,SMYD3,C14orf119,DIABLO,PDLIM3,CKAP2,NAGK,MEIS2,DERL1,SRPK2,HSPB8,SERBP1,TK1,MARCKSL1,PRPSAP2,MATK,C9,GTF2I,BCL7C,CDH26,TOM1,PCGF3,PLCB2,ANXA6,USP14,TEAD3,COASY,ANXA11,ABLIM3,MAPK1,TXNRD1,MLX,TARDBP,F8,EDNRA,CETN3,PSMD10,EPB41L3,SELENBP1,ABCF3,RAB11A,RUVBL1,PAK4,FGFR2,SLC25A10,DDC,FMN1,TAF7,SNX9,FBP1,ZNF655,PPP3R1,PRKAR2B,RPS6,MYLK,VCP,RAC1,MBIP,HSPBP1,AKT3,NUDT18,HIATL1,C1orf87,LHX1,ZMYM3,NPM1,CORO2B,CIAO1,KLHL14,RTN4,PDXK,ANXA13,NFKBIA,ALDH9A1,ACVR2B,C8orf37 |
| Colon | 199 | 8358 | 54.97237569 | 1.189910658 | 0.000529 | 0.209487 | 0.00194 | 0.00045 | CRYM,KCNMB3,HOXA5,EFCAB2,STAT6,ZHX3,CCNB1,ADRB2,DRG1,PROSC,DNAJB5,AFP,STK33,FHL2,NFE2,RAB3B,STAU2,MRPS7,DAZAP2,CALCOCO2,FST,NRAS,TYROBP,RPL10,CSRP1,ERAL1,COX4I1,UPP2,HSPA2,GULP1,GALT,SP110,ELOVL1,LYPD1,GSTP1,RPS6KA2,CYBB,FUBP1,ATAD2,FAM63A,OLR1,PPP2R4,SLC39A9,RHOA,DLX5,RAP1GDS1,ATG3,SGK2,NECAP2,HLA-DOB,CHD4,ST6GALNAC6,IMPDH2,PANK1,DHX9,GNB2L1,MRTO4,BCAS2,CYB5R1,CDC34,CLDN4,RAB39B,RRAGB,CTNNAL1,SFN,ANP32E,NHEJ1,NME1,STARD10,ADD1,CAMK4,TEX264,ZNF641,HN1,ANAPC11,DDX58,ARPC3,SPP1,SULT1E1,EIF4EBP3,RBKS,CCNH,AAK1,ARHGDIG,APEX1,CCT4,CCR10,ABLIM1,BAG5,MAPK3,TIRAP,CDCP1,YWHAB,DBNL,TCOF1,GGT6,RPL14,C1QC,C1orf21,USP15,UGDH,ASF1A,MIPOL1,PTPN11,PRKRA,SMYD3,MCM5,DIABLO,PI16,CKAP2,NAGK,CPNE4,MEIS2,DERL1,HSPB8,HBG2,SERBP1,TK1,MARCKSL1,CYP2C8,PRPSAP2,MATK,C9,GTF2I,BCL7C,PYCR1,CDH26,NDUFA8,TOM1,FBXL3,PCGF3,PLCB2,PAK6,ANXA6,USP14,CLP1,TEAD3,COASY,RECQL5,ANXA11,RNF11,ABLIM3,MAPK1,TXNRD1,CCL5,MLX,TARDBP,RASGEF1A,F8,EDNRA,SLA,CETN3,PSMD10,LEPROTL1,EPB41L3,SELENBP1,MMP7,ABCF3,RAB11A,SNX1,PAK4,FGFR2,SLC25A10,DDC,FMN1,TAF7,HSPBAP1,SNX9,ZNF655,SET,PPP3R1,PRKAR2B,RPS6,MYLK,VCP,RAC1,COG3,HSPBP1,AKT3,NUDT18,HIATL1,C1orf87,LHX1,FABP5,SSPN,ZMYM3,NPM1,CORO2B,CIAO1,ARHGAP29,KLHL14,CYGB,RTN4,PDXK,ANXA13,NFKBIA,ALDH9A1,ACVR2B,UBE2O |
| Cerebral cortex | 172 | 7038 | 47.51381215 | 1.222520069 | 0.000544 | 0.215227 | 0.001975 | 0.000454 | CRYM,KCNMB3,HOXA5,EFCAB2,STAT6,ZHX3,DRG1,PROSC,DNAJB5,AFP,STK33,FHL2,NFE2,STAU2,MRPS7,CALCOCO2,FST,NRAS,TYROBP,RPL10,ERAL1,APOE,COX4I1,UPP2,HSPA2,GULP1,GALT,ELOVL1,LYPD1,GSTP1,RPS6KA2,FUBP1,ATAD2,FAM63A,PPP2R4,SLC39A9,RHOA,DLX5,RAP1GDS1,HPX,SGK2,NECAP2,CHD4,DOK1,PSMD14,IMPDH2,PANK1,XPR1,DHX9,GNB2L1,MRTO4,CYB5R1,CDC34,CLDN4,VSNL1,RRAGB,SFN,ANP32E,NHEJ1,NME1,STARD10,CAMK4,TEX264,ZNF641,ANAPC11,DDX58,ARPC3,CLGN,SULT1E1,PTS,RBKS,AAK1,ARHGDIG,APEX1,CCT4,CCR10,BAG5,MAPK3,CDCP1,YWHAB,DBNL,TCOF1,GGT6,RPL14,C1QC,C1orf21,USP15,MORC4,MIPOL1,PTPN11,PRKRA,SMYD3,MCM5,C14orf119,DIABLO,CKAP2,GABRA5,NAGK,CPNE4,MEIS2,DERL1,SRPK2,HSPB8,SERBP1,MARCKSL1,MATK,GTF2I,BCL7C,CDH26,TOM1,FBXL3,PCGF3,PLCB2,PAK6,ANXA6,USP14,COASY,RECQL5,ANXA11,ABLIM3,MAPK1,TXNRD1,MLX,TARDBP,RASGEF1A,F8,EDNRA,SLA,CETN3,PSMD10,EPB41L3,SELENBP1,MMP7,ABCF3,RAB11A,RUVBL1,PAK4,FGFR2,SLC25A10,DDC,FMN1,TAF7,SNX9,FBP1,ZNF655,SET,PPP3R1,PRKAR2B,RPS6,MYLK,VCP,RAC1,MBIP,HSPBP1,PLEK,AKT3,NUDT18,HIATL1,C1orf87,LHX1,ZMYM3,NPM1,CORO2B,CIAO1,KLHL14,CYGB,RTN4,PDXK,ANXA13,NFKBIA,ALDH9A1,C8orf37 |
| Nasopharynx | 168 | 6845 | 46.40883978 | 1.227962394 | 0.000544 | 0.215602 | 0.00196 | 0.000454 | CRYM,KCNMB3,HOXA5,EFCAB2,STAT6,ZHX3,CCNB1,ADRB2,PROSC,DNAJB5,AFP,STK33,FHL2,NFE2,STAU2,MRPS7,CALCOCO2,FST,NRAS,TYROBP,RPL10,ERAL1,COX4I1,UPP2,HSPA2,GULP1,TM4SF1,GALT,ELOVL1,LYPD1,GSTP1,RPS6KA2,FUBP1,ATAD2,FAM63A,PPP2R4,SLC39A9,RHOA,DLX5,RAP1GDS1,NECAP2,CHD4,ST6GALNAC6,DOK1,PSMD14,IMPDH2,DHX9,GNB2L1,MRTO4,CYB5R1,CDC34,CLDN4,RRAGB,SFN,ANP32E,NHEJ1,NME1,STARD10,ADD1,CAMK4,TEX264,ZNF641,ANAPC11,DDX58,ARPC3,SPP1,CLGN,SULT1E1,RBKS,AAK1,APEX1,CCT4,CCR10,BAG5,MAPK3,CDCP1,YWHAB,DBNL,TCOF1,GGT6,RPL14,C1QC,C1orf21,USP15,MORC4,MIPOL1,PTPN11,PRKRA,SMYD3,MCM5,C14orf119,DIABLO,PDLIM3,CKAP2,NAGK,MEIS2,DERL1,SRPK2,HSPB8,SERBP1,TK1,MARCKSL1,CYP2C8,PRPSAP2,MATK,C9,GTF2I,BCL7C,CDH26,TOM1,PCGF3,PLCB2,PAK6,ANXA6,TEAD3,COASY,RECQL5,ANXA11,ABLIM3,MAPK1,TXNRD1,MLX,TARDBP,RASGEF1A,F8,EDNRA,SLA,CETN3,PSMD10,ALDH1A1,SELENBP1,ABCF3,RAB11A,RUVBL1,PAK4,FGFR2,SLC25A10,FGA,DDC,FMN1,TAF7,SNX9,ZNF655,SET,PPP3R1,PRKAR2B,RPS6,MYLK,VCP,RAC1,HSPBP1,AKT3,NUDT18,HIATL1,C1orf87,NPM1,CORO2B,CIAO1,ARHGAP29,KLHL14,CYGB,RTN4,PDXK,ANXA13,NFKBIA,ALDH9A1,ACVR2B,C8orf37 |
| Spleen | 188 | 7820 | 51.93370166 | 1.201901463 | 0.000551 | 0.218084 | 0.001965 | 0.000454 | KCNMB3,EFCAB2,STAT6,CD84,ZHX3,CCNB1,ADRB2,DRG1,PROSC,DNAJB5,SRPX2,AFP,STK33,FHL2,NFE2,RAB3B,STAU2,MRPS7,DAZAP2,FST,NRAS,TYROBP,RPL10,ERAL1,APOE,COX4I1,CD47,UPP2,HSPA2,GULP1,GALT,SP110,ELOVL1,NAPSA,GSTP1,RPS6KA2,CORO1A,FUBP1,ATAD2,CAPRIN2,OLR1,SLC39A9,RHOA,LAYN,DLX5,RAP1GDS1,ATG3,SGK2,HLA-DOB,CHD4,DOK1,MRPS25,PSMD14,IMPDH2,XPR1,DHX9,GNB2L1,BCAS2,CYB5R1,CDC34,RAB39B,LCAT,RRAGB,ANP32E,FN3K,NHEJ1,NME1,STARD10,CAMK4,DCK,TMEM185A,ANAPC11,DDX58,ARPC3,SPP1,SULT1E1,SH2D1B,PTS,RBKS,AAK1,APEX1,FNIP1,CCR10,ABLIM1,BAG5,MAPK3,TIRAP,CDCP1,YWHAB,DBNL,TCOF1,GGT6,RPL14,C1QC,C1orf21,USP15,UGDH,ASF1A,MIPOL1,PTPN11,PRKRA,SMYD3,MCM5,C14orf119,DIABLO,PDLIM3,CKAP2,NAGK,CPNE4,MEIS2,DERL1,SRPK2,HSPB8,SERBP1,MARCKSL1,CYP2C8,MATK,C9,GTF2I,BCL7C,NDUFA8,DAPP1,TOM1,FBXL3,PCGF3,PLCB2,ANXA6,USP14,TEAD3,COASY,RECQL5,ANXA11,RNF11,ABLIM3,MAPK1,TXNRD1,MLX,TARDBP,MOXD1,EDNRA,SLA,CETN3,PSMD10,LRFN1,LEPROTL1,SELENBP1,ABCF3,RAB11A,RUVBL1,SNX1,PAK4,FGFR2,DDC,FMN1,PECAM1,TAF7,HSPBAP1,SNX9,ZNF655,LST1,SET,PPP3R1,RPS6,MYLK,VCP,RAC1,COG3,HSPBP1,AKT3,HIATL1,GFM2,LHX1,FABP5,SSPN,ZMYM3,NPM1,CORO2B,CIAO1,ARHGAP29,KLHL14,RTN4,PDXK,ANXA13,NFKBIA,DGUOK,ALDH9A1,C8orf37,UBE2O |
| MCF10A | 26 | 640 | 7.182320442 | 2.110805767 | 0.000553 | 0.218916 | 0.001955 | 0.000454 | CSRP1,HPCAL1,CARHSP1,GSTP1,FUBP1,TIMP1,NECAP2,GNB2L1,SFN,ANP32E,NME1,HN1,DBNL,TCOF1,TTC1,DIABLO,SERBP1,RPS13,SRI,CCDC102B,TPD52L2,SET,VCP,FABP5,NPM1,RNPEP |
| Urinary bladder | 171 | 6996 | 47.23756906 | 1.222759325 | 0.000571 | 0.2262 | 0.002002 | 0.000465 | CRYM,KCNMB3,HOXA5,EFCAB2,STAT6,ZHX3,CCNB1,ADRB2,DRG1,PROSC,DNAJB5,AFP,STK33,FHL2,NFE2,STAU2,MRPS7,CALCOCO2,FST,NRAS,TYROBP,RPL10,ERAL1,APOE,COX4I1,CD47,UPP2,HSPA2,GULP1,TM4SF1,GALT,ELOVL1,LYPD1,GSTP1,RPS6KA2,FUBP1,ATAD2,FAM63A,OLR1,PPP2R4,SLC39A9,RHOA,DLX5,RAP1GDS1,SGK2,NECAP2,CHD4,ST6GALNAC6,DOK1,PSMD14,IMPDH2,DHX9,GNB2L1,MRTO4,CYB5R1,CDC34,CLDN4,RAB39B,RRAGB,SFN,ANP32E,NHEJ1,NME1,STARD10,ADD1,CAMK4,TEX264,ZNF641,ANAPC11,DDX58,ARPC3,SPP1,CLGN,SULT1E1,RBKS,CCNH,AAK1,APEX1,CCT4,CCR10,BAG5,MAPK3,CDCP1,YWHAB,DBNL,TCOF1,GGT6,RPL14,C1QC,C1orf21,USP15,MORC4,MIPOL1,PTPN11,PRKRA,SMYD3,MCM5,C14orf119,DIABLO,PDLIM3,CKAP2,NAGK,MEIS2,DERL1,SRPK2,HSPB8,SERBP1,MARCKSL1,PRPSAP2,MATK,C9,GTF2I,BCL7C,CDH26,TOM1,PCGF3,PLCB2,PAK6,USP14,TEAD3,COASY,RECQL5,ANXA11,ABLIM3,MAPK1,TXNRD1,MLX,TARDBP,F8,EDNRA,CETN3,PSMD10,SELENBP1,MMP7,ABCF3,RAB11A,RUVBL1,PAK4,FGFR2,SLC25A10,FGA,DDC,FMN1,TAF7,SNX9,FBP1,ZNF655,SET,PPP3R1,RPS6,MYLK,VCP,RAC1,MBIP,HSPBP1,AKT3,NUDT18,LHX1,ZMYM3,NPM1,CORO2B,CIAO1,ARHGAP29,KLHL14,CYGB,PDXK,ANXA13,NFKBIA,ALDH9A1,ACVR2B,C8orf37 |
| Fallopian tubes | 163 | 6614 | 45.02762431 | 1.233298662 | 0.000587 | 0.232376 | 0.002038 | 0.000473 | KCNMB3,HOXA5,EFCAB2,STAT6,ZHX3,CCNB1,ADRB2,DRG1,PROSC,DNAJB5,AFP,STK33,FHL2,NFE2,RAB3B,STAU2,MRPS7,CALCOCO2,FST,NRAS,RPL10,ERAL1,COX4I1,CD47,UPP2,HSPA2,GULP1,TM4SF1,GALT,ELOVL1,LYPD1,GSTP1,RPS6KA2,FUBP1,ATAD2,FAM63A,PPP2R4,SLC39A9,RHOA,DLX5,RAP1GDS1,SGK2,CHD4,DOK1,IMPDH2,PANK1,DHX9,GNB2L1,MRTO4,CYB5R1,CDC34,CLDN4,RAB39B,RRAGB,ANP32E,NHEJ1,NME1,STARD10,ADD1,CAMK4,ZNF641,ANAPC11,DDX58,ARPC3,SPP1,CLGN,RBKS,CCNH,AAK1,APEX1,CCT4,BAG5,MAPK3,CDCP1,YWHAB,DBNL,TCOF1,GGT6,RPL14,C1QC,C1orf21,USP15,MORC4,MIPOL1,PTPN11,PRKRA,SMYD3,MCM5,DIABLO,PDLIM3,CKAP2,CPNE4,MEIS2,DERL1,SRPK2,HSPB8,SERBP1,TK1,PRPSAP2,MATK,C9,GTF2I,BCL7C,TOM1,PCGF3,PLCB2,PAK6,ANXA6,USP14,TEAD3,COASY,RECQL5,ANXA11,ABLIM3,MAPK1,TXNRD1,MLX,TARDBP,RASGEF1A,MOXD1,F8,EDNRA,CETN3,PSMD10,EPB41L3,SELENBP1,ABCF3,RAB11A,RUVBL1,PAK4,SLC25A10,FGA,DDC,FMN1,TAF7,SNX9,ZNF655,SET,PPP3R1,PRKAR2B,RPS6,MYLK,VCP,RAC1,MBIP,HSPBP1,AKT3,C1orf87,LHX1,ZMYM3,NPM1,CORO2B,CIAO1,ARHGAP29,KLHL14,CYGB,RTN4,PDXK,ANXA13,NFKBIA,ALDH9A1,ACVR2B,C8orf37 |
| Adducin | 21 | 479 | 5.801104972 | 2.301292306 | 0.00072 | 0.285017 | 0.002478 | 0.000574 | MRPS7,GSTP1,CORO1A,FUBP1,ATAD2,CHD4,GNB2L1,ANP32E,ADD1,APEX1,CCT4,MCM5,GTF2I,ANXA6,ANXA11,CCL5,RUVBL1,RPS6,VCP,NPM1,PEX19 |
| BJ635 | 11 | 172 | 3.038674033 | 3.512141372 | 0.000724 | 0.286878 | 0.002473 | 0.000574 | CD84,TYROBP,DOK1,MAPK3,CDCP1,DBNL,PTPN11,MAPK1,HCRTR1,PECAM1,LST1 |
| YTS | 57 | 1856 | 15.74585635 | 1.557882527 | 0.000739 | 0.292465 | 0.0025 | 0.00058 | DRG1,STAU2,CALCOCO2,NRAS,RPL10,KRR1,APOE,HLA-DRB5,COX4I1,CD47,HSPA2,ELOVL1,FUBP3,CORO1A,RPS7,ADRBK1,RHOA,CHD4,SLC5A6,IMPDH2,XPR1,DHX9,CYB5R1,RAB39B,SEPT1,NME1,RPS15,TEX264,ARPC3,HLA-DRB3,EIF4EBP3,CCT4,BAG5,YWHAB,RPL14,PRKRA,MCM5,DERL1,SERBP1,TK1,RAB8B,GTF2I,ANXA6,RPS13,USP14,ANXA11,TARDBP,ABCF3,RAB11A,RUVBL1,RPS6,RAC1,PLEK,SRP54,NPM1,GOLT1B,RTN4 |
| Esophagus | 149 | 5981 | 41.16022099 | 1.247553175 | 0.000773 | 0.306124 | 0.002594 | 0.000602 | KCNMB3,HOXA5,EFCAB2,STAT6,ZHX3,CCNB1,DRG1,PROSC,DNAJB5,AFP,STK33,FHL2,NFE2,RAB3B,STAU2,MRPS7,CALCOCO2,FST,NRAS,TYROBP,RPL10,ERAL1,APOE,COX4I1,UPP2,HSPA2,GULP1,TM4SF1,GALT,ELOVL1,LYPD1,GSTP1,RPS6KA2,FUBP1,ATAD2,FAM63A,PPP2R4,SLC39A9,DLX5,RAP1GDS1,NECAP2,CHD4,DOK1,PSMD14,IMPDH2,DHX9,GNB2L1,MRTO4,CYB5R1,CDC34,CLDN4,SFN,ANP32E,NHEJ1,NME1,STARD10,ADD1,TEX264,ZNF641,ANAPC11,SPP1,RBKS,AAK1,APEX1,CCT4,CCR10,BAG5,MAPK3,CDCP1,YWHAB,DBNL,TCOF1,GGT6,RPL14,C1orf21,MORC4,MIPOL1,PTPN11,SMYD3,MCM5,C14orf119,DIABLO,PDLIM3,CKAP2,NAGK,CPNE4,MEIS2,DERL1,SRPK2,HSPB8,SERBP1,TK1,PRPSAP2,MATK,C9,GTF2I,BCL7C,CDH26,TOM1,PCGF3,PLCB2,PAK6,USP14,TEAD3,COASY,RECQL5,ANXA11,ABLIM3,MAPK1,TXNRD1,MLX,TARDBP,F8,EDNRA,CETN3,PSMD10,ABCF3,RUVBL1,PAK4,FGFR2,SLC25A10,FGA,DDC,FMN1,TAF7,SNX9,ZNF655,SET,RPS6,MYLK,VCP,MBIP,HSPBP1,AKT3,NUDT18,C1orf87,LHX1,ZMYM3,NPM1,CORO2B,CIAO1,KLHL14,CYGB,PDXK,ANXA13,NFKBIA,ALDH9A1,ACVR2B,C8orf37 |
| Gall bladder | 175 | 7239 | 48.34254144 | 1.209162603 | 0.000831 | 0.329043 | 0.002765 | 0.000642 | CRYM,KCNMB3,HOXA5,EFCAB2,STAT6,ZHX3,CCNB1,ADRB2,DRG1,PROSC,DNAJB5,AFP,STK33,FHL2,NFE2,RAB3B,STAU2,MRPS7,CALCOCO2,FST,NRAS,TYROBP,RPL10,ERAL1,COX4I1,CD47,UPP2,HSPA2,GULP1,TM4SF1,GALT,ELOVL1,LYPD1,GSTP1,RPS6KA2,FUBP1,ATAD2,FAM63A,PPP2R4,SLC39A9,RHOA,DLX5,RAP1GDS1,SGK2,NECAP2,CHD4,ST6GALNAC6,DOK1,PSMD14,IMPDH2,PANK1,DHX9,GNB2L1,CYB5R1,CDC34,CLDN4,RAB39B,RRAGB,ANP32E,NHEJ1,NME1,STARD10,ADD1,CAMK4,TEX264,ZNF641,ANAPC11,DDX58,SPP1,CLGN,SULT1E1,RBKS,CCNH,AAK1,APEX1,CCT4,CCR10,BAG5,MAPK3,CDCP1,YWHAB,DBNL,TCOF1,GGT6,RPL14,C1QC,C1orf21,USP15,MORC4,MIPOL1,PTPN11,SMYD3,MCM5,DIABLO,PDLIM3,CKAP2,NAGK,CPNE4,MEIS2,DERL1,SRPK2,HSPB8,SERBP1,TK1,MARCKSL1,CYP2C8,PRPSAP2,MATK,C9,GTF2I,BCL7C,CDH26,TOM1,PCGF3,PLCB2,PAK6,USP14,TEAD3,COASY,RECQL5,ANXA11,ABLIM3,MAPK1,TXNRD1,MLX,TARDBP,HCRTR1,RASGEF1A,F8,EDNRA,SLA,CETN3,PSMD10,ALDH1A1,EPB41L3,SELENBP1,MMP7,ABCF3,RAB11A,RUVBL1,PAK4,FGFR2,SLC25A10,FGA,DDC,FMN1,TAF7,SNX9,ZNF655,SET,PPP3R1,RPS6,MYLK,VCP,RAC1,MBIP,HSPBP1,AKT3,NUDT18,HIATL1,C1orf87,LHX1,ZMYM3,NPM1,CORO2B,CIAO1,ARHGAP29,KLHL14,CYGB,PDXK,ANXA13,NFKBIA,ALDH9A1,ACVR2B,C8orf37 |
| Saliva | 32 | 880 | 8.839779006 | 1.874138417 | 0.000883 | 0.34966 | 0.002914 | 0.000676 | KLK1,APOE,HSPA2,CARHSP1,IGLL1,GSTP1,CORO1A,TIMP1,STATH,HPX,CYB5R1,SFN,ANP32E,NME1,CAMK4,ARPC3,YWHAB,GGT6,NAGK,PRH2,HBG2,CYP2C8,ANXA6,MAPK1,ALDH1A1,FGA,MYLK,VCP,AKT3,FABP5,PDXK,RNPEP |
| Cervix, uterine | 156 | 6334 | 43.09392265 | 1.232923244 | 0.000902 | 0.357266 | 0.002953 | 0.000685 | CRYM,KCNMB3,HOXA5,EFCAB2,STAT6,ZHX3,CCNB1,ADRB2,DRG1,PROSC,DNAJB5,AFP,STK33,FHL2,NFE2,STAU2,MRPS7,CALCOCO2,FST,NRAS,RPL10,ERAL1,APOE,COX4I1,CD47,UPP2,HSPA2,GULP1,TM4SF1,GALT,ELOVL1,LYPD1,GSTP1,RPS6KA2,FUBP1,ATAD2,TIMP1,PPP2R4,SLC39A9,RHOA,DLX5,RAP1GDS1,CHD4,DOK1,PSMD14,IMPDH2,DHX9,GNB2L1,MRTO4,CYB5R1,CDC34,CLDN4,RAB39B,RRAGB,SFN,ANP32E,NHEJ1,NME1,STARD10,CAMK4,TEX264,ZNF641,ANAPC11,DDX58,SPP1,CLGN,SULT1E1,PTS,RBKS,AAK1,APEX1,CCT4,CCR10,BAG5,MAPK3,CDCP1,YWHAB,DBNL,TCOF1,GGT6,RPL14,C1orf21,MIPOL1,PTPN11,PRKRA,SMYD3,MCM5,C14orf119,DIABLO,CKAP2,CPNE4,MEIS2,DERL1,SRPK2,HSPB8,SERBP1,TK1,CYP2C8,PRPSAP2,MATK,C9,GTF2I,BCL7C,CDH26,TOM1,PCGF3,PLCB2,PAK6,ANXA6,USP14,COASY,RECQL5,ANXA11,ABLIM3,MAPK1,TXNRD1,MLX,TARDBP,RASGEF1A,MOXD1,F8,EDNRA,CETN3,PSMD10,ABCF3,RAB11A,RUVBL1,PAK4,FGFR2,SLC25A10,DDC,FMN1,TAF7,SNX9,ZNF655,SET,RPS6,MYLK,VCP,MBIP,HSPBP1,AKT3,NUDT18,C1orf87,LHX1,ZMYM3,NPM1,CORO2B,CIAO1,KLHL14,PDXK,ANXA13,NFKBIA,ALDH9A1,ACVR2B,C8orf37 |
| Prostate | 187 | 7855 | 51.65745856 | 1.190224357 | 0.001031 | 0.408095 | 0.003345 | 0.000776 | CRYM,KCNMB3,HOXA5,EFCAB2,STAT6,ZHX3,ADRB2,DRG1,PROSC,DNAJB5,AFP,STK33,FHL2,NFE2,RAB3B,STAU2,MRPS7,DAZAP2,CALCOCO2,FST,NRAS,TYROBP,RPL10,KLK1,CSRP1,ERAL1,APOE,COX4I1,CD47,HSPA2,GULP1,GALT,SP110,ELOVL1,LYPD1,GSTP1,RPS6KA2,FUBP1,ATAD2,FAM63A,TIMP1,OLR1,PPP2R4,SLC39A9,RHOA,DLX5,SPAG16,CHD4,ST6GALNAC6,PSMD14,IMPDH2,PANK1,DHX9,GNB2L1,BCAS2,CYB5R1,CDC34,CLDN4,RAB39B,RRAGB,CTNNAL1,SFN,ANP32E,NHEJ1,NME1,STARD10,ADD1,CAMK4,HN1,ANAPC11,DDX58,ARPC3,SPP1,CLGN,EIF4EBP3,PTS,RBKS,CCNH,AAK1,APEX1,CCR10,ABLIM1,BAG5,MAPK3,CDCP1,YWHAB,DBNL,TCOF1,GGT6,RPL14,C1QC,C1orf21,USP15,MORC4,UGDH,ASF1A,MIPOL1,PTPN11,PRKRA,SMYD3,MCM5,DIABLO,PI16,CKAP2,NAGK,CPNE4,MEIS2,DERL1,SRPK2,HSPB8,SERBP1,MARCKSL1,PRPSAP2,MATK,GTF2I,BCL7C,CDH26,NDUFA8,TOM1,FBXL3,PCGF3,PLCB2,PAK6,ANXA6,USP14,CLP1,TEAD3,COASY,RECQL5,ANXA11,RNF11,ABLIM3,MAPK1,TXNRD1,MLX,TARDBP,RASGEF1A,F8,EDNRA,CETN3,PSMD10,LEPROTL1,SELENBP1,MMP7,ABCF3,RAB11A,RUVBL1,SNX1,PAK4,FGFR2,SLC25A10,FGA,DDC,FMN1,TAF7,HSPBAP1,SNX9,FBP1,ZNF655,SET,PPP3R1,PRKAR2B,RPS6,MYLK,VCP,RAC1,COG3,MBIP,HSPBP1,AKT3,NUDT18,HIATL1,C1orf87,LHX1,FABP5,SSPN,ZMYM3,NPM1,CIAO1,ARHGAP29,KLHL14,RTN4,PDXK,ANXA13,NFKBIA,ALDH9A1,C8orf37 |
| B cells | 22 | 528 | 6.077348066 | 2.182082841 | 0.001053 | 0.417094 | 0.003391 | 0.000787 | NRAS,APOE,HLA-DRB5,CORO1A,RHOA,IMPDH2,ARPC3,CCT4,YWHAB,RPL14,RPL22,SERBP1,MARCKSL1,RAB8B,ANXA6,RPS13,ANXA11,SRI,RUVBL1,PECAM1,VCP,RAC1 |
| Kidney | 233 | 10172 | 64.36464088 | 1.14374452 | 0.001087 | 0.430322 | 0.00347 | 0.000806 | CRYM,KCNMB3,HOXA5,EFCAB2,STAT6,CD84,ZHX3,CCNB1,ADRB2,DRG1,PROSC,DNAJB5,SRPX2,AFP,STK33,FHL2,NFE2,RAB3B,STAU2,MRPS7,CALCOCO2,FST,NRAS,TYROBP,RPL10,ERAL1,BHMT2,APOE,COX4I1,CD47,UPP2,HSPA2,GULP1,TM4SF1,GALT,SP110,ELOVL1,LYPD1,NAPSA,GSTP1,RPS6KA2,FUBP1,ATAD2,FAM63A,CAPRIN2,OLR1,PPP2R4,SLC39A9,IFIT3,RHOA,LAYN,DLX5,RAP1GDS1,ATG3,HPX,SGK2,NECAP2,SPAG16,CHD4,ST6GALNAC6,DOK1,MRPS25,PSMD14,SLC5A6,IMPDH2,PANK1,XPR1,DHX9,GNB2L1,MRTO4,BCAS2,CYB5R1,CDC34,CLDN4,RAB39B,LCAT,RRAGB,CTNNAL1,ANP32E,FN3K,NHEJ1,NME1,STARD10,ADD1,CAMK4,TEX264,ZNF641,TMEM185A,ANAPC11,DDX58,ARPC3,SPP1,CLGN,SULT1E1,KLF11,EIF4EBP3,PTS,RBKS,CCNH,AAK1,ARHGDIG,APEX1,CCT4,FNIP1,CCR10,ABLIM1,BAG5,MAPK3,TIRAP,CDCP1,YWHAB,DBNL,TCOF1,GGT6,RPL14,C1QC,C1orf21,USP15,MORC4,UGDH,ASF1A,MIPOL1,PTPN11,ZFYVE19,PRKRA,SMYD3,C14orf119,DIABLO,RBBP7,PI16,CKAP2,NAGK,CPNE4,MEIS2,MCTS1,DERL1,SRPK2,HSPB8,SERBP1,MARCKSL1,CYP2C8,STUB1,PRPSAP2,MATK,C9,GTF2I,BCL7C,NDUFA8,DAPP1,TOM1,FBXL3,PCGF3,PLCB2,PAK6,ANXA6,USP14,CLP1,TEAD3,COASY,RECQL5,ANXA11,RNF11,ABLIM3,MAPK1,TXNRD1,POLE3,MLX,TARDBP,RASGEF1A,MOXD1,F8,EDNRA,SLA,CETN3,PSMD10,LRFN1,LEPROTL1,ALDH1A1,EPB41L3,SAR1B,SELENBP1,MMP7,ABCF3,RAB11A,RUVBL1,SNX1,PAK4,FGFR2,SLC25A10,FGA,DDC,FMN1,PECAM1,TAF7,HSPBAP1,SNX9,FBP1,ZNF655,SET,PPP3R1,PRKAR2B,RPS6,ATP6V1C2,MYLK,VCP,RAC1,COG3,MBIP,HSPBP1,AKT3,NUDT18,HIATL1,GFM2,C1orf87,LHX1,ZRANB2,FABP5,SSPN,ZMYM3,NPM1,CORO2B,CIAO1,ARHGAP29,KLHL14,CYGB,RTN4,PDXK,ANXA13,NFKBIA,ALDH9A1,ACVR2B,C8orf37,UBE2O |
| Huh7 | 82 | 2958 | 22.6519337 | 1.397371011 | 0.001117 | 0.442338 | 0.003539 | 0.000821 | DOHH,ZHX3,DRG1,PROSC,SRPX2,AFP,STAU2,MRPS7,FST,NRAS,RPL10,HLA-DRB5,SP110,EDN3,FUBP3,GSTP1,ATAD2,QDPR,IFIT3,HPX,SGK2,CHD4,IMPDH2,PANK1,GLYAT,BCAS2,ARL2BP,VSNL1,FBXL18,SFN,ANP32E,ADD1,HN1,HLA-DRB3,EIF4EBP3,ARHGDIG,APEX1,ZNF761,CCT4,CCR10,MAPK3,MYOT,DBNL,UGDH,ENOPH1,PTPN11,SMYD3,MCM5,QTRT1,CKAP2,MCTS1,RPL22,TK1,RNF126,GTF2I,TOM1,USP14,RECQL5,PDS5B,MAPK1,SRI,TARDBP,HCRTR1,F8,PSMD10,ALDH1A1,RUVBL1,PAK4,LST1,RPS6,MYLK,VCP,HSPBP1,GKAP1,CHORDC1,GFM2,GBE1,FABP5,SSPN,KRCC1,CIAO1,PLEKHG2 |
| Aspc1 | 20 | 466 | 5.524861878 | 2.259013196 | 0.001238 | 0.490106 | 0.00389 | 0.000903 | DRG1,GSTP1,TIMP1,GNB2L1,SFN,ANP32E,NME1,APEX1,CCT4,YWHAB,UGDH,TXNRD1,ALDH1A1,MMP7,FBP1,SET,VCP,NPM1,PDXK,ANXA13 |
| Hippocampus | 175 | 7299 | 48.34254144 | 1.199224621 | 0.001292 | 0.511733 | 0.004029 | 0.000933 | CRYM,KCNMB3,HOXA5,EFCAB2,ZHX3,DRG1,PROSC,DNAJB5,AFP,STK33,FHL2,NFE2,STAU2,MRPS7,CALCOCO2,FST,NRAS,TYROBP,RPL10,ERAL1,APOE,COX4I1,HSPA2,GULP1,TM4SF1,GALT,ELOVL1,LYPD1,GSTP1,RPS6KA2,FUBP1,ATAD2,PPP2R4,SLC39A9,RHOA,DLX5,RAP1GDS1,HPX,SGK2,NECAP2,CHD4,ST6GALNAC6,DOK1,PSMD14,IMPDH2,PANK1,XPR1,DHX9,GNB2L1,MRTO4,CYB5R1,CDC34,CLDN4,LCAT,VSNL1,RRAGB,SFN,ANP32E,SEPT1,NHEJ1,NME1,STARD10,ADD1,CAMK4,TEX264,ZNF641,ANAPC11,ARPC3,SPP1,CLGN,PTS,RBKS,CCNH,AAK1,ARHGDIG,APEX1,CCT4,FNIP1,CCR10,BAG5,MAPK3,YWHAB,DBNL,TCOF1,GGT6,RPL14,C1QC,C1orf21,USP15,MORC4,MIPOL1,PTPN11,PRKRA,SMYD3,C14orf119,DIABLO,CKAP2,GABRA5,NAGK,CPNE4,MEIS2,DERL1,SRPK2,HSPB8,SERBP1,TK1,MARCKSL1,CYP2C8,PRPSAP2,MATK,C9,GTF2I,BCL7C,CDH26,NDUFA8,TOM1,PCGF3,PLCB2,PAK6,ANXA6,USP14,COASY,ANXA11,PDS5B,ABLIM3,MAPK1,TXNRD1,MLX,TARDBP,RASGEF1A,F8,EDNRA,SLA,CETN3,PSMD10,LRFN1,ALDH1A1,SELENBP1,ABCF3,RAB11A,RUVBL1,PAK4,FGFR2,DDC,FMN1,TAF7,SNX9,ZNF655,SET,PPP3R1,PRKAR2B,RPS6,MYLK,VCP,RAC1,MBIP,HSPBP1,AKT3,NUDT18,HIATL1,LHX1,ZMYM3,NPM1,CORO2B,CIAO1,ARHGAP29,KLHL14,CYGB,RTN4,PDXK,ANXA13,NFKBIA,ALDH9A1,ACVR2B,C8orf37 |
| MV4-11 | 17 | 368 | 4.696132597 | 2.455000068 | 0.001299 | 0.514483 | 0.004019 | 0.000933 | CCNB1,GSTP1,CORO1A,GNB2L1,FAM58A,DCK,CCNH,MAPK3,YWHAB,RPS13,MAPK1,PAK4,SLC25A10,RPS6,VCP,PHKG2,PDXK |
| Embryonic stem cells | 82 | 2996 | 22.6519337 | 1.379654739 | 0.001625 | 0.643534 | 0.004989 | 0.001158 | CCNB1,SRPX2,FHL2,RAB3B,STAU2,MRPS7,NRAS,RPL10,KRR1,CSRP1,APOE,COX4I1,CD47,AEBP2,HSPA2,GULP1,ELOVL1,FUBP3,FUBP1,RPS7,RHOA,CHD4,SLC5A6,IMPDH2,XPR1,DHX9,GNB2L1,CYB5R1,RAB39B,FBXL18,ANP32E,FN3K,RPS15,TEX264,ANAPC11,RAB24,EIF4EBP3,AAK1,APEX1,CCT4,CDCP1,ARF5,RPL14,UGDH,MCM5,RBBP7,DERL1,RPL22,SERBP1,MARCKSL1,STUB1,PRPSAP2,RAB8B,GTF2I,PYCR1,ANXA6,RPS13,USP14,COASY,ANXA11,PDS5B,TXNRD1,TARDBP,RAB11A,RUVBL1,TPD52L2,FGFR2,SLC25A10,SET,PRKAR2B,RPS6,RAC1,HSPBP1,SRP54,GFM2,ZMYM3,NPM1,GOLT1B,RTN4,RNPEP,ALDH9A1,UBE2O |
| Vulva/anal skin | 154 | 6325 | 42.54143646 | 1.218973887 | 0.001727 | 0.683778 | 0.00526 | 0.001221 | KCNMB3,HOXA5,EFCAB2,STAT6,ZHX3,CCNB1,DRG1,PROSC,DNAJB5,AFP,STK33,FHL2,NFE2,STAU2,MRPS7,CALCOCO2,FST,NRAS,TYROBP,RPL10,ERAL1,COX4I1,CD47,UPP2,HSPA2,GULP1,TM4SF1,GALT,ELOVL1,LYPD1,GSTP1,RPS6KA2,FUBP1,ATAD2,FAM63A,TIMP1,SLC39A9,DLX5,RAP1GDS1,SGK2,NECAP2,CHD4,ST6GALNAC6,DOK1,PSMD14,IMPDH2,DHX9,GNB2L1,MRTO4,CYB5R1,CDC34,CLDN4,RRAGB,SFN,NHEJ1,STARD10,ADD1,CAMK4,ZNF641,ANAPC11,DDX58,SPP1,SULT1E1,PTS,RBKS,AAK1,APEX1,CCT4,CCR10,BAG5,MAPK3,CDCP1,YWHAB,DBNL,TCOF1,GGT6,RPL14,C1QC,C1orf21,MORC4,PTPN11,PRKRA,SMYD3,MCM5,C14orf119,DIABLO,CKAP2,NAGK,CPNE4,MEIS2,DERL1,SRPK2,HSPB8,SERBP1,TK1,PRPSAP2,MATK,C9,GTF2I,BCL7C,TOM1,PCGF3,PLCB2,PAK6,ANXA6,USP14,COASY,RECQL5,ANXA11,ABLIM3,MAPK1,TXNRD1,MLX,TARDBP,RASGEF1A,MOXD1,F8,EDNRA,CETN3,PSMD10,ALDH1A1,MMP7,ABCF3,RAB11A,RUVBL1,PAK4,FGFR2,SLC25A10,DDC,FMN1,TAF7,SNX9,ZNF655,SET,PRKAR2B,RPS6,MYLK,VCP,HSPBP1,AKT3,NUDT18,C1orf87,LHX1,ZMYM3,NPM1,CORO2B,CIAO1,ARHGAP29,KLHL14,PDXK,ANXA13,NFKBIA,ALDH9A1,ACVR2B |
| Bladder cancer cells | 16 | 347 | 4.419889503 | 2.460407929 | 0.00182 | 0.72064 | 0.005501 | 0.001277 | RPL10,GSTP1,RHOA,SLC5A6,DHX9,GNB2L1,SFN,NME1,CCT4,YWHAB,ANXA6,ANXA11,VCP,RAC1,RTN4,RNPEP |
| Vagina | 131 | 5258 | 36.1878453 | 1.249053408 | 0.002051 | 0.812375 | 0.006154 | 0.001429 | KCNMB3,HOXA5,EFCAB2,STAT6,CCNB1,DRG1,PROSC,DNAJB5,AFP,STK33,FHL2,NFE2,STAU2,MRPS7,FST,NRAS,RPL10,ERAL1,APOE,COX4I1,UPP2,HSPA2,GULP1,GALT,ELOVL1,LYPD1,GSTP1,RPS6KA2,FUBP1,ATAD2,FAM63A,PPP2R4,SLC39A9,DLX5,NECAP2,CHD4,DOK1,PSMD14,IMPDH2,DHX9,GNB2L1,MRTO4,CYB5R1,CLDN4,SFN,ANP32E,NHEJ1,NME1,ZNF641,ANAPC11,DDX58,SPP1,SULT1E1,PTS,APEX1,CCT4,CCR10,BAG5,MAPK3,CDCP1,YWHAB,DBNL,TCOF1,GGT6,RPL14,C1QC,C1orf21,MORC4,PTPN11,SMYD3,MCM5,C14orf119,DIABLO,CKAP2,NAGK,CPNE4,MEIS2,SRPK2,HSPB8,SERBP1,PRPSAP2,C9,GTF2I,TOM1,PCGF3,PLCB2,PAK6,ANXA6,TEAD3,COASY,RECQL5,ANXA11,ABLIM3,MAPK1,TXNRD1,MLX,TARDBP,RASGEF1A,F8,EDNRA,CETN3,PSMD10,SELENBP1,ABCF3,PAK4,FGFR2,SLC25A10,FMN1,TAF7,SNX9,ZNF655,SET,PPP3R1,RPS6,MYLK,VCP,HSPBP1,AKT3,NUDT18,LHX1,ZMYM3,NPM1,CORO2B,CIAO1,KLHL14,RTN4,PDXK,ANXA13,NFKBIA,ALDH9A1,ACVR2B |
| Semen | 32 | 928 | 8.839779006 | 1.777330622 | 0.002069 | 0.819404 | 0.006161 | 0.00143 | RAB3B,APOE,CD47,HSPA2,GSTP1,TIMP1,PPP2R4,RHOA,HPX,PSMD14,GNB2L1,PTN,SFN,NME1,CCT4,YWHAB,UGDH,RAB8B,C9,ANXA6,USP14,ANXA11,ALDH1A1,SELENBP1,MMP7,RUVBL1,FBP1,VCP,RAC1,FABP5,PGM2,ALDH9A1 |
| Seminal vesicle | 158 | 6557 | 43.64640884 | 1.206148043 | 0.002299 | 0.910242 | 0.006793 | 0.001577 | KCNMB3,HOXA5,EFCAB2,STAT6,ZHX3,CCNB1,DRG1,PROSC,DNAJB5,AFP,STK33,FHL2,NFE2,RAB3B,STAU2,MRPS7,CALCOCO2,FST,NRAS,RPL10,ERAL1,APOE,COX4I1,CD47,UPP2,HSPA2,GALT,ELOVL1,LYPD1,GSTP1,RPS6KA2,FUBP1,ATAD2,FAM63A,PPP2R4,SLC39A9,RHOA,DLX5,RAP1GDS1,SGK2,CHD4,PSMD14,IMPDH2,PANK1,DHX9,GNB2L1,MRTO4,CYB5R1,CDC34,CLDN4,RAB39B,RRAGB,SFN,NHEJ1,NME1,STARD10,ADD1,CAMK4,TEX264,ZNF641,ANAPC11,DDX58,ARPC3,SPP1,SULT1E1,RBKS,AAK1,APEX1,CCT4,CCR10,BAG5,MAPK3,CDCP1,YWHAB,DBNL,TCOF1,GGT6,RPL14,C1orf21,USP15,MORC4,MIPOL1,PTPN11,PRKRA,SMYD3,DIABLO,PDLIM3,CKAP2,NAGK,MEIS2,DERL1,SRPK2,HSPB8,SERBP1,CYP2C8,PRPSAP2,MATK,C9,GTF2I,BCL7C,CDH26,TOM1,PCGF3,PLCB2,ANXA6,USP14,TEAD3,COASY,RECQL5,ANXA11,ABLIM3,MAPK1,TXNRD1,MLX,TARDBP,RASGEF1A,F8,EDNRA,SLA,CETN3,PSMD10,ALDH1A1,SELENBP1,ABCF3,RAB11A,RUVBL1,PAK4,FGFR2,SLC25A10,DDC,FMN1,TAF7,SNX9,FBP1,ZNF655,SET,PPP3R1,PRKAR2B,RPS6,MYLK,VCP,RAC1,MBIP,HSPBP1,AKT3,NUDT18,ZMYM3,NPM1,CIAO1,ARHGAP29,KLHL14,CYGB,PDXK,ANXA13,NFKBIA,ALDH9A1,ACVR2B,C8orf37 |
| Dermal microvascular endothelial cells | 3 | 14 | 0.828729282 | 13.8429366 | 0.002474 | 0.979589 | 0.007256 | 0.001684 | CCR10,MYLK,FABP5 |
| BT474 | 21 | 530 | 5.801104972 | 2.080368244 | 0.002492 | 0.986965 | 0.007257 | 0.001685 | CSRP1,CARHSP1,FUBP1,TIMP1,IMPDH2,GNB2L1,NME1,HN1,ARPC3,APEX1,YWHAB,UGDH,RBBP7,HBG2,SERBP1,USP14,FBP1,SET,VCP,NPM1,RNPEP |
| Duodenum | 171 | 7216 | 47.23756906 | 1.185486534 | 0.002819 | 1 | 0.008147 | 0.001891 | CRYM,KCNMB3,HOXA5,EFCAB2,STAT6,ZHX3,CCNB1,DRG1,PROSC,DNAJB5,AFP,STK33,FHL2,NFE2,RAB3B,STAU2,MRPS7,CALCOCO2,FST,NRAS,TYROBP,RPL10,ERAL1,COX4I1,UPP2,HSPA2,GULP1,GALT,ELOVL1,LYPD1,GSTP1,RPS6KA2,FUBP1,ATAD2,FAM63A,TIMP1,PPP2R4,SLC39A9,RHOA,DLX5,RAP1GDS1,SGK2,NECAP2,HLA-DOB,CHD4,PSMD14,IMPDH2,PANK1,DHX9,GNB2L1,MRTO4,CYB5R1,CDC34,CLDN4,RRAGB,SFN,NHEJ1,NME1,STARD10,ADD1,CAMK4,TEX264,ZNF641,ANAPC11,DDX58,ARPC3,SPP1,CLGN,SULT1E1,RBKS,CCNH,AAK1,APEX1,CCT4,CCR10,BAG5,MAPK3,CDCP1,YWHAB,DBNL,TCOF1,GGT6,RPL14,C1QC,C1orf21,USP15,MORC4,MIPOL1,PTPN11,PRKRA,SMYD3,MCM5,DIABLO,CKAP2,NAGK,MEIS2,DERL1,HSPB8,HBG2,SERBP1,TK1,MARCKSL1,CYP2C8,PRPSAP2,MATK,C9,GTF2I,BCL7C,CDH26,TOM1,PCGF3,PLCB2,PAK6,USP14,TEAD3,COASY,RECQL5,ANXA11,ABLIM3,MAPK1,TXNRD1,MLX,TARDBP,MOXD1,F8,EDNRA,SLA,CETN3,PSMD10,ALDH1A1,EPB41L3,SELENBP1,ABCF3,RAB11A,RUVBL1,PAK4,FGFR2,SLC25A10,FGA,DDC,FMN1,TAF7,SNX9,FBP1,ZNF655,SET,PPP3R1,PRKAR2B,RPS6,MYLK,VCP,RAC1,MBIP,HSPBP1,AKT3,NUDT18,C1orf87,LHX1,ZMYM3,NPM1,CIAO1,ARHGAP29,KLHL14,CYGB,RTN4,PDXK,ANXA13,NFKBIA,ALDH9A1,ACVR2B,C8orf37 |
| Lung | 225 | 9906 | 62.15469613 | 1.134343559 | 0.002872 | 1 | 0.00824 | 0.001913 | CRYM,KCNMB3,HOXA5,EFCAB2,STAT6,CD84,ZHX3,CCNB1,ADRB2,DRG1,PROSC,DNAJB5,AFP,STK33,FHL2,NFE2,STAU2,MRPS7,CALCOCO2,FST,NRAS,TYROBP,RPL10,KLK1,ERAL1,APOE,COX4I1,CD47,UPP2,HSPA2,GULP1,TM4SF1,GALT,SP110,ELOVL1,LYPD1,NAPSA,GSTP1,RPS6KA2,CORO1A,FUBP1,ATAD2,FAM63A,CAPRIN2,OLR1,PPP2R4,SLC39A9,QDPR,IFIT3,RHOA,LAYN,DLX5,RAP1GDS1,ATG3,HPX,NECAP2,SPAG16,CHD4,ST6GALNAC6,DOK1,MRPS25,PSMD14,SLC5A6,IMPDH2,PANK1,XPR1,DHX9,GNB2L1,MRTO4,BCAS2,CYB5R1,CDC34,CLDN4,RAB39B,LCAT,RRAGB,CTNNAL1,SFN,ANP32E,FN3K,NHEJ1,NME1,STARD10,ADD1,CAMK4,TEX264,ZNF641,ANAPC11,DDX58,ARPC3,SPP1,CLGN,SULT1E1,KLF11,NOSIP,EIF4EBP3,RBKS,AAK1,ARHGDIG,APEX1,CCT4,FNIP1,CCR10,ABLIM1,BAG5,MAPK3,TIRAP,CDCP1,YWHAB,DBNL,TCOF1,GGT6,RPL14,C1QC,C1orf21,USP15,MORC4,UGDH,MIPOL1,PTPN11,PRKRA,SMYD3,MCM5,DIABLO,CKAP2,NAGK,CPNE4,MEIS2,DERL1,SRPK2,HSPB8,HBG2,SERBP1,TK1,CYP2C8,STUB1,PRPSAP2,MATK,C9,GTF2I,BCL7C,CDH26,NDUFA8,DAPP1,TOM1,FBXL3,PCGF3,PLCB2,PAK6,ANXA6,USP14,TEAD3,COASY,RECQL5,ANXA11,RNF11,ABLIM3,MAPK1,TXNRD1,POLE3,CCL5,MLX,TARDBP,MOXD1,F8,EDNRA,SLA,CETN3,PSMD10,LRFN1,LEPROTL1,ALDH1A1,EPB41L3,SAR1B,SELENBP1,MMP7,ABCF3,RAB11A,RUVBL1,SNX1,FGFR2,SLC25A10,FGA,DDC,FMN1,PECAM1,TAF7,HSPBAP1,SNX9,FBP1,ZNF655,SET,PPP3R1,PRKAR2B,RPS6,MYLK,VCP,RAC1,COG3,MBIP,HSPBP1,PLEK,AKT3,HIATL1,GFM2,C1orf87,LHX1,ZRANB2,FABP5,SSPN,ZMYM3,NPM1,CORO2B,CIAO1,ARHGAP29,KLHL14,CYGB,RTN4,PDXK,ANXA13,NFKBIA,DGUOK,ALDH9A1,C8orf37,UBE2O |
| Salivary glands | 157 | 6549 | 43.37016575 | 1.200037967 | 0.003036 | 1 | 0.008649 | 0.002008 | CRYM,KCNMB3,HOXA5,EFCAB2,STAT6,ZHX3,CCNB1,DRG1,PROSC,DNAJB5,AFP,STK33,NFE2,STAU2,MRPS7,CALCOCO2,FST,NRAS,TYROBP,RPL10,ERAL1,COX4I1,UPP2,HSPA2,GULP1,TM4SF1,GALT,ELOVL1,LYPD1,GSTP1,RPS6KA2,FUBP1,ATAD2,FAM63A,TIMP1,OLR1,PPP2R4,SLC39A9,RHOA,DLX5,CHD4,ST6GALNAC6,IMPDH2,XPR1,DHX9,GNB2L1,MRTO4,CYB5R1,CDC34,CLDN4,RAB39B,RRAGB,SFN,ANP32E,NHEJ1,NME1,STARD10,ADD1,CAMK4,ZNF641,ANAPC11,DDX58,ARPC3,SPP1,SULT1E1,RBKS,AAK1,APEX1,CCR10,BAG5,MAPK3,CDCP1,YWHAB,DBNL,TCOF1,GGT6,RPL14,C1QC,C1orf21,USP15,MORC4,MIPOL1,PTPN11,PRKRA,SMYD3,C14orf119,DIABLO,PI16,CKAP2,NAGK,CPNE4,MEIS2,DERL1,SRPK2,HSPB8,SERBP1,CYP2C8,PRPSAP2,MATK,C9,GTF2I,NDUFA8,TOM1,PCGF3,PLCB2,PAK6,ANXA6,USP14,COASY,RECQL5,ANXA11,ABLIM3,MAPK1,TXNRD1,MLX,TARDBP,MOXD1,F8,EDNRA,SLA,CETN3,PSMD10,ALDH1A1,SELENBP1,ABCF3,RAB11A,RUVBL1,SNX1,PAK4,FGFR2,SLC25A10,DDC,FMN1,TAF7,SNX9,FBP1,ZNF655,SET,PPP3R1,PRKAR2B,RPS6,MYLK,VCP,RAC1,HSPBP1,AKT3,NUDT18,LHX1,ZMYM3,NPM1,CIAO1,KLHL14,CYGB,PDXK,ANXA13,NFKBIA,ALDH9A1 |
| Preadipocytes | 20 | 506 | 5.524861878 | 2.080875143 | 0.003218 | 1 | 0.009101 | 0.002113 | AFP,APOE,AEBP2,GSTP1,TIMP1,GNB2L1,NME1,ARPC3,YWHAB,UGDH,ANXA6,TXNRD1,CCL5,SELENBP1,TPD52L2,RPS6,VCP,FABP5,NPM1,RTN4 |
| Limb bud | 2 | 5 | 0.552486188 | 25.82929112 | 0.003857 | 1 | 0.010832 | 0.002515 | HOXA5,EYA1 |
| Lateral ventricle | 156 | 6563 | 43.09392265 | 1.189911563 | 0.004602 | 1 | 0.012834 | 0.002979 | CRYM,KCNMB3,HOXA5,EFCAB2,ZHX3,PROSC,DNAJB5,AFP,STK33,FHL2,NFE2,STAU2,MRPS7,CALCOCO2,FST,NRAS,TYROBP,RPL10,ERAL1,APOE,COX4I1,UPP2,HSPA2,GULP1,TM4SF1,GALT,ELOVL1,LYPD1,GSTP1,RPS6KA2,FUBP1,ATAD2,FAM63A,TIMP1,PPP2R4,SLC39A9,RHOA,DLX5,RAP1GDS1,HPX,NECAP2,CHD4,ST6GALNAC6,DOK1,PSMD14,IMPDH2,PANK1,XPR1,DHX9,GNB2L1,MRTO4,CYB5R1,CDC34,CLDN4,RRAGB,SFN,ANP32E,NHEJ1,NME1,STARD10,CAMK4,ZNF641,ANAPC11,ARPC3,SPP1,CLGN,SULT1E1,RBKS,AAK1,APEX1,CCT4,BAG5,MAPK3,YWHAB,TCOF1,GGT6,RPL14,C1QC,C1orf21,USP15,MORC4,MIPOL1,PTPN11,PRKRA,SMYD3,C14orf119,DIABLO,CKAP2,NAGK,CPNE4,MEIS2,DERL1,SRPK2,HSPB8,SERBP1,MARCKSL1,CYP2C8,MATK,C9,GTF2I,TOM1,PCGF3,PLCB2,ANXA6,USP14,COASY,RECQL5,ANXA11,ABLIM3,MAPK1,TXNRD1,MLX,TARDBP,RASGEF1A,F8,EDNRA,CETN3,PSMD10,ALDH1A1,SELENBP1,MMP7,ABCF3,RAB11A,RUVBL1,PAK4,FGFR2,FGA,DDC,FMN1,TAF7,SNX9,ZNF655,SET,PPP3R1,PRKAR2B,RPS6,VCP,RAC1,MBIP,HSPBP1,PLEK,AKT3,NUDT18,HIATL1,LHX1,ZMYM3,NPM1,CORO2B,CIAO1,KLHL14,RTN4,PDXK,ANXA13,NFKBIA,ALDH9A1,ACVR2B |
| N06CS97 | 12 | 253 | 3.314917127 | 2.588594716 | 0.005239 | 1 | 0.014508 | 0.003368 | ARPC3,MAPK3,CDCP1,PTPN11,HSPB8,DAPP1,ANXA6,ANXA11,MAPK1,TXNRD1,FGA,PECAM1 |
| N06CS98-2 | 9 | 161 | 2.486187845 | 3.137966505 | 0.005284 | 1 | 0.014531 | 0.003373 | TYROBP,ARPC3,MAPK3,CDCP1,PTPN11,ANXA6,MAPK1,PECAM1,LST1 |
| Embryonal carcinoma cells | 37 | 1196 | 10.22099448 | 1.586922572 | 0.005846 | 1 | 0.015966 | 0.003706 | DRG1,RAB3B,STAU2,MRPS7,NRAS,RPL10,APOE,COX4I1,CD47,ELOVL1,FUBP3,RPS7,PSMD14,SLC5A6,XPR1,GNB2L1,CYB5R1,ADD1,APEX1,CCT4,CDCP1,RPL14,C1orf21,MCM5,SERBP1,MARCKSL1,RAB8B,GTF2I,PYCR1,RPS13,TARDBP,RPS6,VCP,RAC1,NPM1,GOLT1B,RTN4 |
| PT45 | 15 | 358 | 4.143646409 | 2.246806813 | 0.006039 | 1 | 0.016379 | 0.003802 | FST,TIMP1,PTN,SFN,ANP32E,NME1,APEX1,YWHAB,ENOPH1,RPL22,TXNRD1,SET,VCP,FABP5,NPM1 |
| Heart | 132 | 5462 | 36.4640884 | 1.211505212 | 0.006262 | 1 | 0.016869 | 0.003916 | CRYM,KCNMB3,ZHX3,ADRB2,DRG1,PROSC,DNAJB5,SRPX2,STK33,FHL2,NFE2,CALCOCO2,FST,ERAL1,BHMT2,COX4I1,HSPA2,GULP1,TM4SF1,SP110,GSTP1,RPS6KA2,CAPRIN2,OLR1,PPP2R4,LAYN,ATG3,SPAG16,DOK1,MRPS25,PSMD14,SLC5A6,IMPDH2,PANK1,XPR1,GNB2L1,BCAS2,CYB5R1,LCAT,RRAGB,CTNNAL1,FN3K,SEPT1,NME1,ZNF641,TMEM185A,SPP1,KLF11,NOSIP,EIF4EBP3,PTS,AAK1,CCT4,FNIP1,CCR10,ABLIM1,BAG5,TIRAP,MYOT,DBNL,USP15,UGDH,ASF1A,MIPOL1,ZFYVE19,C14orf119,DIABLO,PDLIM3,PI16,CKAP2,NAGK,CPNE4,MEIS2,SRPK2,HSPB8,SERBP1,CYP2C8,STUB1,GTF2I,BCL7C,NDUFA8,DAPP1,TOM1,FBXL3,PAK6,ANXA6,TEAD3,COASY,RECQL5,ANXA11,RNF11,ABLIM3,MAPK1,TXNRD1,POLE3,SRI,MLX,TARDBP,CKM,PSMD10,LRFN1,LEPROTL1,SAR1B,SELENBP1,RUVBL1,SNX1,FGFR2,SLC25A10,HSPBAP1,SNX9,PPP3R1,RPS6,MYLK,RAC1,COG3,MBIP,HSPBP1,AKT3,GFM2,LHX1,ZRANB2,FABP5,SSPN,ZMYM3,CORO2B,ARHGAP29,RTN4,PDXK,NFKBIA,DGUOK,ALDH9A1,UBE2O |
| Oral mucosa | 139 | 5803 | 38.39779006 | 1.20023394 | 0.006604 | 1 | 0.017671 | 0.004089 | CRYM,KCNMB3,HOXA5,EFCAB2,STAT6,ZHX3,CCNB1,DRG1,PROSC,AFP,STK33,FHL2,NFE2,STAU2,MRPS7,CALCOCO2,FST,NRAS,TYROBP,RPL10,ERAL1,APOE,COX4I1,UPP2,HSPA2,GULP1,ELOVL1,LYPD1,GSTP1,RPS6KA2,FUBP1,ATAD2,FAM63A,SLC39A9,DLX5,RAP1GDS1,CHD4,DOK1,PSMD14,IMPDH2,DHX9,GNB2L1,MRTO4,CYB5R1,CDC34,CLDN4,SFN,ANP32E,NHEJ1,NME1,CAMK4,ZNF641,ANAPC11,DDX58,ARPC3,SPP1,SULT1E1,PTS,CCNH,AAK1,APEX1,CCT4,BAG5,MAPK3,CDCP1,YWHAB,DBNL,TCOF1,GGT6,RPL14,C1QC,C1orf21,MORC4,MIPOL1,PTPN11,PRKRA,SMYD3,MCM5,DIABLO,CKAP2,NAGK,CPNE4,MEIS2,DERL1,HSPB8,SERBP1,TK1,MARCKSL1,PRPSAP2,MATK,C9,GTF2I,CDH26,TOM1,PCGF3,PLCB2,PAK6,COASY,RECQL5,ANXA11,ABLIM3,MAPK1,TXNRD1,MLX,TARDBP,RASGEF1A,F8,EDNRA,CETN3,PSMD10,ABCF3,RUVBL1,PAK4,FGFR2,SLC25A10,FGA,DDC,FMN1,TAF7,SNX9,ZNF655,SET,PPP3R1,RPS6,MYLK,VCP,HSPBP1,AKT3,NUDT18,LHX1,ZMYM3,NPM1,CIAO1,KLHL14,PDXK,ANXA13,NFKBIA,ALDH9A1,ACVR2B |
| Tears | 19 | 504 | 5.248618785 | 1.990800994 | 0.006627 | 1 | 0.017613 | 0.004089 | GSTP1,CORO1A,TIMP1,PPP2R4,HPX,GNB2L1,SFN,NME1,MAPK3,YWHAB,PRH2,RAB8B,ANXA11,ALDH1A1,SELENBP1,FGA,FBP1,VCP,FABP5 |
| 051503_BAL20_gly_hui | 12 | 263 | 3.314917127 | 2.490634651 | 0.007065 | 1 | 0.018652 | 0.00433 | APOE,RAP1GDS1,HPX,GNB2L1,CCT4,C1QC,MCM5,RPL22,SERBP1,C9,NDUFA8,FGA |
| Plasma | 259 | 11805 | 71.54696133 | 1.094933911 | 0.007212 | 1 | 0.018913 | 0.00439 | KCNMB3,HOXA5,DOHH,STAT6,CD84,ZHX3,ADRB2,DRG1,PROSC,DNAJB5,AFP,STK33,FHL2,NFE2,RAB3B,STAU2,MRPS7,DAZAP2,CALCOCO2,FST,NRAS,TYROBP,RPL10,KLK1,C2orf44,KRR1,CSRP1,ERAL1,OGFOD2,BHMT2,APOE,HLA-DRB5,COX4I1,HPCAL1,CD47,AEBP2,HSPA2,GULP1,TM4SF1,GALT,SP110,RWDD3,CARHSP1,NAPSA,FUBP3,IGLL1,GSTP1,RPS6KA2,CYBB,CORO1A,FUBP1,ATAD2,FAM63A,CAPRIN2,FBXO31,RPS7,TIMP1,STATH,OLR1,PPP2R4,SLC39A9,ADRBK1,QDPR,IFIT3,RHOA,DLX5,RAP1GDS1,ATG3,TIPIN,HPX,NECAP2,HLA-DOB,CHD4,ST6GALNAC6,DOK1,MRPS25,PSMD14,IMPDH2,GH2,PANK1,DHX9,GNB2L1,CLDN4,LCAT,RRAGB,SFN,ANP32E,FN3K,SEPT1,NHEJ1,NME1,STARD10,ADD1,CAMK4,RPS15,TEX264,DCK,ZNF641,FBXO3,HN1,ARPC3,SPP1,CLGN,RAB24,MTL5,SULT1E1,HLA-DRB3,NOSIP,EIF4EBP3,APEX1,ZNF761,CCT4,SERTAD3,CXXC5,FNIP1,CCR10,MRPL53,ABLIM1,ARIH2,BAG5,MAPK3,CDCP1,MYOT,ARF5,YWHAB,DBNL,TCOF1,RPL14,CALN1,C1QC,TTC1,USP15,MORC4,UGDH,ENOPH1,PTPN11,ZFYVE19,PRKRA,SMYD3,MCM5,C14orf119,DIABLO,QTRT1,PDLIM3,RBBP7,PI16,CKAP2,NAGK,PRH2,CPNE4,MEIS2,HIGD2A,RPL22,FAM81A,ATP6V1E2,C14orf80,HBG2,C11orf49,SERBP1,TK1,MARCKSL1,STUB1,PRPSAP2,RNF126,RAB8B,C9,GTF2I,BCL7C,PYCR1,NDUFA8,TOM1,PLCB2,PAK6,ANXA6,RPS13,USP14,TEAD3,COASY,TMEM106A,RECQL5,ANXA11,PDS5B,MAPK1,TXNRD1,POLE3,CCL5,SRI,TARDBP,MOXD1,CKM,F8,TRIML1,CETN3,PSMD10,LRFN1,ALDH1A1,EPB41L3,SAR1B,SELENBP1,ABCF3,CCDC102B,RAB11A,RUVBL1,TPD52L2,SNX1,PAK4,STK25,FGFR2,SLC25A10,S100A7A,FGA,DDC,FMN1,PECAM1,TAF7,PAF1,SNX9,FBP1,ZNF655,SET,PPP3R1,PRKAR2B,RPS6,MYLK,VCP,RAC1,HSPBP1,PLEK,AKT3,SRP54,NUDT18,CHORDC1,PHKG2,HIATL1,GFM2,C1orf87,ZRANB2,GBE1,EYA1,FABP5,SSPN,ZMYM3,NPM1,KRCC1,CIAO1,PGM2,ARHGAP29,GOLT1B,KLHL14,RTN4,PDXK,RNPEP,PLEKHG2,ANXA13,NFKBIA,ALDH9A1,ACVR2B,UBE2O,PEX19 |
| Hair | 26 | 775 | 7.182320442 | 1.743709112 | 0.007489 | 1 | 0.019512 | 0.004529 | APOE,HSPA2,GSTP1,RPS7,PPP2R4,GNB2L1,SFN,NME1,STARD10,ARPC3,APEX1,YWHAB,DIABLO,TOM1,RPS13,TXNRD1,RUVBL1,TPD52L2,FBP1,SET,RPS6,VCP,RAC1,FABP5,RTN4,RNPEP |
| Entorhinal cortex | 2 | 7 | 0.552486188 | 19.56764479 | 0.007887 | 1 | 0.020413 | 0.004738 | SEPT1,MYLK |
| Monocytes | 25 | 741 | 6.906077348 | 1.756662155 | 0.008087 | 1 | 0.020796 | 0.0048 | CD84,APOE,COX4I1,CD47,ELOVL1,CYBB,TIMP1,OLR1,SLC5A6,DHX9,GNB2L1,DCK,PTS,CCT4,CCR10,YWHAB,RPL14,ANXA6,RPS13,ANXA11,ALDH1A1,PECAM1,RAC1,GOLT1B,RTN4 |
| Cellzome_Abl_inhibitors_NatureBiotechnology_exp143 | 6 | 87 | 1.657458564 | 4.080679029 | 0.008094 | 1 | 0.02068 | 0.0048 | ANP32E,DCK,CCNH,MAPK3,MAPK1,PAK4 |
| Panc1 | 14 | 340 | 3.867403315 | 2.219761564 | 0.009065 | 1 | 0.023012 | 0.005342 | APOE,GSTP1,TIMP1,LAYN,SFN,APEX1,CCT4,YWHAB,TXNRD1,RUVBL1,SET,VCP,FABP5,NPM1 |
| hupo1_b2-hep_cam | 27 | 829 | 7.458563536 | 1.690124555 | 0.00941 | 1 | 0.023734 | 0.005509 | MRPS7,NRAS,COX4I1,CORO1A,RPS7,STATH,SLC39A9,HPX,SLC5A6,XPR1,GNB2L1,CYB5R1,RAB39B,TEX264,CLGN,CCT4,PI16,MARCKSL1,RAB8B,SLA,RAB11A,SLC25A10,FGA,VCP,RAC1,GOLT1B,RTN4 |
| MDA231 | 13 | 310 | 3.591160221 | 2.274126651 | 0.01014 | 1 | 0.025414 | 0.005899 | GSTP1,TIMP1,SFN,NME1,ARPC3,APEX1,CCT4,YWHAB,TXNRD1,SET,VCP,GBE1,NPM1 |
| J82 | 10 | 212 | 2.762430939 | 2.620430383 | 0.010743 | 1 | 0.026756 | 0.006211 | GSTP1,NME1,ARPC3,YWHAB,SERBP1,ANXA6,TXNRD1,GBE1,FABP5,PGM2 |
| DU145 | 29 | 920 | 8.011049724 | 1.631011494 | 0.011068 | 1 | 0.027392 | 0.006359 | AFP,RAB3B,NRAS,CSRP1,ELOVL1,RPS7,TIMP1,SLC5A6,IMPDH2,DHX9,GNB2L1,NME1,CLGN,APEX1,CCT4,CDCP1,YWHAB,UGDH,PTPN11,RPL22,SERBP1,ANXA6,USP14,TXNRD1,RUVBL1,VCP,RAC1,NPM1,RTN4 |
| HCC78 | 11 | 246 | 3.038674033 | 2.460984804 | 0.011158 | 1 | 0.027444 | 0.00637 | PPP2R4,IFIT3,DOK1,EPS8L1,ABLIM1,MAPK3,CDCP1,DBNL,PTPN11,DAPP1,MAPK1 |
| Submandibular gland | 3 | 25 | 0.828729282 | 8.042086979 | 0.013283 | 1 | 0.032469 | 0.007537 | STATH,RPL22,FGFR2 |
| Cerebellum | 173 | 7570 | 47.79005525 | 1.143179278 | 0.013589 | 1 | 0.033013 | 0.007663 | CRYM,KCNMB3,HOXA5,EFCAB2,STAT6,ZHX3,DRG1,PROSC,DNAJB5,AFP,STK33,FHL2,NFE2,RAB3B,STAU2,MRPS7,CALCOCO2,FST,NRAS,TYROBP,RPL10,ERAL1,APOE,COX4I1,CD47,HSPA2,GULP1,GALT,ELOVL1,LYPD1,GSTP1,RPS6KA2,FUBP1,ATAD2,FAM63A,PPP2R4,SLC39A9,RHOA,DLX5,RAP1GDS1,SGK2,CHD4,DOK1,PSMD14,IMPDH2,XPR1,DHX9,GNB2L1,MRTO4,CYB5R1,CDC34,CLDN4,RAB39B,LCAT,VSNL1,RRAGB,SFN,ANP32E,NHEJ1,NME1,STARD10,CAMK4,ANAPC11,ARPC3,SPP1,CLGN,SULT1E1,PTS,RBKS,CCNH,AAK1,ARHGDIG,APEX1,CCT4,FNIP1,CCR10,BAG5,MAPK3,CDCP1,YWHAB,DBNL,TCOF1,GGT6,RPL14,C1QC,C1orf21,USP15,MORC4,MIPOL1,PTPN11,PRKRA,SMYD3,C14orf119,DIABLO,PDLIM3,CKAP2,GABRA5,NAGK,MEIS2,DERL1,SRPK2,HSPB8,SERBP1,MARCKSL1,PRPSAP2,MATK,GTF2I,CDH26,NDUFA8,TOM1,FBXL3,PCGF3,PLCB2,ANXA6,USP14,TEAD3,COASY,RECQL5,ANXA11,PDS5B,ABLIM3,MAPK1,TXNRD1,MLX,TARDBP,HCRTR1,RASGEF1A,MOXD1,F8,EDNRA,SLA,CETN3,PSMD10,LRFN1,ALDH1A1,EPB41L3,SELENBP1,MMP7,ABCF3,RAB11A,RUVBL1,PAK4,FGFR2,SLC25A10,DDC,FMN1,TAF7,SNX9,ZNF655,SET,PPP3R1,PRKAR2B,RPS6,MYLK,VCP,RAC1,MBIP,HSPBP1,AKT3,HIATL1,LHX1,ZMYM3,NPM1,CORO2B,CIAO1,ARHGAP29,KLHL14,CYGB,RTN4,PDXK,ANXA13,NFKBIA,ALDH9A1 |
| Muscle | 28 | 896 | 7.73480663 | 1.619278491 | 0.013822 | 1 | 0.033374 | 0.007747 | HOXA5,FST,COX4I1,GSTP1,CAPRIN2,OLR1,IFIT3,PSMD14,FGF7,DCK,ABLIM1,MYOT,CPNE4,STUB1,BCL7C,POLE3,SRI,CKM,EDNRA,PSMD10,MMP7,SNX1,FBP1,GBE1,SSPN,RTN4,DGUOK,ALDH9A1 |
| Spermatozoa | 6 | 99 | 1.657458564 | 3.592218696 | 0.014732 | 1 | 0.035357 | 0.008207 | CCNB1,ROPN1L,NME1,CKM,SAR1B,VCP |
| Heart muscle | 143 | 6141 | 39.50276243 | 1.166534202 | 0.016084 | 1 | 0.03837 | 0.008887 | CRYM,KCNMB3,HOXA5,EFCAB2,ZHX3,DRG1,PROSC,DNAJB5,AFP,STK33,FHL2,NFE2,STAU2,MRPS7,CALCOCO2,FST,NRAS,TYROBP,RPL10,ERAL1,APOE,COX4I1,CD47,UPP2,HSPA2,GULP1,TM4SF1,GALT,ELOVL1,LYPD1,GSTP1,RPS6KA2,FUBP1,ATAD2,FAM63A,TIMP1,SLC39A9,DLX5,RAP1GDS1,HPX,HLA-DOB,CHD4,ST6GALNAC6,PSMD14,IMPDH2,DHX9,MRTO4,CYB5R1,CDC34,CLDN4,RRAGB,SFN,NHEJ1,STARD10,CAMK4,TEX264,ZNF641,ANAPC11,SPP1,CLGN,SULT1E1,PTS,RBKS,AAK1,CCR10,BAG5,MAPK3,CDCP1,DBNL,TCOF1,GGT6,RPL14,C1QC,C1orf21,MIPOL1,PRKRA,SMYD3,C14orf119,DIABLO,PDLIM3,CKAP2,NAGK,MEIS2,DERL1,HSPB8,SERBP1,CYP2C8,PRPSAP2,MATK,C9,GTF2I,BCL7C,TOM1,PCGF3,PLCB2,PAK6,ANXA6,COASY,RECQL5,ANXA11,ABLIM3,MAPK1,TXNRD1,MLX,TARDBP,RASGEF1A,F8,EDNRA,CETN3,SELENBP1,MMP7,ABCF3,RAB11A,RUVBL1,PAK4,FGFR2,SLC25A10,FMN1,TAF7,SNX9,ZNF655,SET,RPS6,MYLK,VCP,RAC1,MBIP,HSPBP1,AKT3,NUDT18,LHX1,ZMYM3,NPM1,CIAO1,ARHGAP29,KLHL14,CYGB,PDXK,ANXA13,NFKBIA,ALDH9A1,ACVR2B,C8orf37 |
| Granular cell | 2 | 10 | 0.552486188 | 14.34960618 | 0.016241 | 1 | 0.038512 | 0.008887 | VSNL1,APEX1 |
| Sublingual gland | 2 | 10 | 0.552486188 | 14.34960618 | 0.016241 | 1 | 0.038283 | 0.008887 | STATH,FGFR2 |
| Liver | 251 | 11533 | 69.33701657 | 1.086302948 | 0.016549 | 1 | 0.038778 | 0.009002 | CRYM,KCNMB3,HOXA5,EFCAB2,ZHX3,COQ6,ADRB2,DRG1,PROSC,DNAJB5,SRPX2,AFP,STK33,FHL2,NFE2,RAB3B,STAU2,MRPS7,CALCOCO2,FST,NRAS,TYROBP,RPL10,CSRP1,ERAL1,BHMT2,APOE,HLA-DRB5,COX4I1,HPCAL1,METTL8,UPP2,AEBP2,HSPA2,GULP1,TM4SF1,GALT,SP110,ELOVL1,LYPD1,CARHSP1,IGLL1,GSTP1,RPS6KA2,CYBB,CORO1A,FUBP1,ATAD2,FAM63A,CAPRIN2,RPS7,TIMP1,OLR1,PPP2R4,SLC39A9,QDPR,IFIT3,RHOA,LAYN,DLX5,ATG3,HPX,SGK2,HLA-DOB,SPAG16,CHD4,ST6GALNAC6,MRPS25,PSMD14,SLC5A6,IMPDH2,PANK1,GLYAT,XPR1,DHX9,GNB2L1,CDO1,BCAS2,CYB5R1,CDC34,CLDN4,RAB39B,LCAT,VSNL1,CTNNAL1,SFN,ANP32E,FN3K,SEPT1,NHEJ1,NME1,STARD10,ADD1,ACTL6B,CAMK4,RPS15,TEX264,DCK,ZNF641,ANAPC11,DDX58,ARPC3,SPP1,CLGN,SULT1E1,KLF11,HLA-DRB3,EIF4EBP3,PTS,RBKS,CCNH,AAK1,ARHGDIG,APEX1,ZNF761,CCT4,FNIP1,ZNF587,CCR10,ABLIM1,BAG5,MAPK3,TIRAP,CDCP1,ARF5,YWHAB,DBNL,TCOF1,GGT6,RPL14,C1QC,C1orf21,USP15,MORC4,UGDH,ASF1A,MIPOL1,ENOPH1,PTPN11,ZFYVE19,SMYD3,DIABLO,PDLIM3,PI16,CKAP2,NAGK,CPNE4,MEIS2,MCTS1,DERL1,RPL22,SRPK2,HSPB8,ATP6V1E2,HBG2,SERBP1,SPATA7,TK1,MARCKSL1,CYP2C8,STUB1,PRPSAP2,RAB8B,MATK,C9,GTF2I,BCL7C,NDUFA8,DAPP1,TOM1,FBXL3,PCGF3,PLCB2,ANXA6,RPS13,USP14,CLP1,TEAD3,COASY,RECQL5,ANXA11,RNF11,ABLIM3,MAPK1,TXNRD1,POLE3,SRI,MLX,TARDBP,RASGEF1A,MOXD1,F8,EDNRA,TRIML1,PSMD10,LRFN1,LEPROTL1,ALDH1A1,EPB41L3,SAR1B,SELENBP1,MMP7,ABCF3,RAB11A,RUVBL1,TPD52L2,SNX1,PAK4,FGFR2,SLC25A10,FGA,LEMD1,DDC,FMN1,PECAM1,HSPBAP1,SNX9,FBP1,SET,PPP3R1,PRKAR2B,RPS6,MYLK,VCP,RAC1,COG3,MBIP,HSPBP1,AKT3,SRP54,PHKG2,GFM2,ZRANB2,GBE1,FABP5,SSPN,ZMYM3,NPM1,CIAO1,PGM2,ARHGAP29,KLHL14,RTN4,PDXK,RNPEP,ANXA13,DGUOK,ALDH9A1,C8orf37,UBE2O,PEX19 |
| Skin fibroblasts | 8 | 162 | 2.209944751 | 2.814476886 | 0.016692 | 1 | 0.038883 | 0.009026 | CSRP1,TIMP1,QDPR,CCR10,COASY,MOXD1,CKM,GBE1 |
| HPDE6 | 15 | 409 | 4.143646409 | 1.967496277 | 0.018648 | 1 | 0.043184 | 0.010024 | GSTP1,TIMP1,PPP2R4,GNB2L1,SFN,NME1,APEX1,YWHAB,MAPK1,TXNRD1,SET,VCP,FABP5,NPM1,PDXK |
| H2228 | 8 | 166 | 2.209944751 | 2.747165033 | 0.01904 | 1 | 0.043837 | 0.010176 | DOK1,PSMD14,MAPK3,PTPN11,ABLIM3,MAPK1,TXNRD1,GBE1 |
| Oviduct | 2 | 11 | 0.552486188 | 13.17820976 | 0.01959 | 1 | 0.044842 | 0.010247 | LAYN,FGFR2 |
| Enamel pellicle | 1 | 1 | 0.276243094 | 49.6717137 | 0.020064 | 1 | 0.045664 | 0.010247 | STATH |
| Cranial nerve nuclei | 1 | 1 | 0.276243094 | 49.6717137 | 0.020064 | 1 | 0.045403 | 0.010247 | HPX |
| Retinal astrocyte | 1 | 1 | 0.276243094 | 49.6717137 | 0.020064 | 1 | 0.045145 | 0.010247 | TIMP1 |
| Nucleus basalis | 1 | 1 | 0.276243094 | 49.6717137 | 0.020064 | 1 | 0.04489 | 0.010247 | HPX |
| Nuclei of accessory nerves | 1 | 1 | 0.276243094 | 49.6717137 | 0.020064 | 1 | 0.044637 | 0.010247 | HPX |
| Allantois | 1 | 1 | 0.276243094 | 49.6717137 | 0.020064 | 1 | 0.044388 | 0.010247 | ALDH1A1 |
| Ganglia | 1 | 1 | 0.276243094 | 49.6717137 | 0.020064 | 1 | 0.044141 | 0.010247 | RTN4 |
| Lacrimal glands | 16 | 451 | 4.419889503 | 1.894609312 | 0.020371 | 1 | 0.044568 | 0.010346 | GSTP1,CORO1A,TIMP1,PPP2R4,HPX,GNB2L1,SFN,MAPK3,YWHAB,RAB8B,ANXA11,ALDH1A1,SELENBP1,FGA,FBP1,VCP |

**Supplementary Table 5.8: Transcription factor**

| Analysis:Transcription factor |  |  |  |  |  |  |  |  |  |
| --- | --- | --- | --- | --- | --- | --- | --- | --- | --- |
| Name of data set: MG1 vs HC |  |  |  |  |  |  |  |  |  |
| Number of gene in data set: 365 |  |  |  |  |  |  |  |  |  |
| Number of gene mapped to Transcription factor : 321 |  |  |  |  |  |  |  |  |  |
|  |  |  |  |  |  |  |  |  |  |
| Transcription factor | No. of genes  in the data set | No. of genes in the background data set | Percentage of genes | Fold Enrichment | Uncorrected  p-value  (Hypergeometric test) | Corrected  p-value  (Bonferroni method) | Corrected  p-value  (BH method) | Storey and Tibshirani method  q-value | Genes mapped from  input data set |

**Supplementary Table 5.9: Clinical phenotypes**

| Analysis:Clinical phenotypes |  |  |  |  |  |  |  |  |  |
| --- | --- | --- | --- | --- | --- | --- | --- | --- | --- |
| Name of data set: MG1 vs HC |  |  |  |  |  |  |  |  |  |
| Number of gene in data set: 365 |  |  |  |  |  |  |  |  |  |
| Number of gene mapped to Clinical phenotypes : 34 |  |  |  |  |  |  |  |  |  |
|  |  |  |  |  |  |  |  |  |  |
| Clinical phenotypes | No. of genes  in the data set | No. of genes in the background data set | Percentage of genes | Fold Enrichment | Uncorrected  p-value  (Hypergeometric test) | Corrected  p-value  (Bonferroni method) | Corrected  p-value  (BH method) | Storey and Tibshirani method  q-value | Genes mapped from  input data set |
