# Supplementary material for: Evaluation of autoantibody signatures in meningioma patients using human proteome arrays
Source: Oncotarget. 2017 Apr 10;8(35):58443–56. doi: 10.18632/oncotarget.16997 (PMC5601665; doi:10.18632/oncotarget.16997)
Supplement: Supplementary file 13 [file oncotarget-08-58443-s013.docx]

**Supplementary Table 6: Gene set enrichment analysis of MGII vs HC**

**Supplementary Table 6.1: Gene details**

| Name of data set: MG2 vs HC |  |  |  |  |  |  |
| --- | --- | --- | --- | --- | --- | --- |
| No. of mapped items: 86 |  |  |  |  |  |  |
| No. of unmapped items: 18 |  |  |  |  |  |  |
| No. of redundant items: 0 |  |  |  |  |  |  |
| Is data set quantitative :NO |  |  |  |  |  |  |
|  |  |  |  |  |  |  |
| Mapped items: |  |  |  |  |  |  |
| Search Term | Enrtez GeneID | Gene Symbol | Description | Alternate names | Chromosome | Map location |
| CRYM | [1428](http://www.ncbi.nlm.nih.gov/gene/1428) | CRYM | crystallin, mu | DFNA40\|THBP | 16 | 16p12.2 |
| KCNMB3 | [27094](http://www.ncbi.nlm.nih.gov/gene/27094) | KCNMB3 | potassium large conductance calcium-activated channel, subfamily M beta member 3 | BKBETA3\|HBETA3\|KCNMB2\|KCNMBL\|SLOBETA3 | 3 | 3q26.3-q27 |
| EFCAB2 | [84288](http://www.ncbi.nlm.nih.gov/gene/84288) | EFCAB2 | EF-hand calcium binding domain 2 | - | 1 | 1q44 |
| ARPC3 | [10094](http://www.ncbi.nlm.nih.gov/gene/10094) | ARPC3 | actin related protein 2/3 complex, subunit 3, 21kDa | ARC21\|p21-Arc | 12 | 12q24.11 |
| CARHSP1 | [23589](http://www.ncbi.nlm.nih.gov/gene/23589) | CARHSP1 | calcium regulated heat stable protein 1, 24kDa | CRHSP-24\|CSDC1 | 16 | 16p13.2 |
| ADRB2 | [154](http://www.ncbi.nlm.nih.gov/gene/154) | ADRB2 | adrenoceptor beta 2, surface | ADRB2R\|ADRBR\|B2AR\|BAR\|BETA2AR | 5 | 5q31-q32 |
| HOXA5 | [3202](http://www.ncbi.nlm.nih.gov/gene/3202) | HOXA5 | homeobox A5 | HOX1\|HOX1.3\|HOX1C | 7 | 7p15.2 |
| COX4I1 | [1327](http://www.ncbi.nlm.nih.gov/gene/1327) | COX4I1 | cytochrome c oxidase subunit IV isoform 1 | COX4\|COX4-1\|COXIV | 16 | 16q24.1 |
| CCDC28A | [25901](http://www.ncbi.nlm.nih.gov/gene/25901) | CCDC28A | coiled-coil domain containing 28A | C6orf80\|CCRL1AP | 6 | 6q23.1-q24.1 |
| STAT6 | [6778](http://www.ncbi.nlm.nih.gov/gene/6778) | STAT6 | signal transducer and activator of transcription 6, interleukin-4 induced | D12S1644\|IL-4-STAT\|STAT6B\|STAT6C | 12 | 12q13 |
| PAIP1 | [10605](http://www.ncbi.nlm.nih.gov/gene/10605) | PAIP1 | poly(A) binding protein interacting protein 1 | - | 5 | 5p12 |
| PSMD6 | [9861](http://www.ncbi.nlm.nih.gov/gene/9861) | PSMD6 | proteasome (prosome, macropain) 26S subunit, non-ATPase, 6 | Rpn7\|S10\|SGA-113M\|p42A\|p44S10 | 3 | 3p14.1 |
| MAPK3 | [5595](http://www.ncbi.nlm.nih.gov/gene/5595) | MAPK3 | mitogen-activated protein kinase 3 | ERK-1\|ERK1\|ERT2\|HS44KDAP\|HUMKER1A\|P44ERK1\|P44MAPK\|PRKM3\|p44-ERK1\|p44-MAPK | 16 | 16p11.2 |
| DRG1 | [4733](http://www.ncbi.nlm.nih.gov/gene/4733) | DRG1 | developmentally regulated GTP binding protein 1 | NEDD3 | 22 | 22q12.2 |
| GSG1 | [83445](http://www.ncbi.nlm.nih.gov/gene/83445) | GSG1 | germ cell associated 1 | - | 12 | 12p13.1 |
| PRPSAP2 | [5636](http://www.ncbi.nlm.nih.gov/gene/5636) | PRPSAP2 | phosphoribosyl pyrophosphate synthetase-associated protein 2 | PAP41 | 17 | 17p11.2-p12 |
| KRR1 | [11103](http://www.ncbi.nlm.nih.gov/gene/11103) | KRR1 | KRR1, small subunit (SSU) processome component, homolog (yeast) | HRB2\|RIP-1 | 12 | 12q21.2 |
| ZFYVE19 | [84936](http://www.ncbi.nlm.nih.gov/gene/84936) | ZFYVE19 | zinc finger, FYVE domain containing 19 | MPFYVE | 15 | 15q15.1 |
| CCNB1 | [891](http://www.ncbi.nlm.nih.gov/gene/891) | CCNB1 | cyclin B1 | CCNB | 5 | 5q12 |
| CORO1A | [11151](http://www.ncbi.nlm.nih.gov/gene/11151) | CORO1A | coronin, actin binding protein, 1A | CLABP\|CLIPINA\|HCORO1\|IMD8\|TACO\|p57 | 16 | 16p11.2 |
| FST | [10468](http://www.ncbi.nlm.nih.gov/gene/10468) | FST | follistatin | FS | 5 | 5q11.2 |
| MRPL13 | [28998](http://www.ncbi.nlm.nih.gov/gene/28998) | MRPL13 | mitochondrial ribosomal protein L13 | L13\|L13A\|L13mt\|RPL13\|RPML13 | 8 | 8q22.1-q22.3 |
| RNF11 | [26994](http://www.ncbi.nlm.nih.gov/gene/26994) | RNF11 | ring finger protein 11 | SID1669 | 1 | 1p32 |
| PROSC | [11212](http://www.ncbi.nlm.nih.gov/gene/11212) | PROSC | proline synthetase co-transcribed homolog (bacterial) | - | 8 | 8p11.2 |
| EIF4EBP3 | [8637](http://www.ncbi.nlm.nih.gov/gene/8637) | EIF4EBP3 | eukaryotic translation initiation factor 4E binding protein 3 | 4E-BP3\|4EBP3 | 5 | 5q31.3 |
| OIP5 | [11339](http://www.ncbi.nlm.nih.gov/gene/11339) | OIP5 | Opa interacting protein 5 | 5730547N13Rik\|CT86\|LINT-25\|MIS18B\|MIS18beta\|hMIS18beta | 15 | 15q15.1 |
| PRKG1 | [5592](http://www.ncbi.nlm.nih.gov/gene/5592) | PRKG1 | protein kinase, cGMP-dependent, type I | 1\|AAT8\|PKG\|PRKG1B\|PRKGR1B\|cGK\|cGK 1\|cGK1\|cGKI\|cGKI-BETA\|cGKI-alpha | 10 | 10q11.2 |
| CDKN1B | [1027](http://www.ncbi.nlm.nih.gov/gene/1027) | CDKN1B | cyclin-dependent kinase inhibitor 1B (p27, Kip1) | CDKN4\|KIP1\|MEN1B\|MEN4\|P27KIP1 | 12 | 12p13.1-p12 |
| DDI2 | [84301](http://www.ncbi.nlm.nih.gov/gene/84301) | DDI2 | DNA-damage inducible 1 homolog 2 (S. cerevisiae) | - | 1 | 1p36.21 |
| DTD1 | [92675](http://www.ncbi.nlm.nih.gov/gene/92675) | DTD1 | D-tyrosyl-tRNA deacylase 1 | C20orf88\|DUE-B\|DUEB\|HARS2\|bA379J5.3\|bA555E18.1\|pqn-68 | 20 | 20p11.23 |
| SLC39A9 | [55334](http://www.ncbi.nlm.nih.gov/gene/55334) | SLC39A9 | solute carrier family 39, member 9 | ZIP-9\|ZIP9 | 14 | 14q24.1 |
| LCN1 | [3933](http://www.ncbi.nlm.nih.gov/gene/3933) | LCN1 | lipocalin 1 | PMFA\|TLC\|TP\|VEGP | 9 | 9q34 |
| RAB3B | [5865](http://www.ncbi.nlm.nih.gov/gene/5865) | RAB3B | RAB3B, member RAS oncogene family | - | 1 | 1p32-p31 |
| DOK1 | [1796](http://www.ncbi.nlm.nih.gov/gene/1796) | DOK1 | docking protein 1, 62kDa (downstream of tyrosine kinase 1) | P62DOK | 2 | 2p13 |
| HSD17B14 | [51171](http://www.ncbi.nlm.nih.gov/gene/51171) | HSD17B14 | hydroxysteroid (17-beta) dehydrogenase 14 | DHRS10\|SDR47C1\|retSDR3 | 19 | 19q13.33 |
| UBE2V2 | [7336](http://www.ncbi.nlm.nih.gov/gene/7336) | UBE2V2 | ubiquitin-conjugating enzyme E2 variant 2 | DDVIT1\|DDVit-1\|EDAF-1\|EDPF-1\|EDPF1\|MMS2\|UEV-2\|UEV2 | 8 | 8q11.21 |
| BLOC1S2 | [282991](http://www.ncbi.nlm.nih.gov/gene/282991) | BLOC1S2 | biogenesis of lysosomal organelles complex-1, subunit 2 | BLOS2\|CEAP\|CEAP11 | 10 | 10q24.31 |
| AIF1 | [199](http://www.ncbi.nlm.nih.gov/gene/199) | AIF1 | allograft inflammatory factor 1 | AIF-1\|IBA1\|IRT-1\|IRT1 | 6 | 6p21.3 |
| CALCOCO2 | [10241](http://www.ncbi.nlm.nih.gov/gene/10241) | CALCOCO2 | calcium binding and coiled-coil domain 2 | NDP52 | 17 | 17q21.32 |
| SGCG | [6445](http://www.ncbi.nlm.nih.gov/gene/6445) | SGCG | sarcoglycan, gamma (35kDa dystrophin-associated glycoprotein) | A4\|DAGA4\|DMDA\|DMDA1\|LGMD2C\|MAM\|SCARMD2\|SCG3\|TYPE | 13 | 13q12 |
| EPS8L1 | [54869](http://www.ncbi.nlm.nih.gov/gene/54869) | EPS8L1 | EPS8-like 1 | DRC3\|EPS8R1 | 19 | 19q13.42 |
| RBP1 | [5947](http://www.ncbi.nlm.nih.gov/gene/5947) | RBP1 | retinol binding protein 1, cellular | CRABP-I\|CRBP\|CRBP1\|CRBPI\|RBPC | 3 | 3q23 |
| KLK1 | [3816](http://www.ncbi.nlm.nih.gov/gene/3816) | KLK1 | kallikrein 1 | KLKR\|Klk6\|hK1 | 19 | 19q13.3 |
| MIPOL1 | [145282](http://www.ncbi.nlm.nih.gov/gene/145282) | MIPOL1 | mirror-image polydactyly 1 | - | 14 | 14q13.3 |
| RNF25 | [64320](http://www.ncbi.nlm.nih.gov/gene/64320) | RNF25 | ring finger protein 25 | AO7 | 2 | 2q35 |
| DLC1 | [10395](http://www.ncbi.nlm.nih.gov/gene/10395) | DLC1 | DLC1 Rho GTPase activating protein | ARHGAP7\|HP\|STARD12\|p122-RhoGAP | 8 | 8p22 |
| TRIM68 | [55128](http://www.ncbi.nlm.nih.gov/gene/55128) | TRIM68 | tripartite motif containing 68 | GC109\|RNF137\|SS-56\|SS56 | 11 | 11p15.4 |
| CCT4 | [10575](http://www.ncbi.nlm.nih.gov/gene/10575) | CCT4 | chaperonin containing TCP1, subunit 4 (delta) | CCT-DELTA\|Cctd\|SRB | 2 | 2p15 |
| HPCAL1 | [3241](http://www.ncbi.nlm.nih.gov/gene/3241) | HPCAL1 | hippocalcin-like 1 | BDR1\|HLP2\|VILIP-3 | 2 | 2p25.1 |
| RHOA | [387](http://www.ncbi.nlm.nih.gov/gene/387) | RHOA | ras homolog family member A | ARH12\|ARHA\|RHO12\|RHOH12 | 3 | 3p21.3 |
| C1QTNF7 | [114905](http://www.ncbi.nlm.nih.gov/gene/114905) | C1QTNF7 | C1q and tumor necrosis factor related protein 7 | CTRP7\|ZACRP7 | 4 | 4p15.3 |
| PRKRA | [8575](http://www.ncbi.nlm.nih.gov/gene/8575) | PRKRA | protein kinase, interferon-inducible double stranded RNA dependent activator | DYT16\|PACT\|RAX | 2 | 2q31.2 |
| FAIM | [55179](http://www.ncbi.nlm.nih.gov/gene/55179) | FAIM | Fas apoptotic inhibitory molecule | FAIM1 | 3 | 3q22.3 |
| TIRAP | [114609](http://www.ncbi.nlm.nih.gov/gene/114609) | TIRAP | toll-interleukin 1 receptor (TIR) domain containing adaptor protein | BACTS1\|Mal\|MyD88-2\|wyatt | 11 | 11q24.2 |
| DOHH | [83475](http://www.ncbi.nlm.nih.gov/gene/83475) | DOHH | deoxyhypusine hydroxylase/monooxygenase | HLRC1\|hDOHH | 19 | 19p13.3 |
| SULT1E1 | [6783](http://www.ncbi.nlm.nih.gov/gene/6783) | SULT1E1 | sulfotransferase family 1E, estrogen-preferring, member 1 | EST\|EST-1\|ST1E1\|STE | 4 | 4q13.1 |
| PPP2R4 | [5524](http://www.ncbi.nlm.nih.gov/gene/5524) | PPP2R4 | protein phosphatase 2A activator, regulatory subunit 4 | PP2A\|PR53\|PTPA | 9 | 9q34 |
| CKS2 | [1164](http://www.ncbi.nlm.nih.gov/gene/1164) | CKS2 | CDC28 protein kinase regulatory subunit 2 | CKSHS2 | 9 | 9q22 |
| GPSM3 | [63940](http://www.ncbi.nlm.nih.gov/gene/63940) | GPSM3 | G-protein signaling modulator 3 | AGS4\|C6orf9\|G18\|G18.1a\|G18.1b\|G18.2\|NG1 | 6 | 6p21.3 |
| GYPE | [2996](http://www.ncbi.nlm.nih.gov/gene/2996) | GYPE | glycophorin E (MNS blood group) | GPE\|MNS\|MiIX | 4 | 4q31.1 |
| TMEM185B | [79134](http://www.ncbi.nlm.nih.gov/gene/79134) | TMEM185B | transmembrane protein 185B | FAM11B | 2 | 2q14.2 |
| POLR3B | [55703](http://www.ncbi.nlm.nih.gov/gene/55703) | POLR3B | polymerase (RNA) III (DNA directed) polypeptide B | C128\|HLD8\|RPC2 | 12 | 12q23.3 |
| ABLIM1 | [3983](http://www.ncbi.nlm.nih.gov/gene/3983) | ABLIM1 | actin binding LIM protein 1 | ABLIM\|LIMAB1\|LIMATIN\|abLIM-1 | 10 | 10q25 |
| MYL2 | [4633](http://www.ncbi.nlm.nih.gov/gene/4633) | MYL2 | myosin, light chain 2, regulatory, cardiac, slow | CMH10\|MLC2 | 12 | 12q24.11 |
| UGDH | [7358](http://www.ncbi.nlm.nih.gov/gene/7358) | UGDH | UDP-glucose 6-dehydrogenase | GDH\|UDP-GlcDH\|UDPGDH\|UGD | 4 | 4p15.1 |
| OR10G3 | [26533](http://www.ncbi.nlm.nih.gov/gene/26533) | OR10G3 | olfactory receptor, family 10, subfamily G, member 3 | OR14-40 | 14 | 14q11.2 |
| SNX1 | [6642](http://www.ncbi.nlm.nih.gov/gene/6642) | SNX1 | sorting nexin 1 | HsT17379\|VPS5 | 15 | 15q22.31 |
| RAB11A | [8766](http://www.ncbi.nlm.nih.gov/gene/8766) | RAB11A | RAB11A, member RAS oncogene family | YL8 | 15 | 15q22.31 |
| ATF6 | [22926](http://www.ncbi.nlm.nih.gov/gene/22926) | ATF6 | activating transcription factor 6 | ATF6A | 1 | 1q22-q23 |
| HBG1 | [3047](http://www.ncbi.nlm.nih.gov/gene/3047) | HBG1 | hemoglobin, gamma A | HBG-T2\|HBGA\|HBGR\|HSGGL1 | 11 | 11p15.5 |
| CTH | [1491](http://www.ncbi.nlm.nih.gov/gene/1491) | CTH | cystathionine gamma-lyase | - | 1 | 1p31.1 |
| NMRAL1 | [57407](http://www.ncbi.nlm.nih.gov/gene/57407) | NMRAL1 | NmrA-like family domain containing 1 | HSCARG\|SDR48A1 | 16 | 16p13.3 |
| SAT1 | [6303](http://www.ncbi.nlm.nih.gov/gene/6303) | SAT1 | spermidine/spermine N1-acetyltransferase 1 | DC21\|KFSD\|KFSDX\|SAT\|SSAT\|SSAT-1 | X | Xp22.1 |
| RPUSD2 | [27079](http://www.ncbi.nlm.nih.gov/gene/27079) | RPUSD2 | RNA pseudouridylate synthase domain containing 2 | C15orf19\|C18B11 | 15 | 15q13.3 |
| DYNLT3 | [6990](http://www.ncbi.nlm.nih.gov/gene/6990) | DYNLT3 | dynein, light chain, Tctex-type 3 | RP3\|TCTE1L\|TCTEX1L | X | Xp21 |
| MTL5 | [9633](http://www.ncbi.nlm.nih.gov/gene/9633) | MTL5 | metallothionein-like 5, testis-specific (tesmin) | CXCDC2\|MTLT\|TESMIN | 11 | 11q13.2-q13.3 |
| TEAD3 | [7005](http://www.ncbi.nlm.nih.gov/gene/7005) | TEAD3 | TEA domain family member 3 | DTEF-1\|ETFR-1\|TEAD-3\|TEAD5\|TEF-5\|TEF5 | 6 | 6p21.2 |
| HBG2 | [3048](http://www.ncbi.nlm.nih.gov/gene/3048) | HBG2 | hemoglobin, gamma G | HBG-T1\|TNCY | 11 | 11p15.5 |
| Dlx5 | [1749](http://www.ncbi.nlm.nih.gov/gene/1749) | DLX5 | distal-less homeobox 5 | SHFM1D | 7 | 7q22 |
| CDC34 | [997](http://www.ncbi.nlm.nih.gov/gene/997) | CDC34 | cell division cycle 34 | E2-CDC34\|UBC3\|UBCH3\|UBE2R1 | 19 | 19p13.3 |
| Nol3 | [8996](http://www.ncbi.nlm.nih.gov/gene/8996) | NOL3 | nucleolar protein 3 (apoptosis repressor with CARD domain) | ARC\|FCM\|MYP\|NOP\|NOP30 | 16 | 16q22.1 |
| TIPIN | [54962](http://www.ncbi.nlm.nih.gov/gene/54962) | TIPIN | TIMELESS interacting protein | - | 15 | 15q22.31 |
| LRFN1 | [57622](http://www.ncbi.nlm.nih.gov/gene/57622) | LRFN1 | leucine rich repeat and fibronectin type III domain containing 1 | SALM2 | 19 | 19q13.2 |
| P2RX7 | [5027](http://www.ncbi.nlm.nih.gov/gene/5027) | P2RX7 | purinergic receptor P2X, ligand-gated ion channel, 7 | P2X7 | 12 | 12q24 |
| PAIP2 | [51247](http://www.ncbi.nlm.nih.gov/gene/51247) | PAIP2 | poly(A) binding protein interacting protein 2 | PAIP-2\|PAIP2A | 5 | 5q31.2 |
| ZHX3 | [23051](http://www.ncbi.nlm.nih.gov/gene/23051) | ZHX3 | zinc fingers and homeoboxes 3 | TIX1 | 20 | 20q12 |
|  |  |  |  |  |  |  |
|  |  |  |  |  |  |  |
| Unmapped Entries |  |  |  |  |  |  |
|  | IGHG4 |  |  |  |  |  |
|  | C20orf112 |  |  |  |  |  |
|  | RY1 |  |  |  |  |  |
|  | C17orf57 |  |  |  |  |  |
|  | SURB7 |  |  |  |  |  |
|  | LOC389833 |  |  |  |  |  |
|  | HDAC7A |  |  |  |  |  |
|  | IGHG1 |  |  |  |  |  |
|  | NA |  |  |  |  |  |
|  | IL1F7 |  |  |  |  |  |
|  | LOC285382 |  |  |  |  |  |
|  | IGHG1 |  |  |  |  |  |
|  | JUB |  |  |  |  |  |
|  | HCG3 |  |  |  |  |  |
|  | FAM105B |  |  |  |  |  |
|  | LOC339803 |  |  |  |  |  |
|  | MRVI1-AS1 |  |  |  |  |  |
|  | KIAA0174 |  |  |  |  |  |

**Supplementary Table 6.2: Cellular Component**

| Analysis:Cellular component |  |  |  |  |  |  |  |  |  |
| --- | --- | --- | --- | --- | --- | --- | --- | --- | --- |
| Name of data set: MG2 vs HC |  |  |  |  |  |  |  |  |  |
| Number of gene in data set: 86 |  |  |  |  |  |  |  |  |  |
| Number of gene mapped to Cellular component : 74 |  |  |  |  |  |  |  |  |  |
|  |  |  |  |  |  |  |  |  |  |
| Cellular component | No. of genes  in the data set | No. of genes in the background data set | Percentage of genes | Fold Enrichment | Uncorrected  p-value  (Hypergeometric test) | Corrected  p-value  (Bonferroni method) | Corrected  p-value  (BH method) | Storey and Tibshirani method  q-value | Genes mapped from  input data set |
| Cytoplasm | 47 | 5632 | 63.51351351 | 1.638858651 | 1.82E-05 | 0.001257 | 0.001257 | 0.00041 | CRYM,ARPC3,CARHSP1,COX4I1,STAT6,PAIP1,MAPK3,DRG1,PRPSAP2,ZFYVE19,CCNB1,CORO1A,RNF11,PROSC,EIF4EBP3,CDKN1B,DDI2,DTD1,RAB3B,DOK1,UBE2V2,AIF1,CALCOCO2,RBP1,RNF25,DLC1,TRIM68,CCT4,HPCAL1,RHOA,PRKRA,TIRAP,SULT1E1,PPP2R4,ABLIM1,MYL2,SNX1,RAB11A,HBG1,CTH,SAT1,MTL5,DLX5,CDC34,NOL3,PAIP2,ZHX3 |

**Supplementary Table 6.3: Molecular Function**

| Analysis:Molecular function |  |  |  |  |  |  |  |  |  |
| --- | --- | --- | --- | --- | --- | --- | --- | --- | --- |
| Name of data set: MG2 vs HC |  |  |  |  |  |  |  |  |  |
| Number of gene in data set: 86 |  |  |  |  |  |  |  |  |  |
| Number of gene mapped to Molecular function : 85 |  |  |  |  |  |  |  |  |  |
|  |  |  |  |  |  |  |  |  |  |
| Molecular function | No. of genes  in the data set | No. of genes in the background data set | Percentage of genes | Fold Enrichment | Uncorrected  p-value  (Hypergeometric test) | Corrected  p-value  (Bonferroni method) | Corrected  p-value  (BH method) | Storey and Tibshirani method  q-value | Genes mapped from  input data set |

**Supplementary Table 6.4: Biological Process**

| Analysis:Biological process |  |  |  |  |  |  |  |  |  |
| --- | --- | --- | --- | --- | --- | --- | --- | --- | --- |
| Name of data set: MG2 vs HC |  |  |  |  |  |  |  |  |  |
| Number of gene in data set: 86 |  |  |  |  |  |  |  |  |  |
| Number of gene mapped to Biological process : 85 |  |  |  |  |  |  |  |  |  |
|  |  |  |  |  |  |  |  |  |  |
| Biological process | No. of genes  in the data set | No. of genes in the background data set | Percentage of genes | Fold Enrichment | Uncorrected  p-value  (Hypergeometric test) | Corrected  p-value  (Bonferroni method) | Corrected  p-value  (BH method) | Storey and Tibshirani method  q-value | Genes mapped from  input data set |

**Supplementary Table 6.5: Biological Pathway**

| Analysis:Biological pathway |  |  |  |  |  |  |  |  |  |
| --- | --- | --- | --- | --- | --- | --- | --- | --- | --- |
| Name of data set: MG2 vs HC |  |  |  |  |  |  |  |  |  |
| Number of gene in data set: 86 |  |  |  |  |  |  |  |  |  |
| Number of gene mapped to Biological pathway : 40 |  |  |  |  |  |  |  |  |  |
|  |  |  |  |  |  |  |  |  |  |
| Biological pathway | No. of genes  in the data set | No. of genes in the background data set | Percentage of genes | Fold Enrichment | Uncorrected  p-value  (Hypergeometric test) | Corrected  p-value  (Bonferroni method) | Corrected  p-value  (BH method) | Storey and Tibshirani method  q-value | Genes mapped from  input data set |
| Regulation of RAC1 activity | 7 | 204 | 17.5 | 5.845797808 | 0.000341 | 0.13657 | 0.045523 | 0.00318 | ARPC3,MAPK3,CDKN1B,DLC1,RHOA,MYL2,HBG2 |
| RAC1 signaling pathway | 7 | 204 | 17.5 | 5.845797808 | 0.000341 | 0.13657 | 0.034142 | 0.00318 | ARPC3,MAPK3,CDKN1B,DLC1,RHOA,MYL2,HBG2 |

**Supplementary Table 6.6: Protein Domain**

| Analysis:Protein domain |  |  |  |  |  |  |  |  |  |
| --- | --- | --- | --- | --- | --- | --- | --- | --- | --- |
| Name of data set: MG2 vs HC |  |  |  |  |  |  |  |  |  |
| Number of gene in data set: 86 |  |  |  |  |  |  |  |  |  |
| Number of gene mapped to Protein domain : 23 |  |  |  |  |  |  |  |  |  |
|  |  |  |  |  |  |  |  |  |  |
| Protein domain | No. of genes  in the data set | No. of genes in the background data set | Percentage of genes | Fold Enrichment | Uncorrected  p-value  (Hypergeometric test) | Corrected  p-value  (Bonferroni method) | Corrected  p-value  (BH method) | Storey and Tibshirani method  q-value | Genes mapped from  input data set |

**Supplementary Table 6.7: Site of Expression**

| Analysis:Site of expression |  |  |  |  |  |  |  |  |  |
| --- | --- | --- | --- | --- | --- | --- | --- | --- | --- |
| Name of data set: MG2 vs HC |  |  |  |  |  |  |  |  |  |
| Number of gene in data set: 86 |  |  |  |  |  |  |  |  |  |
| Number of gene mapped to Site of expression : 85 |  |  |  |  |  |  |  |  |  |
|  |  |  |  |  |  |  |  |  |  |
| Site of expression | No. of genes  in the data set | No. of genes in the background data set | Percentage of genes | Fold Enrichment | Uncorrected  p-value  (Hypergeometric test) | Corrected  p-value  (Bonferroni method) | Corrected  p-value  (BH method) | Storey and Tibshirani method  q-value | Genes mapped from  input data set |
| OVCAR3 | 35 | 3644 | 41.17647059 | 2.08034975 | 7.91E-06 | 0.002087 | 0.002087 | 0.00053 | ARPC3,CARHSP1,ADRB2,COX4I1,CCDC28A,PAIP1,PSMD6,MAPK3,DRG1,PRPSAP2,CCNB1,CORO1A,FST,MRPL13,PROSC,DDI2,DTD1,SLC39A9,UBE2V2,BLOC1S2,EPS8L1,RBP1,CCT4,HPCAL1,PRKRA,DOHH,PPP2R4,CKS2,ABLIM1,UGDH,SNX1,RAB11A,RPUSD2,NOL3,PAIP2 |
| ES2 | 33 | 3469 | 38.82352941 | 2.064692954 | 2.09E-05 | 0.005527 | 0.002763 | 0.000702 | ARPC3,CARHSP1,HOXA5,COX4I1,PAIP1,PSMD6,MAPK3,DRG1,KRR1,ZFYVE19,CCNB1,FST,MRPL13,PROSC,EIF4EBP3,CDKN1B,DTD1,SLC39A9,RAB3B,UBE2V2,RNF25,CCT4,HPCAL1,PRKRA,DOHH,PPP2R4,CKS2,UGDH,SNX1,RAB11A,HBG1,CTH,HBG2 |
| CRC | 35 | 4170 | 41.17647059 | 1.818014966 | 0.000155 | 0.040868 | 0.013623 | 0.003462 | ARPC3,CARHSP1,COX4I1,PAIP1,PSMD6,MAPK3,DRG1,CORO1A,MRPL13,PROSC,DDI2,DTD1,RAB3B,UBE2V2,BLOC1S2,SGCG,RBP1,KLK1,DLC1,CCT4,HPCAL1,RHOA,C1QTNF7,PRKRA,PPP2R4,UGDH,SNX1,RAB11A,HBG1,CTH,DYNLT3,TEAD3,HBG2,NOL3,PAIP2 |
| H293 | 48 | 6698 | 56.47058824 | 1.537926969 | 0.000217 | 0.057191 | 0.014298 | 0.003618 | ARPC3,CARHSP1,HOXA5,COX4I1,STAT6,PAIP1,PSMD6,MAPK3,DRG1,PRPSAP2,KRR1,ZFYVE19,CCNB1,CORO1A,MRPL13,RNF11,PROSC,EIF4EBP3,OIP5,CDKN1B,DDI2,DTD1,SLC39A9,HSD17B14,UBE2V2,BLOC1S2,RNF25,CCT4,HPCAL1,PRKRA,DOHH,PPP2R4,CKS2,POLR3B,ABLIM1,UGDH,SNX1,RAB11A,HBG1,CTH,RPUSD2,DYNLT3,HBG2,CDC34,NOL3,TIPIN,PAIP2,ZHX3 |
| HCT116 | 25 | 2596 | 29.41176471 | 2.114322423 | 0.00027 | 0.07119 | 0.014238 | 0.003618 | ARPC3,CARHSP1,COX4I1,PAIP1,PSMD6,MAPK3,DRG1,PRPSAP2,KRR1,PROSC,DTD1,RAB3B,UBE2V2,RBP1,CCT4,RHOA,PRKRA,FAIM,DOHH,PPP2R4,UGDH,SNX1,RAB11A,HBG1,HBG2 |
| Erythrocytes | 17 | 1492 | 20 | 2.556736716 | 0.000515 | 0.135838 | 0.02264 | 0.005753 | CARHSP1,STAT6,PAIP1,PSMD6,PRPSAP2,ZFYVE19,PROSC,DDI2,DTD1,CCT4,HPCAL1,DOHH,PPP2R4,SNX1,HBG1,HBG2,CDC34 |
| global_SCX_fractionated | 23 | 2427 | 27.05882353 | 2.089171094 | 0.000648 | 0.171053 | 0.024436 | 0.006039 | ARPC3,CARHSP1,COX4I1,PAIP1,PSMD6,PRPSAP2,ZFYVE19,CORO1A,MRPL13,PROSC,EIF4EBP3,DTD1,BLOC1S2,CCT4,HPCAL1,PRKRA,DOHH,SULT1E1,PPP2R4,UGDH,SNX1,RAB11A,DYNLT3 |
| MDA-MB-468 | 11 | 744 | 12.94117647 | 3.438664197 | 0.00072 | 0.190184 | 0.023773 | 0.006039 | COX4I1,PSMD6,CORO1A,MRPL13,PROSC,EIF4EBP3,DTD1,RBP1,CCT4,RHOA,RAB11A |
| CaOV3 | 28 | 3291 | 32.94117647 | 1.858611958 | 0.00081 | 0.213892 | 0.023766 | 0.006039 | ARPC3,CARHSP1,PAIP1,PSMD6,MAPK3,DRG1,KRR1,MRPL13,PROSC,DDI2,DTD1,RAB3B,UBE2V2,BLOC1S2,EPS8L1,RBP1,CCT4,HPCAL1,PRKRA,DOHH,PPP2R4,CKS2,UGDH,SNX1,RAB11A,CTH,NOL3,PAIP2 |
| B Cell | 21 | 2194 | 24.70588235 | 2.120321379 | 0.001046 | 0.276253 | 0.027625 | 0.00702 | ARPC3,COX4I1,PAIP1,MAPK3,DRG1,CORO1A,PROSC,LCN1,RAB3B,UBE2V2,BLOC1S2,CCT4,HPCAL1,RHOA,DOHH,PPP2R4,GPSM3,UGDH,HBG1,RPUSD2,HBG2 |
| Melanoma | 18 | 1795 | 21.17647059 | 2.241917441 | 0.001532 | 0.40442 | 0.036765 | 0.008756 | ARPC3,COX4I1,PSMD6,DRG1,CORO1A,DTD1,AIF1,CALCOCO2,CCT4,HPCAL1,RHOA,PRKRA,PPP2R4,UGDH,SNX1,RAB11A,HBG1,NOL3 |
| CD8 | 27 | 3259 | 31.76470588 | 1.812688633 | 0.001577 | 0.41635 | 0.034696 | 0.008756 | ARPC3,CARHSP1,COX4I1,STAT6,PSMD6,MAPK3,DRG1,PRPSAP2,CCNB1,CORO1A,MRPL13,PROSC,EIF4EBP3,UBE2V2,AIF1,CCT4,HPCAL1,DOHH,PPP2R4,GPSM3,UGDH,SNX1,HBG1,CTH,RPUSD2,DYNLT3,HBG2 |
| 031003_BALF2 | 13 | 1090 | 15.29411765 | 2.731783455 | 0.001697 | 0.447939 | 0.034457 | 0.008756 | PAIP1,MAPK3,CORO1A,BLOC1S2,CCT4,DOHH,PPP2R4,UGDH,HBG1,RPUSD2,DYNLT3,HBG2,NOL3 |
| Colon | 53 | 8358 | 62.35294118 | 1.357650836 | 0.002102 | 0.554994 | 0.039642 | 0.010074 | CRYM,KCNMB3,EFCAB2,ARPC3,ADRB2,HOXA5,COX4I1,STAT6,PSMD6,MAPK3,DRG1,PRPSAP2,CCNB1,FST,RNF11,PROSC,EIF4EBP3,PRKG1,CDKN1B,DTD1,SLC39A9,RAB3B,HSD17B14,UBE2V2,AIF1,CALCOCO2,SGCG,RBP1,MIPOL1,DLC1,TRIM68,CCT4,RHOA,C1QTNF7,PRKRA,TIRAP,SULT1E1,PPP2R4,ABLIM1,MYL2,UGDH,SNX1,RAB11A,HBG1,CTH,DYNLT3,TEAD3,HBG2,DLX5,CDC34,NOL3,PAIP2,ZHX3 |

**Supplementary Table 6.8: Transcription Factor**

| Analysis:Transcription factor |  |  |  |  |  |  |  |  |  |
| --- | --- | --- | --- | --- | --- | --- | --- | --- | --- |
| Name of data set: MG2 vs HC |  |  |  |  |  |  |  |  |  |
| Number of gene in data set: 86 |  |  |  |  |  |  |  |  |  |
| Number of gene mapped to Transcription factor : 72 |  |  |  |  |  |  |  |  |  |
|  |  |  |  |  |  |  |  |  |  |
| Transcription factor | No. of genes  in the data set | No. of genes in the background data set | Percentage of genes | Fold Enrichment | Uncorrected  p-value  (Hypergeometric test) | Corrected  p-value  (Bonferroni method) | Corrected  p-value  (BH method) | Storey and Tibshirani method  q-value | Genes mapped from  input data set |

**Supplementary Table 6.9: Clinical Phenotypes**

| Analysis:Clinical phenotypes |  |  |  |  |  |  |  |  |  |
| --- | --- | --- | --- | --- | --- | --- | --- | --- | --- |
| Name of data set: MG2 vs HC |  |  |  |  |  |  |  |  |  |
| Number of gene in data set: 86 |  |  |  |  |  |  |  |  |  |
| Number of gene mapped to Clinical phenotypes : 8 |  |  |  |  |  |  |  |  |  |
|  |  |  |  |  |  |  |  |  |  |
| Clinical phenotypes | No. of genes  in the data set | No. of genes in the background data set | Percentage of genes | Fold Enrichment | Uncorrected  p-value  (Hypergeometric test) | Corrected  p-value  (Bonferroni method) | Corrected  p-value  (BH method) | Storey and Tibshirani method  q-value | Genes mapped from  input data set |
| Persistence of fetal hemoglobin (5-30% HbF) | 2 | 3 | 25 | 150.2337043 | 5.09E-05 | 0.00651 | 0.00651 | 0.001969 | HBG1,HBG2 |
| Mental retardation, but majority of patients are normal | 1 | 1 | 12.5 | 196.4594595 | 0.004405 | 0.563877 | 0.04699 | 0.003875 | CTH |
| Muscle biopsy shows dystrophic pattern | 1 | 1 | 12.5 | 196.4594595 | 0.004405 | 0.563877 | 0.043375 | 0.003875 | SGCG |
| Normal dystrophin immunostaining | 1 | 1 | 12.5 | 196.4594595 | 0.004405 | 0.563877 | 0.040277 | 0.003875 | SGCG |
| Normal lower limbs (in some patients) | 1 | 1 | 12.5 | 196.4594595 | 0.004405 | 0.563877 | 0.037592 | 0.003875 | DLX5 |
| Onset 1-12 years | 1 | 1 | 12.5 | 196.4594595 | 0.004405 | 0.563877 | 0.035242 | 0.003875 | SGCG |
| Patchy muscle fiber degeneration | 1 | 1 | 12.5 | 196.4594595 | 0.004405 | 0.563877 | 0.033169 | 0.003875 | SGCG |
| Polydactyly, preaxial or postaxial | 1 | 1 | 12.5 | 196.4594595 | 0.004405 | 0.563877 | 0.031326 | 0.003875 | MIPOL1 |
| Prevalent in North Africa | 1 | 1 | 12.5 | 196.4594595 | 0.004405 | 0.563877 | 0.029678 | 0.003875 | SGCG |
| Progressive proximal muscle involvement | 1 | 1 | 12.5 | 196.4594595 | 0.004405 | 0.563877 | 0.028194 | 0.003875 | SGCG |
| Restriction of flexion at all metacarpophalangeal and interphalangeal joints (in some patients) | 1 | 1 | 12.5 | 196.4594595 | 0.004405 | 0.563877 | 0.026851 | 0.003875 | DLX5 |
| Right ventricular dilatation | 1 | 1 | 12.5 | 196.4594595 | 0.004405 | 0.563877 | 0.025631 | 0.003875 | SGCG |
| Scoliosis, mild (rare) | 1 | 1 | 12.5 | 196.4594595 | 0.004405 | 0.563877 | 0.024516 | 0.003875 | DLX5 |
| Short stature, severe (in some patients) | 1 | 1 | 12.5 | 196.4594595 | 0.004405 | 0.563877 | 0.023495 | 0.003875 | DLX5 |
| Subclinical cardiac involvement in a subset of patients | 1 | 1 | 12.5 | 196.4594595 | 0.004405 | 0.563877 | 0.022555 | 0.003875 | SGCG |
| Tapered fingers (in some patients) | 1 | 1 | 12.5 | 196.4594595 | 0.004405 | 0.563877 | 0.021688 | 0.003875 | DLX5 |
| Ulnar duplication | 1 | 1 | 12.5 | 196.4594595 | 0.004405 | 0.563877 | 0.020884 | 0.003875 | MIPOL1 |
| Unstable gait | 1 | 1 | 12.5 | 196.4594595 | 0.004405 | 0.563877 | 0.020138 | 0.003875 | SGCG |
| Wheelchair use by 10-30 years | 1 | 1 | 12.5 | 196.4594595 | 0.004405 | 0.563877 | 0.019444 | 0.003875 | SGCG |
| Dysplastic carpal bones | 1 | 1 | 12.5 | 196.4594595 | 0.004405 | 0.563877 | 0.018796 | 0.003875 | MIPOL1 |
| Duplication of bones of the hand | 1 | 1 | 12.5 | 196.4594595 | 0.004405 | 0.563877 | 0.01819 | 0.003875 | MIPOL1 |
| Zeugopodial duplication, symmetrical | 1 | 1 | 12.5 | 196.4594595 | 0.004405 | 0.563877 | 0.017621 | 0.003875 | MIPOL1 |
| Dorsalization of palms (in some patients) | 1 | 1 | 12.5 | 196.4594595 | 0.004405 | 0.563877 | 0.017087 | 0.003875 | DLX5 |
| Dislocation of the patella | 1 | 1 | 12.5 | 196.4594595 | 0.004405 | 0.563877 | 0.016585 | 0.003875 | MIPOL1 |
| Absence of gamma-sarcoglycan protein | 1 | 1 | 12.5 | 196.4594595 | 0.004405 | 0.563877 | 0.016111 | 0.003875 | SGCG |
| Duplication of bones of the feet | 1 | 1 | 12.5 | 196.4594595 | 0.004405 | 0.563877 | 0.015663 | 0.003875 | MIPOL1 |
| Cup-shaped hands | 1 | 1 | 12.5 | 196.4594595 | 0.004405 | 0.563877 | 0.01524 | 0.003875 | MIPOL1 |
| Absent tibia | 1 | 1 | 12.5 | 196.4594595 | 0.004405 | 0.563877 | 0.014839 | 0.003875 | MIPOL1 |
| Autopodial duplication, symmetrical | 1 | 1 | 12.5 | 196.4594595 | 0.004405 | 0.563877 | 0.014458 | 0.003875 | MIPOL1 |
| Asymmetric short and severely deformed legs (in some patients) | 1 | 1 | 12.5 | 196.4594595 | 0.004405 | 0.563877 | 0.014097 | 0.003875 | DLX5 |
| Cylindrical nails (in some patients) | 1 | 1 | 12.5 | 196.4594595 | 0.004405 | 0.563877 | 0.013753 | 0.003875 | DLX5 |
| Asymmetrical severely deformed feet (in some patients) | 1 | 1 | 12.5 | 196.4594595 | 0.004405 | 0.563877 | 0.013426 | 0.003875 | DLX5 |
| Cleft nares, bilateral | 1 | 1 | 12.5 | 196.4594595 | 0.004405 | 0.563877 | 0.013113 | 0.003875 | MIPOL1 |
| CYSTATHIONINURIA | 1 | 1 | 12.5 | 196.4594595 | 0.004405 | 0.563877 | 0.012815 | 0.003875 | CTH |
| Dry, bumpy skin | 1 | 2 | 12.5 | 136.010395 | 0.008794 | 1 | 0.025013 | 0.005868 | SAT1 |
| Perifollicular fibrosis | 1 | 2 | 12.5 | 136.010395 | 0.008794 | 1 | 0.024469 | 0.005868 | SAT1 |
| Perifollicular inflammatory infiltrate | 1 | 2 | 12.5 | 136.010395 | 0.008794 | 1 | 0.023948 | 0.005868 | SAT1 |
| Right ventricular hypertrophy | 1 | 2 | 12.5 | 136.010395 | 0.008794 | 1 | 0.02345 | 0.005868 | SGCG |
| Alopecia, beginning in the occiput | 1 | 2 | 12.5 | 136.010395 | 0.008794 | 1 | 0.022971 | 0.005868 | SAT1 |
| Split hand | 1 | 2 | 12.5 | 136.010395 | 0.008794 | 1 | 0.022512 | 0.005868 | DLX5 |
| Absent patella | 1 | 2 | 12.5 | 136.010395 | 0.008794 | 1 | 0.02207 | 0.005868 | MIPOL1 |
| Epidermal hyperplasia | 1 | 2 | 12.5 | 136.010395 | 0.008794 | 1 | 0.021646 | 0.005868 | SAT1 |
| Facial erythema | 1 | 2 | 12.5 | 136.010395 | 0.008794 | 1 | 0.021237 | 0.005868 | SAT1 |
| Allelic disorder to IFAP syndrome ( | 1 | 2 | 12.5 | 136.010395 | 0.008794 | 1 | 0.020844 | 0.005868 | SAT1 |
| Follicular hyperkeratotic papules | 1 | 2 | 12.5 | 136.010395 | 0.008794 | 1 | 0.020465 | 0.005868 | SAT1 |
| Absent radius | 1 | 2 | 12.5 | 136.010395 | 0.008794 | 1 | 0.0201 | 0.005868 | MIPOL1 |
| Folliculitis | 1 | 2 | 12.5 | 136.010395 | 0.008794 | 1 | 0.019747 | 0.005868 | SAT1 |
| Folliculitis of the scalp, trunk, and extensor surfaces of the extremities | 1 | 2 | 12.5 | 136.010395 | 0.008794 | 1 | 0.019407 | 0.005868 | SAT1 |
| Cystathioninuria | 1 | 3 | 12.5 | 104.0079491 | 0.013165 | 1 | 0.028561 | 0.007721 | CTH |
| Scarring alopecia | 1 | 3 | 12.5 | 104.0079491 | 0.013165 | 1 | 0.028085 | 0.007721 | SAT1 |
| Short, broad feet | 1 | 3 | 12.5 | 104.0079491 | 0.013165 | 1 | 0.027625 | 0.007721 | MIPOL1 |
| Keratitis | 1 | 3 | 12.5 | 104.0079491 | 0.013165 | 1 | 0.027179 | 0.007721 | SAT1 |
| Corneal dystrophy | 1 | 3 | 12.5 | 104.0079491 | 0.013165 | 1 | 0.026748 | 0.007721 | SAT1 |
| Calf muscle pseudohypertrophy | 1 | 3 | 12.5 | 104.0079491 | 0.013165 | 1 | 0.02633 | 0.007721 | SGCG |
| Split foot | 1 | 3 | 12.5 | 104.0079491 | 0.013165 | 1 | 0.025925 | 0.007721 | DLX5 |
| Carrier females may have mild features | 1 | 3 | 12.5 | 104.0079491 | 0.013165 | 1 | 0.025532 | 0.007721 | SAT1 |
| Muscle fiber necrosis | 1 | 4 | 12.5 | 84.1969112 | 0.017519 | 1 | 0.03347 | 0.009972 | SGCG |
| Rapid progression | 1 | 4 | 12.5 | 84.1969112 | 0.017519 | 1 | 0.032978 | 0.009972 | SGCG |
| Gowers sign | 1 | 6 | 12.5 | 60.97017707 | 0.026178 | 1 | 0.048562 | 0.014271 | SGCG |
| Blepharitis | 1 | 6 | 12.5 | 60.97017707 | 0.026178 | 1 | 0.047868 | 0.014271 | SAT1 |
| Ectrodactyly | 1 | 6 | 12.5 | 60.97017707 | 0.026178 | 1 | 0.047194 | 0.014271 | DLX5 |
