# Supplementary material for: Evaluation of autoantibody signatures in meningioma patients using human proteome arrays
Source: Oncotarget. 2017 Apr 10;8(35):58443–56. doi: 10.18632/oncotarget.16997 (PMC5601665; doi:10.18632/oncotarget.16997)
Supplement: Supplementary file 14 [file oncotarget-08-58443-s014.docx]

**Supplementary Table 7: Gene set enrichment analysis of MG vs HC**

**Supplementary Table 7.1: Gene details**

| Name of data set: MG vs HC |  |  |  |  |  |  |
| --- | --- | --- | --- | --- | --- | --- |
| No. of mapped items: 165 |  |  |  |  |  |  |
| No. of unmapped items: 36 |  |  |  |  |  |  |
| No. of redundant items: 2 |  |  |  |  |  |  |
| Is data set quantitative :NO |  |  |  |  |  |  |
|  |  |  |  |  |  |  |
| Mapped items: |  |  |  |  |  |  |
| Search Term | Enrtez GeneID | Gene Symbol | Description | Alternate names | Chromosome | Map location |
| CRYM | [1428](http://www.ncbi.nlm.nih.gov/gene/1428) | CRYM | crystallin, mu | DFNA40\|THBP | 16 | 16p12.2 |
| KCNMB3 | [27094](http://www.ncbi.nlm.nih.gov/gene/27094) | KCNMB3 | potassium large conductance calcium-activated channel, subfamily M beta member 3 | BKBETA3\|HBETA3\|KCNMB2\|KCNMBL\|SLOBETA3 | 3 | 3q26.3-q27 |
| EFCAB2 | [84288](http://www.ncbi.nlm.nih.gov/gene/84288) | EFCAB2 | EF-hand calcium binding domain 2 | - | 1 | 1q44 |
| HOXA5 | [3202](http://www.ncbi.nlm.nih.gov/gene/3202) | HOXA5 | homeobox A5 | HOX1\|HOX1.3\|HOX1C | 7 | 7p15.2 |
| ADRB2 | [154](http://www.ncbi.nlm.nih.gov/gene/154) | ADRB2 | adrenoceptor beta 2, surface | ADRB2R\|ADRBR\|B2AR\|BAR\|BETA2AR | 5 | 5q31-q32 |
| STAT6 | [6778](http://www.ncbi.nlm.nih.gov/gene/6778) | STAT6 | signal transducer and activator of transcription 6, interleukin-4 induced | D12S1644\|IL-4-STAT\|STAT6B\|STAT6C | 12 | 12q13 |
| CARHSP1 | [23589](http://www.ncbi.nlm.nih.gov/gene/23589) | CARHSP1 | calcium regulated heat stable protein 1, 24kDa | CRHSP-24\|CSDC1 | 16 | 16p13.2 |
| PSMD6 | [9861](http://www.ncbi.nlm.nih.gov/gene/9861) | PSMD6 | proteasome (prosome, macropain) 26S subunit, non-ATPase, 6 | Rpn7\|S10\|SGA-113M\|p42A\|p44S10 | 3 | 3p14.1 |
| GSG1 | [83445](http://www.ncbi.nlm.nih.gov/gene/83445) | GSG1 | germ cell associated 1 | - | 12 | 12p13.1 |
| DRG1 | [4733](http://www.ncbi.nlm.nih.gov/gene/4733) | DRG1 | developmentally regulated GTP binding protein 1 | NEDD3 | 22 | 22q12.2 |
| CCNB1 | [891](http://www.ncbi.nlm.nih.gov/gene/891) | CCNB1 | cyclin B1 | CCNB | 5 | 5q12 |
| COX4I1 | [1327](http://www.ncbi.nlm.nih.gov/gene/1327) | COX4I1 | cytochrome c oxidase subunit IV isoform 1 | COX4\|COX4-1\|COXIV | 16 | 16q24.1 |
| DOHH | [83475](http://www.ncbi.nlm.nih.gov/gene/83475) | DOHH | deoxyhypusine hydroxylase/monooxygenase | HLRC1\|hDOHH | 19 | 19p13.3 |
| COQ6 | [51004](http://www.ncbi.nlm.nih.gov/gene/51004) | COQ6 | coenzyme Q6 monooxygenase | CGI10\|COQ10D6 | 14 | 14q24.3 |
| ARPC3 | [10094](http://www.ncbi.nlm.nih.gov/gene/10094) | ARPC3 | actin related protein 2/3 complex, subunit 3, 21kDa | ARC21\|p21-Arc | 12 | 12q24.11 |
| PROSC | [11212](http://www.ncbi.nlm.nih.gov/gene/11212) | PROSC | proline synthetase co-transcribed homolog (bacterial) | - | 8 | 8p11.2 |
| OIP5 | [11339](http://www.ncbi.nlm.nih.gov/gene/11339) | OIP5 | Opa interacting protein 5 | 5730547N13Rik\|CT86\|LINT-25\|MIS18B\|MIS18beta\|hMIS18beta | 15 | 15q15.1 |
| KRR1 | [11103](http://www.ncbi.nlm.nih.gov/gene/11103) | KRR1 | KRR1, small subunit (SSU) processome component, homolog (yeast) | HRB2\|RIP-1 | 12 | 12q21.2 |
| FST | [10468](http://www.ncbi.nlm.nih.gov/gene/10468) | FST | follistatin | FS | 5 | 5q11.2 |
| RAB3B | [5865](http://www.ncbi.nlm.nih.gov/gene/5865) | RAB3B | RAB3B, member RAS oncogene family | - | 1 | 1p32-p31 |
| CCDC28A | [25901](http://www.ncbi.nlm.nih.gov/gene/25901) | CCDC28A | coiled-coil domain containing 28A | C6orf80\|CCRL1AP | 6 | 6q23.1-q24.1 |
| MRPL13 | [28998](http://www.ncbi.nlm.nih.gov/gene/28998) | MRPL13 | mitochondrial ribosomal protein L13 | L13\|L13A\|L13mt\|RPL13\|RPML13 | 8 | 8q22.1-q22.3 |
| CALCOCO2 | [10241](http://www.ncbi.nlm.nih.gov/gene/10241) | CALCOCO2 | calcium binding and coiled-coil domain 2 | NDP52 | 17 | 17q21.32 |
| ZHX3 | [23051](http://www.ncbi.nlm.nih.gov/gene/23051) | ZHX3 | zinc fingers and homeoboxes 3 | TIX1 | 20 | 20q12 |
| CORO1A | [11151](http://www.ncbi.nlm.nih.gov/gene/11151) | CORO1A | coronin, actin binding protein, 1A | CLABP\|CLIPINA\|HCORO1\|IMD8\|TACO\|p57 | 16 | 16p11.2 |
| GYPE | [2996](http://www.ncbi.nlm.nih.gov/gene/2996) | GYPE | glycophorin E (MNS blood group) | GPE\|MNS\|MiIX | 4 | 4q31.1 |
| KLK1 | [3816](http://www.ncbi.nlm.nih.gov/gene/3816) | KLK1 | kallikrein 1 | KLKR\|Klk6\|hK1 | 19 | 19q13.3 |
| DNAJB5 | [25822](http://www.ncbi.nlm.nih.gov/gene/25822) | DNAJB5 | DnaJ (Hsp40) homolog, subfamily B, member 5 | Hsc40 | 9 | 9p13.3 |
| SLC39A9 | [55334](http://www.ncbi.nlm.nih.gov/gene/55334) | SLC39A9 | solute carrier family 39, member 9 | ZIP-9\|ZIP9 | 14 | 14q24.1 |
| MAPK3 | [5595](http://www.ncbi.nlm.nih.gov/gene/5595) | MAPK3 | mitogen-activated protein kinase 3 | ERK-1\|ERK1\|ERT2\|HS44KDAP\|HUMKER1A\|P44ERK1\|P44MAPK\|PRKM3\|p44-ERK1\|p44-MAPK | 16 | 16p11.2 |
| MRPS7 | [51081](http://www.ncbi.nlm.nih.gov/gene/51081) | MRPS7 | mitochondrial ribosomal protein S7 | MRP-S\|MRP-S7\|RP-S7\|RPMS7\|S7mt\|bMRP27a | 17 | 17q25 |
| DTD1 | [92675](http://www.ncbi.nlm.nih.gov/gene/92675) | DTD1 | D-tyrosyl-tRNA deacylase 1 | C20orf88\|DUE-B\|DUEB\|HARS2\|bA379J5.3\|bA555E18.1\|pqn-68 | 20 | 20p11.23 |
| HPCAL1 | [3241](http://www.ncbi.nlm.nih.gov/gene/3241) | HPCAL1 | hippocalcin-like 1 | BDR1\|HLP2\|VILIP-3 | 2 | 2p25.1 |
| GPSM3 | [63940](http://www.ncbi.nlm.nih.gov/gene/63940) | GPSM3 | G-protein signaling modulator 3 | AGS4\|C6orf9\|G18\|G18.1a\|G18.1b\|G18.2\|NG1 | 6 | 6p21.3 |
| DOK1 | [1796](http://www.ncbi.nlm.nih.gov/gene/1796) | DOK1 | docking protein 1, 62kDa (downstream of tyrosine kinase 1) | P62DOK | 2 | 2p13 |
| C2orf44 | [80304](http://www.ncbi.nlm.nih.gov/gene/80304) | C2orf44 | chromosome 2 open reading frame 44 | - | 2 | 2p23.3 |
| ZFYVE19 | [84936](http://www.ncbi.nlm.nih.gov/gene/84936) | ZFYVE19 | zinc finger, FYVE domain containing 19 | MPFYVE | 15 | 15q15.1 |
| EIF4EBP3 | [8637](http://www.ncbi.nlm.nih.gov/gene/8637) | EIF4EBP3 | eukaryotic translation initiation factor 4E binding protein 3 | 4E-BP3\|4EBP3 | 5 | 5q31.3 |
| PRPSAP2 | [5636](http://www.ncbi.nlm.nih.gov/gene/5636) | PRPSAP2 | phosphoribosyl pyrophosphate synthetase-associated protein 2 | PAP41 | 17 | 17p11.2-p12 |
| CKS2 | [1164](http://www.ncbi.nlm.nih.gov/gene/1164) | CKS2 | CDC28 protein kinase regulatory subunit 2 | CKSHS2 | 9 | 9q22 |
| METTL8 | [79828](http://www.ncbi.nlm.nih.gov/gene/79828) | METTL8 | methyltransferase like 8 | TIP | 2 | 2q31.1 |
| OR10G3 | [26533](http://www.ncbi.nlm.nih.gov/gene/26533) | OR10G3 | olfactory receptor, family 10, subfamily G, member 3 | OR14-40 | 14 | 14q11.2 |
| TSPAN17 | [26262](http://www.ncbi.nlm.nih.gov/gene/26262) | TSPAN17 | tetraspanin 17 | FBX23\|FBXO23\|TM4SF17 | 5 | 5q35.3 |
| TIMP1 | [7076](http://www.ncbi.nlm.nih.gov/gene/7076) | TIMP1 | TIMP metallopeptidase inhibitor 1 | CLGI\|EPA\|EPO\|HCI\|TIMP | X | Xp11.3-p11.23 |
| HLA-DRB5 | [3127](http://www.ncbi.nlm.nih.gov/gene/3127) | HLA-DRB5 | major histocompatibility complex, class II, DR beta 5 | HLA-DRB | 6 | 6p21.3 |
| RHOA | [387](http://www.ncbi.nlm.nih.gov/gene/387) | RHOA | ras homolog family member A | ARH12\|ARHA\|RHO12\|RHOH12 | 3 | 3p21.3 |
| PPP2R4 | [5524](http://www.ncbi.nlm.nih.gov/gene/5524) | PPP2R4 | protein phosphatase 2A activator, regulatory subunit 4 | PP2A\|PR53\|PTPA | 9 | 9q34 |
| EPS8L1 | [54869](http://www.ncbi.nlm.nih.gov/gene/54869) | EPS8L1 | EPS8-like 1 | DRC3\|EPS8R1 | 19 | 19q13.42 |
| STAU2 | [27067](http://www.ncbi.nlm.nih.gov/gene/27067) | STAU2 | staufen double-stranded RNA binding protein 2 | 39K2\|39K3 | 8 | 8q21.11 |
| GULP1 | [51454](http://www.ncbi.nlm.nih.gov/gene/51454) | GULP1 | GULP, engulfment adaptor PTB domain containing 1 | CED-6\|CED6\|GULP | 2 | 2q32.3-q33 |
| GADD45A | [1647](http://www.ncbi.nlm.nih.gov/gene/1647) | GADD45A | growth arrest and DNA-damage-inducible, alpha | DDIT1\|GADD45 | 1 | 1p31.2 |
| RNF11 | [26994](http://www.ncbi.nlm.nih.gov/gene/26994) | RNF11 | ring finger protein 11 | SID1669 | 1 | 1p32 |
| DLX5 | [1749](http://www.ncbi.nlm.nih.gov/gene/1749) | DLX5 | distal-less homeobox 5 | SHFM1D | 7 | 7q22 |
| BHMT2 | [23743](http://www.ncbi.nlm.nih.gov/gene/23743) | BHMT2 | betaine--homocysteine S-methyltransferase 2 | - | 5 | 5q13 |
| TIPIN | [54962](http://www.ncbi.nlm.nih.gov/gene/54962) | TIPIN | TIMELESS interacting protein | - | 15 | 15q22.31 |
| LCN1 | [3933](http://www.ncbi.nlm.nih.gov/gene/3933) | LCN1 | lipocalin 1 | PMFA\|TLC\|TP\|VEGP | 9 | 9q34 |
| IFIT3 | [3437](http://www.ncbi.nlm.nih.gov/gene/3437) | IFIT3 | interferon-induced protein with tetratricopeptide repeats 3 | CIG-49\|GARG-49\|IFI60\|IFIT4\|IRG2\|ISG60\|P60\|RIG-G | 10 | 10q24 |
| CCT4 | [10575](http://www.ncbi.nlm.nih.gov/gene/10575) | CCT4 | chaperonin containing TCP1, subunit 4 (delta) | CCT-DELTA\|Cctd\|SRB | 2 | 2p15 |
| DDI2 | [84301](http://www.ncbi.nlm.nih.gov/gene/84301) | DDI2 | DNA-damage inducible 1 homolog 2 (S. cerevisiae) | - | 1 | 1p36.21 |
| CDC34 | [997](http://www.ncbi.nlm.nih.gov/gene/997) | CDC34 | cell division cycle 34 | E2-CDC34\|UBC3\|UBCH3\|UBE2R1 | 19 | 19p13.3 |
| SULT1E1 | [6783](http://www.ncbi.nlm.nih.gov/gene/6783) | SULT1E1 | sulfotransferase family 1E, estrogen-preferring, member 1 | EST\|EST-1\|ST1E1\|STE | 4 | 4q13.1 |
| LTC4S | [4056](http://www.ncbi.nlm.nih.gov/gene/4056) | LTC4S | leukotriene C4 synthase | - | 5 | 5q35 |
| NMRAL1 | [57407](http://www.ncbi.nlm.nih.gov/gene/57407) | NMRAL1 | NmrA-like family domain containing 1 | HSCARG\|SDR48A1 | 16 | 16p13.3 |
| HSPA2 | [3306](http://www.ncbi.nlm.nih.gov/gene/3306) | HSPA2 | heat shock 70kDa protein 2 | HSP70-2\|HSP70-3 | 14 | 14q24.1 |
| MIPOL1 | [145282](http://www.ncbi.nlm.nih.gov/gene/145282) | MIPOL1 | mirror-image polydactyly 1 | - | 14 | 14q13.3 |
| GALT | [2592](http://www.ncbi.nlm.nih.gov/gene/2592) | GALT | galactose-1-phosphate uridylyltransferase | - | 9 | 9p13 |
| TIRAP | [114609](http://www.ncbi.nlm.nih.gov/gene/114609) | TIRAP | toll-interleukin 1 receptor (TIR) domain containing adaptor protein | BACTS1\|Mal\|MyD88-2\|wyatt | 11 | 11q24.2 |
| ABLIM1 | [3983](http://www.ncbi.nlm.nih.gov/gene/3983) | ABLIM1 | actin binding LIM protein 1 | ABLIM\|LIMAB1\|LIMATIN\|abLIM-1 | 10 | 10q25 |
| MTL5 | [9633](http://www.ncbi.nlm.nih.gov/gene/9633) | MTL5 | metallothionein-like 5, testis-specific (tesmin) | CXCDC2\|MTLT\|TESMIN | 11 | 11q13.2-q13.3 |
| PRKRA | [8575](http://www.ncbi.nlm.nih.gov/gene/8575) | PRKRA | protein kinase, interferon-inducible double stranded RNA dependent activator | DYT16\|PACT\|RAX | 2 | 2q31.2 |
| PAIP1 | [10605](http://www.ncbi.nlm.nih.gov/gene/10605) | PAIP1 | poly(A) binding protein interacting protein 1 | - | 5 | 5p12 |
| TRIM68 | [55128](http://www.ncbi.nlm.nih.gov/gene/55128) | TRIM68 | tripartite motif containing 68 | GC109\|RNF137\|SS-56\|SS56 | 11 | 11p15.4 |
| CAMK4 | [814](http://www.ncbi.nlm.nih.gov/gene/814) | CAMK4 | calcium/calmodulin-dependent protein kinase IV | CaMK IV\|CaMK-GR\|IV\|caMK | 5 | 5q21.3 |
| UGDH | [7358](http://www.ncbi.nlm.nih.gov/gene/7358) | UGDH | UDP-glucose 6-dehydrogenase | GDH\|UDP-GlcDH\|UDPGDH\|UGD | 4 | 4p15.1 |
| SPOP | [8405](http://www.ncbi.nlm.nih.gov/gene/8405) | SPOP | speckle-type POZ protein | BTBD32\|TEF2 | 17 | 17q21.33 |
| CAPRIN2 | [65981](http://www.ncbi.nlm.nih.gov/gene/65981) | CAPRIN2 | caprin family member 2 | C1QDC1\|EEG-1\|EEG1\|RNG140 | 12 | 12p11 |
| SPAG16 | [79582](http://www.ncbi.nlm.nih.gov/gene/79582) | SPAG16 | sperm associated antigen 16 | PF20\|WDR29 | 2 | 2q34 |
| RAB24 | [53917](http://www.ncbi.nlm.nih.gov/gene/53917) | RAB24 | RAB24, member RAS oncogene family | - | 5 | 5q35.3 |
| IMPDH2 | [3615](http://www.ncbi.nlm.nih.gov/gene/3615) | IMPDH2 | IMP (inosine 5'-monophosphate) dehydrogenase 2 | IMPD2\|IMPDH-II | 3 | 3p21.2 |
| ANP32E | [81611](http://www.ncbi.nlm.nih.gov/gene/81611) | ANP32E | acidic (leucine-rich) nuclear phosphoprotein 32 family, member E | LANP-L\|LANPL | 1 | 1q21.2 |
| UBE2V2 | [7336](http://www.ncbi.nlm.nih.gov/gene/7336) | UBE2V2 | ubiquitin-conjugating enzyme E2 variant 2 | DDVIT1\|DDVit-1\|EDAF-1\|EDPF-1\|EDPF1\|MMS2\|UEV-2\|UEV2 | 8 | 8q11.21 |
| FAIM | [55179](http://www.ncbi.nlm.nih.gov/gene/55179) | FAIM | Fas apoptotic inhibitory molecule | FAIM1 | 3 | 3q22.3 |
| HBG2 | [3048](http://www.ncbi.nlm.nih.gov/gene/3048) | HBG2 | hemoglobin, gamma G | HBG-T1\|TNCY | 11 | 11p15.5 |
| ATG3 | [64422](http://www.ncbi.nlm.nih.gov/gene/64422) | ATG3 | autophagy related 3 | APG3\|APG3-LIKE\|APG3L\|PC3-96 | 3 | 3q13.2 |
| CLGN | [1047](http://www.ncbi.nlm.nih.gov/gene/1047) | CLGN | calmegin | - | 4 | 4q28.3-q31.1 |
| SFN | [2810](http://www.ncbi.nlm.nih.gov/gene/2810) | SFN | stratifin | YWHAS | 1 | 1p36.11 |
| KLF11 | [8462](http://www.ncbi.nlm.nih.gov/gene/8462) | KLF11 | Kruppel-like factor 11 | FKLF\|FKLF1\|MODY7\|TIEG2\|Tieg3 | 2 | 2p25 |
| P2RX7 | [5027](http://www.ncbi.nlm.nih.gov/gene/5027) | P2RX7 | purinergic receptor P2X, ligand-gated ion channel, 7 | P2X7 | 12 | 12q24 |
| TMEM185B | [79134](http://www.ncbi.nlm.nih.gov/gene/79134) | TMEM185B | transmembrane protein 185B | FAM11B | 2 | 2q14.2 |
| TEAD3 | [7005](http://www.ncbi.nlm.nih.gov/gene/7005) | TEAD3 | TEA domain family member 3 | DTEF-1\|ETFR-1\|TEAD-3\|TEAD5\|TEF-5\|TEF5 | 6 | 6p21.2 |
| CALN1 | [83698](http://www.ncbi.nlm.nih.gov/gene/83698) | CALN1 | calneuron 1 | CABP8 | 7 | 7q11 |
| ASF1A | [25842](http://www.ncbi.nlm.nih.gov/gene/25842) | ASF1A | anti-silencing function 1A histone chaperone | CGI-98\|CIA\|HSPC146 | 6 | 6q22.31 |
| RAB11A | [8766](http://www.ncbi.nlm.nih.gov/gene/8766) | RAB11A | RAB11A, member RAS oncogene family | YL8 | 15 | 15q22.31 |
| SNX1 | [6642](http://www.ncbi.nlm.nih.gov/gene/6642) | SNX1 | sorting nexin 1 | HsT17379\|VPS5 | 15 | 15q22.31 |
| FAM63A | [55793](http://www.ncbi.nlm.nih.gov/gene/55793) | FAM63A | family with sequence similarity 63, member A | - | 1 | 1q21.3 |
| RBBP7 | [5931](http://www.ncbi.nlm.nih.gov/gene/5931) | RBBP7 | retinoblastoma binding protein 7 | RbAp46 | X | Xp22.2 |
| PCGF3 | [10336](http://www.ncbi.nlm.nih.gov/gene/10336) | PCGF3 | polycomb group ring finger 3 | DONG1\|RNF3\|RNF3A | 4 | 4p16.3 |
| CKAP2 | [26586](http://www.ncbi.nlm.nih.gov/gene/26586) | CKAP2 | cytoskeleton associated protein 2 | LB1\|TMAP\|se20-10 | 13 | 13q14 |
| PDLIM3 | [27295](http://www.ncbi.nlm.nih.gov/gene/27295) | PDLIM3 | PDZ and LIM domain 3 | ALP | 4 | 4q35 |
| LRFN1 | [57622](http://www.ncbi.nlm.nih.gov/gene/57622) | LRFN1 | leucine rich repeat and fibronectin type III domain containing 1 | SALM2 | 19 | 19q13.2 |
| MARCKSL1 | [65108](http://www.ncbi.nlm.nih.gov/gene/65108) | MARCKSL1 | MARCKS-like 1 | F52\|MACMARCKS\|MLP\|MLP1\|MRP | 1 | 1p35.1 |
| C14orf119 | [55017](http://www.ncbi.nlm.nih.gov/gene/55017) | C14orf119 | chromosome 14 open reading frame 119 | - | 14 | 14q11.2 |
| CDO1 | [1036](http://www.ncbi.nlm.nih.gov/gene/1036) | CDO1 | cysteine dioxygenase type 1 | - | 5 | 5q23.2 |
| CCR10 | [2826](http://www.ncbi.nlm.nih.gov/gene/2826) | CCR10 | chemokine (C-C motif) receptor 10 | GPR2 | 17 | 17q21.1-q21.3 |
| AIF1 | [199](http://www.ncbi.nlm.nih.gov/gene/199) | AIF1 | allograft inflammatory factor 1 | AIF-1\|IBA1\|IRT-1\|IRT1 | 6 | 6p21.3 |
| SEPT1 | [1731](http://www.ncbi.nlm.nih.gov/gene/1731) | SEPT1 | septin 1 | DIFF6\|LARP\|PNUTL3\|SEP1 | 16 | 16p11.1 |
| CDH26 | [60437](http://www.ncbi.nlm.nih.gov/gene/60437) | CDH26 | cadherin 26 | VR20 | 20 | 20q13.33 |
| RPS6KA2 | [6196](http://www.ncbi.nlm.nih.gov/gene/6196) | RPS6KA2 | ribosomal protein S6 kinase, 90kDa, polypeptide 2 | HU-2\|MAPKAPK1C\|RSK\|RSK3\|S6K-alpha\|S6K-alpha2\|p90-RSK3\|pp90RSK3 | 6 | 6q27 |
| ARL2BP | [23568](http://www.ncbi.nlm.nih.gov/gene/23568) | ARL2BP | ADP-ribosylation factor-like 2 binding protein | BART\|BART1\|RP66 | 16 | 16q13 |
| NME1 | [4830](http://www.ncbi.nlm.nih.gov/gene/4830) | NME1 | NME/NM23 nucleoside diphosphate kinase 1 | AWD\|GAAD\|NB\|NBS\|NDKA\|NDPK-A\|NDPKA\|NM23\|NM23-H1 | 17 | 17q21.3 |
| C14orf80 | [283643](http://www.ncbi.nlm.nih.gov/gene/283643) | C14orf80 | chromosome 14 open reading frame 80 | - | 14 | 14q32.33 |
| RAP1GDS1 | [5910](http://www.ncbi.nlm.nih.gov/gene/5910) | RAP1GDS1 | RAP1, GTP-GDP dissociation stimulator 1 | GDS1\|SmgGDS | 4 | 4q23-q25 |
| HN1 | [51155](http://www.ncbi.nlm.nih.gov/gene/51155) | HN1 | hematological and neurological expressed 1 | ARM2\|HN1A | 17 | 17q25.1 |
| GNB2L1 | [10399](http://www.ncbi.nlm.nih.gov/gene/10399) | GNB2L1 | guanine nucleotide binding protein (G protein), beta polypeptide 2-like 1 | Gnb2-rs1\|H12.3\|HLC-7\|PIG21\|RACK1 | 5 | 5q35.3 |
| FN3K | [64122](http://www.ncbi.nlm.nih.gov/gene/64122) | FN3K | fructosamine 3 kinase | - | 17 | 17q25.3 |
| VSNL1 | [7447](http://www.ncbi.nlm.nih.gov/gene/7447) | VSNL1 | visinin-like 1 | HLP3\|HPCAL3\|HUVISL1\|VILIP\|VILIP-1 | 2 | 2p24.3 |
| ABCF3 | [55324](http://www.ncbi.nlm.nih.gov/gene/55324) | ABCF3 | ATP-binding cassette, sub-family F (GCN20), member 3 | EST201864 | 3 | 3q27.1 |
| HLA-DRB3 | [3125](http://www.ncbi.nlm.nih.gov/gene/3125) | HLA-DRB3 | major histocompatibility complex, class II, DR beta 3 | HLA-DR3B | 6 | 6p21.3 |
| ATP6V1E2 | [90423](http://www.ncbi.nlm.nih.gov/gene/90423) | ATP6V1E2 | ATPase, H+ transporting, lysosomal 31kDa, V1 subunit E2 | ATP6E1\|ATP6EL2\|ATP6V1EL2\|VMA4 | 2 | 2p21 |
| NAP1L1 | [4673](http://www.ncbi.nlm.nih.gov/gene/4673) | NAP1L1 | nucleosome assembly protein 1-like 1 | NAP1\|NAP1L\|NRP | 12 | 12q21.2 |
| MPHOSPH9 | [10198](http://www.ncbi.nlm.nih.gov/gene/10198) | MPHOSPH9 | M-phase phosphoprotein 9 | MPP-9\|MPP9 | 12 | 12q24.31 |
| FABP5 | [2171](http://www.ncbi.nlm.nih.gov/gene/2171) | FABP5 | fatty acid binding protein 5 (psoriasis-associated) | E-FABP\|EFABP\|KFABP\|PA-FABP\|PAFABP | 8 | 8q21.13 |
| ANXA11 | [311](http://www.ncbi.nlm.nih.gov/gene/311) | ANXA11 | annexin A11 | ANX11\|CAP50 | 10 | 10q23 |
| DUPD1 | [338599](http://www.ncbi.nlm.nih.gov/gene/338599) | DUPD1 | dual specificity phosphatase and pro isomerase domain containing 1 | DUSP27\|FMDSP | 10 | 10q22.2 |
| BLOC1S2 | [282991](http://www.ncbi.nlm.nih.gov/gene/282991) | BLOC1S2 | biogenesis of lysosomal organelles complex-1, subunit 2 | BLOS2\|CEAP\|CEAP11 | 10 | 10q24.31 |
| TARDBP | [23435](http://www.ncbi.nlm.nih.gov/gene/23435) | TARDBP | TAR DNA binding protein | ALS10\|TDP-43 | 1 | 1p36.22 |
| TTC1 | [7265](http://www.ncbi.nlm.nih.gov/gene/7265) | TTC1 | tetratricopeptide repeat domain 1 | TPR1 | 5 | 5q33.3 |
| PPP3R1 | [5534](http://www.ncbi.nlm.nih.gov/gene/5534) | PPP3R1 | protein phosphatase 3, regulatory subunit B, alpha | CALNB1\|CNB\|CNB1 | 2 | 2p15 |
| S100A7A | [338324](http://www.ncbi.nlm.nih.gov/gene/338324) | S100A7A | S100 calcium binding protein A7A | NICE-2\|S100A15\|S100A7L1\|S100A7f | 1 | 1q21.3 |
| ZMYM3 | [9203](http://www.ncbi.nlm.nih.gov/gene/9203) | ZMYM3 | zinc finger, MYM-type 3 | DXS6673E\|MYM\|XFIM\|ZNF198L2\|ZNF261 | X | Xq13.1 |
| GH1 | [2688](http://www.ncbi.nlm.nih.gov/gene/2688) | GH1 | growth hormone 1 | GH\|GH-N\|GHN\|IGHD1B\|hGH-N | 17 | 17q24.2 |
| ATP6V1C2 | [245973](http://www.ncbi.nlm.nih.gov/gene/245973) | ATP6V1C2 | ATPase, H+ transporting, lysosomal 42kDa, V1 subunit C2 | ATP6C2\|VMA5 | 2 | - |
| SNX9 | [51429](http://www.ncbi.nlm.nih.gov/gene/51429) | SNX9 | sorting nexin 9 | SDP1\|SH3PX1\|SH3PXD3A\|WISP | 6 | 6q25.1-q26 |
| SIGLEC5 | [8778](http://www.ncbi.nlm.nih.gov/gene/8778) | SIGLEC5 | sialic acid binding Ig-like lectin 5 | CD170\|CD33L2\|OB-BP2\|OBBP2\|SIGLEC-5 | 19 | 19q13.3 |
| LST1 | [7940](http://www.ncbi.nlm.nih.gov/gene/7940) | LST1 | leukocyte specific transcript 1 | B144\|D6S49E\|LST-1 | 6 | 6p21.3 |
| COG3 | [83548](http://www.ncbi.nlm.nih.gov/gene/83548) | COG3 | component of oligomeric golgi complex 3 | SEC34 | 13 | 13q14.13 |
| LEMD1 | [93273](http://www.ncbi.nlm.nih.gov/gene/93273) | LEMD1 | LEM domain containing 1 | CT50\|LEMP-1 | 1 | 1q32.1 |
| Lhx1 | [3975](http://www.ncbi.nlm.nih.gov/gene/3975) | LHX1 | LIM homeobox 1 | LIM-1\|LIM1 | 17 | 17q12 |
| SRI | [6717](http://www.ncbi.nlm.nih.gov/gene/6717) | SRI | sorcin | CP-22\|CP22\|SCN\|V19 | 7 | 7q21.1 |
| YWHAB | [7529](http://www.ncbi.nlm.nih.gov/gene/7529) | YWHAB | tyrosine 3-monooxygenase/tryptophan 5-monooxygenase activation protein, beta | GW128\|HEL-S-1\|HS1\|KCIP-1\|YWHAA | 20 | 20q13.1 |
| ANXA6 | [309](http://www.ncbi.nlm.nih.gov/gene/309) | ANXA6 | annexin A6 | ANX6\|CBP68 | 5 | 5q33.1 |
| TPD52L2 | [7165](http://www.ncbi.nlm.nih.gov/gene/7165) | TPD52L2 | tumor protein D52-like 2 | D54 | 20 | 20q13.2-q13.3 |
| Nol3 | [8996](http://www.ncbi.nlm.nih.gov/gene/8996) | NOL3 | nucleolar protein 3 (apoptosis repressor with CARD domain) | ARC\|FCM\|MYP\|NOP\|NOP30 | 16 | 16q22.1 |
| SRPK2 | [6733](http://www.ncbi.nlm.nih.gov/gene/6733) | SRPK2 | SRSF protein kinase 2 | SFRSK2 | 7 | 7q22-q31.1 |
| CLP1 | [10978](http://www.ncbi.nlm.nih.gov/gene/10978) | CLP1 | cleavage and polyadenylation factor I subunit 1 | HEAB\|PCH10\|hClp1 | 11 | 11q12 |
| USP15 | [9958](http://www.ncbi.nlm.nih.gov/gene/9958) | USP15 | ubiquitin specific peptidase 15 | UNPH-2\|UNPH4 | 12 | 12q14 |
| CSAG1 | [158511](http://www.ncbi.nlm.nih.gov/gene/158511) | CSAG1 | chondrosarcoma associated gene 1 | CSAGE\|CT24.1 | X | Xq28 |
| PLEK | [5341](http://www.ncbi.nlm.nih.gov/gene/5341) | PLEK | pleckstrin | P47 | 2 | 2p13.3 |
| MLX | [6945](http://www.ncbi.nlm.nih.gov/gene/6945) | MLX | MLX, MAX dimerization protein | MAD7\|MXD7\|TCFL4\|bHLHd13 | 17 | 17q21.1 |
| FMN1 | [342184](http://www.ncbi.nlm.nih.gov/gene/342184) | FMN1 | formin 1 | FMN\|LD | 15 | 15q13.3 |
| PRKAR2B | [5577](http://www.ncbi.nlm.nih.gov/gene/5577) | PRKAR2B | protein kinase, cAMP-dependent, regulatory, type II, beta | PRKAR2\|RII-BETA | 7 | 7q22 |
| ACVR2B | [93](http://www.ncbi.nlm.nih.gov/gene/93) | ACVR2B | activin A receptor, type IIB | ACTRIIB\|ActR-IIB\|HTX4 | 3 | 3p22 |
| COASY | [80347](http://www.ncbi.nlm.nih.gov/gene/80347) | COASY | CoA synthase | DPCK\|NBIA6\|NBP\|PPAT\|UKR1\|pOV-2 | 17 | 17q12-q21 |
| TMIE | [259236](http://www.ncbi.nlm.nih.gov/gene/259236) | TMIE | transmembrane inner ear | DFNB6 | 3 | 3p21 |
| SERTAD3 | [29946](http://www.ncbi.nlm.nih.gov/gene/29946) | SERTAD3 | SERTA domain containing 3 | RBT1 | 19 | 19q13.2 |
| CAMK2N1 | [55450](http://www.ncbi.nlm.nih.gov/gene/55450) | CAMK2N1 | calcium/calmodulin-dependent protein kinase II inhibitor 1 | PRO1489 | 1 | 1p36.12 |
| ZNF655 | [79027](http://www.ncbi.nlm.nih.gov/gene/79027) | ZNF655 | zinc finger protein 655 | VIK\|VIK-1 | 7 | 7q22.1 |
| FAM104B | [90736](http://www.ncbi.nlm.nih.gov/gene/90736) | FAM104B | family with sequence similarity 104, member B | CXorf44 | X | Xp11.21 |
| SEPT2 | [4735](http://www.ncbi.nlm.nih.gov/gene/4735) | SEPT2 | septin 2 | DIFF6\|NEDD-5\|NEDD5\|Pnutl3\|hNedd5 | 2 | 2q37 |
| SNURF | [8926](http://www.ncbi.nlm.nih.gov/gene/8926) | SNURF | SNRPN upstream reading frame | - | 15 | 15q12 |
| PLEKHG2 | [64857](http://www.ncbi.nlm.nih.gov/gene/64857) | PLEKHG2 | pleckstrin homology domain containing, family G (with RhoGef domain) member 2 | ARHGEF42\|CLG | 19 | 19q13.2 |
| PAIP2 | [51247](http://www.ncbi.nlm.nih.gov/gene/51247) | PAIP2 | poly(A) binding protein interacting protein 2 | PAIP-2\|PAIP2A | 5 | 5q31.2 |
| RTN4 | [57142](http://www.ncbi.nlm.nih.gov/gene/57142) | RTN4 | reticulon 4 | ASY\|NI220/250\|NOGO\|NOGO-A\|NOGOC\|NSP\|NSP-CL\|Nbla00271\|Nbla10545\|Nogo-B\|Nogo-C\|RTN-X\|RTN4-A\|RTN4-B1\|RTN4-B2\|RTN4-C | 2 | 2p16.3 |
| GBE1 | [2632](http://www.ncbi.nlm.nih.gov/gene/2632) | GBE1 | glucan (1,4-alpha-), branching enzyme 1 | APBD\|GBE\|GSD4 | 3 | 3p12.3 |
| ARL5B | [221079](http://www.ncbi.nlm.nih.gov/gene/221079) | ARL5B | ADP-ribosylation factor-like 5B | ARL8 | 10 | 10p12.31 |
|  |  |  |  |  |  |  |
| Redundant entries |  |  |  |  |  |  |
|  | Entry "HSPA2" ignored. Both "HSPA2" and "HSPA2" mappes to "HSPA2" |  |  |  |  |  |
|  | Entry "ARL2BP" ignored. Both "ARL2BP" and "ARL2BP" mappes to "ARL2BP" |  |  |  |  |  |
|  |  |  |  |  |  |  |
| Unmapped Entries |  |  |  |  |  |  |
|  | IGHG4 |  |  |  |  |  |
|  | C20orf112 |  |  |  |  |  |
|  | RY1 |  |  |  |  |  |
|  | C17orf57 |  |  |  |  |  |
|  | HDAC7A |  |  |  |  |  |
|  | LOC389833 |  |  |  |  |  |
|  | SURB7 |  |  |  |  |  |
|  | HCG3 |  |  |  |  |  |
|  | CHP |  |  |  |  |  |
|  | NA |  |  |  |  |  |
|  | KARCA1 |  |  |  |  |  |
|  | WDR42A |  |  |  |  |  |
|  | LOC285382 |  |  |  |  |  |
|  | C8orf43 |  |  |  |  |  |
|  | IL1F7 |  |  |  |  |  |
|  | JUB |  |  |  |  |  |
|  | IGHG1 |  |  |  |  |  |
|  | M6PRBP1 |  |  |  |  |  |
|  | FAM105B |  |  |  |  |  |
|  | NY-REN-7 |  |  |  |  |  |
|  | EIF3S3 |  |  |  |  |  |
|  | C14orf122 |  |  |  |  |  |
|  | FAM119B |  |  |  |  |  |
|  | MGC2408 |  |  |  |  |  |
|  | PCID1 |  |  |  |  |  |
|  | NA |  |  |  |  |  |
|  | UBADC1 |  |  |  |  |  |
|  | RABL5 |  |  |  |  |  |
|  | IGHG1 |  |  |  |  |  |
|  | TXNL2 |  |  |  |  |  |
|  | LOC374395 |  |  |  |  |  |
|  | UBXD4 |  |  |  |  |  |
|  | LOC554174 |  |  |  |  |  |
|  | RP11-56A21.1 |  |  |  |  |  |
|  | IGHG1 |  |  |  |  |  |
|  | C20orf43 |  |  |  |  |  |

**Supplementary Table 7.2: Cellular component**

| Analysis:Cellular component |  |  |  |  |  |  |  |  |  |
| --- | --- | --- | --- | --- | --- | --- | --- | --- | --- |
| Name of data set: MG vs HC |  |  |  |  |  |  |  |  |  |
| Number of gene in data set: 165 |  |  |  |  |  |  |  |  |  |
| Number of gene mapped to Cellular component : 142 |  |  |  |  |  |  |  |  |  |
|  |  |  |  |  |  |  |  |  |  |
| Cellular component | No. of genes  in the data set | No. of genes in the background data set | Percentage of genes | Fold Enrichment | Uncorrected  p-value  (Hypergeometric test) | Corrected  p-value  (Bonferroni method) | Corrected  p-value  (BH method) | Storey and Tibshirani method  q-value | Genes mapped from  input data set |
| Cytoplasm | 85 | 5632 | 59.85915493 | 1.53891686 | 4.18E-07 | 3.34E-05 | 3.34E-05 | 1.49E-05 | CRYM,STAT6,CARHSP1,DRG1,CCNB1,COX4I1,ARPC3,PROSC,RAB3B,CALCOCO2,ZHX3,CORO1A,DNAJB5,MAPK3,DTD1,HPCAL1,DOK1,ZFYVE19,EIF4EBP3,PRPSAP2,RHOA,PPP2R4,STAU2,RNF11,DLX5,IFIT3,CCT4,DDI2,CDC34,SULT1E1,HSPA2,TIRAP,ABLIM1,MTL5,PRKRA,PAIP1,TRIM68,CAMK4,CAPRIN2,IMPDH2,UBE2V2,ATG3,CLGN,SFN,RAB11A,SNX1,FAM63A,CKAP2,PDLIM3,AIF1,SEPT1,RPS6KA2,NME1,RAP1GDS1,HN1,GNB2L1,FN3K,VSNL1,NAP1L1,MPHOSPH9,FABP5,ANXA11,PPP3R1,S100A7A,ZMYM3,LST1,COG3,SRI,YWHAB,ANXA6,TPD52L2,NOL3,SRPK2,USP15,PLEK,MLX,FMN1,PRKAR2B,ACVR2B,COASY,ZNF655,SEPT2,PLEKHG2,PAIP2,RTN4 |
| Phagocytic vesicle | 2 | 4 | 1.408450704 | 62.22130807 | 0.000572 | 0.045771 | 0.022885 | 0.010198 | CORO1A,ANXA11 |
| Exosomes | 34 | 2001 | 23.94366197 | 1.769521723 | 0.000873 | 0.069808 | 0.023269 | 0.010369 | CRYM,COX4I1,ARPC3,RAB3B,CORO1A,KLK1,HPCAL1,HLA-DRB5,RHOA,EPS8L1,RNF11,BHMT2,CCT4,HSPA2,CAMK4,UGDH,IMPDH2,UBE2V2,SFN,RAB11A,MARCKSL1,NME1,RAP1GDS1,GNB2L1,ANXA11,ATP6V1C2,SNX9,SRI,YWHAB,ANXA6,COASY,SEPT2,RTN4,GBE1 |

**Supplementary Table 7.3: Molecular function**

| Analysis:Molecular function |  |  |  |  |  |  |  |  |  |
| --- | --- | --- | --- | --- | --- | --- | --- | --- | --- |
| Name of data set: MG vs HC |  |  |  |  |  |  |  |  |  |
| Number of gene in data set: 165 |  |  |  |  |  |  |  |  |  |
| Number of gene mapped to Molecular function : 164 |  |  |  |  |  |  |  |  |  |
|  |  |  |  |  |  |  |  |  |  |
| Molecular function | No. of genes  in the data set | No. of genes in the background data set | Percentage of genes | Fold Enrichment | Uncorrected  p-value  (Hypergeometric test) | Corrected  p-value  (Bonferroni method) | Corrected  p-value  (BH method) | Storey and Tibshirani method  q-value | Genes mapped from  input data set |
| Calcium ion binding | 10 | 182 | 6.097560976 | 6.656013358 | 6.95E-06 | 0.000341 | 0.000341 | 0.000163 | EFCAB2,CARHSP1,HPCAL1,CALN1,VSNL1,ANXA11,S100A7A,SRI,ANXA6,PLEK |
| Receptor signaling complex scaffold activity | 10 | 320 | 6.097560976 | 3.796776491 | 0.000747 | 0.036581 | 0.01829 | 0.008749 | DOK1,GULP1,TIRAP,SFN,MARCKSL1,AIF1,GNB2L1,TTC1,SNX9,YWHAB |

**Supplementary Table 7.4: Biological process**

| Analysis:Biological process |  |  |  |  |  |  |  |  |  |
| --- | --- | --- | --- | --- | --- | --- | --- | --- | --- |
| Name of data set: MG vs HC |  |  |  |  |  |  |  |  |  |
| Number of gene in data set: 165 |  |  |  |  |  |  |  |  |  |
| Number of gene mapped to Biological process : 164 |  |  |  |  |  |  |  |  |  |
|  |  |  |  |  |  |  |  |  |  |
| Biological process | No. of genes  in the data set | No. of genes in the background data set | Percentage of genes | Fold Enrichment | Uncorrected  p-value  (Hypergeometric test) | Corrected  p-value  (Bonferroni method) | Corrected  p-value  (BH method) | Storey and Tibshirani method  q-value | Genes mapped from  input data set |
| Signal transduction | 56 | 3907 | 34.14634146 | 1.588176627 | 0.000178 | 0.003926 | 0.003926 | 0.001338 | EFCAB2,ADRB2,CARHSP1,CCNB1,OIP5,FST,RAB3B,MAPK3,HPCAL1,GPSM3,DOK1,CKS2,OR10G3,RHOA,PPP2R4,EPS8L1,GULP1,TIRAP,CAMK4,RAB24,SFN,CALN1,RAB11A,SNX1,MARCKSL1,CCR10,AIF1,SEPT1,CDH26,RPS6KA2,ARL2BP,RAP1GDS1,HN1,GNB2L1,VSNL1,MPHOSPH9,ANXA11,DUPD1,TTC1,PPP3R1,S100A7A,GH1,SNX9,SIGLEC5,SRI,YWHAB,ANXA6,NOL3,PLEK,PRKAR2B,ACVR2B,TMIE,CAMK2N1,SEPT2,PLEKHG2,ARL5B |
| Cell communication | 53 | 3687 | 32.31707317 | 1.594722189 | 0.000272 | 0.005977 | 0.002988 | 0.001338 | EFCAB2,ADRB2,CARHSP1,CCNB1,OIP5,FST,RAB3B,HPCAL1,GPSM3,DOK1,CKS2,OR10G3,RHOA,PPP2R4,GULP1,TIRAP,CAMK4,RAB24,SFN,CALN1,RAB11A,SNX1,MARCKSL1,CCR10,AIF1,SEPT1,CDH26,RPS6KA2,ARL2BP,RAP1GDS1,HN1,GNB2L1,VSNL1,MPHOSPH9,ANXA11,DUPD1,TTC1,PPP3R1,S100A7A,GH1,SNX9,SIGLEC5,SRI,ANXA6,NOL3,PLEK,PRKAR2B,ACVR2B,TMIE,CAMK2N1,SEPT2,PLEKHG2,ARL5B |

**Supplementary Table 7.5: Biological pathway**

| Analysis:Biological pathway |  |  |  |  |  |  |  |  |  |
| --- | --- | --- | --- | --- | --- | --- | --- | --- | --- |
| Name of data set: MG vs HC |  |  |  |  |  |  |  |  |  |
| Number of gene in data set: 165 |  |  |  |  |  |  |  |  |  |
| Number of gene mapped to Biological pathway : 66 |  |  |  |  |  |  |  |  |  |
|  |  |  |  |  |  |  |  |  |  |
| Biological pathway | No. of genes  in the data set | No. of genes in the background data set | Percentage of genes | Fold Enrichment | Uncorrected  p-value  (Hypergeometric test) | Corrected  p-value  (Bonferroni method) | Corrected  p-value  (BH method) | Storey and Tibshirani method  q-value | Genes mapped from  input data set |

**Supplementary Table 7.6: Protein Domain**

| Analysis:Protein domain |  |  |  |  |  |  |  |  |  |
| --- | --- | --- | --- | --- | --- | --- | --- | --- | --- |
| Name of data set: MG vs HC |  |  |  |  |  |  |  |  |  |
| Number of gene in data set: 165 |  |  |  |  |  |  |  |  |  |
| Number of gene mapped to Protein domain : 41 |  |  |  |  |  |  |  |  |  |
|  |  |  |  |  |  |  |  |  |  |
| Protein domain | No. of genes  in the data set | No. of genes in the background data set | Percentage of genes | Fold Enrichment | Uncorrected  p-value  (Hypergeometric test) | Corrected  p-value  (Bonferroni method) | Corrected  p-value  (BH method) | Storey and Tibshirani method  q-value | Genes mapped from  input data set |

**Supplementary Table 7.7: Site of expression**

| Analysis:Site of expression |  |  |  |  |  |  |  |  |  |
| --- | --- | --- | --- | --- | --- | --- | --- | --- | --- |
| Name of data set: MG vs HC |  |  |  |  |  |  |  |  |  |
| Number of gene in data set: 165 |  |  |  |  |  |  |  |  |  |
| Number of gene mapped to Site of expression : 163 |  |  |  |  |  |  |  |  |  |
|  |  |  |  |  |  |  |  |  |  |
| Site of expression | No. of genes  in the data set | No. of genes in the background data set | Percentage of genes | Fold Enrichment | Uncorrected  p-value  (Hypergeometric test) | Corrected  p-value  (Bonferroni method) | Corrected  p-value  (BH method) | Storey and Tibshirani method  q-value | Genes mapped from  input data set |
| OVCAR3 | 67 | 3644 | 41.10429448 | 2.056765247 | 7.83E-10 | 2.51E-07 | 2.51E-07 | 7.92E-08 | ADRB2,CARHSP1,PSMD6,DRG1,CCNB1,COX4I1,DOHH,ARPC3,PROSC,FST,CCDC28A,MRPL13,CORO1A,SLC39A9,MAPK3,MRPS7,DTD1,HPCAL1,PRPSAP2,CKS2,TIMP1,PPP2R4,EPS8L1,IFIT3,CCT4,DDI2,HSPA2,GALT,ABLIM1,PRKRA,PAIP1,UGDH,IMPDH2,ANP32E,UBE2V2,ATG3,CLGN,SFN,RAB11A,SNX1,RBBP7,MARCKSL1,NME1,RAP1GDS1,HN1,GNB2L1,FN3K,NAP1L1,FABP5,ANXA11,BLOC1S2,TARDBP,TTC1,SRI,YWHAB,ANXA6,TPD52L2,NOL3,SRPK2,USP15,COASY,SEPT2,SNURF,PLEKHG2,PAIP2,RTN4,GBE1 |
| 041803_BALF3 | 32 | 1072 | 19.63190184 | 3.403299272 | 1.79E-09 | 5.73E-07 | 2.87E-07 | 8.01E-08 | PSMD6,DRG1,DOHH,CORO1A,EIF4EBP3,HLA-DRB5,IFIT3,CCT4,PRKRA,UGDH,IMPDH2,ANP32E,ATG3,RBBP7,MARCKSL1,AIF1,NME1,RAP1GDS1,GNB2L1,HLA-DRB3,NAP1L1,FABP5,ANXA11,BLOC1S2,SRI,ANXA6,TPD52L2,USP15,PLEK,SEPT2,RTN4,GBE1 |
| Erythrocytes | 38 | 1492 | 23.31288344 | 2.887463652 | 3.46E-09 | 1.11E-06 | 3.7E-07 | 8.01E-08 | STAT6,CARHSP1,PSMD6,DOHH,PROSC,DTD1,HPCAL1,ZFYVE19,PRPSAP2,PPP2R4,CCT4,DDI2,CDC34,HSPA2,GALT,PAIP1,ANP32E,HBG2,ATG3,SFN,SNX1,RBBP7,NME1,FN3K,NAP1L1,FABP5,ANXA11,TARDBP,ZMYM3,SNX9,SRI,YWHAB,TPD52L2,USP15,PRKAR2B,COASY,SEPT2,GBE1 |
| CD8 | 61 | 3259 | 37.42331288 | 2.097480059 | 3.67E-09 | 1.18E-06 | 2.94E-07 | 8.01E-08 | STAT6,CARHSP1,PSMD6,DRG1,CCNB1,COX4I1,DOHH,ARPC3,PROSC,MRPL13,CORO1A,MAPK3,MRPS7,HPCAL1,GPSM3,EIF4EBP3,PRPSAP2,HLA-DRB5,PPP2R4,IFIT3,CCT4,HSPA2,UGDH,IMPDH2,ANP32E,UBE2V2,HBG2,ATG3,CLGN,ASF1A,SNX1,RBBP7,CKAP2,MARCKSL1,AIF1,ARL2BP,NME1,RAP1GDS1,HN1,GNB2L1,ABCF3,HLA-DRB3,NAP1L1,FABP5,ANXA11,TARDBP,TTC1,ZMYM3,SNX9,SRI,YWHAB,ANXA6,TPD52L2,SRPK2,USP15,PLEK,MLX,PRKAR2B,COASY,SEPT2,RTN4 |
| Dendritic cells | 44 | 1923 | 26.99386503 | 2.58325023 | 3.96E-09 | 1.27E-06 | 2.54E-07 | 8.01E-08 | CARHSP1,DRG1,COX4I1,ARPC3,PROSC,CORO1A,MAPK3,MRPS7,DTD1,HPCAL1,GPSM3,TIMP1,RHOA,PPP2R4,IFIT3,CCT4,ANP32E,UBE2V2,ATG3,RAB11A,SNX1,RBBP7,AIF1,RPS6KA2,NME1,RAP1GDS1,HN1,GNB2L1,ABCF3,HLA-DRB3,ATP6V1E2,NAP1L1,FABP5,ANXA11,TARDBP,LST1,SRI,YWHAB,ANXA6,TPD52L2,USP15,PLEK,SEPT2,RTN4 |
| HCT116 | 52 | 2596 | 31.90184049 | 2.252242998 | 9.61E-09 | 3.08E-06 | 5.14E-07 | 1.51E-07 | CARHSP1,PSMD6,DRG1,COX4I1,DOHH,ARPC3,PROSC,KRR1,RAB3B,MAPK3,MRPS7,DTD1,PRPSAP2,RHOA,PPP2R4,CCT4,HSPA2,PRKRA,PAIP1,UGDH,IMPDH2,ANP32E,UBE2V2,FAIM,HBG2,ATG3,SFN,RAB11A,SNX1,RBBP7,MARCKSL1,NME1,RAP1GDS1,GNB2L1,ABCF3,NAP1L1,FABP5,ANXA11,TARDBP,ATP6V1C2,LST1,COG3,SRI,YWHAB,ANXA6,TPD52L2,USP15,PRKAR2B,SEPT2,SNURF,RTN4,GBE1 |
| 031003_BALF2 | 31 | 1090 | 19.01840491 | 3.246495839 | 1.05E-08 | 3.36E-06 | 4.8E-07 | 1.51E-07 | DOHH,CORO1A,MAPK3,TIMP1,PPP2R4,IFIT3,CCT4,HSPA2,GALT,PAIP1,UGDH,IMPDH2,ANP32E,HBG2,SFN,RBBP7,MARCKSL1,NME1,GNB2L1,NAP1L1,FABP5,BLOC1S2,TARDBP,SRI,YWHAB,ANXA6,NOL3,USP15,SEPT2,RTN4,GBE1 |
| B Cell | 45 | 2194 | 27.60736196 | 2.314393173 | 7.04E-08 | 2.26E-05 | 2.82E-06 | 8.89E-07 | DRG1,COX4I1,DOHH,ARPC3,PROSC,RAB3B,CORO1A,MAPK3,HPCAL1,GPSM3,TIMP1,HLA-DRB5,RHOA,PPP2R4,LCN1,IFIT3,CCT4,HSPA2,GALT,PAIP1,UGDH,IMPDH2,ANP32E,UBE2V2,HBG2,SFN,RBBP7,MARCKSL1,NME1,GNB2L1,HLA-DRB3,NAP1L1,FABP5,ANXA11,BLOC1S2,TARDBP,GH1,SRI,YWHAB,ANXA6,TPD52L2,PLEK,COASY,SEPT2,RTN4 |
| Lens | 28 | 1028 | 17.17791411 | 3.1218669 | 1.49E-07 | 4.77E-05 | 5.3E-06 | 1.67E-06 | CARHSP1,DRG1,DOHH,CORO1A,HLA-DRB5,IFIT3,CCT4,UGDH,IMPDH2,ANP32E,UBE2V2,SFN,RBBP7,NME1,RAP1GDS1,GNB2L1,HLA-DRB3,NAP1L1,FABP5,ANXA11,TARDBP,SRI,YWHAB,ANXA6,SRPK2,PLEK,PAIP2,GBE1 |
| HTB75 | 31 | 1241 | 19.01840491 | 2.851872477 | 1.98E-07 | 6.37E-05 | 6.37E-06 | 2.01E-06 | CARHSP1,PROSC,DTD1,TIMP1,RHOA,CCT4,HSPA2,UGDH,IMPDH2,UBE2V2,SFN,RAB11A,SNX1,RBBP7,MARCKSL1,NME1,GNB2L1,NAP1L1,FABP5,ANXA11,TARDBP,TTC1,SRI,YWHAB,ANXA6,TPD52L2,NOL3,SEPT2,SNURF,PAIP2,RTN4 |
| CaOV3 | 57 | 3291 | 34.96932515 | 1.943625157 | 2.54E-07 | 8.17E-05 | 7.42E-06 | 2.34E-06 | CARHSP1,PSMD6,DRG1,DOHH,ARPC3,PROSC,KRR1,RAB3B,MRPL13,MAPK3,MRPS7,DTD1,HPCAL1,CKS2,TIMP1,HLA-DRB5,PPP2R4,EPS8L1,IFIT3,CCT4,DDI2,HSPA2,PRKRA,PAIP1,UGDH,IMPDH2,ANP32E,UBE2V2,ATG3,SFN,RAB11A,SNX1,RBBP7,MARCKSL1,NME1,HN1,GNB2L1,FN3K,ABCF3,HLA-DRB3,NAP1L1,FABP5,ANXA11,BLOC1S2,TARDBP,TTC1,SNX9,SRI,YWHAB,ANXA6,TPD52L2,NOL3,SEPT2,SNURF,PAIP2,RTN4,GBE1 |
| CD4 | 39 | 1854 | 23.92638037 | 2.383267731 | 3.62E-07 | 0.000116 | 9.68E-06 | 3.05E-06 | CARHSP1,PSMD6,COX4I1,ARPC3,PROSC,MRPL13,CORO1A,MAPK3,HPCAL1,GPSM3,PRPSAP2,TIMP1,HLA-DRB5,PPP2R4,CCT4,DDI2,GALT,IMPDH2,ANP32E,UBE2V2,SFN,SNX1,RBBP7,ARL2BP,NME1,HN1,GNB2L1,HLA-DRB3,NAP1L1,FABP5,ANXA11,TARDBP,SRI,YWHAB,ANXA6,TPD52L2,PLEK,SEPT2,RTN4 |
| H358 | 33 | 1448 | 20.24539877 | 2.596131311 | 6.11E-07 | 0.000196 | 1.51E-05 | 4.51E-06 | CARHSP1,PSMD6,COX4I1,ARPC3,HPCAL1,CKS2,TIMP1,RHOA,PPP2R4,CCT4,GALT,UGDH,IMPDH2,UBE2V2,SFN,RAB11A,RBBP7,MARCKSL1,AIF1,NME1,HN1,GNB2L1,NAP1L1,FABP5,TARDBP,SNX9,SRI,YWHAB,TPD52L2,USP15,SEPT2,RTN4,GBE1 |
| R | 39 | 1894 | 23.92638037 | 2.332967924 | 6.25E-07 | 0.000201 | 1.43E-05 | 4.51E-06 | CARHSP1,PSMD6,DRG1,CCNB1,DOHH,ARPC3,HPCAL1,EIF4EBP3,PRPSAP2,TIMP1,PPP2R4,CCT4,DDI2,UGDH,IMPDH2,ANP32E,HBG2,ATG3,ASF1A,SNX1,RBBP7,MARCKSL1,NME1,RAP1GDS1,HN1,GNB2L1,ABCF3,NAP1L1,ANXA11,TTC1,SNX9,YWHAB,ANXA6,TPD52L2,USP15,PRKAR2B,COASY,SEPT2,RTN4 |
| K-562 | 46 | 2483 | 28.2208589 | 2.089372637 | 9.49E-07 | 0.000305 | 2.03E-05 | 6.4E-06 | CARHSP1,PSMD6,DRG1,CCNB1,COX4I1,ARPC3,PROSC,MAPK3,HPCAL1,DOK1,PRPSAP2,RHOA,PPP2R4,GADD45A,CCT4,PAIP1,IMPDH2,ANP32E,UBE2V2,HBG2,ASF1A,SNX1,RBBP7,MARCKSL1,RPS6KA2,NME1,HN1,GNB2L1,FN3K,ABCF3,NAP1L1,FABP5,ANXA11,TARDBP,TTC1,ZMYM3,SNX9,SRI,YWHAB,TPD52L2,SRPK2,USP15,PRKAR2B,ACVR2B,SEPT2,RTN4 |
| global_SCX_fractionated | 45 | 2427 | 27.60736196 | 2.092318177 | 1.28E-06 | 0.00041 | 2.56E-05 | 8.06E-06 | CARHSP1,PSMD6,COX4I1,DOHH,ARPC3,PROSC,MRPL13,CORO1A,DTD1,HPCAL1,ZFYVE19,EIF4EBP3,PRPSAP2,PPP2R4,CCT4,SULT1E1,GALT,PRKRA,PAIP1,UGDH,IMPDH2,ANP32E,ATG3,SFN,RAB11A,SNX1,RBBP7,NME1,RAP1GDS1,HN1,GNB2L1,ABCF3,NAP1L1,FABP5,ANXA11,BLOC1S2,TARDBP,COG3,SRI,YWHAB,TPD52L2,USP15,SEPT2,RTN4,GBE1 |
| ES2 | 57 | 3469 | 34.96932515 | 1.843930531 | 1.51E-06 | 0.000485 | 2.85E-05 | 8.98E-06 | HOXA5,CARHSP1,PSMD6,DRG1,CCNB1,COX4I1,DOHH,ARPC3,PROSC,KRR1,FST,RAB3B,MRPL13,SLC39A9,MAPK3,MRPS7,DTD1,HPCAL1,ZFYVE19,EIF4EBP3,CKS2,TIMP1,PPP2R4,CCT4,PRKRA,PAIP1,UGDH,IMPDH2,ANP32E,UBE2V2,HBG2,ATG3,SFN,RAB11A,SNX1,RBBP7,MARCKSL1,C14orf119,NME1,RAP1GDS1,HN1,GNB2L1,NAP1L1,FABP5,ANXA11,TARDBP,TTC1,SNX9,SRI,YWHAB,ANXA6,TPD52L2,COASY,SEPT2,SNURF,RTN4,GBE1 |
| H293 | 90 | 6698 | 55.21472393 | 1.496291044 | 1.84E-06 | 0.000589 | 3.27E-05 | 1.03E-05 | HOXA5,STAT6,CARHSP1,PSMD6,DRG1,CCNB1,COX4I1,DOHH,COQ6,ARPC3,PROSC,OIP5,KRR1,MRPL13,ZHX3,CORO1A,SLC39A9,MAPK3,MRPS7,DTD1,HPCAL1,C2orf44,ZFYVE19,EIF4EBP3,PRPSAP2,CKS2,TIMP1,HLA-DRB5,PPP2R4,STAU2,GULP1,RNF11,TIPIN,CCT4,DDI2,CDC34,HSPA2,GALT,ABLIM1,PRKRA,PAIP1,CAMK4,UGDH,RAB24,IMPDH2,ANP32E,UBE2V2,HBG2,ATG3,CLGN,SFN,ASF1A,RAB11A,SNX1,RBBP7,CKAP2,PDLIM3,MARCKSL1,NME1,RAP1GDS1,HN1,GNB2L1,ABCF3,HLA-DRB3,NAP1L1,FABP5,ANXA11,BLOC1S2,TARDBP,TTC1,PPP3R1,ZMYM3,SNX9,COG3,SRI,YWHAB,ANXA6,TPD52L2,NOL3,SRPK2,USP15,PLEK,MLX,PRKAR2B,COASY,SEPT2,PAIP2,RTN4,GBE1,ARL5B |
| PC3 | 29 | 1244 | 17.79141104 | 2.66856765 | 2.13E-06 | 0.000683 | 3.6E-05 | 1.13E-05 | CARHSP1,PSMD6,CCNB1,FST,RAB3B,DTD1,HPCAL1,TIMP1,RHOA,CCT4,HSPA2,UGDH,IMPDH2,UBE2V2,SFN,RBBP7,MARCKSL1,NME1,HN1,GNB2L1,NAP1L1,FABP5,ANXA11,TARDBP,TTC1,YWHAB,SEPT2,RTN4,GBE1 |
| Leukocytes | 49 | 2827 | 30.06134969 | 1.951765597 | 2.6E-06 | 0.000835 | 4.17E-05 | 1.31E-05 | KCNMB3,PSMD6,DRG1,ARPC3,PROSC,CORO1A,DOK1,EIF4EBP3,PRPSAP2,RHOA,PPP2R4,CCT4,ABLIM1,TRIM68,UGDH,RAB24,IMPDH2,ANP32E,ATG3,P2RX7,ASF1A,SNX1,CKAP2,MARCKSL1,AIF1,NME1,RAP1GDS1,HN1,GNB2L1,NAP1L1,ANXA11,TARDBP,SNX9,SIGLEC5,LST1,COG3,SRI,YWHAB,ANXA6,TPD52L2,CLP1,USP15,PLEK,MLX,COASY,SEPT2,PAIP2,RTN4,GBE1 |
| SH-SY5Y | 17 | 524 | 10.42944785 | 3.816859696 | 5.5E-06 | 0.001766 | 8.41E-05 | 2.65E-05 | PSMD6,ARPC3,C2orf44,CCT4,HSPA2,ANP32E,UBE2V2,ATG3,RBBP7,NME1,HN1,NAP1L1,FABP5,COG3,SRI,YWHAB,RTN4 |
| Cortex | 19 | 662 | 11.65644172 | 3.353954427 | 8.75E-06 | 0.002809 | 0.000128 | 4.02E-05 | CRYM,COX4I1,ARPC3,MAPK3,HPCAL1,CCT4,HSPA2,UBE2V2,RAB11A,NME1,RAP1GDS1,VSNL1,NAP1L1,SRI,YWHAB,ANXA6,PRKAR2B,SEPT2,RTN4 |
| Jurkat | 47 | 2815 | 28.83435583 | 1.882068826 | 1.26E-05 | 0.004042 | 0.000176 | 5.49E-05 | PSMD6,GSG1,DRG1,CCNB1,COX4I1,DOHH,ARPC3,PROSC,MRPL13,CALCOCO2,CORO1A,SLC39A9,MAPK3,DOK1,PRPSAP2,CKS2,CCT4,HSPA2,PRKRA,CAMK4,IMPDH2,ANP32E,UBE2V2,ASF1A,SNX1,RBBP7,MARCKSL1,RPS6KA2,NME1,RAP1GDS1,HN1,GNB2L1,ABCF3,NAP1L1,FABP5,ANXA11,TARDBP,COG3,LHX1,SRI,YWHAB,ANXA6,TPD52L2,USP15,ACVR2B,SEPT2,RTN4 |
| MDA-MB-468 | 20 | 744 | 12.26993865 | 3.132321547 | 1.3E-05 | 0.004186 | 0.000174 | 5.49E-05 | PSMD6,COX4I1,PROSC,MRPL13,CORO1A,DTD1,EIF4EBP3,RHOA,CCT4,IMPDH2,SFN,RAB11A,RBBP7,NME1,GNB2L1,NAP1L1,TTC1,S100A7A,SEPT2,SNURF |
| HEK293 | 64 | 4377 | 39.26380368 | 1.637153301 | 1.44E-05 | 0.004607 | 0.000184 | 5.79E-05 | HOXA5,CARHSP1,PSMD6,DRG1,CCNB1,COX4I1,DOHH,ARPC3,OIP5,KRR1,MRPL13,MRPS7,DTD1,ZFYVE19,EIF4EBP3,PRPSAP2,PPP2R4,STAU2,CCT4,DDI2,CDC34,HSPA2,ABLIM1,PRKRA,PAIP1,UGDH,RAB24,IMPDH2,ANP32E,CLGN,SFN,ASF1A,SNX1,RBBP7,CKAP2,PDLIM3,MARCKSL1,NME1,RAP1GDS1,HN1,GNB2L1,ABCF3,NAP1L1,FABP5,ANXA11,BLOC1S2,TARDBP,TTC1,PPP3R1,ZMYM3,SNX9,COG3,SRI,YWHAB,ANXA6,TPD52L2,SRPK2,USP15,PRKAR2B,COASY,SEPT2,PAIP2,RTN4,GBE1 |
| Brain | 92 | 7215 | 56.44171779 | 1.419536809 | 1.49E-05 | 0.004777 | 0.000184 | 5.79E-05 | CRYM,KCNMB3,CARHSP1,PSMD6,DRG1,COX4I1,ARPC3,PROSC,RAB3B,CALCOCO2,ZHX3,CORO1A,DNAJB5,MAPK3,DTD1,HPCAL1,EIF4EBP3,PRPSAP2,RHOA,PPP2R4,STAU2,GULP1,RNF11,BHMT2,IFIT3,CCT4,SULT1E1,LTC4S,HSPA2,MIPOL1,TIRAP,ABLIM1,PRKRA,CAMK4,UGDH,SPOP,CAPRIN2,SPAG16,RAB24,IMPDH2,ANP32E,UBE2V2,HBG2,ATG3,SFN,KLF11,P2RX7,TEAD3,CALN1,ASF1A,RAB11A,SNX1,RBBP7,CKAP2,MARCKSL1,CDO1,AIF1,SEPT1,RPS6KA2,NME1,RAP1GDS1,HN1,GNB2L1,FN3K,VSNL1,NAP1L1,FABP5,ANXA11,TARDBP,TTC1,PPP3R1,ZMYM3,SNX9,COG3,LHX1,SRI,YWHAB,ANXA6,TPD52L2,NOL3,SRPK2,USP15,MLX,PRKAR2B,ACVR2B,COASY,SEPT2,SNURF,PAIP2,RTN4,GBE1,ARL5B |
| A549 | 23 | 965 | 14.11042945 | 2.756967359 | 1.95E-05 | 0.006253 | 0.000232 | 7.29E-05 | CARHSP1,DRG1,ARPC3,MAPK3,EPS8L1,CCT4,HSPA2,PAIP1,UBE2V2,SFN,RPS6KA2,NME1,HN1,GNB2L1,NAP1L1,FABP5,ANXA11,COG3,YWHAB,ANXA6,CLP1,RTN4,GBE1 |
| H460 | 19 | 714 | 11.65644172 | 3.110115727 | 2.51E-05 | 0.008045 | 0.000287 | 9.05E-05 | FST,TIMP1,RHOA,LCN1,CCT4,UGDH,IMPDH2,ANP32E,SFN,RBBP7,NME1,HN1,GNB2L1,NAP1L1,FABP5,YWHAB,SEPT2,SNURF,GBE1 |
| Skeletal muscles | 107 | 8978 | 65.64417178 | 1.324333088 | 2.86E-05 | 0.009165 | 0.000316 | 9.95E-05 | CRYM,KCNMB3,EFCAB2,HOXA5,CARHSP1,PSMD6,DRG1,COX4I1,COQ6,ARPC3,PROSC,FST,MRPL13,CALCOCO2,ZHX3,CORO1A,DNAJB5,SLC39A9,MAPK3,MRPS7,DTD1,DOK1,ZFYVE19,EIF4EBP3,PRPSAP2,RHOA,PPP2R4,STAU2,GULP1,RNF11,DLX5,BHMT2,LCN1,IFIT3,CCT4,CDC34,SULT1E1,LTC4S,HSPA2,MIPOL1,GALT,TIRAP,ABLIM1,PRKRA,TRIM68,CAMK4,UGDH,SPOP,CAPRIN2,SPAG16,RAB24,IMPDH2,ANP32E,UBE2V2,HBG2,ATG3,CLGN,KLF11,TEAD3,ASF1A,RAB11A,SNX1,FAM63A,PCGF3,CKAP2,PDLIM3,LRFN1,C14orf119,CCR10,AIF1,SEPT1,RPS6KA2,NME1,RAP1GDS1,HN1,GNB2L1,FN3K,ABCF3,NAP1L1,FABP5,ANXA11,DUPD1,BLOC1S2,TARDBP,PPP3R1,ZMYM3,SNX9,SIGLEC5,COG3,LHX1,SRI,YWHAB,ANXA6,TPD52L2,NOL3,SRPK2,CLP1,USP15,MLX,FMN1,COASY,ZNF655,SEPT2,SNURF,PAIP2,RTN4,GBE1 |
| Colorectal cancer cells | 16 | 542 | 9.81595092 | 3.488178916 | 3.34E-05 | 0.010718 | 0.000357 | 0.000113 | COX4I1,ARPC3,HPCAL1,RHOA,CCT4,HSPA2,UGDH,UBE2V2,SFN,RAB11A,MARCKSL1,NME1,GNB2L1,ANXA11,YWHAB,ANXA6 |
| HeLa | 76 | 5699 | 46.62576687 | 1.488723258 | 3.96E-05 | 0.012714 | 0.00041 | 0.000129 | CARHSP1,PSMD6,CCNB1,COX4I1,DOHH,COQ6,ARPC3,PROSC,KRR1,RAB3B,CCDC28A,CALCOCO2,MAPK3,MRPS7,DTD1,HPCAL1,ZFYVE19,EIF4EBP3,PPP2R4,STAU2,TIPIN,LCN1,CCT4,DDI2,NMRAL1,HSPA2,MIPOL1,MTL5,PRKRA,PAIP1,CAMK4,UGDH,CAPRIN2,IMPDH2,ANP32E,UBE2V2,HBG2,ATG3,SFN,ASF1A,RAB11A,SNX1,RBBP7,CKAP2,MARCKSL1,CDO1,AIF1,NME1,RAP1GDS1,HN1,GNB2L1,FN3K,ABCF3,NAP1L1,FABP5,ANXA11,TARDBP,TTC1,S100A7A,ZMYM3,GH1,ATP6V1C2,SRI,YWHAB,ANXA6,TPD52L2,NOL3,SRPK2,USP15,ACVR2B,COASY,ZNF655,SEPT2,SNURF,PAIP2,GBE1 |
| Sy5y | 41 | 2447 | 25.15337423 | 1.89574683 | 5.08E-05 | 0.016306 | 0.00051 | 0.000156 | DRG1,COX4I1,ARPC3,PROSC,MRPL13,CORO1A,MAPK3,MRPS7,DTD1,ZFYVE19,PRPSAP2,RHOA,LCN1,CCT4,ABLIM1,UGDH,IMPDH2,UBE2V2,SFN,RAB11A,SNX1,RBBP7,CKAP2,NME1,C14orf80,GNB2L1,VSNL1,NAP1L1,FABP5,ANXA11,BLOC1S2,TARDBP,TTC1,YWHAB,ANXA6,TPD52L2,SRPK2,COASY,SEPT2,SNURF,RTN4 |
| TOV112D | 26 | 1244 | 15.95092025 | 2.403916313 | 5.08E-05 | 0.016309 | 0.000494 | 0.000156 | CARHSP1,DOHH,PROSC,HPCAL1,TIMP1,RHOA,PPP2R4,CCT4,UGDH,ANP32E,UBE2V2,SFN,RAB11A,RBBP7,NME1,HN1,GNB2L1,FABP5,TARDBP,TTC1,SRI,YWHAB,TPD52L2,SEPT2,RTN4,GBE1 |
| CRC | 60 | 4170 | 36.80981595 | 1.61305532 | 5.29E-05 | 0.016966 | 0.000499 | 0.000157 | CARHSP1,PSMD6,DRG1,COX4I1,ARPC3,PROSC,RAB3B,MRPL13,CORO1A,KLK1,MAPK3,DTD1,HPCAL1,TIMP1,RHOA,PPP2R4,CCT4,DDI2,HSPA2,PRKRA,PAIP1,UGDH,IMPDH2,ANP32E,UBE2V2,HBG2,ATG3,SFN,TEAD3,ASF1A,RAB11A,SNX1,RBBP7,CKAP2,MARCKSL1,ARL2BP,NME1,HN1,GNB2L1,VSNL1,ATP6V1E2,NAP1L1,FABP5,ANXA11,BLOC1S2,TARDBP,TTC1,PPP3R1,ZMYM3,COG3,SRI,YWHAB,ANXA6,TPD52L2,NOL3,COASY,SEPT2,PAIP2,RTN4,GBE1 |
| Platelets | 37 | 2138 | 22.6993865 | 1.964170966 | 6.69E-05 | 0.02149 | 0.000614 | 0.000193 | COX4I1,ARPC3,RAB3B,CALCOCO2,CORO1A,DTD1,HPCAL1,TIMP1,RHOA,PPP2R4,CCT4,LTC4S,HSPA2,PAIP1,UBE2V2,HBG2,SFN,RAB11A,FAM63A,NME1,RAP1GDS1,GNB2L1,FN3K,NAP1L1,FABP5,ANXA11,TARDBP,PPP3R1,SRI,YWHAB,TPD52L2,PLEK,PRKAR2B,COASY,SEPT2,RTN4,GBE1 |
| H23 | 20 | 855 | 12.26993865 | 2.726262929 | 9.2E-05 | 0.02953 | 0.00082 | 0.000258 | CARHSP1,DOHH,TIMP1,CCT4,IMPDH2,ANP32E,SFN,RBBP7,NME1,HN1,GNB2L1,NAP1L1,FABP5,ANXA11,TARDBP,YWHAB,TPD52L2,SEPT2,SNURF,RTN4 |
| TOV21G | 20 | 873 | 12.26993865 | 2.670131694 | 0.000122 | 0.03913 | 0.001058 | 0.000333 | PROSC,TIMP1,RHOA,CCT4,UGDH,IMPDH2,ANP32E,SFN,RAB11A,RBBP7,NME1,HN1,GNB2L1,FABP5,TARDBP,TTC1,YWHAB,TPD52L2,SEPT2,RTN4 |
| Ascites cancer cell | 51 | 3445 | 31.28834356 | 1.665516364 | 0.000126 | 0.04056 | 0.001067 | 0.000336 | CARHSP1,PSMD6,DRG1,COX4I1,PROSC,FST,MRPL13,CORO1A,MAPK3,MRPS7,HPCAL1,EIF4EBP3,TSPAN17,TIMP1,HLA-DRB5,EPS8L1,STAU2,IFIT3,CCT4,HSPA2,ABLIM1,PAIP1,UGDH,IMPDH2,ANP32E,SFN,RAB11A,SNX1,RBBP7,MARCKSL1,NME1,HN1,GNB2L1,HLA-DRB3,NAP1L1,FABP5,ANXA11,TARDBP,TTC1,ZMYM3,LST1,SRI,YWHAB,ANXA6,TPD52L2,SRPK2,PLEK,SEPT2,SNURF,PAIP2,RTN4 |
| H520 | 19 | 823 | 11.65644172 | 2.698829571 | 0.000165 | 0.05297 | 0.001358 | 0.000428 | CARHSP1,PROSC,CCT4,IMPDH2,UBE2V2,HBG2,SFN,RBBP7,MARCKSL1,NME1,HN1,GNB2L1,VSNL1,NAP1L1,FABP5,TARDBP,YWHAB,TPD52L2,SNURF |
| Endometrium | 24 | 1192 | 14.72392638 | 2.324552332 | 0.00018 | 0.057891 | 0.001447 | 0.000456 | STAT6,DRG1,CCNB1,COX4I1,KLK1,MAPK3,TIMP1,RHOA,SULT1E1,HSPA2,MIPOL1,IMPDH2,SFN,C14orf119,NME1,GNB2L1,ANXA11,TARDBP,PPP3R1,ZMYM3,LHX1,SRI,YWHAB,ANXA6 |
| Monocyte | 43 | 2786 | 26.3803681 | 1.744001039 | 0.000212 | 0.068094 | 0.001661 | 0.000523 | DRG1,COX4I1,ARPC3,PROSC,RAB3B,MRPL13,ZHX3,CORO1A,HPCAL1,EIF4EBP3,PRPSAP2,HLA-DRB5,PPP2R4,CCT4,PRKRA,RAB24,IMPDH2,ANP32E,UBE2V2,CLGN,SFN,RBBP7,MARCKSL1,AIF1,ARL2BP,NME1,GNB2L1,HLA-DRB3,NAP1L1,FABP5,ANXA11,TARDBP,PPP3R1,SRI,YWHAB,ANXA6,TPD52L2,USP15,PLEK,MLX,COASY,SEPT2,RTN4 |
| Parathyroid | 18 | 776 | 11.04294479 | 2.720692074 | 0.000234 | 0.074954 | 0.001785 | 0.000562 | STAT6,DRG1,COX4I1,MAPK3,RHOA,HSPA2,MIPOL1,CAMK4,IMPDH2,C14orf119,NME1,GNB2L1,ANXA11,TARDBP,PPP3R1,ZMYM3,LHX1,ANXA6 |
| 050603_BALF11 | 21 | 994 | 12.88343558 | 2.455880424 | 0.000249 | 0.080048 | 0.001862 | 0.000586 | COX4I1,ARPC3,MRPL13,CORO1A,MRPS7,HPCAL1,TIMP1,HLA-DRB5,CCT4,IMPDH2,RBBP7,MARCKSL1,NME1,GNB2L1,HLA-DRB3,NAP1L1,ANXA11,YWHAB,ANXA6,SEPT2,RTN4 |
| Nuclear | 25 | 1305 | 15.33742331 | 2.207559372 | 0.00028 | 0.089961 | 0.002045 | 0.000644 | KRR1,MAPK3,PPP2R4,GULP1,CCT4,DDI2,PRKRA,PAIP1,UGDH,IMPDH2,ANP32E,RBBP7,MARCKSL1,NME1,GNB2L1,BLOC1S2,TARDBP,PPP3R1,COG3,ANXA6,SRPK2,USP15,SEPT2,RTN4,GBE1 |
| Placenta | 119 | 10796 | 73.00613497 | 1.223436171 | 0.00029 | 0.093055 | 0.002068 | 0.000651 | KCNMB3,EFCAB2,HOXA5,ADRB2,STAT6,CARHSP1,PSMD6,DRG1,CCNB1,COX4I1,COQ6,ARPC3,PROSC,FST,RAB3B,MRPL13,CALCOCO2,ZHX3,CORO1A,DNAJB5,SLC39A9,MAPK3,MRPS7,HPCAL1,DOK1,ZFYVE19,EIF4EBP3,PRPSAP2,HLA-DRB5,RHOA,PPP2R4,EPS8L1,STAU2,GULP1,RNF11,DLX5,LCN1,IFIT3,CCT4,DDI2,CDC34,SULT1E1,LTC4S,HSPA2,MIPOL1,GALT,TIRAP,ABLIM1,PRKRA,PAIP1,TRIM68,CAMK4,UGDH,SPOP,CAPRIN2,IMPDH2,ANP32E,UBE2V2,HBG2,ATG3,CLGN,SFN,KLF11,P2RX7,TEAD3,ASF1A,RAB11A,SNX1,FAM63A,RBBP7,PCGF3,CKAP2,PDLIM3,MARCKSL1,C14orf119,CCR10,AIF1,CDH26,RPS6KA2,NME1,RAP1GDS1,HN1,GNB2L1,FN3K,ABCF3,NAP1L1,FABP5,ANXA11,BLOC1S2,TARDBP,TTC1,PPP3R1,ZMYM3,GH1,ATP6V1C2,SNX9,SIGLEC5,COG3,LHX1,SRI,YWHAB,ANXA6,TPD52L2,NOL3,SRPK2,CLP1,USP15,PLEK,MLX,FMN1,PRKAR2B,ACVR2B,COASY,ZNF655,SEPT2,SNURF,PAIP2,RTN4,GBE1 |
| Melanoma | 31 | 1795 | 19.01840491 | 1.972297055 | 0.000307 | 0.098529 | 0.002142 | 0.000674 | PSMD6,DRG1,COX4I1,ARPC3,CALCOCO2,CORO1A,DTD1,HPCAL1,RHOA,PPP2R4,CCT4,HSPA2,PRKRA,UGDH,IMPDH2,SFN,RAB11A,SNX1,AIF1,NME1,RAP1GDS1,GNB2L1,HLA-DRB3,NAP1L1,FABP5,YWHAB,ANXA6,TPD52L2,NOL3,SEPT2,RTN4 |
| LNCaP | 26 | 1398 | 15.95092025 | 2.139343783 | 0.000331 | 0.106279 | 0.002261 | 0.000712 | CARHSP1,ARPC3,DTD1,HPCAL1,RHOA,PPP2R4,CCT4,HSPA2,UGDH,SPOP,IMPDH2,UBE2V2,RAB11A,RBBP7,MARCKSL1,NME1,HN1,GNB2L1,NAP1L1,TARDBP,TTC1,YWHAB,SEPT2,SNURF,PAIP2,RTN4 |
| Neutrophil | 33 | 1979 | 20.24539877 | 1.899983993 | 0.00036 | 0.115498 | 0.002406 | 0.000758 | PSMD6,COX4I1,ARPC3,CORO1A,HPCAL1,EIF4EBP3,TIMP1,HLA-DRB5,PPP2R4,CCT4,PRKRA,IMPDH2,ANP32E,UBE2V2,HBG2,SFN,RBBP7,MARCKSL1,NME1,GNB2L1,HLA-DRB3,FABP5,ANXA11,TARDBP,SIGLEC5,SRI,YWHAB,ANXA6,PLEK,MLX,SEPT2,RTN4,GBE1 |
| H1688 | 20 | 951 | 12.26993865 | 2.451417835 | 0.000375 | 0.120312 | 0.002455 | 0.000773 | CARHSP1,FST,TIMP1,RHOA,CCT4,UGDH,IMPDH2,ANP32E,UBE2V2,SFN,RBBP7,NME1,HN1,GNB2L1,NAP1L1,FABP5,TARDBP,YWHAB,USP15,SEPT2 |
| Uterine cervix | 19 | 893 | 11.65644172 | 2.487570896 | 0.000459 | 0.147316 | 0.002946 | 0.000928 | STAT6,DRG1,CCNB1,COX4I1,MAPK3,RHOA,HSPA2,MIPOL1,CAMK4,IMPDH2,C14orf119,NME1,GNB2L1,ANXA11,TARDBP,PPP3R1,ZMYM3,LHX1,YWHAB |
| Oesophagus | 19 | 914 | 11.65644172 | 2.4304947 | 0.000609 | 0.195428 | 0.003832 | 0.001201 | KCNMB3,STAT6,DRG1,CCNB1,COX4I1,MAPK3,HSPA2,MIPOL1,IMPDH2,C14orf119,NME1,GNB2L1,NAP1L1,TARDBP,PPP3R1,ZMYM3,LHX1,YWHAB,PAIP2 |
| Urine | 46 | 3202 | 28.2208589 | 1.62039303 | 0.000618 | 0.198392 | 0.003815 | 0.001201 | CRYM,CARHSP1,ARPC3,RAB3B,CORO1A,KLK1,MAPK3,PRPSAP2,METTL8,OR10G3,TIMP1,RHOA,PPP2R4,EPS8L1,RNF11,BHMT2,TIPIN,LCN1,IFIT3,HSPA2,GALT,ABLIM1,CAMK4,UGDH,IMPDH2,UBE2V2,HBG2,SFN,RAB11A,NME1,RAP1GDS1,GNB2L1,NAP1L1,MPHOSPH9,FABP5,ANXA11,ATP6V1C2,SNX9,SRI,YWHAB,ANXA6,PRKAR2B,COASY,SEPT2,RTN4,GBE1 |
| Ramos | 27 | 1546 | 16.56441718 | 2.005706348 | 0.000671 | 0.21526 | 0.004062 | 0.001279 | CCNB1,COX4I1,ARPC3,PROSC,CALCOCO2,CORO1A,MAPK3,HPCAL1,DOK1,RHOA,CCT4,CAMK4,ANP32E,SFN,SNX1,CKAP2,NME1,GNB2L1,FABP5,TARDBP,S100A7A,COG3,SRI,YWHAB,ACVR2B,COASY,SEPT2 |
|  | 22 | 1163 | 13.49693252 | 2.193744773 | 0.000794 | 0.254963 | 0.004722 | 0.001487 | ARPC3,PROSC,RAB3B,MAPK3,RHOA,EPS8L1,CCT4,LTC4S,HSPA2,SNX1,RPS6KA2,NME1,GNB2L1,FABP5,ANXA11,GH1,SRI,YWHAB,TPD52L2,ACVR2B,SEPT2,RTN4 |
| Thyroid | 18 | 882 | 11.04294479 | 2.394178222 | 0.00105 | 0.33711 | 0.006129 | 0.00193 | STAT6,DRG1,COX4I1,MAPK3,RHOA,HSPA2,MIPOL1,IMPDH2,C14orf119,NME1,GNB2L1,ANXA11,TARDBP,PPP3R1,ZMYM3,LHX1,YWHAB,ANXA6 |
| BT474 | 13 | 530 | 7.975460123 | 2.946627988 | 0.00107 | 0.343521 | 0.006134 | 0.001932 | CARHSP1,ARPC3,TIMP1,UGDH,IMPDH2,HBG2,RBBP7,NME1,HN1,GNB2L1,NAP1L1,YWHAB,SEPT2 |
| T cells | 28 | 1694 | 17.17791411 | 1.895402747 | 0.001234 | 0.3961 | 0.006949 | 0.002188 | STAT6,DRG1,COX4I1,ARPC3,PROSC,CORO1A,MRPS7,HPCAL1,EIF4EBP3,CCT4,ABLIM1,IMPDH2,UBE2V2,HBG2,RBBP7,CCR10,SEPT1,NME1,GNB2L1,NAP1L1,ANXA11,TARDBP,GH1,SNX9,YWHAB,ANXA6,PRKAR2B,SEPT2 |
| Vulva | 17 | 832 | 10.42944785 | 2.406007267 | 0.001432 | 0.459631 | 0.007925 | 0.002495 | STAT6,DRG1,CCNB1,COX4I1,MAPK3,HSPA2,IMPDH2,C14orf119,NME1,GNB2L1,ANXA11,TARDBP,PPP3R1,ZMYM3,LHX1,YWHAB,ANXA6 |
| Ovary | 96 | 8506 | 58.89570552 | 1.25576913 | 0.00164 | 0.526569 | 0.008925 | 0.00281 | CRYM,KCNMB3,EFCAB2,STAT6,CARHSP1,PSMD6,DRG1,COX4I1,ARPC3,PROSC,FST,RAB3B,CALCOCO2,ZHX3,CORO1A,DNAJB5,SLC39A9,MAPK3,MRPS7,DTD1,HPCAL1,EIF4EBP3,PRPSAP2,TIMP1,HLA-DRB5,RHOA,PPP2R4,STAU2,GULP1,RNF11,DLX5,CCT4,CDC34,SULT1E1,HSPA2,MIPOL1,GALT,ABLIM1,PRKRA,PAIP1,TRIM68,CAMK4,UGDH,SPAG16,IMPDH2,ANP32E,UBE2V2,HBG2,ATG3,CLGN,SFN,ASF1A,RAB11A,SNX1,RBBP7,PCGF3,CKAP2,PDLIM3,LRFN1,MARCKSL1,C14orf119,CCR10,AIF1,RPS6KA2,NME1,RAP1GDS1,GNB2L1,VSNL1,ABCF3,HLA-DRB3,NAP1L1,FABP5,ANXA11,TARDBP,PPP3R1,ZMYM3,SNX9,COG3,LHX1,SRI,YWHAB,ANXA6,TPD52L2,SRPK2,CLP1,USP15,MLX,FMN1,PRKAR2B,ACVR2B,COASY,ZNF655,SEPT2,PAIP2,RTN4,GBE1 |
| Tears | 12 | 504 | 7.36196319 | 2.880838713 | 0.002127 | 0.682675 | 0.011378 | 0.003583 | CORO1A,MAPK3,TIMP1,PPP2R4,LCN1,SFN,NME1,GNB2L1,FABP5,ANXA11,YWHAB,SEPT2 |
| MDA468 | 15 | 725 | 9.202453988 | 2.457970172 | 0.002423 | 0.777803 | 0.012751 | 0.003965 | PSMD6,TIMP1,RHOA,CCT4,IMPDH2,UBE2V2,SFN,MARCKSL1,NME1,HN1,GNB2L1,ANXA11,TTC1,TPD52L2,SEPT2 |
| CPE_SCX_fractionated | 34 | 2302 | 20.85889571 | 1.681231157 | 0.002432 | 0.780648 | 0.012591 | 0.003965 | DRG1,DOHH,CORO1A,MAPK3,HPCAL1,EIF4EBP3,CCT4,DDI2,GALT,PRKRA,PAIP1,UGDH,IMPDH2,ANP32E,ATG3,SFN,RBBP7,NME1,RAP1GDS1,GNB2L1,NAP1L1,FABP5,ANXA11,TARDBP,PPP3R1,COG3,SRI,YWHAB,NOL3,SRPK2,USP15,SEPT2,RTN4,GBE1 |
| Saliva | 17 | 880 | 10.42944785 | 2.274956659 | 0.00258 | 0.828262 | 0.013147 | 0.00414 | CARHSP1,ARPC3,CORO1A,KLK1,TIMP1,LCN1,HSPA2,CAMK4,ANP32E,UBE2V2,HBG2,SFN,AIF1,NME1,FABP5,YWHAB,ANXA6 |
| B cells | 12 | 528 | 7.36196319 | 2.750200774 | 0.003107 | 0.997431 | 0.015585 | 0.004907 | ARPC3,CORO1A,HLA-DRB5,RHOA,CCT4,IMPDH2,MARCKSL1,ANXA11,SRI,YWHAB,ANXA6,SEPT2 |
| Bladder cancer cells | 9 | 347 | 5.521472393 | 3.233271161 | 0.00436 | 1 | 0.021533 | 0.00678 | RHOA,CCT4,SFN,NME1,GNB2L1,ANXA11,YWHAB,ANXA6,RTN4 |
| MCF10A | 13 | 640 | 7.975460123 | 2.441163538 | 0.005443 | 1 | 0.026471 | 0.008297 | CARHSP1,HPCAL1,TIMP1,ANP32E,UBE2V2,SFN,NME1,HN1,GNB2L1,FABP5,TTC1,SRI,TPD52L2 |
| Heart | 65 | 5462 | 39.87730061 | 1.332122601 | 0.0055 | 1 | 0.02635 | 0.008297 | CRYM,KCNMB3,ADRB2,PSMD6,DRG1,COX4I1,PROSC,FST,CALCOCO2,ZHX3,DNAJB5,DOK1,ZFYVE19,EIF4EBP3,PPP2R4,GULP1,RNF11,BHMT2,CCT4,HSPA2,MIPOL1,TIRAP,ABLIM1,UGDH,SPOP,CAPRIN2,SPAG16,IMPDH2,UBE2V2,ATG3,KLF11,P2RX7,TEAD3,ASF1A,SNX1,CKAP2,PDLIM3,LRFN1,C14orf119,CCR10,AIF1,SEPT1,RPS6KA2,NME1,GNB2L1,FN3K,FABP5,ANXA11,TARDBP,PPP3R1,ZMYM3,SNX9,COG3,LHX1,SRI,ANXA6,NOL3,SRPK2,USP15,MLX,COASY,SEPT2,SNURF,PAIP2,RTN4 |
| Preadipocytes | 11 | 506 | 6.748466258 | 2.652915525 | 0.006347 | 1 | 0.029961 | 0.009434 | ARPC3,TIMP1,UGDH,NME1,GNB2L1,FABP5,GH1,YWHAB,ANXA6,TPD52L2,RTN4 |
| Dermal microvascular endothelial cells | 2 | 14 | 1.226993865 | 23.41115852 | 0.006874 | 1 | 0.03198 | 0.009957 | CCR10,FABP5 |
| Nipple aspirate fluid | 14 | 735 | 8.588957055 | 2.275379936 | 0.006895 | 1 | 0.031621 | 0.009957 | FST,CORO1A,TIMP1,HLA-DRB5,RHOA,TRIM68,HBG2,SFN,RAB11A,MARCKSL1,NME1,HLA-DRB3,S100A7A,YWHAB |
| Bone marrow | 86 | 7744 | 52.7607362 | 1.237483405 | 0.006994 | 1 | 0.031621 | 0.009957 | KCNMB3,EFCAB2,HOXA5,ADRB2,STAT6,PSMD6,DRG1,CCNB1,COX4I1,DOHH,ARPC3,PROSC,FST,CALCOCO2,ZHX3,CORO1A,DNAJB5,SLC39A9,MAPK3,MRPS7,DOK1,PRPSAP2,OR10G3,TIMP1,RHOA,PPP2R4,STAU2,GULP1,DLX5,CCT4,CDC34,SULT1E1,LTC4S,HSPA2,GALT,PRKRA,PAIP1,TRIM68,CAMK4,UGDH,CAPRIN2,IMPDH2,ANP32E,UBE2V2,KLF11,P2RX7,TEAD3,RAB11A,SNX1,FAM63A,PCGF3,CKAP2,PDLIM3,C14orf119,CCR10,RPS6KA2,NME1,RAP1GDS1,GNB2L1,FN3K,ABCF3,HLA-DRB3,NAP1L1,FABP5,ANXA11,TARDBP,PPP3R1,ZMYM3,SNX9,SIGLEC5,LST1,LHX1,SRI,YWHAB,ANXA6,TPD52L2,SRPK2,USP15,PLEK,MLX,FMN1,ACVR2B,COASY,ZNF655,SEPT2,RTN4 |
| HCC78 | 7 | 246 | 4.294478528 | 3.665447779 | 0.007104 | 1 | 0.031674 | 0.009973 | MAPK3,DOK1,PPP2R4,EPS8L1,IFIT3,ABLIM1,SEPT2 |
| MDA231 | 8 | 310 | 4.90797546 | 3.264688587 | 0.00727 | 1 | 0.031967 | 0.010066 | ARPC3,TIMP1,CCT4,SFN,NME1,NAP1L1,YWHAB,GBE1 |
| YTS | 27 | 1856 | 16.56441718 | 1.670926988 | 0.008697 | 1 | 0.037726 | 0.011879 | DRG1,COX4I1,ARPC3,KRR1,CALCOCO2,CORO1A,EIF4EBP3,HLA-DRB5,RHOA,STAU2,CCT4,HSPA2,PRKRA,IMPDH2,RAB11A,SEPT1,NME1,ABCF3,HLA-DRB3,NAP1L1,MPHOSPH9,ANXA11,TARDBP,YWHAB,ANXA6,PLEK,RTN4 |
| Ganglia | 1 | 1 | 0.613496933 | 109.8523592 | 0.009034 | 1 | 0.038668 | 0.012015 | RTN4 |
| Retinal astrocyte | 1 | 1 | 0.613496933 | 109.8523592 | 0.009034 | 1 | 0.038159 | 0.012015 | TIMP1 |
| Aspc1 | 10 | 466 | 6.134969325 | 2.644920366 | 0.009917 | 1 | 0.041341 | 0.013018 | DRG1,TIMP1,CCT4,UGDH,ANP32E,SFN,NME1,GNB2L1,YWHAB,SEPT2 |
| Langerhans cells | 2 | 17 | 1.226993865 | 19.5627489 | 0.010093 | 1 | 0.041536 | 0.013079 | LTC4S,CCR10 |
| Breast | 72 | 6357 | 44.17177914 | 1.265550318 | 0.01105 | 1 | 0.044901 | 0.014138 | CRYM,KCNMB3,EFCAB2,HOXA5,ADRB2,STAT6,DRG1,CCNB1,COX4I1,ARPC3,PROSC,FST,CALCOCO2,ZHX3,DNAJB5,SLC39A9,MAPK3,MRPS7,DOK1,PRPSAP2,RHOA,PPP2R4,STAU2,GULP1,DLX5,CCT4,CDC34,HSPA2,MIPOL1,GALT,PRKRA,TRIM68,CAMK4,IMPDH2,CLGN,SFN,TEAD3,RAB11A,FAM63A,PCGF3,CKAP2,PDLIM3,MARCKSL1,C14orf119,CCR10,CDH26,RPS6KA2,NME1,RAP1GDS1,GNB2L1,ABCF3,NAP1L1,ANXA11,TARDBP,PPP3R1,ZMYM3,SNX9,SIGLEC5,LHX1,YWHAB,ANXA6,NOL3,SRPK2,USP15,MLX,FMN1,PRKAR2B,ACVR2B,COASY,ZNF655,SEPT2,RTN4 |
| T-lymphocytes | 2 | 18 | 1.226993865 | 18.5465022 | 0.011288 | 1 | 0.045292 | 0.014261 | GH1,ANXA6 |
| HPDE6 | 9 | 409 | 5.521472393 | 2.744635422 | 0.012168 | 1 | 0.048222 | 0.015184 | DTD1,TIMP1,PPP2R4,SFN,NME1,GNB2L1,FABP5,YWHAB,SEPT2 |
| Pancreatic cancer | 89 | 8226 | 54.60122699 | 1.205041225 | 0.012679 | 1 | 0.049633 | 0.015464 | CRYM,KCNMB3,EFCAB2,HOXA5,ADRB2,STAT6,CARHSP1,PSMD6,DRG1,CCNB1,COX4I1,ARPC3,PROSC,FST,RAB3B,CALCOCO2,ZHX3,CORO1A,DNAJB5,SLC39A9,MAPK3,MRPS7,DOK1,PRPSAP2,TIMP1,HLA-DRB5,RHOA,PPP2R4,EPS8L1,STAU2,GULP1,DLX5,CCT4,CDC34,SULT1E1,HSPA2,MIPOL1,GALT,PRKRA,TRIM68,CAMK4,UGDH,IMPDH2,ANP32E,UBE2V2,CLGN,SFN,P2RX7,TEAD3,RAB11A,SNX1,FAM63A,PCGF3,CKAP2,PDLIM3,MARCKSL1,C14orf119,CCR10,CDH26,RPS6KA2,NME1,RAP1GDS1,GNB2L1,ABCF3,HLA-DRB3,NAP1L1,ANXA11,TARDBP,PPP3R1,ZMYM3,SNX9,SIGLEC5,LHX1,SRI,YWHAB,ANXA6,TPD52L2,NOL3,SRPK2,USP15,MLX,FMN1,PRKAR2B,ACVR2B,COASY,ZNF655,SEPT2,RTN4,GBE1 |
| J82 | 6 | 212 | 3.680981595 | 3.734722646 | 0.012699 | 1 | 0.049112 | 0.015464 | ARPC3,NME1,FABP5,YWHAB,ANXA6,GBE1 |

**Supplementary Table 7.8: Transcription factor**

| Analysis:Transcription factor |  |  |  |  |  |  |  |  |  |
| --- | --- | --- | --- | --- | --- | --- | --- | --- | --- |
| Name of data set: MG vs HC |  |  |  |  |  |  |  |  |  |
| Number of gene in data set: 165 |  |  |  |  |  |  |  |  |  |
| Number of gene mapped to Transcription factor : 141 |  |  |  |  |  |  |  |  |  |
|  |  |  |  |  |  |  |  |  |  |
| Transcription factor | No. of genes  in the data set | No. of genes in the background data set | Percentage of genes | Fold Enrichment | Uncorrected  p-value  (Hypergeometric test) | Corrected  p-value  (Bonferroni method) | Corrected  p-value  (BH method) | Storey and Tibshirani method  q-value | Genes mapped from  input data set |

**Supplementary Table 7.9: Clinical phenotypes**

| Analysis:Clinical phenotypes |  |  |  |  |  |  |  |  |  |
| --- | --- | --- | --- | --- | --- | --- | --- | --- | --- |
| Name of data set: MG vs HC |  |  |  |  |  |  |  |  |  |
| Number of gene in data set: 165 |  |  |  |  |  |  |  |  |  |
| Number of gene mapped to Clinical phenotypes : 8 |  |  |  |  |  |  |  |  |  |
|  |  |  |  |  |  |  |  |  |  |
| Clinical phenotypes | No. of genes  in the data set | No. of genes in the background data set | Percentage of genes | Fold Enrichment | Uncorrected  p-value  (Hypergeometric test) | Corrected  p-value  (Bonferroni method) | Corrected  p-value  (BH method) | Storey and Tibshirani method  q-value | Genes mapped from  input data set |
| Decreased or absent glycogen branching enzyme activity | 1 | 1 | 12.5 | 196.4594595 | 0.004405 | 0.863436 | 0.047969 | 0.001653 | GBE1 |
| Polyglucosan bodies (round intracellular inclusions) found in neuronal and astrocytic processes | 1 | 1 | 12.5 | 196.4594595 | 0.004405 | 0.863436 | 0.045444 | 0.001653 | GBE1 |
| Decreased liver function, progressive | 1 | 1 | 12.5 | 196.4594595 | 0.004405 | 0.863436 | 0.043172 | 0.001653 | GALT |
| Polydactyly, preaxial or postaxial | 1 | 1 | 12.5 | 196.4594595 | 0.004405 | 0.863436 | 0.041116 | 0.001653 | MIPOL1 |
| Fibular duplication | 1 | 1 | 12.5 | 196.4594595 | 0.004405 | 0.863436 | 0.039247 | 0.001653 | MIPOL1 |
| Limb malformations are variable | 1 | 1 | 12.5 | 196.4594595 | 0.004405 | 0.863436 | 0.037541 | 0.001653 | MIPOL1 |
| LAURIN-SANDROW SYNDROME | 1 | 1 | 12.5 | 196.4594595 | 0.004405 | 0.863436 | 0.035977 | 0.001653 | MIPOL1 |
| KOWARSKI SYNDROME | 1 | 1 | 12.5 | 196.4594595 | 0.004405 | 0.863436 | 0.034537 | 0.001653 | GH1 |
| Micturition difficulties | 1 | 1 | 12.5 | 196.4594595 | 0.004405 | 0.863436 | 0.033209 | 0.001653 | GBE1 |
| In untreated patients - elevated blood galactose urine reducing substances (galactosuria), hyperchloremic metabolic acidosis, aminoaciduria, elevated liver enzymes, albuminuria | 1 | 1 | 12.5 | 196.4594595 | 0.004405 | 0.863436 | 0.031979 | 0.001653 | GALT |
| Insulin responses to glucose and to arginine usually greater than normal | 1 | 1 | 12.5 | 196.4594595 | 0.004405 | 0.863436 | 0.030837 | 0.001653 | GH1 |
| White matter abnormalities seen on MRI | 1 | 1 | 12.5 | 196.4594595 | 0.004405 | 0.863436 | 0.029774 | 0.001653 | GBE1 |
| High incidence of E. coli sepsis in untreated neonates | 1 | 1 | 12.5 | 196.4594595 | 0.004405 | 0.863436 | 0.028781 | 0.001653 | GALT |
| Neuromuscular forms can present as perinate, infant, child, or adult | 1 | 1 | 12.5 | 196.4594595 | 0.004405 | 0.863436 | 0.027853 | 0.001653 | GBE1 |
| Nonprogressive hepatic form is less frequent | 1 | 1 | 12.5 | 196.4594595 | 0.004405 | 0.863436 | 0.026982 | 0.001653 | GBE1 |
| Normal immunoreactive growth hormone after stimulation | 1 | 1 | 12.5 | 196.4594595 | 0.004405 | 0.863436 | 0.026165 | 0.001653 | GH1 |
| Normal lower limbs (in some patients) | 1 | 1 | 12.5 | 196.4594595 | 0.004405 | 0.863436 | 0.025395 | 0.001653 | DLX5 |
| Onset after age 40 years | 1 | 1 | 12.5 | 196.4594595 | 0.004405 | 0.863436 | 0.02467 | 0.001653 | GBE1 |
| Ovarian failure due to hypergonadotropic hypogonadism | 1 | 1 | 12.5 | 196.4594595 | 0.004405 | 0.863436 | 0.023984 | 0.001653 | GALT |
| Grooved columella | 1 | 1 | 12.5 | 196.4594595 | 0.004405 | 0.863436 | 0.023336 | 0.001653 | MIPOL1 |
| GALACTOSEMIA | 1 | 1 | 12.5 | 196.4594595 | 0.004405 | 0.863436 | 0.022722 | 0.001653 | GALT |
| Galactose-1-phosphate uridyltransferase deficiency | 1 | 1 | 12.5 | 196.4594595 | 0.004405 | 0.863436 | 0.022139 | 0.001653 | GALT |
| Fetal hydrops (in perinatal or congenital neuromuscular forms) | 1 | 1 | 12.5 | 196.4594595 | 0.004405 | 0.863436 | 0.021586 | 0.001653 | GBE1 |
| Decreased fetal movement (in perinatal or congenital neuromuscular forms) | 1 | 1 | 12.5 | 196.4594595 | 0.004405 | 0.863436 | 0.021059 | 0.001653 | GBE1 |
| Pyramidal tetraparesis | 1 | 1 | 12.5 | 196.4594595 | 0.004405 | 0.863436 | 0.020558 | 0.001653 | GBE1 |
| Cup-shaped hands | 1 | 1 | 12.5 | 196.4594595 | 0.004405 | 0.863436 | 0.02008 | 0.001653 | MIPOL1 |
| Absent tibia | 1 | 1 | 12.5 | 196.4594595 | 0.004405 | 0.863436 | 0.019624 | 0.001653 | MIPOL1 |
| Allelic disorder to adult polyglucosan body disease ( | 1 | 1 | 12.5 | 196.4594595 | 0.004405 | 0.863436 | 0.019187 | 0.001653 | GBE1 |
| Allelic disorder to type IV glycogen storage disease ( | 1 | 1 | 12.5 | 196.4594595 | 0.004405 | 0.863436 | 0.01877 | 0.001653 | GBE1 |
| Amylo(1,4 - 1,6) transglucosidase deficiency (brancher enzyme) | 1 | 1 | 12.5 | 196.4594595 | 0.004405 | 0.863436 | 0.018371 | 0.001653 | GBE1 |
| Antibodies to administered growth hormone | 1 | 1 | 12.5 | 196.4594595 | 0.004405 | 0.863436 | 0.017988 | 0.001653 | GH1 |
| Arthrogryposis multiplex (in perinatal or congenital neuromuscular forms) | 1 | 1 | 12.5 | 196.4594595 | 0.004405 | 0.863436 | 0.017621 | 0.001653 | GBE1 |
| Cylindrical nails (in some patients) | 1 | 1 | 12.5 | 196.4594595 | 0.004405 | 0.863436 | 0.017269 | 0.001653 | DLX5 |
| Ulnar duplication | 1 | 1 | 12.5 | 196.4594595 | 0.004405 | 0.863436 | 0.01693 | 0.001653 | MIPOL1 |
| Asymmetrical severely deformed feet (in some patients) | 1 | 1 | 12.5 | 196.4594595 | 0.004405 | 0.863436 | 0.016605 | 0.001653 | DLX5 |
| Asymmetric short and severely deformed legs (in some patients) | 1 | 1 | 12.5 | 196.4594595 | 0.004405 | 0.863436 | 0.016291 | 0.001653 | DLX5 |
| Autopodial duplication, symmetrical | 1 | 1 | 12.5 | 196.4594595 | 0.004405 | 0.863436 | 0.01599 | 0.001653 | MIPOL1 |
| Tapered fingers (in some patients) | 1 | 1 | 12.5 | 196.4594595 | 0.004405 | 0.863436 | 0.015699 | 0.001653 | DLX5 |
| Liver biopsy shows diffuse interstitial fibrosis | 1 | 1 | 12.5 | 196.4594595 | 0.004405 | 0.863436 | 0.015419 | 0.001653 | GBE1 |
| Broad tissue deposition of amylopectin-like material | 1 | 1 | 12.5 | 196.4594595 | 0.004405 | 0.863436 | 0.015148 | 0.001653 | GBE1 |
| Cirrhosis if untreated | 1 | 1 | 12.5 | 196.4594595 | 0.004405 | 0.863436 | 0.014887 | 0.001653 | GALT |
| Short stature, severe (in some patients) | 1 | 1 | 12.5 | 196.4594595 | 0.004405 | 0.863436 | 0.014635 | 0.001653 | DLX5 |
| Classic hepatic form begins in first months of life with hepatic failure and death by age 5 years | 1 | 1 | 12.5 | 196.4594595 | 0.004405 | 0.863436 | 0.014391 | 0.001653 | GBE1 |
| Speech abnormality if untreated | 1 | 1 | 12.5 | 196.4594595 | 0.004405 | 0.863436 | 0.014155 | 0.001653 | GALT |
| Cleft nares, bilateral | 1 | 1 | 12.5 | 196.4594595 | 0.004405 | 0.863436 | 0.013926 | 0.001653 | MIPOL1 |
| Cardiomyopathy (in a subset of patients) | 1 | 1 | 12.5 | 196.4594595 | 0.004405 | 0.863436 | 0.013705 | 0.001653 | GBE1 |
| N-myc oncogene ( | 1 | 2 | 12.5 | 136.010395 | 0.008794 | 1 | 0.02693 | 0.002143 | NME1 |
| Usually sporadic, but 1-2% of cases are familial | 1 | 2 | 12.5 | 136.010395 | 0.008794 | 1 | 0.026516 | 0.002143 | NME1 |
| Highly variable clinical phenotype | 1 | 2 | 12.5 | 136.010395 | 0.008794 | 1 | 0.026114 | 0.002143 | NME1 |
| Horner's syndrome | 1 | 2 | 12.5 | 136.010395 | 0.008794 | 1 | 0.025725 | 0.002143 | NME1 |
| Hypertension (compression of renal arteries) | 1 | 2 | 12.5 | 136.010395 | 0.008794 | 1 | 0.025346 | 0.002143 | NME1 |
| NEUROBLASTOMA, SUSCEPTIBILITY TO | 1 | 2 | 12.5 | 136.010395 | 0.008794 | 1 | 0.024979 | 0.002143 | NME1 |
| Immunol | 1 | 2 | 12.5 | 136.010395 | 0.008794 | 1 | 0.024622 | 0.002143 | GH1 |
| Neuroblastoma, arises anywhere along the sympathetic chain (including intracranially) | 1 | 2 | 12.5 | 136.010395 | 0.008794 | 1 | 0.024275 | 0.002143 | NME1 |
| Increased urinary catecholamines | 1 | 2 | 12.5 | 136.010395 | 0.008794 | 1 | 0.023938 | 0.002143 | NME1 |
| Increased urinary dopamine | 1 | 2 | 12.5 | 136.010395 | 0.008794 | 1 | 0.02361 | 0.002143 | NME1 |
| Increased urinary homovanillic acid (HVA) | 1 | 2 | 12.5 | 136.010395 | 0.008794 | 1 | 0.023291 | 0.002143 | NME1 |
| Increased urinary vanillylmandelic acid (VMA) | 1 | 2 | 12.5 | 136.010395 | 0.008794 | 1 | 0.022981 | 0.002143 | NME1 |
| Adrenal glands are most common site | 1 | 2 | 12.5 | 136.010395 | 0.008794 | 1 | 0.022678 | 0.002143 | NME1 |
| Absent radius | 1 | 2 | 12.5 | 136.010395 | 0.008794 | 1 | 0.022384 | 0.002143 | MIPOL1 |
| Isolated growth hormone deficiency | 1 | 2 | 12.5 | 136.010395 | 0.008794 | 1 | 0.022097 | 0.002143 | GH1 |
| Absent patella | 1 | 2 | 12.5 | 136.010395 | 0.008794 | 1 | 0.021817 | 0.002143 | MIPOL1 |
| Weight loss (with disseminated disease) | 1 | 2 | 12.5 | 136.010395 | 0.008794 | 1 | 0.021544 | 0.002143 | NME1 |
| Abdominal mass with calcifications on radiology | 1 | 2 | 12.5 | 136.010395 | 0.008794 | 1 | 0.021278 | 0.002143 | NME1 |
| Mediastinal mass with calcifications on radiology | 1 | 2 | 12.5 | 136.010395 | 0.008794 | 1 | 0.021019 | 0.002143 | NME1 |
| Anemia (with bone marrow involvement) | 1 | 2 | 12.5 | 136.010395 | 0.008794 | 1 | 0.020766 | 0.002143 | NME1 |
| Bone pain (with metastatic disease) | 1 | 2 | 12.5 | 136.010395 | 0.008794 | 1 | 0.020518 | 0.002143 | NME1 |
| Paraneoplastic syndromes | 1 | 2 | 12.5 | 136.010395 | 0.008794 | 1 | 0.020277 | 0.002143 | NME1 |
| Pituitary dwarfism | 1 | 2 | 12.5 | 136.010395 | 0.008794 | 1 | 0.020041 | 0.002143 | GH1 |
| Opsoclonus | 1 | 2 | 12.5 | 136.010395 | 0.008794 | 1 | 0.019811 | 0.002143 | NME1 |
| Bluish skin nodules | 1 | 2 | 12.5 | 136.010395 | 0.008794 | 1 | 0.019586 | 0.002143 | NME1 |
| Mean age of onset 14-24 months | 1 | 2 | 12.5 | 136.010395 | 0.008794 | 1 | 0.019366 | 0.002143 | NME1 |
| Palpable abdominal mass | 1 | 2 | 12.5 | 136.010395 | 0.008794 | 1 | 0.01915 | 0.002143 | NME1 |
| Periorbital ecchymoses (soft tissue involvement) | 1 | 2 | 12.5 | 136.010395 | 0.008794 | 1 | 0.01894 | 0.002143 | NME1 |
| Split hand | 1 | 2 | 12.5 | 136.010395 | 0.008794 | 1 | 0.018734 | 0.002143 | DLX5 |
| Spontaneous tumor regression may occur | 1 | 2 | 12.5 | 136.010395 | 0.008794 | 1 | 0.018533 | 0.002143 | NME1 |
| Susceptibility conferred by mutation in the kinesin family member 1B gene (KIF1B, | 1 | 2 | 12.5 | 136.010395 | 0.008794 | 1 | 0.018336 | 0.002143 | NME1 |
| Tumor may secrete vasoactive intestinal peptide (VIP) | 1 | 2 | 12.5 | 136.010395 | 0.008794 | 1 | 0.018143 | 0.002143 | NME1 |
| Symptoms vary according to location of tumor | 1 | 2 | 12.5 | 136.010395 | 0.008794 | 1 | 0.017954 | 0.002143 | NME1 |
| Diarrhea (due to vasoactive intestinal peptide) | 1 | 2 | 12.5 | 136.010395 | 0.008794 | 1 | 0.017768 | 0.002143 | NME1 |
| Esophageal varices | 1 | 3 | 12.5 | 104.0079491 | 0.013165 | 1 | 0.02633 | 0.003022 | GBE1 |
| Split foot | 1 | 3 | 12.5 | 104.0079491 | 0.013165 | 1 | 0.026064 | 0.003022 | DLX5 |
| Normal serum creatine kinase | 1 | 3 | 12.5 | 104.0079491 | 0.013165 | 1 | 0.025803 | 0.003022 | GBE1 |
| Short, broad feet | 1 | 3 | 12.5 | 104.0079491 | 0.013165 | 1 | 0.025548 | 0.003022 | MIPOL1 |
| Persistence of fetal hemoglobin (5-30% HbF) | 1 | 3 | 12.5 | 104.0079491 | 0.013165 | 1 | 0.025297 | 0.003022 | HBG2 |
| Hepatosplenomegaly | 1 | 3 | 12.5 | 104.0079491 | 0.013165 | 1 | 0.025052 | 0.003022 | GBE1 |
| Neurogenic bladder | 1 | 4 | 12.5 | 84.1969112 | 0.017519 | 1 | 0.033017 | 0.003983 | GBE1 |
| Mental retardation if untreated | 1 | 5 | 12.5 | 70.72540541 | 0.021857 | 1 | 0.0408 | 0.004922 | GALT |
| Metabolic | 2 | 55 | 25 | 11.35099099 | 0.022436 | 1 | 0.041485 | 0.005004 | ADRB2,GH1 |
| Upper motor neuron signs | 1 | 6 | 12.5 | 60.97017707 | 0.026178 | 1 | 0.047952 | 0.005678 | GBE1 |
| Portal hypertension | 1 | 6 | 12.5 | 60.97017707 | 0.026178 | 1 | 0.047508 | 0.005678 | GBE1 |
| Ectrodactyly | 1 | 6 | 12.5 | 60.97017707 | 0.026178 | 1 | 0.047072 | 0.005678 | DLX5 |
