# Supplementary material for: Evaluation of autoantibody signatures in meningioma patients using human proteome arrays
Source: Oncotarget. 2017 Apr 10;8(35):58443–56. doi: 10.18632/oncotarget.16997 (PMC5601665; doi:10.18632/oncotarget.16997)
Supplement: Supplementary file 15 [file oncotarget-08-58443-s015.docx]

**Supplementary Table 8: GO clustering using Revigo**

**Supplementary Table 8.1: Input list of proteins for generating GO terms (significant proteins in MG1 vs HC with absolute logFC>0.5, p-value<0.05)**

|  |
| --- |
| CRYM |
| KCNMB3 |
| HOXA5 |
| EFCAB2 |
| DOHH |
| STAT6 |
| CD84 |
| ZHX3 |
| COQ6 |
| CCNB1 |
| ADRB2 |
| DRG1 |
| PROSC |
| DNAJB5 |
| GYPE |
| OIP5 |
| SRPX2 |
| AFP |
| STK33 |
| FHL2 |
| NFE2 |
| RAB3B |
| STAU2 |
| MRPS7 |
| DAZAP2 |
| CALCOCO2 |
| FST |
| NRAS |
| TYROBP |
| RPL10 |
| KLK1 |
| C2orf44 |
| KRR1 |
| CSRP1 |
| ERAL1 |
| OGFOD2 |
| BHMT2 |
| GPSM3 |
| APOE |
| HLA-DRB5 |
| COX4I1 |
| HPCAL1 |
| CD47 |
| METTL8 |
| UPP2 |
| AEBP2 |
| TSPAN17 |
| HSPA2 |
| GULP1 |
| TM4SF1 |
| GALT |
| SP110 |
| RWDD3 |
| ELOVL1 |
| EDN3 |
| LYPD1 |
| LRRC20 |
| CARHSP1 |
| NAPSA |
| CKS2 |
| FUBP3 |
| IGLL1 |
| GSTP1 |
| CAMK2N1 |
| RPS6KA2 |
| CYBB |
| CORO1A |
| FUBP1 |
| ATAD2 |
| FAM63A |
| CAPRIN2 |
| FBXO31 |
| FAM122C |
| RPS7 |
| TIMP1 |
| STATH |
| OLR1 |
| NUDT22 |
| PPP2R4 |
| SLC39A9 |
| ADRBK1 |
| QDPR |
| IFIT3 |
| ROPN1L |
| RHOA |
| CD96 |
| LAYN |
| Dlx5 |
| GADD45A |
| RAP1GDS1 |
| ATG3 |
| TIPIN |
| HPX |
| SGK2 |
| NECAP2 |
| HLA-DOB |
| SPAG16 |
| CHD4 |
| ST6GALNAC6 |
| DOK1 |
| MRPS25 |
| PSMD14 |
| SLC5A6 |
| IMPDH2 |
| GH2 |
| PANK1 |
| GLYAT |
| XPR1 |
| DHX9 |
| GNB2L1 |
| MRTO4 |
| CDO1 |
| BCAS2 |
| FAM58A |
| CYB5R1 |
| FGF7 |
| CDC34 |
| CLDN4 |
| ARL2BP |
| SYNPR |
| RAB39B |
| LCAT |
| VSNL1 |
| EPS8L1 |
| PTN |
| RRAGB |
| CTNNAL1 |
| FBXL18 |
| SFN |
| ANP32E |
| FN3K |
| SEPT1 |
| NHEJ1 |
| NME1 |
| STARD10 |
| NMRAL1 |
| ADD1 |
| ACTL6B |
| CAMK4 |
| RPS15 |
| TEX264 |
| DCK |
| ZNF641 |
| DHFR |
| FBXO3 |
| TMEM185A |
| C6orf141 |
| HN1 |
| ANAPC11 |
| DDX58 |
| ARPC3 |
| SPP1 |
| CLGN |
| RAB24 |
| MTL5 |
| SULT1E1 |
| SH2D1B |
| KLF11 |
| HLA-DRB3 |
| NOSIP |
| EIF4EBP3 |
| PTS |
| RBKS |
| CCNH |
| AAK1 |
| ARHGDIG |
| APEX1 |
| ZNF761 |
| CCT4 |
| SERTAD3 |
| CXXC5 |
| FNIP1 |
| ZNF587 |
| CCR10 |
| MRPL53 |
| ABLIM1 |
| ARIH2 |
| BAG5 |
| MAPK3 |
| TIRAP |
| CDCP1 |
| MYOT |
| ARF5 |
| YWHAB |
| DBNL |
| TCOF1 |
| GGT6 |
| RPL14 |
| CALN1 |
| C1QC |
| C1orf21 |
| RNF32 |
| TTC1 |
| USP15 |
| MORC4 |
| UGDH |
| ASF1A |
| MIPOL1 |
| ENOPH1 |
| PTPN11 |
| ZFYVE19 |
| PRKRA |
| SMYD3 |
| MCM5 |
| C14orf119 |
| DIABLO |
| QTRT1 |
| PDLIM3 |
| RBBP7 |
| PI16 |
| CKAP2 |
| GABRA5 |
| NAGK |
| PRH2 |
| CPNE4 |
| MEIS2 |
| MCTS1 |
| DERL1 |
| HIGD2A |
| RPL22 |
| SRPK2 |
| FAM81A |
| HSPB8 |
| ATP6V1E2 |
| C14orf80 |
| HBG2 |
| C11orf49 |
| SERBP1 |
| SPATA7 |
| TK1 |
| MARCKSL1 |
| SPC25 |
| CYP2C8 |
| STUB1 |
| PRPSAP2 |
| RNF126 |
| RAB8B |
| MATK |
| C9 |
| GTF2I |
| BCL7C |
| PYCR1 |
| CDH26 |
| NDUFA8 |
| DAPP1 |
| TOM1 |
| FBXL3 |
| PCGF3 |
| PLCB2 |
| CHRAC1 |
| PAK6 |
| ANXA6 |
| RPS13 |
| USP14 |
| CLP1 |
| TEAD3 |
| COASY |
| TMEM106A |
| RECQL5 |
| ANXA11 |
| PDS5B |
| RNF11 |
| ABLIM3 |
| MAPK1 |
| TXNRD1 |
| POLE3 |
| CCL5 |
| SRI |
| MLX |
| TARDBP |
| HCRTR1 |
| RASGEF1A |
| MOXD1 |
| CKM |
| F8 |
| EDNRA |
| SLA |
| TRIML1 |
| CETN3 |
| PSMD10 |
| TMEM185B |
| LRFN1 |
| LEPROTL1 |
| ALDH1A1 |
| EPB41L3 |
| SAR1B |
| SELENBP1 |
| MMP7 |
| ABCF3 |
| CCDC102B |
| RAB11A |
| RUVBL1 |
| TPD52L2 |
| SNX1 |
| PAK4 |
| STK25 |
| DUPD1 |
| FGFR2 |
| SLC25A10 |
| S100A7A |
| FGA |
| LEMD1 |
| DDC |
| FMN1 |
| FAM104B |
| PECAM1 |
| TAF7 |
| PAF1 |
| HSPBAP1 |
| SNX9 |
| FBP1 |
| ZNF655 |
| LST1 |
| SET |
| PPP3R1 |
| PRKAR2B |
| RPS6 |
| ATP6V1C2 |
| MYLK |
| VCP |
| RAC1 |
| COG3 |
| MBIP |
| HSPBP1 |
| PLEK |
| AKT3 |
| SRP54 |
| NUDT18 |
| GKAP1 |
| CHORDC1 |
| PHKG2 |
| HIATL1 |
| GFM2 |
| C1orf87 |
| Lhx1 |
| ZRANB2 |
| GBE1 |
| EYA1 |
| FABP5 |
| SSPN |
| ZMYM3 |
| NPM1 |
| CORO2B |
| NRBF2 |
| KRCC1 |
| CIAO1 |
| PGM2 |
| ARHGAP29 |
| GOLT1B |
| KLHL14 |
| CYGB |
| RTN4 |
| OBFC1 |
| PDXK |
| RNPEP |
| FAM9C |
| PLEKHG2 |
| ANXA13 |
| NFKBIA |
| DGUOK |
| ALDH9A1 |
| ACVR2B |
| C8orf37 |
| UBE2O |
| PEX19 |
| IGHG4 |
| WDR42A |
| RY1 |
| HCG3 |
| C20orf112 |
| HDAC7A |
| C17orf57 |
| CHP |
| LOC389833 |
| PRIM2A |
| KARCA1 |
| SURB7 |
| P15RS |
| C20orf42 |
| THRAP6 |
| HRASLS3 |
| C12orf11 |
| IGKC |
| MRS2L |
| APITD1 |
| PBEF1 |
| C8orf43 |
| FAM40B |
| XTP3TPA |
| LOC387758 |
| TMEM166 |
| C11orf67 |
| LOC196541 |
| UBADC1 |
| IGL@ |
| C14orf122 |
| LOC285382 |
| FAM119B |
| C1orf76 |
| C13orf3 |
| C9orf123 |
| LOC652968 |
| PCID1 |
| C14orf126 |
| IGHG1 |
| C20orf28 |
| VIL2 |
| M6PRBP1 |
| ZNF643 |
| C21orf33 |
| MDP-1 |
| C14orf149 |
| LOC158381 |
| NY-REN-7 |
| C13orf15 |
| KBTBD5 |
| MTERFD2 |
| IGHD |
| KIAA1576 |
| CXorf9 |
| EIF3S3 |
| FLJ22222 |
| IGHG1 |
| JUB |
| MGC2408 |
| FAM13A1 |
| SFRS15 |
| SPN |
| LOC729447 |
| SAS10 |
| IHPK1 |
| SGK |
| C20orf43 |
| IGHG1 |
| C6orf115 |
| RP11-56A21.1 |
| TXNL2 |
| LOC554174 |
| PIP5K2C |
| ASAP |
| LOC374395 |

**Supplementary Table 8.2:** **GO output for HC vs MG1 from StringDB**

|  | |  |  |
| --- | --- | --- | --- |
| Biological Process (GO) |  |  |  |
| **pathway ID** | **pathway description** | **count in network** | **false discovery rate** |
| GO:0043933 | macromolecular complex subunit organization | 75 | 0.000879 |
| GO:0044710 | single-organism metabolic process | 118 | 0.00506 |
| GO:0071822 | protein complex subunit organization | 54 | 0.00506 |
| GO:0006793 | phosphorus metabolic process | 64 | 0.00539 |
| GO:0006796 | phosphate-containing compound metabolic process | 62 | 0.00683 |
| GO:0043094 | cellular metabolic compound salvage | 7 | 0.00683 |
| GO:1901566 | organonitrogen compound biosynthetic process | 40 | 0.00683 |
| GO:0000278 | mitotic cell cycle | 36 | 0.007 |
| GO:0007265 | Ras protein signal transduction | 17 | 0.007 |
| GO:0031401 | positive regulation of protein modification process | 42 | 0.007 |
| GO:1904029 | regulation of cyclin-dependent protein kinase activity | 13 | 0.007 |
| GO:0048522 | positive regulation of cellular process | 118 | 0.00803 |
| GO:1903047 | mitotic cell cycle process | 33 | 0.00922 |
| GO:0008152 | metabolic process | 218 | 0.00997 |
| GO:0034613 | cellular protein localization | 43 | 0.0116 |
| GO:0044238 | primary metabolic process | 198 | 0.0116 |
| GO:0044403 | symbiosis, encompassing mutualism through parasitism | 34 | 0.0116 |
| GO:0050790 | regulation of catalytic activity | 69 | 0.0129 |
| GO:0065009 | regulation of molecular function | 80 | 0.0129 |
| GO:0071704 | organic substance metabolic process | 201 | 0.0129 |
| GO:0044093 | positive regulation of molecular function | 56 | 0.0173 |
| GO:0045859 | regulation of protein kinase activity | 30 | 0.0173 |
| GO:0051128 | regulation of cellular component organization | 67 | 0.0173 |
| GO:0009157 | deoxyribonucleoside monophosphate biosynthetic process | 3 | 0.0181 |
| GO:0065003 | macromolecular complex assembly | 43 | 0.0181 |
| GO:0001934 | positive regulation of protein phosphorylation | 33 | 0.019 |
| GO:0048518 | positive regulation of biological process | 128 | 0.019 |
| GO:0031399 | regulation of protein modification process | 53 | 0.0202 |
| GO:0007264 | small GTPase mediated signal transduction | 29 | 0.0223 |
| GO:0051338 | regulation of transferase activity | 35 | 0.0223 |
| GO:0006996 | organelle organization | 82 | 0.0244 |
| GO:0010608 | posttranscriptional regulation of gene expression | 19 | 0.025 |
| GO:0044237 | cellular metabolic process | 192 | 0.0252 |
| GO:0000079 | regulation of cyclin-dependent protein serine/threonine kinase activity | 9 | 0.0285 |
| GO:0007049 | cell cycle | 46 | 0.0285 |
| GO:0032270 | positive regulation of cellular protein metabolic process | 45 | 0.0285 |
| GO:0038179 | neurotrophin signaling pathway | 17 | 0.0285 |
| GO:0071218 | cellular response to misfolded protein | 4 | 0.0285 |
| GO:0019538 | protein metabolic process | 105 | 0.0307 |
| GO:0048583 | regulation of response to stimulus | 91 | 0.0338 |
| GO:0045937 | positive regulation of phosphate metabolic process | 36 | 0.0342 |
| GO:0090083 | regulation of inclusion body assembly | 4 | 0.0342 |
| GO:0006897 | endocytosis | 23 | 0.0401 |
| GO:0035556 | intracellular signal transduction | 57 | 0.0401 |
| GO:0045860 | positive regulation of protein kinase activity | 21 | 0.0401 |
| GO:0051347 | positive regulation of transferase activity | 25 | 0.0401 |
| GO:0001932 | regulation of protein phosphorylation | 41 | 0.0455 |
| GO:0016192 | vesicle-mediated transport | 40 | 0.0471 |
| GO:0044764 | multi-organism cellular process | 29 | 0.0481 |
| GO:0042327 | positive regulation of phosphorylation | 32 | 0.0494 |

**Supplementary Table 8.3:** **GO scatter-plot from REVIGO for HCvsMG1**

|  |  |  |  |  |  |  |  |  |  |
| --- | --- | --- | --- | --- | --- | --- | --- | --- | --- |
| **term_ID** | **description** | **frequency** | **plot_X** | **plot_Y** | **plot_size** | **uniqueness** | **dispensability** | **representative** | **eliminated** |
| GO:0008152 | metabolic process | 82.18% | 0.908 | -2.522 | 7.612 | 0.993 | 0 | 8152 | 0 |
| GO:0016192 | vesicle-mediated transport | 0.38% | 3.012 | 5.148 | 5.277 | 0.938 | 0 | 16192 | 0 |
| GO:0043094 | cellular metabolic compound salvage | 0.37% | -0.556 | 6.152 | 5.263 | 0.905 | 0 | 43094 | 0 |
| GO:0044403 | symbiosis, encompassing mutualism through parasitism | 1.78% | 2.306 | -6.942 | 5.948 | 0.924 | 0 | 44403 | 0 |
| GO:0044764 | multi-organism cellular process | 2.06% | null | null | 6.01 | 0.894 | 0.881 | 44403 | 1 |
| GO:0065009 | regulation of molecular function | 0.84% | -7.236 | -1.157 | 5.619 | 0.7 | 0 | 65009 | 0 |
| GO:0071822 | protein complex subunit organization | 0.87% | -3.19 | -7.416 | 5.635 | 0.832 | 0 | 71822 | 0 |
| GO:0065003 | macromolecular complex assembly | 0.68% | null | null | 5.527 | 0.829 | 0.923 | 71822 | 1 |
| GO:0071704 | organic substance metabolic process | 56.18% | -0.114 | -6.541 | 7.446 | 0.964 | 0.011 | 71704 | 0 |
| GO:0071218 | cellular response to misfolded protein | 0.00% | -6.374 | 4.89 | 2.734 | 0.875 | 0.017 | 71218 | 0 |
| GO:0019538 | protein metabolic process | 12.33% | -3.758 | 6.497 | 6.788 | 0.889 | 0.026 | 19538 | 0 |
| GO:0007049 | cell cycle | 1.41% | -1.345 | 3.4 | 5.846 | 0.905 | 0.029 | 7049 | 0 |
| GO:0044238 | primary metabolic process | 51.93% | 1.498 | -5.333 | 7.412 | 0.963 | 0.057 | 44238 | 0 |
| GO:0044237 | cellular metabolic process | 53.87% | 3.276 | -3.727 | 7.428 | 0.923 | 0.059 | 44237 | 0 |
| GO:1903047 | mitotic cell cycle process | 0.08% | 4.762 | -2.342 | 4.582 | 0.877 | 0.088 | 1903047 | 0 |
| GO:0044710 | single-organism metabolic process | 39.46% | -0.524 | 0.374 | 7.293 | 0.931 | 0.109 | 44710 | 0 |
| GO:0006793 | phosphorus metabolic process | 16.89% | 3.243 | 0.09 | 6.924 | 0.901 | 0.118 | 6793 | 0 |
| GO:0009157 | deoxyribonucleoside monophosphate biosynthetic process | 0.12% | 3.146 | 4.459 | 4.759 | 0.831 | 0.147 | 9157 | 0 |
| GO:1901566 | organonitrogen compound biosynthetic process | 9.30% | -0.618 | 6.844 | 6.665 | 0.915 | 0.208 | 1901566 | 0 |
| GO:0006897 | endocytosis | 0.20% | 2.135 | 3.291 | 4.998 | 0.939 | 0.245 | 6897 | 0 |
| GO:0034613 | cellular protein localization | 0.67% | 2.713 | 3.619 | 5.523 | 0.938 | 0.268 | 34613 | 0 |
| GO:0044093 | positive regulation of molecular function | 0.23% | -7.444 | -2.161 | 5.058 | 0.625 | 0.298 | 44093 | 0 |
| GO:0050790 | regulation of catalytic activity | 0.65% | null | null | 5.51 | 0.578 | 0.854 | 44093 | 1 |
| GO:0051338 | regulation of transferase activity | 0.16% | null | null | 4.891 | 0.607 | 0.757 | 44093 | 1 |
| GO:0048522 | positive regulation of cellular process | 0.43% | -8.204 | -1.462 | 5.329 | 0.593 | 0.316 | 48522 | 0 |
| GO:0001934 | positive regulation of protein phosphorylation | 0.04% | null | null | 4.287 | 0.493 | 0.923 | 48522 | 1 |
| GO:0045860 | positive regulation of protein kinase activity | 0.03% | null | null | 4.117 | 0.451 | 0.951 | 48522 | 1 |
| GO:0032270 | positive regulation of cellular protein metabolic process | 0.11% | null | null | 4.718 | 0.515 | 0.804 | 48522 | 1 |
| GO:0042327 | positive regulation of phosphorylation | 0.05% | null | null | 4.373 | 0.515 | 0.973 | 48522 | 1 |
| GO:0045937 | positive regulation of phosphate metabolic process | 0.05% | null | null | 4.419 | 0.522 | 0.992 | 48522 | 1 |
| GO:0031401 | positive regulation of protein modification process | 0.05% | null | null | 4.375 | 0.518 | 0.936 | 48522 | 1 |
| GO:0010608 | posttranscriptional regulation of gene expression | 0.54% | -7.36 | -0.335 | 5.427 | 0.659 | 0.323 | 10608 | 0 |
| GO:0048518 | positive regulation of biological process | 0.68% | -7.906 | -1.17 | 5.532 | 0.699 | 0.331 | 48518 | 0 |
| GO:0048583 | regulation of response to stimulus | 0.69% | -7.661 | 0.478 | 5.535 | 0.676 | 0.332 | 48583 | 0 |
| GO:0006796 | phosphate-containing compound metabolic process | 16.69% | 4.726 | 1.208 | 6.919 | 0.82 | 0.346 | 6796 | 0 |
| GO:0090083 | regulation of inclusion body assembly | 0.00% | -5.463 | -4.626 | 2.587 | 0.725 | 0.365 | 90083 | 0 |
| GO:0031399 | regulation of protein modification process | 0.18% | -6.362 | -0.381 | 4.96 | 0.572 | 0.366 | 31399 | 0 |
| GO:0001932 | regulation of protein phosphorylation | 0.17% | null | null | 4.916 | 0.498 | 0.778 | 31399 | 1 |
| GO:0045859 | regulation of protein kinase activity | 0.15% | null | null | 4.867 | 0.442 | 0.974 | 31399 | 1 |
| GO:0000079 | regulation of cyclin-dependent protein serine/threonine kinase activity | 0.02% | null | null | 4.08 | 0.478 | 0.854 | 31399 | 1 |
| GO:0007265 | Ras protein signal transduction | 0.10% | -7.292 | 1.378 | 4.703 | 0.67 | 0.373 | 7265 | 0 |
| GO:0038179 | neurotrophin signaling pathway | 0.00% | -6.93 | 2.323 | 3.04 | 0.719 | 0.555 | 38179 | 0 |
| GO:0000278 | mitotic cell cycle | 0.09% | 4.906 | -2.973 | 4.637 | 0.877 | 0.591 | 278 | 0 |
| GO:0035556 | intracellular signal transduction | 2.72% | -6.861 | 0.591 | 6.131 | 0.595 | 0.599 | 35556 | 0 |
| GO:0043933 | macromolecular complex subunit organization | 1.06% | -3.112 | -7.28 | 5.723 | 0.842 | 0.649 | 43933 | 0 |
| GO:0007264 | small GTPase mediated signal transduction | 0.23% | -7.426 | 1.07 | 5.067 | 0.654 | 0.651 | 7264 | 0 |
| GO:0006996 | organelle organization | 0.93% | -3.483 | -7.316 | 5.666 | 0.817 | 0.654 | 6996 | 0 |
| GO:0051128 | regulation of cellular component organization | 1.02% | -5.985 | -3.408 | 5.705 | 0.599 | 0.66 | 51128 | 0 |
| GO:0051347 | positive regulation of transferase activity | 0.03% | -6.987 | -2.211 | 4.168 | 0.623 | 0.668 | 51347 | 0 |

**Supplementary Table 8.4: Input list of proteins for generating GO terms (significant proteins in MG2 vs HC with absolute logFC>0.5, p-value<0.05)**

|  |
| --- |
| CRYM |
| KCNMB3 |
| EFCAB2 |
| ARPC3 |
| CARHSP1 |
| ADRB2 |
| HOXA5 |
| COX4I1 |
| CCDC28A |
| STAT6 |
| PAIP1 |
| PSMD6 |
| MAPK3 |
| DRG1 |
| GSG1 |
| PRPSAP2 |
| KRR1 |
| ZFYVE19 |
| CCNB1 |
| CORO1A |
| FST |
| MRPL13 |
| RNF11 |
| PROSC |
| EIF4EBP3 |
| OIP5 |
| PRKG1 |
| CDKN1B |
| DDI2 |
| DTD1 |
| SLC39A9 |
| LCN1 |
| RAB3B |
| DOK1 |
| HSD17B14 |
| UBE2V2 |
| BLOC1S2 |
| AIF1 |
| CALCOCO2 |
| SGCG |
| EPS8L1 |
| RBP1 |
| KLK1 |
| MIPOL1 |
| RNF25 |
| DLC1 |
| TRIM68 |
| CCT4 |
| HPCAL1 |
| RHOA |
| C1QTNF7 |
| PRKRA |
| FAIM |
| TIRAP |
| DOHH |
| SULT1E1 |
| PPP2R4 |
| CKS2 |
| GPSM3 |
| GYPE |
| TMEM185B |
| POLR3B |
| ABLIM1 |
| MYL2 |
| UGDH |
| OR10G3 |
| SNX1 |
| RAB11A |
| ATF6 |
| HBG1 |
| CTH |
| NMRAL1 |
| SAT1 |
| RPUSD2 |
| DYNLT3 |
| MTL5 |
| TEAD3 |
| HBG2 |
| Dlx5 |
| CDC34 |
| Nol3 |
| TIPIN |
| LRFN1 |
| P2RX7 |
| PAIP2 |
| ZHX3 |
| IGHG4 |
| C20orf112 |
| RY1 |
| C17orf57 |
| SURB7 |
| LOC389833 |
| HDAC7A |
| IGHG1 |
| NA |
| IL1F7 |
| LOC285382 |
| IGHG1 |
| JUB |
| HCG3 |
| FAM105B |
| LOC339803 |
| MRVI1-AS1 |
| KIAA0174 |

**Supplementary Table 8.5: GO output for HC vs MG2 from StringDB**

|  | |  |  |
| --- | --- | --- | --- |
| **pathway ID** | **pathway description** | **count in network** | **false discovery rate** |
| GO:0043933 | macromolecular complex subunit organization | 75 | 0.000879 |
| GO:0044710 | single-organism metabolic process | 118 | 0.00506 |
| GO:0071822 | protein complex subunit organization | 54 | 0.00506 |
| GO:0006793 | phosphorus metabolic process | 64 | 0.00539 |
| GO:0006796 | phosphate-containing compound metabolic process | 62 | 0.00683 |
| GO:0043094 | cellular metabolic compound salvage | 7 | 0.00683 |
| GO:1901566 | organonitrogen compound biosynthetic process | 40 | 0.00683 |
| GO:0000278 | mitotic cell cycle | 36 | 0.007 |
| GO:0007265 | Ras protein signal transduction | 17 | 0.007 |
| GO:0031401 | positive regulation of protein modification process | 42 | 0.007 |
| GO:1904029 | regulation of cyclin-dependent protein kinase activity | 13 | 0.007 |
| GO:0048522 | positive regulation of cellular process | 118 | 0.00803 |
| GO:1903047 | mitotic cell cycle process | 33 | 0.00922 |
| GO:0008152 | metabolic process | 218 | 0.00997 |
| GO:0034613 | cellular protein localization | 43 | 0.0116 |
| GO:0044238 | primary metabolic process | 198 | 0.0116 |
| GO:0044403 | symbiosis, encompassing mutualism through parasitism | 34 | 0.0116 |
| GO:0050790 | regulation of catalytic activity | 69 | 0.0129 |
| GO:0065009 | regulation of molecular function | 80 | 0.0129 |
| GO:0071704 | organic substance metabolic process | 201 | 0.0129 |
| GO:0044093 | positive regulation of molecular function | 56 | 0.0173 |
| GO:0045859 | regulation of protein kinase activity | 30 | 0.0173 |
| GO:0051128 | regulation of cellular component organization | 67 | 0.0173 |
| GO:0009157 | deoxyribonucleoside monophosphate biosynthetic process | 3 | 0.0181 |
| GO:0065003 | macromolecular complex assembly | 43 | 0.0181 |
| GO:0001934 | positive regulation of protein phosphorylation | 33 | 0.019 |
| GO:0048518 | positive regulation of biological process | 128 | 0.019 |
| GO:0031399 | regulation of protein modification process | 53 | 0.0202 |
| GO:0007264 | small GTPase mediated signal transduction | 29 | 0.0223 |
| GO:0051338 | regulation of transferase activity | 35 | 0.0223 |
| GO:0006996 | organelle organization | 82 | 0.0244 |
| GO:0010608 | posttranscriptional regulation of gene expression | 19 | 0.025 |
| GO:0044237 | cellular metabolic process | 192 | 0.0252 |
| GO:0000079 | regulation of cyclin-dependent protein serine/threonine kinase activity | 9 | 0.0285 |
| GO:0007049 | cell cycle | 46 | 0.0285 |
| GO:0032270 | positive regulation of cellular protein metabolic process | 45 | 0.0285 |
| GO:0038179 | neurotrophin signaling pathway | 17 | 0.0285 |
| GO:0071218 | cellular response to misfolded protein | 4 | 0.0285 |
| GO:0019538 | protein metabolic process | 105 | 0.0307 |
| GO:0048583 | regulation of response to stimulus | 91 | 0.0338 |
| GO:0045937 | positive regulation of phosphate metabolic process | 36 | 0.0342 |
| GO:0090083 | regulation of inclusion body assembly | 4 | 0.0342 |
| GO:0006897 | endocytosis | 23 | 0.0401 |
| GO:0035556 | intracellular signal transduction | 57 | 0.0401 |
| GO:0045860 | positive regulation of protein kinase activity | 21 | 0.0401 |
| GO:0051347 | positive regulation of transferase activity | 25 | 0.0401 |
| GO:0001932 | regulation of protein phosphorylation | 41 | 0.0455 |
| GO:0016192 | vesicle-mediated transport | 40 | 0.0471 |
| GO:0044764 | multi-organism cellular process | 29 | 0.0481 |
| GO:0042327 | positive regulation of phosphorylation | 32 | 0.0494 |

**Supplementary Table 8.6: GO output for HC vs MG1 from StringDB**

|  | | | | | | |  |  |  |  |
| --- | --- | --- | --- | --- | --- | --- | --- | --- | --- | --- |
| term_ID | description | frequency | plot_X | plot_Y | plot_size | log10 p-value | uniqueness | dispensability | representative | eliminated |
| GO:0008152 | metabolic process | 82.18% | -2.359 | 2.407 | 7.612 | 2.3385 | 0.993 | 0 | 8152 | 0 |
| GO:0009157 | deoxyribonucleoside monophosphate biosynthetic process | 0.12% | -1.068 | -5.821 | 4.759 | 0.4771 | 0.831 | 0 | 9157 | 0 |
| GO:0016192 | vesicle-mediated transport | 0.38% | -5.118 | 0.49 | 5.277 | 1.6021 | 0.938 | 0 | 16192 | 0 |
| GO:0090083 | regulation of inclusion body assembly | 0.00% | 5.322 | 4.59 | 2.587 | 0.6021 | 0.725 | 0 | 90083 | 0 |
| GO:0044238 | primary metabolic process | 51.93% | -1.201 | 5.265 | 7.412 | 2.2967 | 0.963 | 0.009 | 44238 | 0 |
| GO:0071218 | cellular response to misfolded protein | 0.00% | 6.675 | -4.549 | 2.734 | 0.6021 | 0.875 | 0.016 | 71218 | 0 |
| GO:0044764 | multi-organism cellular process | 2.06% | -3.06 | 5.978 | 6.01 | 1.4624 | 0.894 | 0.027 | 44764 | 0 |
| GO:0044403 | symbiosis, encompassing mutualism through parasitism | 1.78% | null | null | 5.948 | 1.5315 | 0.924 | 0.881 | 44764 | 1 |
| GO:0071704 | organic substance metabolic process | 56.18% | -0.949 | 7.107 | 7.446 | 2.3032 | 0.964 | 0.057 | 71704 | 0 |
| GO:0044237 | cellular metabolic process | 53.87% | -4.167 | 3.417 | 7.428 | 2.2833 | 0.923 | 0.063 | 44237 | 0 |
| GO:1903047 | mitotic cell cycle process | 0.08% | 2.807 | -6.997 | 4.582 | 1.5185 | 0.877 | 0.072 | 1903047 | 0 |
| GO:0007049 | cell cycle | 1.41% | -0.047 | -0.99 | 5.846 | 1.6628 | 0.905 | 0.091 | 7049 | 0 |
| GO:0044710 | single-organism metabolic process | 39.46% | -0.088 | 2.279 | 7.293 | 2.0719 | 0.931 | 0.109 | 44710 | 0 |
| GO:0006793 | phosphorus metabolic process | 16.89% | -2.481 | -2.037 | 6.924 | 1.8062 | 0.901 | 0.118 | 6793 | 0 |
| GO:0043094 | cellular metabolic compound salvage | 0.37% | -2.412 | -4.45 | 5.263 | 0.8451 | 0.905 | 0.147 | 43094 | 0 |
| GO:0010608 | posttranscriptional regulation of gene expression | 0.54% | 7.242 | 0.767 | 5.427 | 1.2788 | 0.659 | 0.195 | 10608 | 0 |
| GO:0019538 | protein metabolic process | 12.33% | 3.427 | -5.173 | 6.788 | 2.0212 | 0.889 | 0.205 | 19538 | 0 |
| GO:1901566 | organonitrogen compound biosynthetic process | 9.30% | -2.938 | -4.967 | 6.665 | 1.6021 | 0.915 | 0.208 | 1901566 | 0 |
| GO:0006897 | endocytosis | 0.20% | -4.531 | -0.171 | 4.998 | 1.3617 | 0.939 | 0.245 | 6897 | 0 |
| GO:0034613 | cellular protein localization | 0.67% | -5.061 | -0.586 | 5.523 | 1.6335 | 0.938 | 0.268 | 34613 | 0 |
| GO:0006796 | phosphate-containing compound metabolic process | 16.69% | -0.173 | -5.9 | 6.919 | 1.7924 | 0.82 | 0.346 | 6796 | 0 |
| GO:0051338 | regulation of transferase activity | 0.16% | 7.862 | 1.176 | 4.891 | 1.5441 | 0.607 | 0.351 | 51338 | 0 |
| GO:0050790 | regulation of catalytic activity | 0.65% | null | null | 5.51 | 1.8388 | 0.578 | 0.854 | 51338 | 1 |
| GO:0044093 | positive regulation of molecular function | 0.23% | null | null | 5.058 | 1.7482 | 0.625 | 0.757 | 51338 | 1 |
| GO:0031399 | regulation of protein modification process | 0.18% | 6.234 | 0.548 | 4.96 | 1.7243 | 0.572 | 0.366 | 31399 | 0 |
| GO:0035556 | intracellular signal transduction | 2.72% | 6.867 | -0.1 | 6.131 | 1.7559 | 0.595 | 0.374 | 35556 | 0 |
| GO:0048522 | positive regulation of cellular process | 0.43% | 7.994 | 1.809 | 5.329 | 2.0719 | 0.593 | 0.38 | 48522 | 0 |
| GO:0001934 | positive regulation of protein phosphorylation | 0.04% | null | null | 4.287 | 1.5185 | 0.493 | 0.923 | 48522 | 1 |
| GO:0045860 | positive regulation of protein kinase activity | 0.03% | null | null | 4.117 | 1.3222 | 0.451 | 0.951 | 48522 | 1 |
| GO:0032270 | positive regulation of cellular protein metabolic process | 0.11% | null | null | 4.718 | 1.6532 | 0.515 | 0.804 | 48522 | 1 |
| GO:0042327 | positive regulation of phosphorylation | 0.05% | null | null | 4.373 | 1.5051 | 0.515 | 0.973 | 48522 | 1 |
| GO:0045937 | positive regulation of phosphate metabolic process | 0.05% | null | null | 4.419 | 1.5563 | 0.522 | 0.992 | 48522 | 1 |
| GO:0031401 | positive regulation of protein modification process | 0.05% | null | null | 4.375 | 1.6232 | 0.518 | 0.936 | 48522 | 1 |
| GO:0048518 | positive regulation of biological process | 0.68% | 7.495 | 2.156 | 5.532 | 2.1072 | 0.699 | 0.385 | 48518 | 0 |
| GO:0065009 | regulation of molecular function | 0.84% | 6.936 | 1.904 | 5.619 | 1.9031 | 0.7 | 0.386 | 65009 | 0 |
| GO:0065003 | macromolecular complex assembly | 0.68% | 3.302 | 7.022 | 5.527 | 1.6335 | 0.829 | 0.496 | 65003 | 0 |
| GO:0071822 | protein complex subunit organization | 0.87% | null | null | 5.635 | 1.7324 | 0.832 | 0.923 | 65003 | 1 |
| GO:0048583 | regulation of response to stimulus | 0.69% | 7.771 | -0.003 | 5.535 | 1.959 | 0.676 | 0.516 | 48583 | 0 |
| GO:0038179 | neurotrophin signaling pathway | 0.00% | 7.182 | -1.72 | 3.04 | 1.2304 | 0.719 | 0.555 | 38179 | 0 |
| GO:0000278 | mitotic cell cycle | 0.09% | 3.211 | -7.167 | 4.637 | 1.5563 | 0.877 | 0.591 | 278 | 0 |
| GO:0007265 | Ras protein signal transduction | 0.10% | 7.587 | -0.912 | 4.703 | 1.2304 | 0.67 | 0.599 | 7265 | 0 |
| GO:0051128 | regulation of cellular component organization | 1.02% | 5.901 | 3.385 | 5.705 | 1.8261 | 0.599 | 0.629 | 51128 | 0 |
| GO:0006996 | organelle organization | 0.93% | 3.089 | 7.232 | 5.666 | 1.9138 | 0.817 | 0.651 | 6996 | 0 |
| GO:0007264 | small GTPase mediated signal transduction | 0.23% | 7.586 | -0.579 | 5.067 | 1.4624 | 0.654 | 0.651 | 7264 | 0 |
| GO:0043933 | macromolecular complex subunit organization | 1.06% | 3.01 | 7.036 | 5.723 | 1.8751 | 0.842 | 0.66 | 43933 | 0 |
| GO:0000079 | regulation of cyclin-dependent protein serine/threonine kinase activity | 0.02% | 5.374 | -2.128 | 4.08 | 0.9542 | 0.478 | 0.676 | 79 | 0 |
| GO:0001932 | regulation of protein phosphorylation | 0.17% | null | null | 4.916 | 1.6128 | 0.498 | 0.974 | 79 | 1 |
| GO:0045859 | regulation of protein kinase activity | 0.15% | null | null | 4.867 | 1.4771 | 0.442 | 0.881 | 79 | 1 |
| GO:0051347 | positive regulation of transferase activity | 0.03% | null | null | 4.168 | 1.3979 | 0.623 | 0.784 | 79 | 1 |
